# Exploring the relationship between admixture and genetic susceptibility to attention deficit hyperactivity disorder in two Latin American cohorts

**DOI:** 10.1038/s10038-024-01246-5

**Published:** 2024-05-07

**Authors:** Nicolás Garzón Rodríguez, Ignacio Briceño-Balcázar, Humberto Nicolini, José Jaime Martínez-Magaña, Alma D. Genis-Mendoza, Julio C. Flores-Lázaro, Jorge A. Villatoro Velázquez, Marycarmen Bustos Gamiño, Maria Elena Medina-Mora, Maria Fernanda Quiroz-Padilla

**Affiliations:** 1 Laboratorio de Bases Biológicas del Comportamiento, Facultad de Psicología, Universidad de La Sabana, Chía, Colombia; 2 Doctorado en Biociencias, Facultad de Ingeniería, Universidad de La Sabana, Chía, Colombia; 3 Laboratorio de Genética, Facultad de Medicina, Universidad de La Sabana, Chía, Colombia; 4 Laboratorio de Enfermedades Psiquiátricas, Neurodegenerativas y Adicciones, Instituto Nacional de Medicina Genómica, Secretaría de Salud, Mexico City, México; 5 Hospital Psiquiátrico Infantil Dr Juan N. Navarro, Mexico City, México; 6 Facultad de Psicología, Universidad Nacional Autónoma de México - UNAM, Mexico City, México; 7 Instituto Nacional de Psiquiatría Ramon de la Fuente Muñiz, Secretaría de Salud, Mexico City, México

**Keywords:** Risk factors, Human behaviour, ADHD, Genome-wide association studies

## Abstract

Contemporary research on the genomics of Attention Deficit Hyperactivity Disorder (ADHD) often underrepresents admixed populations of diverse genomic ancestries, such as Latin Americans. This study explores the relationship between admixture and genetic associations for ADHD in Colombian and Mexican cohorts. Some 546 participants in two groups, ADHD and Control, were genotyped with Infinium PsychArray®. Global ancestry levels were estimated using overall admixture proportions and principal component analysis, while local ancestry was determined using a method to estimate ancestral components along the genome. Genome-wide association analysis (GWAS) was conducted to identify significant associations. Differences between Colombia and Mexico were evaluated using appropriate statistical tests. 354 Single-nucleotide polymorphisms (SNPs) and Single-nucleotide variants (SNVs) related to some genes and intergenic regions exhibited suggestive significance (*p*-value < 5*10e−5) in the GWAS. None of the variants revealed genome-wide significance (*p*-value < 5*10e−8). The study identified a significant relationship between risk SNPs and the European component of admixture, notably observed in the *LOC105379109* gene. Despite differences in risk association loci, such as *FOXP2*, our findings suggest a possible homogeneity in genetic variation’s impact on ADHD between Colombian and Mexican populations. Current reference datasets for ADHD predominantly consist of samples with high European ancestry, underscoring the need for further research to enhance the representation of reference populations and improve the identification of ADHD risk traits in Latin Americans.

## Introduction

Attention Deficit Hyperactivity Disorder (ADHD) is a neurodevelopmental condition characterized by persistent inattention, hyperactivity, and impulsivity, which can affect performance in adulthood as well as in childhood [1]. The etiology of ADHD is complex, with patients showing heterogeneity in cognitive, behavioral, and biological aspects of the disorder [2]. Its worldwide prevalence in children is around 5% and in adults between 2.5–3% [3]. The reported prevalence of ADHD in Latin American countries varies depending on the type of study, diagnostic instruments used [4], and sample size [5]. Latin American populations tend to be highly diverse in terms of the cultural, social, and genetic factors that influence the disorder. In terms of genetic aspects, a high degree of admixture is a common feature both between and within countries [6]. This has promoted the idea that the genomes of Latin American populations are a mosaic of chromosomal fragments with ancestry of different origins [7], which could interfere with the effectiveness of state-of-the-art methods for mapping genetic risk loci [8]. The current research represents an opportunity to improve the associations of certain risk loci for populations with high genetic admixture [9].

An important problem related to admixture, which could potentially impact Genome-Wide Association Studies (GWAS) – the gold standard method for mapping associated loci to complex disorders – is the definition of ancestry and ethnicity in Latin American populations, where genetic findings are still very general and call our attention to the conceptual differences between the two terms. Ancestry has a high biological component that is explained by the line of descent in migration patterns and the geographical location of the population being studied [10, 11]. Conversely, ethnicity implies socialization processes that cause a group of people to share certain identities or cultural traits, such as nationality, language, religion, and/or social norms [10]. Recognizing the differences between ancestry and ethnicity can help us understand the interaction between genetic vulnerability and the complex environmental factors that shape ADHD risk. In this context, it is pertinent to distinguish between “global” ancestry, which presents a comprehensive overview of an individual’s average ancestral origin across the entire genome, and “local” ancestry, which allows for the detection of specific genomic segments that have experienced changes in ancestral composition over time [12].

Genomic research has yet failed to capture all the variation in Latin American populations, leading to weaknesses in addressing diversity when scanning genomes to find variants associated with disease traits [13]. Currently, these analyses are carried out with reference to datasets drawn from populations with predominantly European ancestry, leading to population biases in interpretation [13, 14]. Moreover, recent GWAS studies with large sample sizes have tended to under-represent Latin American populations [9]. The most recent meta-analysis of ADHD studies showed 27 significant GWAS loci for this disorder [15], but with only a small representation of Latin American individuals in the samples.

Among these countries, Colombia and Mexico have particularly diverse genetic backgrounds, with different profiles of Native American, European, and African ancestry [6]. The diversity accounted for by these populations in GWAS may thus be important for better characterizing the genetic influences on ADHD in these kinds of studies [16]. Hence, the present study aimed to analyze the effect of admixture and genetic associations for ADHD in two Latin American cohorts (Colombians and Mexicans) to better understand the genetics of ADHD in people from these two countries.

## Materials and methods

### Sample

We included 576 participants, with equal numbers of Colombian (*n* = 288) and Mexican (*n* = 288) descent. Colombian participants were recruited through private neuropsychological and psychiatric centers, as well as events in local universities. They were assigned to a diagnostic or control group by means of screening for ADHD and a neuropsychiatric interview. Those from Mexico were part of the subsample MxGDAR-Encodat [17] from the Mexican National Survey of Tobacco, Alcohol, and Drug Use [18], which found an ADHD prevalence of 1.4% based on a questionnaire used to assign participants to the ADHD group. The controls for both samples were selected if they did not present ADHD or any of the other mental illnesses screened (Major Depressive Disorder, Generalized Anxiety Disorder, Suicidality, Psychotic Disorders, Panic Disorder, and substance use disorders). For all participants, informed consent was obtained before the start of each study. The research received approval from the institutional ethics committees involved (Universidad de La Sabana, approval number 67-05/04/2018; Instituto Nacional de Medicina Genómica, approval number 01/2017/I; Instituto Nacional de Psiquiatría Ramón de la Fuente Muñiz, approval number CEI/C/083/2015). It was carried out in accordance with the latest version of the Declaration of Helsinki, the code of conduct of the American Psychological Association (1992) [19], and the relevant laws of Mexico and Colombia.

### Measurement tools

#### ADHD

A clinical diagnosis of ADHD was confirmed in the Colombian sample through a semi-structured neuropsychiatric interview, which explored the symptoms of different psychiatric illnesses based on the Diagnostic and Statistical Manual of Mental Disorders, fifth edition (DSM-V). In the Mexican sample, a questionnaire that inquired about the previous diagnosis of ADHD and other disorders helped to select the participants.

#### Genotyping

Genomic DNA was isolated from buccal epithelial samples through distinct protocols: a modified salting-out method [17], implemented with a commercial kit (Qiagen, Redwood City, CA, USA), was employed for the Mexican sample, while a customized version of a manual salting-out method was applied for Colombian cohort [20, 21]. Using the manufacturer’s protocol, genotyping was performed in the Instituto Nacional de Medicina Genómica de México with the commercial Infinium PsychArray Beadchip v1.3 (Illumina, San Diego, CA, USA). Fluorescent intensities were measured with the iScan (Illumina, San Diego, CA, USA), transformed to genotypes with the *GenomeStudio* software (Illumina, San Diego, CA, USA), and converted to *Plink* format files [22, 23].

Quality control of genotypes was performed in the *Plink* software, with the following criteria considered: variant calling greater than 95%, a minor allele frequency (MAF) greater than 5%, a Hardy–Weinberg equilibrium with *p* values < 0.05, and remotion of variants with A/T or G/C alleles to avoid the flip strand effect [24]. Filial relationships were calculated with the *King* software [25], and samples that showed association up to 2 degrees were eliminated. These filters left 546 participants for subsequent analysis (Supplementary Table 1). Single-nucleotide polymorphisms (SNPs) were then LD-pruned (PLINK1.9; --indep 50 5 2) for inclusion in a Principal Component Analysis (PCA) using the *PCAiR* package in R (version 4.3.1) and a maximum likelihood estimation of individual global admixture proportions of five ancestries using the *ADMIXTURE* software [26].

#### Imputation

We imputed the data following the TOPMed Imputation Server protocols [27]. The resulting files were filtered using an *R*^2^ INFO score threshold of 0.8 for imputed SNPs, MAF greater than 5%, and Hardy–Weinberg equilibrium with *p* values < 0.0005. We used the imputed data for downstream analysis.

### Statistical analysis

#### Principal component analysis (PCA)

**Fig. 1:**
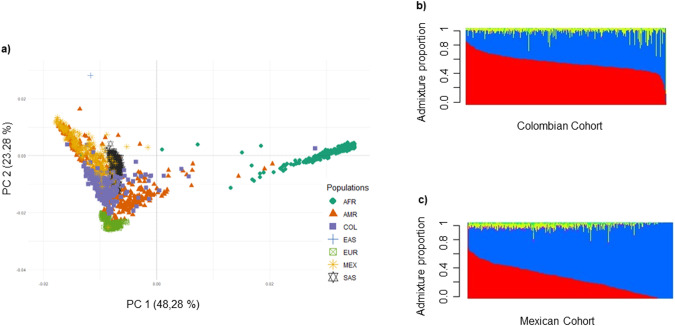
Genetic distribution in the Colombian and Mexican samples. **a** Principal Component Analysis of the samples with the reference populations. AFR African Ancestry, AMR American Ancestry, COL Colombia, EAS East Asian Ancestry, EUR European Ancestry, MEX Mexico, and SAS South Asian Ancestry. **b** Distribution of ancestry proportions in the Colombian cohort. **c** Distribution of ancestry proportions in the Mexican cohort. “Red” = European Ancestry, “Blue” = American Ancestry, “Yellow” = African Ancestry, “Green” = South Asian Ancestry, “Purple” = East Asian Ancestry

To identify the main directions of variation in the genetic data of the Colombian and Mexican samples with respect to reference populations, we performed a PCA with the *PC-AiR* package in R. For this, the reference populations of the 1000 Genomes Project – Phase 3 [28] were used and merged with our genotypes following the procedure previously cited [24, 29]. The first two principal components were chosen, and a graph was made in *RStudio*^®^ to facilitate visualization of the results (Fig. 1a).

#### Global ancestry

Once the data from the microarray reader were extracted and converted, and the quality controls were applied, an analysis was performed with the *ADMIXTURE* software [26] to determine the global ancestry structure in the Colombian and Mexican samples. We performed cross-validation, which confirmed that the probable appropriate number of populations to calculate in an unsupervised analysis was five (Supplementary Fig. 1). Stacked bar charts summarizing the contribution of each of the five reference populations (European, East Asian, American, South Asian, and African) to the ancestry of the study samples are presented (Fig. 1b, c).

#### Population differentiation at known GWAS ADHD loci

Wright’s Fst was performed to assess the genetic variation among populations, specifically in the GWAS ADHD loci [15]. This analysis was conducted considering the 821 SNPs previously identified as risk factors for ADHD which previously indicated a *p* < 5*10e−8 in a large meta-analysis [15]. The test provides a comprehensive understanding of genetic differentiation, which can uncover distinct patterns acting on SNPs identified as being linked with ADHD.

#### Differential effects in risk at known GWAS ADHD loci between Colombia and Mexico

The Mantel–Haenszel test was conducted to examine whether there were differences in the association of specific polymorphisms with ADHD between the Colombian and Mexican samples. This test serves as a valuable mechanism to discern if there are distinct effects of the examined 821 SNPs on ADHD susceptibility in different cohorts (*p*-value threshold for significance: <6.0901e−05). Furthermore, we used the Breslow–Day test to assess the homogeneity of this association across subgroups. This contributed to understanding whether the identified SNPs exhibited consistent or disparate impacts across the two populations.

#### Genetic associations at known GWAS ADHD loci in Colombia and Mexico

Logistic regression was employed to investigate potential associations between the previous genome-wide ADHD-linked variants (821 SNPs) [15] and ADHD diagnosis. The regression models were designed to account for potential confounding effects by incorporating covariates such as age, sex, and the first two principal components derived from the PCA (*p*-value threshold for significance: <6.0901e−05). This approach allowed for the exploration of relationships between genetic variants and ADHD susceptibility while controlling for relevant demographic factors.

#### Genetic associations at known GWAS ADHD loci with admixture proportions

With the goal of investigating possible associations between genetic variants and global admixture proportions, aiming to elucidate the intricate interplay between ancestry and genetic susceptibility in the context of ADHD, we performed multiple linear regression analyses. In these, we used each of the global admixture proportions as the dependent variables, while the identified set of 821 SNPs served as independent predictors (*p*-value threshold for significance: <1.2180e−05).

#### Genome-wide association study (GWAS)

After quality control and imputation, independent association analyses were conducted in each population using the *Plink* software [22]. The analysis includes age, sex, and the first two principal components as covariates. We meta-analyzed the data using *METAL* [30]. The general findings are presented and discussed in the results (suggestive significance = −log10(1e−5), genome-wide significance = -log10(5e–8)). For the GWAS, the suggestive significance at 5*10e−5, the sample size (Cases = 202, Controls = 344), the relative risk of the genotype at 1.5, and the disease allele frequency at 0.5, allowed us to have an expected power at 0.314. We estimate power using the Gas Power Calculator from the University of Michigan [31]. The power estimation graph is in Supplementary Fig. 2.

#### Local-ancestry aware GWAS

Conducting local-ancestry aware GWAS between Colombia and Mexico is relevant to assessing the intricate genetic diversity within these geographic regions. This method allows for detecting the genetic ancestry background of the individuals in specific genomic segments. *RFMix* [14] was used to infer local ancestry in our populations based on the reference populations of the 1000 Genomes Project – Phase 3 [28]. Further, we implemented a local-ancestry aware GWAS using Tractor [8] to perform a genetic association of each genetic variant adjusting for the ancestral genetic background in the specific genomic segments. Using a regression model that considers local ancestry, Tractor provides accurate ancestry-specific effect-size estimates and *P* values for each ancestral background. Age, sex, and the two principal components were also included as covariates; a comparison with the GWAS is presented in Fig. 4. The results were plotted with the *fastman* R package.

## Results

### Principal component analysis (PCA)

We performed PCA of our samples projected to the 1000 Genomes reference panel to explore the continental distribution. The PCA displayed a clear spectrum of variation between the two cohorts studied (Fig. 1a), with principal components 1 and 2 accounting for 71.56% of the total variability. Mexican and Colombian individuals exhibited distinct distributions in the principal component space, with the Mexican sample grouped in the upper quadrant of the graph, and the Colombian sample in the lower quadrant. In addition, some overlap of individuals was observed in the central region of the graph. These patterns of genetic variation suggest some genetic similarity between populations, but also differences that could be due to population divergence over time.

### Global ancestry

Admixture analyses were performed to examine differences in proportions of global ancestry between Mexican and Colombian individuals. These showed a higher contribution of European and American ancestry in both populations (Fig. 1b, c), but with significant differences in all proportions of admixture between the samples. While the Mexican sample had a proportion of American ancestry of 66.71%, Colombia had only 38.38% (*t* = 21.279, *p* = 2.2000e−16); in the latter sample, there was a greater contribution of European (55.29%; Mexico 26.96%; *t* = 23.565, *p* = 2.2000e−16) and African (5.78%; Mexico 4.10%; *t* = 3.676, *p* = 0.0003) ancestry. Mexico also had a greater ancestral descent from the Asian continent, both from the eastern (EAS; 1.12%; *t* = 10.184, *p* = 2.2000e−16) and southern (SAS; 1.12%; *t* = 10.483, *p* = 2.2000e−16) regions, compared to Colombia (EAS = 0.01%; SAS = 0.36%). The complex ancestry structure in these samples highlights the importance of describing admixture in genetic studies of Latin American populations.

**Fig. 2:**
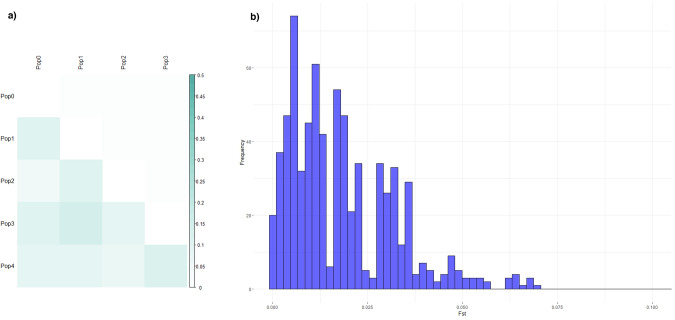
Genetic differences in the Colombian and Mexican samples. **a** Matrix of Fst estimated in ADMIXTURE. The color intensity represents the degree of genetic divergence. Pop0 European Ancestry, Pop1 American Ancestry, Pop2 South Asian Ancestry, Pop3 African Ancestry, Pop4 East Asian Ancestry. **b** Distribution of Fst values across Colombian and Mexican cohorts

Fst values resulting from the Admixture analysis reflected the extent of genetic differences between the five populations (Fig. 2a). Individuals with a high European component presented greater distance from the American (*Fst* = 0.116) and African (*Fst* = 0.106) populations, showing greater proximity to South (*Fst* = 0.045) and East (*Fst* = 0.091) Asians. American ancestry also showed distance from South Asian (*Fst* = 0.102) and African (*Fst* = 0.155) components, being closer to the East Asian component (*Fst* = 0.094). South Asians showed proximity to African (*Fst* = 0.099) and East Asian (*Fst* = 0.064) ancestry. However, East Asia distanced itself from the African component (*Fst* = 0.123).

### Population differentiation at known GWAS ADHD loci

To explore potential differentiation previously described in the GWAS ADHD loci, we calculated Fst values between Mexico and Colombia for each variant. Among the 821 SNPs previously associated with ADHD, only rs7458242, mapped to the *FOXP2* gene, exhibited a high Fst value (*Fst* = *3.76*), which suggests pronounced genetic differentiation between the Colombian and Mexican cohorts in relation to this SNP. Additionally, a small subset of 23 SNPs (2.80%) demonstrated moderate differentiation, with Fst values ranging from 0.05 to 0.07. The majority of SNPs (85.02%) exhibited lower Fst values (ranging from 0.0002 to 0.05), indicating relatively no differentiation between the two populations (Fig. 2b). A minor proportion of 99 SNPs (12.06%) displayed Fst values less than 0 (see Supplementary Table 2).

### Differential effects in risk at known GWAS ADHD loci between Colombia and Mexico

In order to assess potential variation in the effects of previously identified ADHD risk loci, we performed Mantel–Haenszel and Breslow–Day tests between countries. The Mantel–Haenszel test identified a total of 44 genetic variants associated with ADHD, with possible allelic differences between Colombia and Mexico when comparing the diagnosis and control groups (however, none of these passed Bonferroni corrections for multiple tests, all *p* > 6.0901e−05). Nevertheless, in the Breslow–Day test, we detected that only two (rs72854462 and rs1438898 mapped to *TEX41* gene) of the 821 tested SNPs were nominally significant (*p* < 0.05), signaling heterogeneity in the odds ratios (OR) of these variants between Mexico and Colombia (see Supplementary Table 3). Our results suggest that the genetic effects of previously known ADHD GWAS loci are fairly homogeneous between the Colombian and Mexican populations.

### Genetic associations at known GWAS ADHD loci in Colombia and Mexico

Genetic associations of the significant SNPs previously identified in ADHD GWAS were computed by logistic regression, adjusting for age, sex, and two principal components of genetic ancestry. Logistic regression analyses showed that 71 of the 821 SNPs scanned had nominal associations with ADHD in our samples (see Supplementary Table 4). The top ten associated variants were located in the *PTPRF* gene. However, none of these associations passed Bonferroni correction for multiple testing (all *p* > 6.0901e−05; see Table 1).

**Table 1:** Main associations at known GWAS ADHD loci in the Colombian and Mexican cohorts

CHR	RSID	BP	A1	A2	MAF	OR	STAT	*P*
1	rs673253	43596483	T	C	0.210	16.870	31.680	0.0015
1	rs2842189	43541977	T	C	0.223	17.520	31.260	0.0018
1	rs17371903	43605020	G	A	0.189	16.600	30.750	0.0021
1	rs12405972	43631767	T	G	0.178	16.610	30.400	0.0024
1	rs61769649	43632835	A	G	0.179	16.520	30.120	0.0026
1	rs2842185	43554060	C	T	0.188	16.450	29.700	0.0030
1	rs2842178	43556356	A	G	0.188	16.450	29.700	0.0030
1	rs11210869	43560369	G	A	0.183	16.420	29.690	0.0030
1	rs61769611	43563242	A	G	0.185	16.310	29.320	0.0034
1	rs596522	43600753	A	C	0.190	16.170	29.220	0.0035

*CHR* Chromosome, *RSID* Variant ID from dbSNP, *BP* Position in GRCh38, *A1* Reference Allele, *A2* Alternative Allele, *MAF* Minor Allele Frequency, *OR* Odds Ratio, *STAT* Regression Coefficient, *P* *p*-value

### Genetic associations at known GWAS ADHD loci with admixture proportions

**Fig. 3:**
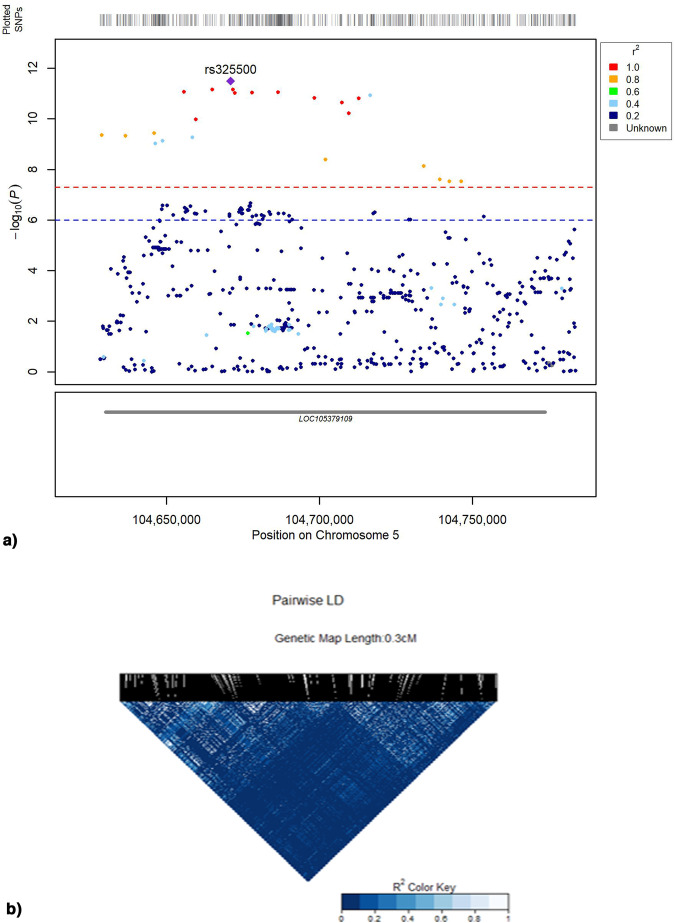
Associations of the LOC105379109 gene with European ancestry. **a** Locus Zoom plot representing the gene LOC105379109 on chromosome 5. The y-axis represents the p-value association between each variant and the European admixture proportions, and the colors represent the R² between the top SNP (rs32500) and the other SNPs calculated in our samples. It provides insights into the potential significance and linkage disequilibrium patterns within this locus regarding the European admixture component. **b** Linkage disequilibrium plot with recombination rates in the LOC105379109 gene on chromosome 5. The genetic distances in centimorgans (cM) were calculated according to the GRCh38 genetic map

We further explored whether previously identified GWAS loci for ADHD could be associated with the global admixture proportions. We identified a total of 149 significant regressions (after Bonferroni correction, *p* < 1.2180e−05) concentrated within the European component of admixture, where *LOC105379109* showed the lowest p-values. The strongest association was found for rs325500; therefore, nearby SNPs were extracted and located based on their linkage disequilibrium (LD), and their variance was explained regarding the reference SNPs, also according to the significance of their associations with European proportions (Fig. 3a, b). This variant was found to have similar association values and similarly high LD compared with 11 other variants within the same gene (rs325501, rs416223, rs325481, rs325502, rs21126, rs396755, rs325528, rs410915, rs325523, rs325521). Collectively, these findings underscore that the influence of these SNPs on ADHD risk may be related to the component of European ancestry present in our samples (for further details, see Supplementary Table 5). We present the functional annotation of the 821 scanned SNPs based on RegulomeDB in Supplementary Table 6, where most of the SNPs analyzed have been determined to be Quantitative Trait Loci (QTLs).

### Local ancestry and GWAS

**Fig. 4:**
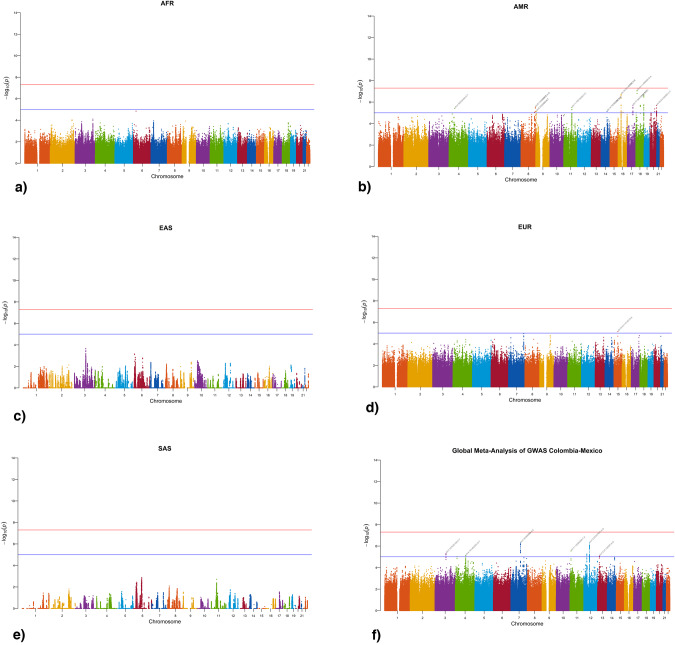
ADHD associations at the genome-wide level. **a** Manhattan plot of the local-ancestry aware GWAS of the African ancestry tracks. **b** Manhattan plot of the local-ancestry aware GWAS of American ancestry tracks. 64 variants showed suggestive significance (*p*-value < 5e−8). **c** Manhattan plot of the local-ancestry aware GWAS of East Asian ancestry. **d** Manhattan plot of the local-ancestry aware GWAS of European ancestry. 12 variants showed suggestive significance (*p*-value < 5e−8). **e** Manhattan plot of the local-ancestry aware GWAS of South Asian ancestry. **f** Global meta-analysis of GWAS from Colombian and Mexican populations. The association analysis was performed in *Plink*, adjusting by age, sex, and two principal components. 278 variants showed suggestive significance (*p*-value < 5e−8)

For both methods, the local-ancestry aware GWAS produced by Tractor and the global meta-analysis of GWAS obtained with METAL, we used 7,547,123 SNPs following stringent quality control measures after the imputation process. Within the local-ancestry aware GWAS, 76 variants showed suggestive significance (*p*-value < 5*10e−5) for the American (Fig. 4b) and European (Fig. 4d) components (Supplementary Table 7). Regarding global meta-analysis of GWAS, 278 variants showed a suggestive significance in the association with ADHD (Fig. 4f) (Supplementary Table 8). These results are related to the *SBF2*, *OR6C76*, *TMTC1*, *ALG5*, *LDB2*, *CCDC88C*, *GRM8*, *SMAD9*, *DUBR*, *RORA*, *LOC105379030*, *PTPRE*, *LINC02267*, *RBM20*, *TNC*, *PCDH7*, *LOC101928663*, *CNTNAP2*, *ATP6V0D2*, *LOC124902110*, *VTI1A*, *FGF14*, *FAM234A*, *BANP*, *EMID1* genes and other intergenic regions in chromosomes 3, 4, 5, 6, 7, 8, 9, 10, 11, 12, 13, 14, 15, 18, 20, 22. None of the variants revealed genome-wide significance (*p*-value < 5*10e−8) either for the local-ancestry aware GWAS or for the global meta-analysis of GWAS. The qqplots for all the analyses are available in Supplementary Fig. 3.

## Discussion

GWAS researchers continue to overlook a huge proportion of human genetic diversity, especially in Latin Americans [13]. Among Latin groups, ancestry differs both between and within countries across Central and South America, because of differences in the pre-Hispanic stage, the extent of colonization by European populations, and the degree of involvement in the trans-Atlantic slave trade [6, 32]. This work has important implications for understanding the contribution of admixture to the genetic liability of ADHD in two Latin American populations: Mexicans and Colombians. Mexico City and Bogotá are large urban areas, where many mixed-race people live, which facilitates the study of genetic variability within and between individuals, information that can improve our knowledge of phenotypic traits associated with disorders such as ADHD. Our admixture results are consistent with other studies of these countries where the proportions of ancestry maintain similar trends [6, 32].

Supporting the differences in admixture proportions between Colombia and Mexico, we found a potential differentiation in genetic variants of the *FOXP2* gene, suggesting that this process could contribute to a distinct genetic makeup underpinning ADHD susceptibility in these populations. Considering the role of the *FOXP2* gene in neurodevelopmental pathways, involving aspects such as linguistic and speech functionalities [33], its presence within the genetic context of ADHD offers a route for delving into the complex interrelationship between genetic elements and neural mechanisms [34]. This gene codes for a transcription factor with a DNA-binding *forkhead* domain and helps to regulate the expression of various genes during embryonic development [35]. Furthermore, *FOXP2* has been characterized as of high importance at an evolutionary level, aiding in distinguishing humans from other primates in terms of brain plasticity and organization [36] and linguistic processing. There are risky variants in this gene that have possible functional effects associated with ADHD outcomes, such as hyperactivity symptoms, impulsive personality traits, and differentiated patterns in cortical brain structures [37].

Even when we identified some genetic variants with differentiation between both populations, we detected a potential homogeneity in the OR of SNPs at loci previously associated with ADHD in GWAS, proposing a possible shared impact of genetic markers on the genetic risk for ADHD in Colombian and Mexican populations, transcending the demographic divergences between these two Latin American countries. Nevertheless, we found a suggestive differential effect of rs72854462 and rs1438898 variants in the *TEX41* gene between the populations. The *testis expressed 41* (*TEX41*) gene responds to a non-protein coding RNA that also participates in processes of transcriptional regulation [38]. Even though it is a relative novelty in the context of ADHD research, *TEX41* has been associated with fundamental cellular processes, including DNA repair and cell cycle regulation [39]. These functions, often crucial to maintaining genomic stability and correct neuronal development, could be related to the etiology of ADHD [40].

Notably, we found a potential replication of the association of the *PTPRF* gene with ADHD in our Colombian and Mexican samples. The *PTPRF* gene encodes a receptor-like protein, tyrosine phosphatase, which participates in neural development and synaptic plasticity [41]. This is due to its potential role in axon guidance during synapse formation [42], which has been suggested as a relevant factor in the biological antecedents of ADHD [34]. Such findings shed light on a potential link between *PTPRF* and ADHD in our samples, suggesting that genetic variations in this gene could impact neural circuits and cognitive processes associated with the disorder [34].

Considering the relationship between the variants previously associated with ADHD in GWAS studies and the component of European ancestry, it is possible that the risk that these SNPs represent is related to the amount of European ancestry in the study samples. The identification of *LOC105379109* as a focal point in this complex network opens avenues for exploration into its specific function and its possible contribution to neurobiological mechanisms associated with ADHD. This also highlights the importance of considering the differences in the ancestral contribution of Latin American populations compared to European populations, which usually provide the reference datasets for genetic analysis [13, 14]. Controlling levels of European ancestry in future research will allow more precise analyses of SNPs that affect the risk of having ADHD, avoiding population bias in the interpretation of results. The application of local-ancestry aware GWAS and global meta-analysis of GWAS revealed 354 genetic variants with suggestive significance *p* < 5 * 10e−05 (as described in Supplementary Tables 7 and 8), hinting at new insights into the genetic contribution to ADHD in the study populations.

One limitation of the study is the type and size of sampling since it was not completely representative of the Colombian and Mexican populations with and without ADHD. Consequently, our statistical power is restricted to 31%, reducing the study’s capacity to extrapolate findings to a wider population. Additionally, in the phenotypic evaluation of ADHD, there were differences that could affect the interpretation of the comparison tests between both populations. This weakens our ability to generalize from the results, but also invites us to continue expanding our samples and to build on the productive cooperation we have established between researchers from different Latin American countries.

In conclusion, although there are differences between the Colombian and Mexican samples, the results showed that these two populations could present high homogeneity in the magnitude of the effect of variants previously associated with ADHD. Nevertheless, we need further studies to better characterize the genetic mechanisms underlying ADHD in Latin American populations. Controlling for ancestry in ADHD association studies will improve our knowledge about genetic variability in Latin American (and other) populations with this disorder. Ongoing genomic research that fosters collaborations, like the one presented here, is needed to strengthen and increase the sample size for identifying genetic markers associated with ADHD.

### Supplementary information

Supplementary Data
