# Supplementary material for: Exploring the relationship between admixture and genetic susceptibility to attention deficit hyperactivity disorder in two Latin American cohorts
Source: J Hum Genet. 2024 May 7;69(8):373–80. doi: 10.1038/s10038-024-01246-5 (PMC11269173; doi:10.1038/s10038-024-01246-5)
Supplement: Supplementary file 1 — Supplementary Data [file 10038_2024_1246_MOESM1_ESM.pdf]

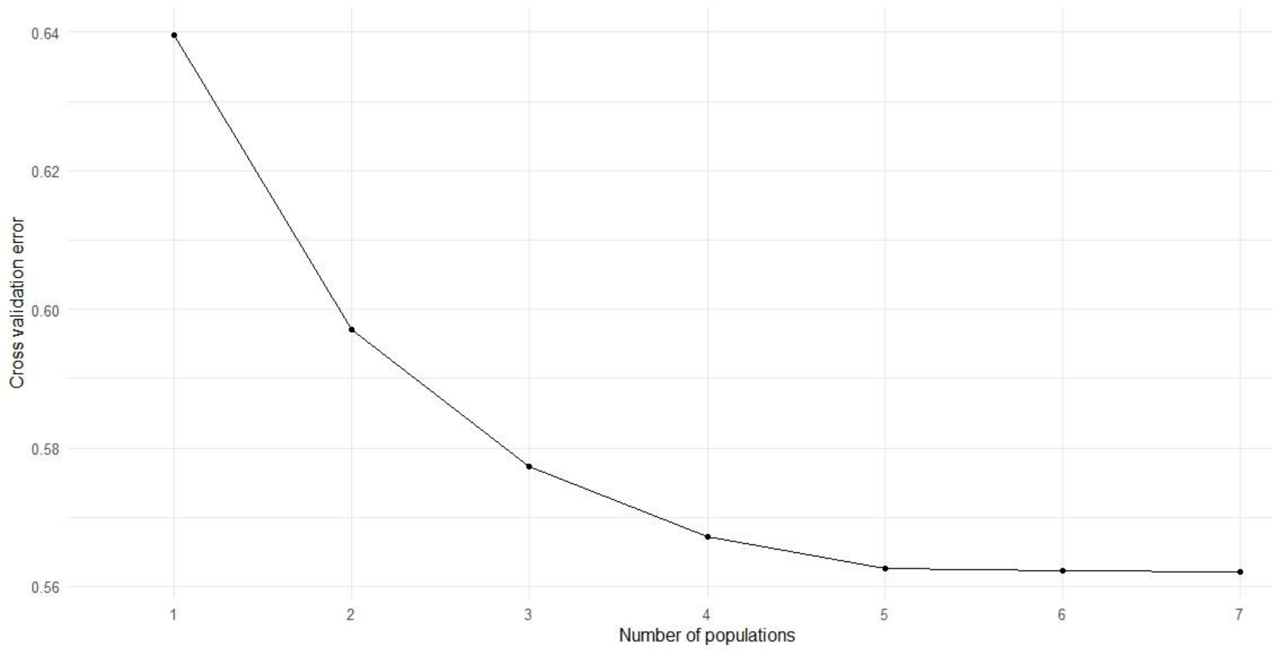

Supplementary Figure 1. **Cross-validation of the number of populations for global admixture analyses**

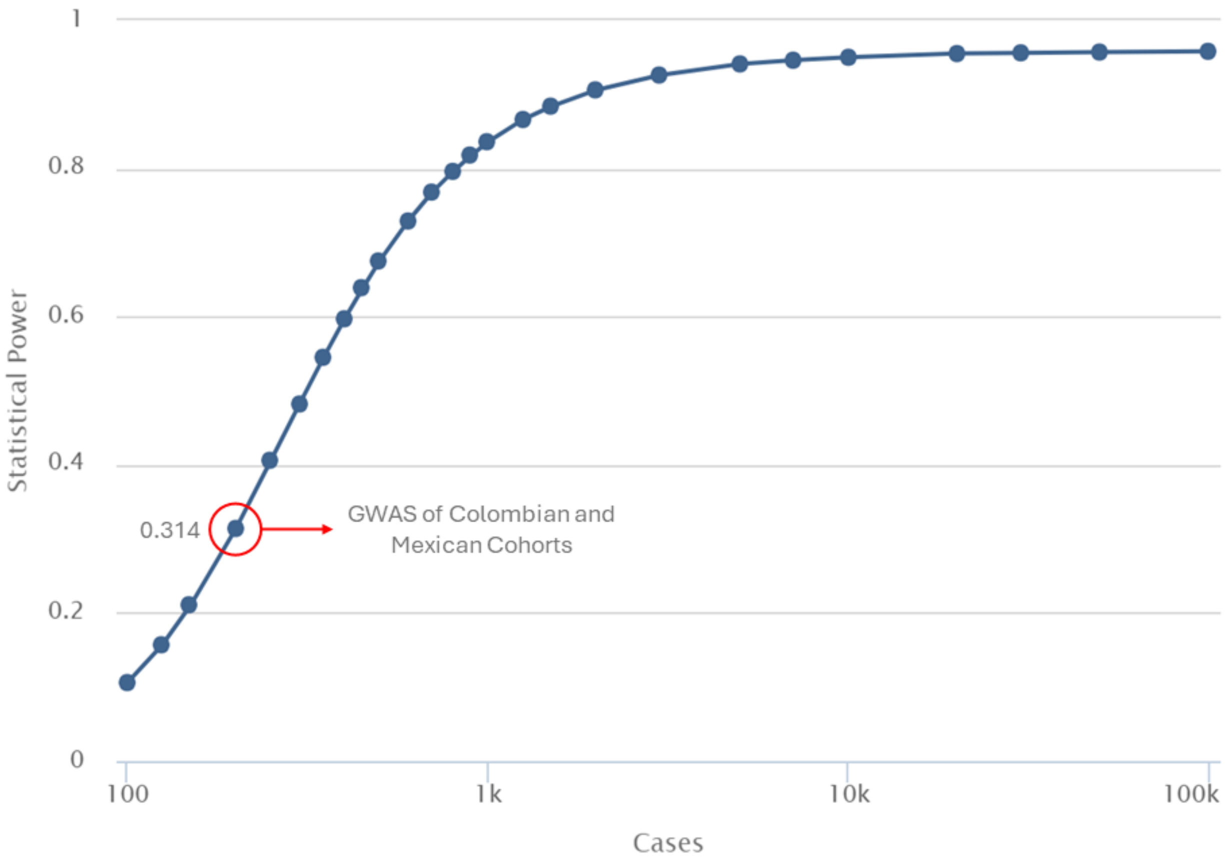

Supplementary Figure 2. **Power estimation graph.** The power was calculated for a suggestive significance at 5*10e-5, the sample size (Cases = 202, Controls = 344), the relative risk of the genotype at 1.5, and the disease allele frequency at 0.5 giving us a power of 0.314.

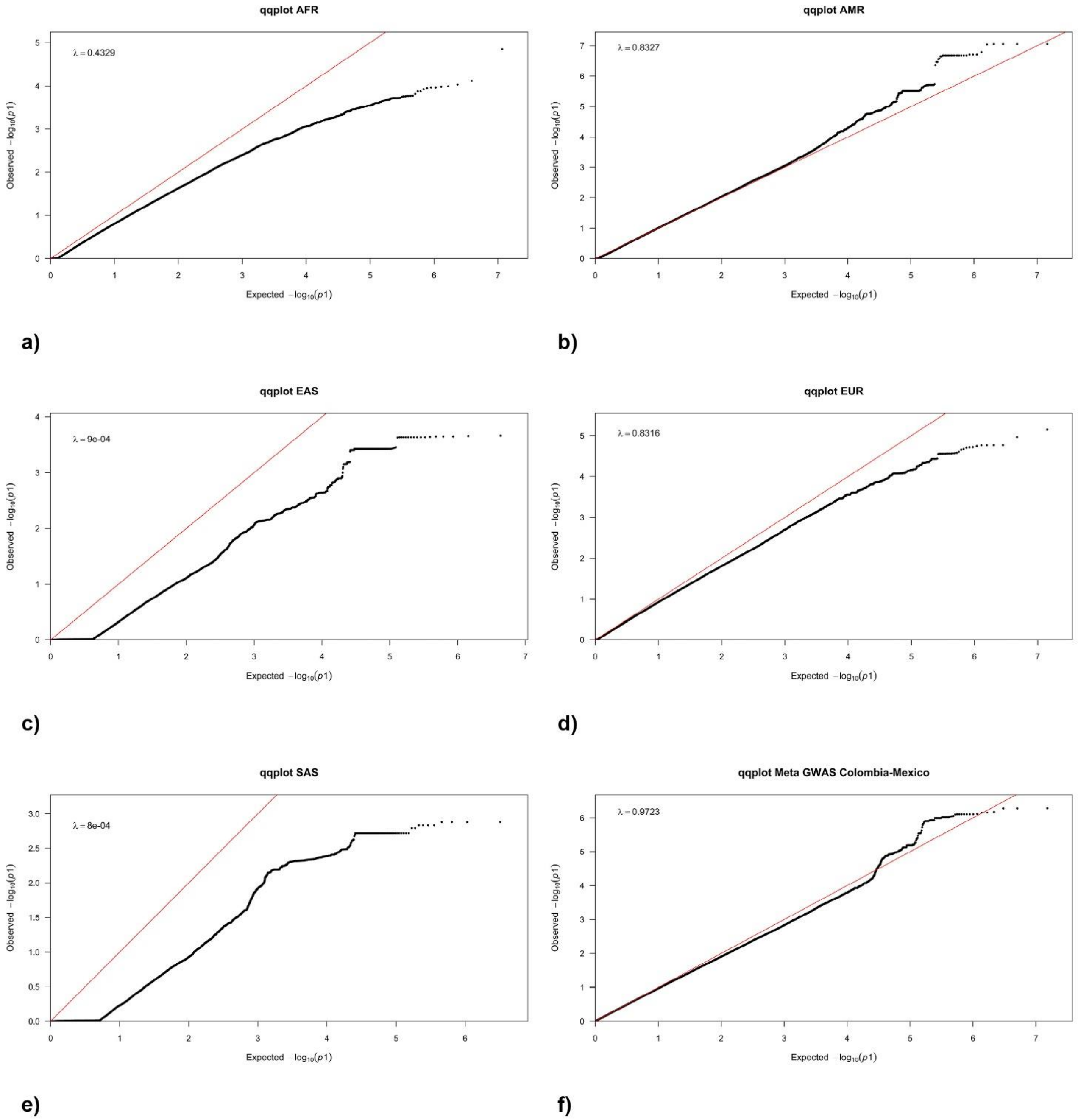

Supplementary Figure 3. **a)** qqplot of the local-ancestry aware GWAS of the African ancestry tracks. **b)** qqplot of the local-ancestry aware GWAS of American ancestry tracks. **c)** qqplot of the local-ancestry aware GWAS of East Asian ancestry. **d)** qqplot of the local-ancestry aware GWAS of European ancestry. **e)** qqplot of the local-ancestry aware GWAS of South Asian ancestry. **f)** qqplot of the Global meta-analysis of GWAS from Colombian and Mexican populations.

Page	Table	Title
ST1	Supplementary Table 1.	Demographic characteristics of the Colombian and Mexican cohorts.
ST2	Supplementary Table 2.	Genetic variation among populations with Wright's Fst
ST3	Supplementary Table 3.	Differences in specific polymorphisms with ADHD (Breslow-Day and Mantel-Haenszel test)
ST4	Supplementary Table 4.	Potential associations between the previous genome-wide ADHD-linked variants and ADHD diagnosis
ST5	Supplementary Table 5.	Possible associations between genetic variants and admixture proportions
ST6	Supplementary Table 6.	Functional annotation of the previous genome-wide ADHD-linked variants
ST7	Supplementary Table 7.	Potential associations in local-ancestry aware GWAS of ADHD in Colombian and Mexican cohorts
ST8	Supplementary Table 8.	Potential associations in global meta-analysis of GWAS for ADHD in Colombian and Mexican cohorts

Supplementary Table 1.

	Group	
	ADHD	Control
Colombia		
n	162	104
Man	67	54
Woman	95	50
Mean age (SD)	21,26 (2,45)	21,64 (2,36)
Mexico		
n	40	240
Man	25	121
Woman	15	119
Mean age (SD)	25,08 (12,58)	35,52 (14,87)

Supplementary Table 2.

CHR	SNP	RSID	POS	NMISS	FST
7	chr7:114544043:G:A	rs7458242	114544043	505	3,76E+00
5	chr5:104655775:C:A	rs416223	104655775	524	0,06892
5	chr5:104665051:G:A	rs325481	104665051	523	0,0684225
5	chr5:104678081:G:A	rs21126	104678081	522	0,0673757
5	chr5:104671732:G:C	rs325501	104671732	523	0,0669403
5	chr5:104670966:A:G	rs325500	104670966	523	0,0654696
5	chr5:104672432:G:A	rs325502	104672432	520	0,0648321
5	chr5:104686538:C:G	rs396755	104686538	526	0,0645571
1	chr1:43604361:A:G	rs530373	43604361	488	0,0635677
5	chr5:104712889:C:G	rs325528	104712889	528	0,0632577
5	chr5:104698439:C:T	rs410915	104698439	529	0,0620159
5	chr5:104707455:C:T	rs325521	104707455	529	0,0620159
1	chr1:43830767:C:G	rs3011216	43830767	471	0,0618112
5	chr5:104709685:C:A	rs325523	104709685	546	0,0567635
5	chr5:104659667:A:G	rs325485	104659667	546	0,0559088
5	chr5:104620815:C:G	rs171697	104620815	537	0,0548147
5	chr5:104636656:G:A	rs30266	104636656	537	0,0548147
5	chr5:104646025:T:G	rs40465	104646025	534	0,054793
5	chr5:104628884:G:A	rs77960	104628884	537	0,053435
1	chr1:43541977:T:C	rs2842189	43541977	442	0,053313
5	chr5:104619100:T:A	rs173823	104619100	537	0,0520177
7	chr7:114571857:G:A	rs2189012	114571857	515	0,0516829
5	chr5:104612267:T:C	rs2431108	104612267	538	0,0506636
7	chr7:114564074:T:A	rs7797335	114564074	518	0,0503734
1	chr1:43548609:T:C	rs2842188	43548609	444	0,0495327
5	chr5:104702059:G:A	rs12055234	104702059	538	0,0490067
1	chr1:43549278:T:C	rs2842187	43549278	443	0,0489348
5	chr5:104739429:T:C	rs6421926	104739429	519	0,0486248
20	chr20:21195965:C:T	rs2144782	21195965	491	0,0481494
5	chr5:104742532:A:C	rs60271	104742532	520	0,0479387
5	chr5:104746478:A:C	rs323509	104746478	520	0,0479387
5	chr5:104734216:A:G	rs161645	104734216	544	0,0472143
7	chr7:114578527:C:T	rs1229762	114578527	517	0,0471613
7	chr7:114583668:C:A	rs1229761	114583668	517	0,0471613
7	chr7:114584108:A:G	rs1229760	114584108	517	0,0471613
1	chr1:43554060:C:T	rs2842185	43554060	528	0,0470758
1	chr1:43556356:A:G	rs2842178	43556356	528	0,0470758
1	chr1:43543362:C:T	rs2782640	43543362	446	0,0465408
1	chr1:43549154:A:G	rs2819335	43549154	444	0,045908
1	chr1:43548902:A:T	rs2819333	43548902	446	0,0458051
1	chr1:43560369:A:G	rs11210869	43560369	535	0,0449017
7	chr7:114518899:G:A	rs9969232	114518899	521	0,0447786
1	chr1:43546066:G:A	rs951740	43546066	447	0,0429159
1	chr1:43550138:T:C	rs2819336	43550138	441	0,0425887
20	chr20:21189014:G:A	rs57940584	21189014	522	0,0421243
1	chr1:43549064:C:T	rs2819334	43549064	448	0,0418711
1	chr1:43563242:G:A	rs61769611	43563242	537	0,0411236

1	chr1:43682946:A:G	rs4660740	43682946	528	0,0408726
7	chr7:114384998:A:G	rs12537376	114384998	536	0,0406616
1	chr1:43566122:G:C	rs11586016	43566122	536	0,0401064
7	chr7:114381815:T:C	rs66571810	114381815	538	0,0398363
7	chr7:114470513:C:T	rs4727799	114470513	546	0,0395833
1	chr1:43574179:A:G	rs2842171	43574179	536	0,0393273
20	chr20:21187755:C:T	rs8120293	21187755	521	0,0391925
1	chr1:43569801:G:A	rs943513	43569801	534	0,0390012
20	chr20:21194910:T:C	rs879064	21194910	522	0,0388437
1	chr1:43686240:C:T	rs113688544	43686240	529	0,0382866
20	chr20:21187721:G:T	rs8120377	21187721	522	0,0377016
20	chr20:21190532:C:T	rs765585	21190532	522	0,0377016
1	chr1:43705540:C:T	rs56319043	43705540	530	0,0370819
11	chr11:28655291:A:G	rs4923554	28655291	545	0,0363599
1	chr1:43570414:G:A	rs1887402	43570414	535	0,0360835
5	chr5:88551455:T:A	rs12653396	88551455	480	0,0359493
1	chr1:43579857:G:A	rs1808730	43579857	540	0,0357677
1	chr1:43583485:G:A	rs3791134	43583485	540	0,0357677
1	chr1:43585959:G:A	rs61769642	43585959	540	0,0357677
1	chr1:43586100:T:C	rs3791139	43586100	540	0,0357677
1	chr1:43605020:A:G	rs17371903	43605020	540	0,0357562
11	chr11:28648922:G:A	rs7105480	28648922	538	0,0356672
11	chr11:28654958:T:C	rs10835389	28654958	538	0,0356672
11	chr11:28655314:A:G	rs4923555	28655314	538	0,0356672
11	chr11:28657323:A:G	rs10767739	28657323	538	0,0356672
11	chr11:28626638:C:T	rs7931459	28626638	529	0,0355959
11	chr11:28626783:A:T	rs7931585	28626783	529	0,0355959
11	chr11:28626831:G:C	rs7931884	28626831	529	0,0355959
11	chr11:28526196:C:T	rs7935201	28526196	501	0,0353726
11	chr11:28526682:A:C	rs990845	28526682	501	0,0353726
11	chr11:28659176:C:A	rs7947218	28659176	537	0,0352903
11	chr11:28634517:A:T	rs7942078	28634517	537	0,0352657
11	chr11:28634687:C:A	rs7942417	28634687	537	0,0352657
11	chr11:28644991:C:T	rs7933262	28644991	537	0,0352657
11	chr11:28646737:A:G	rs7481939	28646737	537	0,0352657
11	chr11:28650082:G:C	rs10835387	28650082	537	0,0352657
7	chr7:114608796:A:G	rs12705966	114608796	526	0,0352385
11	chr11:28662674:G:A	rs7934432	28662674	537	0,0349203
11	chr11:28664505:C:T	rs2356009	28664505	537	0,0349203
11	chr11:28666219:T:C	rs4923556	28666219	537	0,0349203
11	chr11:28668805:C:G	rs4579914	28668805	537	0,0349203
11	chr11:28669587:A:G	rs10767740	28669587	537	0,0349203
20	chr20:21165245:A:G	rs6035795	21165245	543	0,0346236
11	chr11:28655215:G:C	rs4923553	28655215	538	0,0343472
11	chr11:28526861:T:C	rs990846	28526861	501	0,0343065
11	chr11:28521551:G:A	rs4922805	28521551	501	0,0342969
1	chr1:43596483:C:T	rs673253	43596483	521	0,0339334
20	chr20:21154158:T:C	rs6047273	21154158	546	0,0337934
1	chr1:43586107:G:A	rs3791140	43586107	541	0,0336459
20	chr20:21227106:T:G	rs6035813	21227106	545	0,0335385

20	chr20:21267478:G:A	rs910805	21267478	516	0,0335263
20	chr20:21175938:T:C	rs2328609	21175938	518	0,0334348
20	chr20:21179524:A:C	rs6047299	21179524	518	0,0334348
11	chr11:28658596:T:C	rs2356013	28658596	532	0,033312
1	chr1:43577721:G:A	rs552638	43577721	537	0,0327525
1	chr1:43596488:C:T	rs673242	43596488	542	0,0327099
1	chr1:43550344:G:A	rs11580258	43550344	505	0,0325958
20	chr20:21176870:T:G	rs2093070	21176870	519	0,032572
1	chr1:43599477:C:T	rs56175694	43599477	542	0,0325289
1	chr1:43584276:C:T	rs3791136	43584276	541	0,0325192
1	chr1:43585903:A:G	rs6698389	43585903	541	0,0325192
1	chr1:43586163:A:C	rs501299	43586163	541	0,0325192
1	chr1:43600753:C:A	rs596522	43600753	541	0,0323321
20	chr20:21175529:A:G	rs910975	21175529	519	0,0321499
20	chr20:21176291:G:C	rs2103975	21176291	519	0,0321499
20	chr20:21176925:T:C	rs6035808	21176925	519	0,0321499
20	chr20:21180143:G:A	rs4815030	21180143	519	0,0321499
7	chr7:114647061:T:A	rs2045292	114647061	509	0,0321492
20	chr20:21141038:A:T	rs6047269	21141038	530	0,0320123
5	chr5:88624488:C:A	rs1644042	88624488	502	0,0319146
1	chr1:43631767:G:T	rs12405972	43631767	543	0,0317922
20	chr20:21249817:C:T	rs6082358	21249817	500	0,0317355
5	chr5:88605345:G:A	rs6859545	88605345	503	0,0316789
5	chr5:88618013:G:A	rs324895	88618013	503	0,0316789
5	chr5:88618446:A:G	rs324896	88618446	503	0,0316789
5	chr5:88619663:G:T	rs10462334	88619663	503	0,0316789
1	chr1:43592796:C:T	rs605709	43592796	542	0,0315964
1	chr1:43593571:G:A	rs72673082	43593571	542	0,0315964
1	chr1:43594812:G:A	rs583040	43594812	542	0,0315964
1	chr1:43652735:G:A	rs61768371	43652735	545	0,0314354
3	chr3:71450250:C:T	rs17718444	71450250	533	0,0314329
20	chr20:21178512:T:C	rs2145099	21178512	517	0,0313602
20	chr20:21146082:A:G	rs3762194	21146082	529	0,0313241
5	chr5:88563112:T:G	rs17422060	88563112	498	0,0312566
1	chr1:43658317:C:T	rs61768372	43658317	545	0,0312499
5	chr5:88558577:A:C	rs4916723	88558577	476	0,0312151
20	chr20:21183159:A:G	rs2145100	21183159	519	0,0310897
20	chr20:21138467:C:G	rs6047268	21138467	529	0,030861
20	chr20:21140567:A:T	rs2328607	21140567	529	0,030861
5	chr5:88600784:C:T	rs324886	88600784	497	0,0306551
20	chr20:21177599:C:G	rs6047296	21177599	517	0,030308
20	chr20:21177731:T:G	rs2876596	21177731	517	0,030308
20	chr20:21169993:T:C	rs4815028	21169993	525	0,0302574
20	chr20:21173142:G:C	rs6035803	21173142	525	0,0302574
5	chr5:88588020:G:C	rs56022653	88588020	501	0,0300982
20	chr20:21176320:A:G	rs2103976	21176320	519	0,0300609
20	chr20:21144231:T:C	rs4815022	21144231	529	0,0300515
1	chr1:43655886:C:G	rs2274465	43655886	545	0,0299713
20	chr20:21170737:G:A	rs1072271	21170737	525	0,0299406
5	chr5:88587685:T:C	rs62369151	88587685	502	0,0298903

1	chr1:43656802:T:G	rs11589584	43656802	544	0,0297571
1	chr1:43632835:G:A	rs61769649	43632835	544	0,0295617
20	chr20:21166199:T:C	rs2876595	21166199	525	0,0294534
20	chr20:21155077:G:T	rs6035791	21155077	529	0,0294415
20	chr20:21170255:A:G	rs6047285	21170255	526	0,0293882
20	chr20:21170684:G:A	rs6047288	21170684	526	0,0293882
20	chr20:21170688:A:G	rs6035799	21170688	526	0,0293882
20	chr20:21170708:C:T	rs6035800	21170708	526	0,0293882
20	chr20:21171212:C:T	rs2180581	21171212	526	0,0293882
20	chr20:21171591:G:A	rs6082338	21171591	526	0,0293882
20	chr20:21172102:G:A	rs6082339	21172102	526	0,0293882
20	chr20:21173053:G:A	rs6035802	21173053	526	0,0293882
20	chr20:21173847:G:A	rs6047293	21173847	526	0,0293882
5	chr5:88629003:C:T	rs1644045	88629003	501	0,0290468
20	chr20:21157726:C:A	rs4815024	21157726	530	0,0290111
20	chr20:21154956:G:T	rs6035790	21154956	529	0,0287647
11	chr11:28602326:G:T	rs7931440	28602326	517	0,0287431
6	chr6:70142601:A:G	rs9342783	70142601	523	0,0285781
6	chr6:70142949:A:T	rs2296013	70142949	523	0,0285781
6	chr6:70143876:T:C	rs6935524	70143876	523	0,0285781
6	chr6:70144291:A:G	rs9364074	70144291	527	0,0285256
20	chr20:21165647:A:G	rs6047281	21165647	526	0,0285025
20	chr20:21166369:A:G	rs6047283	21166369	526	0,0285025
20	chr20:21166767:A:G	rs6035796	21166767	526	0,0285025
20	chr20:21168043:G:T	rs4815026	21168043	526	0,0285025
20	chr20:21169627:G:C	rs6132402	21169627	525	0,0284731
20	chr20:21167328:G:C	rs4625995	21167328	526	0,028409
20	chr20:21172498:G:A	rs2328608	21172498	526	0,028409
20	chr20:21228388:C:T	rs11697462	21228388	527	0,0282064
20	chr20:21156917:A:G	rs6047277	21156917	530	0,0281345
5	chr5:88628064:T:G	rs1644044	88628064	499	0,0280659
5	chr5:88630645:G:A	rs11740358	88630645	499	0,0280659
20	chr20:21158348:G:A	rs6035792	21158348	530	0,0280481
20	chr20:21158611:G:A	rs6035793	21158611	530	0,0280481
20	chr20:21158741:C:G	rs6047278	21158741	530	0,0280481
20	chr20:21159530:G:A	rs721785	21159530	530	0,0280481
20	chr20:21159701:C:T	rs2093069	21159701	530	0,0280481
20	chr20:21161712:T:C	rs6047279	21161712	530	0,0280481
6	chr6:70148891:T:C	rs2025284	70148891	524	0,027835
6	chr6:70148037:A:G	rs9454993	70148037	526	0,0276599
6	chr6:70148809:A:C	rs2025286	70148809	526	0,0276599
5	chr5:88633148:G:T	rs1628250	88633148	501	0,0276237
5	chr5:88636991:G:T	rs4916661	88636991	501	0,0276237
20	chr20:21165828:A:T	rs6047282	21165828	526	0,0275363
11	chr11:28605957:T:C	rs10835363	28605957	544	0,0275202
20	chr20:21173632:G:C	rs6035804	21173632	526	0,0274448
20	chr20:21073855:C:T	rs6047225	21073855	517	0,0273423
20	chr20:21161882:C:G	rs4815025	21161882	529	0,0272651
20	chr20:21201749:T:C	rs12625304	21201749	513	0,0263045
7	chr7:114560479:G:A	rs4355713	114560479	528	0,0255362

1	chr1:43674404:A:C	rs10789442	43674404	540	0,0252376
5	chr5:88885292:T:G	rs3850651	88885292	497	0,025061
4	chr4:111296654:C:T	rs17638593	111296654	535	0,0239798
1	chr1:43692458:C:G	rs2270972	43692458	543	0,0238416
7	chr7:114570759:T:C	rs17137124	114570759	528	0,0237311
11	chr11:28609522:G:C	rs12226610	28609522	523	0,0228531
11	chr11:28609701:C:A	rs11030386	28609701	523	0,0228531
1	chr1:43682497:T:C	rs4660257	43682497	543	0,0228068
1	chr1:43686518:G:A	rs1971342	43686518	543	0,0228068
1	chr1:43687044:G:A	rs11805774	43687044	543	0,0228068
1	chr1:43687948:G:C	rs17401357	43687948	543	0,0228068
1	chr1:43688808:G:C	rs3791035	43688808	543	0,0228068
11	chr11:28609937:C:T	rs11030387	28609937	522	0,0226546
11	chr11:28620773:G:A	rs10767733	28620773	522	0,0225568
11	chr11:28569621:A:T	rs11030380	28569621	512	0,0225084
11	chr11:28570040:C:T	rs7127383	28570040	512	0,0225084
11	chr11:28605239:G:A	rs10835362	28605239	524	0,0224852
11	chr11:28606106:G:A	rs10835364	28606106	524	0,0224852
11	chr11:28607002:C:A	rs57806515	28607002	524	0,0224852
11	chr11:28607568:G:A	rs11030385	28607568	524	0,0224852
11	chr11:28613204:C:T	rs10767729	28613204	524	0,0224852
11	chr11:28613315:A:G	rs10767730	28613315	524	0,0224852
11	chr11:28613316:T:C	rs10767731	28613316	524	0,0224852
11	chr11:28614382:G:A	rs7110556	28614382	524	0,0224852
11	chr11:28615789:A:G	rs4923549	28615789	524	0,0224852
11	chr11:28609187:T:G	rs11821132	28609187	522	0,0223448
11	chr11:28608504:A:G	rs10835365	28608504	522	0,0223398
4	chr4:111251830:C:A	rs11729045	111251830	526	0,0222563
11	chr11:28619515:G:A	rs12226518	28619515	522	0,0222312
11	chr11:28619698:G:A	rs12226542	28619698	522	0,0222312
11	chr11:28620721:G:A	rs10767732	28620721	522	0,0222312
11	chr11:28578333:T:A	rs11030381	28578333	512	0,0220821
11	chr11:28617583:T:C	rs12221661	28617583	520	0,0220479
7	chr7:114605694:T:A	rs10230558	114605694	505	0,0219488
1	chr1:43685962:T:G	rs61768374	43685962	542	0,0219196
11	chr11:28609199:A:G	rs59617050	28609199	521	0,0218384
11	chr11:28603810:A:T	rs11030384	28603810	522	0,0217838
1	chr1:43879620:G:A	rs2527776	43879620	498	0,0217034
1	chr1:43721141:A:T	rs11810109	43721141	544	0,0216925
11	chr11:28604117:C:T	rs4442537	28604117	524	0,0216056
11	chr11:28631449:A:G	rs4275621	28631449	530	0,0212366
11	chr11:28615816:T:C	rs4923550	28615816	522	0,0212256
11	chr11:28621014:G:C	rs10767735	28621014	518	0,0211734
11	chr11:28620834:C:T	rs10767734	28620834	522	0,0210011
11	chr11:28631941:T:G	rs6484377	28631941	534	0,0209462
11	chr11:28632083:T:C	rs7124523	28632083	534	0,0209462
11	chr11:28632180:T:G	rs7124653	28632180	534	0,0209462
11	chr11:28632479:C:T	rs4290216	28632479	534	0,0209462
1	chr1:43896951:G:A	rs803682	43896951	502	0,0205091
1	chr1:43885226:G:T	rs803678	43885226	501	0,0204988

11	chr11:28671984:T:G	rs4923557	28671984	523	0,0203197
11	chr11:28672893:C:G	rs10835391	28672893	523	0,0203197
7	chr7:114627941:T:C	rs11983431	114627941	498	0,0201978
11	chr11:28648570:G:T	rs6484386	28648570	527	0,020118
11	chr11:28648575:T:A	rs6484387	28648575	527	0,020118
4	chr4:111287592:T:C	rs79573484	111287592	535	0,0200365
1	chr1:43903772:G:A	rs11210935	43903772	515	0,0199736
4	chr4:111295017:T:A	rs56331412	111295017	536	0,0198016
4	chr4:111296367:C:T	rs17576773	111296367	536	0,0198016
4	chr4:111306830:A:T	rs77216804	111306830	536	0,0198016
11	chr11:28632994:A:G	rs10835384	28632994	533	0,0196603
11	chr11:28639271:A:G	rs7937781	28639271	533	0,0196603
11	chr11:28669740:C:T	rs7119404	28669740	525	0,0194603
20	chr20:21074404:T:C	rs6035772	21074404	527	0,0192865
11	chr11:28666860:G:A	rs7129601	28666860	526	0,0191541
11	chr11:28623283:A:G	rs10835381	28623283	530	0,0187718
11	chr11:28623794:G:C	rs4268495	28623794	530	0,0187718
11	chr11:28628859:C:T	rs4350350	28628859	530	0,0187718
1	chr1:43887565:G:A	rs803675	43887565	508	0,0186322
1	chr1:43716573:A:G	rs17531412	43716573	542	0,0184782
1	chr1:43872903:A:C	rs2906457	43872903	489	0,0183573
1	chr1:43900578:G:A	rs3791101	43900578	513	0,0182355
7	chr7:114350297:C:T	rs2049604	114350297	514	0,018231
1	chr1:43718252:C:T	rs112361411	43718252	541	0,0182006
1	chr1:43903763:G:A	rs11210934	43903763	506	0,0180719
20	chr20:21422129:C:A	rs2424362	21422129	539	0,0179992
11	chr11:28622330:G:A	rs10835371	28622330	530	0,0179879
11	chr11:28622366:T:C	rs10835372	28622366	530	0,0179879
11	chr11:28622395:T:C	rs10835373	28622395	530	0,0179879
11	chr11:28622424:G:A	rs10835374	28622424	530	0,0179879
11	chr11:28622491:T:G	rs10767736	28622491	530	0,0179879
11	chr11:28622565:G:T	rs11030388	28622565	530	0,0179879
11	chr11:28622745:C:A	rs73434729	28622745	530	0,0179879
11	chr11:28623055:A:G	rs12576604	28623055	530	0,0179879
11	chr11:28623079:A:G	rs11030391	28623079	530	0,0179879
11	chr11:28623338:T:C	rs10835382	28623338	530	0,0179879
11	chr11:28624151:G:A	rs4274191	28624151	530	0,0179879
11	chr11:28624272:T:G	rs10767737	28624272	530	0,0179879
11	chr11:28625064:T:A	rs4351802	28625064	530	0,0179879
11	chr11:28625609:A:T	rs4434991	28625609	530	0,0179879
11	chr11:28626408:G:T	rs4290215	28626408	530	0,0179879
11	chr11:28627124:G:A	rs11030392	28627124	530	0,0179879
11	chr11:28628635:A:G	rs4575255	28628635	530	0,0179879
11	chr11:28629811:A:C	rs10835383	28629811	530	0,0179879
11	chr11:28622320:A:G	rs10742197	28622320	530	0,0179751
11	chr11:28624925:C:T	rs4576808	28624925	530	0,0179751
7	chr7:114589084:G:A	rs1229758	114589084	528	0,017972
4	chr4:111267775:T:C	rs79861884	111267775	526	0,0179457
11	chr11:28646124:G:A	rs2883826	28646124	531	0,0178955
11	chr11:28646393:C:T	rs4372430	28646393	531	0,0178955

11	chr11:28646869:T:C	rs6484385	28646869	531	0,0178955
11	chr11:28648417:T:C	rs7942414	28648417	531	0,0178955
11	chr11:28649222:A:G	rs2356016	28649222	531	0,0178955
11	chr11:28649587:T:C	rs2356015	28649587	531	0,0178955
11	chr11:28649653:C:G	rs2356014	28649653	531	0,0178955
11	chr11:28650803:C:G	rs10835388	28650803	531	0,0178955
11	chr11:28654623:A:G	rs11030402	28654623	531	0,0178955
11	chr11:28628323:A:G	rs7121736	28628323	528	0,0178369
5	chr5:88497027:G:A	rs6452792	88497027	488	0,0177818
11	chr11:28621106:G:A	rs4636654	28621106	510	0,017662
11	chr11:28645542:T:C	rs7130649	28645542	528	0,0175654
11	chr11:28582930:C:T	rs11030382	28582930	517	0,0174949
11	chr11:28590845:G:A	rs4520584	28590845	517	0,0174949
11	chr11:28592943:A:T	rs2585813	28592943	517	0,0174949
11	chr11:28596075:C:A	rs4244537	28596075	517	0,0174949
11	chr11:28596496:C:G	rs10835361	28596496	517	0,0174949
11	chr11:28596864:G:A	rs2585811	28596864	517	0,0174949
11	chr11:28586227:G:A	rs4923546	28586227	515	0,0173857
11	chr11:28632740:G:A	rs12282355	28632740	536	0,0173229
11	chr11:28633321:G:A	rs7935241	28633321	536	0,0173229
11	chr11:28633511:A:C	rs7938026	28633511	536	0,0173229
11	chr11:28634125:G:T	rs4244538	28634125	536	0,0173229
11	chr11:28634351:C:G	rs6484378	28634351	536	0,0173229
11	chr11:28634462:T:G	rs7928893	28634462	536	0,0173229
11	chr11:28635703:A:G	rs4344470	28635703	536	0,0173229
11	chr11:28637410:G:A	rs6484379	28637410	536	0,0173229
11	chr11:28638055:A:T	rs3935074	28638055	536	0,0173229
11	chr11:28638658:T:C	rs4337023	28638658	536	0,0173229
11	chr11:28644512:C:G	rs11030397	28644512	536	0,0173229
11	chr11:28644517:G:A	rs11030398	28644517	536	0,0173229
11	chr11:28644793:A:C	rs11030399	28644793	536	0,0173229
11	chr11:28646269:A:G	rs4534569	28646269	529	0,0172696
11	chr11:28655190:A:G	rs4923552	28655190	531	0,0171272
11	chr11:28655717:T:C	rs7942482	28655717	531	0,0171272
7	chr7:114571392:A:G	rs10269986	114571392	545	0,01712
11	chr11:28621752:T:C	rs10835368	28621752	518	0,0170367
1	chr1:43883733:G:A	rs803679	43883733	500	0,0169651
11	chr11:28658309:G:A	rs11030403	28658309	530	0,0169598
1	chr1:43707752:G:A	rs112984125	43707752	543	0,0169532
1	chr1:43722794:A:C	rs12410155	43722794	543	0,0169532
1	chr1:43723048:A:G	rs12410444	43723048	543	0,0169532
11	chr11:28622480:G:A	rs10835375	28622480	528	0,0168793
11	chr11:28580626:C:A	rs2582895	28580626	515	0,0168451
11	chr11:28654592:G:A	rs11030401	28654592	528	0,016801
11	chr11:28624037:T:C	rs4267058	28624037	529	0,0167829
11	chr11:28628989:A:G	rs4568992	28628989	529	0,0167829
11	chr11:28629363:A:G	rs6484376	28629363	529	0,0167829
11	chr11:28582122:A:G	rs7128734	28582122	515	0,0166525
11	chr11:28585152:T:C	rs10835360	28585152	515	0,0166525
11	chr11:28587183:C:G	rs4378371	28587183	515	0,0166525

11	chr11:28593837:A:G	rs2585814	28593837	515	0,0166525
11	chr11:28594152:A:G	rs2582896	28594152	515	0,0166525
11	chr11:28594153:T:C	rs2582897	28594153	515	0,0166525
11	chr11:28594704:T:C	rs10742196	28594704	515	0,0166525
11	chr11:28597209:G:A	rs10767727	28597209	515	0,0166525
11	chr11:28639315:G:T	rs7938122	28639315	536	0,0165663
11	chr11:28585698:T:C	rs7952220	28585698	514	0,0164752
11	chr11:28580674:A:G	rs2585817	28580674	514	0,0163891
11	chr11:28627190:A:G	rs11030393	28627190	541	0,0162829
11	chr11:28628549:C:T	rs4471400	28628549	527	0,0161618
11	chr11:28636483:A:G	rs12577364	28636483	544	0,0160559
20	chr20:21350903:T:G	rs6047396	21350903	523	0,0155631
11	chr11:28630347:G:C	rs7951682	28630347	529	0,0155107
5	chr5:88526854:T:A	rs2194027	88526854	487	0,0150215
4	chr4:111309356:T:A	rs12500836	111309356	530	0,0147402
4	chr4:111333132:A:G	rs12503582	111333132	527	0,014462
16	chr16:61781742:A:G	rs7196175	61781742	536	0,014427
20	chr20:21457201:T:G	rs2424370	21457201	534	0,014115
20	chr20:21463034:C:G	rs8114493	21463034	534	0,014115
20	chr20:21294655:A:C	rs6075789	21294655	521	0,0141012
1	chr1:43556632:A:C	rs2494995	43556632	537	0,0140916
5	chr5:88873835:A:G	rs304137	88873835	470	0,0140742
5	chr5:88874514:T:C	rs216057	88874514	470	0,0140742
20	chr20:21293229:C:T	rs6113185	21293229	520	0,0137265
20	chr20:21294030:G:A	rs6035837	21294030	520	0,0137265
20	chr20:21299182:T:G	rs4813424	21299182	522	0,0136747
20	chr20:21374422:T:A	rs6035862	21374422	527	0,0135974
20	chr20:21375208:A:G	rs6047407	21375208	527	0,0135974
5	chr5:88874249:T:C	rs304136	88874249	469	0,0135295
20	chr20:21465660:G:T	rs722095	21465660	534	0,0134864
5	chr5:88871923:G:C	rs188582	88871923	471	0,0134724
20	chr20:21345460:T:C	rs13043724	21345460	528	0,0133767
20	chr20:21346867:T:G	rs6047389	21346867	528	0,0133767
20	chr20:21349142:T:C	rs1958122	21349142	528	0,0133767
20	chr20:21350734:G:A	rs6047395	21350734	528	0,0133767
20	chr20:21456154:A:G	rs804384	21456154	535	0,0132998
20	chr20:21456675:T:C	rs804385	21456675	535	0,0132998
5	chr5:88872578:C:G	rs1671539	88872578	470	0,0130642
5	chr5:88873138:C:A	rs797419	88873138	470	0,0130642
4	chr4:111314599:T:C	rs28865977	111314599	526	0,012971
20	chr20:21328174:G:A	rs6047380	21328174	522	0,0128538
4	chr4:111311007:T:C	rs76729396	111311007	526	0,0128344
1	chr1:43840448:A:G	rs3011219	43840448	529	0,012831
1	chr1:43845072:C:T	rs12723279	43845072	529	0,012831
1	chr1:43845203:G:A	rs11210931	43845203	529	0,012831
20	chr20:21372771:A:C	rs4815041	21372771	524	0,012806
4	chr4:111310701:T:C	rs28391418	111310701	525	0,0127494
20	chr20:21372841:G:C	rs4815042	21372841	526	0,0126751
4	chr4:111311550:T:C	rs76675076	111311550	527	0,0126278
4	chr4:111328737:T:G	rs7665430	111328737	527	0,0126278

20	chr20:21351811:A:G	rs6035852	21351811	528	0,0126097
20	chr20:21352024:G:C	rs6035853	21352024	528	0,0126097
20	chr20:21353013:G:C	rs6082393	21353013	528	0,0126097
20	chr20:21353030:G:A	rs6075794	21353030	528	0,0126097
20	chr20:21359219:A:G	rs1409816	21359219	528	0,0126097
20	chr20:21361740:T:C	rs4497932	21361740	528	0,0126097
20	chr20:21390733:A:G	rs1555946	21390733	527	0,0122764
20	chr20:21371667:C:T	rs6035860	21371667	527	0,0122555
20	chr20:21373277:C:T	rs6047405	21373277	527	0,0122555
1	chr1:43840750:C:T	rs2906471	43840750	529	0,0121232
20	chr20:21299701:C:T	rs6082377	21299701	522	0,0120749
20	chr20:21313246:C:T	rs1359761	21313246	521	0,0119203
4	chr4:111322536:C:G	rs12505218	111322536	524	0,0118679
1	chr1:43843195:A:G	rs3011220	43843195	528	0,0118674
20	chr20:21379890:A:G	rs6047414	21379890	528	0,0118626
20	chr20:21388294:G:T	rs6035865	21388294	528	0,0118626
20	chr20:21252915:A:G	rs1000176	21252915	521	0,0118046
1	chr1:43837594:A:G	rs3011217	43837594	525	0,0117057
16	chr16:61753223:C:T	rs8061672	61753223	538	0,0116392
16	chr16:61758377:A:G	rs7202312	61758377	539	0,0116247
20	chr20:21310893:C:T	rs6035840	21310893	520	0,0115694
20	chr20:21311200:C:T	rs6082384	21311200	520	0,0115694
20	chr20:21311590:G:C	rs6035841	21311590	520	0,0115694
20	chr20:21312173:C:T	rs6047370	21312173	520	0,0115694
20	chr20:21312391:G:A	rs6047371	21312391	520	0,0115694
1	chr1:43658195:A:G	rs559481	43658195	531	0,0115616
1	chr1:43554723:T:C	rs2842183	43554723	538	0,0115501
1	chr1:43556863:C:T	rs2842176	43556863	538	0,0115501
1	chr1:44009451:C:T	rs113551349	44009451	481	0,0115383
20	chr20:21318093:T:C	rs6047374	21318093	522	0,011527
20	chr20:21318094:G:T	rs6047375	21318094	522	0,011527
20	chr20:21319392:G:A	rs1327153	21319392	522	0,011527
20	chr20:21320839:C:T	rs6047377	21320839	522	0,011527
20	chr20:21321927:C:T	rs1546997	21321927	522	0,011527
20	chr20:21321943:T:C	rs1546998	21321943	522	0,011527
20	chr20:21327058:G:A	rs4813426	21327058	522	0,011527
20	chr20:21329677:C:T	rs4496388	21329677	522	0,011527
20	chr20:21371598:A:G	rs6047404	21371598	546	0,0114345
20	chr20:21260820:G:A	rs6047333	21260820	516	0,0114326
5	chr5:88579561:C:T	rs7722396	88579561	498	0,0112761
5	chr5:88584754:A:G	rs7721028	88584754	498	0,0112761
5	chr5:88586361:A:G	rs1080255	88586361	498	0,0112761
5	chr5:88569970:A:G	rs1644047	88569970	499	0,011254
16	chr16:61749670:C:T	rs7206338	61749670	535	0,0112092
20	chr20:21308964:A:G	rs6035839	21308964	521	0,0111811
20	chr20:21315921:C:A	rs6047372	21315921	521	0,0111811
16	chr16:61755912:G:C	rs9931582	61755912	536	0,0111418
20	chr20:21428075:A:G	rs804373	21428075	539	0,011129
4	chr4:111485805:C:T	rs72678859	111485805	514	0,0110589
1	chr1:43853701:G:A	rs3011225	43853701	546	0,0110421

1	chr1:43555785:C:G	rs10890253	43555785	536	0,0110381
5	chr5:88867954:G:A	rs61104616	88867954	518	0,0109826
1	chr1:43831901:A:C	rs37452	43831901	524	0,0109589
1	chr1:43574039:T:C	rs2819340	43574039	529	0,0108491
20	chr20:21314871:G:T	rs4813425	21314871	520	0,0108374
2	chr2:144956787:A:C	rs1438898	144956787	520	0,0107632
1	chr1:43853335:A:G	rs2906468	43853335	541	0,0106349
1	chr1:43848594:C:T	rs2367804	43848594	531	0,0106164
1	chr1:43579794:A:G	rs2004899	43579794	535	0,0106025
5	chr5:88567792:C:T	rs1659071	88567792	499	0,0105953
5	chr5:88574430:T:C	rs10071454	88574430	499	0,0105953
5	chr5:88577805:C:T	rs918429	88577805	499	0,0105953
1	chr1:43565822:A:G	rs10890255	43565822	534	0,0105437
20	chr20:21436041:T:G	rs811698	21436041	539	0,010514
20	chr20:21439854:C:T	rs28650836	21439854	539	0,010514
20	chr20:21440237:T:C	rs8124380	21440237	539	0,010514
20	chr20:21444512:T:A	rs57897530	21444512	539	0,010514
20	chr20:21444955:G:C	rs804381	21444955	539	0,010514
20	chr20:21449932:G:A	rs804383	21449932	539	0,010514
4	chr4:111500989:G:A	rs72678864	111500989	512	0,0104175
20	chr20:21412561:A:G	rs6132424	21412561	546	0,0103443
1	chr1:43560985:G:C	rs12076635	43560985	535	0,0102652
5	chr5:88601453:A:C	rs324885	88601453	488	0,0101291
1	chr1:43557944:G:A	rs2842175	43557944	538	0,0100156
2	chr2:144962572:A:G	rs72854462	144962572	519	0,00996526
1	chr1:43584333:G:A	rs3791137	43584333	538	0,00996442
1	chr1:43586706:T:G	rs10890261	43586706	538	0,00996442
5	chr5:88574395:G:A	rs10063583	88574395	494	0,00988861
5	chr5:104585069:C:A	rs254011	104585069	498	0,00987871
1	chr1:43560418:C:T	rs3000761	43560418	535	0,00987095
5	chr5:88861735:T:C	rs4518438	88861735	542	0,00982306
20	chr20:21291031:G:C	rs6035835	21291031	512	0,00982038
20	chr20:21291449:A:T	rs6047360	21291449	514	0,00980907
5	chr5:104590343:C:T	rs254013	104590343	501	0,00979499
20	chr20:21289658:C:T	rs2328622	21289658	514	0,00978795
20	chr20:21291893:C:T	rs6047361	21291893	514	0,00978795
1	chr1:43783814:A:G	rs917294	43783814	529	0,00974791
4	chr4:111582716:G:A	rs11729080	111582716	506	0,00962674
20	chr20:21287631:G:A	rs6075788	21287631	515	0,00942629
20	chr20:21290685:C:G	rs4813422	21290685	515	0,00942629
20	chr20:21290915:A:G	rs6082372	21290915	515	0,00942629
20	chr20:21290947:G:A	rs6035834	21290947	515	0,00942629
20	chr20:21291136:G:T	rs6047359	21291136	515	0,00942629
20	chr20:21292673:G:A	rs4815038	21292673	515	0,00942629
1	chr1:43584356:A:C	rs3791138	43584356	538	0,0094262
5	chr5:88865600:T:C	rs12054920	88865600	519	0,00923975
20	chr20:21277415:A:G	rs4813421	21277415	514	0,00915111
20	chr20:21400134:C:A	rs720324	21400134	533	0,00907306
1	chr1:43568097:C:A	rs12089622	43568097	533	0,00889553
1	chr1:43756926:G:A	rs11210913	43756926	531	0,00878056

1	chr1:43606749:A:G	rs539096	43606749	545	0,00876262
1	chr1:43552933:G:T	rs2819339	43552933	540	0,00875044
20	chr20:21283152:A:T	rs6035826	21283152	515	0,00871273
20	chr20:21284820:G:A	rs4815036	21284820	515	0,00871273
20	chr20:21285090:C:T	rs6035830	21285090	515	0,00871273
20	chr20:21285802:C:T	rs6035831	21285802	515	0,00871273
20	chr20:21286713:C:T	rs726024	21286713	515	0,00871273
1	chr1:43560382:T:G	rs10789435	43560382	536	0,00865489
1	chr1:43560509:A:C	rs68191270	43560509	536	0,00865489
5	chr5:88864537:C:T	rs56947091	88864537	526	0,00863564
5	chr5:104557287:A:T	rs1592756	104557287	520	0,00857805
20	chr20:21277855:T:C	rs6047345	21277855	514	0,00854877
20	chr20:21277966:T:C	rs6047346	21277966	514	0,00854877
5	chr5:104557847:T:A	rs1592755	104557847	520	0,00854716
20	chr20:21038664:T:C	rs6082289	21038664	519	0,00848195
20	chr20:21283529:T:C	rs6047353	21283529	514	0,00846458
1	chr1:43592279:T:C	rs573350	43592279	538	0,00837376
1	chr1:43744582:C:G	rs6686238	43744582	533	0,00812695
1	chr1:43745339:A:G	rs6429638	43745339	533	0,00812695
1	chr1:43746026:T:C	rs2158956	43746026	533	0,00812695
1	chr1:43747271:A:G	rs28833034	43747271	533	0,00812695
1	chr1:43747507:C:A	rs7549094	43747507	533	0,00812695
1	chr1:43751205:T:C	rs6669157	43751205	533	0,00812695
1	chr1:43753604:T:C	rs3791042	43753604	533	0,00812695
1	chr1:43763926:G:A	rs4660261	43763926	533	0,00812695
1	chr1:43777612:C:T	rs12119149	43777612	533	0,00812695
20	chr20:21281046:C:T	rs6047348	21281046	515	0,00812522
1	chr1:43558813:A:G	rs11580074	43558813	545	0,0080258
1	chr1:43577434:T:C	rs663618	43577434	536	0,00801732
5	chr5:104554395:C:T	rs6867409	104554395	520	0,00799743
5	chr5:104556759:C:G	rs2193933	104556759	519	0,00794696
1	chr1:43585185:T:C	rs3828150	43585185	539	0,00793898
1	chr1:43616091:T:G	rs516921	43616091	528	0,00787757
11	chr11:28522265:G:A	rs2582915	28522265	474	0,00777711
5	chr5:104550512:T:G	rs6869862	104550512	519	0,00762151
1	chr1:43778195:T:C	rs7543520	43778195	534	0,00755261
1	chr1:43781562:C:G	rs7546040	43781562	534	0,00755261
12	chr12:89378126:T:C	rs704061	89378126	459	0,00755035
12	chr12:89377735:T:C	rs796374	89377735	461	0,00749115
12	chr12:89382507:T:C	rs770083	89382507	460	0,00741825
1	chr1:43605550:G:A	rs631248	43605550	537	0,00714441
1	chr1:43576868:C:G	rs650729	43576868	536	0,00701639
5	chr5:104559401:T:G	rs4438849	104559401	519	0,00684703
5	chr5:88879434:C:G	rs188581	88879434	465	0,00684225
1	chr1:43621825:C:T	rs673485	43621825	537	0,00670049
1	chr1:43504626:G:A	rs2842192	43504626	449	0,00660146
1	chr1:43609834:G:C	rs519669	43609834	540	0,00659342
7	chr7:114469294:A:G	rs727644	114469294	527	0,00657074
1	chr1:43739423:T:C	rs10749850	43739423	537	0,00649739
11	chr11:28554714:G:T	rs2582894	28554714	474	0,00646192

11	chr11:28511111:T:C	rs2585807	28511111	472	0,00645044
1	chr1:43729733:C:T	rs4660743	43729733	540	0,00634798
1	chr1:43510486:G:A	rs2819341	43510486	452	0,00632726
1	chr1:43563682:C:G	rs11210871	43563682	529	0,00630913
1	chr1:43657831:G:A	rs509173	43657831	543	0,00620602
20	chr20:21270205:T:C	rs6082363	21270205	517	0,0062047
1	chr1:43637827:A:G	rs2970606	43637827	544	0,0062046
1	chr1:43638043:G:A	rs564318	43638043	544	0,0062046
1	chr1:43639333:C:T	rs486883	43639333	544	0,0062046
1	chr1:43640277:T:C	rs649504	43640277	544	0,0062046
1	chr1:43640595:C:T	rs574736	43640595	544	0,0062046
1	chr1:43640845:C:A	rs636547	43640845	544	0,0062046
1	chr1:43641315:G:T	rs581005	43641315	544	0,0062046
1	chr1:43642285:C:T	rs694197	43642285	544	0,0062046
1	chr1:43642448:T:A	rs483679	43642448	544	0,0062046
1	chr1:43643528:A:G	rs594293	43643528	544	0,0062046
1	chr1:43644545:A:C	rs609171	43644545	544	0,0062046
1	chr1:43644818:A:G	rs620851	43644818	544	0,0062046
1	chr1:43645516:A:G	rs623965	43645516	544	0,0062046
1	chr1:43646708:T:C	rs72883034	43646708	544	0,0062046
1	chr1:43647770:T:C	rs6429631	43647770	544	0,0062046
1	chr1:43648528:G:T	rs617521	43648528	544	0,0062046
20	chr20:21268900:C:T	rs6047339	21268900	517	0,00614144
7	chr7:114489082:G:A	rs10249234	114489082	527	0,00603264
7	chr7:114498696:G:C	rs2040658	114498696	527	0,00603264
7	chr7:114502195:A:G	rs1015511	114502195	527	0,00603264
1	chr1:43737320:A:G	rs3791041	43737320	539	0,00593475
1	chr1:43728545:T:G	rs10890275	43728545	540	0,00591674
1	chr1:43728863:A:G	rs4660259	43728863	541	0,0058989
1	chr1:43729682:T:C	rs4660260	43729682	541	0,0058989
11	chr11:28504006:A:G	rs2582911	28504006	472	0,005838
5	chr5:88851954:G:A	rs2362108	88851954	526	0,00583182
5	chr5:88852224:C:T	rs2362109	88852224	526	0,00583182
5	chr5:88853474:A:G	rs17487515	88853474	526	0,00583182
5	chr5:88853509:G:A	rs17559709	88853509	526	0,00583182
5	chr5:88854472:G:A	rs4145738	88854472	526	0,00583182
5	chr5:88855175:T:A	rs57055575	88855175	526	0,00583182
5	chr5:88856385:T:C	rs4373304	88856385	526	0,00583182
5	chr5:88856757:C:A	rs11749060	88856757	526	0,00583182
5	chr5:88858057:C:T	rs55740951	88858057	526	0,00583182
7	chr7:114503352:C:T	rs10266297	114503352	526	0,00570173
1	chr1:43596124:C:T	rs674725	43596124	539	0,00563778
5	chr5:88862329:G:C	rs7730255	88862329	527	0,00557871
7	chr7:114497885:A:G	rs1989903	114497885	527	0,00556466
1	chr1:43569422:A:G	rs2842186	43569422	538	0,00555543
1	chr1:43565986:C:T	rs2478978	43565986	537	0,00553652
7	chr7:114484605:C:G	rs6974757	114484605	527	0,00551321
1	chr1:43616545:G:A	rs667676	43616545	541	0,00550226
1	chr1:43563291:G:A	rs2842194	43563291	538	0,00550061
1	chr1:43720773:T:A	rs10890273	43720773	542	0,00546419

1	chr1:43662376:A:G	rs1973512	43662376	542	0,00531871
5	chr5:104596006:G:A	rs2431112	104596006	500	0,00531121
5	chr5:104597772:C:T	rs2447832	104597772	501	0,0053053
1	chr1:43575295:T:G	rs10789436	43575295	535	0,00524694
7	chr7:114479375:G:A	rs2189010	114479375	526	0,00524347
7	chr7:114416000:G:C	rs12533005	114416000	526	0,00516451
7	chr7:114476826:G:A	rs7783012	114476826	527	0,00506083
1	chr1:43629437:A:G	rs10890268	43629437	541	0,00503294
1	chr1:43631843:C:G	rs2970609	43631843	541	0,00503294
1	chr1:43634192:G:T	rs585711	43634192	541	0,00503294
1	chr1:43634239:T:C	rs585652	43634239	541	0,00503294
1	chr1:43717869:G:T	rs6429636	43717869	542	0,00502558
1	chr1:43726088:C:T	rs11210907	43726088	541	0,00497008
1	chr1:43731274:C:T	rs3862228	43731274	541	0,00497008
1	chr1:43636043:A:T	rs664728	43636043	544	0,00490777
1	chr1:43636129:A:G	rs664336	43636129	544	0,00490777
1	chr1:43636586:T:C	rs530201	43636586	544	0,00490777
12	chr12:89368722:C:G	rs1427828	89368722	485	0,00486347
12	chr12:89369752:C:T	rs12819667	89369752	485	0,00486347
5	chr5:104590741:T:A	rs445578	104590741	501	0,00479949
5	chr5:104600642:T:A	rs254035	104600642	501	0,00479949
5	chr5:104568407:A:G	rs12658019	104568407	522	0,0046624
1	chr1:43614741:T:C	rs588657	43614741	542	0,00461227
1	chr1:43616202:A:C	rs517849	43616202	541	0,00461224
1	chr1:43617439:C:T	rs571862	43617439	541	0,00461224
1	chr1:43617836:T:C	rs641365	43617836	541	0,00461224
1	chr1:43617848:G:A	rs641351	43617848	541	0,00461224
1	chr1:43618203:A:G	rs639929	43618203	541	0,00461224
1	chr1:43619068:C:T	rs1143701	43619068	541	0,00461224
1	chr1:43624269:A:G	rs559575	43624269	541	0,00461224
1	chr1:43625004:T:C	rs531459	43625004	541	0,00461224
1	chr1:43625065:C:T	rs628342	43625065	541	0,00461224
1	chr1:43625603:A:G	rs2916568	43625603	541	0,00461224
1	chr1:43616041:T:G	rs516790	43616041	540	0,00460686
1	chr1:43620409:T:C	rs568639	43620409	540	0,00460686
12	chr12:89381252:T:G	rs10506965	89381252	482	0,00444944
5	chr5:104591878:C:T	rs2447838	104591878	500	0,00440607
7	chr7:114419101:C:T	rs2894699	114419101	526	0,00429344
7	chr7:114403104:A:G	rs12536335	114403104	521	0,00424738
1	chr1:43623961:A:G	rs643445	43623961	540	0,00422709
1	chr1:43537286:G:A	rs2819330	43537286	471	0,00416776
1	chr1:43649351:A:G	rs519357	43649351	540	0,00415451
7	chr7:114443495:C:G	rs10228494	114443495	525	0,00409808
5	chr5:104565723:C:T	rs10059133	104565723	525	0,00398097
5	chr5:104568065:G:A	rs12659431	104568065	525	0,00398097
7	chr7:114407487:G:C	rs8180817	114407487	520	0,00391202
12	chr12:89364160:G:A	rs10506971	89364160	490	0,00390212
12	chr12:89366967:A:G	rs1427829	89366967	490	0,00390212
5	chr5:104629522:A:C	rs185260	104629522	494	0,00389646
7	chr7:114406314:C:G	rs2030915	114406314	521	0,00381949

1	chr1:43712399:T:G	rs304303	43712399	540	0,00373519
1	chr1:43663428:T:C	rs656131	43663428	507	0,00356669
5	chr5:104571636:G:A	rs7703746	104571636	525	0,00356357
7	chr7:114505470:G:A	rs6466488	114505470	509	0,0033972
1	chr1:43689712:A:G	rs3791036	43689712	543	0,00335044
1	chr1:43685344:T:G	rs6697354	43685344	546	0,00331586
7	chr7:114430980:C:T	rs1476535	114430980	521	0,00329239
1	chr1:43610348:G:A	rs11210887	43610348	546	0,00327821
5	chr5:104563895:T:C	rs6874138	104563895	525	0,00324
1	chr1:43572014:G:A	rs3001723	43572014	534	0,00322746
5	chr5:104623644:A:G	rs254045	104623644	493	0,00316019
5	chr5:104609425:A:G	rs2431109	104609425	495	0,00315385
7	chr7:114429481:C:G	rs7785701	114429481	521	0,0031284
5	chr5:104610781:C:T	rs2112163	104610781	497	0,00311355
5	chr5:104642625:G:A	rs33817	104642625	497	0,00307974
7	chr7:114436339:C:G	rs10280045	114436339	522	0,00301325
1	chr1:43509093:G:T	rs882464	43509093	467	0,00291564
1	chr1:43510950:G:C	rs10789434	43510950	467	0,00291564
1	chr1:43644078:A:G	rs607296	43644078	541	0,00282302
1	chr1:43641757:C:T	rs2367724	43641757	542	0,00279226
5	chr5:104608237:C:G	rs254025	104608237	496	0,00273794
5	chr5:104578822:T:C	rs13164188	104578822	545	0,00273251
5	chr5:104610505:A:T	rs1363100	104610505	496	0,00267783
1	chr1:43547252:C:T	rs1889588	43547252	477	0,00265874
1	chr1:43610798:G:A	rs549845	43610798	543	0,00252847
7	chr7:114451789:C:A	rs10262103	114451789	525	0,0024594
1	chr1:43634413:G:A	rs11210892	43634413	544	0,00243109
5	chr5:104608319:G:T	rs254024	104608319	495	0,00240547
5	chr5:104559315:C:T	rs7706353	104559315	519	0,00239579
5	chr5:104559414:T:C	rs4320234	104559414	519	0,00239579
5	chr5:104560429:T:C	rs1833514	104560429	519	0,00239579
5	chr5:104562057:T:C	rs4521446	104562057	519	0,00239579
5	chr5:104563477:A:G	rs6596578	104563477	519	0,00239579
5	chr5:104611504:C:T	rs2447828	104611504	495	0,0023164
5	chr5:104612344:C:T	rs2447827	104612344	495	0,0023164
1	chr1:43667345:T:C	rs607062	43667345	539	0,00225343
1	chr1:43621160:C:T	rs1143702	43621160	545	0,00209029
5	chr5:104619660:T:C	rs254023	104619660	493	0,00203443
5	chr5:104614550:C:T	rs254020	104614550	494	0,00199855
5	chr5:104609477:C:T	rs2161097	104609477	496	0,00197013
1	chr1:43671586:C:A	rs586339	43671586	538	0,00194602
5	chr5:104581838:T:A	rs10477835	104581838	538	0,00183424
5	chr5:104582136:T:C	rs1363103	104582136	538	0,00183424
5	chr5:104606092:C:A	rs2018142	104606092	530	0,00160319
5	chr5:104605369:T:G	rs1363101	104605369	530	0,00157039
5	chr5:104605700:T:C	rs10455065	104605700	530	0,00157039
5	chr5:104605714:T:G	rs10455066	104605714	530	0,00157039
1	chr1:43540668:A:G	rs2782639	43540668	477	0,00156548
1	chr1:43617344:G:A	rs653953	43617344	540	0,00150195
1	chr1:43613740:T:C	rs603542	43613740	543	0,00148586

1	chr1:43631859:T:C	rs2970610	43631859	543	0,00148586
1	chr1:43612713:T:C	rs499257	43612713	543	0,00118757
5	chr5:104676602:C:G	rs325506	104676602	492	0,00114427
5	chr5:88433894:A:C	rs6414946	88433894	504	0,00113273
7	chr7:114554560:T:G	rs1859100	114554560	504	0,00110962
5	chr5:104604069:C:T	rs12187903	104604069	529	0,00070574
5	chr5:104566479:C:T	rs6865511	104566479	526	0,00060755
5	chr5:104567257:C:T	rs1421668	104567257	526	0,00060755
5	chr5:104572545:C:T	rs2032790	104572545	526	0,00060755
5	chr5:104572709:A:G	rs1421666	104572709	526	0,00060755
5	chr5:104573167:C:T	rs4295362	104573167	526	0,00060755
5	chr5:104573847:C:T	rs959857	104573847	526	0,00060755
5	chr5:104603973:C:T	rs12187898	104603973	533	0,00054776
7	chr7:114508188:A:G	rs2189015	114508188	510	0,00042665
5	chr5:104568333:A:G	rs12658007	104568333	525	0,00042655
5	chr5:104568336:T:C	rs12658451	104568336	525	0,00042655
7	chr7:114522685:G:A	rs6980093	114522685	510	0,000417
5	chr5:104563989:T:C	rs10064425	104563989	526	0,00034684
5	chr5:104568136:A:G	rs12657531	104568136	526	0,00034684
5	chr5:104569324:T:A	rs11242523	104569324	526	0,00034684
5	chr5:104569574:C:T	rs12515429	104569574	526	0,00034684
5	chr5:104569709:A:C	rs17156671	104569709	526	0,00034684
5	chr5:104570372:C:T	rs2403284	104570372	526	0,00034684
5	chr5:104571905:G:A	rs1421667	104571905	526	0,00034684
3	chr3:20687599:C:T	rs4858253	20687599	545	0,00028873
7	chr7:114508276:C:A	rs2396753	114508276	510	-0,00012541
7	chr7:114520816:G:A	rs6969376	114520816	510	-0,00015762
5	chr5:104569213:T:C	rs11242522	104569213	527	-0,00029127
7	chr7:114540007:A:G	rs10262462	114540007	508	-0,0003941
5	chr5:104576507:C:A	rs10071115	104576507	531	-0,00039718
5	chr5:104554297:G:C	rs1592757	104554297	517	-0,00046472
3	chr3:20678505:C:T	rs1846723	20678505	537	-0,00050642
5	chr5:104576413:A:C	rs62362443	104576413	529	-0,00051876
14	chr14:98224586:T:A	rs76284431	98224586	526	-0,00057674
3	chr3:20683444:C:T	rs55686218	20683444	538	-0,00060824
3	chr3:20682712:G:A	rs2886697	20682712	537	-0,00069877
3	chr3:20680326:A:G	rs56285459	20680326	537	-0,00070644
3	chr3:20680427:C:T	rs55712289	20680427	537	-0,00070644
5	chr5:104582527:C:T	rs10072579	104582527	536	-0,00081076
5	chr5:104581651:A:C	rs10477834	104581651	535	-0,00087249
5	chr5:104582166:G:T	rs1363102	104582166	535	-0,00087249
5	chr5:104582921:C:T	rs10072849	104582921	535	-0,00087249
5	chr5:104582998:G:A	rs10057459	104582998	535	-0,00087249
5	chr5:104583048:G:T	rs10057469	104583048	535	-0,00087249
5	chr5:104584121:A:C	rs10052804	104584121	535	-0,00087249
3	chr3:20683524:C:A	rs56135409	20683524	537	-0,00090678
5	chr5:104568525:A:G	rs12658032	104568525	497	-0,00091826
5	chr5:104584139:G:A	rs10059643	104584139	534	-0,00093327
5	chr5:104577563:A:C	rs11738197	104577563	533	-0,00104771
5	chr5:104577567:C:T	rs13172611	104577567	533	-0,00104771

5	chr5:104577620:T:A	rs13158494	104577620	533	-0,00104771
5	chr5:104578055:A:T	rs1363106	104578055	533	-0,00104771
5	chr5:104578313:C:T	rs1421665	104578313	533	-0,00104771
5	chr5:104578842:G:A	rs13177365	104578842	533	-0,00104771
5	chr5:104579057:A:G	rs10053368	104579057	533	-0,00104771
5	chr5:104579085:A:T	rs10053371	104579085	533	-0,00104771
5	chr5:104579135:T:G	rs10054977	104579135	533	-0,00104771
5	chr5:104579581:A:G	rs12658276	104579581	533	-0,00104771
5	chr5:104579742:G:A	rs10479296	104579742	533	-0,00104771
5	chr5:104580027:A:G	rs10479297	104580027	533	-0,00104771
5	chr5:104580648:T:C	rs7710489	104580648	533	-0,00104771
5	chr5:104580662:G:C	rs7723509	104580662	533	-0,00104771
5	chr5:104581130:A:G	rs62362459	104581130	533	-0,00104771
3	chr3:20685150:G:A	rs4858250	20685150	466	-0,00111004
3	chr3:20685154:C:T	rs4858251	20685154	466	-0,00111004
5	chr5:88417014:A:G	rs6452787	88417014	519	-0,00112907
5	chr5:104577501:A:G	rs11738191	104577501	531	-0,00120368
5	chr5:88445076:T:C	rs7722095	88445076	517	-0,0012267
5	chr5:88468801:G:A	rs4244212	88468801	517	-0,0012267
5	chr5:104602414:C:T	rs1592754	104602414	532	-0,00129631
16	chr16:61746654:A:G	rs4131786	61746654	538	-0,00129758
16	chr16:61754668:C:T	rs7192912	61754668	538	-0,00129758
4	chr4:111546456:G:A	rs3934797	111546456	470	-0,00136235
7	chr7:114407085:A:T	rs7795397	114407085	521	-0,00141585
7	chr7:114435085:T:C	rs28534444	114435085	523	-0,00143872
5	chr5:88467651:C:A	rs10060720	88467651	518	-0,00148852
5	chr5:88473876:C:T	rs6891239	88473876	518	-0,00148852
5	chr5:88477912:G:T	rs6874021	88477912	518	-0,00148852
3	chr3:20680838:A:C	rs1604133	20680838	537	-0,00149502
7	chr7:114434202:C:T	rs10268637	114434202	524	-0,0015128
5	chr5:88416702:C:A	rs7708715	88416702	523	-0,00151781
16	chr16:61754062:A:G	rs7187101	61754062	546	-0,00153822
7	chr7:114482451:C:T	rs2106900	114482451	541	-0,00158954
5	chr5:88455935:G:A	rs7728883	88455935	518	-0,00159165
5	chr5:88458988:C:T	rs6866315	88458988	518	-0,00159165
7	chr7:114435631:A:C	rs10246733	114435631	524	-0,00159672
7	chr7:114474994:A:G	rs2396751	114474994	545	-0,00160075
7	chr7:114440281:T:G	rs2014265	114440281	536	-0,00160111
7	chr7:114442439:T:C	rs10261780	114442439	537	-0,00161283
7	chr7:114464334:T:C	rs10228327	114464334	541	-0,00165895
5	chr5:88467699:G:A	rs11952470	88467699	520	-0,00166239
5	chr5:88469954:T:C	rs9293500	88469954	520	-0,00166239
5	chr5:88475276:A:C	rs6452791	88475276	520	-0,00166239
5	chr5:88477838:A:G	rs6873449	88477838	520	-0,00166239
7	chr7:114475213:T:C	rs2189011	114475213	542	-0,00168923
7	chr7:114503596:T:A	rs9649368	114503596	542	-0,00169792
5	chr5:88401026:G:A	rs4916910	88401026	522	-0,00169884
14	chr14:98244157:G:A	rs78745380	98244157	531	-0,001701
14	chr14:98253981:T:A	rs12590023	98253981	531	-0,001701
5	chr5:88389683:C:T	rs6452785	88389683	520	-0,00171468

7	chr7:114451698:G:A	rs10262192	114451698	545	-0,0017154
14	chr14:98239063:A:G	rs12589260	98239063	525	-0,00171633
7	chr7:114483253:C:T	rs2189008	114483253	542	-0,00171939
7	chr7:114499629:G:A	rs12113612	114499629	542	-0,0017291
5	chr5:88456878:A:G	rs7448716	88456878	520	-0,00173709
5	chr5:88461004:C:T	rs4352629	88461004	520	-0,00173709
7	chr7:114456791:A:T	rs10231382	114456791	540	-0,00174194
5	chr5:104482711:A:G	rs4235642	104482711	544	-0,0017466
14	chr14:98259646:C:T	rs1954554	98259646	532	-0,00174734
5	chr5:88385177:A:G	rs6452784	88385177	520	-0,00177625
14	chr14:98255846:A:C	rs74469887	98255846	531	-0,00178151
5	chr5:88461101:C:T	rs4571506	88461101	544	-0,00179163
5	chr5:88386213:G:A	rs6414945	88386213	521	-0,00182692
5	chr5:88397820:G:C	rs2898558	88397820	520	-0,00183653
7	chr7:114552490:T:C	rs7794413	114552490	508	-0,00186539
7	chr7:114507162:C:A	rs10279936	114507162	515	-0,00188178
7	chr7:114531090:A:G	rs9332390	114531090	513	-0,00188653
3	chr3:20695381:T:C	rs62234988	20695381	477	-0,00204363
3	chr3:20696597:A:G	rs17809565	20696597	478	-0,00208597
3	chr3:20698841:C:G	rs2366311	20698841	478	-0,00208597
3	chr3:20706828:T:G	rs62234992	20706828	470	-0,00217384
3	chr3:20703004:G:A	rs62234991	20703004	469	-0,00221504
3	chr3:20700799:C:T	rs55964255	20700799	466	-0,00226598
3	chr3:20627579:T:G	rs4858241	20627579	531	-6,25E+00

Supplementary Table 3.

CHR	SNP	BP	A1	MAF	A2	CHISQ	P	OR	SE	L95	U95	CHISQ_BD	P_BD
2	chr2:144962572:A:G	144962572	G	0,2861	A	1,2330	0,2669	1,2090	0,1681	0,8695	1,6810	5,5230	0,01877
2	chr2:144956787:A:C	144956787	C	0,2865	A	1,2480	0,2639	1,2090	0,1674	0,8709	1,6790	5,1730	0,02294
20	chr20:21038664:T:C	21038664	T	0,2360	C	4,1850	0,0408	1,4530	0,1800	1,0210	2,0680	3,2540	0,07125
20	chr20:21074404:T:C	21074404	T	0,4497	C	2,1260	0,1448	1,2440	0,1498	0,9276	1,6690	2,9490	0,08595
20	chr20:21175529:A:G	21175529	A	0,4364	G	4,0310	0,0447	1,3600	0,1532	1,0070	1,8360	2,2850	0,1306
20	chr20:21176291:G:C	21176291	G	0,4364	C	4,0310	0,0447	1,3600	0,1532	1,0070	1,8360	2,2850	0,1306
20	chr20:21176925:T:C	21176925	T	0,4364	C	4,0310	0,0447	1,3600	0,1532	1,0070	1,8360	2,2850	0,1306
20	chr20:21180143:G:A	21180143	G	0,4364	A	4,0310	0,0447	1,3600	0,1532	1,0070	1,8360	2,2850	0,1306
20	chr20:21176320:A:G	21176320	A	0,4383	G	3,8970	0,0484	1,3520	0,1529	1,0020	1,8250	2,1940	0,1385
20	chr20:21178512:T:C	21178512	T	0,4371	C	3,8060	0,0511	1,3500	0,1540	0,9986	1,8260	2,1130	0,1461
11	chr11:28504006:A:G	28504006	A	0,2383	G	0,0532	0,8175	1,0410	0,1750	0,7384	1,4660	2,0920	0,1481
20	chr20:21183159:A:G	21183159	A	0,4374	G	3,6670	0,0555	1,3400	0,1530	0,9931	1,8090	2,0650	0,1507
1	chr1:43885226:G:T	43885226	G	0,4461	T	0,0222	0,8815	0,9776	0,1521	0,7256	1,3170	2,0380	0,1534
1	chr1:43896951:G:A	43896951	G	0,4462	A	0,0222	0,8815	0,9776	0,1521	0,7256	1,3170	2,0370	0,1535
5	chr5:88629003:C:T	88629003	T	0,4421	C	0,8444	0,3581	1,1500	0,1522	0,8532	1,5500	2,0270	0,1545
5	chr5:88497027:G:A	88497027	A	0,4580	G	0,3720	0,5419	1,1000	0,1559	0,8105	1,4930	1,9930	0,158
6	chr6:70148037:A:G	70148037	G	0,4468	A	0,2805	0,5964	1,0820	0,1485	0,8089	1,4480	1,9810	0,1593
6	chr6:70148809:A:C	70148809	C	0,4468	A	0,2805	0,5964	1,0820	0,1485	0,8089	1,4480	1,9810	0,1593
20	chr20:21141038:A:T	21141038	A	0,4717	T	5,8320	0,0157	1,4360	0,1503	1,0700	1,9280	1,9600	0,1615
1	chr1:43879620:G:A	43879620	G	0,4468	A	0,3124	0,5762	0,9179	0,1535	0,6793	1,2400	1,9080	0,1671
5	chr5:88558577:A:C	88558577	C	0,4611	A	0,8470	0,3574	1,1550	0,1571	0,8492	1,5720	1,9090	0,1671
20	chr20:21177599:C:G	21177599	C	0,4381	G	3,4500	0,0632	1,3310	0,1538	0,9844	1,7990	1,9020	0,1679
20	chr20:21177731:T:G	21177731	T	0,4381	G	3,4500	0,0632	1,3310	0,1538	0,9844	1,7990	1,9020	0,1679
5	chr5:104550512:T:G	104550512	T	0,3776	G	0,4085	0,5227	1,1000	0,1496	0,8202	1,4750	1,8960	0,1685
11	chr11:28648922:G:A	28648922	A	0,1970	G	3,1190	0,0774	0,7358	0,1770	0,5201	1,0410	1,8860	0,1696
11	chr11:28654958:T:C	28654958	C	0,1970	T	3,1190	0,0774	0,7358	0,1770	0,5201	1,0410	1,8860	0,1696
11	chr11:28655314:A:G	28655314	G	0,1970	A	3,1190	0,0774	0,7358	0,1770	0,5201	1,0410	1,8860	0,1696
11	chr11:28657323:A:G	28657323	G	0,1970	A	3,1190	0,0774	0,7358	0,1770	0,5201	1,0410	1,8860	0,1696
20	chr20:21227106:T:G	21227106	T	0,4266	G	2,7070	0,0999	1,2790	0,1494	0,9543	1,7140	1,8670	0,1719
11	chr11:28646737:A:G	28646737	G	0,1974	A	3,1420	0,0763	0,7349	0,1770	0,5195	1,0400	1,8520	0,1735
11	chr11:28650082:G:C	28650082	C	0,1974	G	3,1420	0,0763	0,7349	0,1770	0,5195	1,0400	1,8520	0,1735
6	chr6:70148891:T:C	70148891	C	0,4447	T	0,2520	0,6157	1,0780	0,1487	0,8053	1,4420	1,7800	0,1822
11	chr11:28655215:G:C	28655215	C	0,1980	G	3,1990	0,0737	0,7329	0,1769	0,5181	1,0370	1,7710	0,1832
11	chr11:28659176:C:A	28659176	A	0,1946	C	2,5240	0,1121	0,7576	0,1778	0,5347	1,0740	1,7710	0,1833
5	chr5:104554395:C:T	104554395	C	0,3788	T	0,4790	0,4889	1,1080	0,1496	0,8268	1,4860	1,7510	0,1858
20	chr20:21175938:T:C	21175938	T	0,4228	C	2,9660	0,0850	1,3040	0,1540	0,9643	1,7630	1,7380	0,1874
20	chr20:21179524:A:C	21179524	A	0,4228	C	2,9660	0,0850	1,3040	0,1540	0,9643	1,7630	1,7380	0,1874
6	chr6:70142601:A:G	70142601	G	0,4436	A	0,2732	0,6012	1,0810	0,1487	0,8078	1,4470	1,7300	0,1885
6	chr6:70142949:A:T	70142949	T	0,4436	A	0,2732	0,6012	1,0810	0,1487	0,8078	1,4470	1,7300	0,1885

6	chr6:70143876:T:C	70143876	C	0,4436	T	0,2732	0,6012	1,0810	0,1487	0,8078	1,4470	1,7300	0,1885
11	chr11:28634517:A:T	28634517	T	0,1974	A	2,8480	0,0915	0,7455	0,1771	0,5269	1,0550	1,7200	0,1897
11	chr11:28634687:C:A	28634687	A	0,1974	C	2,8480	0,0915	0,7455	0,1771	0,5269	1,0550	1,7200	0,1897
11	chr11:28644991:C:T	28644991	T	0,1974	C	2,8480	0,0915	0,7455	0,1771	0,5269	1,0550	1,7200	0,1897
6	chr6:70144291:A:G	70144291	G	0,4440	A	0,2678	0,6048	1,0800	0,1483	0,8077	1,4440	1,7110	0,1909
20	chr20:21228388:C:T	21228388	C	0,4412	T	2,8300	0,0925	1,2900	0,1511	0,9590	1,7340	1,6860	0,1942
20	chr20:21176870:T:G	21176870	T	0,4306	G	3,5810	0,0584	1,3370	0,1535	0,9897	1,8060	1,6840	0,1944
5	chr5:104559401:T:G	104559401	T	0,3825	G	0,3059	0,5802	1,0860	0,1497	0,8097	1,4560	1,6520	0,1987
20	chr20:21138467:C:G	21138467	C	0,4802	G	4,1820	0,0409	1,3580	0,1499	1,0120	1,8210	1,6510	0,1989
20	chr20:21140567:A:T	21140567	A	0,4802	T	4,1820	0,0409	1,3580	0,1499	1,0120	1,8210	1,6510	0,1989
11	chr11:28615816:T:C	28615816	C	0,3343	T	1,5250	0,2168	0,8274	0,1547	0,6110	1,1210	1,6450	0,1996
1	chr1:43903763:G:A	43903763	G	0,4417	A	0,0205	0,8861	0,9785	0,1518	0,7266	1,3180	1,6350	0,201
5	chr5:88526854:T:A	88526854	A	0,4928	T	0,2281	0,6329	1,0750	0,1517	0,7986	1,4480	1,6280	0,202
5	chr5:88624488:C:A	88624488	A	0,4472	C	0,7868	0,3751	1,1440	0,1522	0,8492	1,5420	1,5970	0,2063
11	chr11:28626638:C:T	28626638	T	0,1919	C	1,7110	0,1908	0,7939	0,1794	0,5585	1,1280	1,5970	0,2063
11	chr11:28626783:A:T	28626783	T	0,1919	A	1,7110	0,1908	0,7939	0,1794	0,5585	1,1280	1,5970	0,2063
11	chr11:28626831:G:C	28626831	C	0,1919	G	1,7110	0,1908	0,7939	0,1794	0,5585	1,1280	1,5970	0,2063
11	chr11:28662674:G:A	28662674	A	0,1983	G	2,7840	0,0952	0,7479	0,1770	0,5286	1,0580	1,5890	0,2075
11	chr11:28664505:C:T	28664505	T	0,1983	C	2,7840	0,0952	0,7479	0,1770	0,5286	1,0580	1,5890	0,2075
11	chr11:28666219:T:C	28666219	C	0,1983	T	2,7840	0,0952	0,7479	0,1770	0,5286	1,0580	1,5890	0,2075
11	chr11:28668805:C:G	28668805	G	0,1983	C	2,7840	0,0952	0,7479	0,1770	0,5286	1,0580	1,5890	0,2075
11	chr11:28669587:A:G	28669587	G	0,1983	A	2,7840	0,0952	0,7479	0,1770	0,5286	1,0580	1,5890	0,2075
11	chr11:28604117:C:T	28604117	T	0,3349	C	1,4520	0,2282	0,8316	0,1544	0,6145	1,1250	1,5850	0,2081
5	chr5:88605345:G:A	88605345	A	0,4473	G	0,8095	0,3683	1,1460	0,1521	0,8509	1,5450	1,5730	0,2098
5	chr5:88618013:G:A	88618013	A	0,4473	G	0,8095	0,3683	1,1460	0,1521	0,8509	1,5450	1,5730	0,2098
5	chr5:88618446:A:G	88618446	G	0,4473	A	0,8095	0,3683	1,1460	0,1521	0,8509	1,5450	1,5730	0,2098
5	chr5:88619663:G:T	88619663	T	0,4473	G	0,8095	0,3683	1,1460	0,1521	0,8509	1,5450	1,5730	0,2098
11	chr11:28511111:T:C	28511111	T	0,2373	C	0,0065	0,9360	1,0140	0,1758	0,7185	1,4310	1,5620	0,2114
11	chr11:28608504:A:G	28608504	G	0,3381	A	1,5880	0,2076	0,8247	0,1542	0,6096	1,1160	1,5420	0,2143
1	chr1:43887565:G:A	43887565	G	0,3789	A	0,0706	0,7905	0,9599	0,1548	0,7087	1,3000	1,5140	0,2185
11	chr11:28609522:G:C	28609522	C	0,3356	G	1,2870	0,2566	0,8406	0,1543	0,6212	1,1370	1,5110	0,219
11	chr11:28609701:C:A	28609701	A	0,3356	C	1,2870	0,2566	0,8406	0,1543	0,6212	1,1370	1,5110	0,219
11	chr11:28609937:C:T	28609937	T	0,3352	C	1,2470	0,2642	0,8426	0,1546	0,6223	1,1410	1,4870	0,2226
5	chr5:104559315:C:T	104559315	C	0,3603	T	0,4079	0,5230	1,1010	0,1516	0,8181	1,4820	1,4830	0,2232
5	chr5:104559414:T:C	104559414	T	0,3603	C	0,4079	0,5230	1,1010	0,1516	0,8181	1,4820	1,4830	0,2232
5	chr5:104560429:T:C	104560429	T	0,3603	C	0,4079	0,5230	1,1010	0,1516	0,8181	1,4820	1,4830	0,2232
5	chr5:104562057:T:C	104562057	T	0,3603	C	0,4079	0,5230	1,1010	0,1516	0,8181	1,4820	1,4830	0,2232
5	chr5:104563477:A:G	104563477	A	0,3603	G	0,4079	0,5230	1,1010	0,1516	0,8181	1,4820	1,4830	0,2232
11	chr11:28605239:G:A	28605239	A	0,3359	G	1,3070	0,2529	0,8395	0,1543	0,6205	1,1360	1,4770	0,2242
11	chr11:28606106:G:A	28606106	A	0,3359	G	1,3070	0,2529	0,8395	0,1543	0,6205	1,1360	1,4770	0,2242
11	chr11:28607002:C:A	28607002	A	0,3359	C	1,3070	0,2529	0,8395	0,1543	0,6205	1,1360	1,4770	0,2242
11	chr11:28607568:G:A	28607568	A	0,3359	G	1,3070	0,2529	0,8395	0,1543	0,6205	1,1360	1,4770	0,2242

11	chr11:28613204:C:T	28613204	T	0,3359	C	1,3070	0,2529	0,8395	0,1543	0,6205	1,1360	1,4770	0,2242
11	chr11:28613315:A:G	28613315	G	0,3359	A	1,3070	0,2529	0,8395	0,1543	0,6205	1,1360	1,4770	0,2242
11	chr11:28613316:T:C	28613316	C	0,3359	T	1,3070	0,2529	0,8395	0,1543	0,6205	1,1360	1,4770	0,2242
11	chr11:28614382:G:A	28614382	A	0,3359	G	1,3070	0,2529	0,8395	0,1543	0,6205	1,1360	1,4770	0,2242
11	chr11:28615789:A:G	28615789	G	0,3359	A	1,3070	0,2529	0,8395	0,1543	0,6205	1,1360	1,4770	0,2242
11	chr11:28622320:A:G	28622320	G	0,3434	A	3,3880	0,0657	0,7557	0,1535	0,5594	1,0210	1,4720	0,225
11	chr11:28624925:C:T	28624925	T	0,3434	C	3,3880	0,0657	0,7557	0,1535	0,5594	1,0210	1,4720	0,225
11	chr11:28630347:G:C	28630347	C	0,3384	G	3,1160	0,0775	0,7638	0,1540	0,5648	1,0330	1,4700	0,2254
11	chr11:28609187:T:G	28609187	G	0,3362	T	1,3150	0,2515	0,8390	0,1543	0,6201	1,1350	1,4660	0,226
5	chr5:104556759:C:G	104556759	C	0,3844	G	0,0484	0,8258	1,0330	0,1498	0,7705	1,3860	1,4650	0,2261
20	chr20:21170737:G:A	21170737	G	0,4724	A	3,2380	0,0719	1,3100	0,1502	0,9759	1,7580	1,4600	0,227
20	chr20:21158348:G:A	21158348	G	0,4774	A	4,4680	0,0345	1,3720	0,1498	1,0230	1,8400	1,4580	0,2272
20	chr20:21158611:G:A	21158611	G	0,4774	A	4,4680	0,0345	1,3720	0,1498	1,0230	1,8400	1,4580	0,2272
20	chr20:21158741:C:G	21158741	C	0,4774	G	4,4680	0,0345	1,3720	0,1498	1,0230	1,8400	1,4580	0,2272
20	chr20:21159530:G:A	21159530	G	0,4774	A	4,4680	0,0345	1,3720	0,1498	1,0230	1,8400	1,4580	0,2272
20	chr20:21159701:C:T	21159701	C	0,4774	T	4,4680	0,0345	1,3720	0,1498	1,0230	1,8400	1,4580	0,2272
5	chr5:88633148:G:T	88633148	T	0,4431	G	0,6675	0,4139	1,1320	0,1523	0,8401	1,5260	1,4570	0,2273
5	chr5:88636991:G:T	88636991	T	0,4431	G	0,6675	0,4139	1,1320	0,1523	0,8401	1,5260	1,4570	0,2273
11	chr11:28622480:G:A	28622480	A	0,3438	G	3,5720	0,0588	0,7496	0,1538	0,5546	1,0130	1,4540	0,2278
5	chr5:88630645:G:A	88630645	A	0,4429	G	0,6862	0,4075	1,1350	0,1528	0,8411	1,5310	1,4370	0,2306
12	chr12:89377735:T:C	89377735	C	0,4143	T	2,2310	0,1353	1,2660	0,1585	0,9277	1,7270	1,4320	0,2314
12	chr12:89378126:T:C	89378126	C	0,4139	T	2,2880	0,1304	1,2700	0,1589	0,9302	1,7340	1,4260	0,2325
11	chr11:28609199:A:G	28609199	G	0,3369	A	1,3430	0,2466	0,8375	0,1543	0,6189	1,1330	1,4220	0,2332
20	chr20:21144231:T:C	21144231	T	0,4773	C	4,1170	0,0425	1,3550	0,1499	1,0100	1,8170	1,4120	0,2346
12	chr12:89382507:T:C	89382507	C	0,4141	T	2,2710	0,1318	1,2690	0,1589	0,9295	1,7330	1,4060	0,2357
5	chr5:104557287:A:T	104557287	A	0,3798	T	0,2030	0,6523	1,0690	0,1497	0,7975	1,4340	1,3950	0,2375
20	chr20:21166199:T:C	21166199	T	0,4800	C	3,8200	0,0507	1,3400	0,1500	0,9987	1,7980	1,3960	0,2375
20	chr20:21170255:A:G	21170255	A	0,4800	G	3,8250	0,0505	1,3400	0,1500	0,9989	1,7980	1,3910	0,2382
20	chr20:21170684:G:A	21170684	G	0,4800	A	3,8250	0,0505	1,3400	0,1500	0,9989	1,7980	1,3910	0,2382
20	chr20:21170688:A:G	21170688	A	0,4800	G	3,8250	0,0505	1,3400	0,1500	0,9989	1,7980	1,3910	0,2382
20	chr20:21170708:C:T	21170708	C	0,4800	T	3,8250	0,0505	1,3400	0,1500	0,9989	1,7980	1,3910	0,2382
20	chr20:21171212:C:T	21171212	C	0,4800	T	3,8250	0,0505	1,3400	0,1500	0,9989	1,7980	1,3910	0,2382
20	chr20:21171591:G:A	21171591	G	0,4800	A	3,8250	0,0505	1,3400	0,1500	0,9989	1,7980	1,3910	0,2382
20	chr20:21172102:G:A	21172102	G	0,4800	A	3,8250	0,0505	1,3400	0,1500	0,9989	1,7980	1,3910	0,2382
20	chr20:21173053:G:A	21173053	G	0,4800	A	3,8250	0,0505	1,3400	0,1500	0,9989	1,7980	1,3910	0,2382
20	chr20:21173847:G:A	21173847	G	0,4800	A	3,8250	0,0505	1,3400	0,1500	0,9989	1,7980	1,3910	0,2382
11	chr11:28623283:A:G	28623283	G	0,3443	A	3,1740	0,0748	0,7626	0,1534	0,5645	1,0300	1,3720	0,2414
11	chr11:28623794:G:C	28623794	C	0,3443	G	3,1740	0,0748	0,7626	0,1534	0,5645	1,0300	1,3720	0,2414
11	chr11:28628859:C:T	28628859	T	0,3443	C	3,1740	0,0748	0,7626	0,1534	0,5645	1,0300	1,3720	0,2414
5	chr5:104571636:G:A	104571636	A	0,3733	G	0,2321	0,6300	1,0750	0,1510	0,7997	1,4450	1,3390	0,2472
20	chr20:21165647:A:G	21165647	A	0,4791	G	3,9050	0,0481	1,3440	0,1500	1,0020	1,8040	1,3280	0,2492
20	chr20:21166369:A:G	21166369	A	0,4791	G	3,9050	0,0481	1,3440	0,1500	1,0020	1,8040	1,3280	0,2492

20	chr20:21166767:A:G	21166767	A	0,4791	G	3,9050	0,0481	1,3440	0,1500	1,0020	1,8040	1,3280	0,2492
20	chr20:21168043:G:T	21168043	G	0,4791	T	3,9050	0,0481	1,3440	0,1500	1,0020	1,8040	1,3280	0,2492
11	chr11:28522265:G:A	28522265	G	0,2395	A	0,0454	0,8312	1,0380	0,1750	0,7363	1,4620	1,3140	0,2516
5	chr5:104557847:T:A	104557847	T	0,3779	A	0,3169	0,5734	1,0880	0,1498	0,8108	1,4590	1,3140	0,2517
4	chr4:111582716:G:A	111582716	A	0,0385	G	2,4420	0,1181	0,5330	0,3905	0,2479	1,1460	1,3090	0,2527
20	chr20:21157726:C:A	21157726	C	0,4764	A	4,1160	0,0425	1,3540	0,1496	1,0100	1,8150	1,3020	0,2539
11	chr11:28622330:G:A	28622330	A	0,3453	G	3,2480	0,0715	0,7601	0,1534	0,5628	1,0270	1,3010	0,2541
11	chr11:28622366:T:C	28622366	C	0,3453	T	3,2480	0,0715	0,7601	0,1534	0,5628	1,0270	1,3010	0,2541
11	chr11:28622395:T:C	28622395	C	0,3453	T	3,2480	0,0715	0,7601	0,1534	0,5628	1,0270	1,3010	0,2541
11	chr11:28622424:G:A	28622424	A	0,3453	G	3,2480	0,0715	0,7601	0,1534	0,5628	1,0270	1,3010	0,2541
11	chr11:28622491:T:G	28622491	G	0,3453	T	3,2480	0,0715	0,7601	0,1534	0,5628	1,0270	1,3010	0,2541
11	chr11:28622565:G:T	28622565	T	0,3453	G	3,2480	0,0715	0,7601	0,1534	0,5628	1,0270	1,3010	0,2541
11	chr11:28622745:C:A	28622745	A	0,3453	C	3,2480	0,0715	0,7601	0,1534	0,5628	1,0270	1,3010	0,2541
11	chr11:28623055:A:G	28623055	G	0,3453	A	3,2480	0,0715	0,7601	0,1534	0,5628	1,0270	1,3010	0,2541
11	chr11:28623079:A:G	28623079	G	0,3453	A	3,2480	0,0715	0,7601	0,1534	0,5628	1,0270	1,3010	0,2541
11	chr11:28623338:T:C	28623338	C	0,3453	T	3,2480	0,0715	0,7601	0,1534	0,5628	1,0270	1,3010	0,2541
11	chr11:28624151:G:A	28624151	A	0,3453	G	3,2480	0,0715	0,7601	0,1534	0,5628	1,0270	1,3010	0,2541
11	chr11:28624272:T:G	28624272	G	0,3453	T	3,2480	0,0715	0,7601	0,1534	0,5628	1,0270	1,3010	0,2541
11	chr11:28625064:T:A	28625064	A	0,3453	T	3,2480	0,0715	0,7601	0,1534	0,5628	1,0270	1,3010	0,2541
11	chr11:28625609:A:T	28625609	T	0,3453	A	3,2480	0,0715	0,7601	0,1534	0,5628	1,0270	1,3010	0,2541
11	chr11:28626408:G:T	28626408	T	0,3453	G	3,2480	0,0715	0,7601	0,1534	0,5628	1,0270	1,3010	0,2541
11	chr11:28627124:G:A	28627124	A	0,3453	G	3,2480	0,0715	0,7601	0,1534	0,5628	1,0270	1,3010	0,2541
11	chr11:28628635:A:G	28628635	G	0,3453	A	3,2480	0,0715	0,7601	0,1534	0,5628	1,0270	1,3010	0,2541
11	chr11:28629811:A:C	28629811	C	0,3453	A	3,2480	0,0715	0,7601	0,1534	0,5628	1,0270	1,3010	0,2541
20	chr20:21146082:A:G	21146082	A	0,4707	G	4,5340	0,0332	1,3760	0,1501	1,0250	1,8470	1,2990	0,2544
20	chr20:21249817:C:T	21249817	C	0,4350	T	2,3610	0,1244	1,2690	0,1550	0,9366	1,7200	1,2960	0,255
5	chr5:104578822:T:C	104578822	C	0,2505	T	0,0080	0,9289	0,9857	0,1632	0,7158	1,3570	1,2830	0,2574
20	chr20:21161882:C:G	21161882	C	0,4783	G	4,0560	0,0440	1,3510	0,1497	1,0080	1,8120	1,2810	0,2576
11	chr11:28628549:C:T	28628549	T	0,3491	C	3,9070	0,0481	0,7401	0,1534	0,5479	0,9998	1,2460	0,2643
20	chr20:21156917:A:G	21156917	A	0,4755	G	4,1990	0,0405	1,3580	0,1496	1,0130	1,8210	1,2410	0,2653
20	chr20:21169627:G:C	21169627	G	0,4810	C	3,4710	0,0625	1,3210	0,1498	0,9852	1,7720	1,2330	0,2668
20	chr20:21167328:G:C	21167328	G	0,4810	C	3,4760	0,0623	1,3220	0,1498	0,9854	1,7730	1,2290	0,2676
20	chr20:21172498:G:A	21172498	G	0,4810	A	3,4760	0,0623	1,3220	0,1498	0,9854	1,7730	1,2290	0,2676
11	chr11:28655291:A:G	28655291	G	0,2028	A	2,4640	0,1165	0,7631	0,1747	0,5419	1,0750	1,2260	0,2681
5	chr5:104672432:G:A	104672432	G	0,2442	A	2,0310	0,1542	0,7791	0,1733	0,5547	1,0940	1,2150	0,2703
5	chr5:88885292:T:G	88885292	G	0,5000	T	1,1620	0,2811	1,1800	0,1534	0,8733	1,5930	1,2090	0,2715
20	chr20:21161712:T:C	21161712	T	0,4774	C	3,8640	0,0493	1,3410	0,1496	1,0000	1,7990	1,1980	0,2737
11	chr11:28628323:A:G	28628323	G	0,3494	A	3,5470	0,0597	0,7510	0,1532	0,5562	1,0140	1,1970	0,274
20	chr20:21154158:T:C	21154158	T	0,4597	C	3,5080	0,0611	1,3180	0,1477	0,9870	1,7610	1,1940	0,2744
5	chr5:104676602:C:G	104676602	C	0,3974	G	3,2510	0,0714	0,7538	0,1563	0,5549	1,0240	1,1790	0,2775
5	chr5:88600784:C:T	88600784	T	0,4547	C	0,9674	0,3253	1,1630	0,1536	0,8607	1,5710	1,1770	0,278
5	chr5:104581838:T:A	104581838	A	0,2454	T	0,0106	0,9181	1,0170	0,1657	0,7350	1,4070	1,1700	0,2794

5	chr5:104582136:T:C	104582136	C	0,2454	T	0,0106	0,9181	1,0170	0,1657	0,7350	1,4070	1,1700	0,2794
20	chr20:21165828:A:T	21165828	A	0,4800	T	3,5530	0,0595	1,3260	0,1498	0,9884	1,7780	1,1690	0,2796
20	chr20:21187755:C:T	21187755	T	0,4165	C	2,0000	0,1573	1,2410	0,1526	0,9204	1,6740	1,1380	0,286
5	chr5:88563112:T:G	88563112	G	0,4618	T	1,4330	0,2314	1,2010	0,1533	0,8895	1,6220	1,1370	0,2863
5	chr5:104568333:A:G	104568333	G	0,3495	A	0,4980	0,4804	1,1130	0,1526	0,8253	1,5010	1,1280	0,2881
5	chr5:104568336:T:C	104568336	C	0,3495	T	0,4980	0,4804	1,1130	0,1526	0,8253	1,5010	1,1280	0,2881
11	chr11:28605957:T:C	28605957	C	0,3511	T	1,1500	0,2835	0,8521	0,1502	0,6348	1,1440	1,1280	0,2883
1	chr1:43872903:A:C	43872903	A	0,4305	C	0,0028	0,9575	1,0080	0,1563	0,7423	1,3700	1,1200	0,2898
5	chr5:104671732:G:C	104671732	G	0,2390	C	1,4950	0,2214	0,8070	0,1734	0,5745	1,1340	1,1160	0,2908
5	chr5:104563989:T:C	104563989	C	0,3498	T	0,4876	0,4850	1,1120	0,1526	0,8244	1,4990	1,1060	0,2929
5	chr5:104568136:A:G	104568136	G	0,3498	A	0,4876	0,4850	1,1120	0,1526	0,8244	1,4990	1,1060	0,2929
5	chr5:104569324:T:A	104569324	A	0,3498	T	0,4876	0,4850	1,1120	0,1526	0,8244	1,4990	1,1060	0,2929
5	chr5:104569574:C:T	104569574	T	0,3498	C	0,4876	0,4850	1,1120	0,1526	0,8244	1,4990	1,1060	0,2929
5	chr5:104569709:A:C	104569709	C	0,3498	A	0,4876	0,4850	1,1120	0,1526	0,8244	1,4990	1,1060	0,2929
5	chr5:104570372:C:T	104570372	T	0,3498	C	0,4876	0,4850	1,1120	0,1526	0,8244	1,4990	1,1060	0,2929
5	chr5:104571905:G:A	104571905	A	0,3498	G	0,4876	0,4850	1,1120	0,1526	0,8244	1,4990	1,1060	0,2929
5	chr5:104568407:A:G	104568407	G	0,3774	A	0,3147	0,5748	1,0880	0,1515	0,8087	1,4650	1,1060	0,293
11	chr11:28624037:T:C	28624037	C	0,3507	T	3,6550	0,0559	0,7477	0,1531	0,5539	1,0100	1,1050	0,2931
11	chr11:28628989:A:G	28628989	G	0,3507	A	3,6550	0,0559	0,7477	0,1531	0,5539	1,0100	1,1050	0,2931
11	chr11:28629363:A:G	28629363	G	0,3507	A	3,6550	0,0559	0,7477	0,1531	0,5539	1,0100	1,1050	0,2931
11	chr11:28658596:T:C	28658596	C	0,2021	T	2,3970	0,1216	0,7627	0,1774	0,5387	1,0800	1,0990	0,2946
5	chr5:104577563:A:C	104577563	C	0,3396	A	0,1794	0,6719	1,0660	0,1526	0,7907	1,4380	1,0960	0,2951
5	chr5:104577567:C:T	104577567	T	0,3396	C	0,1794	0,6719	1,0660	0,1526	0,7907	1,4380	1,0960	0,2951
5	chr5:104577620:T:A	104577620	A	0,3396	T	0,1794	0,6719	1,0660	0,1526	0,7907	1,4380	1,0960	0,2951
5	chr5:104578055:A:T	104578055	T	0,3396	A	0,1794	0,6719	1,0660	0,1526	0,7907	1,4380	1,0960	0,2951
5	chr5:104578313:C:T	104578313	T	0,3396	C	0,1794	0,6719	1,0660	0,1526	0,7907	1,4380	1,0960	0,2951
5	chr5:104578842:G:A	104578842	A	0,3396	G	0,1794	0,6719	1,0660	0,1526	0,7907	1,4380	1,0960	0,2951
5	chr5:104579057:A:G	104579057	G	0,3396	A	0,1794	0,6719	1,0660	0,1526	0,7907	1,4380	1,0960	0,2951
5	chr5:104579085:A:T	104579085	T	0,3396	A	0,1794	0,6719	1,0660	0,1526	0,7907	1,4380	1,0960	0,2951
5	chr5:104579135:T:G	104579135	G	0,3396	T	0,1794	0,6719	1,0660	0,1526	0,7907	1,4380	1,0960	0,2951
5	chr5:104579581:A:G	104579581	G	0,3396	A	0,1794	0,6719	1,0660	0,1526	0,7907	1,4380	1,0960	0,2951
5	chr5:104579742:G:A	104579742	A	0,3396	G	0,1794	0,6719	1,0660	0,1526	0,7907	1,4380	1,0960	0,2951
5	chr5:104580027:A:G	104580027	G	0,3396	A	0,1794	0,6719	1,0660	0,1526	0,7907	1,4380	1,0960	0,2951
5	chr5:104580648:T:C	104580648	C	0,3396	T	0,1794	0,6719	1,0660	0,1526	0,7907	1,4380	1,0960	0,2951
5	chr5:104580662:G:C	104580662	C	0,3396	G	0,1794	0,6719	1,0660	0,1526	0,7907	1,4380	1,0960	0,2951
5	chr5:104581130:A:G	104581130	G	0,3396	A	0,1794	0,6719	1,0660	0,1526	0,7907	1,4380	1,0960	0,2951
20	chr20:21155077:G:T	21155077	G	0,4707	T	4,2360	0,0396	1,3610	0,1499	1,0140	1,8260	1,0840	0,2977
20	chr20:21173632:G:C	21173632	G	0,4819	C	3,1440	0,0762	1,3030	0,1496	0,9721	1,7480	1,0780	0,2992
20	chr20:21194910:T:C	21194910	C	0,4167	T	1,9130	0,1667	1,2350	0,1522	0,9162	1,6640	1,0780	0,2992
5	chr5:104604069:C:T	104604069	T	0,3535	C	0,6910	0,4058	1,1350	0,1529	0,8409	1,5310	1,0720	0,3005
11	chr11:28554714:G:T	28554714	G	0,2395	T	0,0866	0,7686	1,0520	0,1752	0,7465	1,4840	1,0510	0,3053
20	chr20:21073855:C:T	21073855	C	0,3482	T	0,8367	0,3604	1,1590	0,1604	0,8463	1,5870	1,0500	0,3054

5	chr5:104678081:G:A	104678081	G	0,2452	A	2,4030	0,1211	0,7631	0,1728	0,5439	1,0710	1,0460	0,3064
5	chr5:104655775:C:A	104655775	C	0,2405	A	1,6910	0,1935	0,7967	0,1730	0,5676	1,1180	1,0340	0,3092
5	chr5:88628064:T:G	88628064	G	0,4429	T	0,4626	0,4964	1,1100	0,1533	0,8217	1,4990	1,0220	0,312
20	chr20:21195965:C:T	21195965	T	0,4226	C	2,9490	0,0859	1,3110	0,1575	0,9626	1,7850	1,0180	0,313
1	chr1:43883733:G:A	43883733	G	0,3600	A	0,0411	0,8393	1,0320	0,1581	0,7573	1,4080	1,0140	0,3139
5	chr5:104577501:A:G	104577501	G	0,3399	A	0,1925	0,6609	1,0690	0,1529	0,7921	1,4430	1,0110	0,3147
5	chr5:104665051:G:A	104665051	G	0,2400	A	1,7560	0,1852	0,7930	0,1733	0,5646	1,1140	1,0070	0,3157
5	chr5:104582527:C:T	104582527	T	0,3414	C	0,2138	0,6438	1,0720	0,1520	0,7962	1,4440	1,0020	0,3169
20	chr20:21169993:T:C	21169993	T	0,4667	C	2,8500	0,0914	1,2890	0,1503	0,9598	1,7300	0,9949	0,3185
20	chr20:21173142:G:C	21173142	G	0,4667	C	2,8500	0,0914	1,2890	0,1503	0,9598	1,7300	0,9949	0,3185
5	chr5:88588020:G:C	88588020	C	0,4571	G	0,9971	0,3180	1,1640	0,1524	0,8637	1,5690	0,9948	0,3186
5	chr5:104584139:G:A	104584139	A	0,3408	G	0,2251	0,6352	1,0750	0,1525	0,7970	1,4490	0,9847	0,321
5	chr5:88587685:T:C	88587685	C	0,4572	T	1,0200	0,3126	1,1660	0,1523	0,8653	1,5720	0,9782	0,3226
11	chr11:28617583:T:C	28617583	C	0,3423	T	1,5270	0,2166	0,8272	0,1545	0,6111	1,1200	0,9667	0,3255
5	chr5:104563895:T:C	104563895	C	0,3781	T	0,2797	0,5969	1,0830	0,1508	0,8056	1,4550	0,9652	0,3259
5	chr5:104581651:A:C	104581651	C	0,3411	A	0,2414	0,6232	1,0770	0,1523	0,7993	1,4520	0,9594	0,3273
5	chr5:104582166:G:T	104582166	T	0,3411	G	0,2414	0,6232	1,0770	0,1523	0,7993	1,4520	0,9594	0,3273
5	chr5:104582921:C:T	104582921	T	0,3411	C	0,2414	0,6232	1,0770	0,1523	0,7993	1,4520	0,9594	0,3273
5	chr5:104582998:G:A	104582998	A	0,3411	G	0,2414	0,6232	1,0770	0,1523	0,7993	1,4520	0,9594	0,3273
5	chr5:104583048:G:T	104583048	T	0,3411	G	0,2414	0,6232	1,0770	0,1523	0,7993	1,4520	0,9594	0,3273
5	chr5:104584121:A:C	104584121	C	0,3411	A	0,2414	0,6232	1,0770	0,1523	0,7993	1,4520	0,9594	0,3273
1	chr1:43663428:T:C	43663428	T	0,4990	C	0,0968	0,7557	0,9540	0,1511	0,7095	1,2830	0,9405	0,3322
11	chr11:28627190:A:G	28627190	G	0,3530	A	3,6370	0,0565	0,7497	0,1521	0,5565	1,0100	0,9316	0,3344
20	chr20:21187721:G:T	21187721	T	0,4176	G	1,6690	0,1964	1,2170	0,1520	0,9037	1,6400	0,9315	0,3345
20	chr20:21190532:C:T	21190532	T	0,4176	C	1,6690	0,1964	1,2170	0,1520	0,9037	1,6400	0,9315	0,3345
5	chr5:104576507:C:A	104576507	A	0,3409	C	0,0294	0,8638	1,0260	0,1529	0,7606	1,3850	0,9235	0,3366
5	chr5:104565723:C:T	104565723	T	0,3724	C	0,6520	0,4194	1,1290	0,1511	0,8397	1,5190	0,9189	0,3378
5	chr5:104568065:G:A	104568065	A	0,3724	G	0,6520	0,4194	1,1290	0,1511	0,8397	1,5190	0,9189	0,3378
11	chr11:28619515:G:A	28619515	A	0,3400	G	1,2480	0,2639	0,8423	0,1546	0,6221	1,1400	0,9166	0,3384
11	chr11:28619698:G:A	28619698	A	0,3400	G	1,2480	0,2639	0,8423	0,1546	0,6221	1,1400	0,9166	0,3384
11	chr11:28620721:G:A	28620721	A	0,3400	G	1,2480	0,2639	0,8423	0,1546	0,6221	1,1400	0,9166	0,3384
5	chr5:104569213:T:C	104569213	C	0,3529	T	0,4184	0,5177	1,1030	0,1523	0,8183	1,4870	0,9010	0,3425
11	chr11:28621752:T:C	28621752	T	0,3745	C	4,0120	0,0452	0,7365	0,1536	0,5450	0,9952	0,8990	0,343
11	chr11:28639315:G:T	28639315	T	0,3433	G	4,0370	0,0445	0,7370	0,1529	0,5461	0,9946	0,8960	0,3438
5	chr5:104670966:A:G	104670966	A	0,2380	G	2,1330	0,1442	0,7740	0,1738	0,5506	1,0880	0,8684	0,3514
5	chr5:104576413:A:C	104576413	C	0,3440	A	0,0105	0,9185	1,0160	0,1531	0,7524	1,3710	0,8476	0,3572
11	chr11:28620773:G:A	28620773	A	0,3410	G	1,1640	0,2806	0,8471	0,1546	0,6257	1,1470	0,8337	0,3612
11	chr11:28603810:A:T	28603810	T	0,3324	A	1,7540	0,1854	0,8145	0,1559	0,6001	1,1060	0,8319	0,3617
20	chr20:21165245:A:G	21165245	A	0,4558	G	1,7670	0,1837	1,2170	0,1477	0,9111	1,6260	0,8226	0,3644
1	chr1:43682946:A:G	43682946	G	0,1108	A	0,5011	0,4790	1,1790	0,2282	0,7535	1,8430	0,8212	0,3648
11	chr11:28632740:G:A	28632740	A	0,3442	G	3,8020	0,0512	0,7438	0,1528	0,5512	1,0030	0,8190	0,3655
11	chr11:28633321:G:A	28633321	A	0,3442	G	3,8020	0,0512	0,7438	0,1528	0,5512	1,0030	0,8190	0,3655

11	chr11:28633511:A:C	28633511	C	0,3442	A	3,8020	0,0512	0,7438	0,1528	0,5512	1,0030	0,8190	0,3655
11	chr11:28634125:G:T	28634125	T	0,3442	G	3,8020	0,0512	0,7438	0,1528	0,5512	1,0030	0,8190	0,3655
11	chr11:28634351:C:G	28634351	G	0,3442	C	3,8020	0,0512	0,7438	0,1528	0,5512	1,0030	0,8190	0,3655
11	chr11:28634462:T:G	28634462	G	0,3442	T	3,8020	0,0512	0,7438	0,1528	0,5512	1,0030	0,8190	0,3655
11	chr11:28635703:A:G	28635703	G	0,3442	A	3,8020	0,0512	0,7438	0,1528	0,5512	1,0030	0,8190	0,3655
11	chr11:28637410:G:A	28637410	A	0,3442	G	3,8020	0,0512	0,7438	0,1528	0,5512	1,0030	0,8190	0,3655
11	chr11:28638055:A:T	28638055	T	0,3442	A	3,8020	0,0512	0,7438	0,1528	0,5512	1,0030	0,8190	0,3655
11	chr11:28638658:T:C	28638658	C	0,3442	T	3,8020	0,0512	0,7438	0,1528	0,5512	1,0030	0,8190	0,3655
11	chr11:28644512:C:G	28644512	G	0,3442	C	3,8020	0,0512	0,7438	0,1528	0,5512	1,0030	0,8190	0,3655
11	chr11:28644517:G:A	28644517	A	0,3442	G	3,8020	0,0512	0,7438	0,1528	0,5512	1,0030	0,8190	0,3655
11	chr11:28644793:A:C	28644793	C	0,3442	A	3,8020	0,0512	0,7438	0,1528	0,5512	1,0030	0,8190	0,3655
5	chr5:104603973:C:T	104603973	T	0,3518	C	0,5453	0,4602	1,1180	0,1519	0,8303	1,5060	0,8112	0,3678
5	chr5:104606092:C:A	104606092	A	0,3585	C	0,3854	0,5347	1,0980	0,1520	0,8155	1,4800	0,8036	0,37
20	chr20:21372771:A:C	21372771	A	0,2634	C	2,9390	0,0865	1,3420	0,1705	0,9608	1,8740	0,8008	0,3709
11	chr11:28631941:T:G	28631941	G	0,3483	T	3,7160	0,0539	0,7458	0,1531	0,5525	1,0070	0,7977	0,3718
11	chr11:28632083:T:C	28632083	C	0,3483	T	3,7160	0,0539	0,7458	0,1531	0,5525	1,0070	0,7977	0,3718
11	chr11:28632180:T:G	28632180	G	0,3483	T	3,7160	0,0539	0,7458	0,1531	0,5525	1,0070	0,7977	0,3718
11	chr11:28632479:C:T	28632479	T	0,3483	C	3,7160	0,0539	0,7458	0,1531	0,5525	1,0070	0,7977	0,3718
11	chr11:28646124:G:A	28646124	A	0,3446	G	3,6680	0,0555	0,7473	0,1531	0,5536	1,0090	0,7952	0,3725
11	chr11:28646393:C:T	28646393	T	0,3446	C	3,6680	0,0555	0,7473	0,1531	0,5536	1,0090	0,7952	0,3725
11	chr11:28646869:T:C	28646869	C	0,3446	T	3,6680	0,0555	0,7473	0,1531	0,5536	1,0090	0,7952	0,3725
11	chr11:28648417:T:C	28648417	C	0,3446	T	3,6680	0,0555	0,7473	0,1531	0,5536	1,0090	0,7952	0,3725
11	chr11:28649222:A:G	28649222	G	0,3446	A	3,6680	0,0555	0,7473	0,1531	0,5536	1,0090	0,7952	0,3725
11	chr11:28649587:T:C	28649587	C	0,3446	T	3,6680	0,0555	0,7473	0,1531	0,5536	1,0090	0,7952	0,3725
11	chr11:28649653:C:G	28649653	G	0,3446	C	3,6680	0,0555	0,7473	0,1531	0,5536	1,0090	0,7952	0,3725
11	chr11:28650803:C:G	28650803	G	0,3446	C	3,6680	0,0555	0,7473	0,1531	0,5536	1,0090	0,7952	0,3725
11	chr11:28654623:A:G	28654623	G	0,3446	A	3,6680	0,0555	0,7473	0,1531	0,5536	1,0090	0,7952	0,3725
20	chr20:21372841:G:C	21372841	G	0,2633	C	2,9550	0,0856	1,3430	0,1705	0,9615	1,8760	0,7915	0,3737
11	chr11:28631449:A:G	28631449	G	0,3453	A	3,2250	0,0725	0,7601	0,1537	0,5624	1,0270	0,7904	0,374
20	chr20:21374422:T:A	21374422	T	0,2657	A	2,5800	0,1082	1,3160	0,1697	0,9434	1,8340	0,7902	0,374
20	chr20:21375208:A:G	21375208	A	0,2657	G	2,5800	0,1082	1,3160	0,1697	0,9434	1,8340	0,7902	0,374
5	chr5:104566479:C:T	104566479	T	0,3489	C	0,3212	0,5709	1,0900	0,1528	0,8079	1,4710	0,7708	0,38
5	chr5:104567257:C:T	104567257	T	0,3489	C	0,3212	0,5709	1,0900	0,1528	0,8079	1,4710	0,7708	0,38
5	chr5:104572545:C:T	104572545	T	0,3489	C	0,3212	0,5709	1,0900	0,1528	0,8079	1,4710	0,7708	0,38
5	chr5:104572709:A:G	104572709	G	0,3489	A	0,3212	0,5709	1,0900	0,1528	0,8079	1,4710	0,7708	0,38
5	chr5:104573167:C:T	104573167	T	0,3489	C	0,3212	0,5709	1,0900	0,1528	0,8079	1,4710	0,7708	0,38
5	chr5:104573847:C:T	104573847	T	0,3489	C	0,3212	0,5709	1,0900	0,1528	0,8079	1,4710	0,7708	0,38
20	chr20:21154956:G:T	21154956	G	0,4660	T	4,0200	0,0450	1,3510	0,1500	1,0060	1,8120	0,7662	0,3814
11	chr11:28658309:G:A	28658309	A	0,3453	G	3,8030	0,0512	0,7431	0,1532	0,5503	1,0030	0,7614	0,3829
7	chr7:114434202:C:T	114434202	C	0,4046	T	0,2569	0,6123	1,0780	0,1490	0,8052	1,4440	0,7534	0,3854
5	chr5:104590741:T:A	104590741	A	0,4621	T	0,0847	0,7710	0,9570	0,1509	0,7120	1,2860	0,7433	0,3886
5	chr5:104600642:T:A	104600642	A	0,4621	T	0,0847	0,7710	0,9570	0,1509	0,7120	1,2860	0,7433	0,3886

11	chr11:28655190:A:G	28655190	G	0,3456	A	3,7470	0,0529	0,7449	0,1531	0,5519	1,0060	0,7412	0,3893
11	chr11:28655717:T:C	28655717	C	0,3456	T	3,7470	0,0529	0,7449	0,1531	0,5519	1,0060	0,7412	0,3893
20	chr20:21328174:G:A	21328174	G	0,2644	A	2,2450	0,1341	1,2940	0,1710	0,9256	1,8100	0,7372	0,3906
5	chr5:104605369:T:G	104605369	G	0,3566	T	0,5437	0,4609	1,1180	0,1521	0,8299	1,5070	0,7321	0,3922
5	chr5:104605700:T:C	104605700	C	0,3566	T	0,5437	0,4609	1,1180	0,1521	0,8299	1,5070	0,7321	0,3922
5	chr5:104605714:T:G	104605714	G	0,3566	T	0,5437	0,4609	1,1180	0,1521	0,8299	1,5070	0,7321	0,3922
11	chr11:28645542:T:C	28645542	C	0,3485	T	4,0280	0,0448	0,7369	0,1531	0,5459	0,9947	0,7301	0,3929
20	chr20:21314871:G:T	21314871	G	0,2635	T	2,5830	0,1080	1,3190	0,1712	0,9430	1,8450	0,7191	0,3964
20	chr20:21350903:T:G	21350903	T	0,2648	G	2,2200	0,1362	1,2900	0,1699	0,9247	1,8000	0,7133	0,3983
11	chr11:28646269:A:G	28646269	G	0,3488	A	4,0590	0,0439	0,7360	0,1530	0,5453	0,9935	0,7101	0,3994
20	chr20:21310893:C:T	21310893	C	0,2635	T	2,4430	0,1181	1,3090	0,1712	0,9359	1,8310	0,7071	0,4004
20	chr20:21311200:C:T	21311200	C	0,2635	T	2,4430	0,1181	1,3090	0,1712	0,9359	1,8310	0,7071	0,4004
20	chr20:21311590:G:C	21311590	G	0,2635	C	2,4430	0,1181	1,3090	0,1712	0,9359	1,8310	0,7071	0,4004
20	chr20:21312173:C:T	21312173	C	0,2635	T	2,4430	0,1181	1,3090	0,1712	0,9359	1,8310	0,7071	0,4004
20	chr20:21312391:G:A	21312391	G	0,2635	A	2,4430	0,1181	1,3090	0,1712	0,9359	1,8310	0,7071	0,4004
4	chr4:111333132:A:G	111333132	G	0,0607	A	3,2090	0,0732	0,6010	0,2929	0,3385	1,0670	0,6945	0,4046
5	chr5:104597772:C:T	104597772	T	0,4671	C	0,2062	0,6498	0,9337	0,1509	0,6947	1,2550	0,6939	0,4048
20	chr20:21371667:C:T	21371667	C	0,2638	T	2,7450	0,0976	1,3270	0,1698	0,9514	1,8510	0,6878	0,4069
20	chr20:21373277:C:T	21373277	C	0,2638	T	2,7450	0,0976	1,3270	0,1698	0,9514	1,8510	0,6878	0,4069
5	chr5:104623644:A:G	104623644	G	0,4594	A	0,0057	0,9397	0,9885	0,1529	0,7325	1,3340	0,6782	0,4102
11	chr11:28654592:G:A	28654592	A	0,3494	G	4,1110	0,0426	0,7346	0,1530	0,5442	0,9915	0,6784	0,4102
20	chr20:21308964:A:G	21308964	A	0,2630	G	2,4890	0,1146	1,3120	0,1712	0,9382	1,8350	0,6779	0,4103
20	chr20:21315921:C:A	21315921	C	0,2630	A	2,4890	0,1146	1,3120	0,1712	0,9382	1,8350	0,6779	0,4103
1	chr1:43686240:C:T	43686240	T	0,1087	C	0,3025	0,5823	1,1370	0,2297	0,7248	1,7830	0,6768	0,4107
20	chr20:21313246:C:T	21313246	C	0,2630	T	2,3520	0,1251	1,3020	0,1712	0,9311	1,8210	0,6666	0,4142
20	chr20:21379890:A:G	21379890	A	0,2633	G	2,7950	0,0946	1,3310	0,1698	0,9538	1,8560	0,6591	0,4169
20	chr20:21388294:G:T	21388294	G	0,2633	T	2,7950	0,0946	1,3310	0,1698	0,9538	1,8560	0,6591	0,4169
11	chr11:28582122:A:G	28582122	G	0,3437	A	2,3910	0,1220	0,7872	0,1555	0,5804	1,0680	0,6560	0,418
11	chr11:28585152:T:C	28585152	C	0,3437	T	2,3910	0,1220	0,7872	0,1555	0,5804	1,0680	0,6560	0,418
11	chr11:28587183:C:G	28587183	G	0,3437	C	2,3910	0,1220	0,7872	0,1555	0,5804	1,0680	0,6560	0,418
11	chr11:28593837:A:G	28593837	G	0,3437	A	2,3910	0,1220	0,7872	0,1555	0,5804	1,0680	0,6560	0,418
11	chr11:28594152:A:G	28594152	G	0,3437	A	2,3910	0,1220	0,7872	0,1555	0,5804	1,0680	0,6560	0,418
11	chr11:28594153:T:C	28594153	C	0,3437	T	2,3910	0,1220	0,7872	0,1555	0,5804	1,0680	0,6560	0,418
11	chr11:28594704:T:C	28594704	C	0,3437	T	2,3910	0,1220	0,7872	0,1555	0,5804	1,0680	0,6560	0,418
11	chr11:28597209:G:A	28597209	A	0,3437	G	2,3910	0,1220	0,7872	0,1555	0,5804	1,0680	0,6560	0,418
11	chr11:28632994:A:G	28632994	G	0,3537	A	4,1440	0,0418	0,7338	0,1529	0,5438	0,9903	0,6487	0,4206
11	chr11:28639271:A:G	28639271	G	0,3537	A	4,1440	0,0418	0,7338	0,1529	0,5438	0,9903	0,6487	0,4206
20	chr20:21351811:A:G	21351811	A	0,2633	G	2,6500	0,1036	1,3210	0,1698	0,9467	1,8420	0,6480	0,4208
20	chr20:21352024:G:C	21352024	G	0,2633	C	2,6500	0,1036	1,3210	0,1698	0,9467	1,8420	0,6480	0,4208
20	chr20:21353013:G:C	21353013	G	0,2633	C	2,6500	0,1036	1,3210	0,1698	0,9467	1,8420	0,6480	0,4208
20	chr20:21353030:G:A	21353030	G	0,2633	A	2,6500	0,1036	1,3210	0,1698	0,9467	1,8420	0,6480	0,4208
20	chr20:21359219:A:G	21359219	A	0,2633	G	2,6500	0,1036	1,3210	0,1698	0,9467	1,8420	0,6480	0,4208

20	chr20:21361740:T:C	21361740	T	0,2633	C	2,6500	0,1036	1,3210	0,1698	0,9467	1,8420	0,6480	0,4208
11	chr11:28585698:T:C	28585698	C	0,3434	T	2,3400	0,1261	0,7889	0,1559	0,5812	1,0710	0,6422	0,4229
1	chr1:43541977:T:C	43541977	T	0,2229	C	3,5340	0,0601	1,4140	0,1864	0,9812	2,0380	0,6410	0,4233
11	chr11:28648570:G:T	28648570	T	0,3539	G	4,0490	0,0442	0,7358	0,1533	0,5449	0,9937	0,6393	0,424
11	chr11:28648575:T:A	28648575	A	0,3539	T	4,0490	0,0442	0,7358	0,1533	0,5449	0,9937	0,6393	0,424
20	chr20:21318093:T:C	21318093	T	0,2625	C	2,3970	0,1215	1,3060	0,1712	0,9334	1,8260	0,6382	0,4243
20	chr20:21318094:G:T	21318094	G	0,2625	T	2,3970	0,1215	1,3060	0,1712	0,9334	1,8260	0,6382	0,4243
20	chr20:21319392:G:A	21319392	G	0,2625	A	2,3970	0,1215	1,3060	0,1712	0,9334	1,8260	0,6382	0,4243
20	chr20:21320839:C:T	21320839	C	0,2625	T	2,3970	0,1215	1,3060	0,1712	0,9334	1,8260	0,6382	0,4243
20	chr20:21321927:C:T	21321927	C	0,2625	T	2,3970	0,1215	1,3060	0,1712	0,9334	1,8260	0,6382	0,4243
20	chr20:21321943:T:C	21321943	T	0,2625	C	2,3970	0,1215	1,3060	0,1712	0,9334	1,8260	0,6382	0,4243
20	chr20:21327058:G:A	21327058	G	0,2625	A	2,3970	0,1215	1,3060	0,1712	0,9334	1,8260	0,6382	0,4243
20	chr20:21329677:C:T	21329677	C	0,2625	T	2,3970	0,1215	1,3060	0,1712	0,9334	1,8260	0,6382	0,4243
20	chr20:21345460:T:C	21345460	T	0,2633	C	2,5090	0,1132	1,3110	0,1698	0,9396	1,8280	0,6374	0,4246
20	chr20:21346867:T:G	21346867	T	0,2633	G	2,5090	0,1132	1,3110	0,1698	0,9396	1,8280	0,6374	0,4246
20	chr20:21349142:T:C	21349142	T	0,2633	C	2,5090	0,1132	1,3110	0,1698	0,9396	1,8280	0,6374	0,4246
20	chr20:21350734:G:A	21350734	G	0,2633	A	2,5090	0,1132	1,3110	0,1698	0,9396	1,8280	0,6374	0,4246
20	chr20:21189014:G:A	21189014	A	0,4013	G	1,0900	0,2964	1,1740	0,1535	0,8691	1,5860	0,6220	0,4303
20	chr20:21390733:A:G	21390733	A	0,2628	G	2,6860	0,1012	1,3240	0,1701	0,9483	1,8470	0,6190	0,4314
5	chr5:104609477:C:T	104609477	T	0,4587	C	0,0463	0,8297	0,9676	0,1527	0,7174	1,3050	0,5977	0,4394
20	chr20:21299701:C:T	21299701	C	0,2653	T	1,9350	0,1643	1,2700	0,1706	0,9088	1,7730	0,5839	0,4448
20	chr20:21371598:A:G	21371598	A	0,2564	G	3,5090	0,0610	1,3750	0,1691	0,9870	1,9150	0,5840	0,4448
1	chr1:43546066:G:A	43546066	G	0,2360	A	1,1260	0,2886	1,2100	0,1815	0,8481	1,7280	0,5738	0,4488
20	chr20:21291031:G:C	21291031	G	0,2637	C	1,5110	0,2189	1,2390	0,1734	0,8822	1,7410	0,5724	0,4493
1	chr1:43658195:A:G	43658195	A	0,4407	G	0,1292	0,7193	0,9483	0,1478	0,7098	1,2670	0,5638	0,4527
20	chr20:21283152:A:T	21283152	A	0,2631	T	1,6670	0,1966	1,2520	0,1732	0,8919	1,7590	0,5582	0,455
20	chr20:21284820:G:A	21284820	G	0,2631	A	1,6670	0,1966	1,2520	0,1732	0,8919	1,7590	0,5582	0,455
20	chr20:21285090:C:T	21285090	C	0,2631	T	1,6670	0,1966	1,2520	0,1732	0,8919	1,7590	0,5582	0,455
20	chr20:21285802:C:T	21285802	C	0,2631	T	1,6670	0,1966	1,2520	0,1732	0,8919	1,7590	0,5582	0,455
20	chr20:21286713:C:T	21286713	C	0,2631	T	1,6670	0,1966	1,2520	0,1732	0,8919	1,7590	0,5582	0,455
5	chr5:104707455:C:T	104707455	C	0,2429	T	1,7720	0,1831	0,7950	0,1711	0,5685	1,1120	0,5446	0,4605
1	chr1:43705540:C:T	43705540	T	0,1085	C	0,2553	0,6134	1,1240	0,2286	0,7183	1,7600	0,5441	0,4607
20	chr20:21283529:T:C	21283529	T	0,2626	C	1,6940	0,1931	1,2550	0,1732	0,8934	1,7620	0,5397	0,4626
11	chr11:28582930:C:T	28582930	T	0,3366	C	2,0250	0,1547	0,8022	0,1556	0,5914	1,0880	0,5359	0,4641
1	chr1:43903772:G:A	43903772	G	0,3883	A	0,1398	0,7085	0,9442	0,1539	0,6983	1,2770	0,5348	0,4646
5	chr5:104619660:T:C	104619660	C	0,4604	T	0,1051	0,7458	0,9516	0,1528	0,7052	1,2840	0,5322	0,4657
5	chr5:104596006:G:A	104596006	A	0,4670	G	0,3663	0,5451	0,9125	0,1511	0,6786	1,2270	0,5273	0,4677
4	chr4:111310701:T:C	111310701	C	0,0762	T	2,5950	0,1072	0,6569	0,2666	0,3895	1,1080	0,5227	0,4697
5	chr5:104602414:C:T	104602414	T	0,3383	C	0,0878	0,7670	1,0460	0,1538	0,7741	1,4150	0,5202	0,4707
5	chr5:88871923:G:C	88871923	G	0,2887	C	1,8710	0,1714	0,7906	0,1730	0,5632	1,1100	0,5158	0,4726
20	chr20:21281046:C:T	21281046	C	0,2621	T	1,7300	0,1884	1,2580	0,1733	0,8956	1,7660	0,5148	0,4731
5	chr5:88874249:T:C	88874249	T	0,2814	C	1,2180	0,2698	0,8261	0,1743	0,5870	1,1620	0,5116	0,4744

20	chr20:21289658:C:T	21289658	C	0,2626	T	1,4730	0,2249	1,2360	0,1734	0,8797	1,7360	0,5110	0,4747
20	chr20:21291893:C:T	21291893	C	0,2626	T	1,4730	0,2249	1,2360	0,1734	0,8797	1,7360	0,5110	0,4747
5	chr5:104709685:C:A	104709685	C	0,2381	A	2,1810	0,1397	0,7781	0,1687	0,5590	1,0830	0,5109	0,4748
11	chr11:28636483:A:G	28636483	G	0,3493	A	3,6350	0,0566	0,7496	0,1519	0,5566	1,0100	0,5083	0,4759
20	chr20:21267478:G:A	21267478	G	0,2132	A	1,7300	0,1884	1,2870	0,1904	0,8862	1,8690	0,5068	0,4765
5	chr5:104591878:C:T	104591878	T	0,4630	C	0,0319	0,8582	0,9733	0,1514	0,7234	1,3090	0,5042	0,4777
5	chr5:104611504:C:T	104611504	T	0,4596	C	0,0956	0,7571	0,9537	0,1528	0,7069	1,2870	0,5011	0,479
5	chr5:104612344:C:T	104612344	T	0,4596	C	0,0956	0,7571	0,9537	0,1528	0,7069	1,2870	0,5011	0,479
5	chr5:88872578:C:G	88872578	C	0,2894	G	1,9010	0,1679	0,7891	0,1730	0,5622	1,1080	0,4920	0,4831
5	chr5:88873138:C:A	88873138	C	0,2894	A	1,9010	0,1679	0,7891	0,1730	0,5622	1,1080	0,4920	0,4831
5	chr5:104629522:A:C	104629522	C	0,4555	A	0,0064	0,9364	0,9878	0,1530	0,7319	1,3330	0,4902	0,4838
7	chr7:114435631:A:C	114435631	A	0,4036	C	0,1437	0,7046	1,0580	0,1491	0,7899	1,4170	0,4893	0,4842
20	chr20:21287631:G:A	21287631	G	0,2621	A	1,5070	0,2196	1,2390	0,1734	0,8818	1,7400	0,4868	0,4854
20	chr20:21290685:C:G	21290685	C	0,2621	G	1,5070	0,2196	1,2390	0,1734	0,8818	1,7400	0,4868	0,4854
20	chr20:21290915:A:G	21290915	A	0,2621	G	1,5070	0,2196	1,2390	0,1734	0,8818	1,7400	0,4868	0,4854
20	chr20:21290947:G:A	21290947	G	0,2621	A	1,5070	0,2196	1,2390	0,1734	0,8818	1,7400	0,4868	0,4854
20	chr20:21291136:G:T	21291136	G	0,2621	T	1,5070	0,2196	1,2390	0,1734	0,8818	1,7400	0,4868	0,4854
20	chr20:21292673:G:A	21292673	G	0,2621	A	1,5070	0,2196	1,2390	0,1734	0,8818	1,7400	0,4868	0,4854
5	chr5:88873835:A:G	88873835	A	0,2915	G	1,9630	0,1612	0,7860	0,1731	0,5599	1,1030	0,4818	0,4876
5	chr5:88874514:T:C	88874514	T	0,2915	C	1,9630	0,1612	0,7860	0,1731	0,5599	1,1030	0,4818	0,4876
4	chr4:111296654:C:T	111296654	T	0,0514	C	0,2788	0,5975	0,8448	0,3137	0,4568	1,5620	0,4789	0,4889
1	chr1:43649351:A:G	43649351	A	0,4750	G	0,3373	0,5614	0,9190	0,1453	0,6913	1,2220	0,4721	0,492
20	chr20:21277415:A:G	21277415	A	0,2617	G	1,5320	0,2158	1,2410	0,1735	0,8833	1,7440	0,4695	0,4932
1	chr1:43572014:G:A	43572014	G	0,4625	A	0,4296	0,5122	1,1000	0,1453	0,8272	1,4620	0,4674	0,4942
11	chr11:28586227:G:A	28586227	A	0,3408	G	2,9670	0,0850	0,7651	0,1562	0,5633	1,0390	0,4671	0,4943
20	chr20:21294655:A:C	21294655	A	0,2639	C	1,5640	0,2110	1,2400	0,1710	0,8868	1,7340	0,4651	0,4952
11	chr11:28671984:T:G	28671984	G	0,3518	T	3,1180	0,0774	0,7633	0,1537	0,5648	1,0320	0,4618	0,4968
11	chr11:28672893:C:G	28672893	G	0,3518	C	3,1180	0,0774	0,7633	0,1537	0,5648	1,0320	0,4618	0,4968
7	chr7:114531090:A:G	114531090	A	0,4142	G	0,3582	0,5495	1,0930	0,1494	0,8158	1,4650	0,4611	0,4971
20	chr20:21291449:A:T	21291449	A	0,2617	T	1,4250	0,2326	1,2320	0,1737	0,8764	1,7310	0,4518	0,5015
11	chr11:28669740:C:T	28669740	T	0,3524	C	3,2390	0,0719	0,7599	0,1533	0,5627	1,0260	0,4504	0,5022
5	chr5:104608319:G:T	104608319	T	0,4616	G	0,1731	0,6773	0,9383	0,1527	0,6956	1,2660	0,4495	0,5026
7	chr7:114442439:T:C	114442439	T	0,4171	C	0,1695	0,6806	1,0620	0,1470	0,7964	1,4170	0,4482	0,5032
11	chr11:28621106:G:A	28621106	A	0,3304	G	1,0350	0,3090	0,8521	0,1580	0,6252	1,1610	0,4432	0,5056
4	chr4:111322536:C:G	111322536	G	0,0754	C	2,2230	0,1360	0,6756	0,2684	0,3992	1,1430	0,4429	0,5057
20	chr20:21299182:T:G	21299182	T	0,2634	G	1,6020	0,2056	1,2430	0,1710	0,8891	1,7380	0,4413	0,5065
1	chr1:43621825:C:T	43621825	C	0,4646	T	0,5976	0,4395	0,8932	0,1461	0,6707	1,1890	0,4408	0,5067
1	chr1:43662376:A:G	43662376	A	0,4760	G	0,4394	0,5074	0,9086	0,1446	0,6844	1,2060	0,4390	0,5076
11	chr11:28621014:G:C	28621014	C	0,3417	G	1,6530	0,1986	0,8196	0,1554	0,6045	1,1110	0,4382	0,508
11	chr11:28666860:G:A	28666860	A	0,3527	G	3,2680	0,0707	0,7590	0,1532	0,5621	1,0250	0,4349	0,5096
7	chr7:114476826:G:A	114476826	G	0,3406	A	0,4851	0,4861	0,8974	0,1559	0,6611	1,2180	0,4323	0,5109
20	chr20:21277855:T:C	21277855	T	0,2607	C	1,5930	0,2069	1,2460	0,1736	0,8870	1,7520	0,4296	0,5122

20	chr20:21277966:T:C	21277966	T	0,2607	C	1,5930	0,2069	1,2460	0,1736	0,8870	1,7520	0,4296	0,5122
5	chr5:104610781:C:T	104610781	T	0,4638	C	0,2045	0,6511	0,9332	0,1527	0,6918	1,2590	0,4290	0,5125
5	chr5:104609425:A:G	104609425	G	0,4636	A	0,2039	0,6516	0,9332	0,1528	0,6916	1,2590	0,4234	0,5152
5	chr5:104610505:A:T	104610505	T	0,4607	A	0,1595	0,6896	0,9406	0,1530	0,6969	1,2700	0,4228	0,5155
5	chr5:104628884:G:A	104628884	A	0,1667	G	2,4770	0,1155	0,7380	0,1915	0,5070	1,0740	0,4203	0,5168
5	chr5:104698439:C:T	104698439	C	0,2429	T	2,2480	0,1338	0,7724	0,1712	0,5523	1,0800	0,4202	0,5168
7	chr7:114435085:T:C	114435085	T	0,4054	C	0,1155	0,7339	1,0520	0,1491	0,7854	1,4090	0,4189	0,5175
5	chr5:104608237:C:G	104608237	G	0,4607	C	0,1590	0,6901	0,9408	0,1527	0,6975	1,2690	0,4168	0,5185
20	chr20:21293229:C:T	21293229	C	0,2644	T	1,4520	0,2282	1,2300	0,1712	0,8798	1,7210	0,4161	0,5189
20	chr20:21294030:G:A	21294030	G	0,2644	A	1,4520	0,2282	1,2300	0,1712	0,8798	1,7210	0,4161	0,5189
20	chr20:21268900:C:T	21268900	C	0,2650	T	1,9670	0,1608	1,2730	0,1714	0,9100	1,7820	0,4134	0,5202
1	chr1:43712399:T:G	43712399	T	0,4759	G	0,2601	0,6101	0,9285	0,1455	0,6981	1,2350	0,4047	0,5247
7	chr7:114484605:C:G	114484605	C	0,3416	G	0,5160	0,4726	0,8944	0,1559	0,6589	1,2140	0,3958	0,5293
20	chr20:21400134:C:A	21400134	C	0,4568	A	0,4377	0,5083	1,1020	0,1469	0,8264	1,4700	0,3958	0,5293
1	chr1:43900578:G:A	43900578	A	0,1296	G	0,4408	0,5068	1,1580	0,2180	0,7551	1,7750	0,3954	0,5294
11	chr11:28590845:G:A	28590845	A	0,3366	G	2,2280	0,1355	0,7926	0,1563	0,5834	1,0770	0,3918	0,5313
11	chr11:28592943:A:T	28592943	T	0,3366	A	2,2280	0,1355	0,7926	0,1563	0,5834	1,0770	0,3918	0,5313
11	chr11:28596075:C:A	28596075	A	0,3366	C	2,2280	0,1355	0,7926	0,1563	0,5834	1,0770	0,3918	0,5313
11	chr11:28596496:C:G	28596496	G	0,3366	C	2,2280	0,1355	0,7926	0,1563	0,5834	1,0770	0,3918	0,5313
11	chr11:28596864:G:A	28596864	A	0,3366	G	2,2280	0,1355	0,7926	0,1563	0,5834	1,0770	0,3918	0,5313
5	chr5:104642625:G:A	104642625	A	0,4557	G	0,0122	0,9122	0,9834	0,1516	0,7306	1,3240	0,3885	0,5331
11	chr11:28580626:C:A	28580626	A	0,3369	C	2,3130	0,1283	0,7889	0,1565	0,5805	1,0720	0,3846	0,5352
11	chr11:28526196:C:T	28526196	C	0,3992	T	0,1904	0,6626	0,9360	0,1518	0,6952	1,2600	0,3836	0,5357
11	chr11:28526682:A:C	28526682	A	0,3992	C	0,1904	0,6626	0,9360	0,1518	0,6952	1,2600	0,3836	0,5357
5	chr5:104712889:C:G	104712889	C	0,2405	G	1,4370	0,2306	0,8132	0,1714	0,5811	1,1380	0,3824	0,5363
1	chr1:43543362:C:T	43543362	C	0,2399	T	1,5660	0,2108	1,2520	0,1809	0,8783	1,7850	0,3809	0,5371
1	chr1:43563682:C:G	43563682	G	0,4575	C	1,3000	0,2542	1,1800	0,1456	0,8873	1,5700	0,3809	0,5371
7	chr7:114381815:T:C	114381815	C	0,1636	T	0,0025	0,9598	0,9903	0,1916	0,6803	1,4420	0,3789	0,5382
4	chr4:111311550:T:C	111311550	C	0,0636	T	3,0000	0,0833	0,6131	0,2891	0,3479	1,0810	0,3774	0,539
4	chr4:111328737:T:G	111328737	G	0,0636	T	3,0000	0,0833	0,6131	0,2891	0,3479	1,0810	0,3774	0,539
20	chr20:21428075:A:G	21428075	G	0,4443	A	0,2718	0,6021	1,0790	0,1463	0,8103	1,4380	0,3724	0,5417
1	chr1:43637827:A:G	43637827	A	0,4779	G	0,4938	0,4822	0,9034	0,1445	0,6805	1,1990	0,3699	0,543
1	chr1:43638043:G:A	43638043	G	0,4779	A	0,4938	0,4822	0,9034	0,1445	0,6805	1,1990	0,3699	0,543
1	chr1:43639333:C:T	43639333	C	0,4779	T	0,4938	0,4822	0,9034	0,1445	0,6805	1,1990	0,3699	0,543
1	chr1:43640277:T:C	43640277	T	0,4779	C	0,4938	0,4822	0,9034	0,1445	0,6805	1,1990	0,3699	0,543
1	chr1:43640595:C:T	43640595	C	0,4779	T	0,4938	0,4822	0,9034	0,1445	0,6805	1,1990	0,3699	0,543
1	chr1:43640845:C:A	43640845	C	0,4779	A	0,4938	0,4822	0,9034	0,1445	0,6805	1,1990	0,3699	0,543
1	chr1:43641315:G:T	43641315	G	0,4779	T	0,4938	0,4822	0,9034	0,1445	0,6805	1,1990	0,3699	0,543
1	chr1:43642285:C:T	43642285	C	0,4779	T	0,4938	0,4822	0,9034	0,1445	0,6805	1,1990	0,3699	0,543
1	chr1:43642448:T:A	43642448	T	0,4779	A	0,4938	0,4822	0,9034	0,1445	0,6805	1,1990	0,3699	0,543
1	chr1:43643528:A:G	43643528	A	0,4779	G	0,4938	0,4822	0,9034	0,1445	0,6805	1,1990	0,3699	0,543
1	chr1:43644545:A:C	43644545	A	0,4779	C	0,4938	0,4822	0,9034	0,1445	0,6805	1,1990	0,3699	0,543

1	chr1:43644818:A:G	43644818	A	0,4779	G	0,4938	0,4822	0,9034	0,1445	0,6805	1,1990	0,3699	0,543
1	chr1:43645516:A:G	43645516	A	0,4779	G	0,4938	0,4822	0,9034	0,1445	0,6805	1,1990	0,3699	0,543
1	chr1:43646708:T:C	43646708	T	0,4779	C	0,4938	0,4822	0,9034	0,1445	0,6805	1,1990	0,3699	0,543
1	chr1:43647770:T:C	43647770	T	0,4779	C	0,4938	0,4822	0,9034	0,1445	0,6805	1,1990	0,3699	0,543
1	chr1:43648528:G:T	43648528	G	0,4779	T	0,4938	0,4822	0,9034	0,1445	0,6805	1,1990	0,3699	0,543
1	chr1:43657831:G:A	43657831	G	0,4779	A	0,4945	0,4819	0,9033	0,1446	0,6804	1,1990	0,3692	0,5434
7	chr7:114503596:T:A	114503596	T	0,4151	A	0,3503	0,5540	1,0910	0,1469	0,8179	1,4550	0,3688	0,5437
7	chr7:114483253:C:T	114483253	C	0,4179	T	0,2861	0,5927	1,0820	0,1467	0,8113	1,4420	0,3679	0,5442
5	chr5:88551455:T:A	88551455	T	0,3948	A	0,0046	0,9460	0,9894	0,1568	0,7275	1,3450	0,3672	0,5445
7	chr7:114407085:A:T	114407085	T	0,4021	A	0,6201	0,4310	1,1250	0,1503	0,8383	1,5110	0,3667	0,5448
20	chr20:21270205:T:C	21270205	T	0,2640	C	1,9110	0,1669	1,2690	0,1714	0,9068	1,7760	0,3663	0,545
4	chr4:111311007:T:C	111311007	C	0,0637	T	2,9320	0,0868	0,6164	0,2891	0,3497	1,0860	0,3653	0,5456
7	chr7:114503352:C:T	114503352	C	0,3413	T	0,4668	0,4945	0,8990	0,1563	0,6618	1,2210	0,3647	0,5459
11	chr11:28580674:A:G	28580674	G	0,3375	A	2,3510	0,1252	0,7874	0,1565	0,5794	1,0700	0,3622	0,5473
5	chr5:104620815:C:G	104620815	G	0,1676	C	2,8420	0,0919	0,7230	0,1912	0,4971	1,0520	0,3595	0,5488
5	chr5:104636656:G:A	104636656	A	0,1676	G	2,8420	0,0919	0,7230	0,1912	0,4971	1,0520	0,3595	0,5488
11	chr11:28620834:C:T	28620834	T	0,3180	C	0,7473	0,3873	0,8734	0,1571	0,6419	1,1880	0,3569	0,5503
3	chr3:20687599:C:T	20687599	T	0,3147	C	2,2510	0,1336	0,7930	0,1553	0,5849	1,0750	0,3561	0,5507
1	chr1:43548609:T:C	43548609	T	0,2196	C	2,3420	0,1259	1,3290	0,1876	0,9204	1,9200	0,3520	0,553
11	chr11:28526861:T:C	28526861	T	0,4002	C	0,2107	0,6462	0,9328	0,1517	0,6929	1,2560	0,3450	0,557
20	chr20:21422129:C:A	21422129	A	0,4230	C	0,2188	0,6400	1,0720	0,1480	0,8019	1,4330	0,3420	0,5587
7	chr7:114497885:A:G	114497885	A	0,3397	G	0,3598	0,5486	0,9108	0,1561	0,6708	1,2370	0,3401	0,5598
1	chr1:43604361:A:G	43604361	G	0,4047	A	0,6685	0,4136	1,1330	0,1530	0,8395	1,5290	0,3369	0,5616
1	chr1:43549278:T:C	43549278	T	0,2201	C	2,3160	0,1281	1,3270	0,1876	0,9190	1,9170	0,3344	0,5631
5	chr5:104554297:G:C	104554297	C	0,3008	G	3,5020	0,0613	0,7353	0,1638	0,5334	1,0140	0,3338	0,5635
1	chr1:43616091:T:G	43616091	T	0,4356	G	0,3426	0,5583	0,9168	0,1484	0,6854	1,2260	0,3288	0,5663
5	chr5:104590343:C:T	104590343	T	0,4790	C	0,0025	0,9598	0,9924	0,1506	0,7388	1,3330	0,3230	0,5698
7	chr7:114499629:G:A	114499629	G	0,4161	A	0,4187	0,5176	1,1000	0,1468	0,8246	1,4660	0,3204	0,5714
5	chr5:104612267:T:C	104612267	C	0,1664	T	3,4470	0,0634	0,6987	0,1920	0,4795	1,0180	0,3132	0,5757
7	chr7:114552490:T:C	114552490	T	0,4144	C	0,6992	0,4030	1,1330	0,1495	0,8453	1,5190	0,3098	0,5778
5	chr5:104702059:G:A	104702059	A	0,1645	G	3,2320	0,0722	0,7060	0,1925	0,4841	1,0300	0,3094	0,578
1	chr1:43548902:A:T	43548902	A	0,2321	T	0,9295	0,3350	1,1920	0,1834	0,8321	1,7080	0,3093	0,5781
7	chr7:114489082:G:A	114489082	G	0,3406	A	0,3866	0,5341	0,9077	0,1561	0,6685	1,2330	0,3078	0,579
7	chr7:114498696:G:C	114498696	G	0,3406	C	0,3866	0,5341	0,9077	0,1561	0,6685	1,2330	0,3078	0,579
7	chr7:114502195:A:G	114502195	A	0,3406	G	0,3866	0,5341	0,9077	0,1561	0,6685	1,2330	0,3078	0,579
7	chr7:114479375:G:A	114479375	G	0,3403	A	0,3173	0,5733	0,9160	0,1561	0,6745	1,2440	0,3077	0,5791
11	chr11:28521551:G:A	28521551	G	0,3982	A	0,1181	0,7311	0,9493	0,1518	0,7050	1,2780	0,3053	0,5806
5	chr5:104614550:C:T	104614550	T	0,4605	C	0,0375	0,8464	0,9707	0,1534	0,7186	1,3110	0,3000	0,5839
5	chr5:104686538:C:G	104686538	C	0,2471	G	3,2910	0,0697	0,7323	0,1710	0,5237	1,0240	0,2955	0,5867
20	chr20:21412561:A:G	21412561	G	0,4478	A	0,6003	0,4385	1,1200	0,1459	0,8413	1,4910	0,2950	0,587
1	chr1:43843195:A:G	43843195	G	0,4527	A	0,3283	0,5666	1,0890	0,1484	0,8141	1,4570	0,2946	0,5873
20	chr20:21436041:T:G	21436041	G	0,4453	T	0,1863	0,6660	1,0650	0,1462	0,7998	1,4190	0,2946	0,5873

20	chr20:21439854:C:T	21439854	T	0,4453	C	0,1863	0,6660	1,0650	0,1462	0,7998	1,4190	0,2946	0,5873
20	chr20:21440237:T:C	21440237	C	0,4453	T	0,1863	0,6660	1,0650	0,1462	0,7998	1,4190	0,2946	0,5873
20	chr20:21444512:T:A	21444512	A	0,4453	T	0,1863	0,6660	1,0650	0,1462	0,7998	1,4190	0,2946	0,5873
20	chr20:21444955:G:C	21444955	C	0,4453	G	0,1863	0,6660	1,0650	0,1462	0,7998	1,4190	0,2946	0,5873
20	chr20:21449932:G:A	21449932	A	0,4453	G	0,1863	0,6660	1,0650	0,1462	0,7998	1,4190	0,2946	0,5873
20	chr20:21201749:T:C	21201749	C	0,1462	T	3,0090	0,0828	1,4620	0,2177	0,9542	2,2400	0,2939	0,5877
7	chr7:114482451:C:T	114482451	C	0,4298	T	0,2042	0,6514	1,0680	0,1464	0,8018	1,4230	0,2903	0,59
20	chr20:21260820:G:A	21260820	G	0,3217	A	1,6810	0,1948	1,2350	0,1623	0,8985	1,6980	0,2900	0,5902
7	chr7:114475213:T:C	114475213	T	0,4170	C	0,3925	0,5310	1,0960	0,1468	0,8221	1,4620	0,2895	0,5905
7	chr7:114507162:C:A	114507162	C	0,4184	A	0,3578	0,5497	1,0930	0,1492	0,8161	1,4650	0,2868	0,5923
5	chr5:88433894:A:C	88433894	A	0,4474	C	0,2442	0,6212	1,0770	0,1492	0,8037	1,4420	0,2812	0,5959
1	chr1:43550138:T:C	43550138	T	0,2120	C	1,7150	0,1903	1,2820	0,1913	0,8814	1,8660	0,2800	0,5967
5	chr5:104619100:T:A	104619100	A	0,1657	T	3,3810	0,0660	0,7011	0,1921	0,4811	1,0220	0,2788	0,5975
20	chr20:21252915:A:G	21252915	A	0,3215	G	1,6150	0,2038	1,2290	0,1619	0,8950	1,6880	0,2787	0,5976
16	chr16:61753223:C:T	61753223	C	0,4749	T	0,2274	0,6335	1,0720	0,1463	0,8050	1,4280	0,2775	0,5983
16	chr16:61758377:A:G	61758377	A	0,4750	G	0,2294	0,6320	1,0720	0,1460	0,8056	1,4280	0,2761	0,5993
1	chr1:43840448:A:G	43840448	G	0,4612	A	0,1051	0,7458	1,0490	0,1481	0,7849	1,4030	0,2740	0,6006
1	chr1:43845072:C:T	43845072	T	0,4612	C	0,1051	0,7458	1,0490	0,1481	0,7849	1,4030	0,2740	0,6006
1	chr1:43845203:G:A	43845203	A	0,4612	G	0,1051	0,7458	1,0490	0,1481	0,7849	1,4030	0,2740	0,6006
1	chr1:43616202:A:C	43616202	A	0,4741	C	0,5723	0,4493	0,8959	0,1453	0,6739	1,1910	0,2686	0,6043
1	chr1:43617439:C:T	43617439	C	0,4741	T	0,5723	0,4493	0,8959	0,1453	0,6739	1,1910	0,2686	0,6043
1	chr1:43617836:T:C	43617836	T	0,4741	C	0,5723	0,4493	0,8959	0,1453	0,6739	1,1910	0,2686	0,6043
1	chr1:43617848:G:A	43617848	G	0,4741	A	0,5723	0,4493	0,8959	0,1453	0,6739	1,1910	0,2686	0,6043
1	chr1:43618203:A:G	43618203	A	0,4741	G	0,5723	0,4493	0,8959	0,1453	0,6739	1,1910	0,2686	0,6043
1	chr1:43619068:C:T	43619068	C	0,4741	T	0,5723	0,4493	0,8959	0,1453	0,6739	1,1910	0,2686	0,6043
1	chr1:43624269:A:G	43624269	A	0,4741	G	0,5723	0,4493	0,8959	0,1453	0,6739	1,1910	0,2686	0,6043
1	chr1:43625004:T:C	43625004	T	0,4741	C	0,5723	0,4493	0,8959	0,1453	0,6739	1,1910	0,2686	0,6043
1	chr1:43625065:C:T	43625065	C	0,4741	T	0,5723	0,4493	0,8959	0,1453	0,6739	1,1910	0,2686	0,6043
1	chr1:43625603:A:G	43625603	A	0,4741	G	0,5723	0,4493	0,8959	0,1453	0,6739	1,1910	0,2686	0,6043
1	chr1:43616041:T:G	43616041	T	0,4741	G	0,5726	0,4492	0,8959	0,1453	0,6739	1,1910	0,2683	0,6044
1	chr1:43620409:T:C	43620409	T	0,4741	C	0,5726	0,4492	0,8959	0,1453	0,6739	1,1910	0,2683	0,6044
1	chr1:43837594:A:G	43837594	G	0,4629	A	0,3182	0,5727	1,0880	0,1492	0,8122	1,4570	0,2655	0,6063
1	chr1:43549154:A:G	43549154	A	0,2230	G	1,9850	0,1588	1,2980	0,1864	0,9008	1,8710	0,2651	0,6067
7	chr7:114470513:C:T	114470513	C	0,1630	T	0,0254	0,8733	1,0300	0,1874	0,7135	1,4870	0,2648	0,6068
1	chr1:44009451:C:T	44009451	T	0,0967	C	0,2401	0,6241	1,1280	0,2488	0,6928	1,8370	0,2630	0,6081
1	chr1:43671586:C:A	43671586	C	0,4786	A	0,0765	0,7821	0,9605	0,1456	0,7220	1,2780	0,2581	0,6114
4	chr4:111485805:C:T	111485805	T	0,0418	C	0,5202	0,4707	0,7805	0,3497	0,3933	1,5490	0,2567	0,6124
11	chr11:28602326:G:T	28602326	G	0,3888	T	2,3130	0,1283	0,7944	0,1517	0,5900	1,0690	0,2563	0,6126
4	chr4:111287592:T:C	111287592	C	0,0533	T	0,0016	0,9678	0,9880	0,3018	0,5468	1,7850	0,2543	0,614
5	chr5:88397820:G:C	88397820	G	0,4750	C	0,5346	0,4647	1,1140	0,1475	0,8343	1,4880	0,2533	0,6147
4	chr4:111309356:T:A	111309356	A	0,0651	T	2,3270	0,1271	0,6536	0,2838	0,3748	1,1400	0,2526	0,6153
16	chr16:61755912:G:C	61755912	G	0,4757	C	0,2135	0,6440	1,0700	0,1464	0,8031	1,4260	0,2513	0,6162

5	chr5:88386213:G:A	88386213	G	0,4750	A	0,5266	0,4680	1,1130	0,1472	0,8339	1,4850	0,2474	0,6189
5	chr5:104585069:C:A	104585069	A	0,4769	C	0,0069	0,9338	0,9875	0,1509	0,7347	1,3270	0,2470	0,6192
1	chr1:43636043:A:T	43636043	A	0,4752	T	0,6188	0,4315	0,8925	0,1446	0,6723	1,1850	0,2440	0,6213
1	chr1:43636129:A:G	43636129	A	0,4752	G	0,6188	0,4315	0,8925	0,1446	0,6723	1,1850	0,2440	0,6213
1	chr1:43636586:T:C	43636586	T	0,4752	C	0,6188	0,4315	0,8925	0,1446	0,6723	1,1850	0,2440	0,6213
1	chr1:43616545:G:A	43616545	G	0,4723	A	0,5221	0,4699	0,9003	0,1453	0,6771	1,1970	0,2435	0,6217
1	chr1:43629437:A:G	43629437	A	0,4750	G	0,6035	0,4373	0,8933	0,1453	0,6719	1,1870	0,2409	0,6236
1	chr1:43631843:C:G	43631843	C	0,4750	G	0,6035	0,4373	0,8933	0,1453	0,6719	1,1870	0,2409	0,6236
1	chr1:43634192:G:T	43634192	G	0,4750	T	0,6035	0,4373	0,8933	0,1453	0,6719	1,1870	0,2409	0,6236
1	chr1:43634239:T:C	43634239	T	0,4750	C	0,6035	0,4373	0,8933	0,1453	0,6719	1,1870	0,2409	0,6236
4	chr4:111295017:T:A	111295017	A	0,0532	T	0,0001	0,9912	0,9967	0,3017	0,5518	1,8000	0,2392	0,6248
4	chr4:111296367:C:T	111296367	T	0,0532	C	0,0001	0,9912	0,9967	0,3017	0,5518	1,8000	0,2392	0,6248
4	chr4:111306830:A:T	111306830	T	0,0532	A	0,0001	0,9912	0,9967	0,3017	0,5518	1,8000	0,2392	0,6248
16	chr16:61749670:C:T	61749670	C	0,4757	T	0,2004	0,6544	1,0680	0,1470	0,8007	1,4250	0,2378	0,6258
16	chr16:61754062:A:G	61754062	G	0,3544	A	0,0399	0,8417	1,0300	0,1503	0,7676	1,3830	0,2348	0,628
1	chr1:43623961:A:G	43623961	A	0,4750	G	0,5004	0,4793	0,9023	0,1453	0,6786	1,2000	0,2326	0,6296
7	chr7:114469294:A:G	114469294	A	0,3397	G	0,2756	0,5996	0,9214	0,1563	0,6783	1,2520	0,2304	0,6312
1	chr1:43510486:G:A	43510486	A	0,4856	G	0,0973	0,7551	0,9516	0,1588	0,6971	1,2990	0,2268	0,6339
1	chr1:43667345:T:C	43667345	T	0,4796	C	0,0846	0,7712	0,9587	0,1449	0,7216	1,2740	0,2261	0,6345
7	chr7:114464334:T:C	114464334	T	0,4159	C	0,5207	0,4705	1,1120	0,1469	0,8336	1,4830	0,2249	0,6353
20	chr20:21456154:A:G	21456154	G	0,4467	A	0,0269	0,8698	1,0240	0,1468	0,7683	1,3660	0,2240	0,636
20	chr20:21456675:T:C	21456675	C	0,4467	T	0,0269	0,8698	1,0240	0,1468	0,7683	1,3660	0,2240	0,636
1	chr1:43549064:C:T	43549064	C	0,2355	T	0,7110	0,3991	1,1650	0,1823	0,8152	1,6660	0,2140	0,6437
1	chr1:43596124:C:T	43596124	C	0,4647	T	0,1992	0,6554	0,9371	0,1454	0,7048	1,2460	0,2110	0,646
1	chr1:43848594:C:T	43848594	T	0,4510	C	0,2125	0,6448	1,0710	0,1479	0,8013	1,4310	0,2100	0,6467
7	chr7:114456791:A:T	114456791	A	0,4222	T	0,4123	0,5208	1,0990	0,1464	0,8245	1,4640	0,2066	0,6494
4	chr4:111251830:C:A	111251830	A	0,0456	C	1,4610	0,2268	1,5140	0,3410	0,7763	2,9540	0,2008	0,6541
5	chr5:104568525:A:G	104568525	A	0,3149	G	1,7690	0,1835	0,8047	0,1629	0,5848	1,1070	0,2008	0,6541
7	chr7:114451698:G:A	114451698	G	0,4202	A	0,4792	0,4888	1,1060	0,1460	0,8310	1,4730	0,2001	0,6547
1	chr1:43707752:G:A	43707752	A	0,1648	G	0,6818	0,4090	1,1720	0,1911	0,8060	1,7050	0,1937	0,6599
1	chr1:43722794:A:C	43722794	C	0,1648	A	0,6818	0,4090	1,1720	0,1911	0,8060	1,7050	0,1937	0,6599
1	chr1:43723048:A:G	43723048	G	0,1648	A	0,6818	0,4090	1,1720	0,1911	0,8060	1,7050	0,1937	0,6599
5	chr5:88867954:G:A	88867954	A	0,2558	G	2,1370	0,1438	0,7810	0,1699	0,5597	1,0900	0,1937	0,6599
5	chr5:88385177:A:G	88385177	A	0,4760	G	0,4096	0,5222	1,0990	0,1475	0,8232	1,4680	0,1886	0,6641
16	chr16:61781742:A:G	61781742	A	0,4823	G	0,8604	0,3536	1,1450	0,1464	0,8597	1,5260	0,1875	0,665
7	chr7:114554560:T:G	114554560	T	0,2669	G	0,0158	0,8999	1,0210	0,1654	0,7383	1,4120	0,1872	0,6652
5	chr5:88879434:C:G	88879434	C	0,2581	G	0,5586	0,4548	0,8744	0,1803	0,6142	1,2450	0,1865	0,6659
20	chr20:21457201:T:G	21457201	G	0,4457	T	0,0092	0,9236	1,0140	0,1469	0,7604	1,3530	0,1787	0,6725
20	chr20:21463034:C:G	21463034	G	0,4457	C	0,0092	0,9236	1,0140	0,1469	0,7604	1,3530	0,1787	0,6725
7	chr7:114474994:A:G	114474994	A	0,4156	G	0,6139	0,4333	1,1210	0,1463	0,8418	1,4940	0,1783	0,6729
7	chr7:114440281:T:G	114440281	T	0,4151	G	0,1542	0,6946	1,0600	0,1475	0,7936	1,4150	0,1761	0,6748
4	chr4:111314599:T:C	111314599	C	0,0722	T	4,2880	0,0384	0,5700	0,2769	0,3312	0,9808	0,1753	0,6754

1	chr1:43756926:G:A	43756926	A	0,4991	G	0,7846	0,3757	1,1390	0,1467	0,8543	1,5180	0,1745	0,6762
1	chr1:43509093:G:T	43509093	T	0,4507	G	0,1572	0,6917	1,0640	0,1568	0,7826	1,4470	0,1738	0,6768
1	chr1:43510950:G:C	43510950	C	0,4507	G	0,1572	0,6917	1,0640	0,1568	0,7826	1,4470	0,1738	0,6768
1	chr1:43840750:C:T	43840750	T	0,4584	C	0,0553	0,8141	1,0360	0,1482	0,7745	1,3850	0,1725	0,6779
1	chr1:43718252:C:T	43718252	T	0,1627	C	0,8975	0,3434	1,2010	0,1923	0,8239	1,7510	0,1698	0,6803
5	chr5:104659667:A:G	104659667	A	0,2234	G	2,0090	0,1563	0,7835	0,1714	0,5599	1,0960	0,1669	0,6829
5	chr5:88401026:G:A	88401026	G	0,4751	A	0,4418	0,5063	1,1030	0,1473	0,8264	1,4720	0,1661	0,6836
7	chr7:114505470:G:A	114505470	G	0,3438	A	0,2459	0,6200	0,9252	0,1569	0,6803	1,2580	0,1652	0,6844
5	chr5:104734216:A:G	104734216	A	0,1682	G	2,4260	0,1193	0,7433	0,1896	0,5126	1,0780	0,1645	0,685
7	chr7:114508276:C:A	114508276	C	0,2696	A	0,0994	0,7525	0,9492	0,1656	0,6861	1,3130	0,1618	0,6875
1	chr1:43716573:A:G	43716573	G	0,1624	A	0,9107	0,3399	1,2030	0,1924	0,8250	1,7540	0,1608	0,6884
1	chr1:43685962:T:G	43685962	G	0,1624	T	0,8505	0,3564	1,1950	0,1923	0,8200	1,7420	0,1603	0,6889
7	chr7:114571392:A:G	114571392	A	0,3193	G	2,4540	0,1172	1,2690	0,1523	0,9411	1,7100	0,1588	0,6903
20	chr20:21465660:G:T	21465660	T	0,4448	G	0,0137	0,9070	1,0170	0,1469	0,7628	1,3570	0,1559	0,6929
1	chr1:43739423:T:C	43739423	C	0,4991	T	0,5372	0,4636	1,1130	0,1454	0,8367	1,4800	0,1473	0,7011
1	chr1:43609834:G:C	43609834	G	0,4667	C	0,2091	0,6475	0,9355	0,1458	0,7029	1,2450	0,1471	0,7013
1	chr1:43830767:C:G	43830767	G	0,2686	C	0,5035	0,4780	1,1290	0,1712	0,8070	1,5790	0,1424	0,7059
5	chr5:88416702:C:A	88416702	C	0,4771	A	0,3901	0,5322	1,0960	0,1469	0,8219	1,4620	0,1412	0,7071
16	chr16:61746654:A:G	61746654	G	0,3504	A	0,0302	0,8620	1,0270	0,1530	0,7608	1,3860	0,1390	0,7093
16	chr16:61754668:C:T	61754668	T	0,3504	C	0,0302	0,8620	1,0270	0,1530	0,7608	1,3860	0,1390	0,7093
5	chr5:104646025:T:G	104646025	G	0,1685	T	1,9460	0,1631	0,7661	0,1902	0,5278	1,1120	0,1369	0,7114
7	chr7:114589084:G:A	114589084	G	0,3267	A	1,8570	0,1730	1,2320	0,1538	0,9118	1,6660	0,1368	0,7114
1	chr1:43682497:T:C	43682497	C	0,1593	T	1,1490	0,2837	1,2320	0,1935	0,8431	1,8000	0,1367	0,7116
1	chr1:43686518:G:A	43686518	A	0,1593	G	1,1490	0,2837	1,2320	0,1935	0,8431	1,8000	0,1367	0,7116
1	chr1:43687044:G:A	43687044	A	0,1593	G	1,1490	0,2837	1,2320	0,1935	0,8431	1,8000	0,1367	0,7116
1	chr1:43687948:G:C	43687948	C	0,1593	G	1,1490	0,2837	1,2320	0,1935	0,8431	1,8000	0,1367	0,7116
1	chr1:43688808:G:C	43688808	C	0,1593	G	1,1490	0,2837	1,2320	0,1935	0,8431	1,8000	0,1367	0,7116
4	chr4:111500989:G:A	111500989	A	0,0440	G	0,3722	0,5418	0,8124	0,3448	0,4133	1,5970	0,1355	0,7128
1	chr1:43560369:A:G	43560369	G	0,1832	A	2,7690	0,0961	1,3490	0,1812	0,9461	1,9250	0,1339	0,7144
5	chr5:88389683:C:T	88389683	C	0,4769	T	0,3012	0,5832	1,0840	0,1475	0,8122	1,4480	0,1335	0,7149
1	chr1:43575295:T:G	43575295	T	0,4673	G	0,1362	0,7121	0,9477	0,1456	0,7124	1,2610	0,1298	0,7186
1	chr1:43605550:G:A	43605550	G	0,4907	A	1,5380	0,2148	0,8350	0,1453	0,6280	1,1100	0,1271	0,7214
3	chr3:20678505:C:T	20678505	T	0,2914	C	3,4420	0,0636	0,7431	0,1606	0,5424	1,0180	0,1223	0,7266
5	chr5:104742532:A:C	104742532	A	0,1692	C	1,9230	0,1655	0,7631	0,1941	0,5216	1,1160	0,1205	0,7285
5	chr5:104746478:A:C	104746478	A	0,1692	C	1,9230	0,1655	0,7631	0,1941	0,5216	1,1160	0,1205	0,7285
1	chr1:43552933:G:T	43552933	T	0,4824	G	0,0458	0,8305	0,9693	0,1455	0,7287	1,2890	0,1202	0,7288
7	chr7:114384998:A:G	114384998	G	0,1660	A	0,0276	0,8682	1,0320	0,1902	0,7110	1,4990	0,1184	0,7308
1	chr1:43831901:A:C	43831901	C	0,4580	A	0,1928	0,6606	1,0680	0,1493	0,7969	1,4310	0,1152	0,7342
1	chr1:43689712:A:G	43689712	G	0,4963	A	0,3122	0,5763	1,0840	0,1443	0,8170	1,4390	0,1139	0,7357
3	chr3:20683444:C:T	20683444	T	0,2918	C	3,4750	0,0623	0,7421	0,1606	0,5417	1,0170	0,1135	0,7361
1	chr1:43685344:T:G	43685344	G	0,4963	T	0,3044	0,5812	1,0830	0,1441	0,8164	1,4360	0,1116	0,7384
1	chr1:43717869:G:T	43717869	T	0,4982	G	0,3563	0,5506	1,0900	0,1449	0,8208	1,4490	0,1113	0,7387

1	chr1:43610348:G:A	43610348	A	0,4496	G	2,4660	0,1163	1,2550	0,1445	0,9451	1,6650	0,1092	0,7411
3	chr3:20680838:A:C	20680838	C	0,2989	A	3,1760	0,0747	0,7532	0,1596	0,5509	1,0300	0,1089	0,7414
7	chr7:114520816:G:A	114520816	G	0,2686	A	0,0308	0,8607	0,9714	0,1657	0,7020	1,3440	0,1061	0,7446
3	chr3:20680326:A:G	20680326	G	0,2924	A	3,5210	0,0606	0,7407	0,1606	0,5407	1,0150	0,1023	0,7491
3	chr3:20680427:C:T	20680427	T	0,2924	C	3,5210	0,0606	0,7407	0,1606	0,5407	1,0150	0,1023	0,7491
5	chr5:104739429:T:C	104739429	T	0,1696	C	2,0850	0,1488	0,7545	0,1944	0,5154	1,1040	0,1013	0,7502
3	chr3:20682712:G:A	20682712	A	0,2914	G	3,3490	0,0672	0,7456	0,1610	0,5439	1,0220	0,1003	0,7515
5	chr5:88567792:C:T	88567792	C	0,4800	T	4,4190	0,0356	0,7257	0,1526	0,5381	0,9787	0,0984	0,7538
5	chr5:88574430:T:C	88574430	T	0,4800	C	4,4190	0,0356	0,7257	0,1526	0,5381	0,9787	0,0984	0,7538
5	chr5:88577805:C:T	88577805	C	0,4800	T	4,4190	0,0356	0,7257	0,1526	0,5381	0,9787	0,0984	0,7538
12	chr12:89364160:G:A	89364160	G	0,4969	A	1,7670	0,1837	1,2230	0,1517	0,9088	1,6470	0,0977	0,7547
12	chr12:89366967:A:G	89366967	A	0,4969	G	1,7670	0,1837	1,2230	0,1517	0,9088	1,6470	0,0977	0,7547
7	chr7:114544043:G:A	114544043	G	0,2703	A	0,0015	0,9690	1,0060	0,1651	0,7282	1,3910	0,0969	0,7556
7	chr7:114560479:G:A	114560479	G	0,3409	A	1,5560	0,2123	1,2090	0,1526	0,8967	1,6310	0,0952	0,7576
1	chr1:43550344:G:A	43550344	A	0,1564	G	1,9720	0,1603	1,3190	0,1982	0,8943	1,9450	0,0940	0,7591
1	chr1:43554060:C:T	43554060	C	0,1875	T	2,5420	0,1109	1,3330	0,1812	0,9345	1,9020	0,0933	0,7601
1	chr1:43556356:A:G	43556356	A	0,1875	G	2,5420	0,1109	1,3330	0,1812	0,9345	1,9020	0,0933	0,7601
1	chr1:43744582:C:G	43744582	G	0,4962	C	0,6202	0,4310	1,1220	0,1465	0,8422	1,4960	0,0932	0,7601
1	chr1:43745339:A:G	43745339	G	0,4962	A	0,6202	0,4310	1,1220	0,1465	0,8422	1,4960	0,0932	0,7601
1	chr1:43746026:T:C	43746026	C	0,4962	T	0,6202	0,4310	1,1220	0,1465	0,8422	1,4960	0,0932	0,7601
1	chr1:43747271:A:G	43747271	G	0,4962	A	0,6202	0,4310	1,1220	0,1465	0,8422	1,4960	0,0932	0,7601
1	chr1:43747507:C:A	43747507	A	0,4962	C	0,6202	0,4310	1,1220	0,1465	0,8422	1,4960	0,0932	0,7601
1	chr1:43751205:T:C	43751205	C	0,4962	T	0,6202	0,4310	1,1220	0,1465	0,8422	1,4960	0,0932	0,7601
1	chr1:43753604:T:C	43753604	C	0,4962	T	0,6202	0,4310	1,1220	0,1465	0,8422	1,4960	0,0932	0,7601
1	chr1:43763926:G:A	43763926	A	0,4962	G	0,6202	0,4310	1,1220	0,1465	0,8422	1,4960	0,0932	0,7601
1	chr1:43777612:C:T	43777612	T	0,4962	C	0,6202	0,4310	1,1220	0,1465	0,8422	1,4960	0,0932	0,7601
1	chr1:43614741:T:C	43614741	T	0,4972	C	1,4870	0,2226	0,8380	0,1449	0,6308	1,1130	0,0931	0,7603
1	chr1:43577721:G:A	43577721	A	0,1974	G	2,2700	0,1319	1,3080	0,1778	0,9233	1,8540	0,0928	0,7607
1	chr1:43596483:C:T	43596483	T	0,2102	C	3,4620	0,0628	1,3870	0,1767	0,9809	1,9610	0,0916	0,7622
1	chr1:43720773:T:A	43720773	A	0,4972	T	0,3775	0,5389	1,0930	0,1449	0,8230	1,4520	0,0907	0,7633
1	chr1:43610798:G:A	43610798	A	0,4530	G	2,0520	0,1520	1,2310	0,1451	0,9262	1,6360	0,0889	0,7656
4	chr4:111267775:T:C	111267775	C	0,0409	T	1,2470	0,2641	1,4940	0,3580	0,7406	3,0130	0,0872	0,7678
5	chr5:88579561:C:T	88579561	C	0,4809	T	4,1110	0,0426	0,7345	0,1523	0,5450	0,9898	0,0872	0,7678
5	chr5:88584754:A:G	88584754	A	0,4809	G	4,1110	0,0426	0,7345	0,1523	0,5450	0,9898	0,0872	0,7678
5	chr5:88586361:A:G	88586361	A	0,4809	G	4,1110	0,0426	0,7345	0,1523	0,5450	0,9898	0,0872	0,7678
1	chr1:43504626:G:A	43504626	A	0,4766	G	0,0920	0,7617	0,9527	0,1597	0,6967	1,3030	0,0862	0,7691
7	chr7:114564074:T:A	114564074	T	0,1815	A	0,4234	0,5152	1,1270	0,1848	0,7847	1,6200	0,0859	0,7695
12	chr12:89381252:T:G	89381252	T	0,4917	G	1,2960	0,2549	1,1900	0,1526	0,8822	1,6050	0,0844	0,7714
3	chr3:20683524:C:A	20683524	A	0,2933	C	3,6000	0,0578	0,7382	0,1605	0,5389	1,0110	0,0841	0,7718
1	chr1:43585185:T:C	43585185	T	0,4731	C	0,3875	0,5336	0,9135	0,1453	0,6871	1,2140	0,0840	0,772
7	chr7:114416000:G:C	114416000	C	0,3327	G	0,8870	0,3463	0,8623	0,1575	0,6333	1,1740	0,0793	0,7782
7	chr7:114570759:T:C	114570759	T	0,3390	C	1,6570	0,1981	1,2170	0,1528	0,9020	1,6420	0,0780	0,7801

7	chr7:114540007:A:G	114540007	A	0,2687	G	0,0209	0,8851	0,9763	0,1661	0,7050	1,3520	0,0773	0,7809
1	chr1:43612713:T:C	43612713	C	0,4457	T	2,4940	0,1143	1,2580	0,1454	0,9460	1,6720	0,0757	0,7833
3	chr3:20627579:T:G	20627579	G	0,3917	T	0,1983	0,6561	0,9350	0,1507	0,6959	1,2560	0,0711	0,7898
3	chr3:20685150:G:A	20685150	A	0,2414	G	3,5910	0,0581	0,7054	0,1851	0,4908	1,0140	0,0692	0,7925
3	chr3:20685154:C:T	20685154	T	0,2414	C	3,5910	0,0581	0,7054	0,1851	0,4908	1,0140	0,0692	0,7925
5	chr5:88865600:T:C	88865600	C	0,2534	T	1,4160	0,2341	0,8172	0,1701	0,5855	1,1410	0,0691	0,7926
1	chr1:43778195:T:C	43778195	C	0,4953	T	0,5293	0,4669	1,1130	0,1464	0,8350	1,4820	0,0679	0,7944
1	chr1:43781562:C:G	43781562	G	0,4953	C	0,5293	0,4669	1,1130	0,1464	0,8350	1,4820	0,0679	0,7944
5	chr5:88864537:C:T	88864537	T	0,2500	C	1,1840	0,2766	0,8324	0,1690	0,5977	1,1590	0,0672	0,7954
5	chr5:88861735:T:C	88861735	C	0,2620	T	0,7092	0,3997	0,8717	0,1634	0,6328	1,2010	0,0625	0,8026
1	chr1:43558813:A:G	43558813	A	0,4853	G	0,3157	0,5742	0,9220	0,1444	0,6947	1,2240	0,0622	0,803
5	chr5:88569970:A:G	88569970	A	0,4810	G	4,8200	0,0281	0,7153	0,1527	0,5303	0,9649	0,0604	0,8058
12	chr12:89368722:C:G	89368722	C	0,4928	G	1,4150	0,2342	1,1990	0,1525	0,8892	1,6160	0,0598	0,8068
12	chr12:89369752:C:T	89369752	C	0,4928	T	1,4150	0,2342	1,1990	0,1525	0,8892	1,6160	0,0598	0,8068
1	chr1:43560382:T:G	43560382	T	0,4972	G	0,9390	0,3325	0,8688	0,1451	0,6538	1,1540	0,0585	0,8088
1	chr1:43560509:A:C	43560509	A	0,4972	C	0,9390	0,3325	0,8688	0,1451	0,6538	1,1540	0,0585	0,8088
5	chr5:88601453:A:C	88601453	A	0,4641	C	2,6920	0,1009	0,7775	0,1533	0,5757	1,0500	0,0577	0,8101
1	chr1:43563242:G:A	43563242	A	0,1853	G	2,5380	0,1111	1,3320	0,1807	0,9348	1,8980	0,0571	0,8111
14	chr14:98239063:A:G	98239063	G	0,1419	A	0,0027	0,9585	1,0110	0,2106	0,6690	1,5280	0,0562	0,8126
1	chr1:43613740:T:C	43613740	C	0,4466	T	2,6700	0,1022	1,2680	0,1453	0,9536	1,6860	0,0551	0,8144
1	chr1:43726088:C:T	43726088	T	0,4926	C	0,4028	0,5256	1,0960	0,1449	0,8253	1,4570	0,0539	0,8165
1	chr1:43731274:C:T	43731274	T	0,4926	C	0,4028	0,5256	1,0960	0,1449	0,8253	1,4570	0,0539	0,8165
7	chr7:114451789:C:A	114451789	C	0,3438	A	0,8375	0,3601	0,8673	0,1557	0,6391	1,1770	0,0535	0,8171
11	chr11:28578333:T:A	28578333	T	0,3770	A	2,0140	0,1559	0,8048	0,1532	0,5961	1,0870	0,0494	0,8241
1	chr1:43692458:C:G	43692458	G	0,1602	C	0,6962	0,4041	1,1750	0,1922	0,8059	1,7120	0,0476	0,8273
1	chr1:43576868:C:G	43576868	C	0,4748	G	0,2753	0,5998	0,9265	0,1455	0,6966	1,2320	0,0472	0,828
1	chr1:43606749:A:G	43606749	A	0,4697	G	0,2178	0,6408	0,9346	0,1448	0,7037	1,2410	0,0470	0,8283
7	chr7:114350297:C:T	114350297	T	0,1235	C	0,4019	0,5261	1,1480	0,2182	0,7484	1,7600	0,0468	0,8288
5	chr5:88461101:C:T	88461101	C	0,4825	T	0,8692	0,3512	1,1440	0,1442	0,8624	1,5180	0,0462	0,8298
1	chr1:43728545:T:G	43728545	G	0,4944	T	0,3664	0,5449	1,0920	0,1451	0,8216	1,4510	0,0447	0,8326
1	chr1:43737320:A:G	43737320	G	0,4944	A	0,3622	0,5473	1,0920	0,1454	0,8209	1,4510	0,0441	0,8337
1	chr1:43783814:A:G	43783814	A	0,4830	G	0,2520	0,6156	0,9286	0,1474	0,6956	1,2400	0,0438	0,8343
5	chr5:88417014:A:G	88417014	A	0,4778	G	0,2221	0,6374	1,0720	0,1473	0,8032	1,4310	0,0435	0,8347
5	chr5:88455935:G:A	88455935	G	0,4894	A	0,6613	0,4161	1,1280	0,1476	0,8444	1,5060	0,0435	0,8348
5	chr5:88458988:C:T	88458988	C	0,4894	T	0,6613	0,4161	1,1280	0,1476	0,8444	1,5060	0,0435	0,8348
1	chr1:43728863:A:G	43728863	G	0,4945	A	0,3623	0,5472	1,0910	0,1450	0,8214	1,4500	0,0435	0,8349
1	chr1:43729682:T:C	43729682	C	0,4945	T	0,3623	0,5472	1,0910	0,1450	0,8214	1,4500	0,0435	0,8349
14	chr14:98255846:A:C	98255846	C	0,1441	A	0,0309	0,8604	1,0380	0,2114	0,6859	1,5710	0,0397	0,8421
7	chr7:114419101:C:T	114419101	T	0,3308	C	1,0130	0,3142	0,8538	0,1571	0,6276	1,1620	0,0395	0,8425
7	chr7:114508188:A:G	114508188	A	0,2657	G	0,0970	0,7554	0,9495	0,1666	0,6850	1,3160	0,0391	0,8432
1	chr1:43592279:T:C	43592279	T	0,4814	C	0,6117	0,4342	0,8924	0,1454	0,6711	1,1870	0,0378	0,8458
1	chr1:43634413:G:A	43634413	A	0,4421	G	3,7240	0,0536	1,3230	0,1450	0,9954	1,7580	0,0374	0,8466

5	chr5:88456878:A:G	88456878	A	0,4952	G	0,2991	0,5844	1,0840	0,1477	0,8117	1,4480	0,0358	0,85
5	chr5:88461004:C:T	88461004	C	0,4952	T	0,2991	0,5844	1,0840	0,1477	0,8117	1,4480	0,0358	0,85
1	chr1:43565822:A:G	43565822	A	0,4925	G	0,5561	0,4558	0,8972	0,1453	0,6748	1,1930	0,0344	0,8529
7	chr7:114608796:A:G	114608796	G	0,1492	A	1,5000	0,2207	1,2780	0,2000	0,8637	1,8920	0,0322	0,8575
1	chr1:43568097:C:A	43568097	C	0,4916	A	0,4105	0,5217	0,9109	0,1456	0,6848	1,2120	0,0308	0,8607
3	chr3:20703004:G:A	20703004	A	0,1940	G	1,3660	0,2425	0,7894	0,2019	0,5315	1,1730	0,0307	0,8609
5	chr5:88574395:G:A	88574395	G	0,4605	A	3,0230	0,0821	0,7663	0,1531	0,5676	1,0340	0,0296	0,8633
7	chr7:114430980:C:T	114430980	C	0,3532	T	0,1357	0,7126	0,9446	0,1547	0,6975	1,2790	0,0291	0,8646
1	chr1:43560418:C:T	43560418	T	0,4935	C	0,6531	0,4190	0,8889	0,1456	0,6682	1,1820	0,0287	0,8656
3	chr3:20695381:T:C	20695381	C	0,1929	T	1,6450	0,1996	0,7731	0,2002	0,5222	1,1450	0,0286	0,8658
1	chr1:43566122:G:C	43566122	C	0,1987	G	1,0060	0,3158	1,1930	0,1765	0,8443	1,6870	0,0284	0,8661
3	chr3:20700799:C:T	20700799	T	0,1921	C	1,2760	0,2587	0,7944	0,2034	0,5332	1,1830	0,0276	0,868
1	chr1:43656802:T:G	43656802	G	0,1774	T	2,5840	0,1079	1,3410	0,1834	0,9365	1,9220	0,0273	0,8688
7	chr7:114571857:G:A	114571857	G	0,1806	A	0,9014	0,3424	1,1920	0,1854	0,8289	1,7140	0,0272	0,8691
1	chr1:43540668:A:G	43540668	G	0,4769	A	0,0228	0,8799	0,9771	0,1532	0,7237	1,3190	0,0267	0,8703
5	chr5:88862329:G:C	88862329	C	0,2277	G	0,6821	0,4089	0,8662	0,1742	0,6157	1,2190	0,0265	0,8708
1	chr1:43721141:A:T	43721141	T	0,1590	A	0,8074	0,3689	1,1890	0,1925	0,8155	1,7350	0,0264	0,8709
1	chr1:43577434:T:C	43577434	T	0,4767	C	0,3201	0,5715	0,9209	0,1455	0,6924	1,2250	0,0262	0,8715
1	chr1:43557944:G:A	43557944	A	0,4823	G	0,1960	0,6580	0,9376	0,1455	0,7049	1,2470	0,0260	0,8718
7	chr7:114522685:G:A	114522685	G	0,2667	A	0,0865	0,7687	0,9523	0,1663	0,6874	1,3190	0,0255	0,8732
1	chr1:43574179:A:G	43574179	G	0,1940	A	1,4740	0,2247	1,2410	0,1780	0,8753	1,7590	0,0251	0,874
7	chr7:114518899:G:A	114518899	G	0,1679	A	0,4318	0,5111	1,1330	0,1897	0,7809	1,6430	0,0244	0,876
1	chr1:43674404:A:C	43674404	C	0,1685	A	1,5020	0,2204	1,2580	0,1880	0,8705	1,8190	0,0235	0,8781
1	chr1:43729733:C:T	43729733	T	0,4898	C	0,4867	0,4854	1,1070	0,1451	0,8327	1,4710	0,0231	0,8792
7	chr7:114443495:C:G	114443495	C	0,3438	G	0,8539	0,3555	0,8658	0,1560	0,6377	1,1750	0,0225	0,8809
1	chr1:43592796:C:T	43592796	T	0,1882	C	2,7760	0,0957	1,3500	0,1799	0,9487	1,9200	0,0220	0,8821
1	chr1:43593571:G:A	43593571	A	0,1882	G	2,7760	0,0957	1,3500	0,1799	0,9487	1,9200	0,0220	0,8821
1	chr1:43594812:G:A	43594812	A	0,1882	G	2,7760	0,0957	1,3500	0,1799	0,9487	1,9200	0,0220	0,8821
1	chr1:43600753:C:A	43600753	A	0,1895	C	2,6900	0,1010	1,3430	0,1798	0,9443	1,9110	0,0220	0,8822
1	chr1:43569422:A:G	43569422	G	0,4703	A	0,0377	0,8462	0,9722	0,1453	0,7312	1,2930	0,0207	0,8857
1	chr1:43556632:A:C	43556632	C	0,4944	A	0,3878	0,5335	0,9134	0,1453	0,6870	1,2140	0,0203	0,8868
1	chr1:43853701:G:A	43853701	G	0,4945	A	0,8100	0,3681	0,8781	0,1444	0,6617	1,1650	0,0202	0,887
5	chr5:88467651:C:A	88467651	C	0,4903	A	0,5215	0,4702	1,1130	0,1476	0,8331	1,4860	0,0198	0,888
5	chr5:88473876:C:T	88473876	C	0,4903	T	0,5215	0,4702	1,1130	0,1476	0,8331	1,4860	0,0198	0,888
5	chr5:88477912:G:T	88477912	G	0,4903	T	0,5215	0,4702	1,1130	0,1476	0,8331	1,4860	0,0198	0,888
1	chr1:43584356:A:C	43584356	A	0,4833	C	0,6776	0,4104	0,8871	0,1454	0,6671	1,1800	0,0193	0,8894
1	chr1:43631859:T:C	43631859	C	0,4466	T	3,1250	0,0771	1,2930	0,1452	0,9724	1,7180	0,0189	0,8907
1	chr1:43658317:C:T	43658317	T	0,1807	C	2,4470	0,1178	1,3280	0,1820	0,9297	1,8980	0,0179	0,8936
14	chr14:98259646:C:T	98259646	T	0,1429	C	0,0109	0,9169	1,0220	0,2121	0,6747	1,5490	0,0175	0,8949
3	chr3:20706828:T:G	20706828	G	0,1947	T	1,5370	0,2150	0,7791	0,2010	0,5254	1,1550	0,0170	0,8962
7	chr7:114407487:G:C	114407487	C	0,3385	G	0,9818	0,3217	0,8555	0,1576	0,6282	1,1650	0,0159	0,8995
1	chr1:43547252:C:T	43547252	T	0,4759	C	0,3671	0,5446	0,9115	0,1529	0,6755	1,2300	0,0154	0,9014

1	chr1:43560985:G:C	43560985	C	0,4963	G	1,2250	0,2684	1,1740	0,1451	0,8836	1,5600	0,0150	0,9026
5	chr5:88467699:G:A	88467699	G	0,4962	A	0,2072	0,6490	1,0700	0,1477	0,8007	1,4290	0,0146	0,9038
5	chr5:88469954:T:C	88469954	T	0,4962	C	0,2072	0,6490	1,0700	0,1477	0,8007	1,4290	0,0146	0,9038
5	chr5:88475276:A:C	88475276	A	0,4962	C	0,2072	0,6490	1,0700	0,1477	0,8007	1,4290	0,0146	0,9038
5	chr5:88477838:A:G	88477838	A	0,4962	G	0,2072	0,6490	1,0700	0,1477	0,8007	1,4290	0,0146	0,9038
5	chr5:88445076:T:C	88445076	T	0,4923	C	0,4742	0,4911	1,1070	0,1479	0,8287	1,4800	0,0137	0,9068
14	chr14:98224586:T:A	98224586	A	0,1340	T	0,0150	0,9026	1,0270	0,2162	0,6721	1,5690	0,0136	0,907
1	chr1:43584276:C:T	43584276	T	0,1876	C	2,8440	0,0917	1,3550	0,1800	0,9520	1,9280	0,0131	0,9089
1	chr1:43585903:A:G	43585903	G	0,1876	A	2,8440	0,0917	1,3550	0,1800	0,9520	1,9280	0,0131	0,9089
1	chr1:43586163:A:C	43586163	C	0,1876	A	2,8440	0,0917	1,3550	0,1800	0,9520	1,9280	0,0131	0,9089
7	chr7:114403104:A:G	114403104	G	0,3359	A	0,8295	0,3624	0,8662	0,1577	0,6358	1,1800	0,0125	0,9111
1	chr1:43584333:G:A	43584333	G	0,4842	A	0,7118	0,3989	0,8845	0,1454	0,6651	1,1760	0,0124	0,9113
1	chr1:43586706:T:G	43586706	T	0,4842	G	0,7118	0,3989	0,8845	0,1454	0,6651	1,1760	0,0124	0,9113
1	chr1:43605020:A:G	43605020	G	0,1889	A	3,0740	0,0795	1,3710	0,1799	0,9635	1,9500	0,0116	0,9144
1	chr1:43537286:G:A	43537286	A	0,4851	G	0,0080	0,9288	0,9863	0,1541	0,7292	1,3340	0,0114	0,9151
1	chr1:43599477:C:T	43599477	T	0,1910	C	2,2690	0,1320	1,3100	0,1791	0,9221	1,8610	0,0113	0,9155
1	chr1:43652735:G:A	43652735	A	0,1789	G	2,9190	0,0875	1,3650	0,1828	0,9542	1,9540	0,0107	0,9177
7	chr7:114436339:C:G	114436339	C	0,3391	G	1,1180	0,2903	0,8474	0,1566	0,6234	1,1520	0,0098	0,9212
1	chr1:43655886:C:G	43655886	G	0,1817	C	2,3610	0,1244	1,3220	0,1818	0,9253	1,8870	0,0075	0,9308
7	chr7:114578527:C:T	114578527	C	0,1799	T	0,9340	0,3338	1,1960	0,1857	0,8314	1,7220	0,0073	0,9321
7	chr7:114583668:C:A	114583668	C	0,1799	A	0,9340	0,3338	1,1960	0,1857	0,8314	1,7220	0,0073	0,9321
7	chr7:114584108:A:G	114584108	A	0,1799	G	0,9340	0,3338	1,1960	0,1857	0,8314	1,7220	0,0073	0,9321
1	chr1:43641757:C:T	43641757	T	0,4696	C	1,0380	0,3083	1,1590	0,1447	0,8727	1,5390	0,0064	0,9363
1	chr1:43644078:A:G	43644078	G	0,4695	A	1,0430	0,3072	1,1590	0,1447	0,8730	1,5400	0,0059	0,9389
1	chr1:43565986:C:T	43565986	T	0,4721	C	0,0252	0,8740	0,9772	0,1453	0,7350	1,2990	0,0055	0,9408
7	chr7:114406314:C:G	114406314	G	0,3349	C	1,0960	0,2952	0,8474	0,1581	0,6216	1,1550	0,0054	0,9416
1	chr1:43596488:C:T	43596488	T	0,1891	C	2,4110	0,1205	1,3210	0,1793	0,9296	1,8780	0,0054	0,9417
5	chr5:104482711:A:G	104482711	G	0,3180	A	1,7240	0,1892	0,8155	0,1554	0,6013	1,1060	0,0038	0,9507
1	chr1:43574039:T:C	43574039	T	0,4830	C	0,6206	0,4308	0,8911	0,1462	0,6691	1,1870	0,0034	0,9532
1	chr1:43570414:G:A	43570414	A	0,1963	G	1,7710	0,1833	1,2670	0,1779	0,8942	1,7960	0,0034	0,9533
1	chr1:43569801:G:A	43569801	A	0,2013	G	1,0560	0,3042	1,1980	0,1761	0,8485	1,6920	0,0034	0,9538
1	chr1:43853335:A:G	43853335	A	0,4972	G	0,6366	0,4249	0,8903	0,1456	0,6692	1,1840	0,0026	0,9592
1	chr1:43579794:A:G	43579794	A	0,4888	G	0,6520	0,4194	0,8890	0,1456	0,6682	1,1830	0,0021	0,9631
1	chr1:43632835:G:A	43632835	A	0,1792	G	3,3470	0,0673	1,3980	0,1833	0,9759	2,0020	0,0019	0,9651
7	chr7:114429481:C:G	114429481	C	0,3407	G	1,0850	0,2975	0,8494	0,1566	0,6249	1,1550	0,0018	0,9666
1	chr1:43631767:G:T	43631767	T	0,1777	G	3,5300	0,0603	1,4110	0,1836	0,9844	2,0220	0,0016	0,9682
1	chr1:43586107:G:A	43586107	A	0,1885	G	2,4740	0,1158	1,3260	0,1795	0,9327	1,8850	0,0015	0,9686
1	chr1:43579857:G:A	43579857	A	0,1954	G	1,4630	0,2264	1,2390	0,1774	0,8753	1,7550	0,0007	0,9792
1	chr1:43583485:G:A	43583485	A	0,1954	G	1,4630	0,2264	1,2390	0,1774	0,8753	1,7550	0,0007	0,9792
1	chr1:43585959:G:A	43585959	A	0,1954	G	1,4630	0,2264	1,2390	0,1774	0,8753	1,7550	0,0007	0,9792
1	chr1:43586100:T:C	43586100	C	0,1954	T	1,4630	0,2264	1,2390	0,1774	0,8753	1,7550	0,0007	0,9792
1	chr1:43554723:T:C	43554723	C	0,4907	T	0,3005	0,5836	0,9235	0,1450	0,6950	1,2270	0,0006	0,98

1	chr1:43556863:C:T	43556863	T	0,4907	C	0,3005	0,5836	0,9235	0,1450	0,6950	1,2270	0,0006	0,98
1	chr1:43555785:C:G	43555785	C	0,4935	G	0,3661	0,5451	0,9157	0,1455	0,6885	1,2180	0,0005	0,9825
4	chr4:111546456:G:A	111546456	A	0,1202	G	1,9570	0,1618	1,3980	0,2397	0,8736	2,2360	0,0004	0,9832
7	chr7:114647061:T:A	114647061	A	0,1503	T	0,5760	0,4479	1,1660	0,2024	0,7843	1,7340	0,0004	0,9844
11	chr11:28569621:A:T	28569621	A	0,3740	T	2,0560	0,1516	0,8019	0,1539	0,5930	1,0840	0,0003	0,9857
11	chr11:28570040:C:T	28570040	C	0,3740	T	2,0560	0,1516	0,8019	0,1539	0,5930	1,0840	0,0003	0,9857
5	chr5:88851954:G:A	88851954	A	0,2338	G	0,6923	0,4054	0,8659	0,1730	0,6168	1,2150	0,0003	0,9866
5	chr5:88852224:C:T	88852224	T	0,2338	C	0,6923	0,4054	0,8659	0,1730	0,6168	1,2150	0,0003	0,9866
5	chr5:88853474:A:G	88853474	G	0,2338	A	0,6923	0,4054	0,8659	0,1730	0,6168	1,2150	0,0003	0,9866
5	chr5:88853509:G:A	88853509	A	0,2338	G	0,6923	0,4054	0,8659	0,1730	0,6168	1,2150	0,0003	0,9866
5	chr5:88854472:G:A	88854472	A	0,2338	G	0,6923	0,4054	0,8659	0,1730	0,6168	1,2150	0,0003	0,9866
5	chr5:88855175:T:A	88855175	A	0,2338	T	0,6923	0,4054	0,8659	0,1730	0,6168	1,2150	0,0003	0,9866
5	chr5:88856385:T:C	88856385	C	0,2338	T	0,6923	0,4054	0,8659	0,1730	0,6168	1,2150	0,0003	0,9866
5	chr5:88856757:C:A	88856757	A	0,2338	C	0,6923	0,4054	0,8659	0,1730	0,6168	1,2150	0,0003	0,9866
5	chr5:88858057:C:T	88858057	T	0,2338	C	0,6923	0,4054	0,8659	0,1730	0,6168	1,2150	0,0003	0,9866
14	chr14:98244157:G:A	98244157	A	0,1441	G	0,0615	0,8042	1,0530	0,2101	0,6980	1,5900	0,0003	0,9869
14	chr14:98253981:T:A	98253981	A	0,1441	T	0,0615	0,8042	1,0530	0,2101	0,6980	1,5900	0,0003	0,9869
7	chr7:114627941:T:C	114627941	T	0,3464	C	1,6150	0,2038	1,2200	0,1566	0,8978	1,6590	0,0002	0,9886
7	chr7:114605694:T:A	114605694	T	0,3475	A	1,6050	0,2053	1,2170	0,1552	0,8980	1,6500	0,0002	0,989
1	chr1:43621160:C:T	43621160	T	0,4679	C	1,7830	0,1818	1,2130	0,1444	0,9138	1,6090	0,0001	0,9909
3	chr3:71450250:C:T	71450250	T	0,1886	C	1,8990	0,1682	0,7762	0,1840	0,5413	1,1130	0,0001	0,992
3	chr3:20696597:A:G	20696597	G	0,1935	A	1,3700	0,2418	0,7928	0,1984	0,5374	1,1700	0,0865	0,9926
3	chr3:20698841:C:G	20698841	G	0,1935	C	1,3700	0,2418	0,7928	0,1984	0,5374	1,1700	0,0865	0,9926
5	chr5:88468801:G:A	88468801	G	0,4923	A	0,2925	0,5886	1,0830	0,1479	0,8108	1,4480	0,0500	0,9944
1	chr1:43563291:G:A	43563291	A	0,4740	G	0,0151	0,9021	0,9823	0,1453	0,7389	1,3060	0,0264	0,9959
1	chr1:43617344:G:A	43617344	A	0,4731	G	1,1780	0,2777	1,1710	0,1451	0,8809	1,5560	0,0016	0,999

Supplementary Table 4.

CHR	SNP	RSID	BP	A1	TEST	NMISS	OR	STAT	P
1	chr1:43596483:C:T	rs673253	43596483	T	ADD	520	1,687	3,168	0,001535
1	chr1:43541977:T:C	rs2842189	43541977	T	ADD	442	1,752	3,126	0,001770
1	chr1:43605020:A:G	rs17371903	43605020	G	ADD	539	1,660	3,075	0,002102
1	chr1:43631767:G:T	rs12405972	43631767	T	ADD	542	1,661	3,04	0,002366
1	chr1:43632835:G:A	rs61769649	43632835	A	ADD	543	1,652	3,012	0,002596
1	chr1:43554060:C:T	rs2842185	43554060	C	ADD	527	1,645	2,97	0,002975
1	chr1:43556356:A:G	rs2842178	43556356	A	ADD	527	1,645	2,97	0,002975
1	chr1:43560369:A:G	rs11210869	43560369	G	ADD	534	1,642	2,969	0,002988
1	chr1:43563242:G:A	rs61769611	43563242	A	ADD	536	1,631	2,932	0,003364
1	chr1:43600753:C:A	rs596522	43600753	A	ADD	540	1,617	2,922	0,003479
1	chr1:43652735:G:A	rs61768371	43652735	A	ADD	544	1,624	2,914	0,003565
1	chr1:43584276:C:T	rs3791136	43584276	T	ADD	540	1,615	2,913	0,003585
1	chr1:43585903:A:G	rs6698389	43585903	G	ADD	540	1,615	2,913	0,003585
1	chr1:43586163:A:C	rs501299	43586163	C	ADD	540	1,615	2,913	0,003585
1	chr1:43592796:C:T	rs605709	43592796	T	ADD	541	1,614	2,909	0,003626
1	chr1:43593571:G:A	rs72673082	43593571	A	ADD	541	1,614	2,909	0,003626
1	chr1:43594812:G:A	rs583040	43594812	A	ADD	541	1,614	2,909	0,003626
1	chr1:43577721:G:A	rs552638	43577721	A	ADD	536	1,593	2,846	0,004424
1	chr1:43599477:C:T	rs56175694	43599477	T	ADD	541	1,591	2,831	0,004634
1	chr1:43570414:G:A	rs1887402	43570414	A	ADD	534	1,590	2,828	0,004691
1	chr1:43586107:G:A	rs3791140	43586107	A	ADD	540	1,591	2,827	0,004693
1	chr1:43596488:C:T	rs673242	43596488	T	ADD	541	1,590	2,824	0,004747
1	chr1:43658317:C:T	rs61768372	43658317	T	ADD	544	1,595	2,816	0,004864
1	chr1:43655886:C:G	rs2274465	43655886	G	ADD	544	1,595	2,813	0,004904
1	chr1:43548609:T:C	rs2842188	43548609	T	ADD	444	1,644	2,811	0,004933
1	chr1:43656802:T:G	rs11589584	43656802	G	ADD	543	1,594	2,785	0,005353
1	chr1:43549278:T:C	rs2842187	43549278	T	ADD	443	1,632	2,769	0,005619
1	chr1:43604361:A:G	rs530373	43604361	G	ADD	487	1,484	2,759	0,005796
1	chr1:43574179:A:G	rs2842171	43574179	G	ADD	535	1,572	2,738	0,006176
1	chr1:43543362:C:T	rs2782640	43543362	C	ADD	446	1,611	2,698	0,006968
1	chr1:43549154:A:G	rs2819335	43549154	A	ADD	444	1,605	2,684	0,007272
7	chr7:114571392:A:G	rs10269986	114571392	A	ADD	544	1,479	2,671	0,007571
7	chr7:114608796:A:G	rs12705966	114608796	G	ADD	525	1,671	2,663	0,007751
1	chr1:43569801:G:A	rs943513	43569801	A	ADD	533	1,542	2,653	0,007969
1	chr1:43579857:G:A	rs1808730	43579857	A	ADD	539	1,542	2,648	0,008109
1	chr1:43583485:G:A	rs3791134	43583485	A	ADD	539	1,542	2,648	0,008109
1	chr1:43585959:G:A	rs61769642	43585959	A	ADD	539	1,542	2,648	0,008109
1	chr1:43586100:T:C	rs3791139	43586100	C	ADD	539	1,542	2,648	0,008109
7	chr7:114560479:G:A	rs4355713	114560479	G	ADD	527	1,457	2,598	0,009379
1	chr1:43566122:G:C	rs11586016	43566122	C	ADD	535	1,531	2,588	0,009659
1	chr1:43550344:G:A	rs11580258	43550344	A	ADD	504	1,612	2,581	0,009846
7	chr7:114570759:T:C	rs17137124	114570759	T	ADD	527	1,454	2,58	0,009873
1	chr1:43550138:T:C	rs2819336	43550138	T	ADD	441	1,589	2,563	0,01037
7	chr7:114627941:T:C	rs11983431	114627941	T	ADD	497	1,457	2,512	0,012
7	chr7:114589084:G:A	rs1229758	114589084	G	ADD	527	1,447	2,483	0,01301
7	chr7:114605694:T:A	rs10230558	114605694	T	ADD	504	1,437	2,459	0,01395
1	chr1:43546066:G:A	rs951740	43546066	G	ADD	447	1,521	2,404	0,01621
7	chr7:114571857:G:A	rs2189012	114571857	G	ADD	514	1,528	2,385	0,01708
1	chr1:43548902:A:T	rs2819333	43548902	A	ADD	446	1,511	2,365	0,01805

7	chr7:114578527:C:T	rs1229762	114578527	C	ADD	516	1,529	2,363	0,01814
7	chr7:114583668:C:A	rs1229761	114583668	C	ADD	516	1,529	2,363	0,01814
7	chr7:114584108:A:G	rs1229760	114584108	A	ADD	516	1,529	2,363	0,01814
1	chr1:43674404:A:C	rs10789442	43674404	C	ADD	539	1,493	2,332	0,01969
1	chr1:43634413:G:A	rs11210892	43634413	A	ADD	543	1,369	2,31	0,0209
1	chr1:43549064:C:T	rs2819334	43549064	C	ADD	448	1,487	2,279	0,02267
1	chr1:43605550:G:A	rs631248	43605550	G	ADD	536	0,746	-2,193	0,02833
1	chr1:43631859:T:C	rs2970610	43631859	C	ADD	542	1,345	2,176	0,02953
7	chr7:114564074:T:A	rs7797335	114564074	T	ADD	517	1,465	2,157	0,031
7	chr7:114647061:T:A	rs2045292	114647061	A	ADD	508	1,522	2,153	0,03136
1	chr1:43682497:T:C	rs4660257	43682497	C	ADD	542	1,456	2,141	0,03226
1	chr1:43686518:G:A	rs1971342	43686518	A	ADD	542	1,456	2,141	0,03226
1	chr1:43687044:G:A	rs11805774	43687044	A	ADD	542	1,456	2,141	0,03226
1	chr1:43687948:G:C	rs17401357	43687948	C	ADD	542	1,456	2,141	0,03226
1	chr1:43688808:G:C	rs3791035	43688808	C	ADD	542	1,456	2,141	0,03226
7	chr7:114518899:G:A	rs9969232	114518899	G	ADD	520	1,464	2,092	0,03647
1	chr1:43830767:C:G	rs3011216	43830767	G	ADD	470	1,376	2,06	0,03943
1	chr1:43614741:T:C	rs588657	43614741	T	ADD	541	0,762	-2,056	0,03978
1	chr1:43685962:T:G	rs61768374	43685962	G	ADD	541	1,432	2,052	0,04021
1	chr1:43610348:G:A	rs11210887	43610348	A	ADD	545	1,319	2,047	0,0407
1	chr1:43613740:T:C	rs603542	43613740	C	ADD	542	1,318	2,031	0,04222
1	chr1:43612713:T:C	rs499257	43612713	C	ADD	542	1,308	1,971	0,04872
1	chr1:43574039:T:C	rs2819340	43574039	T	ADD	528	0,765	-1,956	0,05042
1	chr1:43560985:G:C	rs12076635	43560985	C	ADD	534	1,292	1,929	0,05368
1	chr1:43692458:C:G	rs2270972	43692458	G	ADD	542	1,400	1,926	0,05415
1	chr1:43610798:G:A	rs549845	43610798	A	ADD	542	1,295	1,911	0,05603
1	chr1:43721141:A:T	rs11810109	43721141	T	ADD	543	1,392	1,892	0,05854
4	chr4:111251830:C:A	rs11729045	111251830	A	ADD	525	1,819	1,877	0,06047
1	chr1:43560382:T:G	rs10789435	43560382	T	ADD	535	0,781	-1,85	0,06424
1	chr1:43560509:A:C	rs68191270	43560509	A	ADD	535	0,781	-1,85	0,06424
1	chr1:43682946:A:G	rs4660740	43682946	G	ADD	527	1,445	1,837	0,06616
7	chr7:114443495:C:G	rs10228494	114443495	C	ADD	524	0,762	-1,815	0,06949
1	chr1:43756926:G:A	rs11210913	43756926	A	ADD	530	1,279	1,804	0,0712
1	chr1:43579794:A:G	rs2004899	43579794	A	ADD	534	0,784	-1,802	0,07149
4	chr4:111267775:T:C	rs79861884	111267775	C	ADD	525	1,827	1,799	0,07201
16	chr16:61781742:A:G	rs7196175	61781742	A	ADD	535	1,286	1,799	0,07205
7	chr7:114384998:A:G	rs12537376	114384998	G	ADD	535	1,387	1,792	0,07308
1	chr1:43621160:C:T	rs1143702	43621160	T	ADD	544	1,283	1,788	0,07377
1	chr1:43584333:G:A	rs3791137	43584333	G	ADD	537	0,787	-1,776	0,07565
1	chr1:43586706:T:G	rs10890261	43586706	T	ADD	537	0,787	-1,776	0,07565
1	chr1:43716573:A:G	rs17531412	43716573	G	ADD	541	1,361	1,771	0,07657
7	chr7:114436339:C:G	rs10280045	114436339	C	ADD	521	0,767	-1,769	0,07691
1	chr1:43584356:A:C	rs3791138	43584356	A	ADD	537	0,788	-1,769	0,07692
1	chr1:43718252:C:T	rs112361411	43718252	T	ADD	540	1,360	1,768	0,07708
1	chr1:43637827:A:G	rs2970606	43637827	A	ADD	543	0,789	-1,766	0,07747
1	chr1:43638043:G:A	rs564318	43638043	G	ADD	543	0,789	-1,766	0,07747
1	chr1:43639333:C:T	rs486883	43639333	C	ADD	543	0,789	-1,766	0,07747
1	chr1:43640277:T:C	rs649504	43640277	T	ADD	543	0,789	-1,766	0,07747
1	chr1:43640595:C:T	rs574736	43640595	C	ADD	543	0,789	-1,766	0,07747
1	chr1:43640845:C:A	rs636547	43640845	C	ADD	543	0,789	-1,766	0,07747
1	chr1:43641315:G:T	rs581005	43641315	G	ADD	543	0,789	-1,766	0,07747
1	chr1:43642285:C:T	rs694197	43642285	C	ADD	543	0,789	-1,766	0,07747

1	chr1:43642448:T:A	rs483679	43642448	T	ADD	543	0,789	-1,766	0,07747
1	chr1:43643528:A:G	rs594293	43643528	A	ADD	543	0,789	-1,766	0,07747
1	chr1:43644545:A:C	rs609171	43644545	A	ADD	543	0,789	-1,766	0,07747
1	chr1:43644818:A:G	rs620851	43644818	A	ADD	543	0,789	-1,766	0,07747
1	chr1:43645516:A:G	rs623965	43645516	A	ADD	543	0,789	-1,766	0,07747
1	chr1:43646708:T:C	rs72883034	43646708	T	ADD	543	0,789	-1,766	0,07747
1	chr1:43647770:T:C	rs6429631	43647770	T	ADD	543	0,789	-1,766	0,07747
1	chr1:43648528:G:T	rs617521	43648528	G	ADD	543	0,789	-1,766	0,07747
1	chr1:43657831:G:A	rs509173	43657831	G	ADD	542	0,789	-1,764	0,07777
1	chr1:43565822:A:G	rs10890255	43565822	A	ADD	533	0,787	-1,759	0,07865
1	chr1:43560418:C:T	rs3000761	43560418	T	ADD	534	0,794	-1,735	0,08268
7	chr7:114451789:C:A	rs10262103	114451789	C	ADD	524	0,774	-1,733	0,08304
1	chr1:43592279:T:C	rs573350	43592279	T	ADD	537	0,792	-1,733	0,08315
7	chr7:114429481:C:G	rs7785701	114429481	C	ADD	520	0,772	-1,727	0,0842
1	chr1:43621825:C:T	rs673485	43621825	C	ADD	536	0,792	-1,724	0,08476
1	chr1:43777612:C:T	rs12119149	43777612	T	ADD	532	1,265	1,722	0,08504
1	chr1:43636043:A:T	rs664728	43636043	A	ADD	543	0,793	-1,721	0,08532
1	chr1:43636129:A:G	rs664336	43636129	A	ADD	543	0,793	-1,721	0,08532
1	chr1:43636586:T:C	rs530201	43636586	T	ADD	543	0,793	-1,721	0,08532
1	chr1:43563682:C:G	rs11210871	43563682	G	ADD	528	1,264	1,719	0,08557
1	chr1:43629437:A:G	rs10890268	43629437	A	ADD	540	0,793	-1,717	0,08594
1	chr1:43631843:C:G	rs2970609	43631843	C	ADD	540	0,793	-1,717	0,08594
1	chr1:43634192:G:T	rs585711	43634192	G	ADD	540	0,793	-1,717	0,08594
1	chr1:43634239:T:C	rs585652	43634239	T	ADD	540	0,793	-1,717	0,08594
1	chr1:43744582:C:G	rs6686238	43744582	G	ADD	532	1,263	1,713	0,0867
1	chr1:43745339:A:G	rs6429638	43745339	G	ADD	532	1,263	1,713	0,0867
1	chr1:43746026:T:C	rs2158956	43746026	C	ADD	532	1,263	1,713	0,0867
1	chr1:43747271:A:G	rs28833034	43747271	G	ADD	532	1,263	1,713	0,0867
1	chr1:43747507:C:A	rs7549094	43747507	A	ADD	532	1,263	1,713	0,0867
1	chr1:43751205:T:C	rs6669157	43751205	C	ADD	532	1,263	1,713	0,0867
1	chr1:43753604:T:C	rs3791042	43753604	C	ADD	532	1,263	1,713	0,0867
1	chr1:43763926:G:A	rs4660261	43763926	A	ADD	532	1,263	1,713	0,0867
1	chr1:43641757:C:T	rs2367724	43641757	T	ADD	541	1,267	1,705	0,08824
7	chr7:114416000:G:C	rs12533005	114416000	C	ADD	525	0,776	-1,696	0,0899
7	chr7:114484605:C:G	rs6974757	114484605	C	ADD	526	0,779	-1,69	0,09105
7	chr7:114476826:G:A	rs7783012	114476826	G	ADD	526	0,781	-1,68	0,09299
1	chr1:43585185:T:C	rs3828150	43585185	T	ADD	538	0,797	-1,677	0,09355
1	chr1:43662376:A:G	rs1973512	43662376	A	ADD	541	0,799	-1,674	0,09411
1	chr1:43644078:A:G	rs607296	43644078	G	ADD	540	1,261	1,673	0,09425
7	chr7:114503352:C:T	rs10266297	114503352	C	ADD	525	0,781	-1,672	0,09447
7	chr7:114470513:C:T	rs4727799	114470513	C	ADD	545	1,344	1,667	0,09547
7	chr7:114419101:C:T	rs2894699	114419101	T	ADD	525	0,779	-1,666	0,09563
1	chr1:43616545:G:A	rs667676	43616545	G	ADD	540	0,800	-1,665	0,09587
5	chr5:88497027:G:A	rs6452792	88497027	A	ADD	487	1,272	1,664	0,09614
1	chr1:43686240:C:T	rs113688544	43686240	T	ADD	528	1,400	1,659	0,09702
7	chr7:114406314:C:G	rs2030915	114406314	G	ADD	520	0,782	-1,655	0,09785
1	chr1:43556632:A:C	rs2494995	43556632	C	ADD	536	0,801	-1,655	0,09789
7	chr7:114407487:G:C	rs8180817	114407487	C	ADD	519	0,782	-1,654	0,09812
1	chr1:43879620:G:A	rs2527776	43879620	G	ADD	497	0,797	-1,644	0,1001
1	chr1:43616202:A:C	rs517849	43616202	A	ADD	540	0,802	-1,64	0,101
1	chr1:43617439:C:T	rs571862	43617439	C	ADD	540	0,802	-1,64	0,101
1	chr1:43617836:T:C	rs641365	43617836	T	ADD	540	0,802	-1,64	0,101

1	chr1:43617848:G:A	rs641351	43617848	G	ADD	540	0,802	-1,64	0,101
1	chr1:43618203:A:G	rs639929	43618203	A	ADD	540	0,802	-1,64	0,101
1	chr1:43619068:C:T	rs1143701	43619068	C	ADD	540	0,802	-1,64	0,101
1	chr1:43624269:A:G	rs559575	43624269	A	ADD	540	0,802	-1,64	0,101
1	chr1:43625004:T:C	rs531459	43625004	T	ADD	540	0,802	-1,64	0,101
1	chr1:43625065:C:T	rs628342	43625065	C	ADD	540	0,802	-1,64	0,101
1	chr1:43625603:A:G	rs2916568	43625603	A	ADD	540	0,802	-1,64	0,101
1	chr1:43778195:T:C	rs7543520	43778195	C	ADD	533	1,250	1,638	0,1014
1	chr1:43781562:C:G	rs7546040	43781562	G	ADD	533	1,250	1,638	0,1014
1	chr1:43620409:T:C	rs568639	43620409	T	ADD	539	0,802	-1,637	0,1017
1	chr1:43616041:T:G	rs516790	43616041	T	ADD	539	0,802	-1,636	0,1019
1	chr1:43606749:A:G	rs539096	43606749	A	ADD	544	0,805	-1,629	0,1033
1	chr1:43853701:G:A	rs3011225	43853701	G	ADD	545	0,810	-1,626	0,104
7	chr7:114381815:T:C	rs66571810	114381815	C	ADD	537	1,349	1,625	0,1041
7	chr7:114489082:G:A	rs10249234	114489082	G	ADD	526	0,788	-1,618	0,1057
7	chr7:114498696:G:C	rs2040658	114498696	G	ADD	526	0,788	-1,618	0,1057
7	chr7:114502195:A:G	rs1015511	114502195	A	ADD	526	0,788	-1,618	0,1057
7	chr7:114497885:A:G	rs1989903	114497885	A	ADD	526	0,789	-1,608	0,1079
1	chr1:43707752:G:A	rs112984125	43707752	A	ADD	542	1,321	1,604	0,1088
1	chr1:43722794:A:C	rs12410155	43722794	C	ADD	542	1,321	1,604	0,1088
1	chr1:43723048:A:G	rs12410444	43723048	G	ADD	542	1,321	1,604	0,1088
12	chr12:89364160:G:A	rs10506971	89364160	G	ADD	489	1,264	1,601	0,1095
12	chr12:89366967:A:G	rs1427829	89366967	A	ADD	489	1,264	1,601	0,1095
1	chr1:43853335:A:G	rs2906468	43853335	A	ADD	540	0,812	-1,595	0,1106
1	chr1:43557944:G:A	rs2842175	43557944	A	ADD	537	0,807	-1,587	0,1125
7	chr7:114403104:A:G	rs12536335	114403104	G	ADD	520	0,790	-1,586	0,1126
1	chr1:43617344:G:A	rs653953	43617344	A	ADD	539	1,247	1,584	0,1132
1	chr1:43739423:T:C	rs10749850	43739423	C	ADD	536	1,237	1,583	0,1133
1	chr1:43558813:A:G	rs11580074	43558813	A	ADD	544	0,809	-1,58	0,1142
1	chr1:43547252:C:T	rs1889588	43547252	T	ADD	477	0,796	-1,575	0,1151
1	chr1:43623961:A:G	rs643445	43623961	A	ADD	539	0,809	-1,576	0,1151
1	chr1:43555785:C:G	rs10890253	43555785	C	ADD	535	0,810	-1,57	0,1165
1	chr1:43554723:T:C	rs2842183	43554723	C	ADD	537	0,810	-1,569	0,1166
1	chr1:43556863:C:T	rs2842176	43556863	T	ADD	537	0,810	-1,569	0,1166
1	chr1:43658195:A:G	rs559481	43658195	A	ADD	530	0,811	-1,564	0,1177
7	chr7:114469294:A:G	rs727644	114469294	A	ADD	526	0,795	-1,556	0,1196
7	chr7:114479375:G:A	rs2189010	114479375	G	ADD	525	0,796	-1,55	0,1211
1	chr1:43705540:C:T	rs56319043	43705540	T	ADD	529	1,368	1,55	0,1212
1	chr1:43616091:T:G	rs516921	43616091	T	ADD	527	0,812	-1,545	0,1223
1	chr1:43577434:T:C	rs663618	43577434	T	ADD	535	0,811	-1,54	0,1237
1	chr1:43576868:C:G	rs650729	43576868	C	ADD	535	0,812	-1,534	0,1251
1	chr1:43609834:G:C	rs519669	43609834	G	ADD	539	0,815	-1,526	0,127
1	chr1:43903772:G:A	rs11210935	43903772	G	ADD	514	0,811	-1,525	0,1272
1	chr1:43900578:G:A	rs3791101	43900578	A	ADD	512	1,358	1,516	0,1294
1	chr1:43568097:C:A	rs12089622	43568097	C	ADD	532	0,816	-1,507	0,1319
1	chr1:44009451:C:T	rs113551349	44009451	T	ADD	480	1,439	1,506	0,1321
1	chr1:43649351:A:G	rs519357	43649351	A	ADD	539	0,816	-1,499	0,1339
12	chr12:89368722:C:G	rs1427828	89368722	C	ADD	484	1,244	1,492	0,1357
12	chr12:89369752:C:T	rs12819667	89369752	C	ADD	484	1,244	1,492	0,1357
3	chr3:20683524:C:A	rs56135409	20683524	A	ADD	536	0,800	-1,479	0,1391
3	chr3:20680326:A:G	rs56285459	20680326	G	ADD	536	0,801	-1,47	0,1417
3	chr3:20680427:C:T	rs55712289	20680427	T	ADD	536	0,801	-1,47	0,1417

1	chr1:43596124:C:T	rs674725	43596124	C	ADD	538	0,820	-1,467	0,1423
3	chr3:20678505:C:T	rs1846723	20678505	T	ADD	536	0,802	-1,465	0,1429
1	chr1:43504626:G:A	rs2842192	43504626	A	ADD	449	0,802	-1,458	0,1448
6	chr6:70148891:T:C	rs2025284	70148891	C	ADD	523	1,230	1,456	0,1454
1	chr1:43729733:C:T	rs4660743	43729733	T	ADD	539	1,217	1,451	0,1467
1	chr1:43887565:G:A	rs803675	43887565	G	ADD	507	0,817	-1,449	0,1472
1	chr1:43837594:A:G	rs3011217	43837594	G	ADD	524	1,217	1,446	0,1481
1	chr1:43510486:G:A	rs2819341	43510486	A	ADD	452	0,803	-1,444	0,1487
3	chr3:20683444:C:T	rs55686218	20683444	T	ADD	537	0,806	-1,436	0,1509
3	chr3:20680838:A:C	rs1604133	20680838	C	ADD	536	0,808	-1,43	0,1526
1	chr1:43737320:A:G	rs3791041	43737320	G	ADD	538	1,213	1,43	0,1527
6	chr6:70148037:A:G	rs9454993	70148037	G	ADD	525	1,223	1,428	0,1533
6	chr6:70148809:A:C	rs2025286	70148809	C	ADD	525	1,223	1,428	0,1533
3	chr3:20685150:G:A	rs4858250	20685150	A	ADD	465	0,784	-1,425	0,1542
3	chr3:20685154:C:T	rs4858251	20685154	T	ADD	465	0,784	-1,425	0,1542
6	chr6:70144291:A:G	rs9364074	70144291	G	ADD	526	1,224	1,424	0,1543
6	chr6:70142601:A:G	rs9342783	70142601	G	ADD	522	1,225	1,423	0,1548
6	chr6:70142949:A:T	rs2296013	70142949	T	ADD	522	1,225	1,423	0,1548
6	chr6:70143876:T:C	rs6935524	70143876	C	ADD	522	1,225	1,423	0,1548
1	chr1:43728545:T:G	rs10890275	43728545	G	ADD	539	1,211	1,421	0,1553
1	chr1:43728863:A:G	rs4660259	43728863	G	ADD	540	1,211	1,421	0,1554
1	chr1:43729682:T:C	rs4660260	43729682	C	ADD	540	1,211	1,421	0,1554
1	chr1:43726088:C:T	rs11210907	43726088	T	ADD	540	1,209	1,415	0,157
1	chr1:43731274:C:T	rs3862228	43731274	T	ADD	540	1,209	1,415	0,157
3	chr3:20682712:G:A	rs2886697	20682712	A	ADD	536	0,809	-1,408	0,159
1	chr1:43843195:A:G	rs3011220	43843195	G	ADD	527	1,208	1,404	0,1602
1	chr1:43720773:T:A	rs10890273	43720773	A	ADD	541	1,206	1,386	0,1657
1	chr1:43712399:T:G	rs304303	43712399	T	ADD	539	0,830	-1,381	0,1674
1	chr1:43552933:G:T	rs2819339	43552933	T	ADD	539	0,832	-1,368	0,1713
1	chr1:43896951:G:A	rs803682	43896951	G	ADD	501	0,831	-1,356	0,1753
1	chr1:43885226:G:T	rs803678	43885226	G	ADD	500	0,831	-1,351	0,1766
1	chr1:43717869:G:T	rs6429636	43717869	T	ADD	541	1,199	1,346	0,1784
5	chr5:88551455:T:A	rs12653396	88551455	T	ADD	479	1,213	1,339	0,1806
7	chr7:114505470:G:A	rs6466488	114505470	G	ADD	508	0,819	-1,332	0,1827
12	chr12:89381252:T:G	rs10506965	89381252	T	ADD	481	1,216	1,328	0,1842
1	chr1:43575295:T:G	rs10789436	43575295	T	ADD	534	0,835	-1,328	0,1843
1	chr1:43783814:A:G	rs917294	43783814	A	ADD	528	0,840	-1,305	0,1919
20	chr20:21457201:T:G	rs2424370	21457201	G	ADD	533	0,837	-1,285	0,1987
20	chr20:21463034:C:G	rs8114493	21463034	G	ADD	533	0,837	-1,285	0,1987
20	chr20:21465660:G:T	rs722095	21465660	T	ADD	533	0,838	-1,28	0,2006
5	chr5:104568525:A:G	rs12658032	104568525	A	ADD	496	0,818	-1,265	0,206
7	chr7:114430980:C:T	rs1476535	114430980	C	ADD	520	0,828	-1,259	0,2081
1	chr1:43903763:G:A	rs11210934	43903763	G	ADD	505	0,842	-1,258	0,2084
1	chr1:43572014:G:A	rs3001723	43572014	G	ADD	533	1,192	1,254	0,21
1	chr1:43831901:A:C	rs37452	43831901	C	ADD	523	1,186	1,253	0,2103
5	chr5:104482711:A:G	rs4235642	104482711	G	ADD	543	0,828	-1,25	0,2112
1	chr1:43569422:A:G	rs2842186	43569422	G	ADD	537	0,845	-1,242	0,2144
1	chr1:43840448:A:G	rs3011219	43840448	G	ADD	528	1,182	1,234	0,2171
1	chr1:43845072:C:T	rs12723279	43845072	T	ADD	528	1,182	1,234	0,2171
1	chr1:43845203:G:A	rs11210931	43845203	A	ADD	528	1,182	1,234	0,2171
1	chr1:43848594:C:T	rs2367804	43848594	T	ADD	530	1,178	1,226	0,22
1	chr1:43509093:G:T	rs882464	43509093	T	ADD	466	1,202	1,225	0,2206

1	chr1:43510950:G:C	rs10789434	43510950	C	ADD	466	1,202	1,225	0,2206
14	chr14:98224586:T:A	rs76284431	98224586	A	ADD	525	0,777	-1,206	0,2276
1	chr1:43537286:G:A	rs2819330	43537286	A	ADD	471	0,837	-1,199	0,2306
20	chr20:21456154:A:G	rs804384	21456154	G	ADD	534	0,849	-1,185	0,236
20	chr20:21456675:T:C	rs804385	21456675	C	ADD	534	0,849	-1,185	0,236
1	chr1:43689712:A:G	rs3791036	43689712	G	ADD	542	1,171	1,176	0,2397
1	chr1:43685344:T:G	rs6697354	43685344	G	ADD	545	1,171	1,175	0,24
16	chr16:61753223:C:T	rs8061672	61753223	C	ADD	537	1,176	1,167	0,2431
16	chr16:61758377:A:G	rs7202312	61758377	A	ADD	538	1,176	1,166	0,2434
16	chr16:61755912:G:C	rs9931582	61755912	G	ADD	535	1,175	1,163	0,2448
1	chr1:43565986:C:T	rs2478978	43565986	T	ADD	536	0,855	-1,159	0,2464
16	chr16:61749670:C:T	rs7206338	61749670	C	ADD	534	1,174	1,151	0,2499
5	chr5:88569970:A:G	rs1644047	88569970	A	ADD	498	0,848	-1,13	0,2583
14	chr14:98239063:A:G	rs12589260	98239063	G	ADD	524	0,798	-1,112	0,2659
1	chr1:43540668:A:G	rs2782639	43540668	G	ADD	477	0,850	-1,111	0,2664
1	chr1:43840750:C:T	rs2906471	43840750	T	ADD	528	1,160	1,1	0,2714
1	chr1:43563291:G:A	rs2842194	43563291	A	ADD	537	0,864	-1,087	0,2772
1	chr1:43667345:T:C	rs607062	43667345	T	ADD	538	0,864	-1,082	0,2794
5	chr5:88567792:C:T	rs1659071	88567792	C	ADD	498	0,856	-1,071	0,2841
5	chr5:88574430:T:C	rs10071454	88574430	T	ADD	498	0,856	-1,071	0,2841
5	chr5:88577805:C:T	rs918429	88577805	C	ADD	498	0,856	-1,071	0,2841
4	chr4:111314599:T:C	rs28865977	111314599	C	ADD	525	0,754	-1,058	0,2902
1	chr1:43663428:T:C	rs656131	43663428	T	ADD	506	0,866	-1,048	0,2944
11	chr11:28521551:G:A	rs4922805	28521551	G	ADD	500	1,158	1,026	0,3048
5	chr5:104671732:G:C	rs325501	104671732	G	ADD	522	1,180	1,026	0,305
1	chr1:43671586:C:A	rs586339	43671586	C	ADD	537	0,871	-1,024	0,3058
3	chr3:20687599:C:T	rs4858253	20687599	T	ADD	544	0,860	-1,017	0,3091
5	chr5:88579561:C:T	rs7722396	88579561	C	ADD	497	0,863	-1,01	0,3126
5	chr5:88584754:A:G	rs7721028	88584754	A	ADD	497	0,863	-1,01	0,3126
5	chr5:88586361:A:G	rs1080255	88586361	A	ADD	497	0,863	-1,01	0,3126
20	chr20:21189014:G:A	rs57940584	21189014	A	ADD	521	0,865	-1,009	0,3132
5	chr5:104655775:C:A	rs416223	104655775	C	ADD	523	1,170	0,9816	0,3263
20	chr20:21422129:C:A	rs2424362	21422129	A	ADD	538	0,876	-0,9741	0,33
11	chr11:28526196:C:T	rs7935201	28526196	C	ADD	500	1,147	0,9588	0,3377
11	chr11:28526682:A:C	rs990845	28526682	A	ADD	500	1,147	0,9588	0,3377
5	chr5:104665051:G:A	rs325481	104665051	G	ADD	522	1,165	0,9541	0,34
5	chr5:104565723:C:T	rs10059133	104565723	T	ADD	524	1,145	0,954	0,3401
5	chr5:104568065:G:A	rs12659431	104568065	A	ADD	524	1,145	0,954	0,3401
14	chr14:98259646:C:T	rs1954554	98259646	T	ADD	531	0,827	-0,9473	0,3435
11	chr11:28526861:T:C	rs990846	28526861	T	ADD	500	1,140	0,9164	0,3595
1	chr1:43872903:A:C	rs2906457	43872903	A	ADD	488	0,881	-0,9129	0,3613
5	chr5:104550512:T:G	rs6869862	104550512	T	ADD	518	1,139	0,9107	0,3625
11	chr11:28626638:C:T	rs7931459	28626638	T	ADD	528	1,160	0,8874	0,3749
11	chr11:28626783:A:T	rs7931585	28626783	T	ADD	528	1,160	0,8874	0,3749
11	chr11:28626831:G:C	rs7931884	28626831	C	ADD	528	1,160	0,8874	0,3749
5	chr5:104554395:C:T	rs6867409	104554395	C	ADD	519	1,134	0,8865	0,3753
5	chr5:104659667:A:G	rs325485	104659667	A	ADD	545	1,155	0,8862	0,3755
14	chr14:98255846:A:C	rs74469887	98255846	C	ADD	530	0,839	-0,8814	0,3781
4	chr4:111295017:T:A	rs56331412	111295017	A	ADD	535	1,283	0,8733	0,3825
4	chr4:111296367:C:T	rs17576773	111296367	T	ADD	535	1,283	0,8733	0,3825
4	chr4:111306830:A:T	rs77216804	111306830	T	ADD	535	1,283	0,8733	0,3825
14	chr14:98244157:G:A	rs78745380	98244157	A	ADD	530	0,840	-0,8714	0,3835

14	chr14:98253981:T:A	rs12590023	98253981	A	ADD	530	0,840	-0,8714	0,3835
4	chr4:111287592:T:C	rs79573484	111287592	C	ADD	534	1,277	0,8581	0,3909
20	chr20:21436041:T:G	rs811698	21436041	G	ADD	538	0,890	-0,8526	0,3939
20	chr20:21439854:C:T	rs28650836	21439854	T	ADD	538	0,890	-0,8526	0,3939
20	chr20:21440237:T:C	rs8124380	21440237	C	ADD	538	0,890	-0,8526	0,3939
20	chr20:21444512:T:A	rs57897530	21444512	A	ADD	538	0,890	-0,8526	0,3939
20	chr20:21444955:G:C	rs804381	21444955	C	ADD	538	0,890	-0,8526	0,3939
20	chr20:21449932:G:A	rs804383	21449932	A	ADD	538	0,890	-0,8526	0,3939
5	chr5:104604069:C:T	rs12187903	104604069	T	ADD	528	1,130	0,8401	0,4009
4	chr4:111333132:A:G	rs12503582	111333132	G	ADD	526	0,791	-0,8328	0,405
5	chr5:104557847:T:A	rs1592755	104557847	T	ADD	519	1,123	0,8177	0,4135
5	chr5:104605369:T:G	rs1363101	104605369	G	ADD	529	1,125	0,8166	0,4141
5	chr5:104605700:T:C	rs10455065	104605700	C	ADD	529	1,125	0,8166	0,4141
5	chr5:104605714:T:G	rs10455066	104605714	G	ADD	529	1,125	0,8166	0,4141
1	chr1:43883733:G:A	rs803679	43883733	G	ADD	499	0,890	-0,8139	0,4157
5	chr5:104712889:C:G	rs325528	104712889	C	ADD	527	1,137	0,8098	0,4181
5	chr5:104568407:A:G	rs12658019	104568407	G	ADD	521	1,119	0,7988	0,4244
20	chr20:21428075:A:G	rs804373	21428075	G	ADD	538	0,897	-0,7942	0,4271
5	chr5:104559401:T:G	rs4438849	104559401	T	ADD	518	1,117	0,7825	0,4339
5	chr5:104670966:A:G	rs325500	104670966	A	ADD	522	1,134	0,7773	0,437
5	chr5:104557287:A:T	rs1592756	104557287	A	ADD	519	1,114	0,7599	0,4473
5	chr5:104672432:G:A	rs325502	104672432	G	ADD	519	1,128	0,7553	0,4501
5	chr5:104606092:C:A	rs2018142	104606092	A	ADD	529	1,114	0,7467	0,4552
4	chr4:111311550:T:C	rs76675076	111311550	C	ADD	526	0,814	-0,7451	0,4562
4	chr4:111328737:T:G	rs7665430	111328737	G	ADD	526	0,814	-0,7451	0,4562
3	chr3:20695381:T:C	rs62234988	20695381	C	ADD	476	0,876	-0,7307	0,465
4	chr4:111311007:T:C	rs76729396	111311007	C	ADD	525	0,817	-0,729	0,466
20	chr20:21201749:T:C	rs12625304	21201749	C	ADD	512	0,872	-0,7134	0,4756
5	chr5:104559315:C:T	rs7706353	104559315	C	ADD	518	1,109	0,7126	0,4761
5	chr5:104559414:T:C	rs4320234	104559414	T	ADD	518	1,109	0,7126	0,4761
5	chr5:104560429:T:C	rs1833514	104560429	T	ADD	518	1,109	0,7126	0,4761
5	chr5:104562057:T:C	rs4521446	104562057	T	ADD	518	1,109	0,7126	0,4761
5	chr5:104563477:A:G	rs6596578	104563477	A	ADD	518	1,109	0,7126	0,4761
3	chr3:20706828:T:G	rs62234992	20706828	G	ADD	469	0,881	-0,6937	0,4879
5	chr5:104603973:C:T	rs12187898	104603973	T	ADD	532	1,104	0,6888	0,491
11	chr11:28659176:C:A	rs7947218	28659176	A	ADD	536	1,121	0,6807	0,4961
11	chr11:28655291:A:G	rs4923554	28655291	G	ADD	544	1,117	0,6791	0,4971
5	chr5:104571636:G:A	rs7703746	104571636	A	ADD	524	1,100	0,6789	0,4972
11	chr11:28554714:G:T	rs2582894	28554714	G	ADD	473	1,124	0,6777	0,498
5	chr5:104563895:T:C	rs6874138	104563895	C	ADD	524	1,100	0,6766	0,4986
5	chr5:88628064:T:G	rs1644044	88628064	G	ADD	498	0,905	-0,6759	0,4991
11	chr11:28658596:T:C	rs2356013	28658596	C	ADD	531	1,118	0,6751	0,4996
4	chr4:111310701:T:C	rs28391418	111310701	C	ADD	524	0,840	-0,6722	0,5014
5	chr5:104568333:A:G	rs12658007	104568333	G	ADD	524	1,102	0,6703	0,5027
5	chr5:104568336:T:C	rs12658451	104568336	C	ADD	524	1,102	0,6703	0,5027
5	chr5:104678081:G:A	rs21126	104678081	G	ADD	521	1,110	0,6563	0,5117
5	chr5:104563989:T:C	rs10064425	104563989	C	ADD	525	1,099	0,6524	0,5141
5	chr5:104569324:T:A	rs11242523	104569324	A	ADD	525	1,099	0,6524	0,5141
5	chr5:104569574:C:T	rs12515429	104569574	T	ADD	525	1,099	0,6524	0,5141
5	chr5:104569709:A:C	rs17156671	104569709	C	ADD	525	1,099	0,6524	0,5141
5	chr5:104570372:C:T	rs2403284	104570372	T	ADD	525	1,099	0,6524	0,5141
5	chr5:104571905:G:A	rs1421667	104571905	A	ADD	525	1,099	0,6524	0,5141

5	chr5:104568136:A:G	rs12657531	104568136	G	ADD	525	1,098	0,6487	0,5165
3	chr3:20696597:A:G	rs17809565	20696597	G	ADD	477	0,890	-0,6468	0,5178
3	chr3:20698841:C:G	rs2366311	20698841	G	ADD	477	0,890	-0,6468	0,5178
7	chr7:114552490:T:C	rs7794413	114552490	T	ADD	507	1,098	0,6459	0,5183
3	chr3:20703004:G:A	rs62234991	20703004	A	ADD	468	0,891	-0,632	0,5274
11	chr11:28522265:G:A	rs2582915	28522265	G	ADD	473	1,114	0,6321	0,5274
5	chr5:104707455:C:T	rs325521	104707455	C	ADD	528	1,102	0,6137	0,5394
3	chr3:20700799:C:T	rs55964255	20700799	T	ADD	465	0,894	-0,6135	0,5395
4	chr4:111296654:C:T	rs17638593	111296654	T	ADD	534	1,199	0,6135	0,5396
20	chr20:21187721:G:T	rs8120377	21187721	T	ADD	521	0,917	-0,6082	0,5431
20	chr20:21190532:C:T	rs765585	21190532	T	ADD	521	0,917	-0,6082	0,5431
4	chr4:111322536:C:G	rs12505218	111322536	G	ADD	523	0,854	-0,6052	0,545
5	chr5:88574395:G:A	rs10063583	88574395	G	ADD	493	0,915	-0,6022	0,547
5	chr5:104556759:C:G	rs2193933	104556759	C	ADD	518	1,088	0,5978	0,55
11	chr11:28662674:G:A	rs7934432	28662674	A	ADD	536	1,104	0,5976	0,5501
11	chr11:28664505:C:T	rs2356009	28664505	T	ADD	536	1,104	0,5976	0,5501
11	chr11:28666219:T:C	rs4923556	28666219	C	ADD	536	1,104	0,5976	0,5501
11	chr11:28668805:C:G	rs4579914	28668805	G	ADD	536	1,104	0,5976	0,5501
11	chr11:28669587:A:G	rs10767740	28669587	G	ADD	536	1,104	0,5976	0,5501
5	chr5:104566479:C:T	rs6865511	104566479	T	ADD	525	1,088	0,5848	0,5587
5	chr5:104567257:C:T	rs1421668	104567257	T	ADD	525	1,088	0,5848	0,5587
5	chr5:104572545:C:T	rs2032790	104572545	T	ADD	525	1,088	0,5848	0,5587
5	chr5:104572709:A:G	rs1421666	104572709	G	ADD	525	1,088	0,5848	0,5587
5	chr5:104573167:C:T	rs4295362	104573167	T	ADD	525	1,088	0,5848	0,5587
5	chr5:104573847:C:T	rs959857	104573847	T	ADD	525	1,088	0,5848	0,5587
11	chr11:28504006:A:G	rs2582911	28504006	A	ADD	471	1,103	0,5688	0,5695
20	chr20:21400134:C:A	rs720324	21400134	C	ADD	532	0,926	-0,5567	0,5777
5	chr5:88624488:C:A	rs1644042	88624488	A	ADD	501	0,923	-0,5508	0,5818
20	chr20:21194910:T:C	rs879064	21194910	C	ADD	521	0,925	-0,5489	0,5831
3	chr3:20627579:T:G	rs4858241	20627579	G	ADD	530	0,929	-0,5447	0,586
5	chr5:88605345:G:A	rs6859545	88605345	A	ADD	502	0,924	-0,5408	0,5886
5	chr5:88618013:G:A	rs324895	88618013	A	ADD	502	0,924	-0,5408	0,5886
5	chr5:88618446:A:G	rs324896	88618446	G	ADD	502	0,924	-0,5408	0,5886
5	chr5:88619663:G:T	rs10462334	88619663	T	ADD	502	0,924	-0,5408	0,5886
20	chr20:21187755:C:T	rs8120293	21187755	T	ADD	520	0,927	-0,5338	0,5935
7	chr7:114407085:A:T	rs7795397	114407085	T	ADD	520	1,080	0,531	0,5954
5	chr5:88630645:G:A	rs11740358	88630645	A	ADD	498	0,925	-0,5304	0,5958
11	chr11:28620834:C:T	rs10767734	28620834	T	ADD	521	1,082	0,5301	0,596
5	chr5:104709685:C:A	rs325523	104709685	C	ADD	545	1,086	0,5291	0,5967
5	chr5:88636991:G:T	rs4916661	88636991	T	ADD	500	0,926	-0,5246	0,5998
20	chr20:21412561:A:G	rs6132424	21412561	G	ADD	545	0,932	-0,5247	0,5998
5	chr5:88633148:G:T	rs1628250	88633148	T	ADD	500	0,926	-0,5245	0,6
11	chr11:28634517:A:T	rs7942078	28634517	T	ADD	536	1,091	0,5235	0,6006
11	chr11:28634687:C:A	rs7942417	28634687	A	ADD	536	1,091	0,5235	0,6006
11	chr11:28644991:C:T	rs7933262	28644991	T	ADD	536	1,091	0,5235	0,6006
7	chr7:114451698:G:A	rs10262192	114451698	G	ADD	544	1,076	0,5188	0,6039
5	chr5:88601453:A:C	rs324885	88601453	A	ADD	487	0,926	-0,5162	0,6057
11	chr11:28621752:T:C	rs10835368	28621752	T	ADD	517	0,930	-0,5127	0,6081
5	chr5:104569213:T:C	rs11242522	104569213	C	ADD	526	1,076	0,5095	0,6104
4	chr4:111309356:T:A	rs12500836	111309356	A	ADD	529	0,871	-0,5074	0,6119
4	chr4:111582716:G:A	rs11729080	111582716	A	ADD	505	0,838	-0,5028	0,6151
11	chr11:28605957:T:C	rs10835363	28605957	C	ADD	543	1,073	0,5023	0,6154

5	chr5:104698439:C:T	rs410915	104698439	C	ADD	528	1,078	0,4759	0,6341
5	chr5:104581838:T:A	rs10477835	104581838	A	ADD	537	1,078	0,4709	0,6377
5	chr5:104582136:T:C	rs1363103	104582136	C	ADD	537	1,078	0,4709	0,6377
11	chr11:28648922:G:A	rs7105480	28648922	A	ADD	537	1,081	0,4695	0,6387
11	chr11:28654958:T:C	rs10835389	28654958	C	ADD	537	1,081	0,4695	0,6387
11	chr11:28655314:A:G	rs4923555	28655314	G	ADD	537	1,081	0,4695	0,6387
11	chr11:28657323:A:G	rs10767739	28657323	G	ADD	537	1,081	0,4695	0,6387
11	chr11:28511111:T:C	rs2585807	28511111	T	ADD	471	1,084	0,4693	0,6389
7	chr7:114507162:C:A	rs10279936	114507162	C	ADD	514	1,069	0,4688	0,6392
5	chr5:88867954:G:A	rs61104616	88867954	A	ADD	517	0,927	-0,4655	0,6416
11	chr11:28646737:A:G	rs7481939	28646737	G	ADD	536	1,080	0,4651	0,6419
7	chr7:114474994:A:G	rs2396751	114474994	A	ADD	544	1,067	0,4605	0,6451
11	chr11:28655215:G:C	rs4923553	28655215	C	ADD	537	1,079	0,4585	0,6466
20	chr20:21165245:A:G	rs6035795	21165245	A	ADD	542	0,939	-0,4562	0,6483
7	chr7:114508188:A:G	rs2189015	114508188	A	ADD	509	0,931	-0,4538	0,65
5	chr5:88629003:C:T	rs1644045	88629003	T	ADD	500	0,936	-0,4507	0,6522
7	chr7:114508276:C:A	rs2396753	114508276	C	ADD	509	0,932	-0,4492	0,6533
5	chr5:88461101:C:T	rs4571506	88461101	C	ADD	543	1,064	0,4457	0,6558
7	chr7:114464334:T:C	rs10228327	114464334	T	ADD	540	1,064	0,4409	0,6593
11	chr11:28650082:G:C	rs10835387	28650082	C	ADD	536	1,076	0,4388	0,6608
7	chr7:114482451:C:T	rs2106900	114482451	C	ADD	540	1,062	0,4382	0,6612
7	chr7:114499629:G:A	rs12113612	114499629	G	ADD	541	1,063	0,4355	0,6632
5	chr5:88600784:C:T	rs324886	88600784	T	ADD	496	0,940	-0,4268	0,6695
7	chr7:114456791:A:T	rs10231382	114456791	A	ADD	539	1,062	0,4231	0,6722
5	chr5:104646025:T:G	rs40465	104646025	G	ADD	533	1,079	0,4194	0,6749
11	chr11:28621106:G:A	rs4636654	28621106	A	ADD	509	1,065	0,4188	0,6753
7	chr7:114531090:A:G	rs9332390	114531090	A	ADD	512	1,061	0,4159	0,6775
20	chr20:21227106:T:G	rs6035813	21227106	T	ADD	544	0,946	-0,4069	0,6841
7	chr7:114522685:G:A	rs6980093	114522685	G	ADD	509	0,939	-0,4058	0,6849
3	chr3:71450250:C:T	rs17718444	71450250	T	ADD	532	1,071	0,4036	0,6865
5	chr5:104578822:T:C	rs13164188	104578822	C	ADD	544	1,062	0,3802	0,7038
11	chr11:28620773:G:A	rs10767733	28620773	A	ADD	521	1,057	0,3799	0,704
5	chr5:88588020:G:C	rs56022653	88588020	C	ADD	500	0,947	-0,3746	0,7079
7	chr7:114540007:A:G	rs10262462	114540007	A	ADD	507	0,943	-0,3734	0,7088
7	chr7:114475213:T:C	rs2189011	114475213	T	ADD	541	1,054	0,3728	0,7093
5	chr5:88587685:T:C	rs62369151	88587685	C	ADD	501	0,948	-0,3648	0,7153
5	chr5:104686538:C:G	rs396755	104686538	C	ADD	525	1,058	0,3575	0,7207
7	chr7:114503596:T:A	rs9649368	114503596	T	ADD	541	1,050	0,3464	0,729
7	chr7:114520816:G:A	rs6969376	114520816	G	ADD	509	0,948	-0,3452	0,7299
11	chr11:28619515:G:A	rs12226518	28619515	A	ADD	521	1,051	0,3375	0,7358
11	chr11:28619698:G:A	rs12226542	28619698	A	ADD	521	1,051	0,3375	0,7358
20	chr20:21293229:C:T	rs6113185	21293229	C	ADD	519	0,948	-0,3341	0,7383
20	chr20:21294030:G:A	rs6035837	21294030	G	ADD	519	0,948	-0,3341	0,7383
20	chr20:21074404:T:C	rs6035772	21074404	T	ADD	526	0,956	-0,3298	0,7415
5	chr5:88558577:A:C	rs4916723	88558577	C	ADD	475	0,952	-0,3273	0,7434
11	chr11:28609937:C:T	rs11030387	28609937	T	ADD	521	1,049	0,3231	0,7466
11	chr11:28620721:G:A	rs10767732	28620721	A	ADD	521	1,048	0,3224	0,7471
5	chr5:104581651:A:C	rs10477834	104581651	C	ADD	534	1,047	0,3179	0,7506
5	chr5:104582166:G:T	rs1363102	104582166	T	ADD	534	1,047	0,3179	0,7506
5	chr5:104582921:C:T	rs10072849	104582921	T	ADD	534	1,047	0,3179	0,7506
5	chr5:104582998:G:A	rs10057459	104582998	A	ADD	534	1,047	0,3179	0,7506
5	chr5:104583048:G:T	rs10057469	104583048	T	ADD	534	1,047	0,3179	0,7506

5	chr5:104584121:A:C	rs10052804	104584121	C	ADD	534	1,047	0,3179	0,7506
7	chr7:114483253:C:T	rs2189008	114483253	C	ADD	541	1,044	0,3053	0,7601
11	chr11:28654592:G:A	rs11030401	28654592	A	ADD	527	0,957	-0,3045	0,7608
11	chr11:28609522:G:C	rs12226610	28609522	C	ADD	522	1,045	0,3024	0,7624
11	chr11:28609701:C:A	rs11030386	28609701	A	ADD	522	1,045	0,3024	0,7624
5	chr5:104584139:G:A	rs10059643	104584139	A	ADD	533	1,044	0,3	0,7642
11	chr11:28605239:G:A	rs10835362	28605239	A	ADD	523	1,045	0,2984	0,7654
11	chr11:28606106:G:A	rs10835364	28606106	A	ADD	523	1,045	0,2984	0,7654
11	chr11:28607002:C:A	rs57806515	28607002	A	ADD	523	1,045	0,2984	0,7654
11	chr11:28607568:G:A	rs11030385	28607568	A	ADD	523	1,045	0,2984	0,7654
11	chr11:28613204:C:T	rs10767729	28613204	T	ADD	523	1,045	0,2984	0,7654
11	chr11:28613315:A:G	rs10767730	28613315	G	ADD	523	1,045	0,2984	0,7654
11	chr11:28613316:T:C	rs10767731	28613316	C	ADD	523	1,045	0,2984	0,7654
11	chr11:28614382:G:A	rs7110556	28614382	A	ADD	523	1,045	0,2984	0,7654
11	chr11:28615789:A:G	rs4923549	28615789	G	ADD	523	1,045	0,2984	0,7654
20	chr20:21294655:A:C	rs6075789	21294655	A	ADD	520	0,953	-0,2983	0,7655
11	chr11:28639315:G:T	rs7938122	28639315	T	ADD	535	0,958	-0,2975	0,7661
5	chr5:104582527:C:T	rs10072579	104582527	T	ADD	535	1,044	0,2965	0,7669
5	chr5:88865600:T:C	rs12054920	88865600	C	ADD	518	0,954	-0,2873	0,7739
20	chr20:21299182:T:G	rs4813424	21299182	T	ADD	521	0,956	-0,2797	0,7797
11	chr11:28628549:C:T	rs4471400	28628549	T	ADD	526	0,963	-0,2635	0,7922
11	chr11:28621014:G:C	rs10767735	28621014	C	ADD	517	1,039	0,2608	0,7942
11	chr11:28609187:T:G	rs11821132	28609187	G	ADD	521	1,039	0,2594	0,7954
20	chr20:21289658:C:T	rs2328622	21289658	C	ADD	513	0,959	-0,2581	0,7963
11	chr11:28646269:A:G	rs4534569	28646269	G	ADD	528	0,964	-0,257	0,7972
20	chr20:21291449:A:T	rs6047360	21291449	A	ADD	513	0,960	-0,2535	0,7999
11	chr11:28645542:T:C	rs7130649	28645542	C	ADD	527	0,964	-0,2533	0,8
5	chr5:104742532:A:C	rs60271	104742532	A	ADD	519	1,046	0,2452	0,8063
5	chr5:104746478:A:C	rs323509	104746478	A	ADD	519	1,046	0,2452	0,8063
5	chr5:104628884:G:A	rs77960	104628884	A	ADD	536	1,046	0,2448	0,8066
20	chr20:21291893:C:T	rs6047361	21291893	C	ADD	513	0,961	-0,242	0,8088
5	chr5:104577563:A:C	rs11738197	104577563	C	ADD	532	1,035	0,2364	0,8131
5	chr5:104577567:C:T	rs13172611	104577567	T	ADD	532	1,035	0,2364	0,8131
5	chr5:104577620:T:A	rs13158494	104577620	A	ADD	532	1,035	0,2364	0,8131
5	chr5:104578055:A:T	rs1363106	104578055	T	ADD	532	1,035	0,2364	0,8131
5	chr5:104578313:C:T	rs1421665	104578313	T	ADD	532	1,035	0,2364	0,8131
5	chr5:104578842:G:A	rs13177365	104578842	A	ADD	532	1,035	0,2364	0,8131
5	chr5:104579057:A:G	rs10053368	104579057	G	ADD	532	1,035	0,2364	0,8131
5	chr5:104579085:A:T	rs10053371	104579085	T	ADD	532	1,035	0,2364	0,8131
5	chr5:104579135:T:G	rs10054977	104579135	G	ADD	532	1,035	0,2364	0,8131
5	chr5:104579581:A:G	rs12658276	104579581	G	ADD	532	1,035	0,2364	0,8131
5	chr5:104579742:G:A	rs10479296	104579742	A	ADD	532	1,035	0,2364	0,8131
5	chr5:104580027:A:G	rs10479297	104580027	G	ADD	532	1,035	0,2364	0,8131
5	chr5:104580648:T:C	rs7710489	104580648	C	ADD	532	1,035	0,2364	0,8131
5	chr5:104580662:G:C	rs7723509	104580662	C	ADD	532	1,035	0,2364	0,8131
5	chr5:104581130:A:G	rs62362459	104581130	G	ADD	532	1,035	0,2364	0,8131
5	chr5:104577501:A:G	rs11738191	104577501	G	ADD	530	1,035	0,2362	0,8133
5	chr5:104739429:T:C	rs6421926	104739429	T	ADD	518	1,043	0,2281	0,8196
20	chr20:21195965:C:T	rs2144782	21195965	T	ADD	490	0,968	-0,2265	0,8208
11	chr11:28658309:G:A	rs11030403	28658309	A	ADD	529	0,968	-0,2264	0,8209
11	chr11:28632740:G:A	rs12282355	28632740	A	ADD	535	0,969	-0,2193	0,8264
11	chr11:28633321:G:A	rs7935241	28633321	A	ADD	535	0,969	-0,2193	0,8264

11	chr11:28633511:A:C	rs7938026	28633511	C	ADD	535	0,969	-0,2193	0,8264
11	chr11:28634125:G:T	rs4244538	28634125	T	ADD	535	0,969	-0,2193	0,8264
11	chr11:28634351:C:G	rs6484378	28634351	G	ADD	535	0,969	-0,2193	0,8264
11	chr11:28634462:T:G	rs7928893	28634462	G	ADD	535	0,969	-0,2193	0,8264
11	chr11:28635703:A:G	rs4344470	28635703	G	ADD	535	0,969	-0,2193	0,8264
11	chr11:28637410:G:A	rs6484379	28637410	A	ADD	535	0,969	-0,2193	0,8264
11	chr11:28638055:A:T	rs3935074	28638055	T	ADD	535	0,969	-0,2193	0,8264
11	chr11:28638658:T:C	rs4337023	28638658	C	ADD	535	0,969	-0,2193	0,8264
11	chr11:28644512:C:G	rs11030397	28644512	G	ADD	535	0,969	-0,2193	0,8264
11	chr11:28644517:G:A	rs11030398	28644517	A	ADD	535	0,969	-0,2193	0,8264
11	chr11:28644793:A:C	rs11030399	28644793	C	ADD	535	0,969	-0,2193	0,8264
5	chr5:88864537:C:T	rs56947091	88864537	T	ADD	525	0,965	-0,2176	0,8277
11	chr11:28636483:A:G	rs12577364	28636483	G	ADD	543	0,971	-0,2118	0,8322
20	chr20:21287631:G:A	rs6075788	21287631	G	ADD	514	0,967	-0,2059	0,8369
20	chr20:21290685:C:G	rs4813422	21290685	C	ADD	514	0,967	-0,2059	0,8369
20	chr20:21290915:A:G	rs6082372	21290915	A	ADD	514	0,967	-0,2059	0,8369
20	chr20:21290947:G:A	rs6035834	21290947	G	ADD	514	0,967	-0,2059	0,8369
20	chr20:21291136:G:T	rs6047359	21291136	G	ADD	514	0,967	-0,2059	0,8369
20	chr20:21292673:G:A	rs4815038	21292673	G	ADD	514	0,967	-0,2059	0,8369
16	chr16:61746654:A:G	rs4131786	61746654	G	ADD	537	0,970	-0,2053	0,8374
16	chr16:61754668:C:T	rs7192912	61754668	T	ADD	537	0,970	-0,2053	0,8374
11	chr11:28604117:C:T	rs4442537	28604117	T	ADD	523	1,031	0,2051	0,8375
11	chr11:28655190:A:G	rs4923552	28655190	G	ADD	530	0,972	-0,2021	0,8398
11	chr11:28655717:T:C	rs7942482	28655717	C	ADD	530	0,972	-0,2021	0,8398
11	chr11:28632994:A:G	rs10835384	28632994	G	ADD	532	0,972	-0,1981	0,8429
11	chr11:28639271:A:G	rs7937781	28639271	G	ADD	532	0,972	-0,1981	0,8429
11	chr11:28586227:G:A	rs4923546	28586227	A	ADD	514	0,971	-0,1976	0,8433
11	chr11:28617583:T:C	rs12221661	28617583	C	ADD	519	1,028	0,1893	0,8498
11	chr11:28624037:T:C	rs4267058	28624037	C	ADD	528	0,974	-0,185	0,8533
11	chr11:28628989:A:G	rs4568992	28628989	G	ADD	528	0,974	-0,185	0,8533
11	chr11:28629363:A:G	rs6484376	28629363	G	ADD	528	0,974	-0,185	0,8533
20	chr20:21291031:G:C	rs6035835	21291031	G	ADD	511	0,971	-0,1821	0,8555
11	chr11:28609199:A:G	rs59617050	28609199	G	ADD	520	1,027	0,1818	0,8558
11	chr11:28627190:A:G	rs11030393	28627190	G	ADD	540	0,976	-0,1778	0,8589
20	chr20:21371598:A:G	rs6047404	21371598	A	ADD	545	1,028	0,1769	0,8596
11	chr11:28630347:G:C	rs7951682	28630347	C	ADD	528	0,976	-0,1729	0,8627
11	chr11:28646124:G:A	rs2883826	28646124	A	ADD	530	0,976	-0,1715	0,8638
11	chr11:28646393:C:T	rs4372430	28646393	T	ADD	530	0,976	-0,1715	0,8638
11	chr11:28646869:T:C	rs6484385	28646869	C	ADD	530	0,976	-0,1715	0,8638
11	chr11:28648417:T:C	rs7942414	28648417	C	ADD	530	0,976	-0,1715	0,8638
11	chr11:28649222:A:G	rs2356016	28649222	G	ADD	530	0,976	-0,1715	0,8638
11	chr11:28649587:T:C	rs2356015	28649587	C	ADD	530	0,976	-0,1715	0,8638
11	chr11:28649653:C:G	rs2356014	28649653	G	ADD	530	0,976	-0,1715	0,8638
11	chr11:28650803:C:G	rs10835388	28650803	G	ADD	530	0,976	-0,1715	0,8638
11	chr11:28654623:A:G	rs11030402	28654623	G	ADD	530	0,976	-0,1715	0,8638
4	chr4:111500989:G:A	rs72678864	111500989	A	ADD	511	1,055	0,1712	0,8641
11	chr11:28648570:G:T	rs6484386	28648570	T	ADD	526	0,976	-0,1711	0,8642
11	chr11:28648575:T:A	rs6484387	28648575	A	ADD	526	0,976	-0,1711	0,8642
5	chr5:104620815:C:G	rs171697	104620815	G	ADD	536	1,031	0,1702	0,8649
5	chr5:104636656:G:A	rs30266	104636656	A	ADD	536	1,031	0,1702	0,8649
5	chr5:104734216:A:G	rs161645	104734216	A	ADD	543	1,029	0,1619	0,8714
5	chr5:88563112:T:G	rs17422060	88563112	G	ADD	497	0,977	-0,159	0,8737

11	chr11:28608504:A:G	rs10835365	28608504	G	ADD	521	1,023	0,1522	0,879
20	chr20:21038664:T:C	rs6082289	21038664	T	ADD	518	1,024	0,1523	0,879
11	chr11:28622480:G:A	rs10835375	28622480	A	ADD	527	0,979	-0,1519	0,8793
20	chr20:21350903:T:G	rs6047396	21350903	T	ADD	522	0,977	-0,1455	0,8843
11	chr11:28603810:A:T	rs11030384	28603810	T	ADD	521	1,022	0,1449	0,8848
20	chr20:21372771:A:C	rs4815041	21372771	A	ADD	523	1,023	0,1442	0,8854
7	chr7:114544043:G:A	rs7458242	114544043	G	ADD	504	0,978	-0,1439	0,8856
5	chr5:104602414:C:T	rs1592754	104602414	T	ADD	531	1,021	0,1413	0,8876
20	chr20:21277855:T:C	rs6047345	21277855	T	ADD	513	0,978	-0,1398	0,8888
20	chr20:21277966:T:C	rs6047346	21277966	T	ADD	513	0,978	-0,1398	0,8888
11	chr11:28615816:T:C	rs4923550	28615816	C	ADD	521	1,020	0,1353	0,8924
20	chr20:21252915:A:G	rs1000176	21252915	A	ADD	520	0,981	-0,1283	0,8979
20	chr20:21299701:C:T	rs6082377	21299701	C	ADD	521	0,981	-0,1213	0,9035
5	chr5:104576507:C:A	rs10071115	104576507	A	ADD	530	1,018	0,1193	0,905
20	chr20:21154158:T:C	rs6047273	21154158	T	ADD	545	1,016	0,1189	0,9054
20	chr20:21283152:A:T	rs6035826	21283152	A	ADD	514	0,981	-0,1173	0,9067
20	chr20:21284820:G:A	rs4815036	21284820	G	ADD	514	0,981	-0,1173	0,9067
20	chr20:21285090:C:T	rs6035830	21285090	C	ADD	514	0,981	-0,1173	0,9067
20	chr20:21285802:C:T	rs6035831	21285802	C	ADD	514	0,981	-0,1173	0,9067
20	chr20:21286713:C:T	rs726024	21286713	C	ADD	514	0,981	-0,1173	0,9067
20	chr20:21372841:G:C	rs4815042	21372841	G	ADD	525	1,019	0,1158	0,9078
11	chr11:28628323:A:G	rs7121736	28628323	G	ADD	527	0,984	-0,1148	0,9086
20	chr20:21270205:T:C	rs6082363	21270205	T	ADD	516	1,018	0,113	0,91
20	chr20:21283529:T:C	rs6047353	21283529	T	ADD	513	0,982	-0,1105	0,912
11	chr11:28602326:G:T	rs7931440	28602326	G	ADD	516	1,015	0,1047	0,9166
5	chr5:104702059:G:A	rs12055234	104702059	A	ADD	537	0,982	-0,09798	0,9219
2	chr2:144956787:A:C	rs1438898	144956787	C	ADD	519	0,985	-0,09628	0,9233
7	chr7:114440281:T:G	rs2014265	114440281	T	ADD	535	1,013	0,09147	0,9271
16	chr16:61754062:A:G	rs7187101	61754062	G	ADD	545	0,987	-0,08798	0,9299
7	chr7:114442439:T:C	rs10261780	114442439	T	ADD	536	1,013	0,08685	0,9308
20	chr20:21379890:A:G	rs6047414	21379890	A	ADD	527	1,013	0,08237	0,9344
20	chr20:21388294:G:T	rs6035865	21388294	G	ADD	527	1,013	0,08237	0,9344
20	chr20:21281046:C:T	rs6047348	21281046	C	ADD	514	0,987	-0,08092	0,9355
11	chr11:28580674:A:G	rs2585817	28580674	G	ADD	513	0,988	-0,08034	0,936
5	chr5:88861735:T:C	rs4518438	88861735	C	ADD	541	0,988	-0,07984	0,9364
11	chr11:28671984:T:G	rs4923557	28671984	G	ADD	522	1,011	0,07781	0,938
11	chr11:28672893:C:G	rs10835391	28672893	G	ADD	522	1,011	0,07781	0,938
5	chr5:104576413:A:C	rs62362443	104576413	C	ADD	528	1,011	0,07499	0,9402
20	chr20:21371667:C:T	rs6035860	21371667	C	ADD	526	1,012	0,07427	0,9408
20	chr20:21373277:C:T	rs6047405	21373277	C	ADD	526	1,012	0,07427	0,9408
7	chr7:114434202:C:T	rs10268637	114434202	C	ADD	523	1,011	0,07417	0,9409
5	chr5:104612267:T:C	rs2431108	104612267	C	ADD	537	0,987	-0,07136	0,9431
11	chr11:28580626:C:A	rs2582895	28580626	A	ADD	514	0,990	-0,06834	0,9455
11	chr11:28631941:T:G	rs6484377	28631941	G	ADD	533	0,990	-0,0682	0,9456
11	chr11:28632083:T:C	rs7124523	28632083	C	ADD	533	0,990	-0,0682	0,9456
11	chr11:28632180:T:G	rs7124653	28632180	G	ADD	533	0,990	-0,0682	0,9456
11	chr11:28632479:C:T	rs4290216	28632479	T	ADD	533	0,990	-0,0682	0,9456
11	chr11:28631449:A:G	rs4275621	28631449	G	ADD	529	1,009	0,06299	0,9498
11	chr11:28669740:C:T	rs7119404	28669740	T	ADD	524	1,009	0,06122	0,9512
11	chr11:28582930:C:T	rs11030382	28582930	T	ADD	516	1,009	0,06044	0,9518
20	chr20:21308964:A:G	rs6035839	21308964	A	ADD	520	1,010	0,05925	0,9528
20	chr20:21315921:C:A	rs6047372	21315921	C	ADD	520	1,010	0,05925	0,9528

20	chr20:21310893:C:T	rs6035840	21310893	C	ADD	519	1,009	0,05615	0,9552
20	chr20:21311200:C:T	rs6082384	21311200	C	ADD	519	1,009	0,05615	0,9552
20	chr20:21311590:G:C	rs6035841	21311590	G	ADD	519	1,009	0,05615	0,9552
20	chr20:21312173:C:T	rs6047370	21312173	C	ADD	519	1,009	0,05615	0,9552
20	chr20:21312391:G:A	rs6047371	21312391	G	ADD	519	1,009	0,05615	0,9552
11	chr11:28622330:G:A	rs10835371	28622330	A	ADD	529	0,992	-0,05507	0,9561
11	chr11:28622366:T:C	rs10835372	28622366	C	ADD	529	0,992	-0,05507	0,9561
11	chr11:28622395:T:C	rs10835373	28622395	C	ADD	529	0,992	-0,05507	0,9561
11	chr11:28622424:G:A	rs10835374	28622424	A	ADD	529	0,992	-0,05507	0,9561
11	chr11:28622491:T:G	rs10767736	28622491	G	ADD	529	0,992	-0,05507	0,9561
11	chr11:28622565:G:T	rs11030388	28622565	T	ADD	529	0,992	-0,05507	0,9561
11	chr11:28622745:C:A	rs73434729	28622745	A	ADD	529	0,992	-0,05507	0,9561
11	chr11:28623055:A:G	rs12576604	28623055	G	ADD	529	0,992	-0,05507	0,9561
11	chr11:28623079:A:G	rs11030391	28623079	G	ADD	529	0,992	-0,05507	0,9561
11	chr11:28623338:T:C	rs10835382	28623338	C	ADD	529	0,992	-0,05507	0,9561
11	chr11:28624151:G:A	rs4274191	28624151	A	ADD	529	0,992	-0,05507	0,9561
11	chr11:28624272:T:G	rs10767737	28624272	G	ADD	529	0,992	-0,05507	0,9561
11	chr11:28625064:T:A	rs4351802	28625064	A	ADD	529	0,992	-0,05507	0,9561
11	chr11:28625609:A:T	rs4434991	28625609	T	ADD	529	0,992	-0,05507	0,9561
11	chr11:28626408:G:T	rs4290215	28626408	T	ADD	529	0,992	-0,05507	0,9561
11	chr11:28627124:G:A	rs11030392	28627124	A	ADD	529	0,992	-0,05507	0,9561
11	chr11:28628635:A:G	rs4575255	28628635	G	ADD	529	0,992	-0,05507	0,9561
11	chr11:28629811:A:C	rs10835383	28629811	C	ADD	529	0,992	-0,05507	0,9561
2	chr2:144962572:A:G	rs72854462	144962572	G	ADD	518	0,992	-0,05218	0,9584
20	chr20:21390733:A:G	rs1555946	21390733	A	ADD	526	1,008	0,05038	0,9598
11	chr11:28622320:A:G	rs10742197	28622320	G	ADD	529	0,993	-0,04902	0,9609
11	chr11:28624925:C:T	rs4576808	28624925	T	ADD	529	0,993	-0,04902	0,9609
11	chr11:28582122:A:G	rs7128734	28582122	G	ADD	514	0,993	-0,04817	0,9616
11	chr11:28585152:T:C	rs10835360	28585152	C	ADD	514	0,993	-0,04817	0,9616
11	chr11:28587183:C:G	rs4378371	28587183	G	ADD	514	0,993	-0,04817	0,9616
11	chr11:28593837:A:G	rs2585814	28593837	G	ADD	514	0,993	-0,04817	0,9616
11	chr11:28594152:A:G	rs2582896	28594152	G	ADD	514	0,993	-0,04817	0,9616
11	chr11:28594153:T:C	rs2582897	28594153	C	ADD	514	0,993	-0,04817	0,9616
11	chr11:28594704:T:C	rs10742196	28594704	C	ADD	514	0,993	-0,04817	0,9616
11	chr11:28597209:G:A	rs10767727	28597209	A	ADD	514	0,993	-0,04817	0,9616
5	chr5:88862329:G:C	rs7730255	88862329	C	ADD	526	0,992	-0,04716	0,9624
11	chr11:28666860:G:A	rs7129601	28666860	A	ADD	525	1,006	0,04215	0,9664
20	chr20:21345460:T:C	rs13043724	21345460	T	ADD	527	0,993	-0,04157	0,9668
20	chr20:21346867:T:G	rs6047389	21346867	T	ADD	527	0,993	-0,04157	0,9668
20	chr20:21349142:T:C	rs1958122	21349142	T	ADD	527	0,993	-0,04157	0,9668
20	chr20:21350734:G:A	rs6047395	21350734	G	ADD	527	0,993	-0,04157	0,9668
20	chr20:21328174:G:A	rs6047380	21328174	G	ADD	521	0,994	-0,03688	0,9706
4	chr4:111485805:C:T	rs72678859	111485805	T	ADD	513	1,009	0,02918	0,9767
11	chr11:28623283:A:G	rs10835381	28623283	G	ADD	529	1,004	0,02807	0,9776
11	chr11:28623794:G:C	rs4268495	28623794	C	ADD	529	1,004	0,02807	0,9776
11	chr11:28628859:C:T	rs4350350	28628859	T	ADD	529	1,004	0,02807	0,9776
7	chr7:114435085:T:C	rs28534444	114435085	T	ADD	522	0,996	-0,02787	0,9778
5	chr5:104619100:T:A	rs173823	104619100	A	ADD	536	0,995	-0,02743	0,9781
20	chr20:21351811:A:G	rs6035852	21351811	A	ADD	527	1,004	0,0273	0,9782
20	chr20:21352024:G:C	rs6035853	21352024	G	ADD	527	1,004	0,0273	0,9782
20	chr20:21353013:G:C	rs6082393	21353013	G	ADD	527	1,004	0,0273	0,9782
20	chr20:21353030:G:A	rs6075794	21353030	G	ADD	527	1,004	0,0273	0,9782

20	chr20:21359219:A:G	rs1409816	21359219	A	ADD	527	1,004	0,0273	0,9782
20	chr20:21361740:T:C	rs4497932	21361740	T	ADD	527	1,004	0,0273	0,9782
11	chr11:28585698:T:C	rs7952220	28585698	C	ADD	513	0,996	-0,02503	0,98
20	chr20:21374422:T:A	rs6035862	21374422	T	ADD	526	1,004	0,02485	0,9802
20	chr20:21375208:A:G	rs6047407	21375208	A	ADD	526	1,004	0,02485	0,9802
7	chr7:114554560:T:G	rs1859100	114554560	T	ADD	503	0,997	-0,02197	0,9825
11	chr11:28590845:G:A	rs4520584	28590845	A	ADD	516	1,003	0,01998	0,9841
11	chr11:28592943:A:T	rs2585813	28592943	T	ADD	516	1,003	0,01998	0,9841
11	chr11:28596075:C:A	rs4244537	28596075	A	ADD	516	1,003	0,01998	0,9841
11	chr11:28596496:C:G	rs10835361	28596496	G	ADD	516	1,003	0,01998	0,9841
11	chr11:28596864:G:A	rs2585811	28596864	A	ADD	516	1,003	0,01998	0,9841
20	chr20:21318093:T:C	rs6047374	21318093	T	ADD	521	1,002	0,01147	0,9908
20	chr20:21318094:G:T	rs6047375	21318094	G	ADD	521	1,002	0,01147	0,9908
20	chr20:21319392:G:A	rs1327153	21319392	G	ADD	521	1,002	0,01147	0,9908
20	chr20:21320839:C:T	rs6047377	21320839	C	ADD	521	1,002	0,01147	0,9908
20	chr20:21321927:C:T	rs1546997	21321927	C	ADD	521	1,002	0,01147	0,9908
20	chr20:21321943:T:C	rs1546998	21321943	T	ADD	521	1,002	0,01147	0,9908
20	chr20:21327058:G:A	rs4813426	21327058	G	ADD	521	1,002	0,01147	0,9908
20	chr20:21329677:C:T	rs4496388	21329677	C	ADD	521	1,002	0,01147	0,9908
5	chr5:88851954:G:A	rs2362108	88851954	A	ADD	525	0,998	-0,01106	0,9912
5	chr5:88852224:C:T	rs2362109	88852224	T	ADD	525	0,998	-0,01106	0,9912
5	chr5:88853474:A:G	rs17487515	88853474	G	ADD	525	0,998	-0,01106	0,9912
5	chr5:88853509:G:A	rs17559709	88853509	A	ADD	525	0,998	-0,01106	0,9912
5	chr5:88854472:G:A	rs4145738	88854472	A	ADD	525	0,998	-0,01106	0,9912
5	chr5:88855175:T:A	rs57055575	88855175	A	ADD	525	0,998	-0,01106	0,9912
5	chr5:88856385:T:C	rs4373304	88856385	C	ADD	525	0,998	-0,01106	0,9912
5	chr5:88856757:C:A	rs11749060	88856757	A	ADD	525	0,998	-0,01106	0,9912
5	chr5:88858057:C:T	rs55740951	88858057	T	ADD	525	0,998	-0,01106	0,9912
20	chr20:21313246:C:T	rs1359761	21313246	C	ADD	520	1,001	0,008393	0,9933
11	chr11:28569621:A:T	rs11030380	28569621	A	ADD	511	1,001	0,005084	0,9959
11	chr11:28570040:C:T	rs7127383	28570040	C	ADD	511	1,001	0,005084	0,9959
7	chr7:114435631:A:C	rs10246733	114435631	A	ADD	523	1,000	0,002375	0,9981
11	chr11:28578333:T:A	rs11030381	28578333	T	ADD	511	1,000	-0,00157	0,9987
4	chr4:111546456:G:A	rs3934797	111546456	A	ADD	469	#N/D	#N/D	#N/D
5	chr5:88385177:A:G	rs6452784	88385177	A	ADD	519	#N/D	#N/D	#N/D
5	chr5:88386213:G:A	rs6414945	88386213	G	ADD	520	#N/D	#N/D	#N/D
5	chr5:88389683:C:T	rs6452785	88389683	C	ADD	519	#N/D	#N/D	#N/D
5	chr5:88397820:G:C	rs2898558	88397820	G	ADD	519	#N/D	#N/D	#N/D
5	chr5:88401026:G:A	rs4916910	88401026	G	ADD	521	#N/D	#N/D	#N/D
5	chr5:88416702:C:A	rs7708715	88416702	C	ADD	522	#N/D	#N/D	#N/D
5	chr5:88417014:A:G	rs6452787	88417014	A	ADD	518	#N/D	#N/D	#N/D
5	chr5:88433894:A:C	rs6414946	88433894	A	ADD	503	#N/D	#N/D	#N/D
5	chr5:88445076:T:C	rs7722095	88445076	T	ADD	516	#N/D	#N/D	#N/D
5	chr5:88455935:G:A	rs7728883	88455935	G	ADD	517	#N/D	#N/D	#N/D
5	chr5:88456878:A:G	rs7448716	88456878	A	ADD	519	#N/D	#N/D	#N/D
5	chr5:88458988:C:T	rs6866315	88458988	C	ADD	517	#N/D	#N/D	#N/D
5	chr5:88461004:C:T	rs4352629	88461004	C	ADD	519	#N/D	#N/D	#N/D
5	chr5:88467651:C:A	rs10060720	88467651	C	ADD	517	#N/D	#N/D	#N/D
5	chr5:88467699:G:A	rs11952470	88467699	G	ADD	519	#N/D	#N/D	#N/D
5	chr5:88468801:G:A	rs4244212	88468801	G	ADD	516	#N/D	#N/D	#N/D
5	chr5:88469954:T:C	rs9293500	88469954	T	ADD	519	#N/D	#N/D	#N/D
5	chr5:88473876:C:T	rs6891239	88473876	C	ADD	517	#N/D	#N/D	#N/D

5	chr5:88475276:A:C	rs6452791	88475276	A	ADD	519	#N/D	#N/D	#N/D
5	chr5:88477838:A:G	rs6873449	88477838	A	ADD	519	#N/D	#N/D	#N/D
5	chr5:88477912:G:T	rs6874021	88477912	G	ADD	517	#N/D	#N/D	#N/D
5	chr5:88526854:T:A	rs2194027	88526854	A	ADD	486	#N/D	#N/D	#N/D
5	chr5:88871923:G:C	rs188582	88871923	G	ADD	470	#N/D	#N/D	#N/D
5	chr5:88872578:C:G	rs1671539	88872578	C	ADD	469	#N/D	#N/D	#N/D
5	chr5:88873138:C:A	rs797419	88873138	C	ADD	469	#N/D	#N/D	#N/D
5	chr5:88873835:A:G	rs304137	88873835	A	ADD	469	#N/D	#N/D	#N/D
5	chr5:88874249:T:C	rs304136	88874249	T	ADD	468	#N/D	#N/D	#N/D
5	chr5:88874514:T:C	rs216057	88874514	T	ADD	469	#N/D	#N/D	#N/D
5	chr5:88879434:C:G	rs188581	88879434	C	ADD	464	#N/D	#N/D	#N/D
5	chr5:88885292:T:G	rs3850651	88885292	G	ADD	496	#N/D	#N/D	#N/D
5	chr5:104554297:G:C	rs1592757	104554297	C	ADD	516	#N/D	#N/D	#N/D
5	chr5:104585069:C:A	rs254011	104585069	A	ADD	497	#N/D	#N/D	#N/D
5	chr5:104590343:C:T	rs254013	104590343	T	ADD	500	#N/D	#N/D	#N/D
5	chr5:104590741:T:A	rs445578	104590741	A	ADD	500	#N/D	#N/D	#N/D
5	chr5:104591878:C:T	rs2447838	104591878	T	ADD	499	#N/D	#N/D	#N/D
5	chr5:104596006:G:A	rs2431112	104596006	A	ADD	499	#N/D	#N/D	#N/D
5	chr5:104597772:C:T	rs2447832	104597772	T	ADD	500	#N/D	#N/D	#N/D
5	chr5:104600642:T:A	rs254035	104600642	A	ADD	500	#N/D	#N/D	#N/D
5	chr5:104608237:C:G	rs254025	104608237	G	ADD	495	#N/D	#N/D	#N/D
5	chr5:104608319:G:T	rs254024	104608319	T	ADD	494	#N/D	#N/D	#N/D
5	chr5:104609425:A:G	rs2431109	104609425	G	ADD	494	#N/D	#N/D	#N/D
5	chr5:104609477:C:T	rs2161097	104609477	T	ADD	495	#N/D	#N/D	#N/D
5	chr5:104610505:A:T	rs1363100	104610505	T	ADD	495	#N/D	#N/D	#N/D
5	chr5:104610781:C:T	rs2112163	104610781	T	ADD	496	#N/D	#N/D	#N/D
5	chr5:104611504:C:T	rs2447828	104611504	T	ADD	494	#N/D	#N/D	#N/D
5	chr5:104612344:C:T	rs2447827	104612344	T	ADD	494	#N/D	#N/D	#N/D
5	chr5:104614550:C:T	rs254020	104614550	T	ADD	493	#N/D	#N/D	#N/D
5	chr5:104619660:T:C	rs254023	104619660	C	ADD	492	#N/D	#N/D	#N/D
5	chr5:104623644:A:G	rs254045	104623644	G	ADD	492	#N/D	#N/D	#N/D
5	chr5:104629522:A:C	rs185260	104629522	C	ADD	493	#N/D	#N/D	#N/D
5	chr5:104642625:G:A	rs33817	104642625	A	ADD	496	#N/D	#N/D	#N/D
5	chr5:104676602:C:G	rs325506	104676602	C	ADD	491	#N/D	#N/D	#N/D
7	chr7:114350297:C:T	rs2049604	114350297	T	ADD	513	#N/D	#N/D	#N/D
12	chr12:89377735:T:C	rs796374	89377735	C	ADD	460	#N/D	#N/D	#N/D
12	chr12:89378126:T:C	rs704061	89378126	C	ADD	458	#N/D	#N/D	#N/D
12	chr12:89382507:T:C	rs770083	89382507	C	ADD	459	#N/D	#N/D	#N/D
20	chr20:21073855:C:T	rs6047225	21073855	C	ADD	516	#N/D	#N/D	#N/D
20	chr20:21138467:C:G	rs6047268	21138467	C	ADD	528	#N/D	#N/D	#N/D
20	chr20:21140567:A:T	rs2328607	21140567	A	ADD	528	#N/D	#N/D	#N/D
20	chr20:21141038:A:T	rs6047269	21141038	A	ADD	529	#N/D	#N/D	#N/D
20	chr20:21144231:T:C	rs4815022	21144231	T	ADD	528	#N/D	#N/D	#N/D
20	chr20:21146082:A:G	rs3762194	21146082	A	ADD	528	#N/D	#N/D	#N/D
20	chr20:21154956:G:T	rs6035790	21154956	G	ADD	528	#N/D	#N/D	#N/D
20	chr20:21155077:G:T	rs6035791	21155077	G	ADD	528	#N/D	#N/D	#N/D
20	chr20:21156917:A:G	rs6047277	21156917	A	ADD	529	#N/D	#N/D	#N/D
20	chr20:21157726:C:A	rs4815024	21157726	C	ADD	529	#N/D	#N/D	#N/D
20	chr20:21158348:G:A	rs6035792	21158348	G	ADD	529	#N/D	#N/D	#N/D
20	chr20:21158611:G:A	rs6035793	21158611	G	ADD	529	#N/D	#N/D	#N/D
20	chr20:21158741:C:G	rs6047278	21158741	C	ADD	529	#N/D	#N/D	#N/D
20	chr20:21159530:G:A	rs721785	21159530	G	ADD	529	#N/D	#N/D	#N/D

20	chr20:21159701:C:T	rs2093069	21159701	C	ADD	529	#N/D	#N/D	#N/D
20	chr20:21161712:T:C	rs6047279	21161712	T	ADD	529	#N/D	#N/D	#N/D
20	chr20:21161882:C:G	rs4815025	21161882	C	ADD	528	#N/D	#N/D	#N/D
20	chr20:21165647:A:G	rs6047281	21165647	A	ADD	525	#N/D	#N/D	#N/D
20	chr20:21165828:A:T	rs6047282	21165828	A	ADD	525	#N/D	#N/D	#N/D
20	chr20:21166199:T:C	rs2876595	21166199	T	ADD	524	#N/D	#N/D	#N/D
20	chr20:21166369:A:G	rs6047283	21166369	A	ADD	525	#N/D	#N/D	#N/D
20	chr20:21166767:A:G	rs6035796	21166767	A	ADD	525	#N/D	#N/D	#N/D
20	chr20:21167328:G:C	rs4625995	21167328	G	ADD	525	#N/D	#N/D	#N/D
20	chr20:21168043:G:T	rs4815026	21168043	G	ADD	525	#N/D	#N/D	#N/D
20	chr20:21169627:G:C	rs6132402	21169627	G	ADD	524	#N/D	#N/D	#N/D
20	chr20:21169993:T:C	rs4815028	21169993	T	ADD	524	#N/D	#N/D	#N/D
20	chr20:21170255:A:G	rs6047285	21170255	A	ADD	525	#N/D	#N/D	#N/D
20	chr20:21170684:G:A	rs6047288	21170684	G	ADD	525	#N/D	#N/D	#N/D
20	chr20:21170688:A:G	rs6035799	21170688	A	ADD	525	#N/D	#N/D	#N/D
20	chr20:21170708:C:T	rs6035800	21170708	C	ADD	525	#N/D	#N/D	#N/D
20	chr20:21170737:G:A	rs1072271	21170737	G	ADD	524	#N/D	#N/D	#N/D
20	chr20:21171212:C:T	rs2180581	21171212	C	ADD	525	#N/D	#N/D	#N/D
20	chr20:21171591:G:A	rs6082338	21171591	G	ADD	525	#N/D	#N/D	#N/D
20	chr20:21172102:G:A	rs6082339	21172102	G	ADD	525	#N/D	#N/D	#N/D
20	chr20:21172498:G:A	rs2328608	21172498	G	ADD	525	#N/D	#N/D	#N/D
20	chr20:21173053:G:A	rs6035802	21173053	G	ADD	525	#N/D	#N/D	#N/D
20	chr20:21173142:G:C	rs6035803	21173142	G	ADD	524	#N/D	#N/D	#N/D
20	chr20:21173632:G:C	rs6035804	21173632	G	ADD	525	#N/D	#N/D	#N/D
20	chr20:21173847:G:A	rs6047293	21173847	G	ADD	525	#N/D	#N/D	#N/D
20	chr20:21175529:A:G	rs910975	21175529	A	ADD	518	#N/D	#N/D	#N/D
20	chr20:21175938:T:C	rs2328609	21175938	T	ADD	517	#N/D	#N/D	#N/D
20	chr20:21176291:G:C	rs2103975	21176291	G	ADD	518	#N/D	#N/D	#N/D
20	chr20:21176320:A:G	rs2103976	21176320	A	ADD	518	#N/D	#N/D	#N/D
20	chr20:21176870:T:G	rs2093070	21176870	T	ADD	518	#N/D	#N/D	#N/D
20	chr20:21176925:T:C	rs6035808	21176925	T	ADD	518	#N/D	#N/D	#N/D
20	chr20:21177599:C:G	rs6047296	21177599	C	ADD	516	#N/D	#N/D	#N/D
20	chr20:21177731:T:G	rs2876596	21177731	T	ADD	516	#N/D	#N/D	#N/D
20	chr20:21178512:T:C	rs2145099	21178512	T	ADD	516	#N/D	#N/D	#N/D
20	chr20:21179524:A:C	rs6047299	21179524	A	ADD	517	#N/D	#N/D	#N/D
20	chr20:21180143:G:A	rs4815030	21180143	G	ADD	518	#N/D	#N/D	#N/D
20	chr20:21183159:A:G	rs2145100	21183159	A	ADD	518	#N/D	#N/D	#N/D
20	chr20:21228388:C:T	rs11697462	21228388	C	ADD	526	#N/D	#N/D	#N/D
20	chr20:21249817:C:T	rs6082358	21249817	C	ADD	499	#N/D	#N/D	#N/D
20	chr20:21260820:G:A	rs6047333	21260820	G	ADD	515	#N/D	#N/D	#N/D
20	chr20:21267478:G:A	rs910805	21267478	G	ADD	515	#N/D	#N/D	#N/D
20	chr20:21268900:C:T	rs6047339	21268900	C	ADD	516	#N/D	#N/D	#N/D
20	chr20:21277415:A:G	rs4813421	21277415	A	ADD	513	#N/D	#N/D	#N/D
20	chr20:21314871:G:T	rs4813425	21314871	G	ADD	519	#N/D	#N/D	#N/D

Supplementary Table 5.

dependent_var	independent_var	term	estimate	std.error	statistic	p.value
ADV1	chr5.104670966.A.G	independent_variable	0,117635557	0,015218988	7,729525651	5,59873E-14
ADV3	chr5.104670966.A.G	independent_variable	-0,123041476	0,016281054	-7,557341034	1,86427E-13
ADV1	chr5.104665051.G.A	independent_variable	0,115210087	0,015254559	7,552501801	1,9278E-13
ADV1	chr5.104671732.G.C	independent_variable	0,115210087	0,015254559	7,552501801	1,9278E-13
ADV1	chr5.104712889.C.G	independent_variable	0,115049646	0,015267807	7,535440188	2,14064E-13
ADV1	chr5.104698439.C.T	independent_variable	0,113737603	0,015137992	7,51338759	2,48647E-13
ADV1	chr5.104655775.C.A	independent_variable	0,114318877	0,015238958	7,501751668	2,72957E-13
ADV1	chr5.104672432.G.A	independent_variable	0,114561593	0,015285971	7,494557929	2,89857E-13
ADV1	chr5.104678081.G.A	independent_variable	0,114032543	0,015230949	7,486896931	3,03926E-13
ADV1	chr5.104707455.C.T	independent_variable	0,112588188	0,015154288	7,429460882	4,42666E-13
ADV1	chr5.104686538.C.G	independent_variable	0,112040012	0,015197334	7,372346527	6,58356E-13
ADV3	chr5.104665051.G.A	independent_variable	-0,120229704	0,016321324	-7,366418538	6,90479E-13
ADV3	chr5.104671732.G.C	independent_variable	-0,120229704	0,016321324	-7,366418538	6,90479E-13
ADV3	chr5.104712889.C.G	independent_variable	-0,120064568	0,016308118	-7,362257687	7,01656E-13
ADV3	chr5.104655775.C.A	independent_variable	-0,119223831	0,016306923	-7,311240008	1,0008E-12
ADV3	chr5.104672432.G.A	independent_variable	-0,119427401	0,016352753	-7,30319867	1,06679E-12
ADV3	chr5.104698439.C.T	independent_variable	-0,118096824	0,016195724	-7,291852151	1,12732E-12
ADV3	chr5.104678081.G.A	independent_variable	-0,118767626	0,016295889	-7,288195453	1,17461E-12
ADV1	chr5.104709685.C.A	independent_variable	0,109296265	0,015109097	7,233805254	1,60452E-12
ADV3	chr5.104707455.C.T	independent_variable	-0,116844153	0,016212955	-7,206838645	1,99472E-12
ADV3	chr5.104686538.C.G	independent_variable	-0,116829908	0,016261542	-7,184429942	2,3319E-12
ADV3	chr5.104709685.C.A	independent_variable	-0,113889524	0,016107264	-7,070693154	4,75499E-12
ADV1	chr5.104659667.A.G	independent_variable	0,107069657	0,015243085	7,024146064	6,45979E-12
ADV3	chr5.104659667.A.G	independent_variable	-0,110328925	0,016263851	-6,783690234	3,06495E-11
ADV1	chr5.104646025.T.G	independent_variable	0,107659704	0,016249961	6,625228421	8,50827E-11
ADV1	chr5.104620815.C.G	independent_variable	0,108574465	0,016395203	6,62233137	8,62255E-11
ADV1	chr5.104628884.G.A	independent_variable	0,108574465	0,016395203	6,62233137	8,62255E-11
ADV1	chr5.104636656.G.A	independent_variable	0,108574465	0,016395203	6,62233137	8,62255E-11
ADV1	chr5.104619100.T.A	independent_variable	0,10872419	0,016453273	6,608058521	9,42688E-11
ADV1	chr5.104612267.T.C	independent_variable	0,107292417	0,016430835	6,52994306	1,52933E-10
ADV3	chr5.104646025.T.G	independent_variable	-0,110817063	0,01737705	-6,377207874	3,91927E-10
ADV3	chr5.104620815.C.G	independent_variable	-0,111321672	0,017506028	-6,35904784	4,35699E-10
ADV3	chr5.104628884.G.A	independent_variable	-0,111321672	0,017506028	-6,35904784	4,35699E-10
ADV3	chr5.104636656.G.A	independent_variable	-0,111321672	0,017506028	-6,35904784	4,35699E-10
ADV3	chr5.104619100.T.A	independent_variable	-0,11017863	0,017583091	-6,266169428	7,61772E-10
ADV1	chr5.104702059.G.A	independent_variable	0,100824346	0,016176481	6,232773907	9,28554E-10
ADV3	chr5.104612267.T.C	independent_variable	-0,108939258	0,017548992	-6,207721606	1,07765E-09
ADV1	chr1.43604361.A.G	independent_variable	0,102578153	0,016571792	6,189925145	1,28093E-09
ADV1	chr5.104734216.A.G	independent_variable	0,100169631	0,016358175	6,123521019	1,75887E-09
ADV1	chr3.71450250.C.T	independent_variable	0,098752682	0,016127999	6,123058604	1,78661E-09
ADV3	chr3.71450250.C.T	independent_variable	-0,104088885	0,017163062	-6,064703513	2,51225E-09
ADV3	chr5.104702059.G.A	independent_variable	-0,10341512	0,017315543	-5,972386608	4,26102E-09
ADV1	chr5.104739429.T.C	independent_variable	0,099309962	0,016644791	5,966428957	4,50313E-09
ADV3	chr1.43604361.A.G	independent_variable	-0,105833405	0,017736904	-5,96684763	4,6649E-09
ADV1	chr5.104742532.A.C	independent_variable	0,098728409	0,016643957	5,931787102	5,48325E-09
ADV1	chr5.104746478.A.C	independent_variable	0,098728409	0,016643957	5,931787102	5,48325E-09
ADV3	chr5.104734216.A.G	independent_variable	-0,102919834	0,017450995	-5,89764837	6,49372E-09
ADV3	chr5.104739429.T.C	independent_variable	-0,102825166	0,01776517	-5,78802032	1,23611E-08
ADV3	chr5.104742532.A.C	independent_variable	-0,102301993	0,017757333	-5,761112515	1,43486E-08
ADV3	chr5.104746478.A.C	independent_variable	-0,102301993	0,017757333	-5,761112515	1,43486E-08
ADV3	chr1.43566122.G.C	independent_variable	-0,092007931	0,017126063	-5,372392456	1,16197E-07
ADV1	chr1.43569801.G.A	independent_variable	0,084872908	0,01613816	5,259144216	2,10046E-07
ADV1	chr1.43577721.G.A	independent_variable	0,083331594	0,015871809	5,250289602	2,19434E-07
ADV1	chr1.43566122.G.C	independent_variable	0,083416247	0,016120611	5,174509084	3,23926E-07
ADV3	chr5.88873835.A.G	independent_variable	-0,085675201	0,01697373	-5,047517515	6,42021E-07
ADV3	chr5.88874514.T.C	independent_variable	-0,085675201	0,01697373	-5,047517515	6,42021E-07
ADV1	chr1.43554060.C.T	independent_variable	0,082815706	0,016538943	5,007315458	7,54457E-07
ADV1	chr1.43556356.A.G	independent_variable	0,082815706	0,016538943	5,007315458	7,54457E-07
ADV3	chr1.43569801.G.A	independent_variable	-0,085680425	0,017214868	-4,977117685	8,73018E-07
ADV1	chr1.43682946.A.G	independent_variable	0,097629462	0,019626055	4,974482213	8,87398E-07
ADV3	chr5.88867954.G.A	independent_variable	-0,082885259	0,016742004	-4,950737152	1,00298E-06
ADV3	chr1.43577721.G.A	independent_variable	-0,083619109	0,016981196	-4,924217843	1,13027E-06
ADV1	chr1.43605020.A.G	independent_variable	0,080308616	0,016342759	4,914018391	1,18619E-06
ADV1	chr1.43560369.A.G	independent_variable	0,081072565	0,016524564	4,90618471	1,2356E-06
ADV3	chr5.88865600.T.C	independent_variable	-0,081200159	0,016749579	-4,847892569	1,65287E-06
ADV1	chr5.88874249.T.C	independent_variable	0,077520014	0,016040724	4,832700344	1,83024E-06
ADV1	chr5.88873835.A.G	independent_variable	0,077058483	0,016023532	4,809082352	2,04747E-06
ADV1	chr5.88874514.T.C	independent_variable	0,077058483	0,016023532	4,809082352	2,04747E-06

ADV1	chr1.43563242.G.A	independent_variable	0,078930334	0,016447043	4,799059162	2,07099E-06
ADV3	chr5.88872578.C.G	independent_variable	-0,082504878	0,017180939	-4,802117001	2,11654E-06
ADV3	chr5.88873138.C.A	independent_variable	-0,082504878	0,017180939	-4,802117001	2,11654E-06
ADV1	chr5.88867954.G.A	independent_variable	0,075414011	0,015768561	4,782554948	2,26175E-06
ADV3	chr5.88871923.G.C	independent_variable	-0,082095152	0,017149141	-4,787129041	2,27146E-06
ADV1	chr1.43686240.C.T	independent_variable	0,0942037	0,019723072	4,776319825	2,31736E-06
ADV1	chr5.88865600.T.C	independent_variable	0,075279139	0,015760167	4,776544378	2,32626E-06
ADV1	chr1.43570414.G.A	independent_variable	0,077631213	0,016295772	4,763886681	2,45165E-06
ADV1	chr1.43550344.G.A	independent_variable	0,082105929	0,01744598	4,706295107	3,26536E-06
ADV1	chr1.43705540.C.T	independent_variable	0,092677546	0,019704595	4,703346989	3,27181E-06
ADV3	chr11.28662674.G.A	independent_variable	-0,080767334	0,017186511	-4,699460848	3,32174E-06
ADV3	chr11.28664505.C.T	independent_variable	-0,080767334	0,017186511	-4,699460848	3,32174E-06
ADV3	chr11.28666219.T.C	independent_variable	-0,080767334	0,017186511	-4,699460848	3,32174E-06
ADV3	chr11.28668805.C.G	independent_variable	-0,080767334	0,017186511	-4,699460848	3,32174E-06
ADV3	chr11.28669587.A.G	independent_variable	-0,080767334	0,017186511	-4,699460848	3,32174E-06
ADV1	chr11.28662674.G.A	independent_variable	0,075072643	0,016123058	4,656228627	4,06722E-06
ADV1	chr11.28664505.C.T	independent_variable	0,075072643	0,016123058	4,656228627	4,06722E-06
ADV1	chr11.28666219.T.C	independent_variable	0,075072643	0,016123058	4,656228627	4,06722E-06
ADV1	chr11.28668805.C.G	independent_variable	0,075072643	0,016123058	4,656228627	4,06722E-06
ADV1	chr11.28669587.A.G	independent_variable	0,075072643	0,016123058	4,656228627	4,06722E-06
ADV1	chr11.28655291.A.G	independent_variable	0,074091768	0,015936164	4,649285067	4,18705E-06
ADV3	chr11.28659176.C.A	independent_variable	-0,08005694	0,017237329	-4,644393469	4,29783E-06
ADV1	chr20.21170737.G.A	independent_variable	-0,080887277	0,017519035	-4,617108101	4,90307E-06
ADV3	chr5.88864537.C.T	independent_variable	-0,077074143	0,016770353	-4,595856981	5,40644E-06
ADV3	chr11.28655291.A.G	independent_variable	-0,077970505	0,016972257	-4,593997381	5,41101E-06
ADV1	chr11.28659176.C.A	independent_variable	0,074247259	0,016172013	4,591095603	5,50141E-06
ADV3	chr1.43554060.C.T	independent_variable	-0,080705791	0,017680507	-4,564676222	6,23454E-06
ADV3	chr1.43556356.A.G	independent_variable	-0,080705791	0,017680507	-4,564676222	6,23454E-06
ADV3	chr1.43682946.A.G	independent_variable	-0,095257651	0,020954916	-4,545837845	6,79587E-06
ADV1	chr20.21169993.T.C	independent_variable	-0,078996468	0,017417101	-4,535569367	7,1305E-06
ADV1	chr20.21173142.G.C	independent_variable	-0,078996468	0,017417101	-4,535569367	7,1305E-06
ADV1	chr20.21165245.A.G	independent_variable	-0,07615359	0,016819942	-4,527577501	7,34349E-06
ADV1	chr5.88872578.C.G	independent_variable	0,073537727	0,01626261	4,521889694	7,77626E-06
ADV1	chr5.88873138.C.A	independent_variable	0,073537727	0,01626261	4,521889694	7,77626E-06
ADV1	chr5.88879434.C.G	independent_variable	0,073416576	0,016247487	4,518642056	7,91109E-06
ADV1	chr11.28648922.G.A	independent_variable	0,072718306	0,016119885	4,511093206	7,9303E-06
ADV1	chr11.28654958.T.C	independent_variable	0,072718306	0,016119885	4,511093206	7,9303E-06
ADV1	chr11.28655314.A.G	independent_variable	0,072718306	0,016119885	4,511093206	7,9303E-06
ADV1	chr11.28657323.A.G	independent_variable	0,072718306	0,016119885	4,511093206	7,9303E-06
ADV1	chr1.43900578.G.A	independent_variable	0,081471512	0,018055159	4,512367388	7,96273E-06
ADV1	chr5.88871923.G.C	independent_variable	0,073163209	0,016232201	4,507288335	8,30284E-06
ADV1	chr11.28650082.G.C	independent_variable	0,072601283	0,016142227	4,497600231	8,43399E-06
ADV1	chr20.21141038.A.T	independent_variable	-0,078584956	0,017500391	-4,490468619	8,7337E-06
ADV1	chr11.28646737.A.G	independent_variable	0,072337475	0,016134623	4,483369388	8,99484E-06
ADV3	chr5.88874249.T.C	independent_variable	-0,076680132	0,017090028	-4,486834725	9,11327E-06
ADV3	chr1.43579857.G.A	independent_variable	-0,077321141	0,017258662	-4,480135399	9,11717E-06
ADV3	chr1.43583485.G.A	independent_variable	-0,077321141	0,017258662	-4,480135399	9,11717E-06
ADV3	chr1.43585959.G.A	independent_variable	-0,077321141	0,017258662	-4,480135399	9,11717E-06
ADV3	chr1.43586100.T.C	independent_variable	-0,077321141	0,017258662	-4,480135399	9,11717E-06
ADV3	chr1.43574179.A.G	independent_variable	-0,077796015	0,017367205	-4,479478227	9,15769E-06
ADV3	chr1.43560369.A.G	independent_variable	-0,07908215	0,017663469	-4,477158616	9,25748E-06
ADV1	chr1.43596483.C.T	independent_variable	0,072608672	0,016215997	4,477595449	9,28851E-06
ADV3	chr1.43686240.C.T	independent_variable	-0,094067904	0,021018925	-4,47539079	9,35254E-06
ADV1	chr1.43586107.G.A	independent_variable	0,073117541	0,016350326	4,471931534	9,4576E-06
ADV3	chr1.43570414.G.A	independent_variable	-0,077704955	0,017378262	-4,471388292	9,50154E-06
ADV3	chr1.43605020.A.G	independent_variable	-0,078023629	0,017475809	-4,464664891	9,77593E-06
ADV1	chr1.43599477.C.T	independent_variable	0,072786993	0,016304215	4,464305283	9,78477E-06
ADV3	chr11.28648922.G.A	independent_variable	-0,076697399	0,017196657	-4,460017868	9,98974E-06
ADV3	chr11.28654958.T.C	independent_variable	-0,076697399	0,017196657	-4,460017868	9,98974E-06
ADV3	chr11.28655314.A.G	independent_variable	-0,076697399	0,017196657	-4,460017868	9,98974E-06
ADV3	chr11.28657323.A.G	independent_variable	-0,076697399	0,017196657	-4,460017868	9,98974E-06
ADV1	chr5.88864537.C.T	independent_variable	0,070498412	0,015803312	4,460989782	9,9903E-06
ADV3	chr11.28650082.G.C	independent_variable	-0,076554228	0,017220224	-4,445599897	1,0662E-05
ADV1	chr11.28626638.C.T	independent_variable	0,072924508	0,016409199	4,444123558	1,0764E-05
ADV1	chr11.28626783.A.T	independent_variable	0,072924508	0,016409199	4,444123558	1,0764E-05
ADV1	chr11.28626831.G.C	independent_variable	0,072924508	0,016409199	4,444123558	1,0764E-05
ADV1	chr1.43579857.G.A	independent_variable	0,07209354	0,016225524	4,443217929	1,0765E-05
ADV1	chr1.43583485.G.A	independent_variable	0,07209354	0,016225524	4,443217929	1,0765E-05
ADV1	chr1.43585959.G.A	independent_variable	0,07209354	0,016225524	4,443217929	1,0765E-05
ADV1	chr1.43586100.T.C	independent_variable	0,07209354	0,016225524	4,443217929	1,0765E-05
ADV3	chr11.28626638.C.T	independent_variable	-0,077763254	0,017502635	-4,442945447	1,0821E-05

ADV3	chr11.28626783.A.T	independent_variable	-0,077763254	0,017502635	-4,442945447	1,0821E-05
ADV3	chr11.28626831.G.C	independent_variable	-0,077763254	0,017502635	-4,442945447	1,0821E-05
ADV1	chr1.43574179.A.G	independent_variable	0,072505827	0,016328584	4,44042339	1,09162E-05
ADV1	chr11.28634517.A.T	independent_variable	0,071657007	0,016142753	4,438958371	1,09842E-05
ADV1	chr11.28634687.C.A	independent_variable	0,071657007	0,016142753	4,438958371	1,09842E-05
ADV1	chr11.28644991.C.T	independent_variable	0,071657007	0,016142753	4,438958371	1,09842E-05
ADV1	chr1.43600753.C.A	independent_variable	0,072524354	0,016364513	4,431806411	1,13257E-05
ADV3	chr11.28646737.A.G	independent_variable	-0,076257767	0,017210847	-4,430796771	1,13929E-05
ADV3	chr1.43563242.G.A	independent_variable	-0,077710418	0,017553898	-4,426960661	1,159E-05
ADV1	chr11.28655215.G.C	independent_variable	0,071249494	0,0161117	4,422220661	1,18339E-05
ADV1	chr1.43596488.C.T	independent_variable	0,071949934	0,016325099	4,407319899	1,26297E-05
ADV3	chr11.28655215.G.C	independent_variable	-0,075484281	0,017184902	-4,392476746	1,351E-05
ADV3	chr11.28634517.A.T	independent_variable	-0,075612316	0,017222857	-4,39023067	1,365E-05
ADV3	chr11.28634687.C.A	independent_variable	-0,075612316	0,017222857	-4,39023067	1,365E-05
ADV3	chr11.28644991.C.T	independent_variable	-0,075612316	0,017222857	-4,39023067	1,365E-05
ADV1	chr1.43584276.C.T	independent_variable	0,07169567	0,016386446	4,375303311	1,45641E-05
ADV1	chr1.43585903.A.G	independent_variable	0,07169567	0,016386446	4,375303311	1,45641E-05
ADV1	chr1.43586163.A.C	independent_variable	0,07169567	0,016386446	4,375303311	1,45641E-05
ADV3	chr1.43550344.G.A	independent_variable	-0,081278705	0,01870849	-4,34448232	1,68956E-05
ADV1	chr1.43541977.T.C	independent_variable	0,077133348	0,017756853	4,343863512	1,73977E-05
ADV3	chr20.21169993.T.C	independent_variable	0,080468327	0,018634594	4,318222636	1,88226E-05
ADV3	chr20.21173142.G.C	independent_variable	0,080468327	0,018634594	4,318222636	1,88226E-05
ADV3	chr11.28658596.T.C	independent_variable	-0,074394344	0,017233135	-4,316936181	1,8885E-05
ADV3	chr1.43705540.C.T	independent_variable	-0,090672922	0,021019726	-4,313706136	1,91661E-05
ADV1	chr1.43592796.C.T	independent_variable	0,070526119	0,016360711	4,310700174	1,93451E-05
ADV1	chr1.43593571.G.A	independent_variable	0,070526119	0,016360711	4,310700174	1,93451E-05
ADV1	chr1.43594812.G.A	independent_variable	0,070526119	0,016360711	4,310700174	1,93451E-05
ADV1	chr11.28658596.T.C	independent_variable	0,069254372	0,016170719	4,282701974	2,19246E-05
ADV1	chr20.21138467.C.G	independent_variable	-0,076295562	0,017818864	-4,281729921	2,20384E-05
ADV1	chr20.21140567.A.T	independent_variable	-0,076295562	0,017818864	-4,281729921	2,20384E-05
ADV3	chr20.21170737.G.A	independent_variable	0,080296891	0,018762253	4,279704072	2,22618E-05
ADV1	chr7.114381815.T.C	independent_variable	0,072022123	0,016840512	4,276718031	2,24597E-05
ADV1	chr7.114384998.A.G	independent_variable	0,071984557	0,01683607	4,275615101	2,25814E-05
ADV1	chr7.114571857.G.A	independent_variable	0,072635634	0,017017666	4,268248957	2,3472E-05
ADV3	chr20.21141038.A.T	independent_variable	0,079447906	0,018626878	4,265229366	2,36655E-05
ADV3	chr5.88851954.G.A	independent_variable	-0,071996483	0,016898055	-4,260637151	2,4171E-05
ADV3	chr5.88852224.C.T	independent_variable	-0,071996483	0,016898055	-4,260637151	2,4171E-05
ADV3	chr5.88853474.A.G	independent_variable	-0,071996483	0,016898055	-4,260637151	2,4171E-05
ADV3	chr5.88853509.G.A	independent_variable	-0,071996483	0,016898055	-4,260637151	2,4171E-05
ADV3	chr5.88854472.G.A	independent_variable	-0,071996483	0,016898055	-4,260637151	2,4171E-05
ADV3	chr5.88855175.T.A	independent_variable	-0,071996483	0,016898055	-4,260637151	2,4171E-05
ADV3	chr5.88856385.T.C	independent_variable	-0,071996483	0,016898055	-4,260637151	2,4171E-05
ADV3	chr5.88856757.C.A	independent_variable	-0,071996483	0,016898055	-4,260637151	2,4171E-05
ADV3	chr5.88858057.C.T	independent_variable	-0,071996483	0,016898055	-4,260637151	2,4171E-05
ADV1	chr20.21195965.C.T	independent_variable	-0,071666047	0,016817569	-4,261379612	2,43824E-05
ADV3	chr20.21165245.A.G	independent_variable	0,076327069	0,017955985	4,250787147	2,50911E-05
ADV3	chr5.88861735.T.C	independent_variable	-0,069807613	0,016440381	-4,246106725	2,56109E-05
ADV3	chr5.88879434.C.G	independent_variable	-0,072986408	0,017297568	-4,219460775	2,94767E-05
ADV3	chr7.114381815.T.C	independent_variable	-0,075552069	0,017940064	-4,211360096	2,97736E-05
ADV1	chr20.21422129.C.A	independent_variable	-0,06883312	0,01635796	-4,207928207	3,02058E-05
ADV3	chr7.114384998.A.G	independent_variable	-0,075364367	0,017938247	-4,201322808	3,10988E-05
ADV3	chr20.21422129.C.A	independent_variable	0,072859918	0,017365811	4,19559542	3,18418E-05
ADV1	chr20.21144231.T.C	independent_variable	-0,074458146	0,017748491	-4,195181688	3,19912E-05
ADV1	chr1.44009451.C.T	independent_variable	0,086368881	0,020598712	4,192926347	3,2809E-05
ADV1	chr1.43548609.T.C	independent_variable	0,074512836	0,017788591	4,188799197	3,38686E-05
ADV1	chr1.43631767.G.T	independent_variable	0,069531141	0,016652441	4,175432392	3,46611E-05
ADV1	chr20.21146082.A.G	independent_variable	-0,073136717	0,017610194	-4,153089677	3,82591E-05
ADV1	chr1.43549278.T.C	independent_variable	0,074002039	0,017809608	4,155175024	3,90515E-05
ADV3	chr1.43546066.G.A	independent_variable	-0,077298588	0,018625638	-4,150117571	3,98272E-05
ADV3	chr1.43543362.C.T	independent_variable	-0,076971134	0,018611016	-4,135783616	4,23148E-05
ADV1	chr1.43548902.A.T	independent_variable	0,072494322	0,017546011	4,131669718	4,30507E-05
ADV1	chr20.21167328.G.C	independent_variable	-0,073346534	0,017805707	-4,119271154	4,4162E-05
ADV1	chr20.21170255.A.G	independent_variable	-0,073346534	0,017805707	-4,119271154	4,4162E-05
ADV1	chr20.21170684.G.A	independent_variable	-0,073346534	0,017805707	-4,119271154	4,4162E-05
ADV1	chr20.21170688.A.G	independent_variable	-0,073346534	0,017805707	-4,119271154	4,4162E-05
ADV1	chr20.21170708.C.T	independent_variable	-0,073346534	0,017805707	-4,119271154	4,4162E-05
ADV1	chr20.21171212.C.T	independent_variable	-0,073346534	0,017805707	-4,119271154	4,4162E-05
ADV1	chr20.21171591.G.A	independent_variable	-0,073346534	0,017805707	-4,119271154	4,4162E-05
ADV1	chr20.21172102.G.A	independent_variable	-0,073346534	0,017805707	-4,119271154	4,4162E-05
ADV1	chr20.21172498.G.A	independent_variable	-0,073346534	0,017805707	-4,119271154	4,4162E-05
ADV1	chr20.21173053.G.A	independent_variable	-0,073346534	0,017805707	-4,119271154	4,4162E-05

ADV1	chr20.21173632.G.C	independent_variable	-0,073346534	0,017805707	-4,119271154	4,4162E-05
ADV1	chr20.21173847.G.A	independent_variable	-0,073346534	0,017805707	-4,119271154	4,4162E-05
ADV1	chr1.43830767.C.G	independent_variable	0,069018068	0,016756985	4,11876414	4,50236E-05
ADV1	chr4.111296654.C.T	independent_variable	0,105796569	0,02578112	4,103645207	4,70392E-05
ADV1	chr5.88851954.G.A	independent_variable	0,065371675	0,015928757	4,104003525	4,70838E-05
ADV1	chr5.88852224.C.T	independent_variable	0,065371675	0,015928757	4,104003525	4,70838E-05
ADV1	chr5.88853474.A.G	independent_variable	0,065371675	0,015928757	4,104003525	4,70838E-05
ADV1	chr5.88853509.G.A	independent_variable	0,065371675	0,015928757	4,104003525	4,70838E-05
ADV1	chr5.88854472.G.A	independent_variable	0,065371675	0,015928757	4,104003525	4,70838E-05
ADV1	chr5.88855175.T.A	independent_variable	0,065371675	0,015928757	4,104003525	4,70838E-05
ADV1	chr5.88856385.T.C	independent_variable	0,065371675	0,015928757	4,104003525	4,70838E-05
ADV1	chr5.88856757.C.A	independent_variable	0,065371675	0,015928757	4,104003525	4,70838E-05
ADV1	chr5.88858057.C.T	independent_variable	0,065371675	0,015928757	4,104003525	4,70838E-05
ADV3	chr1.43548902.A.T	independent_variable	-0,076947818	0,018739858	-4,106104666	4,79043E-05
ADV1	chr20.21166199.T.C	independent_variable	-0,072985143	0,017818259	-4,096087135	4,86837E-05
ADV1	chr20.21169627.G.C	independent_variable	-0,072985143	0,017818259	-4,096087135	4,86837E-05
ADV1	chr20.21154158.T.C	independent_variable	-0,069304992	0,016934033	-4,092645298	4,91157E-05
ADV1	chr1.43549154.A.G	independent_variable	0,072711611	0,017734182	4,100082709	4,91587E-05
ADV1	chr20.21189014.G.A	independent_variable	-0,066845923	0,016334008	-4,092438585	4,9473E-05
ADV3	chr1.43549064.C.T	independent_variable	-0,076283835	0,018615959	-4,097765591	4,95594E-05
ADV1	chr20.21175938.T.C	independent_variable	-0,067666979	0,016585827	-4,079807324	5,22085E-05
ADV1	chr20.21179524.A.C	independent_variable	-0,067666979	0,016585827	-4,079807324	5,22085E-05
ADV1	chr1.43658317.C.T	independent_variable	0,067494238	0,016552142	4,077674054	5,22992E-05
ADV1	chr1.43652735.G.A	independent_variable	0,06763216	0,01660534	4,072916216	5,3347E-05
ADV1	chr1.43550138.T.C	independent_variable	0,073372828	0,017998757	4,076549675	5,42662E-05
ADV3	chr1.43586107.G.A	independent_variable	-0,070956517	0,017449655	-4,06635639	5,48795E-05
ADV1	chr5.88861735.T.C	independent_variable	0,062847539	0,015464146	4,064080769	5,53875E-05
ADV1	chr1.43546066.G.A	independent_variable	0,070973702	0,017476174	4,061169367	5,77004E-05
ADV3	chr1.43599477.C.T	independent_variable	-0,070480361	0,017412037	-4,047795288	5,9262E-05
ADV1	chr1.43543362.C.T	independent_variable	0,07064452	0,017446029	4,049318073	6,06133E-05
ADV1	chr4.111287592.T.C	independent_variable	0,102790369	0,025515017	4,028622467	6,42642E-05
ADV3	chr20.21138467.C.G	independent_variable	0,07641663	0,018978518	4,026480358	6,49328E-05
ADV3	chr20.21140567.A.T	independent_variable	0,07641663	0,018978518	4,026480358	6,49328E-05
ADV1	chr7.114564074.T.A	independent_variable	0,068112022	0,016936395	4,021636246	6,64312E-05
ADV1	chr20.21158348.G.A	independent_variable	-0,071338706	0,017749739	-4,019141106	6,69093E-05
ADV1	chr20.21158611.G.A	independent_variable	-0,071338706	0,017749739	-4,019141106	6,69093E-05
ADV1	chr20.21158741.C.G	independent_variable	-0,071338706	0,017749739	-4,019141106	6,69093E-05
ADV1	chr20.21159530.G.A	independent_variable	-0,071338706	0,017749739	-4,019141106	6,69093E-05
ADV1	chr20.21159701.C.T	independent_variable	-0,071338706	0,017749739	-4,019141106	6,69093E-05
ADV3	chr4.111296654.C.T	independent_variable	-0,110470719	0,027492931	-4,018149972	6,70991E-05
ADV1	chr20.21165647.A.G	independent_variable	-0,071437562	0,01777741	-4,018445972	6,71693E-05
ADV1	chr20.21165828.A.T	independent_variable	-0,071437562	0,01777741	-4,018445972	6,71693E-05
ADV1	chr20.21166369.A.G	independent_variable	-0,071437562	0,01777741	-4,018445972	6,71693E-05
ADV1	chr20.21166767.A.G	independent_variable	-0,071437562	0,01777741	-4,018445972	6,71693E-05
ADV1	chr20.21168043.G.T	independent_variable	-0,071437562	0,01777741	-4,018445972	6,71693E-05
ADV1	chr1.43632835.G.A	independent_variable	0,066717731	0,016607719	4,017272374	6,71947E-05
ADV1	chr1.43549064.C.T	independent_variable	0,070219498	0,017460549	4,02160886	6,78728E-05
ADV3	chr7.114571857.G.A	independent_variable	-0,072559418	0,018123665	-4,003573223	7,16052E-05
ADV1	chr20.21161882.C.G	independent_variable	-0,071251431	0,017812921	-3,999985764	7,24064E-05
ADV3	chr1.43596488.C.T	independent_variable	-0,069674184	0,017424025	-3,998742152	7,25474E-05
ADV1	chr4.111295017.T.A	independent_variable	0,102075302	0,025576106	3,991041571	7,49807E-05
ADV1	chr4.111296367.C.T	independent_variable	0,102075302	0,025576106	3,991041571	7,49807E-05
ADV1	chr4.111306830.A.T	independent_variable	0,102075302	0,025576106	3,991041571	7,49807E-05
ADV3	chr1.43596483.C.T	independent_variable	-0,069295301	0,017361022	-3,991429876	7,51398E-05
ADV3	chr20.21456154.A.G	independent_variable	0,071736688	0,017990436	3,987490253	7,60967E-05
ADV3	chr20.21456675.T.C	independent_variable	0,071736688	0,017990436	3,987490253	7,60967E-05
ADV1	chr1.43655886.C.G	independent_variable	0,065928404	0,01653725	3,986660717	7,61754E-05
ADV3	chr1.43584276.C.T	independent_variable	-0,069628399	0,017485505	-3,982063933	7,76935E-05
ADV3	chr1.43585903.A.G	independent_variable	-0,069628399	0,017485505	-3,982063933	7,76935E-05
ADV3	chr1.43586163.A.C	independent_variable	-0,069628399	0,017485505	-3,982063933	7,76935E-05
ADV1	chr1.43656802.T.G	independent_variable	0,066169053	0,016625185	3,9800492	7,82812E-05
ADV3	chr20.21457201.T.G	independent_variable	0,071628273	0,01801391	3,976275797	7,96839E-05
ADV3	chr20.21463034.C.G	independent_variable	0,071628273	0,01801391	3,976275797	7,96839E-05
ADV3	chr20.21465660.G.T	independent_variable	0,071628273	0,01801391	3,976275797	7,96839E-05
ADV1	chr1.43674404.A.C	independent_variable	0,067087385	0,01687373	3,975847876	7,97101E-05
ADV1	chr20.21456154.A.G	independent_variable	-0,067295667	0,016938695	-3,972895624	8,07711E-05
ADV1	chr20.21456675.T.C	independent_variable	-0,067295667	0,016938695	-3,972895624	8,07711E-05
ADV1	chr1.43658195.A.G	independent_variable	-0,065175412	0,01643689	-3,965191208	8,34263E-05
ADV1	chr20.21457201.T.G	independent_variable	-0,067240909	0,016961302	-3,964371885	8,36454E-05
ADV1	chr20.21463034.C.G	independent_variable	-0,067240909	0,016961302	-3,964371885	8,36454E-05
ADV1	chr20.21465660.G.T	independent_variable	-0,067240909	0,016961302	-3,964371885	8,36454E-05

ADV1	chr20.21161712.T.C	independent_variable	-0,070359662	0,017757139	-3,962331006	8,44232E-05
ADV3	chr1.43600753.C.A	independent_variable	-0,069296152	0,017495207	-3,960864883	8,47096E-05
ADV1	chr20.21157726.C.A	independent_variable	-0,070534999	0,017806255	-3,961248447	8,47958E-05
ADV3	chr4.111287592.T.C	independent_variable	-0,107671953	0,027222676	-3,955230312	8,67932E-05
ADV1	chr7.114578527.C.T	independent_variable	0,067133788	0,016996085	3,949955926	8,90564E-05
ADV1	chr7.114583668.C.A	independent_variable	0,067133788	0,016996085	3,949955926	8,90564E-05
ADV1	chr7.114584108.A.G	independent_variable	0,067133788	0,016996085	3,949955926	8,90564E-05
ADV3	chr1.43900578.G.A	independent_variable	-0,076371702	0,019426498	-3,931315885	9,61277E-05
ADV1	chr20.21155077.G.T	independent_variable	-0,069263757	0,017639883	-3,926542983	9,76224E-05
ADV3	chr4.111295017.T.A	independent_variable	-0,107001184	0,027266639	-3,924252791	9,83712E-05
ADV3	chr4.111296367.C.T	independent_variable	-0,107001184	0,027266639	-3,924252791	9,83712E-05
ADV3	chr4.111306830.A.T	independent_variable	-0,107001184	0,027266639	-3,924252791	9,83712E-05
ADV3	chr20.21144231.T.C	independent_variable	0,074156088	0,018905232	3,922516759	9,92214E-05
ADV1	chr5.88862329.G.C	independent_variable	0,062628483	0,015975374	3,920313963	0,000100153
ADV1	chr1.43692458.C.G	independent_variable	0,066917988	0,01709255	3,915038341	0,000101937
ADV3	chr1.43592796.C.T	independent_variable	-0,068343145	0,017459363	-3,914412178	0,000102217
ADV3	chr1.43593571.G.A	independent_variable	-0,068343145	0,017459363	-3,914412178	0,000102217
ADV3	chr1.43594812.G.A	independent_variable	-0,068343145	0,017459363	-3,914412178	0,000102217
ADV3	chr20.21146082.A.G	independent_variable	0,073032931	0,018755375	3,893973351	0,000111288
ADV1	chr1.43682497.T.C	independent_variable	0,066604321	0,01713213	3,887684702	0,00011378
ADV1	chr1.43686518.G.A	independent_variable	0,066604321	0,01713213	3,887684702	0,00011378
ADV1	chr1.43687044.G.A	independent_variable	0,066604321	0,01713213	3,887684702	0,00011378
ADV1	chr1.43687948.G.C	independent_variable	0,066604321	0,01713213	3,887684702	0,00011378
ADV1	chr1.43688808.G.C	independent_variable	0,066604321	0,01713213	3,887684702	0,00011378
ADV3	chr4.111485805.C.T	independent_variable	-0,120050484	0,030883342	-3,887224483	0,000114734
ADV3	chr1.43658195.A.G	independent_variable	0,068083993	0,017524993	3,884965415	0,000115325
ADV3	chr1.43541977.T.C	independent_variable	-0,073974972	0,019027894	-3,887711943	0,000116799
ADV1	chr20.21156917.A.G	independent_variable	-0,068664723	0,017778459	-3,862242605	0,000126301
ADV3	chr20.21195965.C.T	independent_variable	0,069520945	0,017998202	3,862660533	0,000127251
ADV1	chr20.21154956.G.T	independent_variable	-0,067715021	0,017573568	-3,85323134	0,000130938
ADV3	chr20.21175938.T.C	independent_variable	0,068275065	0,017785399	3,838826725	0,000138971
ADV3	chr20.21179524.A.C	independent_variable	0,068275065	0,017785399	3,838826725	0,000138971
ADV3	chr20.21189014.G.A	independent_variable	0,066695037	0,017432449	3,825913145	0,000146122
ADV3	chr20.21428075.A.G	independent_variable	0,068299404	0,017859487	3,824264656	0,000146552
ADV3	chr20.21436041.T.G	independent_variable	0,068299404	0,017859487	3,824264656	0,000146552
ADV3	chr20.21439854.C.T	independent_variable	0,068299404	0,017859487	3,824264656	0,000146552
ADV3	chr20.21440237.T.C	independent_variable	0,068299404	0,017859487	3,824264656	0,000146552
ADV3	chr20.21444512.T.A	independent_variable	0,068299404	0,017859487	3,824264656	0,000146552
ADV3	chr20.21444955.G.C	independent_variable	0,068299404	0,017859487	3,824264656	0,000146552
ADV3	chr20.21449932.G.A	independent_variable	0,068299404	0,017859487	3,824264656	0,000146552
ADV3	chr20.21167328.G.C	independent_variable	0,072855432	0,019047629	3,824908119	0,000146576
ADV3	chr20.21170255.A.G	independent_variable	0,072855432	0,019047629	3,824908119	0,000146576
ADV3	chr20.21170684.G.A	independent_variable	0,072855432	0,019047629	3,824908119	0,000146576
ADV3	chr20.21170688.A.G	independent_variable	0,072855432	0,019047629	3,824908119	0,000146576
ADV3	chr20.21170708.C.T	independent_variable	0,072855432	0,019047629	3,824908119	0,000146576
ADV3	chr20.21171212.C.T	independent_variable	0,072855432	0,019047629	3,824908119	0,000146576
ADV3	chr20.21171591.G.A	independent_variable	0,072855432	0,019047629	3,824908119	0,000146576
ADV3	chr20.21172102.G.A	independent_variable	0,072855432	0,019047629	3,824908119	0,000146576
ADV3	chr20.21172498.G.A	independent_variable	0,072855432	0,019047629	3,824908119	0,000146576
ADV3	chr20.21173053.G.A	independent_variable	0,072855432	0,019047629	3,824908119	0,000146576
ADV3	chr20.21173632.G.C	independent_variable	0,072855432	0,019047629	3,824908119	0,000146576
ADV3	chr20.21173847.G.A	independent_variable	0,072855432	0,019047629	3,824908119	0,000146576
ADV1	chr1.43685962.T.G	independent_variable	0,065088095	0,017056723	3,815978998	0,000151333
ADV3	chr20.21169627.G.C	independent_variable	0,072436734	0,019059125	3,800632799	0,000161312
ADV3	chr20.21166199.T.C	independent_variable	0,072436717	0,019059125	3,800631837	0,000161313
ADV2	chr20.21201749.T.C	independent_variable	0,004220125	0,001110252	3,801051289	0,000161461
ADV1	chr20.21177599.C.G	independent_variable	-0,064174923	0,016931085	-3,790360993	0,000168226
ADV1	chr20.21177731.T.G	independent_variable	-0,064174923	0,016931085	-3,790360993	0,000168226
ADV1	chr20.21178512.T.C	independent_variable	-0,064174923	0,016931085	-3,790360993	0,000168226
ADV1	chr1.43616091.T.G	independent_variable	-0,062179477	0,016478661	-3,773333155	0,000179398
ADV1	chr20.21428075.A.G	independent_variable	-0,063455993	0,016831954	-3,769971871	0,000181374
ADV1	chr20.21436041.T.G	independent_variable	-0,063455993	0,016831954	-3,769971871	0,000181374
ADV1	chr20.21439854.C.T	independent_variable	-0,063455993	0,016831954	-3,769971871	0,000181374
ADV1	chr20.21440237.T.C	independent_variable	-0,063455993	0,016831954	-3,769971871	0,000181374
ADV1	chr20.21444512.T.A	independent_variable	-0,063455993	0,016831954	-3,769971871	0,000181374
ADV1	chr20.21444955.G.C	independent_variable	-0,063455993	0,016831954	-3,769971871	0,000181374
ADV1	chr20.21449932.G.A	independent_variable	-0,063455993	0,016831954	-3,769971871	0,000181374
ADV3	chr4.111582716.G.A	independent_variable	-0,119793574	0,031794959	-3,767690765	0,000184235
ADV2	chr20.21267478.G.A	independent_variable	0,00390917	0,001038605	3,763866796	0,0001866
ADV3	chr7.114564074.T.A	independent_variable	-0,067831518	0,018031032	-3,761932195	0,000187933
ADV1	chr4.111582716.G.A	independent_variable	0,111507238	0,029701846	3,754219142	0,000194143

ADV3	chr1.43631767.G.T	independent_variable	-0,066654712	0,017793584	-3,74599695	0,000198966
ADV3	chr20.21154158.T.C	independent_variable	0,067507991	0,018062252	3,737518061	0,000205506
ADV1	chr4.111485805.C.T	independent_variable	0,107949126	0,02890373	3,734781891	0,00020898
ADV1	chr20.21175529.A.G	independent_variable	-0,063040318	0,016886234	-3,733237226	0,000210019
ADV1	chr20.21176291.G.C	independent_variable	-0,063040318	0,016886234	-3,733237226	0,000210019
ADV1	chr20.21176925.T.C	independent_variable	-0,063040318	0,016886234	-3,733237226	0,000210019
ADV1	chr20.21180143.G.A	independent_variable	-0,063040318	0,016886234	-3,733237226	0,000210019
ADV1	chr20.21183159.A.G	independent_variable	-0,063040318	0,016886234	-3,733237226	0,000210019
ADV3	chr5.88862329.G.C	independent_variable	-0,063552206	0,01702783	-3,732255113	0,000210486
ADV1	chr20.21187755.C.T	independent_variable	-0,061826907	0,016587048	-3,727420784	0,000214708
ADV3	chr1.43548609.T.C	independent_variable	-0,071054993	0,019057296	-3,728492978	0,000217666
ADV3	chr1.43616091.T.G	independent_variable	0,06537191	0,017571796	3,720274721	0,000220413
ADV3	chr7.114578527.C.T	independent_variable	-0,06733073	0,018096631	-3,720622301	0,000220591
ADV3	chr7.114583668.C.A	independent_variable	-0,06733073	0,018096631	-3,720622301	0,000220591
ADV3	chr7.114584108.A.G	independent_variable	-0,06733073	0,018096631	-3,720622301	0,000220591
ADV3	chr20.21165647.A.G	independent_variable	0,070731579	0,01901699	3,719388777	0,000221252
ADV3	chr20.21165828.A.T	independent_variable	0,070731579	0,01901699	3,719388777	0,000221252
ADV3	chr20.21166369.A.G	independent_variable	0,070731579	0,01901699	3,719388777	0,000221252
ADV3	chr20.21166767.A.G	independent_variable	0,070731579	0,01901699	3,719388777	0,000221252
ADV3	chr20.21168043.G.T	independent_variable	0,070731579	0,01901699	3,719388777	0,000221252
ADV3	chr1.43652735.G.A	independent_variable	-0,065729854	0,017711496	-3,711140779	0,000227598
ADV3	chr1.43658317.C.T	independent_variable	-0,0654968	0,017655534	-3,709703731	0,000228862
ADV3	chr20.21158348.G.A	independent_variable	0,07008777	0,018952861	3,698004815	0,000240044
ADV3	chr20.21158611.G.A	independent_variable	0,07008777	0,018952861	3,698004815	0,000240044
ADV3	chr20.21158741.C.G	independent_variable	0,07008777	0,018952861	3,698004815	0,000240044
ADV3	chr20.21159530.G.A	independent_variable	0,07008777	0,018952861	3,698004815	0,000240044
ADV3	chr20.21159701.C.T	independent_variable	0,07008777	0,018952861	3,698004815	0,000240044
ADV3	chr20.21157726.C.A	independent_variable	0,070082095	0,018953539	3,697573095	0,000240442
ADV3	chr1.43549278.T.C	independent_variable	-0,070641727	0,019085218	-3,70138426	0,000241571
ADV3	chr20.21412561.A.G	independent_variable	0,065543886	0,017751981	3,692201259	0,000244745
ADV3	chr20.21161882.C.G	independent_variable	0,070203182	0,01901712	3,69157808	0,00024608
ADV3	chr1.43739423.T.C	independent_variable	-0,068765182	0,018633039	-3,690497497	0,000246742
ADV1	chr20.21412561.A.G	independent_variable	-0,06150323	0,016685238	-3,686086483	0,00025055
ADV3	chr1.43549154.A.G	independent_variable	-0,070080254	0,018997831	-3,688855444	0,000253337
ADV3	chr1.43674404.A.C	independent_variable	-0,066240304	0,018008631	-3,678253128	0,000258445
ADV1	chr1.43739423.T.C	independent_variable	0,064285032	0,017480584	3,677510539	0,000259316
ADV3	chr4.111500989.G.A	independent_variable	-0,111338157	0,030281576	-3,676762294	0,000261267
ADV3	chr1.44009451.C.T	independent_variable	-0,081270618	0,022103538	-3,676814965	0,000262894
ADV3	chr20.21154956.G.T	independent_variable	0,068654072	0,018704221	3,67051218	0,000266721
ADV1	chr1.43728863.A.G	independent_variable	0,064322454	0,017533895	3,668463581	0,000268243
ADV1	chr1.43729682.T.C	independent_variable	0,064322454	0,017533895	3,668463581	0,000268243
ADV3	chr1.43550138.T.C	independent_variable	-0,070798355	0,01927972	-3,672167149	0,000270152
ADV3	chr20.21155077.G.T	independent_variable	0,06888114	0,018785157	3,666785442	0,000270537
ADV1	chr20.21176870.T.G	independent_variable	-0,061637928	0,016808207	-3,667132813	0,000270677
ADV1	chr20.21187721.G.T	independent_variable	-0,060867721	0,016618645	-3,662616331	0,000275219
ADV1	chr20.21190532.C.T	independent_variable	-0,060867721	0,016618645	-3,662616331	0,000275219
ADV1	chr20.21194910.T.C	independent_variable	-0,060867721	0,016618645	-3,662616331	0,000275219
ADV3	chr1.43632835.G.A	independent_variable	-0,064765479	0,01772361	-3,6541924	0,000283082
ADV3	chr20.21161712.T.C	independent_variable	0,069157464	0,018959332	3,647674123	0,000290879
ADV1	chr4.111333132.A.G	independent_variable	0,088741648	0,024421344	3,633774122	0,000306769
ADV3	chr1.43656802.T.G	independent_variable	-0,064351868	0,017739544	-3,627594237	0,000313115
ADV3	chr1.43692458.C.G	independent_variable	-0,066069479	0,018232315	-3,623756961	0,000317739
ADV1	chr1.43728545.T.G	independent_variable	0,063139057	0,017445363	3,619245859	0,000323354
ADV1	chr1.43721141.A.T	independent_variable	0,062037865	0,017146224	3,618164865	0,000324462
ADV3	chr1.43655886.C.G	independent_variable	-0,0637516	0,017639161	-3,614208167	0,000329285
ADV3	chr1.43606749.A.G	independent_variable	0,065228313	0,018052468	3,613262835	0,00033046
ADV3	chr1.43682497.T.C	independent_variable	-0,065821087	0,018270063	-3,602674271	0,000344001
ADV3	chr1.43686518.G.A	independent_variable	-0,065821087	0,018270063	-3,602674271	0,000344001
ADV3	chr1.43687044.G.A	independent_variable	-0,065821087	0,018270063	-3,602674271	0,000344001
ADV3	chr1.43687948.G.C	independent_variable	-0,065821087	0,018270063	-3,602674271	0,000344001
ADV3	chr1.43688808.G.C	independent_variable	-0,065821087	0,018270063	-3,602674271	0,000344001
ADV3	chr1.43685962.T.G	independent_variable	-0,065476066	0,018179637	-3,601615779	0,000345427
ADV3	chr20.21156917.A.G	independent_variable	0,068004227	0,018923715	3,593598178	0,000356676
ADV1	chr20.21228388.C.T	independent_variable	-0,060954584	0,017030354	-3,579173129	0,00037664
ADV1	chr1.43756926.G.A	independent_variable	0,063253936	0,017696673	3,574340541	0,000383243
ADV3	chr1.43756926.G.A	independent_variable	-0,067289765	0,018855984	-3,568615991	0,00039151
ADV1	chr4.111500989.G.A	independent_variable	0,100653111	0,028336545	3,552060104	0,000417679
ADV1	chr1.43726088.C.T	independent_variable	0,061854968	0,017472038	3,540226223	0,000434366
ADV1	chr1.43731274.C.T	independent_variable	0,061854968	0,017472038	3,540226223	0,000434366
ADV1	chr1.43737320.A.G	independent_variable	0,061655585	0,017438217	3,535658848	0,000441904
ADV3	chr1.43737320.A.G	independent_variable	-0,065316212	0,018584832	-3,514490321	0,000477796

ADV1	chr20.21176320.A.G	independent_variable	-0,059445301	0,016941659	-3,508824133	0,000489355
ADV1	chr1.43729733.C.T	independent_variable	0,061382571	0,017529156	3,501741325	0,000500635
ADV1	chr1.43744582.C.G	independent_variable	0,062161437	0,017754538	3,501157734	0,000502228
ADV1	chr1.43745339.A.G	independent_variable	0,062161437	0,017754538	3,501157734	0,000502228
ADV1	chr1.43746026.T.C	independent_variable	0,062161437	0,017754538	3,501157734	0,000502228
ADV1	chr1.43747271.A.G	independent_variable	0,062161437	0,017754538	3,501157734	0,000502228
ADV1	chr1.43747507.C.A	independent_variable	0,062161437	0,017754538	3,501157734	0,000502228
ADV1	chr1.43751205.T.C	independent_variable	0,062161437	0,017754538	3,501157734	0,000502228
ADV1	chr1.43753604.T.C	independent_variable	0,062161437	0,017754538	3,501157734	0,000502228
ADV1	chr1.43763926.G.A	independent_variable	0,062161437	0,017754538	3,501157734	0,000502228
ADV3	chr7.114570759.T.C	independent_variable	-0,059054581	0,016875821	-3,499360488	0,000505931
ADV1	chr1.43720773.T.A	independent_variable	0,061593128	0,017619445	3,495747228	0,00051161
ADV3	chr20.21177599.C.G	independent_variable	0,063315927	0,018152414	3,488016987	0,00052827
ADV3	chr20.21177731.T.G	independent_variable	0,063315927	0,018152414	3,488016987	0,00052827
ADV3	chr20.21178512.T.C	independent_variable	0,063315927	0,018152414	3,488016987	0,00052827
ADV3	chr1.43830767.C.G	independent_variable	-0,062618511	0,017984786	-3,481748912	0,000544767
ADV3	chr20.21176870.T.G	independent_variable	0,062574636	0,018004546	3,47549089	0,000552795
ADV1	chr4.111309356.T.A	independent_variable	0,08193722	0,023617587	3,469330771	0,0005644
ADV1	chr1.43716573.A.G	independent_variable	0,05920185	0,017094537	3,463202993	0,00057613
ADV3	chr1.43744582.C.G	independent_variable	-0,065306599	0,018894504	-3,456380687	0,000591334
ADV3	chr1.43745339.A.G	independent_variable	-0,065306599	0,018894504	-3,456380687	0,000591334
ADV3	chr1.43746026.T.C	independent_variable	-0,065306599	0,018894504	-3,456380687	0,000591334
ADV3	chr1.43747271.A.G	independent_variable	-0,065306599	0,018894504	-3,456380687	0,000591334
ADV3	chr1.43747507.C.A	independent_variable	-0,065306599	0,018894504	-3,456380687	0,000591334
ADV3	chr1.43751205.T.C	independent_variable	-0,065306599	0,018894504	-3,456380687	0,000591334
ADV3	chr1.43753604.T.C	independent_variable	-0,065306599	0,018894504	-3,456380687	0,000591334
ADV3	chr1.43763926.G.A	independent_variable	-0,065306599	0,018894504	-3,456380687	0,000591334
ADV3	chr7.114560479.G.A	independent_variable	-0,058362836	0,016896535	-3,45413036	0,000596609
ADV1	chr1.43777612.C.T	independent_variable	0,061350671	0,017767162	3,453037232	0,000598547
ADV1	chr1.43778195.T.C	independent_variable	0,060995497	0,017705715	3,444961035	0,000616225
ADV1	chr1.43781562.C.G	independent_variable	0,060995497	0,017705715	3,444961035	0,000616225
ADV1	chr1.43718252.C.T	independent_variable	0,058828608	0,01710682	3,438897894	0,000629274
ADV1	chr7.114518899.G.A	independent_variable	0,059469323	0,017321714	3,433223851	0,00064411
ADV1	chr1.43560418.C.T	independent_variable	-0,060385569	0,017608746	-3,429294103	0,000652004
ADV1	chr20.21400134.C.A	independent_variable	-0,058434164	0,017044142	-3,428401529	0,000654286
ADV3	chr20.21175529.A.G	independent_variable	0,062040318	0,018102165	3,427231863	0,00065836
ADV3	chr20.21176291.G.C	independent_variable	0,062040318	0,018102165	3,427231863	0,00065836
ADV3	chr20.21176925.T.C	independent_variable	0,062040318	0,018102165	3,427231863	0,00065836
ADV3	chr20.21180143.G.A	independent_variable	0,062040318	0,018102165	3,427231863	0,00065836
ADV3	chr20.21183159.A.G	independent_variable	0,062040318	0,018102165	3,427231863	0,00065836
ADV1	chr1.43717869.G.T	independent_variable	0,059976445	0,017523634	3,422603254	0,000667275
ADV3	chr20.21400134.C.A	independent_variable	0,062099676	0,018184298	3,415016359	0,000686563
ADV3	chr1.43778195.T.C	independent_variable	-0,064280519	0,018837882	-3,412300726	0,000693196
ADV3	chr1.43781562.C.G	independent_variable	-0,064280519	0,018837882	-3,412300726	0,000693196
ADV3	chr1.43777612.C.T	independent_variable	-0,064495826	0,018904186	-3,411721918	0,000694732
ADV1	chr1.43707752.G.A	independent_variable	0,05793771	0,016984589	3,411192985	0,000695121
ADV1	chr1.43722794.A.C	independent_variable	0,05793771	0,016984589	3,411192985	0,000695121
ADV1	chr1.43723048.A.G	independent_variable	0,05793771	0,016984589	3,411192985	0,000695121
ADV1	chr20.21249817.C.T	independent_variable	-0,05839272	0,017144391	-3,405937223	0,000712683
ADV1	chr1.43560382.T.G	independent_variable	-0,060053891	0,017669529	-3,398726172	0,000727556
ADV1	chr1.43560509.A.C	independent_variable	-0,060053891	0,017669529	-3,398726172	0,000727556
ADV1	chr20.21227106.T.G	independent_variable	-0,055639833	0,016389604	-3,394824789	0,000736913
ADV3	chr4.111333132.A.G	independent_variable	-0,088476255	0,026062909	-3,39471906	0,000738952
ADV3	chr20.21187755.C.T	independent_variable	0,060136122	0,017716554	3,394346392	0,000740551
ADV3	chr11.28521551.G.A	independent_variable	-0,060486531	0,017836331	-3,391197973	0,000751099
ADV3	chr11.28526196.C.T	independent_variable	-0,060486531	0,017836331	-3,391197973	0,000751099
ADV3	chr11.28526682.A.C	independent_variable	-0,060486531	0,017836331	-3,391197973	0,000751099
ADV1	chr7.114470513.C.T	independent_variable	0,057091002	0,01689094	3,379977774	0,000776919
ADV3	chr7.114589084.G.A	independent_variable	-0,056994829	0,016888317	-3,374808161	0,000793195
ADV1	chr4.111311550.T.C	independent_variable	0,080885726	0,02396891	3,37461011	0,00079386
ADV1	chr4.111328737.T.G	independent_variable	0,080885726	0,02396891	3,37461011	0,00079386
ADV2	chr5.104712889.C.G	independent_variable	-0,00338103	0,00100305	-3,370749511	0,000804719
ADV1	chr4.111311007.T.C	independent_variable	0,080662295	0,023987556	3,362672485	0,000828337
ADV3	chr1.43721141.A.T	independent_variable	-0,061351083	0,018273908	-3,357304971	0,0008423
ADV3	chr7.114571392.A.G	independent_variable	-0,055267811	0,016500305	-3,349502321	0,000865779
ADV1	chr1.43574039.T.C	independent_variable	-0,058246602	0,01743898	-3,340023426	0,000897037
ADV3	chr20.21187721.G.T	independent_variable	0,059217256	0,017738723	3,338304287	0,000903308
ADV3	chr20.21190532.C.T	independent_variable	0,059217256	0,017738723	3,338304287	0,000903308
ADV3	chr20.21194910.T.C	independent_variable	0,059217256	0,017738723	3,338304287	0,000903308
ADV3	chr7.114470513.C.T	independent_variable	-0,0596642	0,017978206	-3,318695978	0,000964901
ADV3	chr11.28578333.T.A	independent_variable	-0,057708446	0,017391595	-3,318180132	0,000970773

ADV1	chr1.43616545.G.A	independent_variable	-0,056809142	0,017139844	-3,314449218	0,000979959
ADV3	chr11.28526861.T.C	independent_variable	-0,059108161	0,017866938	-3,308242388	0,00100662
ADV2	chr5.104698439.C.T	independent_variable	-0,003303664	0,001000211	-3,30296869	0,001021606
ADV2	chr5.104707455.C.T	independent_variable	-0,003303641	0,001000211	-3,302945013	0,001021691
ADV1	chr1.43560985.G.C	independent_variable	0,058169061	0,017648573	3,295963919	0,001046131
ADV3	chr1.43509093.G.T	independent_variable	-0,063920946	0,019466485	-3,283640923	0,001102046
ADV3	chr1.43510950.G.C	independent_variable	-0,063920946	0,019466485	-3,283640923	0,001102046
ADV1	chr1.43606749.A.G	independent_variable	-0,055752909	0,01700329	-3,278948346	0,001108675
ADV3	chr20.21228388.C.T	independent_variable	0,059404388	0,018182779	3,267068647	0,001157714
ADV3	chr7.114518899.G.A	independent_variable	-0,05982215	0,018411034	-3,249255341	0,001232042
ADV1	chr1.43556632.A.C	independent_variable	-0,057241841	0,017709828	-3,232207558	0,001304023
ADV3	chr5.88551455.T.A	independent_variable	-0,058891553	0,018210219	-3,233983856	0,001305255
ADV3	chr4.111309356.T.A	independent_variable	-0,081396758	0,025202322	-3,229732509	0,00131617
ADV3	chr1.43616545.G.A	independent_variable	0,058749532	0,018242739	3,220433679	0,001357126
ADV3	chr20.21176320.A.G	independent_variable	0,05834911	0,018158164	3,213381631	0,001393641
ADV3	chr1.43716573.A.G	independent_variable	-0,058510428	0,018216305	-3,211981057	0,001396763
ADV1	chr1.43558813.A.G	independent_variable	-0,05568271	0,017385722	-3,202783854	0,001440823
ADV3	chr1.43728863.A.G	independent_variable	-0,059912707	0,018706992	-3,202690654	0,001441883
ADV3	chr1.43729682.T.C	independent_variable	-0,059912707	0,018706992	-3,202690654	0,001441883
ADV1	chr11.28605957.T.C	independent_variable	0,050033808	0,015697217	3,187431686	0,001518191
ADV3	chr1.43718252.C.T	independent_variable	-0,058094282	0,018228384	-3,187023262	0,001520765
ADV3	chr1.43707752.G.A	independent_variable	-0,057349815	0,018096935	-3,169034762	0,001615915
ADV3	chr1.43722794.A.C	independent_variable	-0,057349815	0,018096935	-3,169034762	0,001615915
ADV3	chr1.43723048.A.G	independent_variable	-0,057349815	0,018096935	-3,169034762	0,001615915
ADV1	chr1.43584333.G.A	independent_variable	-0,05519085	0,017444872	-3,163729125	0,001645939
ADV1	chr1.43584356.A.C	independent_variable	-0,05519085	0,017444872	-3,163729125	0,001645939
ADV1	chr1.43586706.T.G	independent_variable	-0,05519085	0,017444872	-3,163729125	0,001645939
ADV3	chr11.28569621.A.T	independent_variable	-0,055066105	0,01740592	-3,163642333	0,001650998
ADV3	chr11.28570040.C.T	independent_variable	-0,055066105	0,01740592	-3,163642333	0,001650998
ADV3	chr1.43728545.T.G	independent_variable	-0,058767963	0,01863361	-3,153868905	0,00170117
ADV1	chr4.111251830.C.A	independent_variable	0,086378747	0,027446612	3,147155179	0,001742466
ADV3	chr20.21249817.C.T	independent_variable	0,057472047	0,018291449	3,14201716	0,001777786
ADV1	chr1.43552933.G.T	independent_variable	-0,054725587	0,017436641	-3,138539589	0,001790999
ADV2	chr5.104709685.C.A	independent_variable	-0,003047379	0,000975698	-3,123280834	0,001883662
ADV4	chr4.111485805.C.T	independent_variable	0,002859849	0,000916659	3,119861735	0,001911356
ADV1	chr11.28671984.T.G	independent_variable	0,049627402	0,015915734	3,118134618	0,001920585
ADV1	chr11.28672893.C.G	independent_variable	0,049627402	0,015915734	3,118134618	0,001920585
ADV1	chr1.43554723.T.C	independent_variable	-0,054790143	0,017583766	-3,115950323	0,001931732
ADV1	chr1.43556863.C.T	independent_variable	-0,054790143	0,017583766	-3,115950323	0,001931732
ADV3	chr4.111311550.T.C	independent_variable	-0,079693933	0,025580241	-3,115448843	0,001937051
ADV3	chr4.111328737.T.G	independent_variable	-0,079693933	0,025580241	-3,115448843	0,001937051
ADV1	chr1.43585185.T.C	independent_variable	-0,053529184	0,017218367	-3,10884209	0,001977771
ADV1	chr1.43557944.G.A	independent_variable	-0,05417513	0,017441387	-3,106125135	0,001995895
ADV2	chr5.104659667.A.G	independent_variable	-0,00304875	0,000981983	-3,104686577	0,002003952
ADV1	chr11.28609522.G.C	independent_variable	0,049105219	0,015824632	3,103087597	0,002019076
ADV1	chr11.28609701.C.A	independent_variable	0,049105219	0,015824632	3,103087597	0,002019076
ADV3	chr4.111311007.T.C	independent_variable	-0,07942275	0,025597816	-3,102715838	0,002020963
ADV1	chr1.43555785.C.G	independent_variable	-0,054912626	0,017708729	-3,100878965	0,002031339
ADV3	chr7.114605694.T.A	independent_variable	-0,054109259	0,017467656	-3,097682903	0,002059393
ADV1	chr7.114647061.T.A	independent_variable	0,054880897	0,017719537	3,097196953	0,002061834
ADV1	chr11.28615816.T.C	independent_variable	0,049088521	0,015858696	3,095369435	0,002071586
ADV3	chr20.21227106.T.G	independent_variable	0,054059584	0,017478166	3,09297799	0,002083473
ADV1	chr1.43577434.T.C	independent_variable	-0,053587107	0,017340008	-3,090373817	0,002103258
ADV3	chr1.43726088.C.T	independent_variable	-0,057522157	0,018637816	-3,086314273	0,002130683
ADV3	chr1.43731274.C.T	independent_variable	-0,057522157	0,018637816	-3,086314273	0,002130683
ADV1	chr1.43509093.G.T	independent_variable	0,056087357	0,018160192	3,088478213	0,002132258
ADV1	chr1.43510950.G.C	independent_variable	0,056087357	0,018160192	3,088478213	0,002132258
ADV1	chr11.28609187.T.G	independent_variable	0,048931935	0,015858774	3,085480379	0,002140428
ADV1	chr4.111322536.C.G	independent_variable	0,068968923	0,022387657	3,080667337	0,002174264
ADV1	chr4.111310701.T.C	independent_variable	0,068400152	0,022244859	3,074874562	0,002215948
ADV1	chr1.43592279.T.C	independent_variable	-0,053478028	0,01741786	-3,070298532	0,002246835
ADV1	chr1.43609834.G.C	independent_variable	-0,052468625	0,017091554	-3,069856844	0,002249694
ADV1	chr11.28609937.C.T	independent_variable	0,048592838	0,015840821	3,067570718	0,002270483
ADV1	chr11.28605239.G.A	independent_variable	0,048495389	0,015813101	3,066785505	0,002275911
ADV1	chr11.28606106.G.A	independent_variable	0,048495389	0,015813101	3,066785505	0,002275911
ADV1	chr11.28607002.C.A	independent_variable	0,048495389	0,015813101	3,066785505	0,002275911
ADV1	chr11.28607568.G.A	independent_variable	0,048495389	0,015813101	3,066785505	0,002275911
ADV1	chr11.28613204.C.T	independent_variable	0,048495389	0,015813101	3,066785505	0,002275911
ADV1	chr11.28613315.A.G	independent_variable	0,048495389	0,015813101	3,066785505	0,002275911
ADV1	chr11.28613316.T.C	independent_variable	0,048495389	0,015813101	3,066785505	0,002275911
ADV1	chr11.28614382.G.A	independent_variable	0,048495389	0,015813101	3,066785505	0,002275911

ADV1	chr11.28615789.A.G	independent_variable	0,048495389	0,015813101	3,066785505	0,002275911
ADV1	chr1.43637827.A.G	independent_variable	-0,052764604	0,017236637	-3,061189133	0,002313866
ADV1	chr1.43638043.G.A	independent_variable	-0,052764604	0,017236637	-3,061189133	0,002313866
ADV1	chr1.43639333.C.T	independent_variable	-0,052764604	0,017236637	-3,061189133	0,002313866
ADV1	chr1.43640277.T.C	independent_variable	-0,052764604	0,017236637	-3,061189133	0,002313866
ADV1	chr1.43640595.C.T	independent_variable	-0,052764604	0,017236637	-3,061189133	0,002313866
ADV1	chr1.43640845.C.A	independent_variable	-0,052764604	0,017236637	-3,061189133	0,002313866
ADV1	chr1.43641315.G.T	independent_variable	-0,052764604	0,017236637	-3,061189133	0,002313866
ADV1	chr1.43642285.C.T	independent_variable	-0,052764604	0,017236637	-3,061189133	0,002313866
ADV1	chr1.43642448.T.A	independent_variable	-0,052764604	0,017236637	-3,061189133	0,002313866
ADV1	chr1.43643528.A.G	independent_variable	-0,052764604	0,017236637	-3,061189133	0,002313866
ADV1	chr1.43644545.A.C	independent_variable	-0,052764604	0,017236637	-3,061189133	0,002313866
ADV1	chr1.43644818.A.G	independent_variable	-0,052764604	0,017236637	-3,061189133	0,002313866
ADV1	chr1.43645516.A.G	independent_variable	-0,052764604	0,017236637	-3,061189133	0,002313866
ADV1	chr1.43646708.T.C	independent_variable	-0,052764604	0,017236637	-3,061189133	0,002313866
ADV1	chr1.43647770.T.C	independent_variable	-0,052764604	0,017236637	-3,061189133	0,002313866
ADV1	chr1.43648528.G.T	independent_variable	-0,052764604	0,017236637	-3,061189133	0,002313866
ADV3	chr1.43560418.C.T	independent_variable	0,057439811	0,01879371	3,056331688	0,002352921
ADV1	chr7.114608796.A.G	independent_variable	0,053436173	0,017486877	3,055787007	0,002359081
ADV1	chr1.43657831.G.A	independent_variable	-0,052727048	0,017258674	-3,055104124	0,002360718
ADV3	chr1.43729733.C.T	independent_variable	-0,057100085	0,018696435	-3,054062625	0,002369415
ADV1	chr11.28521551.G.A	independent_variable	0,051420187	0,016830403	3,055196519	0,002369467
ADV1	chr11.28526196.C.T	independent_variable	0,051420187	0,016830403	3,055196519	0,002369467
ADV1	chr11.28526682.A.C	independent_variable	0,051420187	0,016830403	3,055196519	0,002369467
ADV1	chr11.28603810.A.T	independent_variable	0,048406374	0,01585914	3,052269804	0,002387305
ADV3	chr5.88497027.G.A	independent_variable	-0,057689651	0,018897668	-3,052739108	0,002391875
ADV2	chr5.104655775.C.A	independent_variable	-0,003083472	0,001011366	-3,048819619	0,002413944
ADV1	chr1.43662376.A.G	independent_variable	-0,052507232	0,017235137	-3,046522464	0,002428183
ADV1	chr11.28604117.C.T	independent_variable	0,048139523	0,0158072	3,045417496	0,002440932
ADV1	chr1.43579794.A.G	independent_variable	-0,053543784	0,017588001	-3,044335919	0,002447111
ADV1	chr5.88551455.T.A	independent_variable	0,052446903	0,017220771	3,045560732	0,002450747
ADV1	chr11.28609199.A.G	independent_variable	0,048349731	0,01588564	3,043612389	0,002456054
ADV1	chr1.43569422.A.G	independent_variable	-0,051940233	0,017105377	-3,036485641	0,002509947
ADV3	chr1.43574039.T.C	independent_variable	0,056448133	0,01860962	3,03327703	0,002538357
ADV3	chr1.43720773.T.A	independent_variable	-0,056958696	0,01879738	-3,030140073	0,002561483
ADV2	chr5.104670966.A.G	independent_variable	-0,003069801	0,001013598	-3,028619299	0,002578531
ADV2	chr5.104665051.G.A	independent_variable	-0,003069778	0,001013598	-3,028595668	0,002578729
ADV2	chr5.104671732.G.C	independent_variable	-0,003069778	0,001013598	-3,028595668	0,002578729
ADV3	chr4.111322536.C.G	independent_variable	-0,07214197	0,023855692	-3,024098821	0,002616451
ADV2	chr7.114469294.A.G	independent_variable	0,00303062	0,001003482	3,020105274	0,002649874
ADV3	chr1.43560382.T.G	independent_variable	0,056929005	0,018853081	3,019612749	0,002652011
ADV3	chr1.43560509.A.C	independent_variable	0,056929005	0,018853081	3,019612749	0,002652011
ADV1	chr7.114570759.T.C	independent_variable	0,047967304	0,015893606	3,018025137	0,002667576
ADV3	chr1.43609834.G.C	independent_variable	0,054771987	0,018187007	3,011599888	0,002720873
ADV1	chr7.114560479.G.A	independent_variable	0,047866988	0,015910136	3,008584372	0,002750402
ADV3	chr4.111310701.T.C	independent_variable	-0,071302609	0,023704761	-3,007944658	0,00275683
ADV1	chr11.28608504.A.G	independent_variable	0,047739901	0,015874082	3,007411836	0,002762324
ADV3	chr1.43637827.A.G	independent_variable	0,055131881	0,018342681	3,005661002	0,002772744
ADV3	chr1.43638043.G.A	independent_variable	0,055131881	0,018342681	3,005661002	0,002772744
ADV3	chr1.43639333.C.T	independent_variable	0,055131881	0,018342681	3,005661002	0,002772744
ADV3	chr1.43640277.T.C	independent_variable	0,055131881	0,018342681	3,005661002	0,002772744
ADV3	chr1.43640595.C.T	independent_variable	0,055131881	0,018342681	3,005661002	0,002772744
ADV3	chr1.43640845.C.A	independent_variable	0,055131881	0,018342681	3,005661002	0,002772744
ADV3	chr1.43641315.G.T	independent_variable	0,055131881	0,018342681	3,005661002	0,002772744
ADV3	chr1.43642285.C.T	independent_variable	0,055131881	0,018342681	3,005661002	0,002772744
ADV3	chr1.43642448.T.A	independent_variable	0,055131881	0,018342681	3,005661002	0,002772744
ADV3	chr1.43643528.A.G	independent_variable	0,055131881	0,018342681	3,005661002	0,002772744
ADV3	chr1.43644545.A.C	independent_variable	0,055131881	0,018342681	3,005661002	0,002772744
ADV3	chr1.43644818.A.G	independent_variable	0,055131881	0,018342681	3,005661002	0,002772744
ADV3	chr1.43645516.A.G	independent_variable	0,055131881	0,018342681	3,005661002	0,002772744
ADV3	chr1.43646708.T.C	independent_variable	0,055131881	0,018342681	3,005661002	0,002772744
ADV3	chr1.43647770.T.C	independent_variable	0,055131881	0,018342681	3,005661002	0,002772744
ADV3	chr1.43648528.G.T	independent_variable	0,055131881	0,018342681	3,005661002	0,002772744
ADV3	chr1.43657831.G.A	independent_variable	0,055039926	0,018365654	2,99689436	0,00285261
ADV1	chr1.43563682.C.G	independent_variable	0,051135608	0,017066746	2,996213056	0,002862256
ADV2	chr5.104678081.G.A	independent_variable	-0,003035011	0,001013482	-2,994636441	0,002878598
ADV3	chr11.28605957.T.C	independent_variable	-0,050025434	0,016716581	-2,992563708	0,002892482
ADV2	chr5.104686538.C.G	independent_variable	-0,003007442	0,001006032	-2,989411405	0,002926409
ADV2	chr7.114489082.G.A	independent_variable	0,002997261	0,001004228	2,984643253	0,002971367
ADV2	chr7.114498696.G.C	independent_variable	0,002997261	0,001004228	2,984643253	0,002971367
ADV2	chr7.114502195.A.G	independent_variable	0,002997261	0,001004228	2,984643253	0,002971367

ADV3	chr1.43662376.A.G	independent_variable	0,054641367	0,018339627	2,979415344	0,003017916
ADV1	chr1.43596124.C.T	independent_variable	-0,050825934	0,017069064	-2,977663767	0,003035667
ADV1	chr1.43605550.G.A	independent_variable	-0,0523542	0,017587109	-2,976850906	0,003044091
ADV1	chr1.43565822.A.G	independent_variable	-0,052596473	0,01767241	-2,976191286	0,003051291
ADV3	chr11.28671984.T.G	independent_variable	-0,050600242	0,017005901	-2,975452033	0,003061373
ADV3	chr11.28672893.C.G	independent_variable	-0,050600242	0,017005901	-2,975452033	0,003061373
ADV1	chr11.28526861.T.C	independent_variable	0,050146742	0,016858161	2,974627014	0,003075549
ADV1	chr1.43636043.A.T	independent_variable	-0,051106584	0,017211239	-2,969372788	0,003116169
ADV1	chr1.43636129.A.G	independent_variable	-0,051106584	0,017211239	-2,969372788	0,003116169
ADV1	chr1.43636586.T.C	independent_variable	-0,051106584	0,017211239	-2,969372788	0,003116169
ADV1	chr11.28619515.G.A	independent_variable	0,047521907	0,01600847	2,968547713	0,003130075
ADV1	chr11.28619698.G.A	independent_variable	0,047521907	0,01600847	2,968547713	0,003130075
ADV1	chr11.28620721.G.A	independent_variable	0,047521907	0,01600847	2,968547713	0,003130075
ADV2	chr7.114503352.C.T	independent_variable	0,002983849	0,001006393	2,964894355	0,003165778
ADV2	chr5.104672432.G.A	independent_variable	-0,003014578	0,001017373	-2,96310005	0,003185602
ADV3	chr1.43585185.T.C	independent_variable	0,054235539	0,018333779	2,95823026	0,003230445
ADV3	chr1.43717869.G.T	independent_variable	-0,055346596	0,018716572	-2,957090334	0,00324145
ADV1	chr1.43649351.A.G	independent_variable	-0,051014517	0,01727628	-2,952864619	0,003285941
ADV1	chr1.43563291.G.A	independent_variable	-0,050696989	0,017180111	-2,950911571	0,003306973
ADV1	chr1.43629437.A.G	independent_variable	-0,050960965	0,017274666	-2,950040584	0,003315377
ADV1	chr1.43631843.C.G	independent_variable	-0,050960965	0,017274666	-2,950040584	0,003315377
ADV1	chr1.43634192.G.T	independent_variable	-0,050960965	0,017274666	-2,950040584	0,003315377
ADV1	chr1.43634239.T.C	independent_variable	-0,050960965	0,017274666	-2,950040584	0,003315377
ADV4	chr4.111500989.G.A	independent_variable	0,002654259	0,000899599	2,95049156	0,003318553
ADV1	chr11.28578333.T.A	independent_variable	0,048284013	0,016367544	2,949985153	0,003323904
ADV2	chr7.114484605.C.G	independent_variable	0,002963947	0,001004987	2,949240145	0,003327567
ADV1	chr5.88497027.G.A	independent_variable	0,052757608	0,017914689	2,944935811	0,003385067
ADV3	chr4.111251830.C.A	independent_variable	-0,085623436	0,029146747	-2,937666943	0,003452487
ADV1	chr1.43685344.T.G	independent_variable	0,051498176	0,01755445	2,933625165	0,003491561
ADV1	chr4.111314599.T.C	independent_variable	0,066731851	0,022762043	2,931716285	0,003518223
ADV1	chr11.28621106.G.A	independent_variable	0,047501355	0,016207949	2,930744413	0,003533767
ADV2	chr7.114497885.A.G	independent_variable	0,002941116	0,001003989	2,929430434	0,003543492
ADV3	chr20.21074404.T.C	independent_variable	0,053117985	0,018133066	2,929343731	0,003544465
ADV1	chr1.43565986.C.T	independent_variable	-0,050319228	0,017192047	-2,926889833	0,003569288
ADV1	chr1.43689712.A.G	independent_variable	0,05142736	0,01759541	2,92277129	0,003614429
ADV3	chr7.114647061.T.A	independent_variable	-0,054772251	0,018737951	-2,923065021	0,00362091
ADV3	chr1.43649351.A.G	independent_variable	0,053625099	0,01836699	2,919645501	0,003651123
ADV3	chr1.43569422.A.G	independent_variable	0,053201267	0,018240391	2,91667354	0,003686091
ADV1	chr1.43621825.C.T	independent_variable	-0,05008351	0,017173828	-2,916269437	0,003691074
ADV3	chr1.43636043.A.T	independent_variable	0,053350345	0,018315587	2,912838397	0,003729268
ADV3	chr1.43636129.A.G	independent_variable	0,053350345	0,018315587	2,912838397	0,003729268
ADV3	chr1.43636586.T.C	independent_variable	0,053350345	0,018315587	2,912838397	0,003729268
ADV3	chr1.43596124.C.T	independent_variable	0,052902676	0,018166573	2,912088882	0,00373948
ADV3	chr11.28602326.G.T	independent_variable	-0,050793235	0,017450219	-2,910750563	0,003761742
ADV2	chr7.114479375.G.A	independent_variable	0,002927587	0,001006142	2,909715295	0,003771303
ADV3	chr4.111314599.T.C	independent_variable	-0,070543214	0,024254974	-2,908401967	0,003786923
ADV1	chr11.28617583.T.C	independent_variable	0,046740552	0,016070997	2,908379124	0,003788999
ADV3	chr11.28621014.G.C	independent_variable	-0,049333436	0,016968214	-2,907402935	0,003801263
ADV3	chr7.114627941.T.C	independent_variable	-0,0507712	0,017463351	-2,907299984	0,00380889
ADV1	chr1.43576868.C.G	independent_variable	-0,050240163	0,017287934	-2,906082483	0,003811724
ADV3	chr1.43558813.A.G	independent_variable	0,053803657	0,01852765	2,90396548	0,00383463
ADV3	chr5.88629003.C.T	independent_variable	0,05408477	0,018619898	2,904676008	0,00383942
ADV3	chr1.43572014.G.A	independent_variable	-0,053363711	0,018378884	-2,903533823	0,003842973
ADV3	chr5.88605345.G.A	independent_variable	0,054076197	0,018654599	2,898813172	0,003910048
ADV3	chr5.88618013.G.A	independent_variable	0,054076197	0,018654599	2,898813172	0,003910048
ADV3	chr5.88618446.A.G	independent_variable	0,054076197	0,018654599	2,898813172	0,003910048
ADV3	chr5.88619663.G.T	independent_variable	0,054076197	0,018654599	2,898813172	0,003910048
ADV2	chr7.114476826.G.A	independent_variable	0,00290769	0,001004729	2,894003895	0,003961766
ADV1	chr11.28620773.G.A	independent_variable	0,046321162	0,016024132	2,890712672	0,004004368
ADV1	chr11.28621014.G.C	independent_variable	0,0465624	0,01612098	2,888310842	0,004035845
ADV3	chr11.28615816.T.C	independent_variable	-0,048729542	0,016877521	-2,887245256	0,004048052
ADV3	chr1.43629437.A.G	independent_variable	0,053055735	0,018382212	2,886254096	0,00405485
ADV3	chr1.43631843.C.G	independent_variable	0,053055735	0,018382212	2,886254096	0,00405485
ADV3	chr1.43634192.G.T	independent_variable	0,053055735	0,018382212	2,886254096	0,00405485
ADV3	chr1.43634239.T.C	independent_variable	0,053055735	0,018382212	2,886254096	0,00405485
ADV3	chr11.28609522.G.C	independent_variable	-0,04849658	0,016850378	-2,87807075	0,004165416
ADV3	chr11.28609701.C.A	independent_variable	-0,04849658	0,016850378	-2,87807075	0,004165416
ADV3	chr5.88624488.C.A	independent_variable	0,053593269	0,018666613	2,871076272	0,004264277
ADV3	chr1.43641757.C.T	independent_variable	-0,052788951	0,018427014	-2,864758818	0,004335721
ADV3	chr1.43552933.G.T	independent_variable	0,053218284	0,0185905	2,862660144	0,004364713
ADV3	chr7.114608796.A.G	independent_variable	-0,053157105	0,018571183	-2,862343541	0,004373428

ADV1	chr1.43616202.A.C	independent_variable	-0,049314333	0,017249481	-2,858887967	0,004415844
ADV1	chr1.43617439.C.T	independent_variable	-0,049314333	0,017249481	-2,858887967	0,004415844
ADV1	chr1.43617836.T.C	independent_variable	-0,049314333	0,017249481	-2,858887967	0,004415844
ADV1	chr1.43617848.G.A	independent_variable	-0,049314333	0,017249481	-2,858887967	0,004415844
ADV1	chr1.43618203.A.G	independent_variable	-0,049314333	0,017249481	-2,858887967	0,004415844
ADV1	chr1.43619068.C.T	independent_variable	-0,049314333	0,017249481	-2,858887967	0,004415844
ADV1	chr1.43624269.A.G	independent_variable	-0,049314333	0,017249481	-2,858887967	0,004415844
ADV1	chr1.43625004.T.C	independent_variable	-0,049314333	0,017249481	-2,858887967	0,004415844
ADV1	chr1.43625065.C.T	independent_variable	-0,049314333	0,017249481	-2,858887967	0,004415844
ADV1	chr1.43625603.A.G	independent_variable	-0,049314333	0,017249481	-2,858887967	0,004415844
ADV3	chr11.28609187.T.G	independent_variable	-0,048276715	0,016886404	-2,858910305	0,004421608
ADV1	chr1.43572014.G.A	independent_variable	0,049295662	0,017250814	2,857584707	0,004435933
ADV1	chr7.114589084.G.A	independent_variable	0,045472437	0,015914922	2,857220297	0,004442878
ADV1	chr11.28628323.A.G	independent_variable	0,045403524	0,015897385	2,856037343	0,004459207
ADV3	chr11.28603810.A.T	independent_variable	-0,04819301	0,016878393	-2,855307941	0,004471281
ADV3	chr1.43556632.A.C	independent_variable	0,053925737	0,018892003	2,854421414	0,004478714
ADV3	chr1.43563291.G.A	independent_variable	0,052267228	0,018317617	2,853385708	0,0044928
ADV3	chr1.43577434.T.C	independent_variable	0,052693643	0,018467155	2,853370823	0,004493639
ADV3	chr11.28605239.G.A	independent_variable	-0,047990391	0,016831717	-2,851187987	0,004528043
ADV3	chr11.28606106.G.A	independent_variable	-0,047990391	0,016831717	-2,851187987	0,004528043
ADV3	chr11.28607002.C.A	independent_variable	-0,047990391	0,016831717	-2,851187987	0,004528043
ADV3	chr11.28607568.G.A	independent_variable	-0,047990391	0,016831717	-2,851187987	0,004528043
ADV3	chr11.28613204.C.T	independent_variable	-0,047990391	0,016831717	-2,851187987	0,004528043
ADV3	chr11.28613315.A.G	independent_variable	-0,047990391	0,016831717	-2,851187987	0,004528043
ADV3	chr11.28613316.T.C	independent_variable	-0,047990391	0,016831717	-2,851187987	0,004528043
ADV3	chr11.28614382.G.A	independent_variable	-0,047990391	0,016831717	-2,851187987	0,004528043
ADV3	chr11.28615789.A.G	independent_variable	-0,047990391	0,016831717	-2,851187987	0,004528043
ADV3	chr11.28609937.C.T	independent_variable	-0,048056855	0,016871632	-2,84838218	0,004568207
ADV3	chr5.104557847.T.A	independent_variable	-0,049176403	0,017270333	-2,847449678	0,004582081
ADV3	chr1.43644078.A.G	independent_variable	-0,052457297	0,018437396	-2,845157598	0,004607756
ADV1	chr1.43575295.T.G	independent_variable	-0,048842957	0,017170163	-2,844641503	0,004617035
ADV3	chr5.88600784.C.T	independent_variable	0,053962501	0,018975389	2,843815261	0,004642111
ADV1	chr4.111267775.T.C	independent_variable	0,083038373	0,029217225	2,842103339	0,004656368
ADV3	chr11.28604117.C.T	independent_variable	-0,047803225	0,016824259	-2,841327143	0,004668219
ADV1	chr1.43616041.T.G	independent_variable	-0,048934798	0,017261501	-2,834909777	0,004756218
ADV1	chr1.43620409.T.C	independent_variable	-0,048934798	0,017261501	-2,834909777	0,004756218
ADV3	chr1.43584333.G.A	independent_variable	0,052669013	0,018593637	2,832636368	0,004790315
ADV3	chr1.43584356.A.C	independent_variable	0,052669013	0,018593637	2,832636368	0,004790315
ADV3	chr1.43586706.T.G	independent_variable	0,052669013	0,018593637	2,832636368	0,004790315
ADV3	chr20.21073855.C.T	independent_variable	0,048848594	0,017254711	2,831029369	0,004821258
ADV3	chr1.43565986.C.T	independent_variable	0,051842662	0,018329101	2,828434453	0,004853029
ADV1	chr1.43504626.G.A	independent_variable	-0,056473192	0,019951486	-2,830525586	0,004856657
ADV1	chr7.114571392.A.G	independent_variable	0,043968014	0,015556379	2,826365576	0,004881399
ADV3	chr1.43621825.C.T	independent_variable	0,051660598	0,01827764	2,826437051	0,004882942
ADV3	chr4.111267775.T.C	independent_variable	-0,087737238	0,031051967	-2,825496979	0,004900814
ADV3	chr5.104550512.T.G	independent_variable	-0,048878845	0,017318061	-2,822420157	0,004949837
ADV3	chr5.104554395.C.T	independent_variable	-0,048785448	0,017287566	-2,821996309	0,004955925
ADV3	chr5.104557287.A.T	independent_variable	-0,048795845	0,017302914	-2,820094026	0,004984946
ADV1	chr20.21073855.C.T	independent_variable	-0,045648889	0,016197873	-2,818202775	0,005015036
ADV3	chr1.43616202.A.C	independent_variable	0,05165596	0,018353396	2,814517889	0,005063601
ADV3	chr1.43617439.C.T	independent_variable	0,05165596	0,018353396	2,814517889	0,005063601
ADV3	chr1.43617836.T.C	independent_variable	0,05165596	0,018353396	2,814517889	0,005063601
ADV3	chr1.43617848.G.A	independent_variable	0,05165596	0,018353396	2,814517889	0,005063601
ADV3	chr1.43618203.A.G	independent_variable	0,05165596	0,018353396	2,814517889	0,005063601
ADV3	chr1.43619068.C.T	independent_variable	0,05165596	0,018353396	2,814517889	0,005063601
ADV3	chr1.43624269.A.G	independent_variable	0,05165596	0,018353396	2,814517889	0,005063601
ADV3	chr1.43625004.T.C	independent_variable	0,05165596	0,018353396	2,814517889	0,005063601
ADV3	chr1.43625065.C.T	independent_variable	0,05165596	0,018353396	2,814517889	0,005063601
ADV3	chr1.43625603.A.G	independent_variable	0,05165596	0,018353396	2,814517889	0,005063601
ADV3	chr11.28609199.A.G	independent_variable	-0,047597112	0,016913241	-2,814192266	0,005075604
ADV2	chr7.114505470.G.A	independent_variable	0,002913098	0,001035832	2,812327301	0,005109126
ADV1	chr11.28645542.T.C	independent_variable	0,044786284	0,015952	2,807565481	0,005177074
ADV1	chr1.43623961.A.G	independent_variable	-0,048423723	0,017296215	-2,79967164	0,005299203
ADV1	chr5.104676602.C.G	independent_variable	0,047924509	0,01712	2,799328747	0,005322928
ADV3	chr1.43557944.G.A	independent_variable	0,052030253	0,018597945	2,797634504	0,00533294
ADV1	chr1.43614741.T.C	independent_variable	-0,049109334	0,017560555	-2,796570686	0,005348886
ADV3	chr11.28582122.A.G	independent_variable	-0,04681384	0,01677195	-2,7911984	0,005447043
ADV3	chr11.28585152.T.C	independent_variable	-0,04681384	0,01677195	-2,7911984	0,005447043
ADV3	chr11.28587183.C.G	independent_variable	-0,04681384	0,01677195	-2,7911984	0,005447043
ADV3	chr11.28593837.A.G	independent_variable	-0,04681384	0,01677195	-2,7911984	0,005447043
ADV3	chr11.28594152.A.G	independent_variable	-0,04681384	0,01677195	-2,7911984	0,005447043

ADV3	chr11.28594153.T.C	independent_variable	-0,04681384	0,01677195	-2,7911984	0,005447043
ADV3	chr11.28594704.T.C	independent_variable	-0,04681384	0,01677195	-2,7911984	0,005447043
ADV3	chr11.28597209.G.A	independent_variable	-0,04681384	0,01677195	-2,7911984	0,005447043
ADV3	chr1.43620409.T.C	independent_variable	0,051252783	0,018366221	2,790600387	0,005447783
ADV3	chr1.43616041.T.G	independent_variable	0,051221053	0,018364411	2,789147585	0,005471926
ADV1	chr11.28669740.C.T	independent_variable	0,044669435	0,016029071	2,78677622	0,00551698
ADV3	chr11.28585698.T.C	independent_variable	-0,04681198	0,016800513	-2,78634229	0,005528457
ADV1	chr11.28569621.A.T	independent_variable	0,045602443	0,01637655	2,784618405	0,005558268
ADV1	chr11.28570040.C.T	independent_variable	0,045602443	0,01637655	2,784618405	0,005558268
ADV2	chr20.21328174.G.A	independent_variable	0,002724863	0,000980027	2,780396005	0,00562606
ADV2	chr20.21299182.T.G	independent_variable	0,00272484	0,000980027	2,780372085	0,005626468
ADV1	chr11.28648570.G.T	independent_variable	0,044343942	0,015956882	2,778985312	0,005648291
ADV1	chr11.28648575.T.A	independent_variable	0,044343942	0,015956882	2,778985312	0,005648291
ADV1	chr1.43568097.C.A	independent_variable	-0,049101778	0,017670629	-2,778722721	0,005650564
ADV1	chr20.21074404.T.C	independent_variable	-0,047381732	0,01705326	-2,778455925	0,005657367
ADV2	chr20.21374422.T.A	independent_variable	0,002697214	0,000971148	2,777347136	0,005676419
ADV2	chr20.21375208.A.G	independent_variable	0,002697214	0,000971148	2,777347136	0,005676419
ADV3	chr1.43575295.T.G	independent_variable	0,050727594	0,018267646	2,776909179	0,005680993
ADV2	chr5.104676602.C.G	independent_variable	-0,003026659	0,001089964	-2,776843269	0,005699229
ADV3	chr11.28608504.A.G	independent_variable	-0,046915129	0,016902622	-2,775612459	0,005708248
ADV1	chr11.28666860.G.A	independent_variable	0,044390433	0,016006297	2,773310642	0,005746648
ADV1	chr11.28646269.A.G	independent_variable	0,044202121	0,015940558	2,772934417	0,005752062
ADV1	chr11.28632994.A.G	independent_variable	0,043870091	0,015832548	2,77087997	0,005786446
ADV1	chr11.28639271.A.G	independent_variable	0,043870091	0,015832548	2,77087997	0,005786446
ADV1	chr11.28628549.C.T	independent_variable	0,044133257	0,01593894	2,768895332	0,005823562
ADV1	chr1.43644078.A.G	independent_variable	0,047957353	0,017334185	2,766634479	0,005858276
ADV3	chr1.43623961.A.G	independent_variable	0,050918968	0,018407006	2,76628198	0,005864886
ADV1	chr11.28624037.T.C	independent_variable	0,043950339	0,015890199	2,76587729	0,00587617
ADV1	chr11.28628989.A.G	independent_variable	0,043950339	0,015890199	2,76587729	0,00587617
ADV1	chr11.28629363.A.G	independent_variable	0,043950339	0,015890199	2,76587729	0,00587617
ADV3	chr11.28628323.A.G	independent_variable	-0,046917899	0,016969418	-2,764850202	0,005894815
ADV2	chr20.21294655.A.C	independent_variable	0,00271274	0,000981706	2,763292082	0,005925345
ADV3	chr11.28590845.G.A	independent_variable	-0,046051215	0,016673805	-2,761890043	0,005952067
ADV3	chr11.28592943.A.T	independent_variable	-0,046051215	0,016673805	-2,761890043	0,005952067
ADV3	chr11.28596075.C.A	independent_variable	-0,046051215	0,016673805	-2,761890043	0,005952067
ADV3	chr11.28596496.C.G	independent_variable	-0,046051215	0,016673805	-2,761890043	0,005952067
ADV3	chr11.28596864.G.A	independent_variable	-0,046051215	0,016673805	-2,761890043	0,005952067
ADV3	chr1.43554723.T.C	independent_variable	0,051764813	0,018754797	2,760083898	0,005976452
ADV3	chr1.43556863.C.T	independent_variable	0,051764813	0,018754797	2,760083898	0,005976452
ADV3	chr1.43605550.G.A	independent_variable	0,05173895	0,018749186	2,75953041	0,005986814
ADV1	chr1.43641757.C.T	independent_variable	0,047788548	0,017325148	2,758334168	0,0060066
ADV3	chr5.104559401.T.G	independent_variable	-0,047833598	0,017349835	-2,757005936	0,006039559
ADV3	chr5.104556759.C.G	independent_variable	-0,047845676	0,017365738	-2,755176638	0,006072931
ADV4	chr4.111582716.G.A	independent_variable	0,002605469	0,000945876	2,754557878	0,006089643
ADV4	chr11.28655190.A.G	independent_variable	0,001366119	0,000496585	2,751029618	0,006144441
ADV4	chr11.28655717.T.C	independent_variable	0,001366119	0,000496585	2,751029618	0,006144441
ADV3	chr11.28582930.C.T	independent_variable	-0,045872404	0,016681995	-2,749815195	0,006172521
ADV3	chr1.43576868.C.G	independent_variable	0,050581624	0,018402939	2,748562371	0,006188279
ADV2	chr20.21293229.C.T	independent_variable	0,002700534	0,000983393	2,746139857	0,00623984
ADV2	chr20.21294030.G.A	independent_variable	0,002700534	0,000983393	2,746139857	0,00623984
ADV2	chr20.21299701.C.T	independent_variable	0,002687771	0,000979967	2,742714937	0,006303513
ADV3	chr11.28580674.A.G	independent_variable	-0,046008923	0,016774945	-2,742716855	0,006306787
ADV2	chr20.21268900.C.T	independent_variable	0,0027817	0,001015128	2,740243954	0,006352476
ADV1	chr11.28623283.A.G	independent_variable	0,043305487	0,015817555	2,737811746	0,006393645
ADV1	chr11.28623794.G.C	independent_variable	0,043305487	0,015817555	2,737811746	0,006393645
ADV1	chr11.28628859.C.T	independent_variable	0,043305487	0,015817555	2,737811746	0,006393645
ADV2	chr7.114464334.T.C	independent_variable	0,002830564	0,001034182	2,73700888	0,00640475
ADV3	chr1.43555785.C.G	independent_variable	0,051671981	0,018882535	2,736496006	0,006416535
ADV3	chr11.28580626.C.A	independent_variable	-0,045798478	0,016738588	-2,736101671	0,006432659
ADV3	chr1.43592279.T.C	independent_variable	0,050773826	0,01856496	2,734927934	0,00644597
ADV1	chr11.28654592.G.A	independent_variable	0,043598664	0,015968138	2,730353709	0,006538824
ADV3	chr5.88630645.G.A	independent_variable	0,051010584	0,018704821	2,727135676	0,006614572
ADV2	chr7.114474994.A.G	independent_variable	0,00280018	0,001027787	2,724475146	0,006647933
ADV3	chr5.88633148.G.T	independent_variable	0,050926667	0,018686193	2,725363455	0,006648734
ADV3	chr11.28619515.G.A	independent_variable	-0,04651021	0,017073646	-2,724093646	0,006664767
ADV3	chr11.28619698.G.A	independent_variable	-0,04651021	0,017073646	-2,724093646	0,006664767
ADV2	chr7.114435085.T.C	independent_variable	0,002892669	0,001061917	2,724007583	0,00666606
ADV2	chr7.114434202.C.T	independent_variable	0,002882491	0,001059088	2,721673257	0,006712228
ADV3	chr11.28620721.G.A	independent_variable	-0,046472092	0,017075602	-2,721549173	0,006715554
ADV3	chr5.88636991.G.T	independent_variable	0,050820993	0,018696977	2,71813956	0,006793448
ADV3	chr11.28645542.T.C	independent_variable	-0,046250726	0,017024122	-2,716775976	0,006809242

ADV2	chr3.20683444.C.T	independent_variable	-0,00265212	0,000977121	-2,71421834	0,006857198
ADV3	chr1.43621160.C.T	independent_variable	-0,04978484	0,018357305	-2,711990707	0,006900023
ADV4	chr11.28658596.T.C	independent_variable	0,001388212	0,000511939	2,711673197	0,006911784
ADV3	chr11.28621106.G.A	independent_variable	-0,046853147	0,017281395	-2,711190049	0,006931214
ADV2	chr20.21270205.T.C	independent_variable	0,002745587	0,001014474	2,706415259	0,007027079
ADV1	chr1.43853335.A.G	independent_variable	-0,047108187	0,017427844	-2,703041564	0,007087599
ADV1	chr20.21267478.G.A	independent_variable	-0,044003107	0,016283829	-2,702257915	0,007114733
ADV3	chr1.43565822.A.G	independent_variable	0,050954655	0,018863367	2,70124916	0,00712827
ADV2	chr7.114503596.T.A	independent_variable	0,002784336	0,001031653	2,698908574	0,007174602
ADV3	chr1.43579794.A.G	independent_variable	0,050573353	0,018741746	2,698433348	0,007187598
ADV2	chr3.20682712.G.A	independent_variable	-0,002639518	0,000978894	-2,696428	0,007229587
ADV3	chr5.88628064.T.G	independent_variable	0,050413443	0,018691068	2,697194345	0,007230185
ADV3	chr20.21038664.T.C	independent_variable	0,046432128	0,017230005	2,694841317	0,007271394
ADV1	chr11.28627190.A.G	independent_variable	0,042280726	0,015702453	2,692619161	0,00730989
ADV2	chr3.20680326.A.G	independent_variable	-0,002634804	0,000978926	-2,691525947	0,007335229
ADV2	chr3.20680427.C.T	independent_variable	-0,002634804	0,000978926	-2,691525947	0,007335229
ADV2	chr3.20683524.C.A	independent_variable	-0,002634804	0,000978926	-2,691525947	0,007335229
ADV2	chr7.114475213.T.C	independent_variable	0,002784419	0,001034658	2,69114903	0,007341332
ADV3	chr11.28646269.A.G	independent_variable	-0,045762833	0,017005833	-2,691008073	0,007349876
ADV2	chr7.114435631.A.C	independent_variable	0,002841817	0,001057802	2,686529842	0,007449954
ADV4	chr11.28658309.G.A	independent_variable	0,001331986	0,000495828	2,686385696	0,007450491
ADV2	chr7.114507162.C.A	independent_variable	0,002894773	0,001078629	2,683751739	0,007515356
ADV3	chr1.43560985.G.C	independent_variable	-0,050555966	0,018857655	-2,680925317	0,007569297
ADV2	chr7.114407085.A.T	independent_variable	0,002833568	0,001057238	2,680160502	0,007592564
ADV4	chr11.28630347.G.C	independent_variable	0,001322247	0,000493414	2,67978899	0,007597301
ADV3	chr11.28669740.C.T	independent_variable	-0,045833792	0,017105719	-2,679442618	0,007606833
ADV3	chr11.28628549.C.T	independent_variable	-0,045546341	0,017014366	-2,676934357	0,007662328
ADV1	chr11.28590845.G.A	independent_variable	0,042432167	0,01585792	2,675771325	0,007693153
ADV1	chr11.28592943.A.T	independent_variable	0,042432167	0,01585792	2,675771325	0,007693153
ADV1	chr11.28596075.C.A	independent_variable	0,042432167	0,01585792	2,675771325	0,007693153
ADV1	chr11.28596496.C.G	independent_variable	0,042432167	0,01585792	2,675771325	0,007693153
ADV1	chr11.28596864.G.A	independent_variable	0,042432167	0,01585792	2,675771325	0,007693153
ADV1	chr11.28582930.C.T	independent_variable	0,042432167	0,01585792	2,675771325	0,007693153
ADV3	chr11.28648570.G.T	independent_variable	-0,045551296	0,017035299	-2,673935813	0,007730229
ADV3	chr11.28648575.T.A	independent_variable	-0,045551296	0,017035299	-2,673935813	0,007730229
ADV3	chr11.28624037.T.C	independent_variable	-0,045264363	0,016962361	-2,66851792	0,007853387
ADV3	chr11.28628989.A.G	independent_variable	-0,045264363	0,016962361	-2,66851792	0,007853387
ADV3	chr11.28629363.A.G	independent_variable	-0,045264363	0,016962361	-2,66851792	0,007853387
ADV1	chr11.28631941.T.G	independent_variable	0,042033972	0,015768351	2,665717665	0,007915972
ADV1	chr11.28632083.T.C	independent_variable	0,042033972	0,015768351	2,665717665	0,007915972
ADV1	chr11.28632180.T.G	independent_variable	0,042033972	0,015768351	2,665717665	0,007915972
ADV1	chr11.28632479.C.T	independent_variable	0,042033972	0,015768351	2,665717665	0,007915972
ADV3	chr11.28632994.A.G	independent_variable	-0,045068404	0,016906727	-2,665708393	0,00791663
ADV3	chr11.28639271.A.G	independent_variable	-0,045068404	0,016906727	-2,665708393	0,00791663
ADV2	chr7.114499629.G.A	independent_variable	0,002753434	0,001033298	2,664705018	0,007936037
ADV3	chr11.28666860.G.A	independent_variable	-0,045518907	0,017081846	-2,664753448	0,007941992
ADV1	chr11.28636483.A.G	independent_variable	0,04178442	0,015694954	2,662283714	0,007991736
ADV1	chr11.28622330.G.A	independent_variable	0,04213628	0,01583155	2,661538506	0,008015374
ADV1	chr11.28622366.T.C	independent_variable	0,04213628	0,01583155	2,661538506	0,008015374
ADV1	chr11.28622395.T.C	independent_variable	0,04213628	0,01583155	2,661538506	0,008015374
ADV1	chr11.28622424.G.A	independent_variable	0,04213628	0,01583155	2,661538506	0,008015374
ADV1	chr11.28622491.T.G	independent_variable	0,04213628	0,01583155	2,661538506	0,008015374
ADV1	chr11.28622565.G.T	independent_variable	0,04213628	0,01583155	2,661538506	0,008015374
ADV1	chr11.28622745.C.A	independent_variable	0,04213628	0,01583155	2,661538506	0,008015374
ADV1	chr11.28623055.A.G	independent_variable	0,04213628	0,01583155	2,661538506	0,008015374
ADV1	chr11.28623079.A.G	independent_variable	0,04213628	0,01583155	2,661538506	0,008015374
ADV1	chr11.28623338.T.C	independent_variable	0,04213628	0,01583155	2,661538506	0,008015374
ADV1	chr11.28624151.G.A	independent_variable	0,04213628	0,01583155	2,661538506	0,008015374
ADV1	chr11.28624272.T.G	independent_variable	0,04213628	0,01583155	2,661538506	0,008015374
ADV1	chr11.28625064.T.A	independent_variable	0,04213628	0,01583155	2,661538506	0,008015374
ADV1	chr11.28625609.A.T	independent_variable	0,04213628	0,01583155	2,661538506	0,008015374
ADV1	chr11.28626408.G.T	independent_variable	0,04213628	0,01583155	2,661538506	0,008015374
ADV1	chr11.28627124.G.A	independent_variable	0,04213628	0,01583155	2,661538506	0,008015374
ADV1	chr11.28628635.A.G	independent_variable	0,04213628	0,01583155	2,661538506	0,008015374
ADV1	chr11.28629811.A.C	independent_variable	0,04213628	0,01583155	2,661538506	0,008015374
ADV2	chr3.20680838.A.C	independent_variable	-0,002604428	0,00097905	-2,660157638	0,00804474
ADV3	chr1.43617344.G.A	independent_variable	-0,049466281	0,018600483	-2,659408422	0,008061098
ADV2	chr7.114483253.C.T	independent_variable	0,002753543	0,001036348	2,656967391	0,00811805
ADV3	chr5.88587685.T.C	independent_variable	0,050962001	0,019179843	2,657060428	0,008134631
ADV1	chr11.28646124.G.A	independent_variable	0,04212369	0,015870464	2,654219087	0,0081885
ADV1	chr11.28646393.C.T	independent_variable	0,04212369	0,015870464	2,654219087	0,0081885

ADV1	chr11.28646869.T.C	independent_variable	0,04212369	0,015870464	2,654219087	0,0081885
ADV1	chr11.28648417.T.C	independent_variable	0,04212369	0,015870464	2,654219087	0,0081885
ADV1	chr11.28649222.A.G	independent_variable	0,04212369	0,015870464	2,654219087	0,0081885
ADV1	chr11.28649587.T.C	independent_variable	0,04212369	0,015870464	2,654219087	0,0081885
ADV1	chr11.28649653.C.G	independent_variable	0,04212369	0,015870464	2,654219087	0,0081885
ADV1	chr11.28650803.C.G	independent_variable	0,04212369	0,015870464	2,654219087	0,0081885
ADV1	chr11.28654623.A.G	independent_variable	0,04212369	0,015870464	2,654219087	0,0081885
ADV1	chr1.43853701.G.A	independent_variable	-0,045831907	0,017283475	-2,651776137	0,008240515
ADV1	chr11.28655190.A.G	independent_variable	0,042102686	0,015879423	2,651398963	0,008256273
ADV1	chr11.28655717.T.C	independent_variable	0,042102686	0,015879423	2,651398963	0,008256273
ADV3	chr11.28617583.T.C	independent_variable	-0,045403101	0,017146268	-2,647987401	0,008344098
ADV1	chr11.28580626.C.A	independent_variable	0,04214377	0,015919341	2,647331312	0,008362508
ADV2	chr20.21277415.A.G	independent_variable	0,002624102	0,00099167	2,646143735	0,008392012
ADV1	chr11.28580674.A.G	independent_variable	0,042141279	0,015954896	2,641275687	0,008511884
ADV3	chr11.28620773.G.A	independent_variable	-0,045137036	0,017090193	-2,641107405	0,008512146
ADV2	chr20.21390733.A.G	independent_variable	0,002567888	0,000972342	2,640929816	0,008514166
ADV4	chr11.28621106.G.A	independent_variable	0,001350201	0,000511246	2,640999171	0,008520741
ADV4	chr11.28628323.A.G	independent_variable	0,001310029	0,000496809	2,636887349	0,008614451
ADV3	chr11.28654592.G.A	independent_variable	-0,044894226	0,017041563	-2,634396075	0,008677075
ADV1	chr20.21038664.T.C	independent_variable	-0,042545112	0,016164145	-2,632067018	0,008740357
ADV3	chr5.88588020.G.C	independent_variable	0,050476623	0,019191672	2,63013162	0,008798837
ADV3	chr11.28586227.G.A	independent_variable	-0,044043809	0,01675833	-2,628174138	0,00884166
ADV1	chr1.43621160.C.T	independent_variable	0,045326897	0,017255509	2,626807283	0,008862445
ADV4	chr11.28645542.T.C	independent_variable	0,001309711	0,0004987	2,626249995	0,008884706
ADV4	chr11.28639315.G.T	independent_variable	0,001292004	0,000492014	2,625950098	0,008888641
ADV1	chr11.28622480.G.A	independent_variable	0,041614125	0,015847277	2,625948003	0,008892488
ADV2	chr20.21372841.G.C	independent_variable	0,002555792	0,000973981	2,6240682	0,008942036
ADV2	chr7.114416000.G.C	independent_variable	0,002628149	0,001002313	2,622084218	0,008993568
ADV4	chr11.28628549.C.T	independent_variable	0,001305521	0,000497903	2,62203773	0,008994291
ADV3	chr1.43504626.G.A	independent_variable	0,055699058	0,021230302	2,623564053	0,008999101
ADV2	chr20.21351811.A.G	independent_variable	0,002543479	0,000970619	2,620472209	0,009034659
ADV2	chr20.21352024.G.C	independent_variable	0,002543479	0,000970619	2,620472209	0,009034659
ADV2	chr20.21353013.G.C	independent_variable	0,002543479	0,000970619	2,620472209	0,009034659
ADV2	chr20.21353030.G.A	independent_variable	0,002543479	0,000970619	2,620472209	0,009034659
ADV2	chr20.21359219.A.G	independent_variable	0,002543479	0,000970619	2,620472209	0,009034659
ADV2	chr20.21361740.T.C	independent_variable	0,002543479	0,000970619	2,620472209	0,009034659
ADV2	chr20.21379890.A.G	independent_variable	0,002543468	0,000970618	2,620461699	0,009034934
ADV2	chr20.21388294.G.T	independent_variable	0,002543468	0,000970618	2,620461699	0,009034934
ADV2	chr20.21345460.T.C	independent_variable	0,002543451	0,000970618	2,620444183	0,009035392
ADV2	chr20.21346867.T.G	independent_variable	0,002543451	0,000970618	2,620444183	0,009035392
ADV2	chr20.21349142.T.C	independent_variable	0,002543451	0,000970618	2,620444183	0,009035392
ADV2	chr20.21350734.G.A	independent_variable	0,002543451	0,000970618	2,620444183	0,009035392
ADV1	chr14.98224586.T.A	independent_variable	-0,047020717	0,017961271	-2,617894817	0,009103257
ADV4	chr11.28624037.T.C	independent_variable	0,001298401	0,000496344	2,615927752	0,009153703
ADV4	chr11.28628989.A.G	independent_variable	0,001298401	0,000496344	2,615927752	0,009153703
ADV4	chr11.28629363.A.G	independent_variable	0,001298401	0,000496344	2,615927752	0,009153703
ADV4	chr11.28646269.A.G	independent_variable	0,001302125	0,000497962	2,614908174	0,009180713
ADV1	chr11.28658309.G.A	independent_variable	0,041543254	0,015892431	2,614027659	0,00920361
ADV2	chr7.114419101.C.T	independent_variable	0,002616424	0,001002307	2,610400184	0,009302478
ADV4	chr11.28632994.A.G	independent_variable	0,001297133	0,00049724	2,608665033	0,009345713
ADV4	chr11.28639271.A.G	independent_variable	0,001297133	0,00049724	2,608665033	0,009345713
ADV1	chr5.88605345.G.A	independent_variable	-0,045839115	0,017581149	-2,607287814	0,009398364
ADV1	chr5.88618013.G.A	independent_variable	-0,045839115	0,017581149	-2,607287814	0,009398364
ADV1	chr5.88618446.A.G	independent_variable	-0,045839115	0,017581149	-2,607287814	0,009398364
ADV1	chr5.88619663.G.T	independent_variable	-0,045839115	0,017581149	-2,607287814	0,009398364
ADV2	chr20.21318093.T.C	independent_variable	0,002557696	0,000981164	2,606797726	0,009401633
ADV2	chr20.21318094.G.T	independent_variable	0,002557696	0,000981164	2,606797726	0,009401633
ADV2	chr20.21319392.G.A	independent_variable	0,002557696	0,000981164	2,606797726	0,009401633
ADV2	chr20.21320839.C.T	independent_variable	0,002557696	0,000981164	2,606797726	0,009401633
ADV2	chr20.21321927.C.T	independent_variable	0,002557696	0,000981164	2,606797726	0,009401633
ADV2	chr20.21321943.T.C	independent_variable	0,002557696	0,000981164	2,606797726	0,009401633
ADV2	chr20.21327058.G.A	independent_variable	0,002557696	0,000981164	2,606797726	0,009401633
ADV2	chr20.21329677.C.T	independent_variable	0,002557696	0,000981164	2,606797726	0,009401633
ADV2	chr20.21350903.T.G	independent_variable	0,002552681	0,000979312	2,606607366	0,009406286
ADV1	chr11.28632740.G.A	independent_variable	0,041076941	0,015760343	2,60634808	0,009406902
ADV1	chr11.28633321.G.A	independent_variable	0,041076941	0,015760343	2,60634808	0,009406902
ADV1	chr11.28633511.A.C	independent_variable	0,041076941	0,015760343	2,60634808	0,009406902
ADV1	chr11.28634125.G.T	independent_variable	0,041076941	0,015760343	2,60634808	0,009406902
ADV1	chr11.28634351.C.G	independent_variable	0,041076941	0,015760343	2,60634808	0,009406902
ADV1	chr11.28634462.T.G	independent_variable	0,041076941	0,015760343	2,60634808	0,009406902
ADV1	chr11.28635703.A.G	independent_variable	0,041076941	0,015760343	2,60634808	0,009406902

ADV1	chr11.28637410.G.A	independent_variable	0,041076941	0,015760343	2,60634808	0,009406902
ADV1	chr11.28638055.A.T	independent_variable	0,041076941	0,015760343	2,60634808	0,009406902
ADV1	chr11.28638658.T.C	independent_variable	0,041076941	0,015760343	2,60634808	0,009406902
ADV1	chr11.28644512.C.G	independent_variable	0,041076941	0,015760343	2,60634808	0,009406902
ADV1	chr11.28644517.G.A	independent_variable	0,041076941	0,015760343	2,60634808	0,009406902
ADV1	chr11.28644793.A.C	independent_variable	0,041076941	0,015760343	2,60634808	0,009406902
ADV4	chr11.28646124.G.A	independent_variable	0,001293746	0,000496674	2,604820513	0,009450838
ADV4	chr11.28646393.C.T	independent_variable	0,001293746	0,000496674	2,604820513	0,009450838
ADV4	chr11.28646869.T.C	independent_variable	0,001293746	0,000496674	2,604820513	0,009450838
ADV4	chr11.28648417.T.C	independent_variable	0,001293746	0,000496674	2,604820513	0,009450838
ADV4	chr11.28649222.A.G	independent_variable	0,001293746	0,000496674	2,604820513	0,009450838
ADV4	chr11.28649587.T.C	independent_variable	0,001293746	0,000496674	2,604820513	0,009450838
ADV4	chr11.28649653.C.G	independent_variable	0,001293746	0,000496674	2,604820513	0,009450838
ADV4	chr11.28650803.C.G	independent_variable	0,001293746	0,000496674	2,604820513	0,009450838
ADV4	chr11.28654623.A.G	independent_variable	0,001293746	0,000496674	2,604820513	0,009450838
ADV1	chr11.28582122.A.G	independent_variable	0,041552808	0,01595482	2,604404769	0,009470282
ADV1	chr11.28585152.T.C	independent_variable	0,041552808	0,01595482	2,604404769	0,009470282
ADV1	chr11.28587183.C.G	independent_variable	0,041552808	0,01595482	2,604404769	0,009470282
ADV1	chr11.28593837.A.G	independent_variable	0,041552808	0,01595482	2,604404769	0,009470282
ADV1	chr11.28594152.A.G	independent_variable	0,041552808	0,01595482	2,604404769	0,009470282
ADV1	chr11.28594153.T.C	independent_variable	0,041552808	0,01595482	2,604404769	0,009470282
ADV1	chr11.28594704.T.C	independent_variable	0,041552808	0,01595482	2,604404769	0,009470282
ADV1	chr11.28597209.G.A	independent_variable	0,041552808	0,01595482	2,604404769	0,009470282
ADV2	chr20.21371667.C.T	independent_variable	0,002531373	0,000972254	2,603612368	0,009485767
ADV2	chr20.21373277.C.T	independent_variable	0,002531373	0,000972254	2,603612368	0,009485767
ADV2	chr20.21313246.C.T	independent_variable	0,002557128	0,000982924	2,601551445	0,009545252
ADV4	chr11.28654592.G.A	independent_variable	0,001297568	0,000499069	2,599978336	0,009584988
ADV4	chr11.28632740.G.A	independent_variable	0,001279901	0,000492339	2,599634246	0,009590528
ADV4	chr11.28633321.G.A	independent_variable	0,001279901	0,000492339	2,599634246	0,009590528
ADV4	chr11.28633511.A.C	independent_variable	0,001279901	0,000492339	2,599634246	0,009590528
ADV4	chr11.28634125.G.T	independent_variable	0,001279901	0,000492339	2,599634246	0,009590528
ADV4	chr11.28634351.C.G	independent_variable	0,001279901	0,000492339	2,599634246	0,009590528
ADV4	chr11.28634462.T.G	independent_variable	0,001279901	0,000492339	2,599634246	0,009590528
ADV4	chr11.28635703.A.G	independent_variable	0,001279901	0,000492339	2,599634246	0,009590528
ADV4	chr11.28637410.G.A	independent_variable	0,001279901	0,000492339	2,599634246	0,009590528
ADV4	chr11.28638055.A.T	independent_variable	0,001279901	0,000492339	2,599634246	0,009590528
ADV4	chr11.28638658.T.C	independent_variable	0,001279901	0,000492339	2,599634246	0,009590528
ADV4	chr11.28644512.C.G	independent_variable	0,001279901	0,000492339	2,599634246	0,009590528
ADV4	chr11.28644517.G.A	independent_variable	0,001279901	0,000492339	2,599634246	0,009590528
ADV4	chr11.28644793.A.C	independent_variable	0,001279901	0,000492339	2,599634246	0,009590528
ADV2	chr20.21372771.A.C	independent_variable	0,002538833	0,000977386	2,597573743	0,009653512
ADV1	chr11.28585698.T.C	independent_variable	0,041475016	0,015981736	2,59515083	0,009726167
ADV2	chr3.20678505.C.T	independent_variable	-0,002511171	0,00096837	-2,593195282	0,009769151
ADV2	chr7.114407487.G.C	independent_variable	0,002623729	0,001012609	2,591058221	0,009837872
ADV3	chr14.98224586.T.A	independent_variable	0,049561667	0,019128805	2,590944201	0,009837983
ADV2	chr20.21308964.A.G	independent_variable	0,002545329	0,000982834	2,589784793	0,009873335
ADV2	chr20.21315921.C.A	independent_variable	0,002545329	0,000982834	2,589784793	0,009873335
ADV2	chr7.114403104.A.G	independent_variable	0,002613504	0,001010061	2,587471173	0,009939018
ADV1	chr7.114605694.T.A	independent_variable	0,042391177	0,016397474	2,585226068	0,010011818
ADV2	chr20.21310893.C.T	independent_variable	0,002544714	0,000984604	2,584506146	0,010024292
ADV2	chr20.21311200.C.T	independent_variable	0,002544714	0,000984604	2,584506146	0,010024292
ADV2	chr20.21311590.G.C	independent_variable	0,002544714	0,000984604	2,584506146	0,010024292
ADV2	chr20.21312173.C.T	independent_variable	0,002544714	0,000984604	2,584506146	0,010024292
ADV2	chr20.21312391.G.A	independent_variable	0,002544714	0,000984604	2,584506146	0,010024292
ADV2	chr3.20687599.C.T	independent_variable	-0,002498282	0,00096806	-2,580711041	0,010121025
ADV1	chr5.88624488.C.A	independent_variable	-0,04541017	0,017594012	-2,581001492	0,010135213
ADV3	chr7.114350297.C.T	independent_variable	-0,051002484	0,019773785	-2,579298022	0,010177953
ADV2	chr20.21291449.A.T	independent_variable	0,002563598	0,000994505	2,577763256	0,010222672
ADV3	chr1.43614741.T.C	independent_variable	0,04816173	0,018701139	2,575336722	0,010279164
ADV2	chr20.21314871.G.T	independent_variable	0,002532873	0,000984513	2,57271696	0,010367678
ADV2	chr7.114451698.G.A	independent_variable	0,002659173	0,0010346	2,570241626	0,010428216
ADV1	chr11.28631449.A.G	independent_variable	0,040697768	0,015838676	2,569518319	0,010457333
ADV2	chr5.104702059.G.A	independent_variable	-0,002712509	0,001058391	-2,562862265	0,010653234
ADV4	chr11.28622480.G.A	independent_variable	0,001270322	0,00049567	2,562839495	0,010659121
ADV1	chr11.28602326.G.T	independent_variable	0,04199937	0,016398198	2,561218694	0,010714317
ADV2	chr7.114442439.T.C	independent_variable	0,002682288	0,001047966	2,559518416	0,010755505
ADV2	chr20.21283152.A.T	independent_variable	0,002538434	0,000992694	2,557115839	0,010841001
ADV2	chr20.21284820.G.A	independent_variable	0,002538434	0,000992694	2,557115839	0,010841001
ADV2	chr20.21285090.C.T	independent_variable	0,002538434	0,000992694	2,557115839	0,010841001
ADV2	chr20.21285802.C.T	independent_variable	0,002538434	0,000992694	2,557115839	0,010841001
ADV2	chr20.21286713.C.T	independent_variable	0,002538434	0,000992694	2,557115839	0,010841001

ADV2	chr20.21287631.G.A	independent_variable	0,002538434	0,000992694	2,557115839	0,010841001
ADV2	chr20.21290685.C.G	independent_variable	0,002538434	0,000992694	2,557115839	0,010841001
ADV2	chr20.21290915.A.G	independent_variable	0,002538434	0,000992694	2,557115839	0,010841001
ADV2	chr20.21290947.G.A	independent_variable	0,002538434	0,000992694	2,557115839	0,010841001
ADV2	chr20.21291136.G.T	independent_variable	0,002538434	0,000992694	2,557115839	0,010841001
ADV2	chr20.21292673.G.A	independent_variable	0,002538434	0,000992694	2,557115839	0,010841001
ADV2	chr7.114406314.C.G	independent_variable	0,002580633	0,001009734	2,55575418	0,010879627
ADV3	chr11.28623283.A.G	independent_variable	-0,043119987	0,01688668	-2,55349108	0,010944802
ADV3	chr11.28623794.G.C	independent_variable	-0,043119987	0,01688668	-2,55349108	0,010944802
ADV3	chr11.28628859.C.T	independent_variable	-0,043119987	0,01688668	-2,55349108	0,010944802
ADV4	chr11.28622330.G.A	independent_variable	0,001259903	0,000494099	2,54989892	0,011056797
ADV4	chr11.28622366.T.C	independent_variable	0,001259903	0,000494099	2,54989892	0,011056797
ADV4	chr11.28622395.T.C	independent_variable	0,001259903	0,000494099	2,54989892	0,011056797
ADV4	chr11.28622424.G.A	independent_variable	0,001259903	0,000494099	2,54989892	0,011056797
ADV4	chr11.28622491.T.G	independent_variable	0,001259903	0,000494099	2,54989892	0,011056797
ADV4	chr11.28622565.G.T	independent_variable	0,001259903	0,000494099	2,54989892	0,011056797
ADV4	chr11.28622745.C.A	independent_variable	0,001259903	0,000494099	2,54989892	0,011056797
ADV4	chr11.28623055.A.G	independent_variable	0,001259903	0,000494099	2,54989892	0,011056797
ADV4	chr11.28623079.A.G	independent_variable	0,001259903	0,000494099	2,54989892	0,011056797
ADV4	chr11.28623338.T.C	independent_variable	0,001259903	0,000494099	2,54989892	0,011056797
ADV4	chr11.28624151.G.A	independent_variable	0,001259903	0,000494099	2,54989892	0,011056797
ADV4	chr11.28624272.T.G	independent_variable	0,001259903	0,000494099	2,54989892	0,011056797
ADV4	chr11.28625064.T.A	independent_variable	0,001259903	0,000494099	2,54989892	0,011056797
ADV4	chr11.28625609.A.T	independent_variable	0,001259903	0,000494099	2,54989892	0,011056797
ADV4	chr11.28626408.G.T	independent_variable	0,001259903	0,000494099	2,54989892	0,011056797
ADV4	chr11.28627124.G.A	independent_variable	0,001259903	0,000494099	2,54989892	0,011056797
ADV4	chr11.28628635.A.G	independent_variable	0,001259903	0,000494099	2,54989892	0,011056797
ADV4	chr11.28629811.A.C	independent_variable	0,001259903	0,000494099	2,54989892	0,011056797
ADV4	chr11.28631449.A.G	independent_variable	0,001259903	0,000494099	2,54989892	0,011056797
ADV4	chr11.28648570.G.T	independent_variable	0,001276637	0,000500876	2,548807823	0,011092636
ADV4	chr11.28648575.T.A	independent_variable	0,001276637	0,000500876	2,548807823	0,011092636
ADV1	chr14.98259646.C.T	independent_variable	-0,045095773	0,017698796	-2,547957143	0,011116691
ADV3	chr14.98259646.C.T	independent_variable	0,047917965	0,018830069	2,544757859	0,011217785
ADV4	chr11.28631941.T.G	independent_variable	0,00125861	0,000494807	2,543635726	0,011252367
ADV4	chr11.28632083.T.C	independent_variable	0,00125861	0,000494807	2,543635726	0,011252367
ADV4	chr11.28632180.T.G	independent_variable	0,00125861	0,000494807	2,543635726	0,011252367
ADV4	chr11.28632479.C.T	independent_variable	0,00125861	0,000494807	2,543635726	0,011252367
ADV3	chr11.28627190.A.G	independent_variable	-0,042495739	0,016730834	-2,539965358	0,011365981
ADV2	chr20.21291893.C.T	independent_variable	0,002525887	0,00099441	2,540087492	0,011376881
ADV2	chr20.21289658.C.T	independent_variable	0,002525866	0,000994409	2,540066368	0,011377559
ADV4	chr11.28603810.A.T	independent_variable	0,001268236	0,000499703	2,53797968	0,011440189
ADV1	chr11.28586227.G.A	independent_variable	0,040438834	0,015933573	2,537964008	0,011444682
ADV4	chr11.28626638.C.T	independent_variable	0,001323105	0,00052162	2,536531162	0,011483142
ADV4	chr11.28626783.A.T	independent_variable	0,001323105	0,00052162	2,536531162	0,011483142
ADV4	chr11.28626831.G.C	independent_variable	0,001323105	0,00052162	2,536531162	0,011483142
ADV2	chr7.114440281.T.G	independent_variable	0,002656628	0,001047779	2,535484185	0,011513308
ADV3	chr14.98255846.A.C	independent_variable	0,047664351	0,018798928	2,535482398	0,011516065
ADV1	chr1.43617344.G.A	independent_variable	0,044329252	0,017490954	2,534410275	0,011546101
ADV4	chr11.28585698.T.C	independent_variable	0,001289154	0,000508588	2,534770373	0,01154879
ADV3	chr1.43853335.A.G	independent_variable	0,047037019	0,018564072	2,533766184	0,01156656
ADV1	chr11.28622320.A.G	independent_variable	0,040085614	0,015825854	2,532919453	0,011600114
ADV1	chr11.28624925.C.T	independent_variable	0,040085614	0,015825854	2,532919453	0,011600114
ADV2	chr7.114443495.C.G	independent_variable	0,002560005	0,001011327	2,531332891	0,011654853
ADV2	chr20.21281046.C.T	independent_variable	0,002511052	0,00099312	2,528448595	0,0117556
ADV1	chr7.114350297.C.T	independent_variable	0,047204333	0,018670295	2,528312112	0,0117607
ADV1	chr5.104557847.T.A	independent_variable	0,041239993	0,016341481	2,523638678	0,011912688
ADV1	chr14.98255846.A.C	independent_variable	-0,044595633	0,017672521	-2,52344498	0,011912943
ADV4	chr11.28582122.A.G	independent_variable	0,001281216	0,000507807	2,523039258	0,011935681
ADV4	chr11.28585152.T.C	independent_variable	0,001281216	0,000507807	2,523039258	0,011935681
ADV4	chr11.28587183.C.G	independent_variable	0,001281216	0,000507807	2,523039258	0,011935681
ADV4	chr11.28593837.A.G	independent_variable	0,001281216	0,000507807	2,523039258	0,011935681
ADV4	chr11.28594152.A.G	independent_variable	0,001281216	0,000507807	2,523039258	0,011935681
ADV4	chr11.28594153.T.C	independent_variable	0,001281216	0,000507807	2,523039258	0,011935681
ADV4	chr11.28594704.T.C	independent_variable	0,001281216	0,000507807	2,523039258	0,011935681
ADV4	chr11.28597209.G.A	independent_variable	0,001281216	0,000507807	2,523039258	0,011935681
ADV2	chr20.21291031.G.C	independent_variable	0,002516913	0,000997952	2,522077689	0,011969731
ADV1	chr20.21201749.T.C	independent_variable	-0,045866911	0,018190509	-2,521474844	0,011989404
ADV4	chr11.28627190.A.G	independent_variable	0,001230315	0,000488794	2,51704082	0,01212352
ADV1	chr5.88600784.C.T	independent_variable	-0,045011094	0,017878311	-2,517636808	0,012129069
ADV4	chr11.28604117.C.T	independent_variable	0,001252483	0,000498163	2,514205532	0,012229758
ADV2	chr5.104739429.T.C	independent_variable	-0,002740844	0,001090681	-2,512965989	0,012275202

ADV1	chr5.88629003.C.T	independent_variable	-0,044124194	0,017558698	-2,512953669	0,012286614
ADV2	chr20.21277855.T.C	independent_variable	0,00249822	0,000994244	2,512684156	0,012287866
ADV2	chr20.21277966.T.C	independent_variable	0,00249822	0,000994244	2,512684156	0,012287866
ADV4	chr11.28609937.C.T	independent_variable	0,001255704	0,000500009	2,511362658	0,012328649
ADV4	chr11.28615816.T.C	independent_variable	0,001255704	0,000500009	2,511362658	0,012328649
ADV5	chr7.114571392.A.G	independent_variable	0,01125912	0,004485266	2,510245606	0,012354369
ADV2	chr7.114451789.C.A	independent_variable	0,002536363	0,001010849	2,509141773	0,012403735
ADV4	chr11.28636483.A.G	independent_variable	0,00122081	0,000486612	2,508795123	0,012405151
ADV4	chr11.28609187.T.G	independent_variable	0,001254057	0,000500309	2,50656335	0,012495216
ADV3	chr20.21267478.G.A	independent_variable	0,043635686	0,017411921	2,506081076	0,012515633
ADV4	chr11.28671984.T.G	independent_variable	0,001257231	0,000501892	2,50498384	0,012549882
ADV4	chr11.28672893.C.G	independent_variable	0,001257231	0,000501892	2,50498384	0,012549882
ADV2	chr7.114436339.C.G	independent_variable	0,002534242	0,001011723	2,50487784	0,012554186
ADV2	chr20.21283529.T.C	independent_variable	0,002488228	0,000993679	2,504055056	0,012587853
ADV1	chr5.104554395.C.T	independent_variable	0,040952706	0,01635711	2,503663886	0,012597996
ADV3	chr20.21201749.T.C	independent_variable	0,048393673	0,019332348	2,503248626	0,012616828
ADV2	chr5.104742532.A.C	independent_variable	-0,002725066	0,001089405	-2,501425859	0,012676919
ADV2	chr5.104746478.A.C	independent_variable	-0,002725066	0,001089405	-2,501425859	0,012676919
ADV2	chr7.114456791.A.T	independent_variable	0,00261467	0,001045914	2,499889128	0,012719859
ADV4	chr11.28609522.G.C	independent_variable	0,001247854	0,000499239	2,49951334	0,012742928
ADV4	chr11.28609701.C.A	independent_variable	0,001247854	0,000499239	2,49951334	0,012742928
ADV2	chr7.114482451.C.T	independent_variable	0,002612562	0,001046312	2,496924922	0,012824865
ADV1	chr5.104557287.A.T	independent_variable	0,040884646	0,016371967	2,497234757	0,012825899
ADV3	chr1.43685344.T.G	independent_variable	-0,046717556	0,018717815	-2,495887258	0,012859252
ADV1	chr5.104550512.T.G	independent_variable	0,040893092	0,016385703	2,495656789	0,012882994
ADV4	chr11.28619515.G.A	independent_variable	0,00125225	0,000501803	2,495502073	0,012886745
ADV4	chr11.28619698.G.A	independent_variable	0,00125225	0,000501803	2,495502073	0,012886745
ADV4	chr11.28620721.G.A	independent_variable	0,00125225	0,000501803	2,495502073	0,012886745
ADV3	chr11.28636483.A.G	independent_variable	-0,041696197	0,016719962	-2,493797374	0,012935423
ADV3	chr1.43563682.C.G	independent_variable	-0,04549044	0,018242298	-2,493679239	0,01294817
ADV2	chr5.104734216.A.G	independent_variable	-0,002611866	0,001047681	-2,492997055	0,012964276
ADV4	chr11.28609199.A.G	independent_variable	0,001249395	0,0005014	2,491811166	0,013020392
ADV3	chr1.43689712.A.G	independent_variable	-0,046691923	0,018761561	-2,488701356	0,01312068
ADV4	chr11.28605239.G.A	independent_variable	0,001240058	0,000498472	2,487716379	0,013167619
ADV4	chr11.28606106.G.A	independent_variable	0,001240058	0,000498472	2,487716379	0,013167619
ADV4	chr11.28607002.C.A	independent_variable	0,001240058	0,000498472	2,487716379	0,013167619
ADV4	chr11.28607568.G.A	independent_variable	0,001240058	0,000498472	2,487716379	0,013167619
ADV4	chr11.28613204.C.T	independent_variable	0,001240058	0,000498472	2,487716379	0,013167619
ADV4	chr11.28613315.A.G	independent_variable	0,001240058	0,000498472	2,487716379	0,013167619
ADV4	chr11.28613316.T.C	independent_variable	0,001240058	0,000498472	2,487716379	0,013167619
ADV4	chr11.28614382.G.A	independent_variable	0,001240058	0,000498472	2,487716379	0,013167619
ADV4	chr11.28615789.A.G	independent_variable	0,001240058	0,000498472	2,487716379	0,013167619
ADV4	chr11.28617583.T.C	independent_variable	0,001253684	0,000504005	2,487443089	0,013180032
ADV3	chr14.98244157.G.A	independent_variable	0,046597135	0,018749718	2,485217863	0,013255142
ADV3	chr14.98253981.T.A	independent_variable	0,046597135	0,018749718	2,485217863	0,013255142
ADV1	chr1.43510486.G.A	independent_variable	-0,050294075	0,02022778	-2,486386235	0,013266461
ADV3	chr11.28622330.G.A	independent_variable	-0,041969335	0,016900712	-2,48328795	0,013326913
ADV3	chr11.28622366.T.C	independent_variable	-0,041969335	0,016900712	-2,48328795	0,013326913
ADV3	chr11.28622395.T.C	independent_variable	-0,041969335	0,016900712	-2,48328795	0,013326913
ADV3	chr11.28622424.G.A	independent_variable	-0,041969335	0,016900712	-2,48328795	0,013326913
ADV3	chr11.28622491.T.G	independent_variable	-0,041969335	0,016900712	-2,48328795	0,013326913
ADV3	chr11.28622565.G.T	independent_variable	-0,041969335	0,016900712	-2,48328795	0,013326913
ADV3	chr11.28622745.C.A	independent_variable	-0,041969335	0,016900712	-2,48328795	0,013326913
ADV3	chr11.28623055.A.G	independent_variable	-0,041969335	0,016900712	-2,48328795	0,013326913
ADV3	chr11.28623079.A.G	independent_variable	-0,041969335	0,016900712	-2,48328795	0,013326913
ADV3	chr11.28623338.T.C	independent_variable	-0,041969335	0,016900712	-2,48328795	0,013326913
ADV3	chr11.28624151.G.A	independent_variable	-0,041969335	0,016900712	-2,48328795	0,013326913
ADV3	chr11.28624272.T.G	independent_variable	-0,041969335	0,016900712	-2,48328795	0,013326913
ADV3	chr11.28625064.T.A	independent_variable	-0,041969335	0,016900712	-2,48328795	0,013326913
ADV3	chr11.28625609.A.T	independent_variable	-0,041969335	0,016900712	-2,48328795	0,013326913
ADV3	chr11.28626408.G.T	independent_variable	-0,041969335	0,016900712	-2,48328795	0,013326913
ADV3	chr11.28627124.G.A	independent_variable	-0,041969335	0,016900712	-2,48328795	0,013326913
ADV3	chr11.28628635.A.G	independent_variable	-0,041969335	0,016900712	-2,48328795	0,013326913
ADV3	chr11.28629811.A.C	independent_variable	-0,041969335	0,016900712	-2,48328795	0,013326913
ADV4	chr11.28580626.C.A	independent_variable	0,001257967	0,00050654	2,483453146	0,01332989
ADV4	chr11.28620834.C.T	independent_variable	0,001238116	0,000498653	2,482921261	0,01334526
ADV1	chr14.98244157.G.A	independent_variable	-0,043717162	0,017610446	-2,482456267	0,013357107
ADV1	chr14.98253981.T.A	independent_variable	-0,043717162	0,017610446	-2,482456267	0,013357107
ADV3	chr11.28655190.A.G	independent_variable	-0,042035085	0,016944267	-2,480785073	0,013419149
ADV3	chr11.28655717.T.C	independent_variable	-0,042035085	0,016944267	-2,480785073	0,013419149
ADV3	chr11.28631941.T.G	independent_variable	-0,04175946	0,016839052	-2,479917575	0,013449698

ADV3	chr11.28632083.T.C	independent_variable	-0,04175946	0,016839052	-2,479917575	0,013449698
ADV3	chr11.28632180.T.G	independent_variable	-0,04175946	0,016839052	-2,479917575	0,013449698
ADV3	chr11.28632479.C.T	independent_variable	-0,04175946	0,016839052	-2,479917575	0,013449698
ADV4	chr11.28608504.A.G	independent_variable	0,001241598	0,00050063	2,480069775	0,013451172
ADV4	chr11.28666860.G.A	independent_variable	0,001235399	0,000499295	2,474287611	0,013665791
ADV3	chr11.28646124.G.A	independent_variable	-0,041885908	0,016935534	-2,473255762	0,013701847
ADV3	chr11.28646393.C.T	independent_variable	-0,041885908	0,016935534	-2,473255762	0,013701847
ADV3	chr11.28646869.T.C	independent_variable	-0,041885908	0,016935534	-2,473255762	0,013701847
ADV3	chr11.28648417.T.C	independent_variable	-0,041885908	0,016935534	-2,473255762	0,013701847
ADV3	chr11.28649222.A.G	independent_variable	-0,041885908	0,016935534	-2,473255762	0,013701847
ADV3	chr11.28649587.T.C	independent_variable	-0,041885908	0,016935534	-2,473255762	0,013701847
ADV3	chr11.28649653.C.G	independent_variable	-0,041885908	0,016935534	-2,473255762	0,013701847
ADV3	chr11.28650803.C.G	independent_variable	-0,041885908	0,016935534	-2,473255762	0,013701847
ADV3	chr11.28654623.A.G	independent_variable	-0,041885908	0,016935534	-2,473255762	0,013701847
ADV2	chr20.21371598.A.G	independent_variable	0,002394715	0,000969341	2,47045787	0,013799535
ADV4	chr11.28669740.C.T	independent_variable	0,001234737	0,000500111	2,468925124	0,013870453
ADV4	chr11.28620773.G.A	independent_variable	0,001239648	0,000502141	2,468728135	0,013879846
ADV4	chr11.28580674.A.G	independent_variable	0,001253155	0,000507653	2,468525183	0,013892656
ADV2	chr7.114429481.C.G	independent_variable	0,002503676	0,001014526	2,467827368	0,013915025
ADV1	chr5.88636991.G.T	independent_variable	-0,043348808	0,017582148	-2,465501215	0,014017606
ADV4	chr11.28582930.C.T	independent_variable	0,00124379	0,000504656	2,464628202	0,014040902
ADV4	chr11.28590845.G.A	independent_variable	0,00124379	0,000504656	2,464628202	0,014040902
ADV4	chr11.28592943.A.T	independent_variable	0,00124379	0,000504656	2,464628202	0,014040902
ADV4	chr11.28596075.C.A	independent_variable	0,00124379	0,000504656	2,464628202	0,014040902
ADV4	chr11.28596496.C.G	independent_variable	0,00124379	0,000504656	2,464628202	0,014040902
ADV4	chr11.28596864.G.A	independent_variable	0,00124379	0,000504656	2,464628202	0,014040902
ADV3	chr1.43853701.G.A	independent_variable	0,045339828	0,018405308	2,463410439	0,014070673
ADV1	chr14.98239063.A.G	independent_variable	-0,04356213	0,017699032	-2,461271867	0,014166351
ADV3	chr5.88563112.T.G	independent_variable	0,046979466	0,019096126	2,460156936	0,014227542
ADV3	chr14.98239063.A.G	independent_variable	0,046387985	0,018860191	2,459571314	0,014232853
ADV3	chr11.28622480.G.A	independent_variable	-0,041572732	0,016923727	-2,456476216	0,014352744
ADV1	chr5.88633148.G.T	independent_variable	-0,043114192	0,017586385	-2,451566398	0,014565366
ADV1	chr5.104559401.T.G	independent_variable	0,040185085	0,016413938	2,448229322	0,014687317
ADV3	chr11.28658309.G.A	independent_variable	-0,041506138	0,016961182	-2,447125347	0,014724914
ADV1	chr5.104556759.C.G	independent_variable	0,040118426	0,016429314	2,441880846	0,014945059
ADV1	chr11.28621752.T.C	independent_variable	0,039904734	0,016350692	2,44055329	0,01500011
ADV1	chr7.114489082.G.A	independent_variable	-0,039240393	0,016088695	-2,439004096	0,015058032
ADV1	chr7.114498696.G.C	independent_variable	-0,039240393	0,016088695	-2,439004096	0,015058032
ADV1	chr7.114502195.A.G	independent_variable	-0,039240393	0,016088695	-2,439004096	0,015058032
ADV1	chr5.88630645.G.A	independent_variable	-0,042975688	0,017618826	-2,439191304	0,015069
ADV4	chr5.104712889.C.G	independent_variable	0,001217056	0,0004992	2,438012111	0,01509831
ADV4	chr11.28621014.G.C	independent_variable	0,00123281	0,00050626	2,43513035	0,015224155
ADV4	chr3.71450250.C.T	independent_variable	0,001261637	0,000518173	2,434780105	0,015229144
ADV5	chr7.114589084.G.A	independent_variable	0,011262438	0,004630592	2,432180831	0,015340777
ADV3	chr5.104559315.C.T	independent_variable	-0,041848816	0,017208295	-2,431897846	0,015358445
ADV3	chr5.104559414.T.C	independent_variable	-0,041848816	0,017208295	-2,431897846	0,015358445
ADV3	chr5.104560429.T.C	independent_variable	-0,041848816	0,017208295	-2,431897846	0,015358445
ADV3	chr5.104562057.T.C	independent_variable	-0,041848816	0,017208295	-2,431897846	0,015358445
ADV3	chr5.104563477.A.G	independent_variable	-0,041848816	0,017208295	-2,431897846	0,015358445
ADV4	chr11.28586227.G.A	independent_variable	0,001232299	0,00050719	2,429660395	0,015455127
ADV3	chr11.28632740.G.A	independent_variable	-0,040861442	0,016825211	-2,428584218	0,01548701
ADV3	chr11.28633321.G.A	independent_variable	-0,040861442	0,016825211	-2,428584218	0,01548701
ADV3	chr11.28633511.A.C	independent_variable	-0,040861442	0,016825211	-2,428584218	0,01548701
ADV3	chr11.28634125.G.T	independent_variable	-0,040861442	0,016825211	-2,428584218	0,01548701
ADV3	chr11.28634351.C.G	independent_variable	-0,040861442	0,016825211	-2,428584218	0,01548701
ADV3	chr11.28634462.T.G	independent_variable	-0,040861442	0,016825211	-2,428584218	0,01548701
ADV3	chr11.28635703.A.G	independent_variable	-0,040861442	0,016825211	-2,428584218	0,01548701
ADV3	chr11.28637410.G.A	independent_variable	-0,040861442	0,016825211	-2,428584218	0,01548701
ADV3	chr11.28638055.A.T	independent_variable	-0,040861442	0,016825211	-2,428584218	0,01548701
ADV3	chr11.28638658.T.C	independent_variable	-0,040861442	0,016825211	-2,428584218	0,01548701
ADV3	chr11.28644512.C.G	independent_variable	-0,040861442	0,016825211	-2,428584218	0,01548701
ADV3	chr11.28644517.G.A	independent_variable	-0,040861442	0,016825211	-2,428584218	0,01548701
ADV3	chr11.28644793.A.C	independent_variable	-0,040861442	0,016825211	-2,428584218	0,01548701
ADV1	chr1.43547252.C.T	independent_variable	-0,045969871	0,01893061	-2,428335386	0,015538587
ADV4	chr11.28659176.C.A	independent_variable	0,001239871	0,000511655	2,423257723	0,015712779
ADV3	chr2.144956787.A.C	independent_variable	0,040711206	0,016823855	2,41985005	0,015870084
ADV1	chr2.144956787.A.C	independent_variable	-0,038142518	0,015770547	-2,418591886	0,015924443
ADV4	chr11.28605957.T.C	independent_variable	0,001177651	0,000487395	2,416212324	0,016012379
ADV3	chr1.43568097.C.A	independent_variable	0,045470955	0,018866879	2,410094127	0,016287369
ADV1	chr7.114627941.T.C	independent_variable	0,039538525	0,016412274	2,409082723	0,016356117
ADV1	chr5.88628064.T.G	independent_variable	-0,042377573	0,017607912	-2,406734662	0,016459657

ADV1	chr11.28639315.G.T	independent_variable	0,037886098	0,015766816	2,402901032	0,016605742
ADV1	chr7.114479375.G.A	independent_variable	-0,038661441	0,016109688	-2,39988754	0,016748102
ADV3	chr5.104568407.A.G	independent_variable	-0,041450966	0,017284158	-2,398205716	0,016827034
ADV3	chr11.28631449.A.G	independent_variable	-0,040530892	0,01690736	-2,397233592	0,016865894
ADV1	chr7.114469294.A.G	independent_variable	-0,038440505	0,016079189	-2,390699317	0,017167682
ADV5	chr7.114570759.T.C	independent_variable	0,011051508	0,004644082	2,379696805	0,017682413
ADV5	chr7.114605694.T.A	independent_variable	0,011561157	0,004858337	2,37965327	0,017700794
ADV4	chr11.28650082.G.C	independent_variable	0,001211862	0,000510533	2,373719384	0,017962036
ADV4	chr11.28648922.G.A	independent_variable	0,001208633	0,00050982	2,370703912	0,018106998
ADV4	chr11.28654958.T.C	independent_variable	0,001208633	0,00050982	2,370703912	0,018106998
ADV4	chr11.28655314.A.G	independent_variable	0,001208633	0,00050982	2,370703912	0,018106998
ADV4	chr11.28657323.A.G	independent_variable	0,001208633	0,00050982	2,370703912	0,018106998
ADV1	chr7.114503352.C.T	independent_variable	-0,038149408	0,016096625	-2,37002528	0,018148025
ADV1	chr11.28630347.G.C	independent_variable	0,037486536	0,015842039	2,366269727	0,018329091
ADV4	chr5.104709685.C.A	independent_variable	0,001150652	0,000486364	2,365826299	0,018339505
ADV4	chr5.104672432.G.A	independent_variable	0,001191575	0,000503928	2,36457303	0,018418657
ADV1	chr1.43663428.T.C	independent_variable	-0,043154238	0,018254357	-2,364051371	0,018453834
ADV4	chr11.28634517.A.T	independent_variable	0,001204791	0,000510525	2,359904647	0,018637719
ADV4	chr11.28634687.C.A	independent_variable	0,001204791	0,000510525	2,359904647	0,018637719
ADV4	chr11.28644991.C.T	independent_variable	0,001204791	0,000510525	2,359904647	0,018637719
ADV4	chr11.28646737.A.G	independent_variable	0,001204791	0,000510525	2,359904647	0,018637719
ADV4	chr11.28662674.G.A	independent_variable	0,001204791	0,000510525	2,359904647	0,018637719
ADV4	chr11.28664505.C.T	independent_variable	0,001204791	0,000510525	2,359904647	0,018637719
ADV4	chr11.28666219.T.C	independent_variable	0,001204791	0,000510525	2,359904647	0,018637719
ADV4	chr11.28668805.C.G	independent_variable	0,001204791	0,000510525	2,359904647	0,018637719
ADV4	chr11.28669587.A.G	independent_variable	0,001204791	0,000510525	2,359904647	0,018637719
ADV1	chr7.114497885.A.G	independent_variable	-0,037958979	0,01608571	-2,359795009	0,018650013
ADV4	chr5.104678081.G.A	independent_variable	0,001184802	0,000502065	2,3598589	0,018650356
ADV3	chr5.104605369.T.G	independent_variable	-0,040216062	0,017042728	-2,359719753	0,018651675
ADV3	chr5.104605700.T.C	independent_variable	-0,040216062	0,017042728	-2,359719753	0,018651675
ADV3	chr5.104605714.T.G	independent_variable	-0,040216062	0,017042728	-2,359719753	0,018651675
ADV3	chr1.43667345.T.C	independent_variable	0,043497682	0,018475584	2,354333259	0,018915118
ADV5	chr7.114560479.G.A	independent_variable	0,010942166	0,004651454	2,352418563	0,019019303
ADV3	chr6.70148037.A.G	independent_variable	-0,043032209	0,01830501	-2,350843173	0,01910056
ADV3	chr6.70148809.A.C	independent_variable	-0,043032209	0,01830501	-2,350843173	0,01910056
ADV3	chr6.70142601.A.G	independent_variable	-0,04325688	0,018408147	-2,349876902	0,019151819
ADV3	chr6.70142949.A.T	independent_variable	-0,04325688	0,018408147	-2,349876902	0,019151819
ADV3	chr6.70143876.T.C	independent_variable	-0,04325688	0,018408147	-2,349876902	0,019151819
ADV4	chr5.104665051.G.A	independent_variable	0,001185117	0,000504737	2,347991038	0,019248013
ADV4	chr5.104670966.A.G	independent_variable	0,001185117	0,000504737	2,347991038	0,019248013
ADV4	chr5.104671732.G.C	independent_variable	0,001185117	0,000504737	2,347991038	0,019248013
ADV2	chr7.114430980.C.T	independent_variable	0,002401218	0,00102296	2,347323767	0,019283593
ADV3	chr1.43671586.C.A	independent_variable	0,043066808	0,018369406	2,344485616	0,019417427
ADV3	chr5.104565723.C.T	independent_variable	-0,040482237	0,017268694	-2,344255838	0,01943835
ADV3	chr5.104568065.G.A	independent_variable	-0,040482237	0,017268694	-2,344255838	0,01943835
ADV3	chr6.70144291.A.G	independent_variable	-0,042848697	0,018282103	-2,343750965	0,019462972
ADV1	chr1.43667345.T.C	independent_variable	-0,04065848	0,01734891	-2,343575458	0,019463693
ADV1	chr1.43671586.C.A	independent_variable	-0,040324322	0,017220007	-2,341713458	0,019560726
ADV4	chr11.28655215.G.C	independent_variable	0,0011913	0,000509268	2,339241125	0,019689308
ADV3	chr1.43663428.T.C	independent_variable	0,045559825	0,019476264	2,339248735	0,019711471
ADV1	chr1.43537286.G.A	independent_variable	-0,045429199	0,019442341	-2,336611628	0,01987955
ADV4	chr11.28521551.G.A	independent_variable	0,00125865	0,00053916	2,334464661	0,019967224
ADV4	chr11.28526196.C.T	independent_variable	0,00125865	0,00053916	2,334464661	0,019967224
ADV4	chr11.28526682.A.C	independent_variable	0,00125865	0,00053916	2,334464661	0,019967224
ADV4	chr11.28622320.A.G	independent_variable	0,001152885	0,000494102	2,333291798	0,020007365
ADV4	chr11.28624925.C.T	independent_variable	0,001152885	0,000494102	2,333291798	0,020007365
ADV4	chr5.104655775.C.A	independent_variable	0,001173948	0,000503682	2,330734114	0,020147367
ADV1	chr5.88587685.T.C	independent_variable	-0,042110131	0,018109281	-2,325334262	0,020453351
ADV3	chr1.43510486.G.A	independent_variable	0,04996555	0,021523088	2,321486131	0,020706373
ADV3	chr11.28621752.T.C	independent_variable	-0,04030496	0,017398609	-2,316562145	0,020918375
ADV3	chr6.70148891.T.C	independent_variable	-0,042327162	0,018297244	-2,313308069	0,021093426
ADV3	chr11.28622320.A.G	independent_variable	-0,039070216	0,016897059	-2,31224951	0,021147702
ADV3	chr11.28624925.C.T	independent_variable	-0,039070216	0,016897059	-2,31224951	0,021147702
ADV4	chr5.104698439.C.T	independent_variable	0,001152399	0,000498678	2,310907076	0,021223073
ADV4	chr5.104707455.C.T	independent_variable	0,001152399	0,000498678	2,310907076	0,021223073
ADV1	chr5.104605369.T.G	independent_variable	0,037020293	0,01604783	2,306872199	0,021448069
ADV1	chr5.104605700.T.C	independent_variable	0,037020293	0,01604783	2,306872199	0,021448069
ADV1	chr5.104605714.T.G	independent_variable	0,037020293	0,01604783	2,306872199	0,021448069
ADV4	chr11.28623283.A.G	independent_variable	0,001140501	0,000494399	2,306844061	0,02144965
ADV4	chr11.28623794.G.C	independent_variable	0,001140501	0,000494399	2,306844061	0,02144965
ADV4	chr11.28628859.C.T	independent_variable	0,001140501	0,000494399	2,306844061	0,02144965

ADV4	chr11.28526861.T.C	independent_variable	0,001245474	0,000539859	2,307034176	0,021461468
ADV1	chr2.144962572.A.G	independent_variable	-0,036573944	0,015880432	-2,303082414	0,021670265
ADV1	chr5.104568407.A.G	independent_variable	0,037392007	0,016249962	2,301052017	0,021783361
ADV1	chr5.88588020.G.C	independent_variable	-0,04167789	0,018122032	-2,299846429	0,021868808
ADV3	chr7.114489082.G.A	independent_variable	0,03929434	0,017094954	2,29859284	0,021920055
ADV3	chr7.114498696.G.C	independent_variable	0,03929434	0,017094954	2,29859284	0,021920055
ADV3	chr7.114502195.A.G	independent_variable	0,03929434	0,017094954	2,29859284	0,021920055
ADV1	chr7.114484605.C.G	independent_variable	-0,03699168	0,01610793	-2,296488695	0,022040866
ADV3	chr7.114479375.G.A	independent_variable	0,039239056	0,017113021	2,292935724	0,022246937
ADV3	chr2.144962572.A.G	independent_variable	0,038823728	0,016936262	2,292343322	0,022286702
ADV1	chr7.114505470.G.A	independent_variable	-0,037360761	0,016328035	-2,288135832	0,022540319
ADV1	chr11.28620834.C.T	independent_variable	0,036450324	0,015961412	2,283652822	0,022794346
ADV5	chr7.114627941.T.C	independent_variable	0,011216812	0,004922114	2,278860671	0,023099505
ADV3	chr5.104606092.C.A	independent_variable	-0,038883562	0,017071855	-2,277641312	0,023146874
ADV2	chr7.114531090.A.G	independent_variable	0,002459365	0,001080136	2,276902794	0,023204587
ADV3	chr5.104571636.G.A	independent_variable	-0,03922226	0,017274253	-2,27056191	0,023579371
ADV4	chr11.28621752.T.C	independent_variable	0,001156348	0,000513777	2,250679017	0,024826106
ADV3	chr7.114497885.A.G	independent_variable	0,038442707	0,01708926	2,249524339	0,024892671
ADV2	chr5.104646025.T.G	independent_variable	-0,002390754	0,001062895	-2,249285575	0,024902497
ADV3	chr7.114469294.A.G	independent_variable	0,03840952	0,017083107	2,248391894	0,02496522
ADV2	chr5.104620815.C.G	independent_variable	-0,002377876	0,001061005	-2,241153856	0,025425459
ADV2	chr5.104628884.G.A	independent_variable	-0,002377876	0,001061005	-2,241153856	0,025425459
ADV2	chr5.104636656.G.A	independent_variable	-0,002377876	0,001061005	-2,241153856	0,025425459
ADV3	chr7.114503352.C.T	independent_variable	0,038296581	0,017111052	2,238119618	0,025632539
ADV2	chr7.114564074.T.A	independent_variable	-0,002409192	0,001076989	-2,236969618	0,025714634
ADV3	chr5.104676602.C.G	independent_variable	-0,040764815	0,018252086	-2,233433236	0,025971017
ADV3	chr5.104603973.C.T	independent_variable	-0,037833558	0,016957954	-2,231021415	0,026096503
ADV4	chr11.28655291.A.G	independent_variable	0,001117103	0,000501218	2,228774826	0,026237395
ADV1	chr5.104565723.C.T	independent_variable	0,036187287	0,016263996	2,224993601	0,026507473
ADV1	chr5.104568065.G.A	independent_variable	0,036187287	0,016263996	2,224993601	0,026507473
ADV1	chr5.104603973.C.T	independent_variable	0,035408817	0,01596218	2,218294557	0,026956996
ADV1	chr7.114476826.G.A	independent_variable	-0,03571023	0,01610435	-2,217427643	0,027021333
ADV3	chr11.28630347.G.C	independent_variable	-0,037481313	0,016907324	-2,216868427	0,027058149
ADV3	chr1.43537286.G.A	independent_variable	0,045787989	0,020699818	2,211999585	0,027447923
ADV4	chr16.61754062.A.G	independent_variable	0,00108108	0,000489024	2,210690164	0,027472595
ADV4	chr5.104686538.C.G	independent_variable	0,001106769	0,000500922	2,209464195	0,027574077
ADV2	chr3.20685150.G.A	independent_variable	-0,002479066	0,001121984	-2,209537875	0,027624877
ADV2	chr3.20685154.C.T	independent_variable	-0,002479066	0,001121984	-2,209537875	0,027624877
ADV3	chr5.104566479.C.T	independent_variable	-0,037693694	0,017104917	-2,203675867	0,027981347
ADV3	chr5.104567257.C.T	independent_variable	-0,037693694	0,017104917	-2,203675867	0,027981347
ADV3	chr5.104572545.C.T	independent_variable	-0,037693694	0,017104917	-2,203675867	0,027981347
ADV3	chr5.104572709.A.G	independent_variable	-0,037693694	0,017104917	-2,203675867	0,027981347
ADV3	chr5.104573167.C.T	independent_variable	-0,037693694	0,017104917	-2,203675867	0,027981347
ADV3	chr5.104573847.C.T	independent_variable	-0,037693694	0,017104917	-2,203675867	0,027981347
ADV3	chr5.104604069.C.T	independent_variable	-0,03735228	0,017048349	-2,190961675	0,028891711
ADV3	chr11.28639315.G.T	independent_variable	-0,036848522	0,016833509	-2,188998296	0,029029191
ADV2	chr20.21189014.G.A	independent_variable	0,002243439	0,001026224	2,186111108	0,029252606
ADV3	chr3.20627579.T.G	independent_variable	0,037695999	0,017266684	2,183163791	0,029462535
ADV2	chr7.114552490.T.C	independent_variable	0,002381552	0,001091047	2,182813185	0,029508494
ADV3	chr5.104568136.A.G	independent_variable	-0,037346218	0,017114145	-2,182184224	0,029539336
ADV3	chr5.104563895.T.C	independent_variable	-0,037834067	0,017337926	-2,182156411	0,029542249
ADV2	chr5.104612267.T.C	independent_variable	-0,002316011	0,001061659	-2,181501195	0,029580102
ADV3	chr1.43712399.T.G	independent_variable	0,040073553	0,018391736	2,178889121	0,029773109
ADV3	chr5.104563989.T.C	independent_variable	-0,037286366	0,017116977	-2,17832659	0,029826768
ADV3	chr5.104569324.T.A	independent_variable	-0,037286366	0,017116977	-2,17832659	0,029826768
ADV3	chr5.104569574.C.T	independent_variable	-0,037286366	0,017116977	-2,17832659	0,029826768
ADV3	chr5.104569709.A.C	independent_variable	-0,037286366	0,017116977	-2,17832659	0,029826768
ADV3	chr5.104570372.C.T	independent_variable	-0,037286366	0,017116977	-2,17832659	0,029826768
ADV3	chr5.104571905.G.A	independent_variable	-0,037286366	0,017116977	-2,17832659	0,029826768
ADV3	chr7.114484605.C.G	independent_variable	0,037208124	0,017113492	2,174198222	0,030136188
ADV1	chr1.43712399.T.G	independent_variable	-0,037507068	0,017270934	-2,171687317	0,030315433
ADV2	chr5.104619100.T.A	independent_variable	-0,00231248	0,001064892	-2,171562424	0,030327363
ADV3	chr5.104578822.T.C	independent_variable	-0,036170359	0,016662974	-2,170702529	0,030386222
ADV3	chr7.114505470.G.A	independent_variable	0,037695277	0,017366074	2,170627411	0,030422709
ADV3	chr1.43547252.C.T	independent_variable	0,043735421	0,020165297	2,16884586	0,030589858
ADV1	chr5.104604069.C.T	independent_variable	0,034780685	0,016051094	2,166873168	0,030691848
ADV3	chr5.104568333.A.G	independent_variable	-0,037059773	0,017145202	-2,16152445	0,031107857
ADV3	chr5.104568336.T.C	independent_variable	-0,037059773	0,017145202	-2,16152445	0,031107857
ADV2	chr7.114571857.G.A	independent_variable	-0,002141733	0,000991066	-2,161040606	0,031154264
ADV1	chr5.104559315.C.T	independent_variable	0,035120018	0,016269338	2,158663043	0,031335811
ADV1	chr5.104559414.T.C	independent_variable	0,035120018	0,016269338	2,158663043	0,031335811

ADV1	chr5.104560429.T.C	independent_variable	0,035120018	0,016269338	2,158663043	0,031335811
ADV1	chr5.104562057.T.C	independent_variable	0,035120018	0,016269338	2,158663043	0,031335811
ADV1	chr5.104563477.A.G	independent_variable	0,035120018	0,016269338	2,158663043	0,031335811
ADV5	chr7.114499629.G.A	independent_variable	0,010295223	0,004771199	2,157785191	0,031384783
ADV1	chr5.104606092.C.A	independent_variable	0,034643082	0,016079777	2,15445045	0,031656521
ADV1	chr5.88563112.T.G	independent_variable	-0,038779974	0,018003731	-2,153996558	0,031721491
ADV5	chr7.114464334.T.C	independent_variable	0,010264258	0,004777166	2,148608128	0,032110276
ADV4	chr3.20700799.C.T	independent_variable	-0,001162108	0,000541309	-2,146847251	0,032323104
ADV1	chr1.43610348.G.A	independent_variable	0,036036124	0,016793671	2,145815766	0,032329506
ADV5	chr7.114474994.A.G	independent_variable	0,010157943	0,004745982	2,14032476	0,032773374
ADV4	chr16.61746654.A.G	independent_variable	0,001027011	0,000480373	2,137945204	0,032972818
ADV4	chr16.61754668.C.T	independent_variable	0,001027011	0,000480373	2,137945204	0,032972818
ADV5	chr7.114475213.T.C	independent_variable	0,010212479	0,004778488	2,137177948	0,033032106
ADV3	chr16.61746654.A.G	independent_variable	-0,036186005	0,016958349	-2,13381648	0,033311103
ADV3	chr16.61754668.C.T	independent_variable	-0,036186005	0,016958349	-2,13381648	0,033311103
ADV1	chr5.104571636.G.A	independent_variable	0,034661629	0,016270349	2,130355612	0,033608278
ADV1	chr1.43610798.G.A	independent_variable	0,036062571	0,0169417	2,128627683	0,03373625
ADV3	chr7.114476826.G.A	independent_variable	0,03635674	0,017107331	2,125214094	0,034035097
ADV5	chr7.114483253.C.T	independent_variable	0,010113497	0,004785941	2,113167876	0,035044549
ADV1	chr7.114407487.G.C	independent_variable	-0,034198719	0,016192286	-2,112037695	0,035161315
ADV1	chr1.43634413.G.A	independent_variable	0,03523639	0,016690722	2,111136427	0,035217839
ADV4	chr11.28578333.T.A	independent_variable	0,001100074	0,000521024	2,11136926	0,035226473
ADV1	chr7.114403104.A.G	independent_variable	-0,033908812	0,016152283	-2,099320104	0,036271055
ADV2	chr7.114578527.C.T	independent_variable	-0,002070156	0,000987335	-2,09671152	0,0365063
ADV2	chr7.114583668.C.A	independent_variable	-0,002070156	0,000987335	-2,09671152	0,0365063
ADV2	chr7.114584108.A.G	independent_variable	-0,002070156	0,000987335	-2,09671152	0,0365063
ADV1	chr5.104578822.T.C	independent_variable	0,032829097	0,015662676	2,096008105	0,036543768
ADV3	chr7.114407487.G.C	independent_variable	0,03598536	0,017208777	2,091105052	0,037005269
ADV1	chr5.104566479.C.T	independent_variable	0,033682631	0,016117058	2,089874616	0,037110597
ADV1	chr5.104567257.C.T	independent_variable	0,033682631	0,016117058	2,089874616	0,037110597
ADV1	chr5.104572545.C.T	independent_variable	0,033682631	0,016117058	2,089874616	0,037110597
ADV1	chr5.104572709.A.G	independent_variable	0,033682631	0,016117058	2,089874616	0,037110597
ADV1	chr5.104573167.C.T	independent_variable	0,033682631	0,016117058	2,089874616	0,037110597
ADV1	chr5.104573847.C.T	independent_variable	0,033682631	0,016117058	2,089874616	0,037110597
ADV3	chr11.28620834.C.T	independent_variable	-0,035551827	0,017012058	-2,089801655	0,037120891
ADV3	chr16.61754062.A.G	independent_variable	-0,035183934	0,016844854	-2,08870518	0,037198525
ADV3	chr7.114403104.A.G	independent_variable	0,035784886	0,017167266	2,084483703	0,037604531
ADV3	chr5.104576413.A.C	independent_variable	-0,035363402	0,016974673	-2,083303816	0,037704921
ADV1	chr1.43540668.A.G	independent_variable	-0,0396339	0,019020639	-2,083731306	0,037718585
ADV1	chr5.104568136.A.G	independent_variable	0,03344361	0,016125379	2,073973599	0,038569581
ADV1	chr5.104563989.T.C	independent_variable	0,033383761	0,016127994	2,06992644	0,038948636
ADV1	chr5.104569324.T.A	independent_variable	0,033383761	0,016127994	2,06992644	0,038948636
ADV1	chr5.104569574.C.T	independent_variable	0,033383761	0,016127994	2,06992644	0,038948636
ADV1	chr5.104569709.A.C	independent_variable	0,033383761	0,016127994	2,06992644	0,038948636
ADV1	chr5.104570372.C.T	independent_variable	0,033383761	0,016127994	2,06992644	0,038948636
ADV1	chr5.104571905.G.A	independent_variable	0,033383761	0,016127994	2,06992644	0,038948636
ADV1	chr7.114406314.C.G	independent_variable	-0,033300086	0,016146926	-2,062317295	0,03967465
ADV3	chr5.104569213.T.C	independent_variable	-0,035245208	0,01712165	-2,058517046	0,040033418
ADV1	chr5.104568333.A.G	independent_variable	0,033200761	0,016150231	2,055745307	0,040302921
ADV1	chr5.104568336.T.C	independent_variable	0,033200761	0,016150231	2,055745307	0,040302921
ADV1	chr5.104563895.T.C	independent_variable	0,033544359	0,016329164	2,054260707	0,040446886
ADV1	chr1.43840448.A.G	independent_variable	0,035585203	0,01733482	2,052816428	0,040583584
ADV1	chr1.43845072.C.T	independent_variable	0,035585203	0,01733482	2,052816428	0,040583584
ADV1	chr1.43845203.G.A	independent_variable	0,035585203	0,01733482	2,052816428	0,040583584
ADV1	chr7.114436339.C.G	independent_variable	-0,033139708	0,016152142	-2,051722202	0,040696937
ADV3	chr7.114406314.C.G	independent_variable	0,035072806	0,017161812	2,043654005	0,04149208
ADV3	chr7.114436339.C.G	independent_variable	0,034983941	0,01712043	2,043403164	0,041516009
ADV5	chr7.114482451.C.T	independent_variable	0,009841148	0,00483043	2,037323655	0,042105552
ADV3	chr5.104581838.T.A	independent_variable	-0,034312794	0,016857502	-2,035461329	0,042295828
ADV3	chr5.104582136.T.C	independent_variable	-0,034312794	0,016857502	-2,035461329	0,042295828
ADV2	chr20.21227106.T.G	independent_variable	0,002091757	0,001029416	2,031985143	0,04264143
ADV1	chr1.43783814.A.G	independent_variable	-0,035491964	0,01747007	-2,031586808	0,0426967
ADV3	chr7.114429481.C.G	independent_variable	0,034656815	0,017168138	2,018670635	0,044035233
ADV1	chr1.43843195.A.G	independent_variable	0,034533743	0,01716021	2,012431192	0,044683705
ADV1	chr1.43631859.T.C	independent_variable	0,033824517	0,01682155	2,010784822	0,044843842
ADV1	chr7.114429481.C.G	independent_variable	-0,032565211	0,01619684	-2,010590374	0,044885496
ADV5	chr1.43566122.G.C	independent_variable	0,009604633	0,004779836	2,009406311	0,044996709
ADV4	chr3.20685150.G.A	independent_variable	-0,001065182	0,00053184	-2,002824814	0,04577734
ADV4	chr3.20685154.C.T	independent_variable	-0,001065182	0,00053184	-2,002824814	0,04577734
ADV3	chr7.114443495.C.G	independent_variable	0,034174736	0,017098951	1,998645165	0,046163878
ADV1	chr7.114443495.C.G	independent_variable	-0,032121552	0,016137215	-1,990526435	0,047052987

ADV5	chr7.114552490.T.C	independent_variable	0,010038592	0,005055756	1,985576974	0,047619619
ADV1	chr6.70142601.A.G	independent_variable	0,034300228	0,01736861	1,974840125	0,048813817
ADV1	chr6.70142949.A.T	independent_variable	0,034300228	0,01736861	1,974840125	0,048813817
ADV1	chr6.70143876.T.C	independent_variable	0,034300228	0,01736861	1,974840125	0,048813817
ADV2	chr4.111546456.G.A	independent_variable	-0,002224381	0,001126674	-1,974290313	0,04893627
ADV3	chr5.104602414.C.T	independent_variable	-0,033311317	0,016909383	-1,969990114	0,049359711
ADV3	chr7.114451789.C.A	independent_variable	0,033634389	0,017091032	1,967955473	0,049601033
ADV1	chr6.70148037.A.G	independent_variable	0,033907336	0,017270106	1,963354307	0,050133448
ADV1	chr6.70148809.A.C	independent_variable	0,033907336	0,017270106	1,963354307	0,050133448
ADV1	chr1.43837594.A.G	independent_variable	0,034286024	0,017479546	1,961493982	0,050351496
ADV1	chr6.70144291.A.G	independent_variable	0,033785489	0,017247487	1,958864508	0,05065759
ADV1	chr7.114451789.C.A	independent_variable	-0,031584569	0,016129835	-1,958145824	0,050744103
ADV4	chr3.20687599.C.T	independent_variable	-0,000941525	0,000481424	-1,955710335	0,051011695
ADV3	chr1.43783814.A.G	independent_variable	0,036376368	0,018619703	1,953649185	0,05127138
ADV2	chr20.21195965.C.T	independent_variable	0,002120354	0,001085823	1,95276243	0,051417841
ADV5	chr1.43843195.A.G	independent_variable	-0,009572581	0,004911703	-1,948933189	0,051834628
ADV4	chr11.28602326.G.T	independent_variable	0,00101322	0,000519998	1,948508973	0,051896813
ADV1	chr1.43612713.T.C	independent_variable	0,032763129	0,016842258	1,945293131	0,052257355
ADV1	chr1.43613740.T.C	independent_variable	0,032763129	0,016842258	1,945293131	0,052257355
ADV5	chr7.114531090.A.G	independent_variable	0,009725631	0,005003066	1,943934129	0,052451909
ADV1	chr5.104576413.A.C	independent_variable	0,031028463	0,015976276	1,942158699	0,052650318
ADV3	chr5.104576507.C.A	independent_variable	-0,032917474	0,016969628	-1,939787665	0,052936678
ADV1	chr3.20627579.T.G	independent_variable	-0,031547551	0,01627145	-1,938828433	0,053053718
ADV1	chr6.70148891.T.C	independent_variable	0,033421392	0,017242328	1,938334105	0,053121252
ADV1	chr5.104581838.T.A	independent_variable	0,03068886	0,015834678	1,93807914	0,053138346
ADV1	chr5.104582136.T.C	independent_variable	0,03068886	0,015834678	1,93807914	0,053138346
ADV1	chr1.43840750.C.T	independent_variable	0,033410881	0,017279679	1,933536028	0,053705401
ADV4	chr11.28569621.A.T	independent_variable	0,001006963	0,000520927	1,933022249	0,053786679
ADV4	chr11.28570040.C.T	independent_variable	0,001006963	0,000520927	1,933022249	0,053786679
ADV1	chr5.104569213.T.C	independent_variable	0,031140311	0,016130712	1,930498195	0,054083351
ADV5	chr1.43606749.A.G	independent_variable	-0,009530195	0,004940955	-1,928816466	0,054274529
ADV1	chr7.114416000.G.C	independent_variable	-0,030899719	0,01604404	-1,925931302	0,054653643
ADV2	chr20.21252915.A.G	independent_variable	0,001950993	0,001014114	1,923840522	0,054921145
ADV1	chr1.43848594.C.T	independent_variable	0,032784058	0,017061844	1,921483874	0,055207698
ADV3	chr5.88558577.A.C	independent_variable	0,037788113	0,019710017	1,917203479	0,055812872
ADV2	chr20.21187755.C.T	independent_variable	0,001996028	0,001042458	1,914732109	0,056076115
ADV1	chr5.104602414.C.T	independent_variable	0,030377001	0,015916167	1,90856253	0,056858452
ADV2	chr20.21457201.T.G	independent_variable	0,00199476	0,001045671	1,907636498	0,056976364
ADV2	chr20.21463034.C.G	independent_variable	0,00199476	0,001045671	1,907636498	0,056976364
ADV2	chr20.21465660.G.T	independent_variable	0,00199476	0,001045671	1,907636498	0,056976364
ADV3	chr7.114430980.C.T	independent_variable	0,033034663	0,017318608	1,907466414	0,057011905
ADV2	chr20.21187721.G.T	independent_variable	0,001980753	0,00104118	1,90241242	0,057669427
ADV2	chr20.21190532.C.T	independent_variable	0,001980753	0,00104118	1,90241242	0,057669427
ADV2	chr20.21194910.T.C	independent_variable	0,001980753	0,00104118	1,90241242	0,057669427
ADV2	chr20.21456154.A.G	independent_variable	0,001980909	0,001044477	1,896556734	0,058426882
ADV2	chr20.21456675.T.C	independent_variable	0,001980909	0,001044477	1,896556734	0,058426882
ADV2	chr20.21260820.G.A	independent_variable	0,001941191	0,001023509	1,896603067	0,058440735
ADV2	chr7.114518899.G.A	independent_variable	-0,002070153	0,001091893	-1,895930894	0,058524372
ADV1	chr1.43831901.A.C	independent_variable	0,032971191	0,017397517	1,895166456	0,058622577
ADV4	chr3.20678505.C.T	independent_variable	-0,000914108	0,000482562	-1,894282294	0,058726606
ADV5	chr6.70148891.T.C	independent_variable	0,009448553	0,004991869	1,892788633	0,058938913
ADV4	chr5.104659667.A.G	independent_variable	0,00092784	0,000490346	1,892215008	0,058993061
ADV1	chr7.114419101.C.T	independent_variable	-0,030307565	0,016038733	-1,889648312	0,059356751
ADV5	chr6.70148037.A.G	independent_variable	0,00939628	0,004978733	1,887283106	0,059674687
ADV5	chr6.70148809.A.C	independent_variable	0,00939628	0,004978733	1,887283106	0,059674687
ADV4	chr3.20683444.C.T	independent_variable	-0,000906179	0,000481742	-1,881044989	0,060507617
ADV5	chr6.70144291.A.G	independent_variable	0,009351975	0,004972381	1,880784075	0,060554546
ADV5	chr7.114503596.T.A	independent_variable	0,00896923	0,004769305	1,880615509	0,060562162
ADV2	chr5.104568525.A.G	independent_variable	-0,001973619	0,001050284	-1,87912818	0,060814288
ADV2	chr20.21165245.A.G	independent_variable	0,002009918	0,001070084	1,878280009	0,060880443
ADV5	chr7.114507162.C.A	independent_variable	0,009392197	0,00500529	1,876454155	0,0611604
ADV5	chr1.43848594.C.T	independent_variable	-0,009126034	0,004869279	-1,874206517	0,061452881
ADV5	chr7.114451698.G.A	independent_variable	0,008970897	0,004797424	1,869940628	0,062029922
ADV5	chr11.28569621.A.T	independent_variable	0,009085563	0,004860338	1,869327221	0,062150154
ADV5	chr11.28570040.C.T	independent_variable	0,009085563	0,004860338	1,869327221	0,062150154
ADV4	chr3.20682712.G.A	independent_variable	-0,000901443	0,000482633	-1,867760608	0,062341945
ADV3	chr1.43540668.A.G	independent_variable	0,037861205	0,020269413	1,867898486	0,062391739
ADV2	chr20.21249817.C.T	independent_variable	0,002100031	0,001125516	1,865838797	0,062651599
ADV3	chr7.114416000.G.C	independent_variable	0,031801802	0,017059894	1,864126578	0,062862909
ADV2	chr20.21154158.T.C	independent_variable	0,001976805	0,001066527	1,853498448	0,064352141
ADV5	chr5.104557847.T.A	independent_variable	0,008485949	0,004590984	1,84839443	0,065115037

ADV5	chr6.70142601.A.G	independent_variable	0,009234844	0,004996673	1,848198457	0,065140142
ADV5	chr6.70142949.A.T	independent_variable	0,009234844	0,004996673	1,848198457	0,065140142
ADV5	chr6.70143876.T.C	independent_variable	0,009234844	0,004996673	1,848198457	0,065140142
ADV5	chr11.28578333.T.A	independent_variable	0,008973239	0,004865098	1,844410922	0,065703021
ADV2	chr20.21228388.C.T	independent_variable	0,001985776	0,001080031	1,838627724	0,066534625
ADV5	chr5.104550512.T.G	independent_variable	0,00842281	0,004606167	1,828594361	0,068036365
ADV3	chr1.43840448.A.G	independent_variable	-0,033723581	0,018456384	-1,82720407	0,068234469
ADV3	chr1.43845072.C.T	independent_variable	-0,033723581	0,018456384	-1,82720407	0,068234469
ADV3	chr1.43845203.G.A	independent_variable	-0,033723581	0,018456384	-1,82720407	0,068234469
ADV5	chr5.104554395.C.T	independent_variable	0,008379337	0,004595338	1,823442751	0,068812498
ADV3	chr7.114419101.C.T	independent_variable	0,03108539	0,017053545	1,82281103	0,06890171
ADV1	chr7.114430980.C.T	independent_variable	-0,029759982	0,016343632	-1,820891611	0,069198988
ADV5	chr5.104557287.A.T	independent_variable	0,008364919	0,004599448	1,818678972	0,069537727
ADV3	chr5.104577563.A.C	independent_variable	-0,030745259	0,016945058	-1,814408595	0,070179019
ADV3	chr5.104577567.C.T	independent_variable	-0,030745259	0,016945058	-1,814408595	0,070179019
ADV3	chr5.104577620.T.A	independent_variable	-0,030745259	0,016945058	-1,814408595	0,070179019
ADV3	chr5.104578055.A.T	independent_variable	-0,030745259	0,016945058	-1,814408595	0,070179019
ADV3	chr5.104578313.C.T	independent_variable	-0,030745259	0,016945058	-1,814408595	0,070179019
ADV3	chr5.104578842.G.A	independent_variable	-0,030745259	0,016945058	-1,814408595	0,070179019
ADV3	chr5.104579057.A.G	independent_variable	-0,030745259	0,016945058	-1,814408595	0,070179019
ADV3	chr5.104579085.A.T	independent_variable	-0,030745259	0,016945058	-1,814408595	0,070179019
ADV3	chr5.104579135.T.G	independent_variable	-0,030745259	0,016945058	-1,814408595	0,070179019
ADV3	chr5.104579581.A.G	independent_variable	-0,030745259	0,016945058	-1,814408595	0,070179019
ADV3	chr5.104579742.G.A	independent_variable	-0,030745259	0,016945058	-1,814408595	0,070179019
ADV3	chr5.104580027.A.G	independent_variable	-0,030745259	0,016945058	-1,814408595	0,070179019
ADV3	chr5.104580648.T.C	independent_variable	-0,030745259	0,016945058	-1,814408595	0,070179019
ADV3	chr5.104580662.G.C	independent_variable	-0,030745259	0,016945058	-1,814408595	0,070179019
ADV3	chr5.104581130.A.G	independent_variable	-0,030745259	0,016945058	-1,814408595	0,070179019
ADV5	chr7.114456791.A.T	independent_variable	0,00879958	0,004850548	1,814141474	0,070212843
ADV3	chr5.104584139.G.A	independent_variable	-0,030521295	0,016919779	-1,803882648	0,071815237
ADV5	chr11.28602326.G.T	independent_variable	0,008727505	0,004864343	1,794179559	0,073370937
ADV2	chr20.21175938.T.C	independent_variable	0,001891092	0,00105453	1,793303468	0,073509896
ADV2	chr20.21179524.A.C	independent_variable	0,001891092	0,00105453	1,793303468	0,073509896
ADV5	chr5.88526854.T.A	independent_variable	-0,009629701	0,005381079	-1,789548105	0,074150386
ADV1	chr5.104576507.C.A	independent_variable	0,028541496	0,0159815	1,785908508	0,074686758
ADV5	chr5.104559401.T.G	independent_variable	0,008232752	0,004611048	1,785440575	0,074775963
ADV5	chr5.104556759.C.G	independent_variable	0,008218216	0,004615306	1,780643739	0,07555778
ADV2	chr7.114470513.C.T	independent_variable	-0,001877443	0,001059032	-1,772791501	0,076822794
ADV5	chr5.88629003.C.T	independent_variable	-0,008994515	0,005075755	-1,772054566	0,076995653
ADV3	chr5.104581651.A.C	independent_variable	-0,029908677	0,016906285	-1,769086359	0,07745128
ADV3	chr5.104582166.G.T	independent_variable	-0,029908677	0,016906285	-1,769086359	0,07745128
ADV3	chr5.104582921.C.T	independent_variable	-0,029908677	0,016906285	-1,769086359	0,07745128
ADV3	chr5.104582998.G.A	independent_variable	-0,029908677	0,016906285	-1,769086359	0,07745128
ADV3	chr5.104583048.G.T	independent_variable	-0,029908677	0,016906285	-1,769086359	0,07745128
ADV3	chr5.104584121.A.C	independent_variable	-0,029908677	0,016906285	-1,769086359	0,07745128
ADV1	chr5.104554297.G.C	independent_variable	0,028588992	0,016170715	1,767948508	0,077661547
ADV3	chr5.104582527.C.T	independent_variable	-0,02981532	0,016878735	-1,766442871	0,07789284
ADV4	chr3.20706828.T.G	independent_variable	-0,000976285	0,000554328	-1,76120596	0,078856529
ADV5	chr5.88558577.A.C	independent_variable	-0,008893159	0,005077101	-1,751621249	0,080485677
ADV2	chr20.21073855.C.T	independent_variable	0,001783784	0,001020324	1,748252837	0,081016119
ADV2	chr20.21400134.C.A	independent_variable	0,001848026	0,001058381	1,746088101	0,08137394
ADV4	chr3.20703004.G.A	independent_variable	-0,000969672	0,000556028	-1,743925574	0,081830056
ADV3	chr1.43840750.C.T	independent_variable	-0,03194633	0,018395035	-1,736682176	0,083027623
ADV3	chr5.88526854.T.A	independent_variable	0,035167225	0,020266188	1,735265934	0,083328916
ADV1	chr16.61746654.A.G	independent_variable	0,027656188	0,015969022	1,731864827	0,083872962
ADV1	chr16.61754668.C.T	independent_variable	0,027656188	0,015969022	1,731864827	0,083872962
ADV3	chr5.104577501.A.G	independent_variable	-0,029229073	0,016973334	-1,722058432	0,08564347
ADV3	chr1.43837594.A.G	independent_variable	-0,032033718	0,018620402	-1,720355874	0,085959285
ADV1	chr20.21252915.A.G	independent_variable	-0,027204687	0,01584493	-1,716933289	0,086587974
ADV3	chr1.43610798.G.A	independent_variable	-0,030867412	0,018050226	-1,710084556	0,087823599
ADV3	chr5.88885292.T.G	independent_variable	0,033620593	0,019705529	1,706150301	0,088607351
ADV2	chr7.114381815.T.C	independent_variable	-0,001808565	0,001065439	-1,69748272	0,090185909
ADV1	chr16.61754062.A.G	independent_variable	0,026827865	0,015853666	1,69221835	0,091176972
ADV3	chr12.89378126.T.C	independent_variable	-0,032427804	0,019180923	-1,690627952	0,091589746
ADV3	chr1.43610348.G.A	independent_variable	-0,030229215	0,01789666	-1,689098094	0,091773651
ADV3	chr1.43831901.A.C	independent_variable	-0,031301421	0,018535291	-1,688747198	0,091865098
ADV5	chr16.61746654.A.G	independent_variable	0,007814464	0,004629233	1,688068651	0,091979751
ADV5	chr16.61754668.C.T	independent_variable	0,007814464	0,004629233	1,688068651	0,091979751
ADV4	chr1.43574039.T.C	independent_variable	0,000900038	0,000533405	1,68734438	0,092128878
ADV1	chr5.88569970.A.G	independent_variable	0,031608986	0,018737665	1,686922383	0,092245774
ADV2	chr20.21422129.C.A	independent_variable	0,00174211	0,001032932	1,686567908	0,092267272

ADV1	chr5.104577563.A.C	independent_variable	0,026892689	0,015953554	1,685686412	0,092443693
ADV1	chr5.104577567.C.T	independent_variable	0,026892689	0,015953554	1,685686412	0,092443693
ADV1	chr5.104577620.T.A	independent_variable	0,026892689	0,015953554	1,685686412	0,092443693
ADV1	chr5.104578055.A.T	independent_variable	0,026892689	0,015953554	1,685686412	0,092443693
ADV1	chr5.104578313.C.T	independent_variable	0,026892689	0,015953554	1,685686412	0,092443693
ADV1	chr5.104578842.G.A	independent_variable	0,026892689	0,015953554	1,685686412	0,092443693
ADV1	chr5.104579057.A.G	independent_variable	0,026892689	0,015953554	1,685686412	0,092443693
ADV1	chr5.104579085.A.T	independent_variable	0,026892689	0,015953554	1,685686412	0,092443693
ADV1	chr5.104579135.T.G	independent_variable	0,026892689	0,015953554	1,685686412	0,092443693
ADV1	chr5.104579581.A.G	independent_variable	0,026892689	0,015953554	1,685686412	0,092443693
ADV1	chr5.104579742.G.A	independent_variable	0,026892689	0,015953554	1,685686412	0,092443693
ADV1	chr5.104580027.A.G	independent_variable	0,026892689	0,015953554	1,685686412	0,092443693
ADV1	chr5.104580648.T.C	independent_variable	0,026892689	0,015953554	1,685686412	0,092443693
ADV1	chr5.104580662.G.C	independent_variable	0,026892689	0,015953554	1,685686412	0,092443693
ADV1	chr5.104581130.A.G	independent_variable	0,026892689	0,015953554	1,685686412	0,092443693
ADV2	chr7.114384998.A.G	independent_variable	-0,001796338	0,001066201	-1,684802749	0,092610921
ADV2	chr7.114608796.A.G	independent_variable	-0,001887297	0,001120554	-1,684254036	0,092728089
ADV1	chr5.104584139.G.A	independent_variable	0,026746431	0,015928923	1,679111099	0,093717567
ADV1	chr5.104585069.C.A	independent_variable	0,031020489	0,018496463	1,677103889	0,094152208
ADV1	chr20.21260820.G.A	independent_variable	-0,026605299	0,015887613	-1,674593824	0,094622232
ADV4	chr1.43565822.A.G	independent_variable	0,000927396	0,000554019	1,673943509	0,094729478
ADV5	chr5.88871923.G.C	independent_variable	0,008044981	0,004807868	1,673294789	0,094936081
ADV5	chr7.114440281.T.G	independent_variable	0,00786265	0,004711135	1,668950457	0,09571333
ADV5	chr5.88872578.C.G	independent_variable	0,008022902	0,004818102	1,665158266	0,096550426
ADV5	chr5.88873138.C.A	independent_variable	0,008022902	0,004818102	1,665158266	0,096550426
ADV3	chr12.89377735.T.C	independent_variable	-0,031825278	0,019137269	-1,662999991	0,096995061
ADV5	chr16.61754062.A.G	independent_variable	0,007602206	0,004575091	1,661651219	0,097158843
ADV1	chr5.104590343.C.T	independent_variable	0,03061529	0,018459445	1,658516287	0,097841703
ADV3	chr12.89382507.T.C	independent_variable	-0,031759625	0,019169454	-1,656782928	0,098248212
ADV2	chr5.104554297.G.C	independent_variable	-0,001675818	0,00101263	-1,654916035	0,098550776
ADV4	chr1.43663428.T.C	independent_variable	0,000930929	0,000563403	1,652331237	0,099088611
ADV1	chr5.104581651.A.C	independent_variable	0,026211027	0,015914332	1,647007667	0,100145875
ADV1	chr5.104582166.G.T	independent_variable	0,026211027	0,015914332	1,647007667	0,100145875
ADV1	chr5.104582921.C.T	independent_variable	0,026211027	0,015914332	1,647007667	0,100145875
ADV1	chr5.104582998.G.A	independent_variable	0,026211027	0,015914332	1,647007667	0,100145875
ADV1	chr5.104583048.G.T	independent_variable	0,026211027	0,015914332	1,647007667	0,100145875
ADV1	chr5.104584121.A.C	independent_variable	0,026211027	0,015914332	1,647007667	0,100145875
ADV4	chr1.43579794.A.G	independent_variable	0,000873446	0,000530412	1,646731549	0,100202666
ADV5	chr7.114442439.T.C	independent_variable	0,007755374	0,004712076	1,645850739	0,100381797
ADV1	chr5.104582527.C.T	independent_variable	0,02612751	0,015888368	1,644442678	0,100673322
ADV1	chr5.88567792.C.T	independent_variable	0,030668633	0,018694858	1,640484957	0,101537064
ADV1	chr5.88574430.T.C	independent_variable	0,030668633	0,018694858	1,640484957	0,101537064
ADV1	chr5.88577805.C.T	independent_variable	0,030668633	0,018694858	1,640484957	0,101537064
ADV3	chr1.43634413.G.A	independent_variable	-0,02912091	0,017791731	-1,636766506	0,102259734
ADV5	chr12.89368722.C.G	independent_variable	0,009000516	0,005502609	1,635681514	0,102557712
ADV5	chr12.89369752.C.T	independent_variable	0,009000516	0,005502609	1,635681514	0,102557712
ADV2	chr20.21176870.T.G	independent_variable	0,00173821	0,001063896	1,63381634	0,102906188
ADV5	chr12.89381252.T.G	independent_variable	0,009033206	0,005538174	1,631080219	0,103529305
ADV1	chr5.88579561.C.T	independent_variable	0,030591247	0,018768788	1,629899954	0,103757493
ADV1	chr5.88584754.A.G	independent_variable	0,030591247	0,018768788	1,629899954	0,103757493
ADV1	chr5.88586361.A.G	independent_variable	0,030591247	0,018768788	1,629899954	0,103757493
ADV1	chr5.88885292.T.G	independent_variable	-0,030117305	0,018599922	-1,619216773	0,106037541
ADV2	chr5.104482711.A.G	independent_variable	-0,001569096	0,000973264	-1,612199729	0,107500586
ADV5	chr5.88873835.A.G	independent_variable	0,007771321	0,004819423	1,612500376	0,107527198
ADV5	chr5.88874514.T.C	independent_variable	0,007771321	0,004819423	1,612500376	0,107527198
ADV4	chr3.20696597.A.G	independent_variable	-0,000898555	0,000557878	-1,610666898	0,107915231
ADV4	chr3.20698841.C.G	independent_variable	-0,000898555	0,000557878	-1,610666898	0,107915231
ADV5	chr12.89364160.G.A	independent_variable	0,008884475	0,00552609	1,607732618	0,108540512
ADV5	chr12.89366967.A.G	independent_variable	0,008884475	0,00552609	1,607732618	0,108540512
ADV2	chr20.21154956.G.T	independent_variable	0,001791384	0,001114878	1,60679832	0,108697574
ADV5	chr11.28521551.G.A	independent_variable	0,00806586	0,005028751	1,60394884	0,109358038
ADV5	chr11.28526196.C.T	independent_variable	0,00806586	0,005028751	1,60394884	0,109358038
ADV5	chr11.28526682.A.C	independent_variable	0,00806586	0,005028751	1,60394884	0,109358038
ADV2	chr20.21146082.A.G	independent_variable	0,001790851	0,001119699	1,599404801	0,110329996
ADV4	chr3.20695381.T.C	independent_variable	-0,00089126	0,000559564	-1,592776245	0,111875597
ADV1	chr5.104577501.A.G	independent_variable	0,025391936	0,015973903	1,589588741	0,112524966
ADV2	chr7.114647061.T.A	independent_variable	-0,001744137	0,001099762	-1,58592133	0,113380628
ADV5	chr5.104559315.C.T	independent_variable	0,007233747	0,004570431	1,582727335	0,114095136
ADV5	chr5.104559414.T.C	independent_variable	0,007233747	0,004570431	1,582727335	0,114095136
ADV5	chr5.104560429.T.C	independent_variable	0,007233747	0,004570431	1,582727335	0,114095136
ADV5	chr5.104562057.T.C	independent_variable	0,007233747	0,004570431	1,582727335	0,114095136

ADV5	chr5.104563477.A.G	independent_variable	0,007233747	0,004570431	1,582727335	0,114095136
ADV5	chr11.28526861.T.C	independent_variable	0,007949791	0,005035038	1,578894022	0,114994035
ADV4	chr5.104742532.A.C	independent_variable	0,000848426	0,000540887	1,568581933	0,117356016
ADV4	chr5.104746478.A.C	independent_variable	0,000848426	0,000540887	1,568581933	0,117356016
ADV3	chr1.43631859.T.C	independent_variable	-0,028071957	0,017927354	-1,565872875	0,117963092
ADV4	chr1.43576868.C.G	independent_variable	0,000815703	0,000521122	1,565284456	0,118108606
ADV1	chr20.21291449.A.T	independent_variable	-0,024838237	0,015871366	-1,564971587	0,118207401
ADV2	chr20.21141038.A.T	independent_variable	0,001737675	0,001113761	1,5601865	0,119314966
ADV4	chr5.104739429.T.C	independent_variable	0,000844205	0,00054161	1,558696134	0,119680219
ADV5	chr5.88587685.T.C	independent_variable	-0,008012002	0,005145702	-1,557027935	0,120096559
ADV1	chr5.88574395.G.A	independent_variable	0,028747284	0,018474654	1,556039102	0,120341786
ADV5	chr5.88600784.C.T	independent_variable	-0,008029348	0,005179875	-1,550104628	0,121755492
ADV5	chr5.88588020.G.C	independent_variable	-0,007975931	0,005152632	-1,547933314	0,122272306
ADV1	chr20.21294655.A.C	independent_variable	-0,024414354	0,015781543	-1,547019449	0,122468081
ADV2	chr20.21428075.A.G	independent_variable	0,00164047	0,001060921	1,546270231	0,122628413
ADV2	chr20.21436041.T.G	independent_variable	0,00164047	0,001060921	1,546270231	0,122628413
ADV2	chr20.21439854.C.T	independent_variable	0,00164047	0,001060921	1,546270231	0,122628413
ADV2	chr20.21440237.T.C	independent_variable	0,00164047	0,001060921	1,546270231	0,122628413
ADV2	chr20.21444512.T.A	independent_variable	0,00164047	0,001060921	1,546270231	0,122628413
ADV2	chr20.21444955.G.C	independent_variable	0,00164047	0,001060921	1,546270231	0,122628413
ADV2	chr20.21449932.G.A	independent_variable	0,00164047	0,001060921	1,546270231	0,122628413
ADV5	chr5.104670966.A.G	independent_variable	0,007290623	0,004732092	1,540676268	0,124002713
ADV3	chr1.43612713.T.C	independent_variable	-0,027596421	0,017941202	-1,538158972	0,124594394
ADV3	chr1.43613740.T.C	independent_variable	-0,027596421	0,017941202	-1,538158972	0,124594394
ADV2	chr20.21155077.G.T	independent_variable	0,001716215	0,00111992	1,532443765	0,126013025
ADV5	chr5.104712889.C.G	independent_variable	0,007178919	0,004691098	1,530328166	0,126536672
ADV1	chr20.21328174.G.A	independent_variable	-0,024079979	0,015766012	-1,527334782	0,127285793
ADV2	chr20.21175529.A.G	independent_variable	0,001628085	0,001069691	1,522014521	0,128616817
ADV2	chr20.21176291.G.C	independent_variable	0,001628085	0,001069691	1,522014521	0,128616817
ADV2	chr20.21176925.T.C	independent_variable	0,001628085	0,001069691	1,522014521	0,128616817
ADV2	chr20.21180143.G.A	independent_variable	0,001628085	0,001069691	1,522014521	0,128616817
ADV2	chr20.21183159.A.G	independent_variable	0,001628085	0,001069691	1,522014521	0,128616817
ADV2	chr20.21144231.T.C	independent_variable	0,0017168	0,001129995	1,519299786	0,12928674
ADV4	chr5.104619100.T.A	independent_variable	0,000802813	0,000529437	1,516352835	0,130020774
ADV4	chr1.43577434.T.C	independent_variable	0,0007919	0,000523301	1,513277818	0,130800706
ADV1	chr5.88558577.A.C	independent_variable	-0,028172901	0,018650159	-1,510598443	0,131557121
ADV1	chr20.21350903.T.G	independent_variable	-0,023956112	0,015864349	-1,510059529	0,131634454
ADV1	chr20.21299182.T.G	independent_variable	-0,023802375	0,015766823	-1,509649417	0,13174027
ADV5	chr11.28585698.T.C	independent_variable	0,005012141	0,003324188	1,507779091	0,132227839
ADV4	chr1.43560382.T.G	independent_variable	0,000830315	0,00055174	1,504902068	0,132940484
ADV4	chr1.43560509.A.C	independent_variable	0,000830315	0,00055174	1,504902068	0,132940484
ADV1	chr1.43896951.G.A	independent_variable	-0,026280624	0,017469324	-1,50438703	0,13311313
ADV4	chr16.61755912.G.C	independent_variable	-0,000798366	0,000532386	-1,499599137	0,134309226
ADV2	chr20.21169993.T.C	independent_variable	0,001638545	0,001093311	1,498700553	0,134554663
ADV2	chr20.21173142.G.C	independent_variable	0,001638545	0,001093311	1,498700553	0,134554663
ADV5	chr5.88605345.G.A	independent_variable	-0,00761452	0,005085554	-1,49728439	0,134949003
ADV5	chr5.88618013.G.A	independent_variable	-0,00761452	0,005085554	-1,49728439	0,134949003
ADV5	chr5.88618446.A.G	independent_variable	-0,00761452	0,005085554	-1,49728439	0,134949003
ADV5	chr5.88619663.G.T	independent_variable	-0,00761452	0,005085554	-1,49728439	0,134949003
ADV4	chr1.43570414.G.A	independent_variable	-0,000760379	0,000507933	-1,497004198	0,134984085
ADV4	chr5.104612267.T.C	independent_variable	0,000788878	0,000527909	1,494343184	0,135674398
ADV5	chr11.28582122.A.G	independent_variable	0,004958504	0,003319118	1,493922175	0,135810762
ADV5	chr11.28585152.T.C	independent_variable	0,004958504	0,003319118	1,493922175	0,135810762
ADV5	chr11.28587183.C.G	independent_variable	0,004958504	0,003319118	1,493922175	0,135810762
ADV5	chr11.28593837.A.G	independent_variable	0,004958504	0,003319118	1,493922175	0,135810762
ADV5	chr11.28594152.A.G	independent_variable	0,004958504	0,003319118	1,493922175	0,135810762
ADV5	chr11.28594153.T.C	independent_variable	0,004958504	0,003319118	1,493922175	0,135810762
ADV5	chr11.28594704.T.C	independent_variable	0,004958504	0,003319118	1,493922175	0,135810762
ADV5	chr11.28597209.G.A	independent_variable	0,004958504	0,003319118	1,493922175	0,135810762
ADV4	chr16.61758377.A.G	independent_variable	-0,000790531	0,000529377	-1,493322397	0,135940114
ADV1	chr20.21291031.G.C	independent_variable	-0,023905816	0,016009711	-1,49320717	0,136001337
ADV5	chr5.88867954.G.A	independent_variable	0,006819499	0,004569973	1,492240368	0,136247265
ADV1	chr20.21314871.G.T	independent_variable	-0,023585832	0,0158165	-1,491216955	0,136513242
ADV1	chr20.21310893.C.T	independent_variable	-0,023585832	0,0158165	-1,491216955	0,136513242
ADV1	chr20.21311200.C.T	independent_variable	-0,023585832	0,0158165	-1,491216955	0,136513242
ADV1	chr20.21311590.G.C	independent_variable	-0,023585832	0,0158165	-1,491216955	0,136513242
ADV1	chr20.21312173.C.T	independent_variable	-0,023585832	0,0158165	-1,491216955	0,136513242
ADV1	chr20.21312391.G.A	independent_variable	-0,023585832	0,0158165	-1,491216955	0,136513242
ADV4	chr16.61749670.C.T	independent_variable	-0,000794535	0,000533037	-1,490579934	0,13666335
ADV1	chr5.104596006.G.A	independent_variable	0,027153911	0,018233669	1,489218161	0,137062929
ADV5	chr5.88624488.C.A	independent_variable	-0,007577858	0,005092504	-1,488041634	0,1373703

ADV1	chr20.21313246.C.T	independent_variable	-0,023475931	0,015788512	-1,486899577	0,137648576
ADV2	chr20.21176320.A.G	independent_variable	0,001590978	0,001071674	1,484573237	0,138266335
ADV4	chr16.61753223.C.T	independent_variable	-0,000786739	0,000530022	-1,484352603	0,138303218
ADV1	chr1.43885226.G.T	independent_variable	-0,025824723	0,017482619	-1,477165549	0,140262091
ADV2	chr20.21412561.A.G	independent_variable	0,001548324	0,001049095	1,47586632	0,140558347
ADV3	chr5.88569970.A.G	independent_variable	-0,029314858	0,019895516	-1,473440459	0,141265357
ADV1	chr5.104629522.A.C	independent_variable	0,026877146	0,018294668	1,469124581	0,142438098
ADV2	chr20.21177599.C.G	independent_variable	0,001576935	0,001073588	1,468845282	0,142485301
ADV2	chr20.21177731.T.G	independent_variable	0,001576935	0,001073588	1,468845282	0,142485301
ADV2	chr20.21178512.T.C	independent_variable	0,001576935	0,001073588	1,468845282	0,142485301
ADV1	chr20.21289658.C.T	independent_variable	-0,023421669	0,015965705	-1,466998726	0,142990356
ADV1	chr20.21291893.C.T	independent_variable	-0,023421669	0,015965705	-1,466998726	0,142990356
ADV4	chr5.104734216.A.G	independent_variable	0,000762634	0,000522297	1,460153047	0,144827397
ADV5	chr5.104665051.G.A	independent_variable	0,006904296	0,004733205	1,458693817	0,145251983
ADV5	chr5.104671732.G.C	independent_variable	0,006904296	0,004733205	1,458693817	0,145251983
ADV4	chr20.21252915.A.G	independent_variable	0,000736265	0,000505254	1,457216943	0,145661266
ADV1	chr20.21308964.A.G	independent_variable	-0,02297519	0,015801683	-1,453971057	0,146558767
ADV1	chr20.21315921.C.A	independent_variable	-0,02297519	0,015801683	-1,453971057	0,146558767
ADV4	chr5.104620815.C.G	independent_variable	0,000767043	0,000527746	1,453431931	0,14669018
ADV4	chr5.104628884.G.A	independent_variable	0,000767043	0,000527746	1,453431931	0,14669018
ADV4	chr5.104636656.G.A	independent_variable	0,000767043	0,000527746	1,453431931	0,14669018
ADV1	chr20.21293229.C.T	independent_variable	-0,022841611	0,015719967	-1,453031746	0,146820446
ADV1	chr20.21294030.G.A	independent_variable	-0,022841611	0,015719967	-1,453031746	0,146820446
ADV1	chr20.21318093.T.C	independent_variable	-0,022867862	0,015773805	-1,449736601	0,14773482
ADV1	chr20.21318094.G.T	independent_variable	-0,022867862	0,015773805	-1,449736601	0,14773482
ADV1	chr20.21319392.G.A	independent_variable	-0,022867862	0,015773805	-1,449736601	0,14773482
ADV1	chr20.21320839.C.T	independent_variable	-0,022867862	0,015773805	-1,449736601	0,14773482
ADV1	chr20.21321927.C.T	independent_variable	-0,022867862	0,015773805	-1,449736601	0,14773482
ADV1	chr20.21321943.T.C	independent_variable	-0,022867862	0,015773805	-1,449736601	0,14773482
ADV1	chr20.21327058.G.A	independent_variable	-0,022867862	0,015773805	-1,449736601	0,14773482
ADV1	chr20.21329677.C.T	independent_variable	-0,022867862	0,015773805	-1,449736601	0,14773482
ADV5	chr5.88628064.T.G	independent_variable	-0,007392767	0,00510396	-1,448437618	0,148125276
ADV5	chr5.88630645.G.A	independent_variable	-0,007392767	0,00510396	-1,448437618	0,148125276
ADV4	chr1.43887565.G.A	independent_variable	0,000753674	0,00052162	1,444872239	0,14911274
ADV2	chr20.21138467.C.G	independent_variable	0,001639729	0,001135478	1,44408703	0,149308721
ADV2	chr20.21140567.A.T	independent_variable	0,001639729	0,001135478	1,44408703	0,149308721
ADV5	chr5.104655775.C.A	independent_variable	0,006814496	0,004723089	1,442805097	0,149675154
ADV1	chr20.21299701.C.T	independent_variable	-0,022729708	0,015765781	-1,4417115	0,149985731
ADV5	chr1.43560985.G.C	independent_variable	-0,007385141	0,005126393	-1,440611487	0,150281628
ADV1	chr1.43903763.G.A	independent_variable	-0,024872525	0,01730176	-1,437571975	0,151176095
ADV4	chr20.21260820.G.A	independent_variable	0,000733146	0,000510079	1,437319536	0,151235681
ADV1	chr20.21283152.A.T	independent_variable	-0,022807684	0,015950351	-1,429917325	0,153349479
ADV1	chr20.21284820.G.A	independent_variable	-0,022807684	0,015950351	-1,429917325	0,153349479
ADV1	chr20.21285090.C.T	independent_variable	-0,022807684	0,015950351	-1,429917325	0,153349479
ADV1	chr20.21285802.C.T	independent_variable	-0,022807684	0,015950351	-1,429917325	0,153349479
ADV1	chr20.21286713.C.T	independent_variable	-0,022807684	0,015950351	-1,429917325	0,153349479
ADV1	chr20.21287631.G.A	independent_variable	-0,022807684	0,015950351	-1,429917325	0,153349479
ADV1	chr20.21290685.C.G	independent_variable	-0,022807684	0,015950351	-1,429917325	0,153349479
ADV1	chr20.21290915.A.G	independent_variable	-0,022807684	0,015950351	-1,429917325	0,153349479
ADV1	chr20.21290947.G.A	independent_variable	-0,022807684	0,015950351	-1,429917325	0,153349479
ADV1	chr20.21291136.G.T	independent_variable	-0,022807684	0,015950351	-1,429917325	0,153349479
ADV1	chr20.21292673.G.A	independent_variable	-0,022807684	0,015950351	-1,429917325	0,153349479
ADV5	chr5.88851954.G.A	independent_variable	0,006501529	0,004560542	1,425604288	0,154578007
ADV5	chr5.88852224.C.T	independent_variable	0,006501529	0,004560542	1,425604288	0,154578007
ADV5	chr5.88853474.A.G	independent_variable	0,006501529	0,004560542	1,425604288	0,154578007
ADV5	chr5.88853509.G.A	independent_variable	0,006501529	0,004560542	1,425604288	0,154578007
ADV5	chr5.88854472.G.A	independent_variable	0,006501529	0,004560542	1,425604288	0,154578007
ADV5	chr5.88855175.T.A	independent_variable	0,006501529	0,004560542	1,425604288	0,154578007
ADV5	chr5.88856385.T.C	independent_variable	0,006501529	0,004560542	1,425604288	0,154578007
ADV5	chr5.88856757.C.A	independent_variable	0,006501529	0,004560542	1,425604288	0,154578007
ADV5	chr5.88858057.C.T	independent_variable	0,006501529	0,004560542	1,425604288	0,154578007
ADV5	chr5.104686538.C.G	independent_variable	0,006690593	0,004696264	1,424662976	0,154849934
ADV5	chr5.104709685.C.A	independent_variable	0,006490008	0,004556198	1,424435101	0,154893942
ADV5	chr5.88563112.T.G	independent_variable	-0,00737326	0,005200151	-1,417893384	0,156849816
ADV4	chr1.43574179.A.G	independent_variable	-0,000717717	0,000507371	-1,414579909	0,157774511
ADV1	chr20.21277415.A.G	independent_variable	-0,022554271	0,015977421	-1,411634037	0,158665158
ADV2	chr20.21156917.A.G	independent_variable	0,001588163	0,001126504	1,409815692	0,159182867
ADV5	chr5.104672432.G.A	independent_variable	0,006688831	0,004750797	1,407938677	0,159749069
ADV5	chr12.89378126.T.C	independent_variable	0,007491867	0,005323178	1,407404871	0,159987142
ADV3	chr5.104590343.C.T	independent_variable	-0,027527836	0,019567207	-1,406835231	0,160098751
ADV2	chr3.20706828.T.G	independent_variable	-0,001625687	0,001157973	-1,403908039	0,161009457

ADV3	chr5.104585069.C.A	independent_variable	-0,027494515	0,019589382	-1,40354174	0,161081096
ADV5	chr12.89382507.T.C	independent_variable	0,007430266	0,005314799	1,398033356	0,162779714
ADV5	chr5.88633148.G.T	independent_variable	-0,007135866	0,005109083	-1,396701906	0,163124105
ADV4	chr5.104646025.T.G	independent_variable	0,000738069	0,000528982	1,39526319	0,163518642
ADV5	chr12.89377735.T.C	independent_variable	0,007392719	0,005306059	1,393259763	0,164215579
ADV1	chr5.104597772.C.T	independent_variable	0,025393245	0,018237377	1,392373698	0,164429413
ADV5	chr5.104698439.C.T	independent_variable	0,006510509	0,004676816	1,392081309	0,164484944
ADV5	chr5.104678081.G.A	independent_variable	0,006585311	0,00473281	1,391416552	0,164694107
ADV1	chr20.21283529.T.C	independent_variable	-0,022194077	0,015973644	-1,389418553	0,165309427
ADV5	chr5.104482711.A.G	independent_variable	-0,006276942	0,004524824	-1,387223474	0,165943781
ADV2	chr20.21170737.G.A	independent_variable	0,001524931	0,001100807	1,38528477	0,166555938
ADV2	chr4.111485805.C.T	independent_variable	-0,002613156	0,001886663	-1,385067741	0,166634927
ADV2	chr11.28671984.T.G	independent_variable	-0,00134918	0,000974316	-1,384745657	0,166722956
ADV2	chr11.28672893.C.G	independent_variable	-0,00134918	0,000974316	-1,384745657	0,166722956
ADV2	chr20.21038664.T.C	independent_variable	0,001425229	0,001031601	1,381569213	0,167700749
ADV3	chr1.43843195.A.G	independent_variable	-0,025230545	0,018292577	-1,379277762	0,168395266
ADV2	chr20.21158348.G.A	independent_variable	0,001556894	0,001129006	1,37899545	0,168480024
ADV2	chr20.21158611.G.A	independent_variable	0,001556894	0,001129006	1,37899545	0,168480024
ADV2	chr20.21158741.C.G	independent_variable	0,001556894	0,001129006	1,37899545	0,168480024
ADV2	chr20.21159530.G.A	independent_variable	0,001556894	0,001129006	1,37899545	0,168480024
ADV2	chr20.21159701.C.T	independent_variable	0,001556894	0,001129006	1,37899545	0,168480024
ADV2	chr20.21161712.T.C	independent_variable	0,001556884	0,001129006	1,378986821	0,168482683
ADV2	chr3.20703004.G.A	independent_variable	-0,001601894	0,00116144	-1,379231634	0,168483486
ADV2	chr20.21157726.C.A	independent_variable	0,00154944	0,001129181	1,372180534	0,170589906
ADV5	chr5.104707455.C.T	independent_variable	0,00640723	0,004677087	1,369918904	0,171295575
ADV1	chr1.43879620.G.A	independent_variable	-0,024223514	0,017733296	-1,365990532	0,172560964
ADV5	chr1.43574179.A.G	independent_variable	0,00655029	0,004820085	1,358957277	0,174733809
ADV1	chr20.21281046.C.T	independent_variable	-0,021584525	0,015958283	-1,352559372	0,176792305
ADV2	chr3.20700799.C.T	independent_variable	-0,001584862	0,001171947	-1,352332463	0,176927689
ADV2	chr20.21161882.C.G	independent_variable	0,001531013	0,001132957	1,351341902	0,177165736
ADV5	chr4.111485805.C.T	independent_variable	0,0118546	0,008786937	1,349116365	0,17789591
ADV5	chr5.88861735.T.C	independent_variable	0,006116946	0,004538887	1,347675151	0,178328014
ADV4	chr5.88885292.T.G	independent_variable	0,000804296	0,000596802	1,347676621	0,17837889
ADV5	chr20.21201749.T.C	independent_variable	-0,006918959	0,005142338	-1,345488983	0,179064097
ADV2	chr3.20695381.T.C	independent_variable	-0,001536724	0,001145454	-1,341584539	0,180371764
ADV4	chr1.43605550.G.A	independent_variable	0,000733507	0,000549	1,336078149	0,182091747
ADV5	chr20.21428075.A.G	independent_variable	-0,006375448	0,004774928	-1,335192545	0,182379051
ADV5	chr20.21436041.T.G	independent_variable	-0,006375448	0,004774928	-1,335192545	0,182379051
ADV5	chr20.21439854.C.T	independent_variable	-0,006375448	0,004774928	-1,335192545	0,182379051
ADV5	chr20.21440237.T.C	independent_variable	-0,006375448	0,004774928	-1,335192545	0,182379051
ADV5	chr20.21444512.T.A	independent_variable	-0,006375448	0,004774928	-1,335192545	0,182379051
ADV5	chr20.21444955.G.C	independent_variable	-0,006375448	0,004774928	-1,335192545	0,182379051
ADV5	chr20.21449932.G.A	independent_variable	-0,006375448	0,004774928	-1,335192545	0,182379051
ADV1	chr20.21374422.T.A	independent_variable	-0,021094266	0,01580389	-1,33475146	0,182536248
ADV1	chr20.21375208.A.G	independent_variable	-0,021094266	0,01580389	-1,33475146	0,182536248
ADV1	chr20.21277855.T.C	independent_variable	-0,021326068	0,015985724	-1,334069526	0,182774146
ADV1	chr20.21277966.T.C	independent_variable	-0,021326068	0,015985724	-1,334069526	0,182774146
ADV3	chr5.88579561.C.T	independent_variable	-0,02652971	0,019932555	-1,330973866	0,183809089
ADV3	chr5.88584754.A.G	independent_variable	-0,02652971	0,019932555	-1,330973866	0,183809089
ADV3	chr5.88586361.A.G	independent_variable	-0,02652971	0,019932555	-1,330973866	0,183809089
ADV1	chr20.21345460.T.C	independent_variable	-0,020958904	0,015782417	-1,32799081	0,184757103
ADV1	chr20.21346867.T.G	independent_variable	-0,020958904	0,015782417	-1,32799081	0,184757103
ADV1	chr20.21349142.T.C	independent_variable	-0,020958904	0,015782417	-1,32799081	0,184757103
ADV1	chr20.21350734.G.A	independent_variable	-0,020958904	0,015782417	-1,32799081	0,184757103
ADV3	chr1.43848594.C.T	independent_variable	-0,02413067	0,018179721	-1,32733996	0,184968802
ADV5	chr1.43579857.G.A	independent_variable	0,006345361	0,00478228	1,326848475	0,185121682
ADV5	chr1.43583485.G.A	independent_variable	0,006345361	0,00478228	1,326848475	0,185121682
ADV5	chr1.43585959.G.A	independent_variable	0,006345361	0,00478228	1,326848475	0,185121682
ADV5	chr1.43586100.T.C	independent_variable	0,006345361	0,00478228	1,326848475	0,185121682
ADV4	chr3.20680326.A.G	independent_variable	-0,000640044	0,000483366	-1,324141503	0,186021481
ADV4	chr3.20680427.C.T	independent_variable	-0,000640044	0,000483366	-1,324141503	0,186021481
ADV4	chr3.20683524.C.A	independent_variable	-0,000640044	0,000483366	-1,324141503	0,186021481
ADV3	chr5.88567792.C.T	independent_variable	-0,026241695	0,019855436	-1,321637829	0,186896814
ADV3	chr5.88574430.T.C	independent_variable	-0,026241695	0,019855436	-1,321637829	0,186896814
ADV3	chr5.88577805.C.T	independent_variable	-0,026241695	0,019855436	-1,321637829	0,186896814
ADV2	chr4.111582716.G.A	independent_variable	-0,002596695	0,001965983	-1,320812658	0,187163234
ADV2	chr4.111500989.G.A	independent_variable	-0,002439358	0,001849377	-1,3190158	0,187755752
ADV3	chr12.89368722.C.G	independent_variable	-0,02631674	0,020011092	-1,315107652	0,189097728
ADV3	chr12.89369752.C.T	independent_variable	-0,02631674	0,020011092	-1,315107652	0,189097728
ADV1	chr5.88526854.T.A	independent_variable	-0,025133815	0,019123331	-1,314301103	0,189366121
ADV2	chr4.111251830.C.A	independent_variable	-0,002308161	0,00175698	-1,313709146	0,189518974

ADV1	chr12.89378126.T.C	independent_variable	0,023764242	0,018120658	1,311444758	0,190366033
ADV5	chr5.88636991.G.T	independent_variable	-0,006803055	0,005213477	-1,304897901	0,192529047
ADV3	chr12.89381252.T.G	independent_variable	-0,026216448	0,020093822	-1,30470191	0,192619543
ADV5	chr20.21456154.A.G	independent_variable	-0,006268184	0,004815771	-1,301595176	0,193617095
ADV5	chr20.21456675.T.C	independent_variable	-0,006268184	0,004815771	-1,301595176	0,193617095
ADV2	chr20.21165647.A.G	independent_variable	0,001443582	0,001109851	1,300699558	0,193933029
ADV2	chr20.21165828.A.T	independent_variable	0,001443582	0,001109851	1,300699558	0,193933029
ADV2	chr20.21166369.A.G	independent_variable	0,001443582	0,001109851	1,300699558	0,193933029
ADV2	chr20.21166767.A.G	independent_variable	0,001443582	0,001109851	1,300699558	0,193933029
ADV2	chr20.21168043.G.T	independent_variable	0,001443582	0,001109851	1,300699558	0,193933029
ADV1	chr5.104642625.G.A	independent_variable	0,023802851	0,018319624	1,299308918	0,194442761
ADV5	chr20.21074404.T.C	independent_variable	-0,006473883	0,004983571	-1,299044928	0,194498727
ADV4	chr1.43563242.G.A	independent_variable	-0,000664741	0,000512545	-1,296940894	0,19521057
ADV3	chr20.21252915.A.G	independent_variable	0,021934139	0,016948355	1,294175079	0,196180517
ADV2	chr3.20696597.A.G	independent_variable	-0,001478958	0,001142713	-1,294251144	0,196206236
ADV2	chr3.20698841.C.G	independent_variable	-0,001478958	0,001142713	-1,294251144	0,196206236
ADV5	chr20.21457201.T.G	independent_variable	-0,006233018	0,00482196	-1,292631709	0,196699585
ADV5	chr20.21463034.C.G	independent_variable	-0,006233018	0,00482196	-1,292631709	0,196699585
ADV5	chr20.21465660.G.T	independent_variable	-0,006233018	0,00482196	-1,292631709	0,196699585
ADV1	chr5.88601453.A.C	independent_variable	0,023782683	0,018424101	1,290846297	0,197370686
ADV1	chr20.21371598.A.G	independent_variable	-0,020172612	0,015640218	-1,28979092	0,197671304
ADV1	chr12.89377735.T.C	independent_variable	0,023306288	0,018077147	1,289268074	0,197954226
ADV1	chr12.89382507.T.C	independent_variable	0,023180453	0,018106947	1,280196625	0,201124
ADV4	chr2.144956787.A.C	independent_variable	-0,000633544	0,000496118	-1,277002518	0,202173478
ADV3	chr1.43896951.G.A	independent_variable	0,023772048	0,018656923	1,274167621	0,203195917
ADV5	chr1.43543362.C.T	independent_variable	0,006791844	0,005330718	1,274095635	0,203296011
ADV2	chr20.21166199.T.C	independent_variable	0,0014174	0,001113788	1,27259412	0,203727559
ADV2	chr20.21169627.G.C	independent_variable	0,001417393	0,001113788	1,272588388	0,203729592
ADV1	chr20.21390733.A.G	independent_variable	-0,020096712	0,015810545	-1,2710955	0,204257565
ADV5	chr5.88864537.C.T	independent_variable	0,005766025	0,004541757	1,269558143	0,204805606
ADV3	chr5.104629522.A.C	independent_variable	-0,024623552	0,01939929	-1,269301695	0,204933578
ADV1	chr5.104590741.T.A	independent_variable	0,023034735	0,018168992	1,267804828	0,205458904
ADV1	chr5.104600642.T.A	independent_variable	0,023034735	0,018168992	1,267804828	0,205458904
ADV2	chr20.21167328.G.C	independent_variable	0,001404448	0,001112565	1,262351532	0,207383846
ADV2	chr20.21170255.A.G	independent_variable	0,001404448	0,001112565	1,262351532	0,207383846
ADV2	chr20.21170684.G.A	independent_variable	0,001404448	0,001112565	1,262351532	0,207383846
ADV2	chr20.21170688.A.G	independent_variable	0,001404448	0,001112565	1,262351532	0,207383846
ADV2	chr20.21170708.C.T	independent_variable	0,001404448	0,001112565	1,262351532	0,207383846
ADV2	chr20.21171212.C.T	independent_variable	0,001404448	0,001112565	1,262351532	0,207383846
ADV2	chr20.21171591.G.A	independent_variable	0,001404448	0,001112565	1,262351532	0,207383846
ADV2	chr20.21172102.G.A	independent_variable	0,001404448	0,001112565	1,262351532	0,207383846
ADV2	chr20.21172498.G.A	independent_variable	0,001404448	0,001112565	1,262351532	0,207383846
ADV2	chr20.21173053.G.A	independent_variable	0,001404448	0,001112565	1,262351532	0,207383846
ADV2	chr20.21173632.G.C	independent_variable	0,001404448	0,001112565	1,262351532	0,207383846
ADV2	chr20.21173847.G.A	independent_variable	0,001404448	0,001112565	1,262351532	0,207383846
ADV4	chr5.88600784.C.T	independent_variable	-0,000675646	0,000536624	-1,259068876	0,208598872
ADV1	chr20.21371667.C.T	independent_variable	-0,01988703	0,015811002	-1,257796991	0,209024345
ADV1	chr20.21373277.C.T	independent_variable	-0,01988703	0,015811002	-1,257796991	0,209024345
ADV2	chr1.43547252.C.T	independent_variable	0,001530148	0,001218386	1,255881195	0,209776427
ADV1	chr20.21372841.G.C	independent_variable	-0,019870126	0,015843417	-1,254156561	0,210344284
ADV3	chr5.104554297.G.C	independent_variable	-0,021611336	0,017233001	-1,254066859	0,210386624
ADV1	chr20.21351811.A.G	independent_variable	-0,019783678	0,015783376	-1,253450327	0,210598715
ADV1	chr20.21352024.G.C	independent_variable	-0,019783678	0,015783376	-1,253450327	0,210598715
ADV1	chr20.21353013.G.C	independent_variable	-0,019783678	0,015783376	-1,253450327	0,210598715
ADV1	chr20.21353030.G.A	independent_variable	-0,019783678	0,015783376	-1,253450327	0,210598715
ADV1	chr20.21359219.A.G	independent_variable	-0,019783678	0,015783376	-1,253450327	0,210598715
ADV1	chr20.21361740.T.C	independent_variable	-0,019783678	0,015783376	-1,253450327	0,210598715
ADV4	chr1.43557944.G.A	independent_variable	0,000661379	0,000528074	1,252436384	0,210957073
ADV4	chr1.43568097.C.A	independent_variable	0,000693559	0,000553808	1,252345246	0,21099538
ADV4	chr1.43606749.A.G	independent_variable	0,000663401	0,000530198	1,25123226	0,211388552
ADV3	chr5.88574395.G.A	independent_variable	-0,024450131	0,019548069	-1,250769663	0,211613004
ADV4	chr16.61781742.A.G	independent_variable	-0,00067122	0,000537372	-1,249079192	0,212183368
ADV1	chr5.104482711.A.G	independent_variable	0,019550617	0,015666762	1,247904103	0,212605027
ADV4	chr1.43596488.C.T	independent_variable	-0,000639004	0,000512581	-1,246641325	0,213069524
ADV5	chr1.43634413.G.A	independent_variable	-0,0060246	0,004833496	-1,246427011	0,213146097
ADV3	chr1.43885226.G.T	independent_variable	0,023247289	0,018668961	1,245237439	0,213628913
ADV2	chr1.43604361.A.G	independent_variable	-0,001230984	0,000988961	-1,244723857	0,213833161
ADV3	chr1.43903763.G.A	independent_variable	0,022984184	0,018477355	1,243910942	0,214110565
ADV1	chr5.104568525.A.G	independent_variable	0,020640511	0,016635484	1,240752077	0,215285077
ADV4	chr1.43560369.A.G	independent_variable	-0,000637733	0,00051627	-1,235271167	0,217273812
ADV1	chr5.104623644.A.G	independent_variable	0,022768832	0,018467032	1,232944865	0,218186311

ADV4	chr5.104702059.G.A	independent_variable	0,000642225	0,000521056	1,232546464	0,218285371
ADV5	chr1.43549064.C.T	independent_variable	0,006627233	0,005381609	1,23145932	0,218800051
ADV3	chr20.21260820.G.A	independent_variable	0,020855917	0,016955396	1,230045991	0,21924256
ADV4	chr1.43586107.G.A	independent_variable	-0,000631729	0,000513995	-1,229058288	0,219586247
ADV1	chr20.21372771.A.C	independent_variable	-0,019509554	0,01589166	-1,2276599	0,220127979
ADV5	chr1.43830767.C.G	independent_variable	-0,006206956	0,005071354	-1,223924994	0,22159502
ADV4	chr1.43560418.C.T	independent_variable	0,000656192	0,000536542	1,223002857	0,221869079
ADV4	chr1.43556632.A.C	independent_variable	0,000658469	0,000538882	1,221917473	0,222276943
ADV1	chr20.21379890.A.G	independent_variable	-0,019290014	0,015796026	-1,221194095	0,222559619
ADV1	chr20.21388294.G.T	independent_variable	-0,019290014	0,015796026	-1,221194095	0,222559619
ADV5	chr1.43509093.G.T	independent_variable	0,006859393	0,005616851	1,221216932	0,222622709
ADV5	chr1.43510950.G.C	independent_variable	0,006859393	0,005616851	1,221216932	0,222622709
ADV4	chr1.43592796.C.T	independent_variable	-0,000626417	0,000513343	-1,220269665	0,222895284
ADV4	chr1.43593571.G.A	independent_variable	-0,000626417	0,000513343	-1,220269665	0,222895284
ADV4	chr1.43594812.G.A	independent_variable	-0,000626417	0,000513343	-1,220269665	0,222895284
ADV5	chr1.43610348.G.A	independent_variable	-0,005926394	0,004859733	-1,219489703	0,223186846
ADV4	chr1.43577721.G.A	independent_variable	-0,000623407	0,000511355	-1,219126554	0,223333399
ADV1	chr5.104608237.C.G	independent_variable	0,022402773	0,018394376	1,217914231	0,223837957
ADV5	chr4.111500989.G.A	independent_variable	0,010470084	0,008618096	1,214895217	0,224968221
ADV4	chr1.43903772.G.A	independent_variable	0,000627714	0,000517115	1,213875733	0,225353722
ADV1	chr20.21268900.C.T	independent_variable	-0,019278339	0,015914847	-1,21134301	0,226319508
ADV2	chr16.61749670.C.T	independent_variable	0,001337431	0,00110567	1,209610711	0,226964536
ADV5	chr1.43631859.T.C	independent_variable	-0,005890454	0,004870342	-1,20945394	0,227016751
ADV4	chr1.43783814.A.G	independent_variable	0,000664238	0,000549583	1,208621221	0,227350409
ADV1	chr5.104591878.C.T	independent_variable	0,022016732	0,018219869	1,208391316	0,227470254
ADV5	chr1.43546066.G.A	independent_variable	0,006510191	0,00538781	1,20831857	0,227566456
ADV3	chr5.104596006.G.A	independent_variable	-0,023345966	0,019340187	-1,207122059	0,227958178
ADV4	chr1.43585185.T.C	independent_variable	0,000645018	0,000536263	1,202802571	0,229582777
ADV4	chr1.43584276.C.T	independent_variable	-0,00061906	0,000514767	-1,202602194	0,229658315
ADV4	chr1.43585903.A.G	independent_variable	-0,00061906	0,000514767	-1,202602194	0,229658315
ADV4	chr1.43586163.A.C	independent_variable	-0,00061906	0,000514767	-1,202602194	0,229658315
ADV2	chr16.61753223.C.T	independent_variable	0,001321244	0,001099499	1,201678141	0,230018923
ADV2	chr16.61755912.G.C	independent_variable	0,001324549	0,001104443	1,199291201	0,230946609
ADV5	chr20.21422129.C.A	independent_variable	-0,005555584	0,004650456	-1,194632046	0,232758195
ADV1	chr5.104610505.A.T	independent_variable	0,021992166	0,018409772	1,194591998	0,232819715
ADV2	chr16.61758377.A.G	independent_variable	0,001308494	0,001098284	1,191398638	0,234023427
ADV4	chr1.43566122.G.C	independent_variable	-0,00059955	0,000504439	-1,188547649	0,235146016
ADV4	chr1.43685962.T.G	independent_variable	-0,000633832	0,000533949	-1,187063885	0,235724357
ADV1	chr5.104609425.A.G	independent_variable	0,021835139	0,018491966	1,180790575	0,238255281
ADV5	chr5.104659667.A.G	independent_variable	0,005380176	0,004587814	1,172710104	0,241425335
ADV5	chr5.88551455.T.A	independent_variable	0,0057575	0,004909225	1,172791896	0,241463386
ADV4	chr5.88563112.T.G	independent_variable	-0,000642257	0,00054771	-1,172621479	0,241510499
ADV2	chr7.114350297.C.T	independent_variable	-0,001401534	0,001198564	-1,16934426	0,242809047
ADV4	chr1.43592279.T.C	independent_variable	0,000633242	0,000542371	1,167543493	0,243510063
ADV2	chr1.43903763.G.A	independent_variable	-0,001282821	0,001099499	-1,166732861	0,243870014
ADV4	chr1.43579857.G.A	independent_variable	-0,000591644	0,000509859	-1,160405665	0,246398343
ADV4	chr1.43583485.G.A	independent_variable	-0,000591644	0,000509859	-1,160405665	0,246398343
ADV4	chr1.43585959.G.A	independent_variable	-0,000591644	0,000509859	-1,160405665	0,246398343
ADV4	chr1.43586100.T.C	independent_variable	-0,000591644	0,000509859	-1,160405665	0,246398343
ADV3	chr1.43879620.G.A	independent_variable	0,021924824	0,01891398	1,159186161	0,246938059
ADV1	chr5.104608319.G.T	independent_variable	0,021356678	0,018447047	1,157728863	0,247535274
ADV4	chr1.43569801.G.A	independent_variable	-0,000581187	0,000504799	-1,15132279	0,250116635
ADV3	chr5.104642625.G.A	independent_variable	-0,022395684	0,019463847	-1,150629901	0,25044006
ADV5	chr1.43610798.G.A	independent_variable	-0,005638656	0,004903166	-1,150003155	0,250650667
ADV4	chr5.88587685.T.C	independent_variable	-0,000620511	0,000542646	-1,143490971	0,253381981
ADV2	chr11.28648570.G.T	independent_variable	-0,001138027	0,0009959	-1,14271261	0,253678777
ADV2	chr11.28648575.T.A	independent_variable	-0,001138027	0,0009959	-1,14271261	0,253678777
ADV2	chr4.111267775.T.C	independent_variable	-0,002111146	0,00184974	-1,141320301	0,254257968
ADV4	chr1.43584333.G.A	independent_variable	0,000619814	0,000543534	1,140339519	0,254654162
ADV4	chr1.43584356.A.C	independent_variable	0,000619814	0,000543534	1,140339519	0,254654162
ADV4	chr1.43586706.T.G	independent_variable	0,000619814	0,000543534	1,140339519	0,254654162
ADV1	chr5.104609477.C.T	independent_variable	0,020894913	0,0183599	1,138073325	0,255641511
ADV5	chr5.88865600.T.C	independent_variable	0,005196903	0,004568214	1,137622586	0,255805054
ADV1	chr1.43887565.G.A	independent_variable	-0,018956959	0,016673697	-1,136937953	0,256102346
ADV4	chr3.20680838.A.C	independent_variable	-0,000549023	0,000483561	-1,135374802	0,256726695
ADV1	chr20.21270205.T.C	independent_variable	-0,01805476	0,015921953	-1,133953854	0,257341466
ADV4	chr5.88588020.G.C	independent_variable	-0,000616116	0,000543366	-1,13388825	0,257385867
ADV4	chr5.104676602.C.G	independent_variable	0,000621065	0,000548817	1,131642784	0,258338157
ADV4	chr1.43599477.C.T	independent_variable	-0,000581336	0,000513852	-1,131329748	0,258418497
ADV1	chr1.43872903.A.C	independent_variable	-0,020066278	0,017746636	-1,130708878	0,258734176
ADV3	chr12.89364160.G.A	independent_variable	-0,022615581	0,0200133	-1,130027614	0,259019699

ADV3	chr12.89366967.A.G	independent_variable	-0,022615581	0,0200133	-1,130027614	0,259019699
ADV2	chr11.28669740.C.T	independent_variable	-0,001127713	0,000999024	-1,128814362	0,259493663
ADV3	chr5.104597772.C.T	independent_variable	-0,021811874	0,019329268	-1,128437625	0,259677379
ADV5	chr7.114430980.C.T	independent_variable	-0,005205929	0,004613961	-1,128299241	0,259714859
ADV2	chr11.28666860.G.A	independent_variable	-0,001124687	0,000997399	-1,127620304	0,259996368
ADV5	chr5.104554297.G.C	independent_variable	-0,005344551	0,004740697	-1,127376525	0,260108298
ADV5	chr1.43612713.T.C	independent_variable	-0,005488359	0,004871287	-1,126675317	0,260379278
ADV5	chr1.43613740.T.C	independent_variable	-0,005488359	0,004871287	-1,126675317	0,260379278
ADV3	chr20.21291449.A.T	independent_variable	0,01909917	0,016958303	1,126243037	0,260590263
ADV5	chr20.21412561.A.G	independent_variable	-0,005479552	0,004868708	-1,125463282	0,260889057
ADV1	chr1.43903772.G.A	independent_variable	-0,018605549	0,016573106	-1,122635002	0,262117679
ADV2	chr11.28632994.A.G	independent_variable	-0,001097533	0,000985394	-1,113801813	0,265868125
ADV2	chr11.28639271.A.G	independent_variable	-0,001097533	0,000985394	-1,113801813	0,265868125
ADV5	chr3.20627579.T.G	independent_variable	-0,005186291	0,004662484	-1,112345068	0,266495049
ADV5	chr5.104742532.A.C	independent_variable	0,005450277	0,004923933	1,106895098	0,268853039
ADV5	chr5.104746478.A.C	independent_variable	0,005450277	0,004923933	1,106895098	0,268853039
ADV1	chr5.104610781.C.T	independent_variable	0,02039359	0,018426297	1,106765534	0,26893287
ADV5	chr20.21270205.T.C	independent_variable	0,00524569	0,004746066	1,105271235	0,269558158
ADV4	chr4.111333132.A.G	independent_variable	0,000849474	0,000768738	1,105023788	0,269655437
ADV1	chr5.104611504.C.T	independent_variable	0,020271002	0,018360111	1,104078451	0,270097694
ADV1	chr5.104612344.C.T	independent_variable	0,020271002	0,018360111	1,104078451	0,270097694
ADV2	chr11.28631941.T.G	independent_variable	-0,001078779	0,00098021	-1,100559109	0,271586311
ADV2	chr11.28632083.T.C	independent_variable	-0,001078779	0,00098021	-1,100559109	0,271586311
ADV2	chr11.28632180.T.G	independent_variable	-0,001078779	0,00098021	-1,100559109	0,271586311
ADV2	chr11.28632479.C.T	independent_variable	-0,001078779	0,00098021	-1,100559109	0,271586311
ADV2	chr11.28655190.A.G	independent_variable	-0,001083812	0,000985164	-1,100133448	0,271774352
ADV2	chr11.28655717.T.C	independent_variable	-0,001083812	0,000985164	-1,100133448	0,271774352
ADV4	chr1.43575295.T.G	independent_variable	0,000569187	0,000518337	1,09810196	0,272655774
ADV4	chr1.43682497.T.C	independent_variable	-0,000588948	0,000536493	-1,097774377	0,272791369
ADV4	chr1.43686518.G.A	independent_variable	-0,000588948	0,000536493	-1,097774377	0,272791369
ADV4	chr1.43687044.G.A	independent_variable	-0,000588948	0,000536493	-1,097774377	0,272791369
ADV4	chr1.43687948.G.C	independent_variable	-0,000588948	0,000536493	-1,097774377	0,272791369
ADV4	chr1.43688808.G.C	independent_variable	-0,000588948	0,000536493	-1,097774377	0,272791369
ADV5	chr5.104739429.T.C	independent_variable	0,005411899	0,004930511	1,097634539	0,272875052
ADV1	chr5.104619660.T.C	independent_variable	0,020125706	0,018430439	1,091981912	0,27537657
ADV3	chr20.21294655.A.C	independent_variable	0,018350451	0,01684717	1,089230502	0,276557748
ADV1	chr16.61781742.A.G	independent_variable	0,019411872	0,017839329	1,088150314	0,277019621
ADV4	chr1.43600753.C.A	independent_variable	-0,000560732	0,000516002	-1,086684674	0,277661914
ADV3	chr20.21328174.G.A	independent_variable	0,018284519	0,016831394	1,086334184	0,277834486
ADV2	chr11.28658309.G.A	independent_variable	-0,001070356	0,000986669	-1,084818248	0,278497232
ADV5	chr5.104568525.A.G	independent_variable	-0,004915261	0,004537787	-1,083184668	0,279253801
ADV4	chr4.111295017.T.A	independent_variable	0,000869688	0,000804472	1,081066483	0,280155517
ADV4	chr4.111296367.C.T	independent_variable	0,000869688	0,000804472	1,081066483	0,280155517
ADV4	chr4.111306830.A.T	independent_variable	0,000869688	0,000804472	1,081066483	0,280155517
ADV5	chr20.21268900.C.T	independent_variable	0,005117987	0,004745049	1,078595225	0,281273127
ADV4	chr5.88605345.G.A	independent_variable	-0,000575959	0,00053403	-1,078514148	0,281323354
ADV4	chr5.88618013.G.A	independent_variable	-0,000575959	0,00053403	-1,078514148	0,281323354
ADV4	chr5.88618446.A.G	independent_variable	-0,000575959	0,00053403	-1,078514148	0,281323354
ADV4	chr5.88619663.G.T	independent_variable	-0,000575959	0,00053403	-1,078514148	0,281323354
ADV5	chr11.28580674.A.G	independent_variable	0,003576469	0,003316886	1,078261043	0,281425006
ADV4	chr4.111287592.T.C	independent_variable	0,000866235	0,000805247	1,075738194	0,282531053
ADV4	chr12.89378126.T.C	independent_variable	0,000622647	0,000578754	1,075839994	0,282566455
ADV3	chr20.21291031.G.C	independent_variable	0,01831547	0,017092105	1,07157483	0,284417866
ADV2	chr1.43872903.A.C	independent_variable	-0,001195094	0,001115757	-1,071105526	0,284652515
ADV4	chr1.43655886.C.G	independent_variable	-0,000553444	0,000517731	-1,068979167	0,285554029
ADV4	chr5.88624488.C.A	independent_variable	-0,000571521	0,00053475	-1,068762945	0,285692202
ADV3	chr20.21350903.T.G	independent_variable	0,018075256	0,016913635	1,068679578	0,285708967
ADV4	chr1.43614741.T.C	independent_variable	0,000583412	0,000546089	1,068345299	0,28584213
ADV4	chr1.43558813.A.G	independent_variable	0,000578515	0,000542094	1,067185101	0,286362494
ADV4	chr12.89382507.T.C	independent_variable	0,000615429	0,000577854	1,065025065	0,28742573
ADV2	chr11.28654592.G.A	independent_variable	-0,001057059	0,000992843	-1,064679477	0,287509489
ADV2	chr11.28646124.G.A	independent_variable	-0,001047705	0,000984695	-1,063989782	0,287818756
ADV2	chr11.28646393.C.T	independent_variable	-0,001047705	0,000984695	-1,063989782	0,287818756
ADV2	chr11.28646869.T.C	independent_variable	-0,001047705	0,000984695	-1,063989782	0,287818756
ADV2	chr11.28648417.T.C	independent_variable	-0,001047705	0,000984695	-1,063989782	0,287818756
ADV2	chr11.28649222.A.G	independent_variable	-0,001047705	0,000984695	-1,063989782	0,287818756
ADV2	chr11.28649587.T.C	independent_variable	-0,001047705	0,000984695	-1,063989782	0,287818756
ADV2	chr11.28649653.C.G	independent_variable	-0,001047705	0,000984695	-1,063989782	0,287818756
ADV2	chr11.28650803.C.G	independent_variable	-0,001047705	0,000984695	-1,063989782	0,287818756
ADV2	chr11.28654623.A.G	independent_variable	-0,001047705	0,000984695	-1,063989782	0,287818756
ADV3	chr20.21314871.G.T	independent_variable	0,017961321	0,016882097	1,063927145	0,287857408

ADV4	chr2.144962572.A.G	independent_variable	-0,000533717	0,000501813	-1,063577698	0,28801656
ADV3	chr20.21310893.C.T	independent_variable	0,017949479	0,016882643	1,063191285	0,288190598
ADV3	chr20.21311200.C.T	independent_variable	0,017949479	0,016882643	1,063191285	0,288190598
ADV3	chr20.21311590.G.C	independent_variable	0,017949479	0,016882643	1,063191285	0,288190598
ADV3	chr20.21312173.C.T	independent_variable	0,017949479	0,016882643	1,063191285	0,288190598
ADV3	chr20.21312391.G.A	independent_variable	0,017949479	0,016882643	1,063191285	0,288190598
ADV1	chr5.104614550.C.T	independent_variable	0,01956962	0,018418771	1,062482444	0,28853795
ADV5	chr20.21073855.C.T	independent_variable	-0,005048473	0,004766728	-1,059106693	0,290047675
ADV3	chr1.43887565.G.A	independent_variable	0,018734071	0,017717249	1,057391619	0,290837437
ADV3	chr3.20700799.C.T	independent_variable	0,020185227	0,019091819	1,057271061	0,290938025
ADV3	chr20.21313246.C.T	independent_variable	0,017771017	0,016853388	1,054447738	0,292168601
ADV4	chr12.89377735.T.C	independent_variable	0,00060826	0,000576959	1,054253092	0,292321685
ADV5	chr20.21400134.C.A	independent_variable	-0,005294808	0,005037224	-1,051136137	0,293674192
ADV4	chr1.43692458.C.G	independent_variable	-0,00056308	0,000535744	-1,051024291	0,293716675
ADV2	chr1.43896951.G.A	independent_variable	-0,001168032	0,001111267	-1,051081539	0,293728868
ADV3	chr20.21299182.T.G	independent_variable	0,017670787	0,016832654	1,049792072	0,294301362
ADV4	chr1.43549278.T.C	independent_variable	-0,000568333	0,00054403	-1,044671921	0,29674691
ADV5	chr11.28659176.C.A	independent_variable	0,005008036	0,004798802	1,043601389	0,297141171
ADV2	chr1.43879620.G.A	independent_variable	-0,001161235	0,00111329	-1,043066497	0,297425609
ADV4	chr1.43658317.C.T	independent_variable	-0,000540476	0,000518564	-1,042253597	0,297757928
ADV3	chr5.104623644.A.G	independent_variable	-0,020389527	0,019580306	-1,041328314	0,298235668
ADV3	chr20.21289658.C.T	independent_variable	0,017742346	0,017043977	1,04097457	0,29837865
ADV3	chr20.21291893.C.T	independent_variable	0,017742324	0,017043978	1,040973228	0,298379272
ADV2	chr1.43885226.G.T	independent_variable	-0,001155423	0,001112759	-1,038340826	0,299614278
ADV5	chr7.114381815.T.C	independent_variable	0,005141253	0,004952914	1,038025708	0,299726124
ADV2	chr16.61781742.A.G	independent_variable	0,001155121	0,001114322	1,036613359	0,300385261
ADV5	chr1.43563682.C.G	independent_variable	-0,005226392	0,005043856	-1,0361897	0,300588869
ADV4	chr1.43548609.T.C	independent_variable	-0,000561215	0,000543105	-1,033345999	0,302007064
ADV4	chr20.21195965.C.T	independent_variable	0,000560832	0,000543957	1,031022998	0,3030397
ADV4	chr1.43555785.C.G	independent_variable	0,000568879	0,000552102	1,030386774	0,303294937
ADV5	chr11.28662674.G.A	independent_variable	0,004925637	0,004787	1,028960943	0,30396299
ADV5	chr11.28664505.C.T	independent_variable	0,004925637	0,004787	1,028960943	0,30396299
ADV5	chr11.28666219.T.C	independent_variable	0,004925637	0,004787	1,028960943	0,30396299
ADV5	chr11.28668805.C.G	independent_variable	0,004925637	0,004787	1,028960943	0,30396299
ADV5	chr11.28669587.A.G	independent_variable	0,004925637	0,004787	1,028960943	0,30396299
ADV2	chr11.28645542.T.C	independent_variable	-0,001020499	0,000992308	-1,028408696	0,304230287
ADV4	chr1.43632835.G.A	independent_variable	-0,000535665	0,000521118	-1,027915178	0,304448236
ADV2	chr4.111309356.T.A	independent_variable	-0,00153936	0,001498514	-1,027257897	0,304769408
ADV4	chr5.88633148.G.T	independent_variable	-0,000548527	0,000534508	-1,026228658	0,30528102
ADV4	chr5.88636991.G.T	independent_variable	-0,000548527	0,000534508	-1,026228658	0,30528102
ADV2	chr3.20627579.T.G	independent_variable	-0,000892831	0,000870343	-1,025838034	0,305436782
ADV5	chr1.43685344.T.G	independent_variable	-0,005230738	0,005100416	-1,025551254	0,305558955
ADV3	chr20.21308964.A.G	independent_variable	0,017282074	0,016867532	1,024576362	0,306040426
ADV3	chr20.21315921.C.A	independent_variable	0,017282074	0,016867532	1,024576362	0,306040426
ADV2	chr11.28646269.A.G	independent_variable	-0,001014539	0,000990707	-1,024056186	0,306278567
ADV2	chr11.28632740.G.A	independent_variable	-0,000998668	0,000975828	-1,02340508	0,306579744
ADV2	chr11.28633321.G.A	independent_variable	-0,000998668	0,000975828	-1,02340508	0,306579744
ADV2	chr11.28633511.A.C	independent_variable	-0,000998668	0,000975828	-1,02340508	0,306579744
ADV2	chr11.28634125.G.T	independent_variable	-0,000998668	0,000975828	-1,02340508	0,306579744
ADV2	chr11.28634351.C.G	independent_variable	-0,000998668	0,000975828	-1,02340508	0,306579744
ADV2	chr11.28634462.T.G	independent_variable	-0,000998668	0,000975828	-1,02340508	0,306579744
ADV2	chr11.28635703.A.G	independent_variable	-0,000998668	0,000975828	-1,02340508	0,306579744
ADV2	chr11.28637410.G.A	independent_variable	-0,000998668	0,000975828	-1,02340508	0,306579744
ADV2	chr11.28638055.A.T	independent_variable	-0,000998668	0,000975828	-1,02340508	0,306579744
ADV2	chr11.28638658.T.C	independent_variable	-0,000998668	0,000975828	-1,02340508	0,306579744
ADV2	chr11.28644512.C.G	independent_variable	-0,000998668	0,000975828	-1,02340508	0,306579744
ADV2	chr11.28644517.G.A	independent_variable	-0,000998668	0,000975828	-1,02340508	0,306579744
ADV2	chr11.28644793.A.C	independent_variable	-0,000998668	0,000975828	-1,02340508	0,306579744
ADV5	chr1.43689712.A.G	independent_variable	-0,005223003	0,005117836	-1,020549108	0,307924257
ADV3	chr7.114482451.C.T	independent_variable	-0,01824534	0,017932517	-1,017444473	0,309398507
ADV3	chr20.21318093.T.C	independent_variable	0,017106632	0,01683838	1,015931031	0,310134682
ADV3	chr20.21318094.G.T	independent_variable	0,017106632	0,01683838	1,015931031	0,310134682
ADV3	chr20.21319392.G.A	independent_variable	0,017106632	0,01683838	1,015931031	0,310134682
ADV3	chr20.21320839.C.T	independent_variable	0,017106632	0,01683838	1,015931031	0,310134682
ADV3	chr20.21321927.C.T	independent_variable	0,017106632	0,01683838	1,015931031	0,310134682
ADV3	chr20.21321943.T.C	independent_variable	0,017106632	0,01683838	1,015931031	0,310134682
ADV3	chr20.21327058.G.A	independent_variable	0,017106632	0,01683838	1,015931031	0,310134682
ADV3	chr20.21329677.C.T	independent_variable	0,017106632	0,01683838	1,015931031	0,310134682
ADV4	chr1.43721141.A.T	independent_variable	-0,000544562	0,000536183	-1,015626808	0,310260284
ADV5	chr7.114384998.A.G	independent_variable	0,005002212	0,004957142	1,009091971	0,313387542
ADV3	chr5.104590741.T.A	independent_variable	-0,019426206	0,019255337	-1,008873891	0,313524067

ADV3	chr5.104600642.T.A	independent_variable	-0,019426206	0,019255337	-1,008873891	0,313524067
ADV5	chr1.43548902.A.T	independent_variable	0,005418765	0,005370529	1,008981713	0,313532945
ADV3	chr5.88601453.A.C	independent_variable	-0,019699183	0,019533727	-1,008470282	0,313730569
ADV5	chr11.28590845.G.A	independent_variable	0,003328038	0,003303727	1,007358691	0,314235495
ADV5	chr11.28592943.A.T	independent_variable	0,003328038	0,003303727	1,007358691	0,314235495
ADV5	chr11.28596075.C.A	independent_variable	0,003328038	0,003303727	1,007358691	0,314235495
ADV5	chr11.28596496.C.G	independent_variable	0,003328038	0,003303727	1,007358691	0,314235495
ADV5	chr11.28596864.G.A	independent_variable	0,003328038	0,003303727	1,007358691	0,314235495
ADV5	chr20.21038664.T.C	independent_variable	-0,004836244	0,004803271	-1,00686459	0,314470852
ADV4	chr5.88628064.T.G	independent_variable	-0,00053947	0,000535962	-1,00654536	0,314643095
ADV4	chr5.88630645.G.A	independent_variable	-0,00053947	0,000535962	-1,00654536	0,314643095
ADV5	chr11.28580626.C.A	independent_variable	0,003335608	0,003316284	1,005826955	0,314973005
ADV3	chr20.21283152.A.T	independent_variable	0,01705899	0,017028853	1,001769756	0,316927089
ADV3	chr20.21284820.G.A	independent_variable	0,01705899	0,017028853	1,001769756	0,316927089
ADV3	chr20.21285090.C.T	independent_variable	0,01705899	0,017028853	1,001769756	0,316927089
ADV3	chr20.21285802.C.T	independent_variable	0,01705899	0,017028853	1,001769756	0,316927089
ADV3	chr20.21286713.C.T	independent_variable	0,01705899	0,017028853	1,001769756	0,316927089
ADV3	chr20.21287631.G.A	independent_variable	0,01705899	0,017028853	1,001769756	0,316927089
ADV3	chr20.21290685.C.G	independent_variable	0,01705899	0,017028853	1,001769756	0,316927089
ADV3	chr20.21290915.A.G	independent_variable	0,01705899	0,017028853	1,001769756	0,316927089
ADV3	chr20.21290947.G.A	independent_variable	0,01705899	0,017028853	1,001769756	0,316927089
ADV3	chr20.21291136.G.T	independent_variable	0,01705899	0,017028853	1,001769756	0,316927089
ADV3	chr20.21292673.G.A	independent_variable	0,01705899	0,017028853	1,001769756	0,316927089
ADV5	chr5.104571636.G.A	independent_variable	0,004662644	0,004654863	1,001671684	0,316965397
ADV3	chr20.21299701.C.T	independent_variable	0,016768067	0,016830078	0,996315429	0,319560298
ADV2	chr11.28623283.A.G	independent_variable	-0,000979789	0,000984207	-0,995511053	0,319943772
ADV2	chr11.28623794.G.C	independent_variable	-0,000979789	0,000984207	-0,995511053	0,319943772
ADV2	chr11.28628859.C.T	independent_variable	-0,000979789	0,000984207	-0,995511053	0,319943772
ADV3	chr20.21293229.C.T	independent_variable	0,016699221	0,016784593	0,99491367	0,320242773
ADV3	chr20.21294030.G.A	independent_variable	0,016699221	0,016784593	0,99491367	0,320242773
ADV4	chr5.88629003.C.T	independent_variable	-0,000530156	0,000533558	-0,993623351	0,32088784
ADV4	chr20.21350903.T.G	independent_variable	0,000479292	0,000482573	0,993200559	0,321073272
ADV4	chr7.114403104.A.G	independent_variable	-0,000475883	0,00047963	-0,992185904	0,321569193
ADV3	chr5.104609425.A.G	independent_variable	-0,019438912	0,019595933	-0,991987059	0,321690457
ADV2	chr11.28624037.T.C	independent_variable	-0,000981551	0,000989581	-0,991884618	0,321709005
ADV2	chr11.28628989.A.G	independent_variable	-0,000981551	0,000989581	-0,991884618	0,321709005
ADV2	chr11.28629363.A.G	independent_variable	-0,000981551	0,000989581	-0,991884618	0,321709005
ADV3	chr7.114451698.G.A	independent_variable	-0,017585062	0,017741258	-0,991195901	0,322031407
ADV4	chr1.43652735.G.A	independent_variable	-0,000514344	0,000520265	-0,988618086	0,323290351
ADV2	chr11.28639315.G.T	independent_variable	-0,000963432	0,000975374	-0,987756149	0,323719427
ADV5	chr5.104565723.C.T	independent_variable	0,004597263	0,004654987	0,987599572	0,323805466
ADV5	chr5.104568065.G.A	independent_variable	0,004597263	0,004654987	0,987599572	0,323805466
ADV5	chr5.88574395.G.A	independent_variable	-0,005127196	0,00519474	-0,986997737	0,324128878
ADV2	chr1.43560418.C.T	independent_variable	0,001093044	0,001109419	0,985240538	0,324952955
ADV3	chr3.20696597.A.G	independent_variable	0,018423399	0,01870267	0,985067884	0,325091163
ADV3	chr3.20698841.C.G	independent_variable	0,018423399	0,01870267	0,985067884	0,325091163
ADV5	chr5.88497027.G.A	independent_variable	0,005039832	0,005125587	0,9832693	0,325964151
ADV4	chr1.43631767.G.T	independent_variable	-0,000514189	0,00052351	-0,982195915	0,326442373
ADV2	chr11.28628549.C.T	independent_variable	-0,00097094	0,000992716	-0,978064506	0,328492889
ADV2	chr11.28622330.G.A	independent_variable	-0,000962954	0,000984731	-0,977884842	0,328579108
ADV2	chr11.28622366.T.C	independent_variable	-0,000962954	0,000984731	-0,977884842	0,328579108
ADV2	chr11.28622395.T.C	independent_variable	-0,000962954	0,000984731	-0,977884842	0,328579108
ADV2	chr11.28622424.G.A	independent_variable	-0,000962954	0,000984731	-0,977884842	0,328579108
ADV2	chr11.28622491.T.G	independent_variable	-0,000962954	0,000984731	-0,977884842	0,328579108
ADV2	chr11.28622565.G.T	independent_variable	-0,000962954	0,000984731	-0,977884842	0,328579108
ADV2	chr11.28622745.C.A	independent_variable	-0,000962954	0,000984731	-0,977884842	0,328579108
ADV2	chr11.28623055.A.G	independent_variable	-0,000962954	0,000984731	-0,977884842	0,328579108
ADV2	chr11.28623079.A.G	independent_variable	-0,000962954	0,000984731	-0,977884842	0,328579108
ADV2	chr11.28623338.T.C	independent_variable	-0,000962954	0,000984731	-0,977884842	0,328579108
ADV2	chr11.28624151.G.A	independent_variable	-0,000962954	0,000984731	-0,977884842	0,328579108
ADV2	chr11.28624272.T.G	independent_variable	-0,000962954	0,000984731	-0,977884842	0,328579108
ADV2	chr11.28625064.T.A	independent_variable	-0,000962954	0,000984731	-0,977884842	0,328579108
ADV2	chr11.28625609.A.T	independent_variable	-0,000962954	0,000984731	-0,977884842	0,328579108
ADV2	chr11.28626408.G.T	independent_variable	-0,000962954	0,000984731	-0,977884842	0,328579108
ADV2	chr11.28627124.G.A	independent_variable	-0,000962954	0,000984731	-0,977884842	0,328579108
ADV2	chr11.28628635.A.G	independent_variable	-0,000962954	0,000984731	-0,977884842	0,328579108
ADV2	chr11.28629811.A.C	independent_variable	-0,000962954	0,000984731	-0,977884842	0,328579108
ADV2	chr11.28631449.A.G	independent_variable	-0,000962885	0,000984732	-0,977814513	0,328613864
ADV5	chr5.104576507.C.A	independent_variable	0,004452434	0,004557584	0,976928618	0,329051025
ADV5	chr11.28586227.G.A	independent_variable	0,003238097	0,003317729	0,975998075	0,329525423
ADV2	chr1.43509093.G.T	independent_variable	0,001191755	0,001224511	0,973250216	0,33093496

ADV2	chr1.43510950.G.C	independent_variable	0,001191755	0,001224511	0,973250216	0,33093496
ADV5	chr5.88567792.C.T	independent_variable	-0,005171134	0,005318175	-0,972351297	0,331348801
ADV5	chr5.88574430.T.C	independent_variable	-0,005171134	0,005318175	-0,972351297	0,331348801
ADV5	chr5.88577805.C.T	independent_variable	-0,005171134	0,005318175	-0,972351297	0,331348801
ADV5	chr1.43717869.G.T	independent_variable	-0,005003471	0,005147931	-0,971938242	0,331516425
ADV5	chr1.43720773.T.A	independent_variable	-0,005002441	0,005147911	-0,971741935	0,331614011
ADV5	chr5.104576413.A.C	independent_variable	0,004447721	0,004577096	0,971734374	0,331628497
ADV5	chr5.104646025.T.G	independent_variable	0,004810086	0,004951632	0,971414317	0,33178345
ADV4	chr1.43656802.T.G	independent_variable	-0,000506203	0,000521775	-0,97015543	0,332401753
ADV4	chr7.114416000.G.C	independent_variable	-0,000460702	0,000475114	-0,969666793	0,332659964
ADV3	chr20.21277415.A.G	independent_variable	0,016504607	0,017059954	0,967447308	0,333777227
ADV4	chr4.111311550.T.C	independent_variable	0,000728859	0,000753427	0,967392672	0,333793198
ADV4	chr4.111328737.T.G	independent_variable	0,000728859	0,000753427	0,967392672	0,333793198
ADV3	chr5.104608237.C.G	independent_variable	-0,018834091	0,019491889	-0,966252711	0,334390532
ADV4	chr7.114406314.C.G	independent_variable	-0,000462508	0,000479426	-0,964712216	0,33513826
ADV1	chr7.114522685.G.A	independent_variable	0,01573904	0,016328644	0,963891461	0,335558951
ADV4	chr5.104581838.T.A	independent_variable	0,000474277	0,000492711	0,962586966	0,336188892
ADV4	chr5.104582136.T.C	independent_variable	0,000474277	0,000492711	0,962586966	0,336188892
ADV3	chr20.21283529.T.C	independent_variable	0,016416468	0,017054136	0,962609206	0,336198074
ADV2	chr11.28582930.C.T	independent_variable	-0,000973187	0,0010111	-0,962502764	0,336248816
ADV2	chr11.28582122.A.G	independent_variable	-0,0009787	0,001017665	-0,961711293	0,336647723
ADV2	chr11.28585152.T.C	independent_variable	-0,0009787	0,001017665	-0,961711293	0,336647723
ADV2	chr11.28587183.C.G	independent_variable	-0,0009787	0,001017665	-0,961711293	0,336647723
ADV2	chr11.28593837.A.G	independent_variable	-0,0009787	0,001017665	-0,961711293	0,336647723
ADV2	chr11.28594152.A.G	independent_variable	-0,0009787	0,001017665	-0,961711293	0,336647723
ADV2	chr11.28594153.T.C	independent_variable	-0,0009787	0,001017665	-0,961711293	0,336647723
ADV2	chr11.28594704.T.C	independent_variable	-0,0009787	0,001017665	-0,961711293	0,336647723
ADV2	chr11.28597209.G.A	independent_variable	-0,0009787	0,001017665	-0,961711293	0,336647723
ADV4	chr4.111311007.T.C	independent_variable	0,000725219	0,000754183	0,961595637	0,336696277
ADV4	chr1.43718252.C.T	independent_variable	-0,000514268	0,000535125	-0,961025799	0,336970107
ADV5	chr11.28582930.C.T	independent_variable	0,003169622	0,003302027	0,959901959	0,337554996
ADV5	chr3.71450250.C.T	independent_variable	0,004616597	0,004810472	0,959697251	0,337644381
ADV2	chr11.28622320.A.G	independent_variable	-0,000944015	0,000983795	-0,959564239	0,337713765
ADV2	chr11.28624925.C.T	independent_variable	-0,000944015	0,000983795	-0,959564239	0,337713765
ADV2	chr11.28628323.A.G	independent_variable	-0,000948272	0,000990697	-0,957176339	0,338917978
ADV4	chr7.114350297.C.T	independent_variable	0,000538031	0,000562445	0,956592877	0,339224237
ADV5	chr5.104606092.C.A	independent_variable	0,004395534	0,00459845	0,955872862	0,339573906
ADV1	chr3.20700799.C.T	independent_variable	-0,017152412	0,01796603	-0,954713563	0,34021968
ADV4	chr1.43569422.A.G	independent_variable	0,000499057	0,000523213	0,953830524	0,340599386
ADV4	chr1.43716573.A.G	independent_variable	-0,000509331	0,000534483	-0,95294155	0,341046038
ADV5	chr5.104568407.A.G	independent_variable	0,004458659	0,004688118	0,951055088	0,342018239
ADV4	chr1.43885226.G.T	independent_variable	0,000512804	0,000540193	0,949298762	0,342928206
ADV4	chr7.114430980.C.T	independent_variable	-0,000469916	0,000495504	-0,948359987	0,343387588
ADV2	chr11.28622480.G.A	independent_variable	-0,000935274	0,000987784	-0,946840927	0,344154607
ADV5	chr5.104676602.C.G	independent_variable	-0,004754077	0,005023816	-0,946308015	0,344457921
ADV2	chr11.28585698.T.C	independent_variable	-0,00096434	0,001019262	-0,946115795	0,344535816
ADV5	chr5.88601453.A.C	independent_variable	-0,004992899	0,005277462	-0,946079618	0,344578111
ADV2	chr11.28580674.A.G	independent_variable	-0,000961997	0,001016912	-0,945998367	0,344595648
ADV3	chr5.104591878.C.T	independent_variable	-0,018240866	0,019306679	-0,944795664	0,345221361
ADV3	chr7.114531090.A.G	independent_variable	-0,017229032	0,018259276	-0,943576939	0,345831781
ADV5	chr5.104702059.G.A	independent_variable	0,004661115	0,004944419	0,942702104	0,346257994
ADV3	chr5.104610505.A.T	independent_variable	-0,018389159	0,019508686	-0,942613923	0,346339158
ADV4	chr7.114419101.C.T	independent_variable	-0,000447611	0,000474918	-0,942502099	0,346369959
ADV2	chr11.28590845.G.A	independent_variable	-0,000952792	0,001010989	-0,942435466	0,346411612
ADV2	chr11.28592943.A.T	independent_variable	-0,000952792	0,001010989	-0,942435466	0,346411612
ADV2	chr11.28596075.C.A	independent_variable	-0,000952792	0,001010989	-0,942435466	0,346411612
ADV2	chr11.28596496.C.G	independent_variable	-0,000952792	0,001010989	-0,942435466	0,346411612
ADV2	chr11.28596864.G.A	independent_variable	-0,000952792	0,001010989	-0,942435466	0,346411612
ADV5	chr5.104734216.A.G	independent_variable	0,004599486	0,00488265	0,942006072	0,346609203
ADV3	chr3.20703004.G.A	independent_variable	0,01783378	0,018962964	0,940453178	0,347471346
ADV4	chr1.43896951.G.A	independent_variable	0,000507088	0,000539459	0,939993472	0,347674792
ADV2	chr4.111311007.T.C	independent_variable	-0,001432143	0,001523731	-0,939891981	0,347706026
ADV5	chr5.88461101.C.T	independent_variable	-0,004775321	0,00510192	-0,935985083	0,34969771
ADV4	chr4.111309356.T.A	independent_variable	0,000694168	0,000742723	0,934626026	0,350408294
ADV2	chr1.43556632.A.C	independent_variable	0,001043805	0,001116833	0,934611063	0,350410413
ADV4	chr20.21038664.T.C	independent_variable	-0,000476021	0,000509561	-0,934178768	0,350647783
ADV2	chr11.28620721.G.A	independent_variable	-0,000897813	0,000961178	-0,934075274	0,350698596
ADV2	chr4.111311550.T.C	independent_variable	-0,001421502	0,00152234	-0,93376087	0,350856509
ADV2	chr4.111328737.T.G	independent_variable	-0,001421502	0,00152234	-0,93376087	0,350856509
ADV3	chr7.114522685.G.A	independent_variable	-0,016199228	0,017350464	-0,933648088	0,350929003
ADV5	chr7.114443495.C.G	independent_variable	-0,004250018	0,004552336	-0,933590496	0,350945979

ADV2	chr1.43560382.T.G	independent_variable	0,001041122	0,001115205	0,933570175	0,350947585
ADV2	chr1.43560509.A.C	independent_variable	0,001041122	0,001115205	0,933570175	0,350947585
ADV5	chr3.20687599.C.T	independent_variable	0,004212391	0,004513254	0,933337835	0,351060395
ADV2	chr11.28617583.T.C	independent_variable	-0,00090126	0,000965684	-0,933286406	0,351106927
ADV3	chr3.20695381.T.C	independent_variable	0,017472391	0,018747388	0,93199069	0,351814796
ADV1	chr7.114554560.T.G	independent_variable	0,015367244	0,016497961	0,931463258	0,35206172
ADV2	chr11.28620773.G.A	independent_variable	-0,000895295	0,000961929	-0,930729623	0,352425327
ADV3	chr5.104610781.C.T	independent_variable	-0,018127085	0,019522728	-0,928511872	0,35359465
ADV3	chr1.43872903.A.C	independent_variable	0,017566969	0,018926883	0,928149083	0,353790019
ADV1	chr7.114520816.G.A	independent_variable	0,01514133	0,016324823	0,927503499	0,354105629
ADV2	chr4.111333132.A.G	independent_variable	-0,001437746	0,001553723	-0,925355645	0,355206089
ADV2	chr11.28580626.C.A	independent_variable	-0,00093888	0,00101485	-0,92514188	0,35532709
ADV5	chr1.43726088.C.T	independent_variable	-0,0047238	0,005108948	-0,924613076	0,355580963
ADV5	chr1.43731274.C.T	independent_variable	-0,0047238	0,005108948	-0,924613076	0,355580963
ADV4	chr1.43707752.G.A	independent_variable	-0,00049012	0,000530861	-0,923254977	0,356285924
ADV4	chr1.43722794.A.C	independent_variable	-0,00049012	0,000530861	-0,923254977	0,356285924
ADV4	chr1.43723048.A.G	independent_variable	-0,00049012	0,000530861	-0,923254977	0,356285924
ADV3	chr20.21281046.C.T	independent_variable	0,0157277	0,017036267	0,923189332	0,356342547
ADV3	chr3.20706828.T.G	independent_variable	0,017437505	0,01890644	0,92230504	0,356844709
ADV5	chr1.43900578.G.A	independent_variable	-0,004984318	0,005406881	-0,921847135	0,357043339
ADV4	chr1.43879620.G.A	independent_variable	0,000500671	0,000544233	0,91995617	0,358042852
ADV5	chr5.104563895.T.C	independent_variable	0,004286537	0,004670998	0,917691961	0,359203152
ADV3	chr1.43903772.G.A	independent_variable	0,016192105	0,01765767	0,917001222	0,359572888
ADV3	chr20.21374422.T.A	independent_variable	0,015415195	0,016842728	0,915243351	0,360484069
ADV3	chr20.21375208.A.G	independent_variable	0,015415195	0,016842728	0,915243351	0,360484069
ADV5	chr4.111582716.G.A	independent_variable	0,008277465	0,009052527	0,91438163	0,360953585
ADV5	chr1.43728863.A.G	independent_variable	-0,004683362	0,005131435	-0,912680892	0,361818434
ADV5	chr1.43729682.T.C	independent_variable	-0,004683362	0,005131435	-0,912680892	0,361818434
ADV5	chr7.114429481.C.G	independent_variable	-0,004175635	0,004579702	-0,911770094	0,362313056
ADV5	chr7.114451789.C.A	independent_variable	-0,004146264	0,004549881	-0,911290566	0,362562122
ADV3	chr5.104608319.G.T	independent_variable	-0,017808408	0,019549541	-0,910937406	0,36277353
ADV4	chr7.114470513.C.T	independent_variable	0,000479536	0,000527034	0,909875954	0,363290884
ADV4	chr1.43552933.G.T	independent_variable	0,000495833	0,00054546	0,909016779	0,363748298
ADV2	chr11.28602326.G.T	independent_variable	-0,000946854	0,001042788	-0,908002263	0,364301713
ADV5	chr5.104566479.C.T	independent_variable	0,004179125	0,004606709	0,907182348	0,364727366
ADV5	chr5.104567257.C.T	independent_variable	0,004179125	0,004606709	0,907182348	0,364727366
ADV5	chr5.104572545.C.T	independent_variable	0,004179125	0,004606709	0,907182348	0,364727366
ADV5	chr5.104572709.A.G	independent_variable	0,004179125	0,004606709	0,907182348	0,364727366
ADV5	chr5.104573167.C.T	independent_variable	0,004179125	0,004606709	0,907182348	0,364727366
ADV5	chr5.104573847.C.T	independent_variable	0,004179125	0,004606709	0,907182348	0,364727366
ADV3	chr20.21345460.T.C	independent_variable	0,015248643	0,016813941	0,906904726	0,364872449
ADV3	chr20.21346867.T.G	independent_variable	0,015248643	0,016813941	0,906904726	0,364872449
ADV3	chr20.21349142.T.C	independent_variable	0,015248643	0,016813941	0,906904726	0,364872449
ADV3	chr20.21350734.G.A	independent_variable	0,015248643	0,016813941	0,906904726	0,364872449
ADV4	chr1.43565986.C.T	independent_variable	0,000476655	0,000525933	0,906303075	0,36518345
ADV4	chr1.43554060.C.T	independent_variable	-0,000462104	0,000510279	-0,905589151	0,365567969
ADV4	chr1.43556356.A.G	independent_variable	-0,000462104	0,000510279	-0,905589151	0,365567969
ADV5	chr1.43728545.T.G	independent_variable	-0,004647955	0,00513759	-0,90469569	0,366031565
ADV5	chr1.43729733.C.T	independent_variable	-0,004631439	0,005125767	-0,903560068	0,366633114
ADV5	chr5.88385177.A.G	independent_variable	-0,004718956	0,005239162	-0,900708209	0,368162043
ADV5	chr5.88389683.C.T	independent_variable	-0,004718956	0,005239162	-0,900708209	0,368162043
ADV4	chr7.114451789.C.A	independent_variable	-0,000439857	0,000488626	-0,900191753	0,368432517
ADV4	chr1.43563291.G.A	independent_variable	0,000471874	0,000525302	0,898290834	0,369433851
ADV5	chr5.88386213.G.A	independent_variable	-0,004675249	0,005220907	-0,895486051	0,370942444
ADV3	chr20.21277855.T.C	independent_variable	0,015265835	0,017066635	0,894484152	0,371483191
ADV3	chr20.21277966.T.C	independent_variable	0,015265835	0,017066635	0,894484152	0,371483191
ADV2	chr11.28619515.G.A	independent_variable	-0,000859695	0,000961465	-0,894151043	0,371654737
ADV2	chr11.28619698.G.A	independent_variable	-0,000859695	0,000961465	-0,894151043	0,371654737
ADV3	chr7.114520816.G.A	independent_variable	-0,015496157	0,017346487	-0,893331121	0,372102876
ADV1	chr1.43883733.G.A	independent_variable	-0,014970544	0,016797312	-0,891246394	0,373227391
ADV5	chr5.88397820.G.C	independent_variable	-0,004654789	0,005227701	-0,890408398	0,373659992
ADV5	chr5.88579561.C.T	independent_variable	-0,004735335	0,005331969	-0,88810252	0,37491607
ADV5	chr5.88584754.A.G	independent_variable	-0,004735335	0,005331969	-0,88810252	0,37491607
ADV5	chr5.88586361.A.G	independent_variable	-0,004735335	0,005331969	-0,88810252	0,37491607
ADV2	chr11.28621014.G.C	independent_variable	-0,00086053	0,000969389	-0,887703842	0,375113661
ADV3	chr7.114483253.C.T	independent_variable	-0,015792087	0,017813586	-0,88651928	0,375732401
ADV5	chr5.104569213.T.C	independent_variable	0,004085343	0,004613964	0,885430259	0,376329975
ADV5	chr5.104568333.A.G	independent_variable	0,004086735	0,004617227	0,885106007	0,376506203
ADV5	chr5.104568336.T.C	independent_variable	0,004086735	0,004617227	0,885106007	0,376506203
ADV5	chr20.21371598.A.G	independent_variable	0,003991864	0,004516725	0,883796081	0,377196717
ADV3	chr16.61781742.A.G	independent_variable	-0,016753384	0,018974146	-0,882958553	0,377655975

ADV2	chr1.43557944.G.A	independent_variable	0,00096975	0,001098718	0,882619321	0,377837646
ADV1	chr7.114508188.A.G	independent_variable	0,01433575	0,016244095	0,882520688	0,377912705
ADV5	chr5.104568136.A.G	independent_variable	0,004066901	0,004609668	0,882254602	0,378043674
ADV5	chr5.104563989.T.C	independent_variable	0,004066901	0,004609668	0,882254602	0,378043674
ADV5	chr5.104569324.T.A	independent_variable	0,004066901	0,004609668	0,882254602	0,378043674
ADV5	chr5.104569574.C.T	independent_variable	0,004066901	0,004609668	0,882254602	0,378043674
ADV5	chr5.104569709.A.C	independent_variable	0,004066901	0,004609668	0,882254602	0,378043674
ADV5	chr5.104570372.C.T	independent_variable	0,004066901	0,004609668	0,882254602	0,378043674
ADV5	chr5.104571905.G.A	independent_variable	0,004066901	0,004609668	0,882254602	0,378043674
ADV1	chr12.89368722.C.G	independent_variable	0,016589876	0,018803626	0,882270038	0,378069661
ADV1	chr12.89369752.C.T	independent_variable	0,016589876	0,018803626	0,882270038	0,378069661
ADV5	chr5.104620815.C.G	independent_variable	0,004358079	0,004941309	0,88196856	0,378189889
ADV5	chr5.104628884.G.A	independent_variable	0,004358079	0,004941309	0,88196856	0,378189889
ADV5	chr5.104636656.G.A	independent_variable	0,004358079	0,004941309	0,88196856	0,378189889
ADV3	chr7.114554560.T.G	independent_variable	-0,015418061	0,017541507	-0,878947342	0,379850346
ADV3	chr5.104609477.C.T	independent_variable	-0,017073303	0,019454876	-0,877584785	0,380595686
ADV1	chr3.20696597.A.G	independent_variable	-0,015442608	0,017604795	-0,877181903	0,380830341
ADV1	chr3.20698841.C.G	independent_variable	-0,015442608	0,017604795	-0,877181903	0,380830341
ADV5	chr7.114436339.C.G	independent_variable	-0,003981206	0,004567745	-0,871591307	0,383833603
ADV2	chr11.28620834.C.T	independent_variable	-0,000836527	0,00096029	-0,871118696	0,384091334
ADV1	chr12.89381252.T.G	independent_variable	0,016374108	0,018885095	0,867038668	0,386354041
ADV5	chr1.43641757.C.T	independent_variable	0,004368883	0,005039509	0,866926364	0,386367404
ADV5	chr16.61755912.G.C	independent_variable	-0,004439054	0,005129701	-0,865363058	0,387228145
ADV5	chr11.28658596.T.C	independent_variable	0,004141244	0,004790788	0,864418309	0,387749192
ADV2	chr20.21074404.T.C	independent_variable	0,000925058	0,001070373	0,86423951	0,387851017
ADV2	chr1.43558813.A.G	independent_variable	0,000943244	0,001092085	0,863709534	0,388128823
ADV4	chr7.114518899.G.A	independent_variable	0,000456878	0,000529479	0,862881267	0,388601302
ADV5	chr5.88456878.A.G	independent_variable	-0,00459226	0,005328117	-0,861891706	0,389145925
ADV5	chr5.88461004.C.T	independent_variable	-0,00459226	0,005328117	-0,861891706	0,389145925
ADV5	chr5.88467699.G.A	independent_variable	-0,00459226	0,005328117	-0,861891706	0,389145925
ADV5	chr5.88469954.T.C	independent_variable	-0,00459226	0,005328117	-0,861891706	0,389145925
ADV5	chr5.88475276.A.C	independent_variable	-0,00459226	0,005328117	-0,861891706	0,389145925
ADV5	chr5.88477838.A.G	independent_variable	-0,00459226	0,005328117	-0,861891706	0,389145925
ADV4	chr1.43550138.T.C	independent_variable	-0,000474572	0,000550584	-0,86194395	0,389188937
ADV5	chr1.43572014.G.A	independent_variable	0,00437912	0,005089144	0,860482616	0,389910679
ADV3	chr7.114508188.A.G	independent_variable	-0,014852872	0,017272661	-0,859906446	0,390246234
ADV4	chr5.104614550.C.T	independent_variable	0,000494829	0,000575843	0,85931249	0,390586618
ADV2	chr1.43883733.G.A	independent_variable	-0,00090773	0,001057109	-0,85869053	0,390924388
ADV5	chr16.61749670.C.T	independent_variable	-0,004401808	0,005135967	-0,857055423	0,391799433
ADV5	chr7.114403104.A.G	independent_variable	-0,004013629	0,004685596	-0,856588671	0,392067573
ADV4	chr20.21371598.A.G	independent_variable	0,000413957	0,000483745	0,8557343	0,392521509
ADV5	chr1.43617344.G.A	independent_variable	0,004341886	0,005082777	0,854235043	0,393354973
ADV4	chr5.104578822.T.C	independent_variable	0,000415004	0,000485857	0,854168849	0,393388111
ADV4	chr1.43549154.A.G	independent_variable	-0,000462934	0,000541907	-0,854267393	0,393419608
ADV4	chr7.114429481.C.G	independent_variable	-0,000419578	0,000491772	-0,853196702	0,393943814
ADV5	chr7.114407487.G.C	independent_variable	-0,004003261	0,00469695	-0,852310631	0,394435592
ADV2	chr11.28630347.G.C	independent_variable	-0,000838667	0,000984154	-0,852170597	0,394506504
ADV2	chr11.28586227.G.A	independent_variable	-0,000865433	0,001016174	-0,851659087	0,394800717
ADV4	chr1.43537286.G.A	independent_variable	0,00051069	0,000600104	0,851002461	0,395202259
ADV3	chr20.21390733.A.G	independent_variable	0,014339471	0,016854002	0,850805112	0,395265491
ADV5	chr7.114350297.C.T	independent_variable	0,004661715	0,005481413	0,8504586	0,395467701
ADV5	chr16.61753223.C.T	independent_variable	-0,004340801	0,005106574	-0,850041755	0,395681405
ADV3	chr5.104611504.C.T	independent_variable	-0,016497118	0,019461727	-0,847669767	0,397033244
ADV3	chr5.104612344.C.T	independent_variable	-0,016497118	0,019461727	-0,847669767	0,397033244
ADV4	chr7.114407487.G.C	independent_variable	-0,000407042	0,00048101	-0,846222894	0,397819157
ADV4	chr5.88567792.C.T	independent_variable	0,000474369	0,000561105	0,845419156	0,398283668
ADV4	chr5.88574430.T.C	independent_variable	0,000474369	0,000561105	0,845419156	0,398283668
ADV4	chr5.88577805.C.T	independent_variable	0,000474369	0,000561105	0,845419156	0,398283668
ADV3	chr20.21371667.C.T	independent_variable	0,014243415	0,016849087	0,845352345	0,398299237
ADV3	chr20.21373277.C.T	independent_variable	0,014243415	0,016849087	0,845352345	0,398299237
ADV3	chr1.43883733.G.A	independent_variable	0,015077861	0,017847661	0,84480879	0,398623277
ADV2	chr7.114508276.C.A	independent_variable	0,000871396	0,001032651	0,843843143	0,399154244
ADV5	chr5.88445076.T.C	independent_variable	-0,004465797	0,005297261	-0,843038995	0,399598001
ADV5	chr5.88468801.G.A	independent_variable	-0,004465797	0,005297261	-0,843038995	0,399598001
ADV3	chr20.21372841.G.C	independent_variable	0,014209353	0,01688361	0,841606315	0,400392268
ADV5	chr16.61758377.A.G	independent_variable	-0,004290924	0,005100668	-0,841247593	0,400583689
ADV5	chr5.104577563.A.C	independent_variable	0,003830948	0,004569879	0,838304163	0,402237087
ADV5	chr5.104577567.C.T	independent_variable	0,003830948	0,004569879	0,838304163	0,402237087
ADV5	chr5.104577620.T.A	independent_variable	0,003830948	0,004569879	0,838304163	0,402237087
ADV5	chr5.104578055.A.T	independent_variable	0,003830948	0,004569879	0,838304163	0,402237087
ADV5	chr5.104578313.C.T	independent_variable	0,003830948	0,004569879	0,838304163	0,402237087

ADV5	chr5.104578842.G.A	independent_variable	0,003830948	0,004569879	0,838304163	0,402237087
ADV5	chr5.104579057.A.G	independent_variable	0,003830948	0,004569879	0,838304163	0,402237087
ADV5	chr5.104579085.A.T	independent_variable	0,003830948	0,004569879	0,838304163	0,402237087
ADV5	chr5.104579135.T.G	independent_variable	0,003830948	0,004569879	0,838304163	0,402237087
ADV5	chr5.104579581.A.G	independent_variable	0,003830948	0,004569879	0,838304163	0,402237087
ADV5	chr5.104579742.G.A	independent_variable	0,003830948	0,004569879	0,838304163	0,402237087
ADV5	chr5.104580027.A.G	independent_variable	0,003830948	0,004569879	0,838304163	0,402237087
ADV5	chr5.104580648.T.C	independent_variable	0,003830948	0,004569879	0,838304163	0,402237087
ADV5	chr5.104580662.G.C	independent_variable	0,003830948	0,004569879	0,838304163	0,402237087
ADV5	chr5.104581130.A.G	independent_variable	0,003830948	0,004569879	0,838304163	0,402237087
ADV5	chr5.88455935.G.A	independent_variable	-0,00442128	0,00527781	-0,837711064	0,402580839
ADV5	chr5.88458988.C.T	independent_variable	-0,00442128	0,00527781	-0,837711064	0,402580839
ADV5	chr5.88467651.C.A	independent_variable	-0,00442128	0,00527781	-0,837711064	0,402580839
ADV5	chr5.88473876.C.T	independent_variable	-0,00442128	0,00527781	-0,837711064	0,402580839
ADV5	chr5.88477912.G.T	independent_variable	-0,00442128	0,00527781	-0,837711064	0,402580839
ADV4	chr1.43560985.G.C	independent_variable	-0,000462355	0,000551965	-0,837652963	0,402601085
ADV3	chr20.21351811.A.G	independent_variable	0,014073388	0,016820224	0,836694434	0,403144301
ADV3	chr20.21352024.G.C	independent_variable	0,014073388	0,016820224	0,836694434	0,403144301
ADV3	chr20.21353013.G.C	independent_variable	0,014073388	0,016820224	0,836694434	0,403144301
ADV3	chr20.21353030.G.A	independent_variable	0,014073388	0,016820224	0,836694434	0,403144301
ADV3	chr20.21359219.A.G	independent_variable	0,014073388	0,016820224	0,836694434	0,403144301
ADV3	chr20.21361740.T.C	independent_variable	0,014073388	0,016820224	0,836694434	0,403144301
ADV5	chr1.43604361.A.G	independent_variable	0,004318046	0,005170963	0,835056396	0,404096261
ADV3	chr7.114456791.A.T	independent_variable	-0,014919631	0,017881499	-0,834361323	0,404447722
ADV3	chr7.114464334.T.C	independent_variable	-0,014788953	0,017766921	-0,832386948	0,405559213
ADV5	chr5.104577501.A.G	independent_variable	0,003815301	0,0045869	0,831782099	0,405907255
ADV5	chr7.114406314.C.G	independent_variable	-0,003890779	0,00468355	-0,830732797	0,406506584
ADV2	chr1.43887565.G.A	independent_variable	-0,000869991	0,001047981	-0,830158938	0,406840423
ADV1	chr3.20703004.G.A	independent_variable	-0,014823036	0,017856483	-0,830120664	0,406894718
ADV1	chr16.61749670.C.T	independent_variable	0,014666516	0,017700883	0,828575426	0,407715571
ADV3	chr5.104619660.T.C	independent_variable	-0,016181584	0,019542409	-0,828024018	0,408059179
ADV5	chr1.43644078.A.G	independent_variable	0,004176634	0,005045895	0,827729078	0,408190271
ADV5	chr5.104584139.G.A	independent_variable	0,003774663	0,004562742	0,827279415	0,408449622
ADV1	chr3.20695381.T.C	independent_variable	-0,014530435	0,017646432	-0,823420557	0,410682094
ADV1	chr7.114540007.A.G	independent_variable	0,013470738	0,01637468	0,822656577	0,411090821
ADV5	chr5.104582527.C.T	independent_variable	0,003730068	0,004548143	0,820129925	0,412507964
ADV2	chr14.98244157.G.A	independent_variable	0,000775518	0,00094656	0,819300914	0,412983706
ADV2	chr14.98253981.T.A	independent_variable	0,000775518	0,00094656	0,819300914	0,412983706
ADV5	chr5.104581651.A.C	independent_variable	0,003718749	0,004555639	0,816295746	0,414695673
ADV5	chr5.104582166.G.T	independent_variable	0,003718749	0,004555639	0,816295746	0,414695673
ADV5	chr5.104582921.C.T	independent_variable	0,003718749	0,004555639	0,816295746	0,414695673
ADV5	chr5.104582998.G.A	independent_variable	0,003718749	0,004555639	0,816295746	0,414695673
ADV5	chr5.104583048.G.T	independent_variable	0,003718749	0,004555639	0,816295746	0,414695673
ADV5	chr5.104584121.A.C	independent_variable	0,003718749	0,004555639	0,816295746	0,414695673
ADV4	chr5.88569970.A.G	independent_variable	0,000458534	0,000562505	0,815162989	0,415369475
ADV4	chr1.43548902.A.T	independent_variable	-0,000439621	0,000539678	-0,814598682	0,415738762
ADV3	chr5.104614550.C.T	independent_variable	-0,015901911	0,019526	-0,814396773	0,415811687
ADV4	chr6.70144291.A.G	independent_variable	-0,000438051	0,000540202	-0,810902336	0,417789206
ADV4	chr14.98255846.A.C	independent_variable	-0,000437038	0,000539416	-0,810206109	0,418186042
ADV4	chr14.98244157.G.A	independent_variable	-0,000437038	0,000539416	-0,810206109	0,418186042
ADV4	chr14.98253981.T.A	independent_variable	-0,000437038	0,000539416	-0,810206109	0,418186042
ADV4	chr7.114436339.C.G	independent_variable	-0,000397205	0,000490495	-0,809805326	0,41842248
ADV4	chr1.43605020.A.G	independent_variable	-0,000414538	0,000512966	-0,808119798	0,419378796
ADV3	chr20.21372771.A.C	independent_variable	0,01368586	0,016936486	0,808069611	0,419418603
ADV5	chr7.114470513.C.T	independent_variable	0,003971124	0,004921942	0,806820607	0,420122436
ADV3	chr20.21379890.A.G	independent_variable	0,013579734	0,016834354	0,806667995	0,420222362
ADV3	chr20.21388294.G.T	independent_variable	0,013579734	0,016834354	0,806667995	0,420222362
ADV1	chr16.61755912.G.C	independent_variable	0,014242815	0,017691501	0,805065346	0,421140274
ADV4	chr6.70142601.A.G	independent_variable	-0,000437593	0,000543934	-0,804496582	0,421477186
ADV4	chr6.70142949.A.T	independent_variable	-0,000437593	0,000543934	-0,804496582	0,421477186
ADV4	chr6.70143876.T.C	independent_variable	-0,000437593	0,000543934	-0,804496582	0,421477186
ADV1	chr3.20706828.T.G	independent_variable	-0,014304823	0,017805164	-0,803408673	0,422146416
ADV3	chr20.21371598.A.G	independent_variable	0,01337204	0,016656383	0,802817751	0,422430681
ADV2	chr7.114508188.A.G	independent_variable	0,000829191	0,001033656	0,802192424	0,422816761
ADV4	chr6.70148037.A.G	independent_variable	-0,000433555	0,000540898	-0,801546282	0,423178782
ADV4	chr6.70148809.A.C	independent_variable	-0,000433555	0,000540898	-0,801546282	0,423178782
ADV2	chr7.114540007.A.G	independent_variable	0,000825289	0,001036391	0,796310668	0,426224924
ADV5	chr1.43739423.T.C	independent_variable	0,004111874	0,005168659	0,795539932	0,426652305
ADV4	chr20.21372771.A.C	independent_variable	0,000398388	0,000501079	0,795059275	0,426940369
ADV1	chr16.61758377.A.G	independent_variable	0,013975839	0,017615279	0,793393004	0,427899301
ADV5	chr5.104619660.T.C	independent_variable	-0,004095772	0,005163303	-0,793246547	0,428017343

ADV5	chr1.43621160.C.T	independent_variable	0,003975392	0,00501247	0,793100367	0,428065749
ADV2	chr7.114520816.G.A	independent_variable	0,000818964	0,001033032	0,792777141	0,42827776
ADV2	chr1.43686240.C.T	independent_variable	0,000993578	0,001256728	0,790606998	0,429528991
ADV5	chr11.28626638.C.T	independent_variable	0,003869047	0,004893822	0,790598128	0,429534164
ADV5	chr11.28626783.A.T	independent_variable	0,003869047	0,004893822	0,790598128	0,429534164
ADV5	chr11.28626831.G.C	independent_variable	0,003869047	0,004893822	0,790598128	0,429534164
ADV3	chr7.114540007.A.G	independent_variable	-0,013705303	0,017398351	-0,787735782	0,431220114
ADV4	chr20.21277855.T.C	independent_variable	0,000402806	0,000511786	0,787059735	0,431611063
ADV4	chr20.21277966.T.C	independent_variable	0,000402806	0,000511786	0,787059735	0,431611063
ADV3	chr7.114499629.G.A	independent_variable	-0,013962603	0,017764585	-0,785979671	0,432224143
ADV4	chr1.43674404.A.C	independent_variable	-0,000417488	0,00053173	-0,78515102	0,432710639
ADV1	chr4.111546456.G.A	independent_variable	0,015872218	0,020270633	0,78301539	0,434014117
ADV4	chr6.70148891.T.C	independent_variable	-0,000424489	0,000542299	-0,782757957	0,434124215
ADV4	chr1.43596124.C.T	independent_variable	0,000415605	0,000531637	0,781745581	0,43470864
ADV1	chr5.88433894.A.C	independent_variable	0,013785841	0,017634342	0,781761022	0,434723583
ADV4	chr5.88579561.C.T	independent_variable	0,000437895	0,000562206	0,778886769	0,436417853
ADV4	chr5.88584754.A.G	independent_variable	0,000437895	0,000562206	0,778886769	0,436417853
ADV4	chr5.88586361.A.G	independent_variable	0,000437895	0,000562206	0,778886769	0,436417853
ADV3	chr5.104568525.A.G	independent_variable	-0,013646275	0,017572472	-0,776571151	0,437782741
ADV2	chr1.43554723.T.C	independent_variable	0,000859202	0,001108389	0,775180866	0,438574641
ADV2	chr1.43556863.C.T	independent_variable	0,000859202	0,001108389	0,775180866	0,438574641
ADV2	chr11.28608504.A.G	independent_variable	-0,000780649	0,001007466	-0,774864202	0,438772104
ADV4	chr14.98259646.C.T	independent_variable	-0,000418149	0,000539964	-0,77440159	0,43903866
ADV3	chr7.114552490.T.C	independent_variable	-0,014293913	0,018478409	-0,773546729	0,439560114
ADV4	chr1.43554723.T.C	independent_variable	0,000413865	0,000535354	0,773068944	0,439822357
ADV4	chr1.43556863.C.T	independent_variable	0,000413865	0,000535354	0,773068944	0,439822357
ADV5	chr5.104614550.C.T	independent_variable	-0,003986592	0,005158761	-0,772780847	0,440023172
ADV4	chr5.88558577.A.C	independent_variable	-0,00043875	0,000570232	-0,769423849	0,442024977
ADV4	chr1.43510486.G.A	independent_variable	0,000482798	0,000627993	0,768796207	0,442417594
ADV5	chr5.88885292.T.G	independent_variable	-0,004145028	0,00539506	-0,768300635	0,44267497
ADV2	chr1.43903772.G.A	independent_variable	-0,000798194	0,001039748	-0,76768075	0,44303007
ADV4	chr5.88872578.C.G	independent_variable	0,000393791	0,000513028	0,767580943	0,443123271
ADV4	chr5.88873138.C.A	independent_variable	0,000393791	0,000513028	0,767580943	0,443123271
ADV2	chr4.111296654.C.T	independent_variable	-0,001257048	0,001638033	-0,767413111	0,443175746
ADV1	chr16.61753223.C.T	independent_variable	0,01349256	0,017620343	0,765737613	0,4441695
ADV5	chr5.104591878.C.T	independent_variable	-0,003900411	0,005096811	-0,765264907	0,444476326
ADV2	chr7.114522685.G.A	independent_variable	0,000790439	0,001033387	0,764900939	0,444685716
ADV2	chr11.28636483.A.G	independent_variable	-0,000752785	0,000984897	-0,764328117	0,445004355
ADV2	chr11.28627190.A.G	independent_variable	-0,000756044	0,000989186	-0,764309482	0,445017299
ADV3	chr7.114507162.C.A	independent_variable	-0,013926067	0,018281484	-0,761758031	0,446554488
ADV5	chr5.104609477.C.T	independent_variable	-0,003900867	0,005134483	-0,759739086	0,447772961
ADV2	chr11.28609199.A.G	independent_variable	-0,00076611	0,001009038	-0,759247747	0,448049062
ADV4	chr20.21277415.A.G	independent_variable	0,000388163	0,000511648	0,758653428	0,448408992
ADV2	chr14.98224586.T.A	independent_variable	0,000734107	0,000970233	0,75662977	0,449611747
ADV3	chr7.114475213.T.C	independent_variable	-0,013449829	0,017791037	-0,755989021	0,44998543
ADV2	chr1.43555785.C.G	independent_variable	0,000841945	0,001117841	0,753188787	0,451668404
ADV4	chr1.43550344.G.A	independent_variable	-0,000425578	0,000566633	-0,751064054	0,452965334
ADV5	chr5.104611504.C.T	independent_variable	-0,003860686	0,005141427	-0,750897648	0,453072513
ADV5	chr5.104612344.C.T	independent_variable	-0,003860686	0,005141427	-0,750897648	0,453072513
ADV5	chr1.44009451.C.T	independent_variable	-0,00468481	0,006249277	-0,749656411	0,453829684
ADV5	chr3.20685150.G.A	independent_variable	-0,003712649	0,004958286	-0,748776594	0,454371263
ADV5	chr3.20685154.C.T	independent_variable	-0,003712649	0,004958286	-0,748776594	0,454371263
ADV5	chr5.104590741.T.A	independent_variable	-0,003777942	0,005079911	-0,743702346	0,457406596
ADV5	chr5.104600642.T.A	independent_variable	-0,003777942	0,005079911	-0,743702346	0,457406596
ADV4	chr20.21281046.C.T	independent_variable	0,000379562	0,000510632	0,743317082	0,457630005
ADV2	chr5.88385177.A.G	independent_variable	0,000840376	0,00113099	0,743044645	0,457791498
ADV2	chr5.88389683.C.T	independent_variable	0,000840376	0,00113099	0,743044645	0,457791498
ADV4	chr7.114443495.C.G	independent_variable	-0,000363108	0,00048903	-0,742506585	0,458113811
ADV4	chr7.114505470.G.A	independent_variable	-0,000374104	0,000504479	-0,741565614	0,458693922
ADV2	chr11.28609522.G.C	independent_variable	-0,000743691	0,001004785	-0,740148914	0,459543037
ADV2	chr11.28609701.C.A	independent_variable	-0,000743691	0,001004785	-0,740148914	0,459543037
ADV2	chr11.28605957.T.C	independent_variable	-0,0007297	0,000986106	-0,739981775	0,459631451
ADV4	chr7.114531090.A.G	independent_variable	0,000388154	0,000524795	0,739629788	0,459864302
ADV5	chr4.111267775.T.C	independent_variable	0,006159425	0,008334594	0,739019196	0,460226241
ADV2	chr11.28609187.T.G	independent_variable	-0,000743776	0,001007	-0,738605956	0,460479535
ADV3	chr3.20685150.G.A	independent_variable	0,013510158	0,018341306	0,736597377	0,461739481
ADV3	chr3.20685154.C.T	independent_variable	0,013510158	0,018341306	0,736597377	0,461739481
ADV3	chr3.20680838.A.C	independent_variable	0,012307557	0,016721643	0,736025583	0,462037705
ADV2	chr11.28605239.G.A	independent_variable	-0,000738176	0,001003103	-0,735892916	0,462126406
ADV2	chr11.28606106.G.A	independent_variable	-0,000738176	0,001003103	-0,735892916	0,462126406
ADV2	chr11.28607002.C.A	independent_variable	-0,000738176	0,001003103	-0,735892916	0,462126406

ADV2	chr11.28607568.G.A	independent_variable	-0,000738176	0,001003103	-0,735892916	0,462126406
ADV2	chr11.28613204.C.T	independent_variable	-0,000738176	0,001003103	-0,735892916	0,462126406
ADV2	chr11.28613315.A.G	independent_variable	-0,000738176	0,001003103	-0,735892916	0,462126406
ADV2	chr11.28613316.T.C	independent_variable	-0,000738176	0,001003103	-0,735892916	0,462126406
ADV2	chr11.28614382.G.A	independent_variable	-0,000738176	0,001003103	-0,735892916	0,462126406
ADV2	chr11.28615789.A.G	independent_variable	-0,000738176	0,001003103	-0,735892916	0,462126406
ADV2	chr5.88397820.G.C	independent_variable	0,000828014	0,001128474	0,733746653	0,463435121
ADV5	chr20.21267478.G.A	independent_variable	-0,003580623	0,004889245	-0,732346807	0,464290719
ADV4	chr7.114484605.C.G	independent_variable	-0,000356904	0,000487961	-0,731417792	0,464850331
ADV2	chr7.114560479.G.A	independent_variable	-0,00073498	0,001006297	-0,730380598	0,465482748
ADV4	chr20.21283529.T.C	independent_variable	0,000373551	0,000511517	0,730279638	0,465553279
ADV4	chr20.21291449.A.T	independent_variable	0,000373551	0,000511517	0,730279638	0,465553279
ADV4	chr20.21390733.A.G	independent_variable	0,000364606	0,000499508	0,729930985	0,465757931
ADV5	chr5.104596006.G.A	independent_variable	-0,003715256	0,005118456	-0,72585498	0,468268654
ADV2	chr11.28609937.C.T	independent_variable	-0,000729039	0,001006358	-0,724432999	0,469125758
ADV2	chr5.88386213.G.A	independent_variable	0,000815351	0,001127176	0,72335738	0,469786224
ADV4	chr1.43682946.A.G	independent_variable	-0,000443456	0,000614099	-0,722125621	0,470538151
ADV4	chr5.104605369.T.G	independent_variable	0,000362995	0,000502785	0,721968344	0,470633547
ADV4	chr5.104605700.T.C	independent_variable	0,000362995	0,000502785	0,721968344	0,470633547
ADV4	chr5.104605714.T.G	independent_variable	0,000362995	0,000502785	0,721968344	0,470633547
ADV4	chr20.21318093.T.C	independent_variable	0,000363641	0,000504022	0,721478897	0,470939186
ADV4	chr20.21318094.G.T	independent_variable	0,000363641	0,000504022	0,721478897	0,470939186
ADV4	chr20.21319392.G.A	independent_variable	0,000363641	0,000504022	0,721478897	0,470939186
ADV4	chr20.21320839.C.T	independent_variable	0,000363641	0,000504022	0,721478897	0,470939186
ADV4	chr20.21321927.C.T	independent_variable	0,000363641	0,000504022	0,721478897	0,470939186
ADV4	chr20.21321943.T.C	independent_variable	0,000363641	0,000504022	0,721478897	0,470939186
ADV4	chr20.21327058.G.A	independent_variable	0,000363641	0,000504022	0,721478897	0,470939186
ADV4	chr20.21329677.C.T	independent_variable	0,000363641	0,000504022	0,721478897	0,470939186
ADV4	chr7.114503352.C.T	independent_variable	-0,000351742	0,000488736	-0,719696508	0,472032743
ADV5	chr4.111296654.C.T	independent_variable	0,005431804	0,007559242	0,718564646	0,472724076
ADV5	chr5.104608319.G.T	independent_variable	-0,003708156	0,005163071	-0,718207491	0,472969573
ADV4	chr20.21372841.G.C	independent_variable	0,000358848	0,000500356	0,71718496	0,473579541
ADV3	chr7.114503596.T.A	independent_variable	-0,012712991	0,017740989	-0,716588645	0,47393775
ADV4	chr20.21345460.T.C	independent_variable	0,00035649	0,000498542	0,715063848	0,474886843
ADV4	chr20.21346867.T.G	independent_variable	0,00035649	0,000498542	0,715063848	0,474886843
ADV4	chr20.21349142.T.C	independent_variable	0,00035649	0,000498542	0,715063848	0,474886843
ADV4	chr20.21350734.G.A	independent_variable	0,00035649	0,000498542	0,715063848	0,474886843
ADV4	chr20.21351811.A.G	independent_variable	0,00035649	0,000498542	0,715063848	0,474886843
ADV4	chr20.21352024.G.C	independent_variable	0,00035649	0,000498542	0,715063848	0,474886843
ADV4	chr20.21353013.G.C	independent_variable	0,00035649	0,000498542	0,715063848	0,474886843
ADV4	chr20.21353030.G.A	independent_variable	0,00035649	0,000498542	0,715063848	0,474886843
ADV4	chr20.21359219.A.G	independent_variable	0,00035649	0,000498542	0,715063848	0,474886843
ADV4	chr20.21361740.T.C	independent_variable	0,00035649	0,000498542	0,715063848	0,474886843
ADV4	chr20.21379890.A.G	independent_variable	0,00035649	0,000498542	0,715063848	0,474886843
ADV4	chr20.21388294.G.T	independent_variable	0,00035649	0,000498542	0,715063848	0,474886843
ADV4	chr20.21283152.A.T	independent_variable	0,000365021	0,000510506	0,715017971	0,474923203
ADV4	chr20.21284820.G.A	independent_variable	0,000365021	0,000510506	0,715017971	0,474923203
ADV4	chr20.21285090.C.T	independent_variable	0,000365021	0,000510506	0,715017971	0,474923203
ADV4	chr20.21285802.C.T	independent_variable	0,000365021	0,000510506	0,715017971	0,474923203
ADV4	chr20.21286713.C.T	independent_variable	0,000365021	0,000510506	0,715017971	0,474923203
ADV4	chr20.21287631.G.A	independent_variable	0,000365021	0,000510506	0,715017971	0,474923203
ADV4	chr20.21290685.C.G	independent_variable	0,000365021	0,000510506	0,715017971	0,474923203
ADV4	chr20.21290915.A.G	independent_variable	0,000365021	0,000510506	0,715017971	0,474923203
ADV4	chr20.21290947.G.A	independent_variable	0,000365021	0,000510506	0,715017971	0,474923203
ADV4	chr20.21291136.G.T	independent_variable	0,000365021	0,000510506	0,715017971	0,474923203
ADV4	chr20.21292673.G.A	independent_variable	0,000365021	0,000510506	0,715017971	0,474923203
ADV3	chr7.114474994.A.G	independent_variable	-0,01262934	0,017686707	-0,714058323	0,475497935
ADV5	chr5.104605369.T.G	independent_variable	0,003278977	0,004593999	0,71375237	0,475695692
ADV5	chr5.104605700.T.C	independent_variable	0,003278977	0,004593999	0,71375237	0,475695692
ADV5	chr5.104605714.T.G	independent_variable	0,003278977	0,004593999	0,71375237	0,475695692
ADV4	chr20.21270205.T.C	independent_variable	0,000362573	0,000508619	0,712858344	0,47625628
ADV5	chr5.104585069.C.A	independent_variable	-0,003650274	0,005127997	-0,71183226	0,476903315
ADV5	chr7.114476826.G.A	independent_variable	-0,003210853	0,004513347	-0,711412861	0,477144397
ADV5	chr11.28621014.G.C	independent_variable	0,002398738	0,003372636	0,711235409	0,477259751
ADV5	chr5.104597772.C.T	independent_variable	-0,003623028	0,005100967	-0,71026299	0,477872862
ADV4	chr4.111267775.T.C	independent_variable	0,000650583	0,000918008	0,708689932	0,47883207
ADV4	chr20.21308964.A.G	independent_variable	0,000357758	0,000504886	0,708591836	0,478895944
ADV4	chr20.21313246.C.T	independent_variable	0,000357758	0,000504886	0,708591836	0,478895944
ADV4	chr20.21315921.C.A	independent_variable	0,000357758	0,000504886	0,708591836	0,478895944
ADV5	chr5.104610505.A.T	independent_variable	-0,003640485	0,005145896	-0,707454012	0,47961799
ADV5	chr1.43756926.G.A	independent_variable	0,003707765	0,005242167	0,707296218	0,479693878

ADV5	chr3.20680326.A.G	independent_variable	-0,003128343	0,0044305	-0,706092487	0,480437983
ADV5	chr3.20680427.C.T	independent_variable	-0,003128343	0,0044305	-0,706092487	0,480437983
ADV5	chr3.20683524.C.A	independent_variable	-0,003128343	0,0044305	-0,706092487	0,480437983
ADV2	chr11.28615816.T.C	independent_variable	-0,000708835	0,001006238	-0,704440867	0,481473623
ADV4	chr7.114489082.G.A	independent_variable	-0,000343288	0,000487707	-0,703881133	0,48181887
ADV4	chr7.114498696.G.C	independent_variable	-0,000343288	0,000487707	-0,703881133	0,48181887
ADV4	chr7.114502195.A.G	independent_variable	-0,000343288	0,000487707	-0,703881133	0,48181887
ADV4	chr7.114476826.G.A	independent_variable	-0,000343288	0,000487707	-0,703881133	0,48181887
ADV4	chr1.43616545.G.A	independent_variable	0,000376101	0,000535051	0,702926656	0,482404942
ADV5	chr3.20682712.G.A	independent_variable	-0,003113396	0,004430557	-0,702709728	0,482542306
ADV4	chr20.21371667.C.T	independent_variable	0,000350731	0,000499389	0,702320742	0,482790421
ADV4	chr20.21373277.C.T	independent_variable	0,000350731	0,000499389	0,702320742	0,482790421
ADV4	chr20.21289658.C.T	independent_variable	0,000358966	0,000511393	0,701936881	0,483037484
ADV4	chr20.21291893.C.T	independent_variable	0,000358966	0,000511393	0,701936881	0,483037484
ADV4	chr1.43705540.C.T	independent_variable	-0,000431789	0,000615285	-0,701770707	0,483131379
ADV4	chr5.88871923.G.C	independent_variable	0,000360038	0,00051304	0,701774484	0,483167908
ADV5	chr7.114479375.G.A	independent_variable	-0,003167078	0,004520596	-0,700588585	0,483870761
ADV2	chr11.28604117.C.T	independent_variable	-0,000701497	0,001002655	-0,69963931	0,48446424
ADV2	chr1.43552933.G.T	independent_variable	0,00076719	0,001096657	0,699571914	0,484497042
ADV2	chr5.88861735.T.C	independent_variable	0,000685136	0,000979699	0,699333149	0,48464497
ADV4	chr14.98239063.A.G	independent_variable	-0,000378869	0,000543316	-0,697326403	0,485908406
ADV5	chr11.28655215.G.C	independent_variable	0,00332745	0,004776913	0,696568969	0,486374543
ADV5	chr5.104608237.C.G	independent_variable	-0,003582902	0,005145581	-0,696306464	0,486564407
ADV4	chr20.21310893.C.T	independent_variable	0,000351833	0,000505755	0,695659035	0,48695436
ADV4	chr20.21311200.C.T	independent_variable	0,000351833	0,000505755	0,695659035	0,48695436
ADV4	chr20.21311590.G.C	independent_variable	0,000351833	0,000505755	0,695659035	0,48695436
ADV4	chr20.21312173.C.T	independent_variable	0,000351833	0,000505755	0,695659035	0,48695436
ADV4	chr20.21312391.G.A	independent_variable	0,000351833	0,000505755	0,695659035	0,48695436
ADV4	chr20.21314871.G.T	independent_variable	0,000351833	0,000505755	0,695659035	0,48695436
ADV4	chr20.21328174.G.A	independent_variable	0,000349508	0,000503903	0,693600406	0,48824254
ADV4	chr20.21299182.T.G	independent_variable	0,000349508	0,000503903	0,693600406	0,48824254
ADV3	chr5.104482711.A.G	independent_variable	-0,011573348	0,016689145	-0,693465586	0,488314482
ADV4	chr7.114479375.G.A	independent_variable	-0,000338069	0,000488484	-0,692078381	0,489194657
ADV2	chr11.28603810.A.T	independent_variable	-0,000696034	0,001005884	-0,691962512	0,489269718
ADV2	chr1.43576868.C.G	independent_variable	-0,000756647	0,001094483	-0,691328263	0,489659704
ADV1	chr7.114508276.C.A	independent_variable	0,011203586	0,016234229	0,690121213	0,490433121
ADV3	chr7.114508276.C.A	independent_variable	-0,011874482	0,017261553	-0,687915029	0,491820271
ADV4	chr1.43540668.A.G	independent_variable	0,000400444	0,000583371	0,686431565	0,492775943
ADV5	chr7.114497885.A.G	independent_variable	-0,003095081	0,004511046	-0,68611153	0,492945665
ADV4	chr1.43686240.C.T	independent_variable	-0,000421745	0,000615674	-0,685013518	0,493636562
ADV4	chr20.21268900.C.T	independent_variable	0,000348173	0,0005085	0,684706203	0,493837359
ADV1	chr12.89364160.G.A	independent_variable	0,012895588	0,018843185	0,684363512	0,494070521
ADV1	chr12.89366967.A.G	independent_variable	0,012895588	0,018843185	0,684363512	0,494070521
ADV5	chr20.21277855.T.C	independent_variable	0,003159173	0,004618424	0,684037037	0,494261253
ADV5	chr20.21277966.T.C	independent_variable	0,003159173	0,004618424	0,684037037	0,494261253
ADV5	chr3.20683444.C.T	independent_variable	-0,003024648	0,004423039	-0,683839287	0,494372178
ADV5	chr5.104581838.T.A	independent_variable	0,003144735	0,004603354	0,683139801	0,494813677
ADV5	chr5.104582136.T.C	independent_variable	0,003144735	0,004603354	0,683139801	0,494813677
ADV4	chr1.43853701.G.A	independent_variable	0,00036685	0,000537324	0,682734955	0,495064966
ADV4	chr20.21294655.A.C	independent_variable	0,000343582	0,000504771	0,680670507	0,496383548
ADV5	chr20.21293229.C.T	independent_variable	0,003104208	0,00456293	0,680310307	0,496611943
ADV5	chr20.21294030.G.A	independent_variable	0,003104208	0,00456293	0,680310307	0,496611943
ADV2	chr14.98259646.C.T	independent_variable	0,000640802	0,000942879	0,679622274	0,497040375
ADV4	chr20.21169993.T.C	independent_variable	-0,000378892	0,000558343	-0,678600866	0,497690948
ADV4	chr20.21173142.G.C	independent_variable	-0,000378892	0,000558343	-0,678600866	0,497690948
ADV2	chr7.114544043.G.A	independent_variable	0,000705932	0,001040279	0,678598625	0,497704297
ADV4	chr7.114469294.A.G	independent_variable	-0,000329712	0,00048746	-0,676388423	0,499091936
ADV4	chr7.114497885.A.G	independent_variable	-0,000329712	0,00048746	-0,676388423	0,499091936
ADV5	chr4.111295017.T.A	independent_variable	0,005063367	0,007491201	0,675908514	0,499391341
ADV5	chr4.111296367.C.T	independent_variable	0,005063367	0,007491201	0,675908514	0,499391341
ADV5	chr4.111306830.A.T	independent_variable	0,005063367	0,007491201	0,675908514	0,499391341
ADV5	chr5.88416702.C.A	independent_variable	-0,003489174	0,005163863	-0,67569079	0,499536779
ADV4	chr20.21291031.G.C	independent_variable	0,000346723	0,000513183	0,675632829	0,49958002
ADV2	chr1.43574039.T.C	independent_variable	0,00068683	0,001017019	0,67533687	0,499757965
ADV4	chr5.88417014.A.G	independent_variable	-0,000369549	0,000547374	-0,675129616	0,499895238
ADV4	chr20.21374422.T.A	independent_variable	0,000336881	0,000499277	0,674738558	0,500138904
ADV4	chr20.21375208.A.G	independent_variable	0,000336881	0,000499277	0,674738558	0,500138904
ADV5	chr1.43872903.A.C	independent_variable	0,003523519	0,005227023	0,674096616	0,500569722
ADV5	chr20.21299182.T.G	independent_variable	0,003057208	0,004547995	0,672209994	0,50174858
ADV5	chr4.111287592.T.C	independent_variable	0,005032466	0,007498468	0,67113259	0,502426802
ADV4	chr1.43547252.C.T	independent_variable	0,000385682	0,000576426	0,66909206	0,503761815

ADV5	chr5.88401026.G.A	independent_variable	-0,003457245	0,005170386	-0,668662931	0,504007236
ADV4	chr20.21293229.C.T	independent_variable	0,000337615	0,000505642	0,667694446	0,504626014
ADV4	chr20.21294030.G.A	independent_variable	0,000337615	0,000505642	0,667694446	0,504626014
ADV5	chr11.28648922.G.A	independent_variable	0,003192754	0,00478293	0,667530932	0,504720352
ADV5	chr11.28654958.T.C	independent_variable	0,003192754	0,00478293	0,667530932	0,504720352
ADV5	chr11.28655314.A.G	independent_variable	0,003192754	0,00478293	0,667530932	0,504720352
ADV5	chr11.28657323.A.G	independent_variable	0,003192754	0,00478293	0,667530932	0,504720352
ADV4	chr5.104606092.C.A	independent_variable	0,000336079	0,000503501	0,667484111	0,504754576
ADV4	chr20.21299701.C.T	independent_variable	0,000335399	0,000503792	0,665750104	0,505866048
ADV2	chr1.43575295.T.G	independent_variable	-0,000722594	0,001087489	-0,664461261	0,50668249
ADV4	chr7.114507162.C.A	independent_variable	0,000348175	0,000524959	0,66324249	0,507473175
ADV4	chr1.43541977.T.C	independent_variable	-0,000360018	0,000543124	-0,6628653	0,507763915
ADV2	chr11.28621752.T.C	independent_variable	-0,000663943	0,001002317	-0,662408376	0,508005283
ADV5	chr5.88417014.A.G	independent_variable	-0,003435013	0,005201679	-0,660366355	0,509312866
ADV5	chr11.28650082.G.C	independent_variable	0,003162474	0,004789616	0,66027725	0,509360098
ADV5	chr20.21294655.A.C	independent_variable	0,003007548	0,004555872	0,660147523	0,509452026
ADV4	chr20.21187755.C.T	independent_variable	0,000350822	0,000532292	0,659079334	0,510137132
ADV5	chr3.20678505.C.T	independent_variable	-0,00291742	0,004429835	-0,658584349	0,510446034
ADV5	chr11.28634517.A.T	independent_variable	0,003151311	0,004789459	0,657967972	0,51084172
ADV5	chr11.28634687.C.A	independent_variable	0,003151311	0,004789459	0,657967972	0,51084172
ADV5	chr11.28644991.C.T	independent_variable	0,003151311	0,004789459	0,657967972	0,51084172
ADV5	chr11.28646737.A.G	independent_variable	0,003151311	0,004789459	0,657967972	0,51084172
ADV5	chr20.21277415.A.G	independent_variable	0,003037364	0,00461714	0,657845462	0,51093309
ADV5	chr7.114416000.G.C	independent_variable	-0,003069475	0,004679648	-0,655920092	0,512163434
ADV5	chr5.104578822.T.C	independent_variable	0,002973872	0,004537987	0,655328311	0,512533927
ADV4	chr1.43616041.T.G	independent_variable	0,000352253	0,000537778	0,655015625	0,512737646
ADV4	chr1.43620409.T.C	independent_variable	0,000352253	0,000537778	0,655015625	0,512737646
ADV5	chr1.43616091.T.G	independent_variable	-0,003202667	0,004910137	-0,652256228	0,514520817
ADV5	chr3.20680838.A.C	independent_variable	-0,002889705	0,00443068	-0,652203529	0,51454999
ADV2	chr11.28621106.G.A	independent_variable	-0,000637857	0,000980154	-0,650772469	0,515487605
ADV5	chr1.43737320.A.G	independent_variable	0,00334805	0,005145566	0,650666908	0,515539823
ADV1	chr7.114544043.G.A	independent_variable	0,010713277	0,016469492	0,650492265	0,515671301
ADV4	chr5.88874249.T.C	independent_variable	0,000342296	0,000526598	0,650014468	0,516002492
ADV4	chr20.21187721.G.T	independent_variable	0,000345339	0,000531581	0,649645411	0,516208153
ADV4	chr20.21190532.C.T	independent_variable	0,000345339	0,000531581	0,649645411	0,516208153
ADV4	chr20.21194910.T.C	independent_variable	0,000345339	0,000531581	0,649645411	0,516208153
ADV4	chr1.43549064.C.T	independent_variable	-0,00034806	0,000536439	-0,648835262	0,51677881
ADV3	chr20.21268900.C.T	independent_variable	0,011030461	0,01701347	0,648336915	0,517056024
ADV2	chr1.43705540.C.T	independent_variable	0,000811871	0,001252954	0,647965911	0,517288658
ADV3	chr5.88433894.A.C	independent_variable	-0,012064016	0,018632812	-0,647460821	0,517629716
ADV4	chr20.21227106.T.G	independent_variable	0,000331988	0,000512986	0,647166853	0,517797448
ADV5	chr20.21260820.G.A	independent_variable	0,003075028	0,004752606	0,647019477	0,517908171
ADV4	chr1.43616202.A.C	independent_variable	0,000347468	0,000537132	0,646895678	0,517974843
ADV4	chr1.43617439.C.T	independent_variable	0,000347468	0,000537132	0,646895678	0,517974843
ADV4	chr1.43617836.T.C	independent_variable	0,000347468	0,000537132	0,646895678	0,517974843
ADV4	chr1.43617848.G.A	independent_variable	0,000347468	0,000537132	0,646895678	0,517974843
ADV4	chr1.43618203.A.G	independent_variable	0,000347468	0,000537132	0,646895678	0,517974843
ADV4	chr1.43619068.C.T	independent_variable	0,000347468	0,000537132	0,646895678	0,517974843
ADV4	chr1.43624269.A.G	independent_variable	0,000347468	0,000537132	0,646895678	0,517974843
ADV4	chr1.43625004.T.C	independent_variable	0,000347468	0,000537132	0,646895678	0,517974843
ADV4	chr1.43625065.C.T	independent_variable	0,000347468	0,000537132	0,646895678	0,517974843
ADV4	chr1.43625603.A.G	independent_variable	0,000347468	0,000537132	0,646895678	0,517974843
ADV5	chr20.21299701.C.T	independent_variable	0,002938439	0,004546971	0,646240905	0,518408452
ADV5	chr11.28655291.A.G	independent_variable	0,003035212	0,004699968	0,645794094	0,51868552
ADV5	chr20.21281046.C.T	independent_variable	0,002966177	0,004607884	0,643717861	0,520046073
ADV2	chr1.43623961.A.G	independent_variable	-0,000697821	0,001085271	-0,642992405	0,52050297
ADV5	chr5.104612267.T.C	independent_variable	0,003174005	0,004946844	0,641622136	0,521392835
ADV4	chr1.43662376.A.G	independent_variable	0,000343949	0,00053778	0,639572508	0,522721789
ADV5	chr1.43658195.A.G	independent_variable	-0,003132309	0,004897584	-0,639562094	0,522734194
ADV5	chr20.21372771.A.C	independent_variable	0,002886426	0,004532559	0,636820343	0,524521038
ADV2	chr5.88864537.C.T	independent_variable	0,000636309	0,001001047	0,635643278	0,525286452
ADV4	chr5.104590741.T.A	independent_variable	0,000364474	0,000574405	0,634524128	0,52602962
ADV4	chr5.104600642.T.A	independent_variable	0,000364474	0,000574405	0,634524128	0,52602962
ADV3	chr3.20680326.A.G	independent_variable	0,010603259	0,01672429	0,634003521	0,526349497
ADV3	chr3.20680427.C.T	independent_variable	0,010603259	0,01672429	0,634003521	0,526349497
ADV3	chr3.20683524.C.A	independent_variable	0,010603259	0,01672429	0,634003521	0,526349497
ADV4	chr7.114571857.G.A	independent_variable	0,000320528	0,000506286	0,633097548	0,52695206
ADV2	chr1.43620409.T.C	independent_variable	-0,000684804	0,001082903	-0,632378507	0,527408228
ADV5	chr20.21283529.T.C	independent_variable	0,002915797	0,004615921	0,631682566	0,52787625
ADV4	chr7.114589084.G.A	independent_variable	0,000304127	0,000481599	0,631493685	0,527992107
ADV3	chr3.20682712.G.A	independent_variable	0,010531667	0,016709806	0,630268669	0,528787922

ADV5	chr7.114419101.C.T	independent_variable	-0,002946583	0,004677624	-0,629931519	0,529013969
ADV2	chr1.43585185.T.C	independent_variable	-0,000682595	0,001086393	-0,628313027	0,530066028
ADV5	chr20.21350903.T.G	independent_variable	0,002848842	0,004537825	0,627798932	0,530410731
ADV5	chr14.98244157.G.A	independent_variable	-0,003218469	0,005128458	-0,627570528	0,530556112
ADV5	chr14.98253981.T.A	independent_variable	-0,003218469	0,005128458	-0,627570528	0,530556112
ADV5	chr14.98255846.A.C	independent_variable	-0,003218469	0,005128458	-0,627570528	0,530556112
ADV4	chr1.43623961.A.G	independent_variable	0,000337764	0,00053884	0,626834743	0,531033464
ADV5	chr20.21390733.A.G	independent_variable	0,002824707	0,00450862	0,626512556	0,531251119
ADV2	chr4.111287592.T.C	independent_variable	-0,001017141	0,001626664	-0,625292478	0,532046734
ADV5	chr7.114484605.C.G	independent_variable	-0,002823421	0,004516366	-0,625153352	0,532142038
ADV5	chr20.21345460.T.C	independent_variable	0,002810277	0,004499413	0,624587526	0,532512628
ADV5	chr20.21346867.T.G	independent_variable	0,002810277	0,004499413	0,624587526	0,532512628
ADV5	chr20.21349142.T.C	independent_variable	0,002810277	0,004499413	0,624587526	0,532512628
ADV5	chr20.21350734.G.A	independent_variable	0,002810277	0,004499413	0,624587526	0,532512628
ADV5	chr20.21351811.A.G	independent_variable	0,002810277	0,004499413	0,624587526	0,532512628
ADV5	chr20.21352024.G.C	independent_variable	0,002810277	0,004499413	0,624587526	0,532512628
ADV5	chr20.21353013.G.C	independent_variable	0,002810277	0,004499413	0,624587526	0,532512628
ADV5	chr20.21353030.G.A	independent_variable	0,002810277	0,004499413	0,624587526	0,532512628
ADV5	chr20.21359219.A.G	independent_variable	0,002810277	0,004499413	0,624587526	0,532512628
ADV5	chr20.21361740.T.C	independent_variable	0,002810277	0,004499413	0,624587526	0,532512628
ADV5	chr20.21379890.A.G	independent_variable	0,002810277	0,004499413	0,624587526	0,532512628
ADV5	chr20.21388294.G.T	independent_variable	0,002810277	0,004499413	0,624587526	0,532512628
ADV5	chr20.21318093.T.C	independent_variable	0,002839851	0,004549509	0,624210575	0,532763053
ADV5	chr20.21318094.G.T	independent_variable	0,002839851	0,004549509	0,624210575	0,532763053
ADV5	chr20.21319392.G.A	independent_variable	0,002839851	0,004549509	0,624210575	0,532763053
ADV5	chr20.21320839.C.T	independent_variable	0,002839851	0,004549509	0,624210575	0,532763053
ADV5	chr20.21321927.C.T	independent_variable	0,002839851	0,004549509	0,624210575	0,532763053
ADV5	chr20.21321943.T.C	independent_variable	0,002839851	0,004549509	0,624210575	0,532763053
ADV5	chr20.21327058.G.A	independent_variable	0,002839851	0,004549509	0,624210575	0,532763053
ADV5	chr20.21329677.C.T	independent_variable	0,002839851	0,004549509	0,624210575	0,532763053
ADV4	chr1.43636043.A.T	independent_variable	0,000334471	0,000536501	0,623430299	0,53326405
ADV4	chr1.43636129.A.G	independent_variable	0,000334471	0,000536501	0,623430299	0,53326405
ADV4	chr1.43636586.T.C	independent_variable	0,000334471	0,000536501	0,623430299	0,53326405
ADV2	chr14.98255846.A.C	independent_variable	0,000586779	0,000941947	0,622942584	0,533590708
ADV4	chr1.43641757.C.T	independent_variable	0,000336128	0,000539927	0,622542911	0,533847727
ADV4	chr7.114578527.C.T	independent_variable	0,000313869	0,000504157	0,622562006	0,533847938
ADV4	chr7.114583668.C.A	independent_variable	0,000313869	0,000504157	0,622562006	0,533847938
ADV4	chr7.114584108.A.G	independent_variable	0,000313869	0,000504157	0,622562006	0,533847938
ADV4	chr4.111296654.C.T	independent_variable	0,000499351	0,000802137	0,622525655	0,533862512
ADV5	chr1.43885226.G.T	independent_variable	0,003220079	0,005176365	0,622073534	0,53417764
ADV4	chr1.43609834.G.C	independent_variable	0,000330987	0,000532801	0,621221329	0,534717119
ADV5	chr5.104602414.C.T	independent_variable	0,002839068	0,004572389	0,620915754	0,534921983
ADV2	chr11.28578333.T.A	independent_variable	-0,000648872	0,001045644	-0,620547758	0,535174421
ADV4	chr1.43563682.C.G	independent_variable	-0,000329289	0,000531159	-0,619944541	0,535562255
ADV2	chr4.111295017.T.A	independent_variable	-0,001007196	0,001625233	-0,619724375	0,535703599
ADV2	chr4.111296367.C.T	independent_variable	-0,001007196	0,001625233	-0,619724375	0,535703599
ADV2	chr4.111306830.A.T	independent_variable	-0,001007196	0,001625233	-0,619724375	0,535703599
ADV4	chr1.43629437.A.G	independent_variable	0,000333004	0,000538194	0,618742658	0,5363473
ADV4	chr1.43631843.C.G	independent_variable	0,000333004	0,000538194	0,618742658	0,5363473
ADV4	chr1.43634192.G.T	independent_variable	0,000333004	0,000538194	0,618742658	0,5363473
ADV4	chr1.43634239.T.C	independent_variable	0,000333004	0,000538194	0,618742658	0,5363473
ADV5	chr5.104590343.C.T	independent_variable	-0,003195568	0,00516791	-0,618348168	0,536627989
ADV5	chr20.21283152.A.T	independent_variable	0,002845205	0,004606705	0,617622731	0,537098116
ADV5	chr20.21284820.G.A	independent_variable	0,002845205	0,004606705	0,617622731	0,537098116
ADV5	chr20.21285090.C.T	independent_variable	0,002845205	0,004606705	0,617622731	0,537098116
ADV5	chr20.21285802.C.T	independent_variable	0,002845205	0,004606705	0,617622731	0,537098116
ADV5	chr20.21286713.C.T	independent_variable	0,002845205	0,004606705	0,617622731	0,537098116
ADV5	chr20.21287631.G.A	independent_variable	0,002845205	0,004606705	0,617622731	0,537098116
ADV5	chr20.21290685.C.G	independent_variable	0,002845205	0,004606705	0,617622731	0,537098116
ADV5	chr20.21290915.A.G	independent_variable	0,002845205	0,004606705	0,617622731	0,537098116
ADV5	chr20.21290947.G.A	independent_variable	0,002845205	0,004606705	0,617622731	0,537098116
ADV5	chr20.21291136.G.T	independent_variable	0,002845205	0,004606705	0,617622731	0,537098116
ADV5	chr20.21292673.G.A	independent_variable	0,002845205	0,004606705	0,617622731	0,537098116
ADV3	chr7.114544043.G.A	independent_variable	-0,010762814	0,017499192	-0,615046316	0,538802275
ADV5	chr7.114505470.G.A	independent_variable	-0,002873438	0,004673036	-0,614897362	0,538898374
ADV5	chr7.114503352.C.T	independent_variable	-0,002779222	0,004523588	-0,614384456	0,539227967
ADV2	chr1.43616202.A.C	independent_variable	-0,000664632	0,001081964	-0,614282433	0,539287902
ADV2	chr1.43617439.C.T	independent_variable	-0,000664632	0,001081964	-0,614282433	0,539287902
ADV2	chr1.43617836.T.C	independent_variable	-0,000664632	0,001081964	-0,614282433	0,539287902
ADV2	chr1.43617848.G.A	independent_variable	-0,000664632	0,001081964	-0,614282433	0,539287902
ADV2	chr1.43618203.A.G	independent_variable	-0,000664632	0,001081964	-0,614282433	0,539287902

ADV2	chr1.43619068.C.T	independent_variable	-0,000664632	0,001081964	-0,614282433	0,539287902
ADV2	chr1.43624269.A.G	independent_variable	-0,000664632	0,001081964	-0,614282433	0,539287902
ADV2	chr1.43625004.T.C	independent_variable	-0,000664632	0,001081964	-0,614282433	0,539287902
ADV2	chr1.43625065.C.T	independent_variable	-0,000664632	0,001081964	-0,614282433	0,539287902
ADV2	chr1.43625603.A.G	independent_variable	-0,000664632	0,001081964	-0,614282433	0,539287902
ADV2	chr1.43540668.A.G	independent_variable	0,000750264	0,001223316	0,613303663	0,539969126
ADV4	chr5.104591878.C.T	independent_variable	0,000353396	0,000576341	0,613172154	0,540042446
ADV5	chr1.43896951.G.A	independent_variable	0,003169548	0,005169245	0,613154881	0,540052738
ADV5	chr20.21371667.C.T	independent_variable	0,002761467	0,004507093	0,612693462	0,540344343
ADV5	chr20.21373277.C.T	independent_variable	0,002761467	0,004507093	0,612693462	0,540344343
ADV5	chr20.21308964.A.G	independent_variable	0,002789987	0,004557355	0,612194501	0,540677186
ADV5	chr20.21313246.C.T	independent_variable	0,002789987	0,004557355	0,612194501	0,540677186
ADV5	chr20.21315921.C.A	independent_variable	0,002789987	0,004557355	0,612194501	0,540677186
ADV4	chr1.43853335.A.G	independent_variable	0,000332807	0,00054374	0,612070812	0,540749009
ADV4	chr5.104609477.C.T	independent_variable	0,000354526	0,000580642	0,610576701	0,541760651
ADV5	chr20.21372841.G.C	independent_variable	0,002746089	0,004516313	0,608037753	0,543425876
ADV5	chr16.61781742.A.G	independent_variable	-0,003142397	0,005176257	-0,607079084	0,544056463
ADV5	chr20.21291449.A.T	independent_variable	0,002801885	0,004616213	0,606966086	0,544142476
ADV5	chr20.21289658.C.T	independent_variable	0,002794458	0,004614767	0,605547004	0,545083925
ADV5	chr20.21291893.C.T	independent_variable	0,002794458	0,004614767	0,605547004	0,545083925
ADV2	chr1.43568097.C.A	independent_variable	0,000618916	0,001023429	0,604747663	0,545604998
ADV4	chr1.43657831.G.A	independent_variable	0,000324639	0,000538218	0,603174597	0,546645325
ADV2	chr1.43616041.T.G	independent_variable	-0,000653074	0,001083218	-0,602902119	0,546827861
ADV5	chr5.104604069.C.T	independent_variable	0,002769851	0,004595833	0,602687443	0,546975885
ADV3	chr4.111546456.G.A	independent_variable	-0,013037033	0,021633445	-0,602633244	0,547044588
ADV4	chr7.114564074.T.A	independent_variable	0,000302845	0,000502729	0,602401296	0,547171673
ADV2	chr11.28569621.A.T	independent_variable	-0,000628855	0,001044739	-0,601925852	0,547490986
ADV2	chr11.28570040.C.T	independent_variable	-0,000628855	0,001044739	-0,601925852	0,547490986
ADV4	chr5.88574395.G.A	independent_variable	0,000334382	0,000556162	0,601230552	0,547963337
ADV4	chr1.43621825.C.T	independent_variable	0,000321945	0,000535759	0,600913301	0,548152227
ADV5	chr20.21310893.C.T	independent_variable	0,002739765	0,004565241	0,600135813	0,548678172
ADV5	chr20.21311200.C.T	independent_variable	0,002739765	0,004565241	0,600135813	0,548678172
ADV5	chr20.21311590.G.C	independent_variable	0,002739765	0,004565241	0,600135813	0,548678172
ADV5	chr20.21312173.C.T	independent_variable	0,002739765	0,004565241	0,600135813	0,548678172
ADV5	chr20.21312391.G.A	independent_variable	0,002739765	0,004565241	0,600135813	0,548678172
ADV5	chr20.21314871.G.T	independent_variable	0,002739765	0,004565241	0,600135813	0,548678172
ADV5	chr7.114489082.G.A	independent_variable	-0,002707861	0,004513976	-0,599883943	0,548842405
ADV5	chr7.114498696.G.C	independent_variable	-0,002707861	0,004513976	-0,599883943	0,548842405
ADV5	chr7.114502195.A.G	independent_variable	-0,002707861	0,004513976	-0,599883943	0,548842405
ADV4	chr5.104611504.C.T	independent_variable	0,0003487	0,000581399	0,599760966	0,548941099
ADV4	chr5.104612344.C.T	independent_variable	0,0003487	0,000581399	0,599760966	0,548941099
ADV5	chr20.21328174.G.A	independent_variable	0,00272105	0,004548406	0,598242477	0,549938625
ADV4	chr1.43883733.G.A	independent_variable	0,00030401	0,00050818	0,598233235	0,549956289
ADV5	chr5.104619100.T.A	independent_variable	0,002964138	0,00495995	0,597614632	0,55034996
ADV2	chr14.98239063.A.G	independent_variable	0,000569607	0,000953529	0,597367395	0,550520653
ADV4	chr1.43649351.A.G	independent_variable	0,000322451	0,000539878	0,597266661	0,55058064
ADV4	chr1.43637827.A.G	independent_variable	0,000319937	0,00053758	0,59514254	0,551996585
ADV4	chr1.43638043.G.A	independent_variable	0,000319937	0,00053758	0,59514254	0,551996585
ADV4	chr1.43639333.C.T	independent_variable	0,000319937	0,00053758	0,59514254	0,551996585
ADV4	chr1.43640277.T.C	independent_variable	0,000319937	0,00053758	0,59514254	0,551996585
ADV4	chr1.43640595.C.T	independent_variable	0,000319937	0,00053758	0,59514254	0,551996585
ADV4	chr1.43640845.C.A	independent_variable	0,000319937	0,00053758	0,59514254	0,551996585
ADV4	chr1.43641315.G.T	independent_variable	0,000319937	0,00053758	0,59514254	0,551996585
ADV4	chr1.43642285.C.T	independent_variable	0,000319937	0,00053758	0,59514254	0,551996585
ADV4	chr1.43642448.T.A	independent_variable	0,000319937	0,00053758	0,59514254	0,551996585
ADV4	chr1.43643528.A.G	independent_variable	0,000319937	0,00053758	0,59514254	0,551996585
ADV4	chr1.43644545.A.C	independent_variable	0,000319937	0,00053758	0,59514254	0,551996585
ADV4	chr1.43644818.A.G	independent_variable	0,000319937	0,00053758	0,59514254	0,551996585
ADV4	chr1.43645516.A.G	independent_variable	0,000319937	0,00053758	0,59514254	0,551996585
ADV4	chr1.43646708.T.C	independent_variable	0,000319937	0,00053758	0,59514254	0,551996585
ADV4	chr1.43647770.T.C	independent_variable	0,000319937	0,00053758	0,59514254	0,551996585
ADV4	chr1.43648528.G.T	independent_variable	0,000319937	0,00053758	0,59514254	0,551996585
ADV4	chr7.114560479.G.A	independent_variable	0,00028869	0,000486263	0,593690832	0,55297413
ADV4	chr14.98224586.T.A	independent_variable	-0,000332753	0,000560584	-0,593583317	0,553046976
ADV5	chr14.98259646.C.T	independent_variable	-0,003044856	0,005133552	-0,593128423	0,553348218
ADV5	chr7.114469294.A.G	independent_variable	-0,002669865	0,004511234	-0,591825804	0,554222003
ADV4	chr1.43671586.C.A	independent_variable	0,000320331	0,000542414	0,590565634	0,555060411
ADV3	chr3.20678505.C.T	independent_variable	0,009869573	0,016734926	0,58975897	0,555601235
ADV4	chr5.104585069.C.A	independent_variable	0,000345349	0,000586561	0,588768984	0,55628428
ADV5	chr20.21291031.G.C	independent_variable	0,002726676	0,00463137	0,58874079	0,556295827
ADV2	chr2.144956787.A.C	independent_variable	0,00059438	0,001011252	0,587766567	0,556945123

ADV4	chr5.104619660.T.C	independent_variable	0,000342989	0,000584153	0,587156146	0,55736873
ADV5	chr20.21374422.T.A	independent_variable	0,002644931	0,004506052	0,586973091	0,557474102
ADV5	chr20.21375208.A.G	independent_variable	0,002644931	0,004506052	0,586973091	0,557474102
ADV1	chr11.28554714.G.T	independent_variable	0,009800886	0,016883413	0,580503828	0,561851944
ADV4	chr5.104608237.C.G	independent_variable	0,00033752	0,000581864	0,580066359	0,562134341
ADV4	chr5.104610505.A.T	independent_variable	0,00033752	0,000581864	0,580066359	0,562134341
ADV5	chr14.98239063.A.G	independent_variable	-0,003016602	0,005200822	-0,580023989	0,562148232
ADV2	chr1.43663428.T.C	independent_variable	-0,00067043	0,001157602	-0,579154088	0,562743446
ADV4	chr20.21170737.G.A	independent_variable	-0,000325155	0,000562064	-0,578501667	0,563174498
ADV4	chr1.43667345.T.C	independent_variable	0,000314063	0,000542992	0,578393694	0,563240833
ADV2	chr5.88865600.T.C	independent_variable	0,000583794	0,001012193	0,576761608	0,56435154
ADV2	chr5.104556759.C.G	independent_variable	-0,000600382	0,001041846	-0,576267922	0,564684882
ADV2	chr1.43605550.G.A	independent_variable	-0,000636193	0,001104327	-0,576090955	0,564795964
ADV2	chr1.43596124.C.T	independent_variable	-0,0006184	0,001076403	-0,574505387	0,565866439
ADV4	chr5.104597772.C.T	independent_variable	0,000331124	0,0005768	0,574070338	0,566178874
ADV3	chr16.61749670.C.T	independent_variable	-0,010807603	0,018828298	-0,574008514	0,566204183
ADV5	chr1.43778195.T.C	independent_variable	0,002984546	0,005200529	0,573892652	0,566282987
ADV5	chr1.43781562.C.G	independent_variable	0,002984546	0,005200529	0,573892652	0,566282987
ADV3	chr7.114440281.T.G	independent_variable	-0,01025488	0,017871426	-0,573814301	0,566335065
ADV4	chr7.114571392.A.G	independent_variable	0,000277377	0,000484076	0,573001896	0,56688061
ADV5	chr5.104603973.C.T	independent_variable	0,002616968	0,004589256	0,570237959	0,568757509
ADV2	chr12.89378126.T.C	independent_variable	0,000549047	0,000962749	0,570290477	0,568760965
ADV5	chr20.21176870.T.G	independent_variable	-0,002887082	0,005064209	-0,570095299	0,568860715
ADV3	chr20.21270205.T.C	independent_variable	0,009700893	0,017022396	0,56988997	0,569000837
ADV3	chr3.20683444.C.T	independent_variable	0,009519681	0,016707964	0,569769066	0,569073056
ADV5	chr1.43903763.G.A	independent_variable	0,002908369	0,005108574	0,569311396	0,569398465
ADV2	chr1.43606749.A.G	independent_variable	-0,00060854	0,001068963	-0,569280317	0,569401327
ADV5	chr1.43879620.G.A	independent_variable	0,00295928	0,005215545	0,567396028	0,570701858
ADV2	chr1.43682946.A.G	independent_variable	0,000707792	0,001251264	0,56566113	0,571865375
ADV4	chr5.104590343.C.T	independent_variable	0,000329056	0,000584324	0,563139748	0,573592667
ADV3	chr7.114442439.T.C	independent_variable	-0,010070396	0,01788992	-0,562908957	0,573732692
ADV4	chr11.28511111.T.C	independent_variable	0,000313367	0,000559182	0,560402906	0,575471673
ADV4	chr5.104608319.G.T	independent_variable	0,00032597	0,000583865	0,558297711	0,576894565
ADV5	chr20.21154956.G.T	independent_variable	-0,002803143	0,005021514	-0,558226724	0,576926649
ADV4	chr4.111314599.T.C	independent_variable	0,00039849	0,000714254	0,557910565	0,577143732
ADV5	chr5.88569970.A.G	independent_variable	-0,002975762	0,005334575	-0,557825458	0,577214747
ADV5	chr14.98224586.T.A	independent_variable	-0,0029423	0,005279804	-0,557274476	0,577577871
ADV4	chr5.104623644.A.G	independent_variable	0,000325762	0,000585391	0,556486786	0,578131654
ADV4	chr4.111322536.C.G	independent_variable	0,000380024	0,000683007	0,5563979	0,578177307
ADV2	chr5.104559315.C.T	independent_variable	-0,000573251	0,001032467	-0,555225072	0,57898087
ADV2	chr5.104559414.T.C	independent_variable	-0,000573251	0,001032467	-0,555225072	0,57898087
ADV2	chr5.104560429.T.C	independent_variable	-0,000573251	0,001032467	-0,555225072	0,57898087
ADV2	chr5.104562057.T.C	independent_variable	-0,000573251	0,001032467	-0,555225072	0,57898087
ADV2	chr5.104563477.A.G	independent_variable	-0,000573251	0,001032467	-0,555225072	0,57898087
ADV2	chr12.89382507.T.C	independent_variable	0,000533475	0,000961345	0,554926064	0,579216137
ADV5	chr1.43744582.C.G	independent_variable	0,002889009	0,005218639	0,553594258	0,580089577
ADV5	chr1.43745339.A.G	independent_variable	0,002889009	0,005218639	0,553594258	0,580089577
ADV5	chr1.43746026.T.C	independent_variable	0,002889009	0,005218639	0,553594258	0,580089577
ADV5	chr1.43747271.A.G	independent_variable	0,002889009	0,005218639	0,553594258	0,580089577
ADV5	chr1.43747507.C.A	independent_variable	0,002889009	0,005218639	0,553594258	0,580089577
ADV5	chr1.43751205.T.C	independent_variable	0,002889009	0,005218639	0,553594258	0,580089577
ADV5	chr1.43753604.T.C	independent_variable	0,002889009	0,005218639	0,553594258	0,580089577
ADV5	chr1.43763926.G.A	independent_variable	0,002889009	0,005218639	0,553594258	0,580089577
ADV5	chr1.43777612.C.T	independent_variable	0,002889009	0,005218639	0,553594258	0,580089577
ADV2	chr5.88873835.A.G	independent_variable	0,000603588	0,001094983	0,551230747	0,581738571
ADV2	chr5.88874514.T.C	independent_variable	0,000603588	0,001094983	0,551230747	0,581738571
ADV4	chr5.104602414.C.T	independent_variable	0,000273741	0,000497199	0,550567244	0,582162346
ADV3	chr16.61755912.G.C	independent_variable	-0,010329943	0,018820082	-0,54887875	0,583318082
ADV2	chr5.88551455.T.A	independent_variable	0,000607776	0,001108654	0,548210566	0,583803254
ADV2	chr1.43614741.T.C	independent_variable	-0,000602527	0,001100894	-0,547307537	0,584393584
ADV5	chr20.21252915.A.G	independent_variable	0,002583275	0,004728238	0,546350387	0,585059893
ADV4	chr20.21165245.A.G	independent_variable	-0,000290912	0,000533062	-0,545737619	0,585471257
ADV3	chr16.61758377.A.G	independent_variable	-0,010202877	0,018739299	-0,544464155	0,586348117
ADV2	chr5.88433894.A.C	independent_variable	0,000605281	0,001113663	0,543504904	0,587023483
ADV5	chr2.144956787.A.C	independent_variable	-0,002529585	0,004669279	-0,541750794	0,588223135
ADV4	chr5.104610781.C.T	independent_variable	0,00031483	0,000582346	0,540622794	0,589010777
ADV2	chr1.43565822.A.G	independent_variable	0,000553858	0,001024677	0,540519808	0,58906482
ADV2	chr12.89377735.T.C	independent_variable	0,000518009	0,000959947	0,539622609	0,589718998
ADV2	chr1.43685962.T.G	independent_variable	0,000577514	0,001076532	0,536458082	0,591862924
ADV5	chr1.43596483.C.T	independent_variable	-0,002570169	0,004806939	-0,534678845	0,593100931
ADV2	chr1.43616545.G.A	independent_variable	-0,000576219	0,001077938	-0,534556901	0,593176719

ADV2	chr1.43649351.A.G	independent_variable	-0,000580371	0,001087533	-0,53365843	0,59379828
ADV2	chr5.88601453.A.C	independent_variable	0,000632028	0,001185754	0,533018186	0,594264633
ADV2	chr5.104559401.T.G	independent_variable	-0,00055393	0,001040951	-0,532138422	0,594858727
ADV5	chr1.43903772.G.A	independent_variable	0,002583945	0,004857102	0,531993161	0,594961041
ADV2	chr1.43504626.G.A	independent_variable	0,000683093	0,001287183	0,53068856	0,595898166
ADV4	chr11.28504006.A.G	independent_variable	0,000295926	0,000558827	0,529548306	0,596675179
ADV5	chr1.43837594.A.G	independent_variable	-0,00265437	0,005017737	-0,528997525	0,597031601
ADV4	chr1.43712399.T.G	independent_variable	0,000283748	0,000536836	0,528557075	0,597330712
ADV2	chr1.43609834.G.C	independent_variable	-0,000566189	0,001073234	-0,527554394	0,598026143
ADV4	chr5.104609425.A.G	independent_variable	0,000308678	0,000585123	0,527544135	0,598053106
ADV2	chr2.144962572.A.G	independent_variable	0,000532529	0,001012961	0,525714693	0,599311862
ADV5	chr20.21169993.T.C	independent_variable	-0,002731461	0,005201616	-0,525117747	0,599723871
ADV5	chr20.21173142.G.C	independent_variable	-0,002731461	0,005201616	-0,525117747	0,599723871
ADV4	chr11.28522265.G.A	independent_variable	0,000292049	0,000556538	0,524760513	0,599996059
ADV4	chr1.43617344.G.A	independent_variable	0,000285475	0,000544352	0,524430427	0,6001952
ADV2	chr5.88867954.G.A	independent_variable	0,000531387	0,001013475	0,52432172	0,600279946
ADV4	chr1.44009451.C.T	independent_variable	-0,000336472	0,000645094	-0,521585372	0,602200128
ADV4	chr4.111310701.T.C	independent_variable	0,000353838	0,0006787	0,52134685	0,602345855
ADV2	chr1.43574179.A.G	independent_variable	-0,000542484	0,001040865	-0,521185588	0,602453556
ADV5	chr20.21141038.A.T	independent_variable	-0,002612909	0,00501556	-0,520960672	0,602612577
ADV4	chr7.114570759.T.C	independent_variable	0,000251265	0,000482684	0,520557209	0,602894308
ADV5	chr1.43667345.T.C	independent_variable	-0,002636161	0,005070385	-0,519913362	0,603338212
ADV2	chr5.104557287.A.T	independent_variable	-0,000539541	0,001038564	-0,519506766	0,603629302
ADV2	chr3.71450250.C.T	independent_variable	-0,000542023	0,001048455	-0,516972821	0,605390388
ADV3	chr16.61753223.C.T	independent_variable	-0,009686262	0,018744505	-0,516752103	0,605542363
ADV2	chr1.43579857.G.A	independent_variable	-0,000526209	0,001032492	-0,509649676	0,610505903
ADV2	chr1.43583485.G.A	independent_variable	-0,000526209	0,001032492	-0,509649676	0,610505903
ADV2	chr1.43585959.G.A	independent_variable	-0,000526209	0,001032492	-0,509649676	0,610505903
ADV2	chr1.43586100.T.C	independent_variable	-0,000526209	0,001032492	-0,509649676	0,610505903
ADV2	chr5.104550512.T.G	independent_variable	-0,000528744	0,001038655	-0,50906604	0,610923123
ADV4	chr12.89368722.C.G	independent_variable	0,000308459	0,00060664	0,508470804	0,611355368
ADV4	chr12.89369752.C.T	independent_variable	0,000308459	0,00060664	0,508470804	0,611355368
ADV2	chr1.43636043.A.T	independent_variable	-0,00054912	0,001080497	-0,508210752	0,611512324
ADV2	chr1.43636129.A.G	independent_variable	-0,00054912	0,001080497	-0,508210752	0,611512324
ADV2	chr1.43636586.T.C	independent_variable	-0,00054912	0,001080497	-0,508210752	0,611512324
ADV2	chr5.88872578.C.G	independent_variable	0,000550411	0,001091348	0,50433993	0,614259879
ADV2	chr5.88873138.C.A	independent_variable	0,000550411	0,001091348	0,50433993	0,614259879
ADV1	chr11.28522265.G.A	independent_variable	0,0084949	0,016895838	0,502780651	0,615353008
ADV2	chr5.88401026.G.A	independent_variable	0,00056746	0,001129118	0,502569716	0,615479591
ADV4	chr12.89381252.T.G	independent_variable	0,000306687	0,000610521	0,50233634	0,615661318
ADV5	chr1.43671586.C.A	independent_variable	-0,002532926	0,005066308	-0,499954899	0,617312016
ADV4	chr1.43504626.G.A	independent_variable	0,000305318	0,000612016	0,498872556	0,618114577
ADV2	chr1.43537286.G.A	independent_variable	0,000626822	0,001261065	0,497057597	0,619381357
ADV2	chr5.88874249.T.C	independent_variable	0,000544312	0,00109572	0,49676174	0,619590838
ADV5	chr4.111314599.T.C	independent_variable	0,003287561	0,006650864	0,494305782	0,621297398
ADV4	chr11.28554714.G.T	independent_variable	0,000274819	0,000556196	0,494104474	0,621462261
ADV3	chr11.28554714.G.T	independent_variable	-0,008869663	0,017963628	-0,493756787	0,621707633
ADV4	chr5.104563895.T.C	independent_variable	0,000252379	0,000511284	0,493618137	0,62178311
ADV2	chr5.104557847.T.A	independent_variable	-0,00051121	0,001036788	-0,493070598	0,622171658
ADV4	chr1.43903763.G.A	independent_variable	0,000262841	0,000533462	0,492708321	0,622433272
ADV2	chr5.88416702.C.A	independent_variable	0,000554914	0,001127824	0,492021982	0,622911048
ADV4	chr20.21175529.A.G	independent_variable	0,000268921	0,000546687	0,491910676	0,622991283
ADV4	chr20.21176291.G.C	independent_variable	0,000268921	0,000546687	0,491910676	0,622991283
ADV4	chr20.21176925.T.C	independent_variable	0,000268921	0,000546687	0,491910676	0,622991283
ADV4	chr20.21180143.G.A	independent_variable	0,000268921	0,000546687	0,491910676	0,622991283
ADV4	chr20.21183159.A.G	independent_variable	0,000268921	0,000546687	0,491910676	0,622991283
ADV5	chr1.43663428.T.C	independent_variable	-0,002666037	0,005422732	-0,491640943	0,623186813
ADV4	chr5.88601453.A.C	independent_variable	0,000277338	0,000564766	0,491067785	0,623600175
ADV2	chr1.43600753.C.A	independent_variable	-0,000508763	0,001037476	-0,490384772	0,62406121
ADV2	chr1.43853701.G.A	independent_variable	0,000530676	0,001082224	0,490357227	0,624078852
ADV2	chr1.43671586.C.A	independent_variable	-0,000529856	0,001088571	-0,486744984	0,626638032
ADV4	chr5.88385177.A.G	independent_variable	-0,000275997	0,000567989	-0,485919867	0,627229464
ADV4	chr5.88389683.C.T	independent_variable	-0,000275997	0,000567989	-0,485919867	0,627229464
ADV2	chr5.104606092.C.A	independent_variable	-0,00049117	0,001011634	-0,485521283	0,627508022
ADV2	chr5.88871923.G.C	independent_variable	0,000526872	0,001089272	0,483692243	0,628830051
ADV4	chr1.43596483.C.T	independent_variable	-0,000242854	0,00050299	-0,48282078	0,629426584
ADV2	chr1.43596483.C.T	independent_variable	-0,000500437	0,00103838	-0,481940621	0,630051293
ADV2	chr1.43605020.A.G	independent_variable	-0,000500244	0,001040239	-0,480893412	0,630787759
ADV4	chr20.21177599.C.G	independent_variable	0,000264044	0,000549074	0,480889466	0,630799291
ADV4	chr20.21177731.T.G	independent_variable	0,000264044	0,000549074	0,480889466	0,630799291
ADV4	chr20.21178512.T.C	independent_variable	0,000264044	0,000549074	0,480889466	0,630799291

ADV5	chr2.144962572.A.G	independent_variable	-0,002248654	0,004685424	-0,479925424	0,631483397
ADV5	chr1.43712399.T.G	independent_variable	-0,002410578	0,005041689	-0,478129031	0,63275259
ADV4	chr1.43689712.A.G	independent_variable	0,000260907	0,000548559	0,475622208	0,634535536
ADV2	chr5.104554395.C.T	independent_variable	-0,00049352	0,001037697	-0,475591723	0,634565766
ADV2	chr5.88417014.A.G	independent_variable	0,000538335	0,001133346	0,474996237	0,634990249
ADV2	chr1.43629437.A.G	independent_variable	-0,000514535	0,001084221	-0,474566359	0,635288298
ADV2	chr1.43631843.C.G	independent_variable	-0,000514535	0,001084221	-0,474566359	0,635288298
ADV2	chr1.43634192.G.T	independent_variable	-0,000514535	0,001084221	-0,474566359	0,635288298
ADV2	chr1.43634239.T.C	independent_variable	-0,000514535	0,001084221	-0,474566359	0,635288298
ADV5	chr20.21175938.T.C	independent_variable	-0,002380511	0,005023215	-0,473901808	0,635770382
ADV5	chr20.21179524.A.C	independent_variable	-0,002380511	0,005023215	-0,473901808	0,635770382
ADV2	chr1.43667345.T.C	independent_variable	-0,000517068	0,001093196	-0,472987481	0,636414303
ADV4	chr5.88401026.G.A	independent_variable	-0,000266302	0,00056534	-0,471047458	0,637804389
ADV4	chr12.89364160.G.A	independent_variable	0,000283039	0,000600998	0,470948365	0,637888052
ADV4	chr12.89366967.A.G	independent_variable	0,000283039	0,000600998	0,470948365	0,637888052
ADV2	chr1.43637827.A.G	independent_variable	-0,000508923	0,001082673	-0,4700617	0,638500012
ADV2	chr1.43638043.G.A	independent_variable	-0,000508923	0,001082673	-0,4700617	0,638500012
ADV2	chr1.43639333.C.T	independent_variable	-0,000508923	0,001082673	-0,4700617	0,638500012
ADV2	chr1.43640277.T.C	independent_variable	-0,000508923	0,001082673	-0,4700617	0,638500012
ADV2	chr1.43640595.C.T	independent_variable	-0,000508923	0,001082673	-0,4700617	0,638500012
ADV2	chr1.43640845.C.A	independent_variable	-0,000508923	0,001082673	-0,4700617	0,638500012
ADV2	chr1.43641315.G.T	independent_variable	-0,000508923	0,001082673	-0,4700617	0,638500012
ADV2	chr1.43642285.C.T	independent_variable	-0,000508923	0,001082673	-0,4700617	0,638500012
ADV2	chr1.43642448.T.A	independent_variable	-0,000508923	0,001082673	-0,4700617	0,638500012
ADV2	chr1.43643528.A.G	independent_variable	-0,000508923	0,001082673	-0,4700617	0,638500012
ADV2	chr1.43644545.A.C	independent_variable	-0,000508923	0,001082673	-0,4700617	0,638500012
ADV2	chr1.43644818.A.G	independent_variable	-0,000508923	0,001082673	-0,4700617	0,638500012
ADV2	chr1.43645516.A.G	independent_variable	-0,000508923	0,001082673	-0,4700617	0,638500012
ADV2	chr1.43646708.T.C	independent_variable	-0,000508923	0,001082673	-0,4700617	0,638500012
ADV2	chr1.43647770.T.C	independent_variable	-0,000508923	0,001082673	-0,4700617	0,638500012
ADV2	chr1.43648528.G.T	independent_variable	-0,000508923	0,001082673	-0,4700617	0,638500012
ADV5	chr1.43649351.A.G	independent_variable	-0,002352616	0,005032455	-0,467488746	0,640339534
ADV5	chr1.43541977.T.C	independent_variable	-0,002546307	0,005451291	-0,467101724	0,640658321
ADV4	chr5.104642625.G.A	independent_variable	0,000269429	0,000578523	0,465719648	0,64162103
ADV2	chr1.43617344.G.A	independent_variable	0,000509607	0,001096453	0,464777746	0,642278668
ADV4	chr20.21176320.A.G	independent_variable	0,000253577	0,000547656	0,463022134	0,643543338
ADV5	chr1.43548609.T.C	independent_variable	-0,002521326	0,00544895	-0,462717787	0,643794382
ADV5	chr1.43682946.A.G	independent_variable	-0,002636248	0,005703942	-0,462180095	0,644143283
ADV5	chr7.114434202.C.T	independent_variable	-0,002204036	0,004773285	-0,461744078	0,644457222
ADV4	chr5.104629522.A.C	independent_variable	0,000267744	0,000579915	0,461696412	0,64450311
ADV2	chr12.89364160.G.A	independent_variable	0,000552489	0,00119729	0,461449689	0,644681622
ADV2	chr12.89366967.A.G	independent_variable	0,000552489	0,00119729	0,461449689	0,644681622
ADV2	chr1.43548902.A.T	independent_variable	-0,000525752	0,001139855	-0,461244608	0,644848989
ADV4	chr5.88873835.A.G	independent_variable	0,000241766	0,000525634	0,459950914	0,645765059
ADV4	chr5.88874514.T.C	independent_variable	0,000241766	0,000525634	0,459950914	0,645765059
ADV3	chr5.88397820.G.C	independent_variable	0,008757556	0,019041881	0,459910233	0,645773628
ADV2	chr1.43657831.G.A	independent_variable	-0,000497453	0,001083908	-0,458944232	0,646458544
ADV4	chr5.104577563.A.C	independent_variable	0,00022735	0,000497155	0,457301987	0,647641038
ADV4	chr5.104577567.C.T	independent_variable	0,00022735	0,000497155	0,457301987	0,647641038
ADV4	chr5.104577620.T.A	independent_variable	0,00022735	0,000497155	0,457301987	0,647641038
ADV4	chr5.104578055.A.T	independent_variable	0,00022735	0,000497155	0,457301987	0,647641038
ADV4	chr5.104578313.C.T	independent_variable	0,00022735	0,000497155	0,457301987	0,647641038
ADV4	chr5.104578842.G.A	independent_variable	0,00022735	0,000497155	0,457301987	0,647641038
ADV4	chr5.104579057.A.G	independent_variable	0,00022735	0,000497155	0,457301987	0,647641038
ADV4	chr5.104579085.A.T	independent_variable	0,00022735	0,000497155	0,457301987	0,647641038
ADV4	chr5.104579135.T.G	independent_variable	0,00022735	0,000497155	0,457301987	0,647641038
ADV4	chr5.104579581.A.G	independent_variable	0,00022735	0,000497155	0,457301987	0,647641038
ADV4	chr5.104579742.G.A	independent_variable	0,00022735	0,000497155	0,457301987	0,647641038
ADV4	chr5.104580027.A.G	independent_variable	0,00022735	0,000497155	0,457301987	0,647641038
ADV4	chr5.104580648.T.C	independent_variable	0,00022735	0,000497155	0,457301987	0,647641038
ADV4	chr5.104580662.G.C	independent_variable	0,00022735	0,000497155	0,457301987	0,647641038
ADV4	chr5.104581130.A.G	independent_variable	0,00022735	0,000497155	0,457301987	0,647641038
ADV2	chr1.43848594.C.T	independent_variable	0,000489516	0,001071439	0,456876745	0,647947185
ADV4	chr5.104569213.T.C	independent_variable	0,000229817	0,000505249	0,454859242	0,649398533
ADV5	chr1.43549278.T.C	independent_variable	-0,002478047	0,00545871	-0,453961972	0,650079525
ADV5	chr5.104623644.A.G	independent_variable	-0,002349577	0,005176294	-0,453911162	0,650093336
ADV4	chr5.104577501.A.G	independent_variable	0,000226463	0,000498972	0,453859759	0,650115908
ADV4	chr1.43685344.T.G	independent_variable	0,000247351	0,000546659	0,452478515	0,651104678
ADV5	chr1.43600753.C.A	independent_variable	-0,002158799	0,004779881	-0,451642915	0,651707923
ADV2	chr1.43853335.A.G	independent_variable	0,000488265	0,00109522	0,445814484	0,655910276
ADV4	chr5.104584139.G.A	independent_variable	0,000221128	0,000496381	0,445481326	0,656153171

ADV5	chr1.43568097.C.A	independent_variable	0,002318399	0,005206201	0,445314946	0,656273653
ADV5	chr5.104609425.A.G	independent_variable	-0,002297496	0,005175696	-0,443900918	0,657308986
ADV2	chr5.104605369.T.G	independent_variable	-0,000446242	0,001010307	-0,441689237	0,658894932
ADV2	chr5.104605700.T.C	independent_variable	-0,000446242	0,001010307	-0,441689237	0,658894932
ADV2	chr5.104605714.T.G	independent_variable	-0,000446242	0,001010307	-0,441689237	0,658894932
ADV4	chr5.88456878.A.G	independent_variable	-0,000251899	0,000571442	-0,440812432	0,659532702
ADV4	chr5.88461004.C.T	independent_variable	-0,000251899	0,000571442	-0,440812432	0,659532702
ADV4	chr5.88467699.G.A	independent_variable	-0,000251899	0,000571442	-0,440812432	0,659532702
ADV4	chr5.88469954.T.C	independent_variable	-0,000251899	0,000571442	-0,440812432	0,659532702
ADV4	chr5.88475276.A.C	independent_variable	-0,000251899	0,000571442	-0,440812432	0,659532702
ADV4	chr5.88477838.A.G	independent_variable	-0,000251899	0,000571442	-0,440812432	0,659532702
ADV2	chr1.43718252.C.T	independent_variable	0,000474665	0,001078188	0,440243184	0,659937474
ADV2	chr1.43707752.G.A	independent_variable	0,000469734	0,001069592	0,439171397	0,660712701
ADV2	chr1.43722794.A.C	independent_variable	0,000469734	0,001069592	0,439171397	0,660712701
ADV2	chr1.43723048.A.G	independent_variable	0,000469734	0,001069592	0,439171397	0,660712701
ADV5	chr5.88433894.A.C	independent_variable	-0,00224702	0,005128928	-0,438107108	0,661497096
ADV2	chr1.43716573.A.G	independent_variable	0,00047157	0,001076794	0,437939363	0,66160536
ADV5	chr20.21189014.G.A	independent_variable	-0,002075011	0,004748092	-0,43701995	0,662278302
ADV5	chr1.43831901.A.C	independent_variable	-0,002180446	0,004997011	-0,436350029	0,662763203
ADV4	chr4.111251830.C.A	independent_variable	0,000380708	0,000872582	0,436300223	0,662798623
ADV4	chr1.43644078.A.G	independent_variable	0,000235796	0,000540673	0,436115009	0,662927907
ADV4	chr5.88416702.C.A	independent_variable	-0,000246089	0,000565578	-0,435109921	0,663662823
ADV5	chr7.114435631.A.C	independent_variable	-0,002072974	0,004766746	-0,434882322	0,663827576
ADV5	chr1.43637827.A.G	independent_variable	-0,002178245	0,005019906	-0,433921383	0,664518198
ADV5	chr1.43638043.G.A	independent_variable	-0,002178245	0,005019906	-0,433921383	0,664518198
ADV5	chr1.43639333.C.T	independent_variable	-0,002178245	0,005019906	-0,433921383	0,664518198
ADV5	chr1.43640277.T.C	independent_variable	-0,002178245	0,005019906	-0,433921383	0,664518198
ADV5	chr1.43640595.C.T	independent_variable	-0,002178245	0,005019906	-0,433921383	0,664518198
ADV5	chr1.43640845.C.A	independent_variable	-0,002178245	0,005019906	-0,433921383	0,664518198
ADV5	chr1.43641315.G.T	independent_variable	-0,002178245	0,005019906	-0,433921383	0,664518198
ADV5	chr1.43642285.C.T	independent_variable	-0,002178245	0,005019906	-0,433921383	0,664518198
ADV5	chr1.43642448.T.A	independent_variable	-0,002178245	0,005019906	-0,433921383	0,664518198
ADV5	chr1.43643528.A.G	independent_variable	-0,002178245	0,005019906	-0,433921383	0,664518198
ADV5	chr1.43644545.A.C	independent_variable	-0,002178245	0,005019906	-0,433921383	0,664518198
ADV5	chr1.43644818.A.G	independent_variable	-0,002178245	0,005019906	-0,433921383	0,664518198
ADV5	chr1.43645516.A.G	independent_variable	-0,002178245	0,005019906	-0,433921383	0,664518198
ADV5	chr1.43646708.T.C	independent_variable	-0,002178245	0,005019906	-0,433921383	0,664518198
ADV5	chr1.43647770.T.C	independent_variable	-0,002178245	0,005019906	-0,433921383	0,664518198
ADV5	chr1.43648528.G.T	independent_variable	-0,002178245	0,005019906	-0,433921383	0,664518198
ADV4	chr5.104581651.A.C	independent_variable	0,000214948	0,000495609	0,433703434	0,664679295
ADV4	chr5.104582166.G.T	independent_variable	0,000214948	0,000495609	0,433703434	0,664679295
ADV4	chr5.104582921.C.T	independent_variable	0,000214948	0,000495609	0,433703434	0,664679295
ADV4	chr5.104582998.G.A	independent_variable	0,000214948	0,000495609	0,433703434	0,664679295
ADV4	chr5.104583048.G.T	independent_variable	0,000214948	0,000495609	0,433703434	0,664679295
ADV4	chr5.104584121.A.C	independent_variable	0,000214948	0,000495609	0,433703434	0,664679295
ADV4	chr5.104576413.A.C	independent_variable	0,000216041	0,000501302	0,430958534	0,666674735
ADV2	chr1.43599477.C.T	independent_variable	-0,000446175	0,001035516	-0,430872663	0,666732895
ADV5	chr5.104610781.C.T	independent_variable	-0,002219573	0,005151089	-0,430893997	0,666733019
ADV2	chr1.43621825.C.T	independent_variable	-0,00046366	0,001079113	-0,429668062	0,667610112
ADV2	chr11.28659176.C.A	independent_variable	-0,000438267	0,001022957	-0,428431224	0,668509629
ADV5	chr4.111322536.C.G	independent_variable	0,002801793	0,006556433	0,427334949	0,669311597
ADV2	chr11.28646737.A.G	independent_variable	-0,000435841	0,001020414	-0,427121758	0,669462485
ADV2	chr11.28662674.G.A	independent_variable	-0,000435776	0,001020414	-0,42705757	0,669509206
ADV2	chr11.28664505.C.T	independent_variable	-0,000435776	0,001020414	-0,42705757	0,669509206
ADV2	chr11.28666219.T.C	independent_variable	-0,000435776	0,001020414	-0,42705757	0,669509206
ADV2	chr11.28668805.C.G	independent_variable	-0,000435776	0,001020414	-0,42705757	0,669509206
ADV2	chr11.28669587.A.G	independent_variable	-0,000435776	0,001020414	-0,42705757	0,669509206
ADV4	chr5.104576507.C.A	independent_variable	0,000212988	0,000499236	0,426627987	0,669823871
ADV1	chr11.28511111.T.C	independent_variable	0,007233615	0,016963347	0,426426158	0,669992582
ADV5	chr1.43657831.G.A	independent_variable	-0,002140018	0,005025982	-0,425791057	0,670429441
ADV4	chr1.43720773.T.A	independent_variable	0,000234457	0,000551694	0,424976253	0,671023272
ADV3	chr11.28522265.G.A	independent_variable	-0,007633377	0,017976454	-0,424631984	0,671298464
ADV5	chr1.43840448.A.G	independent_variable	-0,002110069	0,004971949	-0,424394837	0,671451079
ADV5	chr1.43845072.C.T	independent_variable	-0,002110069	0,004971949	-0,424394837	0,671451079
ADV5	chr1.43845203.G.A	independent_variable	-0,002110069	0,004971949	-0,424394837	0,671451079
ADV2	chr5.88574395.G.A	independent_variable	0,000495636	0,001168535	0,424151648	0,671640612
ADV5	chr1.43623961.A.G	independent_variable	-0,002135151	0,005034358	-0,42411575	0,671650927
ADV4	chr5.104582527.C.T	independent_variable	0,000208807	0,000494842	0,421967973	0,67321822
ADV4	chr20.21154158.T.C	independent_variable	0,000222865	0,000531224	0,41953051	0,674994084
ADV2	chr5.104568407.A.G	independent_variable	-0,000432652	0,001032006	-0,419233556	0,675218577
ADV4	chr20.21228388.C.T	independent_variable	0,000224974	0,000536815	0,41909155	0,675320639

ADV2	chr12.89381252.T.G	independent_variable	0,000502468	0,001201213	0,418300248	0,675914689
ADV4	chr5.88386213.G.A	independent_variable	-0,00023689	0,000566967	-0,417819767	0,676251701
ADV5	chr1.43705540.C.T	independent_variable	-0,002384814	0,005712477	-0,417474652	0,676500963
ADV5	chr1.43609834.G.C	independent_variable	-0,002068104	0,004978086	-0,415441621	0,677984282
ADV2	chr5.104629522.A.C	independent_variable	-0,000480091	0,001157112	-0,414904287	0,678392829
ADV4	chr20.21422129.C.A	independent_variable	-0,000213337	0,000514328	-0,414786821	0,678463622
ADV2	chr11.28648922.G.A	independent_variable	-0,000422326	0,001019119	-0,414402712	0,678744993
ADV2	chr11.28654958.T.C	independent_variable	-0,000422326	0,001019119	-0,414402712	0,678744993
ADV2	chr11.28655314.A.G	independent_variable	-0,000422326	0,001019119	-0,414402712	0,678744993
ADV2	chr11.28657323.A.G	independent_variable	-0,000422326	0,001019119	-0,414402712	0,678744993
ADV5	chr1.43592279.T.C	independent_variable	0,00211119	0,005097294	0,414178575	0,678909021
ADV2	chr11.28650082.G.C	independent_variable	-0,000421423	0,00102058	-0,412924491	0,679827372
ADV5	chr1.43563291.G.A	independent_variable	-0,00208606	0,005057815	-0,412442889	0,680179744
ADV5	chr1.43579794.A.G	independent_variable	0,002126969	0,005169736	0,411427039	0,680924812
ADV4	chr5.104571636.G.A	independent_variable	0,000209308	0,000509632	0,410703824	0,681457934
ADV4	chr5.88397820.G.C	independent_variable	-0,000232254	0,000567666	-0,409139538	0,682606372
ADV2	chr1.43662376.A.G	independent_variable	-0,000441993	0,001083046	-0,408101896	0,68336063
ADV2	chr1.43631767.G.T	independent_variable	-0,000429433	0,001052561	-0,407989215	0,683443006
ADV4	chr1.43739423.T.C	independent_variable	0,000224391	0,000552102	0,406430325	0,684588967
ADV5	chr1.43662376.A.G	independent_variable	-0,002036037	0,005022222	-0,405405573	0,685339983
ADV5	chr1.43636043.A.T	independent_variable	-0,002029067	0,005010104	-0,404994898	0,685641055
ADV5	chr1.43636129.A.G	independent_variable	-0,002029067	0,005010104	-0,404994898	0,685641055
ADV5	chr1.43636586.T.C	independent_variable	-0,002029067	0,005010104	-0,404994898	0,685641055
ADV5	chr1.43565986.C.T	independent_variable	-0,002046914	0,005064031	-0,404206417	0,686222467
ADV2	chr1.43712399.T.G	independent_variable	-0,000439622	0,001087656	-0,404191773	0,686232328
ADV4	chr20.21400134.C.A	independent_variable	-0,000218731	0,000541729	-0,403763577	0,686549132
ADV5	chr1.43616202.A.C	independent_variable	-0,002024427	0,005018564	-0,403387726	0,686822985
ADV5	chr1.43617439.C.T	independent_variable	-0,002024427	0,005018564	-0,403387726	0,686822985
ADV5	chr1.43617836.T.C	independent_variable	-0,002024427	0,005018564	-0,403387726	0,686822985
ADV5	chr1.43617848.G.A	independent_variable	-0,002024427	0,005018564	-0,403387726	0,686822985
ADV5	chr1.43618203.A.G	independent_variable	-0,002024427	0,005018564	-0,403387726	0,686822985
ADV5	chr1.43619068.C.T	independent_variable	-0,002024427	0,005018564	-0,403387726	0,686822985
ADV5	chr1.43624269.A.G	independent_variable	-0,002024427	0,005018564	-0,403387726	0,686822985
ADV5	chr1.43625004.T.C	independent_variable	-0,002024427	0,005018564	-0,403387726	0,686822985
ADV5	chr1.43625065.C.T	independent_variable	-0,002024427	0,005018564	-0,403387726	0,686822985
ADV5	chr1.43625603.A.G	independent_variable	-0,002024427	0,005018564	-0,403387726	0,686822985
ADV5	chr4.111310701.T.C	independent_variable	0,002618243	0,006514692	0,401898153	0,687923166
ADV4	chr1.43717869.G.T	independent_variable	0,000221524	0,000551564	0,401629036	0,688115955
ADV2	chr1.43569801.G.A	independent_variable	-0,000415741	0,001035801	-0,401371524	0,688307782
ADV5	chr7.114578527.C.T	independent_variable	0,001953247	0,004881633	0,400121669	0,689232816
ADV5	chr7.114583668.C.A	independent_variable	0,001953247	0,004881633	0,400121669	0,689232816
ADV5	chr7.114584108.A.G	independent_variable	0,001953247	0,004881633	0,400121669	0,689232816
ADV4	chr5.104596006.G.A	independent_variable	0,00023149	0,000578855	0,39991047	0,689393932
ADV2	chr1.43566122.G.C	independent_variable	-0,000413497	0,00103439	-0,399749312	0,689501011
ADV5	chr1.43631767.G.T	independent_variable	-0,0019329	0,004848171	-0,398686357	0,690281629
ADV5	chr5.104629522.A.C	independent_variable	-0,0020412	0,005120652	-0,398621075	0,690345368
ADV5	chr7.114518899.G.A	independent_variable	0,00196612	0,004941167	0,397906037	0,690863066
ADV1	chr3.20680838.A.C	independent_variable	-0,006264382	0,015748149	-0,39778529	0,690947132
ADV5	chr1.43616041.T.G	independent_variable	-0,001985396	0,005024716	-0,395126028	0,692906492
ADV5	chr1.43620409.T.C	independent_variable	-0,001985396	0,005024716	-0,395126028	0,692906492
ADV3	chr5.88386213.G.A	independent_variable	0,007548874	0,019120103	0,394813464	0,693142757
ADV4	chr7.114381815.T.C	independent_variable	0,000197288	0,000501391	0,393481095	0,69412065
ADV2	chr5.104568333.A.G	independent_variable	-0,000398587	0,001013742	-0,393184137	0,694343705
ADV2	chr5.104568336.T.C	independent_variable	-0,000398587	0,001013742	-0,393184137	0,694343705
ADV2	chr11.28634517.A.T	independent_variable	-0,000400824	0,001020158	-0,392903713	0,694547102
ADV2	chr11.28634687.C.A	independent_variable	-0,000400824	0,001020158	-0,392903713	0,694547102
ADV2	chr11.28644991.C.T	independent_variable	-0,000400824	0,001020158	-0,392903713	0,694547102
ADV4	chr1.43756926.G.A	independent_variable	0,000219937	0,000561152	0,39193886	0,695261234
ADV4	chr20.21176870.T.G	independent_variable	0,000212182	0,000543957	0,390071395	0,696644406
ADV2	chr5.88629003.C.T	independent_variable	-0,00043586	0,001121107	-0,388776577	0,69760726
ADV5	chr1.43584333.G.A	independent_variable	0,00197435	0,00510803	0,386518976	0,699265743
ADV5	chr1.43584356.A.C	independent_variable	0,00197435	0,00510803	0,386518976	0,699265743
ADV5	chr1.43586706.T.G	independent_variable	0,00197435	0,00510803	0,386518976	0,699265743
ADV2	chr1.43831901.A.C	independent_variable	0,000422885	0,001095947	0,385862127	0,699755988
ADV2	chr1.43577721.G.A	independent_variable	-0,000399004	0,001036513	-0,38494873	0,700428387
ADV5	chr1.43549154.A.G	independent_variable	-0,0020716	0,0054323	-0,381348501	0,703127913
ADV5	chr1.43629437.A.G	independent_variable	-0,001913202	0,005028406	-0,380478814	0,703739916
ADV5	chr1.43631843.C.G	independent_variable	-0,001913202	0,005028406	-0,380478814	0,703739916
ADV5	chr1.43634192.G.T	independent_variable	-0,001913202	0,005028406	-0,380478814	0,703739916
ADV5	chr1.43634239.T.C	independent_variable	-0,001913202	0,005028406	-0,380478814	0,703739916
ADV5	chr20.21165245.A.G	independent_variable	-0,001892432	0,004978535	-0,380118186	0,704006872

ADV2	chr11.28658596.T.C	independent_variable	-0,000389527	0,001024926	-0,380053574	0,704057899
ADV2	chr1.43843195.A.G	independent_variable	0,000409406	0,001080813	0,378794452	0,704993354
ADV5	chr1.43596124.C.T	independent_variable	-0,001873888	0,004993023	-0,375301346	0,707584554
ADV4	chr1.43509093.G.T	independent_variable	-0,000217646	0,000579972	-0,37526991	0,707630853
ADV4	chr1.43510950.G.C	independent_variable	-0,000217646	0,000579972	-0,37526991	0,707630853
ADV5	chr7.114564074.T.A	independent_variable	0,001825864	0,004867749	0,375094017	0,70774467
ADV5	chr1.43569801.G.A	independent_variable	0,001804339	0,004810665	0,375070536	0,707757488
ADV5	chr1.43550138.T.C	independent_variable	-0,002058548	0,005523074	-0,372717891	0,7095383
ADV2	chr5.88526854.T.A	independent_variable	-0,000449329	0,001208265	-0,371879974	0,710144636
ADV2	chr1.43570414.G.A	independent_variable	-0,000386642	0,001042314	-0,370946006	0,710825135
ADV2	chr1.43554060.C.T	independent_variable	-0,000391283	0,001060572	-0,368936155	0,712323769
ADV2	chr1.43556356.A.G	independent_variable	-0,000391283	0,001060572	-0,368936155	0,712323769
ADV5	chr1.43840750.C.T	independent_variable	-0,001826077	0,004954123	-0,368597365	0,712575883
ADV4	chr5.88879434.C.G	independent_variable	0,000194058	0,000528781	0,366991911	0,713792721
ADV5	chr1.43569422.A.G	independent_variable	-0,001843047	0,005038351	-0,365803565	0,714655982
ADV2	chr5.104603973.C.T	independent_variable	-0,000366728	0,00100265	-0,365758363	0,714691053
ADV5	chr20.21146082.A.G	independent_variable	-0,001835548	0,005043972	-0,363909149	0,716071802
ADV3	chr11.28511111.T.C	independent_variable	-0,006564877	0,018055015	-0,36360408	0,716317173
ADV2	chr1.43655886.C.G	independent_variable	-0,000378418	0,00104393	-0,362494034	0,717123925
ADV1	chr3.20685150.G.A	independent_variable	-0,006253241	0,017266123	-0,362168195	0,717391232
ADV1	chr3.20685154.C.T	independent_variable	-0,006253241	0,017266123	-0,362168195	0,717391232
ADV3	chr5.88385177.A.G	independent_variable	0,006896969	0,019187187	0,359457022	0,719399711
ADV3	chr5.88389683.C.T	independent_variable	0,006896969	0,019187187	0,359457022	0,719399711
ADV4	chr5.104566479.C.T	independent_variable	0,000180941	0,000505132	0,358205745	0,720333604
ADV4	chr5.104567257.C.T	independent_variable	0,000180941	0,000505132	0,358205745	0,720333604
ADV4	chr5.104572545.C.T	independent_variable	0,000180941	0,000505132	0,358205745	0,720333604
ADV4	chr5.104572709.A.G	independent_variable	0,000180941	0,000505132	0,358205745	0,720333604
ADV4	chr5.104573167.C.T	independent_variable	0,000180941	0,000505132	0,358205745	0,720333604
ADV4	chr5.104573847.C.T	independent_variable	0,000180941	0,000505132	0,358205745	0,720333604
ADV5	chr20.21138467.C.G	independent_variable	-0,001829078	0,005113125	-0,357722038	0,720694561
ADV5	chr20.21140567.A.T	independent_variable	-0,001829078	0,005113125	-0,357722038	0,720694561
ADV2	chr1.43596488.C.T	independent_variable	-0,000371307	0,001038465	-0,357554182	0,720816674
ADV2	chr1.43610798.G.A	independent_variable	0,000377388	0,001058309	0,356595133	0,721533955
ADV5	chr5.88874249.T.C	independent_variable	-0,001726524	0,0048422	-0,356557715	0,721583977
ADV1	chr11.28504006.A.G	independent_variable	0,006036724	0,016952954	0,356086869	0,721935291
ADV5	chr7.114571857.G.A	independent_variable	0,001745006	0,004901474	0,356016508	0,721974561
ADV5	chr11.28617583.T.C	independent_variable	-0,001689887	0,00475545	-0,355357956	0,722466094
ADV1	chr7.114451698.G.A	independent_variable	0,005902682	0,016706291	0,353320918	0,723984991
ADV5	chr1.43555785.C.G	independent_variable	0,001829863	0,005187598	0,352738005	0,724424049
ADV2	chr5.104604069.C.T	independent_variable	-0,000356139	0,001010433	-0,35246172	0,724632933
ADV4	chr5.88445076.T.C	independent_variable	-0,000201051	0,000570696	-0,352289918	0,724764965
ADV4	chr5.88468801.G.A	independent_variable	-0,000201051	0,000570696	-0,352289918	0,724764965
ADV2	chr1.43510486.G.A	independent_variable	0,000459131	0,001303728	0,352167761	0,724877318
ADV2	chr5.104565723.C.T	independent_variable	-0,000360697	0,001025153	-0,351846966	0,725094751
ADV2	chr5.104568065.G.A	independent_variable	-0,000360697	0,001025153	-0,351846966	0,725094751
ADV5	chr7.114647061.T.A	independent_variable	0,0017906	0,005092337	0,351626389	0,72526456
ADV4	chr20.21074404.T.C	independent_variable	-0,00018745	0,000533341	-0,351464308	0,725381054
ADV2	chr12.89368722.C.G	independent_variable	0,00041791	0,001194087	0,349982618	0,726504302
ADV2	chr12.89369752.C.T	independent_variable	0,00041791	0,001194087	0,349982618	0,726504302
ADV4	chr5.104603973.C.T	independent_variable	0,000174463	0,000499101	0,349554317	0,726811787
ADV4	chr5.88864537.C.T	independent_variable	0,000173347	0,000496265	0,34930357	0,727001745
ADV2	chr5.104609425.A.G	independent_variable	-0,000407379	0,001167532	-0,348923749	0,727295525
ADV5	chr1.43616545.G.A	independent_variable	-0,001740226	0,004999658	-0,34806899	0,727924267
ADV4	chr1.43543362.C.T	independent_variable	-0,000186303	0,00053613	-0,347495617	0,728383675
ADV4	chr7.114384998.A.G	independent_variable	0,000173967	0,000501819	0,346671962	0,728974341
ADV1	chr7.114482451.C.T	independent_variable	0,005828334	0,016845557	0,345986415	0,729487929
ADV2	chr1.43840750.C.T	independent_variable	0,000375632	0,001087273	0,345480912	0,729870716
ADV2	chr5.104566479.C.T	independent_variable	-0,000349047	0,001014498	-0,344058642	0,730940208
ADV2	chr5.104567257.C.T	independent_variable	-0,000349047	0,001014498	-0,344058642	0,730940208
ADV2	chr5.104572545.C.T	independent_variable	-0,000349047	0,001014498	-0,344058642	0,730940208
ADV2	chr5.104572709.A.G	independent_variable	-0,000349047	0,001014498	-0,344058642	0,730940208
ADV2	chr5.104573167.C.T	independent_variable	-0,000349047	0,001014498	-0,344058642	0,730940208
ADV2	chr5.104573847.C.T	independent_variable	-0,000349047	0,001014498	-0,344058642	0,730940208
ADV5	chr1.43575295.T.G	independent_variable	-0,001731169	0,005045185	-0,343132934	0,73163375
ADV5	chr1.43554723.T.C	independent_variable	0,001752309	0,005153654	0,340012823	0,733980072
ADV5	chr1.43556863.C.T	independent_variable	0,001752309	0,005153654	0,340012823	0,733980072
ADV2	chr11.28626638.C.T	independent_variable	-0,000353445	0,001041465	-0,339372816	0,734464092
ADV2	chr11.28626783.A.T	independent_variable	-0,000353445	0,001041465	-0,339372816	0,734464092
ADV2	chr11.28626831.G.C	independent_variable	-0,000353445	0,001041465	-0,339372816	0,734464092
ADV4	chr5.104568333.A.G	independent_variable	0,000170827	0,000506261	0,337429088	0,73592884
ADV4	chr5.104568336.T.C	independent_variable	0,000170827	0,000506261	0,337429088	0,73592884

ADV4	chr1.43726088.C.T	independent_variable	0,00018453	0,000547504	0,337039144	0,736218589
ADV4	chr1.43731274.C.T	independent_variable	0,00018453	0,000547504	0,337039144	0,736218589
ADV2	chr1.43550344.G.A	independent_variable	0,00038784	0,001155185	0,335738716	0,7372079
ADV5	chr7.114608796.A.G	independent_variable	0,001683817	0,005037132	0,334280938	0,738301321
ADV2	chr1.43837594.A.G	independent_variable	0,000367067	0,001100717	0,333479452	0,738906051
ADV2	chr1.43586107.G.A	independent_variable	-0,000346645	0,001041237	-0,332916757	0,739326569
ADV2	chr1.43652735.G.A	independent_variable	-0,000348965	0,0010489	-0,3326962	0,739492021
ADV4	chr5.88497027.G.A	independent_variable	0,000185119	0,000556709	0,33252308	0,739637692
ADV2	chr16.61754062.A.G	independent_variable	-0,000327216	0,000989052	-0,33083762	0,740894531
ADV4	chr5.104563989.T.C	independent_variable	0,0001671	0,000505444	0,330600552	0,741078375
ADV4	chr5.104569324.T.A	independent_variable	0,0001671	0,000505444	0,330600552	0,741078375
ADV4	chr5.104569574.C.T	independent_variable	0,0001671	0,000505444	0,330600552	0,741078375
ADV4	chr5.104569709.A.C	independent_variable	0,0001671	0,000505444	0,330600552	0,741078375
ADV4	chr5.104570372.C.T	independent_variable	0,0001671	0,000505444	0,330600552	0,741078375
ADV4	chr5.104571905.G.A	independent_variable	0,0001671	0,000505444	0,330600552	0,741078375
ADV4	chr5.104568136.A.G	independent_variable	0,00016709	0,000505444	0,33058092	0,741093198
ADV4	chr1.43621160.C.T	independent_variable	0,000176936	0,000537061	0,329452677	0,741940599
ADV5	chr7.114435085.T.C	independent_variable	-0,00157491	0,004794553	-0,328479068	0,742681448
ADV2	chr5.104576413.A.C	independent_variable	-0,00032885	0,001007126	-0,326523245	0,744158139
ADV2	chr5.104563989.T.C	independent_variable	-0,00033144	0,001015118	-0,326503711	0,744173649
ADV2	chr5.104569324.T.A	independent_variable	-0,00033144	0,001015118	-0,326503711	0,744173649
ADV2	chr5.104569574.C.T	independent_variable	-0,00033144	0,001015118	-0,326503711	0,744173649
ADV2	chr5.104569709.A.C	independent_variable	-0,00033144	0,001015118	-0,326503711	0,744173649
ADV2	chr5.104570372.C.T	independent_variable	-0,00033144	0,001015118	-0,326503711	0,744173649
ADV2	chr5.104571905.G.A	independent_variable	-0,00033144	0,001015118	-0,326503711	0,744173649
ADV2	chr5.104568136.A.G	independent_variable	-0,00033143	0,001015118	-0,326494054	0,74418095
ADV4	chr1.43872903.A.C	independent_variable	0,000170938	0,000525016	0,325587092	0,744876621
ADV2	chr1.43658317.C.T	independent_variable	-0,00034036	0,001045581	-0,325522592	0,744910983
ADV4	chr5.88861735.T.C	independent_variable	0,000157957	0,000486821	0,324466023	0,74571089
ADV2	chr1.43548609.T.C	independent_variable	-0,000375415	0,001158608	-0,324022718	0,746074105
ADV5	chr11.28620773.G.A	independent_variable	-0,001528491	0,004738613	-0,322560865	0,747157485
ADV2	chr1.43592796.C.T	independent_variable	-0,000333515	0,00103997	-0,320696428	0,74856452
ADV2	chr1.43593571.G.A	independent_variable	-0,000333515	0,00103997	-0,320696428	0,74856452
ADV2	chr1.43594812.G.A	independent_variable	-0,000333515	0,00103997	-0,320696428	0,74856452
ADV2	chr1.43632835.G.A	independent_variable	-0,000335115	0,001050153	-0,319110524	0,74976565
ADV2	chr1.43560369.A.G	independent_variable	-0,000334676	0,001056557	-0,316760909	0,751549054
ADV4	chr4.111546456.G.A	independent_variable	0,000201383	0,000636873	0,316205414	0,751987577
ADV4	chr1.43604361.A.G	independent_variable	0,000168114	0,000532249	0,315856276	0,752247197
ADV4	chr1.43729733.C.T	independent_variable	0,000173365	0,000549348	0,315582642	0,752441694
ADV2	chr5.88497027.G.A	independent_variable	-0,000292996	0,000930827	-0,314769777	0,753071553
ADV4	chr5.104604069.C.T	independent_variable	0,000157845	0,000503111	0,313737353	0,753844672
ADV4	chr7.114552490.T.C	independent_variable	0,000171538	0,000547681	0,313207279	0,754252247
ADV2	chr16.61746654.A.G	independent_variable	-0,000311657	0,000999949	-0,311673066	0,755410191
ADV2	chr16.61754668.C.T	independent_variable	-0,000311657	0,000999949	-0,311673066	0,755410191
ADV2	chr5.104610781.C.T	independent_variable	-0,000361732	0,00116209	-0,311277529	0,755720696
ADV5	chr1.43556632.A.C	independent_variable	0,001613884	0,005194707	0,310678538	0,756166014
ADV2	chr1.43656802.T.G	independent_variable	-0,000323055	0,001051857	-0,307127731	0,758864117
ADV2	chr5.104623644.A.G	independent_variable	-0,000355443	0,001168361	-0,304223762	0,761086312
ADV2	chr5.104571636.G.A	independent_variable	-0,000311348	0,001025184	-0,303699403	0,761477694
ADV4	chr1.43900578.G.A	independent_variable	-0,000180556	0,000595731	-0,303082309	0,761950482
ADV4	chr20.21201749.T.C	independent_variable	0,000172057	0,000571601	0,301008971	0,763530092
ADV2	chr1.43830767.C.G	independent_variable	-0,000295151	0,000983707	-0,300039247	0,764280157
ADV5	chr20.21144231.T.C	independent_variable	-0,001516892	0,005089694	-0,298031977	0,765796218
ADV5	chr11.28619515.G.A	independent_variable	-0,001404264	0,004736097	-0,296502338	0,766964867
ADV5	chr11.28619698.G.A	independent_variable	-0,001404264	0,004736097	-0,296502338	0,766964867
ADV5	chr11.28620721.G.A	independent_variable	-0,001404264	0,004736097	-0,296502338	0,766964867
ADV2	chr1.43584276.C.T	independent_variable	-0,000308574	0,001042761	-0,29591978	0,767405317
ADV2	chr1.43585903.A.G	independent_variable	-0,000308574	0,001042761	-0,29591978	0,767405317
ADV2	chr1.43586163.A.C	independent_variable	-0,000308574	0,001042761	-0,29591978	0,767405317
ADV5	chr11.28639315.G.T	independent_variable	-0,00136614	0,004620785	-0,295651053	0,767611508
ADV5	chr20.21155077.G.T	independent_variable	-0,00148208	0,005044193	-0,293818957	0,769011928
ADV4	chr7.114647061.T.A	independent_variable	-0,000155111	0,000528234	-0,293640478	0,769152804
ADV2	chr1.43612713.T.C	independent_variable	0,000307139	0,001051418	0,29211938	0,770307337
ADV2	chr1.43613740.T.C	independent_variable	0,000307139	0,001051418	0,29211938	0,770307337
ADV2	chr5.88461101.C.T	independent_variable	0,000319441	0,00109946	0,29054391	0,771511298
ADV2	chr5.104576507.C.A	independent_variable	-0,000289472	0,001003027	-0,288598583	0,773001629
ADV4	chr20.21456154.A.G	independent_variable	-0,000153752	0,000534608	-0,287597081	0,773766964
ADV4	chr20.21456675.T.C	independent_variable	-0,000153752	0,000534608	-0,287597081	0,773766964
ADV3	chr11.28504006.A.G	independent_variable	-0,005178542	0,018043853	-0,286997574	0,774240659
ADV5	chr1.43621825.C.T	independent_variable	-0,001435334	0,005005998	-0,286722806	0,774435578
ADV5	chr1.43605020.A.G	independent_variable	-0,001370305	0,004793582	-0,285862495	0,775093471

ADV4	chr5.88455935.G.A	independent_variable	-0,000162264	0,000569598	-0,284874313	0,775854734
ADV4	chr5.88458988.C.T	independent_variable	-0,000162264	0,000569598	-0,284874313	0,775854734
ADV4	chr5.88467651.C.A	independent_variable	-0,000162264	0,000569598	-0,284874313	0,775854734
ADV4	chr5.88473876.C.T	independent_variable	-0,000162264	0,000569598	-0,284874313	0,775854734
ADV4	chr5.88477912.G.T	independent_variable	-0,000162264	0,000569598	-0,284874313	0,775854734
ADV5	chr1.43783814.A.G	independent_variable	-0,001461617	0,005145472	-0,284058922	0,776476797
ADV2	chr1.43621160.C.T	independent_variable	0,000305545	0,001081428	0,282538624	0,777638199
ADV5	chr11.28621106.G.A	independent_variable	-0,001360567	0,00482601	-0,281923877	0,778116694
ADV2	chr5.104596006.G.A	independent_variable	-0,000324147	0,001155417	-0,280545259	0,779175714
ADV4	chr5.88865600.T.C	independent_variable	0,000140272	0,000502051	0,279398476	0,780050777
ADV2	chr11.28655215.G.C	independent_variable	-0,000283994	0,001017963	-0,278982153	0,780366126
ADV4	chr20.21457201.T.G	independent_variable	-0,00014911	0,000535282	-0,278563165	0,780688325
ADV4	chr20.21463034.C.G	independent_variable	-0,00014911	0,000535282	-0,278563165	0,780688325
ADV4	chr20.21465660.G.T	independent_variable	-0,00014911	0,000535282	-0,278563165	0,780688325
ADV2	chr5.104608237.C.G	independent_variable	-0,000323271	0,001161221	-0,278388338	0,780830757
ADV4	chr1.43728863.A.G	independent_variable	0,000152959	0,000549921	0,27814648	0,781006582
ADV4	chr1.43729682.T.C	independent_variable	0,000152959	0,000549921	0,27814648	0,781006582
ADV4	chr1.43843195.A.G	independent_variable	-0,000140107	0,00050668	-0,276519259	0,782257889
ADV5	chr11.28620834.C.T	independent_variable	-0,001300075	0,004705789	-0,27627152	0,782449296
ADV5	chr11.28608504.A.G	independent_variable	-0,001285728	0,004690902	-0,27408982	0,784124441
ADV5	chr1.43577721.G.A	independent_variable	0,001309839	0,00480966	0,272335136	0,785469414
ADV2	chr1.43641757.C.T	independent_variable	0,000295337	0,001087442	0,271589152	0,786041738
ADV1	chr7.114531090.A.G	independent_variable	0,004655928	0,017158202	0,271352896	0,786229211
ADV5	chr11.28646269.A.G	independent_variable	0,00127313	0,004692917	0,271287654	0,786276032
ADV2	chr1.43549278.T.C	independent_variable	-0,000314045	0,001158137	-0,271164116	0,78639169
ADV4	chr5.104482711.A.G	independent_variable	-0,000131217	0,00048542	-0,270316653	0,787019553
ADV4	chr1.43737320.A.G	independent_variable	0,000148948	0,000551081	0,270283576	0,787045942
ADV2	chr11.28655291.A.G	independent_variable	-0,000273597	0,001013767	-0,269882084	0,787353504
ADV2	chr1.43840448.A.G	independent_variable	0,000293246	0,00109128	0,268717373	0,788252343
ADV2	chr1.43845072.C.T	independent_variable	0,000293246	0,00109128	0,268717373	0,788252343
ADV2	chr1.43845203.G.A	independent_variable	0,000293246	0,00109128	0,268717373	0,788252343
ADV1	chr3.20680326.A.G	independent_variable	-0,004200041	0,015749868	-0,266671521	0,789824835
ADV1	chr3.20680427.C.T	independent_variable	-0,004200041	0,015749868	-0,266671521	0,789824835
ADV1	chr3.20683524.C.A	independent_variable	-0,004200041	0,015749868	-0,266671521	0,789824835
ADV4	chr20.21155077.G.T	independent_variable	0,000148538	0,000557063	0,266644489	0,789847198
ADV4	chr20.21146082.A.G	independent_variable	0,000148538	0,000557063	0,266644489	0,789847198
ADV5	chr1.43599477.C.T	independent_variable	-0,001279218	0,004803944	-0,266284955	0,790121403
ADV5	chr5.104642625.G.A	independent_variable	-0,001402417	0,00529936	-0,264638953	0,791397764
ADV5	chr11.28609199.A.G	independent_variable	-0,001235912	0,004698516	-0,263043108	0,792621743
ADV5	chr11.28622320.A.G	independent_variable	-0,001224276	0,004656373	-0,262924863	0,792711055
ADV5	chr11.28624925.C.T	independent_variable	-0,001224276	0,004656373	-0,262924863	0,792711055
ADV5	chr1.43596488.C.T	independent_variable	-0,001265536	0,004816715	-0,262738456	0,792852383
ADV1	chr5.88397820.G.C	independent_variable	-0,004698479	0,017889385	-0,262640625	0,792932007
ADV4	chr1.43728545.T.G	independent_variable	0,000144427	0,000550434	0,262387284	0,793123314
ADV2	chr5.104610505.A.T	independent_variable	-0,000300013	0,001161139	-0,258377936	0,796222856
ADV5	chr1.43655886.C.G	independent_variable	-0,001245031	0,004841331	-0,257167076	0,797147218
ADV5	chr1.43537286.G.A	independent_variable	-0,001496222	0,005826967	-0,25677539	0,797464773
ADV2	chr1.43563242.G.A	independent_variable	-0,000269785	0,001051439	-0,256586457	0,797596669
ADV5	chr1.43554060.C.T	independent_variable	-0,00125663	0,004897632	-0,256579169	0,79760398
ADV5	chr1.43556356.A.G	independent_variable	-0,00125663	0,004897632	-0,256579169	0,79760398
ADV4	chr20.21249817.C.T	independent_variable	0,000142196	0,00055421	0,256574642	0,797613113
ADV5	chr20.21249817.C.T	independent_variable	-0,001321504	0,005169849	-0,255617587	0,798351679
ADV5	chr1.43592796.C.T	independent_variable	-0,001223141	0,004823608	-0,253573796	0,799921356
ADV5	chr1.43593571.G.A	independent_variable	-0,001223141	0,004823608	-0,253573796	0,799921356
ADV5	chr1.43594812.G.A	independent_variable	-0,001223141	0,004823608	-0,253573796	0,799921356
ADV2	chr5.104582527.C.T	independent_variable	-0,000251092	0,000994407	-0,252504216	0,800748518
ADV5	chr1.43570414.G.A	independent_variable	0,001220672	0,004834601	0,252486664	0,800762258
ADV2	chr5.104597772.C.T	independent_variable	-0,000289438	0,001151603	-0,251334442	0,801659016
ADV5	chr11.28645542.T.C	independent_variable	0,001175235	0,004699217	0,25009162	0,802614141
ADV4	chr1.43778195.T.C	independent_variable	0,000138975	0,000556806	0,249593931	0,802997734
ADV4	chr1.43781562.C.G	independent_variable	0,000138975	0,000556806	0,249593931	0,802997734
ADV5	chr11.28609187.T.G	independent_variable	-0,001165509	0,004688545	-0,248586548	0,80377876
ADV2	chr1.43543362.C.T	independent_variable	-0,000279023	0,001132194	-0,24644463	0,805451957
ADV1	chr3.20682712.G.A	independent_variable	-0,003877285	0,015735769	-0,246399451	0,80546754
ADV5	chr11.28628323.A.G	independent_variable	0,00115261	0,004687452	0,245892571	0,805861312
ADV5	chr1.43586107.G.A	independent_variable	-0,001182747	0,004829891	-0,244880678	0,806642022
ADV2	chr11.28521551.G.A	independent_variable	-0,000258191	0,001056538	-0,24437499	0,807040858
ADV2	chr11.28526196.C.T	independent_variable	-0,000258191	0,001056538	-0,24437499	0,807040858
ADV2	chr11.28526682.A.C	independent_variable	-0,000258191	0,001056538	-0,24437499	0,807040858
ADV5	chr1.43560382.T.G	independent_variable	0,001253506	0,005141426	0,243805095	0,807475411
ADV5	chr1.43560509.A.C	independent_variable	0,001253506	0,005141426	0,243805095	0,807475411

ADV2	chr7.114571392.A.G	independent_variable	-0,000236659	0,000974955	-0,242738605	0,80829956
ADV2	chr5.104563895.T.C	independent_variable	-0,000249244	0,001028612	-0,242310925	0,808634238
ADV3	chr7.114407085.A.T	independent_variable	-0,0043409	0,01796689	-0,241605537	0,809181259
ADV4	chr1.43616091.T.G	independent_variable	0,00012276	0,000510148	0,240635778	0,809931178
ADV4	chr5.88867954.G.A	independent_variable	0,000120312	0,000502703	0,239329769	0,810944916
ADV5	chr11.28609522.G.C	independent_variable	-0,001112811	0,004678463	-0,237858283	0,812084524
ADV5	chr11.28609701.C.A	independent_variable	-0,001112811	0,004678463	-0,237858283	0,812084524
ADV2	chr5.104609477.C.T	independent_variable	-0,000275239	0,00115885	-0,237510757	0,812359046
ADV2	chr5.88558577.A.C	independent_variable	-0,000283257	0,001194512	-0,237132322	0,81265659
ADV2	chr5.104581651.A.C	independent_variable	-0,000236073	0,000995901	-0,237044676	0,81271321
ADV2	chr5.104582166.G.T	independent_variable	-0,000236073	0,000995901	-0,237044676	0,81271321
ADV2	chr5.104582921.C.T	independent_variable	-0,000236073	0,000995901	-0,237044676	0,81271321
ADV2	chr5.104582998.G.A	independent_variable	-0,000236073	0,000995901	-0,237044676	0,81271321
ADV2	chr5.104583048.G.T	independent_variable	-0,000236073	0,000995901	-0,237044676	0,81271321
ADV2	chr5.104584121.A.C	independent_variable	-0,000236073	0,000995901	-0,237044676	0,81271321
ADV2	chr5.104642625.G.A	independent_variable	-0,000274141	0,00115696	-0,236948952	0,81279442
ADV2	chr7.114554560.T.G	independent_variable	0,000225072	0,000952932	0,236188893	0,813382433
ADV5	chr1.43584276.C.T	independent_variable	-0,001139736	0,004836878	-0,235634623	0,813805708
ADV5	chr1.43585903.A.G	independent_variable	-0,001139736	0,004836878	-0,235634623	0,813805708
ADV5	chr1.43586163.A.C	independent_variable	-0,001139736	0,004836878	-0,235634623	0,813805708
ADV2	chr7.114570759.T.C	independent_variable	-0,000215465	0,000919058	-0,234441539	0,81473344
ADV5	chr1.43560418.C.T	independent_variable	0,001196574	0,005125586	0,233451221	0,815500673
ADV5	chr20.21157726.C.A	independent_variable	-0,001174164	0,005083406	-0,230979821	0,817419954
ADV5	chr1.43658317.C.T	independent_variable	-0,001116691	0,00484893	-0,23029642	0,817948176
ADV5	chr11.28554714.G.T	independent_variable	-0,001153793	0,005009755	-0,230309269	0,817951242
ADV5	chr11.28628549.C.T	independent_variable	0,001078496	0,004697708	0,229579107	0,818508258
ADV2	chr5.88567792.C.T	independent_variable	0,0002698	0,001176122	0,229398358	0,818653685
ADV2	chr5.88574430.T.C	independent_variable	0,0002698	0,001176122	0,229398358	0,818653685
ADV2	chr5.88577805.C.T	independent_variable	0,0002698	0,001176122	0,229398358	0,818653685
ADV5	chr11.28504006.A.G	independent_variable	-0,001147769	0,005034145	-0,227996735	0,819747933
ADV5	chr11.28522265.G.A	independent_variable	-0,001141157	0,005013002	-0,227639379	0,820025176
ADV4	chr1.43777612.C.T	independent_variable	0,00012717	0,000558734	0,227603883	0,820041809
ADV4	chr1.43744582.C.G	independent_variable	0,00012717	0,000558734	0,227603883	0,820041809
ADV4	chr1.43745339.A.G	independent_variable	0,00012717	0,000558734	0,227603883	0,820041809
ADV4	chr1.43746026.T.C	independent_variable	0,00012717	0,000558734	0,227603883	0,820041809
ADV4	chr1.43747271.A.G	independent_variable	0,00012717	0,000558734	0,227603883	0,820041809
ADV4	chr1.43747507.C.A	independent_variable	0,00012717	0,000558734	0,227603883	0,820041809
ADV4	chr1.43751205.T.C	independent_variable	0,00012717	0,000558734	0,227603883	0,820041809
ADV4	chr1.43753604.T.C	independent_variable	0,00012717	0,000558734	0,227603883	0,820041809
ADV4	chr1.43763926.G.A	independent_variable	0,00012717	0,000558734	0,227603883	0,820041809
ADV2	chr1.43577434.T.C	independent_variable	-0,000250073	0,001099336	-0,227476813	0,820140062
ADV5	chr11.28609937.C.T	independent_variable	-0,001062656	0,004686082	-0,226768592	0,820692805
ADV5	chr11.28648570.G.T	independent_variable	0,001068734	0,004718252	0,226510516	0,820892545
ADV5	chr11.28648575.T.A	independent_variable	0,001068734	0,004718252	0,226510516	0,820892545
ADV2	chr5.104611504.C.T	independent_variable	-0,000261871	0,001160315	-0,225689331	0,821536586
ADV2	chr5.104612344.C.T	independent_variable	-0,000261871	0,001160315	-0,225689331	0,821536586
ADV3	chr5.88456878.A.G	independent_variable	0,004422901	0,019637281	0,225229794	0,821889339
ADV3	chr5.88461004.C.T	independent_variable	0,004422901	0,019637281	0,225229794	0,821889339
ADV3	chr5.88467699.G.A	independent_variable	0,004422901	0,019637281	0,225229794	0,821889339
ADV3	chr5.88469954.T.C	independent_variable	0,004422901	0,019637281	0,225229794	0,821889339
ADV3	chr5.88475276.A.C	independent_variable	0,004422901	0,019637281	0,225229794	0,821889339
ADV3	chr5.88477838.A.G	independent_variable	0,004422901	0,019637281	0,225229794	0,821889339
ADV5	chr11.28671984.T.G	independent_variable	0,001064783	0,00473503	0,224873571	0,822165799
ADV5	chr11.28672893.C.G	independent_variable	0,001064783	0,00473503	0,224873571	0,822165799
ADV5	chr11.28654592.G.A	independent_variable	0,001055058	0,004702141	0,224378259	0,822550117
ADV1	chr3.20678505.C.T	independent_variable	-0,003526844	0,015750832	-0,22391475	0,822909118
ADV5	chr11.28669740.C.T	independent_variable	0,001057323	0,004733471	0,223371562	0,823333554
ADV5	chr1.43632835.G.A	independent_variable	-0,001081571	0,004870485	-0,22206632	0,824345904
ADV2	chr5.104584139.G.A	independent_variable	-0,000220955	0,000997402	-0,221530252	0,824764589
ADV2	chr11.28526861.T.C	independent_variable	-0,000233871	0,001057786	-0,221094458	0,825109325
ADV2	chr1.43572014.G.A	independent_variable	-0,000221111	0,001001388	-0,220804677	0,825329236
ADV4	chr20.21175938.T.C	independent_variable	-0,000118641	0,00053955	-0,219889505	0,826044173
ADV4	chr20.21179524.A.C	independent_variable	-0,000118641	0,00053955	-0,219889505	0,826044173
ADV2	chr1.43541977.T.C	independent_variable	-0,000252162	0,001156359	-0,218065947	0,827478826
ADV4	chr1.43546066.G.A	independent_variable	-0,000116128	0,000536699	-0,216374912	0,828794673
ADV4	chr7.114544043.G.A	independent_variable	-0,00010896	0,000504496	-0,215977317	0,829092983
ADV2	chr5.88600784.C.T	independent_variable	-0,000246374	0,001143139	-0,215524023	0,829447559
ADV5	chr11.28605239.G.A	independent_variable	-0,001006888	0,004672097	-0,215510873	0,829453215
ADV5	chr11.28606106.G.A	independent_variable	-0,001006888	0,004672097	-0,215510873	0,829453215
ADV5	chr11.28607002.C.A	independent_variable	-0,001006888	0,004672097	-0,215510873	0,829453215
ADV5	chr11.28607568.G.A	independent_variable	-0,001006888	0,004672097	-0,215510873	0,829453215

ADV5	chr11.28613204.C.T	independent_variable	-0,001006888	0,004672097	-0,215510873	0,829453215
ADV5	chr11.28613315.A.G	independent_variable	-0,001006888	0,004672097	-0,215510873	0,829453215
ADV5	chr11.28613316.T.C	independent_variable	-0,001006888	0,004672097	-0,215510873	0,829453215
ADV5	chr11.28614382.G.A	independent_variable	-0,001006888	0,004672097	-0,215510873	0,829453215
ADV5	chr11.28615789.A.G	independent_variable	-0,001006888	0,004672097	-0,215510873	0,829453215
ADV5	chr11.28666860.G.A	independent_variable	0,001017752	0,00472597	0,215352988	0,829575914
ADV5	chr11.28632994.A.G	independent_variable	0,0009987	0,004668816	0,213908544	0,830700488
ADV5	chr11.28639271.A.G	independent_variable	0,0009987	0,004668816	0,213908544	0,830700488
ADV5	chr1.43652735.G.A	independent_variable	-0,001039087	0,004864379	-0,213611431	0,830930265
ADV5	chr11.28624037.T.C	independent_variable	0,000997166	0,004682727	0,21294562	0,831451725
ADV5	chr11.28628989.A.G	independent_variable	0,000997166	0,004682727	0,21294562	0,831451725
ADV5	chr11.28629363.A.G	independent_variable	0,000997166	0,004682727	0,21294562	0,831451725
ADV4	chr5.104556759.C.G	independent_variable	0,000109389	0,000518275	0,211064372	0,832920208
ADV2	chr1.43560985.G.C	independent_variable	0,000234304	0,001114825	0,210170902	0,833614605
ADV4	chr20.21412561.A.G	independent_variable	-0,00010943	0,000522005	-0,209634002	0,834031822
ADV5	chr1.43560369.A.G	independent_variable	-0,001018113	0,004867284	-0,209174698	0,834391784
ADV2	chr5.104569213.T.C	independent_variable	-0,000210314	0,001014949	-0,207216524	0,835921111
ADV1	chr7.114456791.A.T	independent_variable	0,003473042	0,0168417	0,206216837	0,83669955
ADV2	chr5.104577563.A.C	independent_variable	-0,000205756	0,00099891	-0,205980374	0,836885189
ADV2	chr5.104577567.C.T	independent_variable	-0,000205756	0,00099891	-0,205980374	0,836885189
ADV2	chr5.104577620.T.A	independent_variable	-0,000205756	0,00099891	-0,205980374	0,836885189
ADV2	chr5.104578055.A.T	independent_variable	-0,000205756	0,00099891	-0,205980374	0,836885189
ADV2	chr5.104578313.C.T	independent_variable	-0,000205756	0,00099891	-0,205980374	0,836885189
ADV2	chr5.104578842.G.A	independent_variable	-0,000205756	0,00099891	-0,205980374	0,836885189
ADV2	chr5.104579057.A.G	independent_variable	-0,000205756	0,00099891	-0,205980374	0,836885189
ADV2	chr5.104579085.A.T	independent_variable	-0,000205756	0,00099891	-0,205980374	0,836885189
ADV2	chr5.104579135.T.G	independent_variable	-0,000205756	0,00099891	-0,205980374	0,836885189
ADV2	chr5.104579581.A.G	independent_variable	-0,000205756	0,00099891	-0,205980374	0,836885189
ADV2	chr5.104579742.G.A	independent_variable	-0,000205756	0,00099891	-0,205980374	0,836885189
ADV2	chr5.104580027.A.G	independent_variable	-0,000205756	0,00099891	-0,205980374	0,836885189
ADV2	chr5.104580648.T.C	independent_variable	-0,000205756	0,00099891	-0,205980374	0,836885189
ADV2	chr5.104580662.G.C	independent_variable	-0,000205756	0,00099891	-0,205980374	0,836885189
ADV2	chr5.104581130.A.G	independent_variable	-0,000205756	0,00099891	-0,205980374	0,836885189
ADV4	chr20.21428075.A.G	independent_variable	-0,000108437	0,000528333	-0,205243146	0,837459937
ADV4	chr20.21436041.T.G	independent_variable	-0,000108437	0,000528333	-0,205243146	0,837459937
ADV4	chr20.21439854.C.T	independent_variable	-0,000108437	0,000528333	-0,205243146	0,837459937
ADV4	chr20.21440237.T.C	independent_variable	-0,000108437	0,000528333	-0,205243146	0,837459937
ADV4	chr20.21444512.T.A	independent_variable	-0,000108437	0,000528333	-0,205243146	0,837459937
ADV4	chr20.21444955.G.C	independent_variable	-0,000108437	0,000528333	-0,205243146	0,837459937
ADV4	chr20.21449932.G.A	independent_variable	-0,000108437	0,000528333	-0,205243146	0,837459937
ADV2	chr1.43689712.A.G	independent_variable	0,000226594	0,001104767	0,205105957	0,83756649
ADV2	chr5.104577501.A.G	independent_variable	-0,000204656	0,001002513	-0,204142709	0,838320492
ADV5	chr1.43656802.T.G	independent_variable	-0,000988018	0,004878645	-0,202518847	0,839587123
ADV5	chr11.28511111.T.C	independent_variable	-0,001016338	0,005037586	-0,201751005	0,840198676
ADV5	chr20.21156917.A.G	independent_variable	-0,001022068	0,005071915	-0,201515202	0,840373354
ADV2	chr5.88579561.C.T	independent_variable	0,000235876	0,001180431	0,199821527	0,841702061
ADV2	chr5.88584754.A.G	independent_variable	0,000235876	0,001180431	0,199821527	0,841702061
ADV2	chr5.88586361.A.G	independent_variable	0,000235876	0,001180431	0,199821527	0,841702061
ADV4	chr5.104568525.A.G	independent_variable	-0,000105337	0,000528153	-0,1994444	0,841997033
ADV2	chr5.104591878.C.T	independent_variable	-0,000228822	0,001150693	-0,198856234	0,842456364
ADV4	chr5.88461101.C.T	independent_variable	-0,000107487	0,000546806	-0,196572161	0,844236042
ADV5	chr11.28615816.T.C	independent_variable	-0,000905857	0,004687286	-0,193258283	0,846832157
ADV2	chr5.88587685.T.C	independent_variable	-0,000219324	0,001138131	-0,192705544	0,847267828
ADV4	chr1.43830767.C.G	independent_variable	0,000102482	0,00053217	0,192574272	0,847375753
ADV5	chr20.21177599.C.G	independent_variable	-0,000981954	0,00511413	-0,192007993	0,847811628
ADV5	chr20.21177731.T.G	independent_variable	-0,000981954	0,00511413	-0,192007993	0,847811628
ADV5	chr20.21178512.T.C	independent_variable	-0,000981954	0,00511413	-0,192007993	0,847811628
ADV1	chr5.88386213.G.A	independent_variable	-0,003452038	0,017982705	-0,191964365	0,847845202
ADV5	chr11.28604117.C.T	independent_variable	-0,000887293	0,004669825	-0,190005559	0,849378638
ADV2	chr5.104590343.C.T	independent_variable	-0,000220936	0,001166641	-0,189378034	0,849873543
ADV2	chr1.43549064.C.T	independent_variable	-0,000214928	0,001135077	-0,189350961	0,849903895
ADV5	chr1.43614741.T.C	independent_variable	0,000966765	0,005106925	0,189304683	0,849925155
ADV2	chr5.88569970.A.G	independent_variable	0,000223073	0,001179017	0,189202779	0,85001113
ADV2	chr5.104585069.C.A	independent_variable	-0,000221046	0,001171071	-0,188755289	0,850361822
ADV2	chr1.43726088.C.T	independent_variable	0,000206404	0,001102499	0,187214471	0,851562929
ADV2	chr1.43731274.C.T	independent_variable	0,000206404	0,001102499	0,187214471	0,851562929
ADV1	chr3.20683444.C.T	independent_variable	-0,002936709	0,015733309	-0,186655506	0,852001369
ADV4	chr7.114508188.A.G	independent_variable	9,0288E-05	0,000484239	0,186453245	0,852163775
ADV2	chr1.43685344.T.G	independent_variable	0,000202702	0,00110097	0,184112184	0,853994057
ADV5	chr20.21167328.G.C	independent_variable	-0,000967954	0,00529071	-0,18295347	0,854905292
ADV5	chr20.21170255.A.G	independent_variable	-0,000967954	0,00529071	-0,18295347	0,854905292

ADV5	chr20.21170684.G.A	independent_variable	-0,000967954	0,00529071	-0,18295347	0,854905292
ADV5	chr20.21170688.A.G	independent_variable	-0,000967954	0,00529071	-0,18295347	0,854905292
ADV5	chr20.21170708.C.T	independent_variable	-0,000967954	0,00529071	-0,18295347	0,854905292
ADV5	chr20.21171212.C.T	independent_variable	-0,000967954	0,00529071	-0,18295347	0,854905292
ADV5	chr20.21171591.G.A	independent_variable	-0,000967954	0,00529071	-0,18295347	0,854905292
ADV5	chr20.21172102.G.A	independent_variable	-0,000967954	0,00529071	-0,18295347	0,854905292
ADV5	chr20.21172498.G.A	independent_variable	-0,000967954	0,00529071	-0,18295347	0,854905292
ADV5	chr20.21173053.G.A	independent_variable	-0,000967954	0,00529071	-0,18295347	0,854905292
ADV5	chr20.21173632.G.C	independent_variable	-0,000967954	0,00529071	-0,18295347	0,854905292
ADV5	chr20.21173847.G.A	independent_variable	-0,000967954	0,00529071	-0,18295347	0,854905292
ADV4	chr20.21144231.T.C	independent_variable	0,000102196	0,000562073	0,181820446	0,855793551
ADV4	chr7.114605694.T.A	independent_variable	8,98146E-05	0,000494064	0,181787501	0,855822727
ADV1	chr5.88445076.T.C	independent_variable	0,003340521	0,018393327	0,181615916	0,855955616
ADV1	chr5.88468801.G.A	independent_variable	0,003340521	0,018393327	0,181615916	0,855955616
ADV2	chr5.88588020.G.C	independent_variable	-0,000206653	0,001139559	-0,181344355	0,856170927
ADV2	chr5.104602414.C.T	independent_variable	-0,000178521	0,000999029	-0,178693991	0,858246273
ADV4	chr5.104550512.T.G	independent_variable	9,16593E-05	0,000518183	0,176885887	0,859667297
ADV5	chr20.21175529.A.G	independent_variable	-0,000896977	0,005091511	-0,176171083	0,860228522
ADV5	chr20.21176291.G.C	independent_variable	-0,000896977	0,005091511	-0,176171083	0,860228522
ADV5	chr20.21176925.T.C	independent_variable	-0,000896977	0,005091511	-0,176171083	0,860228522
ADV5	chr20.21180143.G.A	independent_variable	-0,000896977	0,005091511	-0,176171083	0,860228522
ADV5	chr20.21183159.A.G	independent_variable	-0,000896977	0,005091511	-0,176171083	0,860228522
ADV5	chr20.21227106.T.G	independent_variable	-0,000843473	0,004792012	-0,176016437	0,860346652
ADV5	chr20.21166199.T.C	independent_variable	-0,000928202	0,005297118	-0,175227659	0,860968569
ADV5	chr20.21169627.G.C	independent_variable	-0,000928202	0,005297118	-0,175227659	0,860968569
ADV1	chr7.114483253.C.T	independent_variable	0,002892185	0,016739201	0,172779146	0,862889769
ADV4	chr1.43572014.G.A	independent_variable	-9,00204E-05	0,000526187	-0,171080714	0,864225349
ADV2	chr5.104590741.T.A	independent_variable	-0,000195032	0,001146945	-0,170045188	0,865043517
ADV2	chr5.104600642.T.A	independent_variable	-0,000195032	0,001146945	-0,170045188	0,865043517
ADV4	chr20.21156917.A.G	independent_variable	9,4458E-05	0,000560128	0,168636467	0,866147175
ADV5	chr11.28603810.A.T	independent_variable	-0,000785574	0,004685057	-0,167676428	0,866903031
ADV4	chr5.104557287.A.T	independent_variable	8,57945E-05	0,000517415	0,165813555	0,868368373
ADV1	chr5.88417014.A.G	independent_variable	0,002976908	0,018029184	0,165116056	0,868917176
ADV2	chr5.104619660.T.C	independent_variable	-0,000191309	0,001165502	-0,164143304	0,869685879
ADV4	chr1.43831901.A.C	independent_variable	8,77207E-05	0,000544984	0,160960175	0,872187041
ADV2	chr5.88563112.T.G	independent_variable	-0,000183938	0,001148538	-0,160149561	0,87282846
ADV2	chr1.43729733.C.T	independent_variable	0,000175542	0,001106138	0,158697653	0,873966639
ADV4	chr7.114522685.G.A	independent_variable	7,66459E-05	0,00048409	0,158329706	0,87425992
ADV5	chr5.88862329.G.C	independent_variable	0,00074602	0,004721471	0,158005754	0,874513019
ADV2	chr1.43658195.A.G	independent_variable	0,00016417	0,001057781	0,155202584	0,876720812
ADV5	chr20.21165647.A.G	independent_variable	-0,00080942	0,005278345	-0,153347359	0,87818339
ADV5	chr20.21165828.A.T	independent_variable	-0,00080942	0,005278345	-0,153347359	0,87818339
ADV5	chr20.21166369.A.G	independent_variable	-0,00080942	0,005278345	-0,153347359	0,87818339
ADV5	chr20.21166767.A.G	independent_variable	-0,00080942	0,005278345	-0,153347359	0,87818339
ADV5	chr20.21168043.G.T	independent_variable	-0,00080942	0,005278345	-0,153347359	0,87818339
ADV1	chr5.88385177.A.G	independent_variable	-0,002742344	0,018044695	-0,1519751	0,879265747
ADV1	chr5.88389683.C.T	independent_variable	-0,002742344	0,018044695	-0,1519751	0,879265747
ADV2	chr5.104614550.C.T	independent_variable	-0,00017591	0,001163062	-0,151247197	0,879842714
ADV2	chr1.43610348.G.A	independent_variable	0,000157772	0,001049446	0,150338854	0,880553005
ADV4	chr5.88551455.T.A	independent_variable	7,93175E-05	0,000527797	0,150280341	0,880606835
ADV5	chr4.111251830.C.A	independent_variable	0,001172162	0,007801495	0,150248419	0,880626443
ADV5	chr1.43550344.G.A	independent_variable	-0,000789611	0,005271998	-0,149774552	0,881002522
ADV2	chr6.70148037.A.G	independent_variable	0,000162135	0,001086725	0,149196112	0,88145629
ADV2	chr6.70148809.A.C	independent_variable	0,000162135	0,001086725	0,149196112	0,88145629
ADV5	chr1.43853335.A.G	independent_variable	-0,000749915	0,005081216	-0,147585735	0,882724903
ADV2	chr1.43737320.A.G	independent_variable	0,00016357	0,001109952	0,147367105	0,88289758
ADV5	chr20.21176320.A.G	independent_variable	-0,000748326	0,005100445	-0,146717872	0,883411904
ADV2	chr6.70142601.A.G	independent_variable	0,000159389	0,001092618	0,145877828	0,884074257
ADV2	chr6.70142949.A.T	independent_variable	0,000159389	0,001092618	0,145877828	0,884074257
ADV2	chr6.70143876.T.C	independent_variable	0,000159389	0,001092618	0,145877828	0,884074257
ADV2	chr1.43778195.T.C	independent_variable	0,000161449	0,0011209	0,144035578	0,885526919
ADV2	chr1.43781562.C.G	independent_variable	0,000161449	0,0011209	0,144035578	0,885526919
ADV4	chr7.114608796.A.G	independent_variable	-7,55775E-05	0,000526944	-0,14342608	0,886008824
ADV2	chr5.104608319.G.T	independent_variable	-0,000166055	0,001160986	-0,143028884	0,886325808
ADV4	chr5.88433894.A.C	independent_variable	-8,0033E-05	0,000559935	-0,142932739	0,886400654
ADV1	chr3.20687599.C.T	independent_variable	-0,002206004	0,015627562	-0,141161133	0,887795002
ADV5	chr20.21187721.G.T	independent_variable	-0,000675589	0,004812439	-0,140383926	0,888411018
ADV5	chr20.21190532.C.T	independent_variable	-0,000675589	0,004812439	-0,140383926	0,888411018
ADV5	chr20.21194910.T.C	independent_variable	-0,000675589	0,004812439	-0,140383926	0,888411018
ADV1	chr5.88455935.G.A	independent_variable	0,002572387	0,018327126	0,14035953	0,888430704
ADV1	chr5.88458988.C.T	independent_variable	0,002572387	0,018327126	0,14035953	0,888430704

ADV1	chr5.88467651.C.A	independent_variable	0,002572387	0,018327126	0,14035953	0,888430704
ADV1	chr5.88473876.C.T	independent_variable	0,002572387	0,018327126	0,14035953	0,888430704
ADV1	chr5.88477912.G.T	independent_variable	0,002572387	0,018327126	0,14035953	0,888430704
ADV4	chr3.20627579.T.G	independent_variable	-6,9348E-05	0,000496659	-0,139628895	0,889006352
ADV5	chr1.43718252.C.T	independent_variable	-0,000694802	0,005000117	-0,138957207	0,889535867
ADV4	chr20.21158348.G.A	independent_variable	7,76842E-05	0,000561409	0,138373768	0,889997784
ADV4	chr20.21158611.G.A	independent_variable	7,76842E-05	0,000561409	0,138373768	0,889997784
ADV4	chr20.21158741.C.G	independent_variable	7,76842E-05	0,000561409	0,138373768	0,889997784
ADV4	chr20.21159530.G.A	independent_variable	7,76842E-05	0,000561409	0,138373768	0,889997784
ADV4	chr20.21159701.C.T	independent_variable	7,76842E-05	0,000561409	0,138373768	0,889997784
ADV4	chr20.21157726.C.A	independent_variable	7,76842E-05	0,000561409	0,138373768	0,889997784
ADV4	chr20.21161712.T.C	independent_variable	7,76842E-05	0,000561409	0,138373768	0,889997784
ADV2	chr5.88851954.G.A	independent_variable	0,000138924	0,001006588	0,138015165	0,890281451
ADV2	chr5.88852224.C.T	independent_variable	0,000138924	0,001006588	0,138015165	0,890281451
ADV2	chr5.88853474.A.G	independent_variable	0,000138924	0,001006588	0,138015165	0,890281451
ADV2	chr5.88853509.G.A	independent_variable	0,000138924	0,001006588	0,138015165	0,890281451
ADV2	chr5.88854472.G.A	independent_variable	0,000138924	0,001006588	0,138015165	0,890281451
ADV2	chr5.88855175.T.A	independent_variable	0,000138924	0,001006588	0,138015165	0,890281451
ADV2	chr5.88856385.T.C	independent_variable	0,000138924	0,001006588	0,138015165	0,890281451
ADV2	chr5.88856757.C.A	independent_variable	0,000138924	0,001006588	0,138015165	0,890281451
ADV2	chr5.88858057.C.T	independent_variable	0,000138924	0,001006588	0,138015165	0,890281451
ADV5	chr7.114540007.A.G	independent_variable	-0,00064128	0,004647806	-0,137974729	0,890315278
ADV4	chr7.114440281.T.G	independent_variable	7,20737E-05	0,000522707	0,137885332	0,890383019
ADV2	chr6.70144291.A.G	independent_variable	0,000149271	0,001085414	0,137524613	0,890668865
ADV2	chr1.43717869.G.T	independent_variable	0,00015203	0,001110985	0,136842189	0,891206538
ADV5	chr20.21187755.C.T	independent_variable	-0,000656028	0,004819306	-0,136125069	0,891775193
ADV1	chr5.88461101.C.T	independent_variable	0,002397635	0,017672424	0,135670998	0,892131748
ADV2	chr5.88885292.T.G	independent_variable	-0,000162629	0,001218795	-0,133434576	0,893903931
ADV4	chr5.104559315.C.T	independent_variable	6,82589E-05	0,000514407	0,132694385	0,894486713
ADV4	chr5.104559414.T.C	independent_variable	6,82589E-05	0,000514407	0,132694385	0,894486713
ADV4	chr5.104560429.T.C	independent_variable	6,82589E-05	0,000514407	0,132694385	0,894486713
ADV4	chr5.104562057.T.C	independent_variable	6,82589E-05	0,000514407	0,132694385	0,894486713
ADV4	chr5.104563477.A.G	independent_variable	6,82589E-05	0,000514407	0,132694385	0,894486713
ADV5	chr1.43585185.T.C	independent_variable	-0,000668717	0,005040957	-0,132656731	0,894514556
ADV4	chr20.21154956.G.T	independent_variable	7,27634E-05	0,000554705	0,131174956	0,895686967
ADV5	chr5.88879434.C.G	independent_variable	-0,000641554	0,00489117	-0,131165681	0,895701218
ADV5	chr20.21228388.C.T	independent_variable	-0,000660505	0,005042462	-0,130988501	0,895834582
ADV5	chr1.43716573.A.G	independent_variable	-0,000653742	0,004994017	-0,130904989	0,895899221
ADV5	chr4.111546456.G.A	independent_variable	-0,0008122	0,006217609	-0,130628932	0,896124985
ADV4	chr7.114520816.G.A	independent_variable	6,3023E-05	0,000483948	0,130226773	0,896438563
ADV4	chr7.114464334.T.C	independent_variable	6,68707E-05	0,000517294	0,129270115	0,897192141
ADV2	chr1.43739423.T.C	independent_variable	0,000143827	0,001114907	0,129003493	0,897403367
ADV4	chr20.21073855.C.T	independent_variable	6,49836E-05	0,000509855	0,127455098	0,89862996
ADV4	chr20.21165647.A.G	independent_variable	7,18743E-05	0,000566689	0,126831952	0,899122059
ADV4	chr20.21165828.A.T	independent_variable	7,18743E-05	0,000566689	0,126831952	0,899122059
ADV4	chr20.21166369.A.G	independent_variable	7,18743E-05	0,000566689	0,126831952	0,899122059
ADV4	chr20.21166767.A.G	independent_variable	7,18743E-05	0,000566689	0,126831952	0,899122059
ADV4	chr20.21168043.G.T	independent_variable	7,18743E-05	0,000566689	0,126831952	0,899122059
ADV4	chr1.43610798.G.A	independent_variable	6,60366E-05	0,000525474	0,12567039	0,900039528
ADV4	chr7.114627941.T.C	independent_variable	6,09847E-05	0,000488632	0,124806969	0,900726936
ADV4	chr1.43634413.G.A	independent_variable	-6,37624E-05	0,000518367	-0,123006359	0,90214766
ADV5	chr1.43686240.C.T	independent_variable	-0,000707742	0,005772372	-0,122608554	0,902463822
ADV5	chr11.28636483.A.G	independent_variable	-0,000556233	0,004567723	-0,121774773	0,903122528
ADV4	chr20.21138467.C.G	independent_variable	6,83286E-05	0,000564691	0,121001755	0,903735765
ADV4	chr20.21140567.A.T	independent_variable	6,83286E-05	0,000564691	0,121001755	0,903735765
ADV4	chr7.114474994.A.G	independent_variable	6,18623E-05	0,000513905	0,120376836	0,904229167
ADV2	chr1.43720773.T.A	independent_variable	0,000133487	0,001111098	0,120139818	0,904417075
ADV5	chr3.20696597.A.G	independent_variable	-0,000603256	0,005022294	-0,120115527	0,904442313
ADV5	chr3.20698841.C.G	independent_variable	-0,000603256	0,005022294	-0,120115527	0,904442313
ADV2	chr5.88862329.G.C	independent_variable	0,000120423	0,001008156	0,119448648	0,904965614
ADV2	chr1.43728545.T.G	independent_variable	0,000132377	0,001108581	0,119411112	0,904994234
ADV3	chr5.88401026.G.A	independent_variable	0,002257359	0,018983125	0,118913985	0,905389425
ADV2	chr6.70148891.T.C	independent_variable	-0,000118307	0,000994984	-0,118903306	0,905397705
ADV2	chr1.43631859.T.C	independent_variable	0,000123382	0,001051487	0,117340873	0,906633486
ADV5	chr7.114544043.G.A	independent_variable	-0,000547414	0,004668973	-0,117245153	0,906712586
ADV4	chr1.43658195.A.G	independent_variable	5,9592E-05	0,000509116	0,117050141	0,906864745
ADV5	chr20.21170737.G.A	independent_variable	-0,000609329	0,005236961	-0,116351566	0,907418582
ADV4	chr20.21161882.C.G	independent_variable	6,53517E-05	0,000563378	0,115999682	0,907696982
ADV4	chr7.114554560.T.G	independent_variable	-5,83981E-05	0,000505951	-0,115422475	0,908156419
ADV4	chr5.88862329.G.C	independent_variable	5,72596E-05	0,000496621	0,115298454	0,908252648
ADV3	chr5.88461101.C.T	independent_variable	0,002165782	0,01880653	0,115161164	0,908360031

ADV2	chr5.88633148.G.T	independent_variable	-0,000128037	0,001116453	-0,114681748	0,908743511
ADV2	chr1.43744582.C.G	independent_variable	0,000128932	0,001124701	0,114636261	0,90877677
ADV2	chr1.43745339.A.G	independent_variable	0,000128932	0,001124701	0,114636261	0,90877677
ADV2	chr1.43746026.T.C	independent_variable	0,000128932	0,001124701	0,114636261	0,90877677
ADV2	chr1.43747271.A.G	independent_variable	0,000128932	0,001124701	0,114636261	0,90877677
ADV2	chr1.43747507.C.A	independent_variable	0,000128932	0,001124701	0,114636261	0,90877677
ADV2	chr1.43751205.T.C	independent_variable	0,000128932	0,001124701	0,114636261	0,90877677
ADV2	chr1.43753604.T.C	independent_variable	0,000128932	0,001124701	0,114636261	0,90877677
ADV2	chr1.43763926.G.A	independent_variable	0,000128932	0,001124701	0,114636261	0,90877677
ADV2	chr1.43777612.C.T	independent_variable	0,000128924	0,001124701	0,114629963	0,90878176
ADV4	chr5.104565723.C.T	independent_variable	5,83476E-05	0,000509708	0,114472616	0,908907085
ADV4	chr5.104568065.G.A	independent_variable	5,83476E-05	0,000509708	0,114472616	0,908907085
ADV5	chr1.43707752.G.A	independent_variable	-0,000567589	0,004960824	-0,114414213	0,908951897
ADV5	chr1.43722794.A.C	independent_variable	-0,000567589	0,004960824	-0,114414213	0,908951897
ADV5	chr1.43723048.A.G	independent_variable	-0,000567589	0,004960824	-0,114414213	0,908951897
ADV2	chr5.88456878.A.G	independent_variable	0,00011216	0,000981791	0,114240368	0,909091518
ADV2	chr5.88461004.C.T	independent_variable	0,00011216	0,000981791	0,114240368	0,909091518
ADV2	chr5.88467699.G.A	independent_variable	0,00011216	0,000981791	0,114240368	0,909091518
ADV2	chr5.88469954.T.C	independent_variable	0,00011216	0,000981791	0,114240368	0,909091518
ADV2	chr5.88475276.A.C	independent_variable	0,00011216	0,000981791	0,114240368	0,909091518
ADV2	chr5.88477838.A.G	independent_variable	0,00011216	0,000981791	0,114240368	0,909091518
ADV5	chr7.114520816.G.A	independent_variable	-0,000527137	0,00463159	-0,113813319	0,909430746
ADV1	chr7.114435085.T.C	independent_variable	-0,001916716	0,016940672	-0,113142842	0,909960892
ADV3	chr5.88416702.C.A	independent_variable	0,002104211	0,01895987	0,110982339	0,911673091
ADV5	chr1.43540668.A.G	independent_variable	0,000622057	0,005610637	0,110871051	0,911765432
ADV2	chr1.43721141.A.T	independent_variable	0,000119103	0,001080578	0,110221982	0,912274129
ADV2	chr1.43728863.A.G	independent_variable	0,000120601	0,001107353	0,108909154	0,913315078
ADV2	chr1.43729682.T.C	independent_variable	0,000120601	0,001107353	0,108909154	0,913315078
ADV2	chr5.88636991.G.T	independent_variable	-0,000120558	0,001116328	-0,107995334	0,914042803
ADV5	chr11.28632740.G.A	independent_variable	-0,000496724	0,004623575	-0,107432804	0,914485982
ADV5	chr11.28633321.G.A	independent_variable	-0,000496724	0,004623575	-0,107432804	0,914485982
ADV5	chr11.28633511.A.C	independent_variable	-0,000496724	0,004623575	-0,107432804	0,914485982
ADV5	chr11.28634125.G.T	independent_variable	-0,000496724	0,004623575	-0,107432804	0,914485982
ADV5	chr11.28634351.C.G	independent_variable	-0,000496724	0,004623575	-0,107432804	0,914485982
ADV5	chr11.28634462.T.G	independent_variable	-0,000496724	0,004623575	-0,107432804	0,914485982
ADV5	chr11.28635703.A.G	independent_variable	-0,000496724	0,004623575	-0,107432804	0,914485982
ADV5	chr11.28637410.G.A	independent_variable	-0,000496724	0,004623575	-0,107432804	0,914485982
ADV5	chr11.28638055.A.T	independent_variable	-0,000496724	0,004623575	-0,107432804	0,914485982
ADV5	chr11.28638658.T.C	independent_variable	-0,000496724	0,004623575	-0,107432804	0,914485982
ADV5	chr11.28644512.C.G	independent_variable	-0,000496724	0,004623575	-0,107432804	0,914485982
ADV5	chr11.28644517.G.A	independent_variable	-0,000496724	0,004623575	-0,107432804	0,914485982
ADV5	chr11.28644793.A.C	independent_variable	-0,000496724	0,004623575	-0,107432804	0,914485982
ADV5	chr20.21195965.C.T	independent_variable	-0,000536016	0,005008579	-0,107019631	0,914817297
ADV2	chr1.43616091.T.G	independent_variable	-0,000112489	0,001058077	-0,106314472	0,915373383
ADV5	chr11.28630347.G.C	independent_variable	-0,0004888	0,004657832	-0,104941474	0,916462177
ADV4	chr20.21166199.T.C	independent_variable	5,92813E-05	0,000568706	0,104238944	0,917019691
ADV4	chr20.21169627.G.C	independent_variable	5,92736E-05	0,000568706	0,104225315	0,917030501
ADV4	chr7.114540007.A.G	independent_variable	5,058E-05	0,000485635	0,104152311	0,917089747
ADV5	chr20.21161882.C.G	independent_variable	-0,000548046	0,005263272	-0,104126385	0,917108667
ADV5	chr3.20706828.T.G	independent_variable	-0,000530697	0,005099012	-0,104078324	0,917151779
ADV3	chr5.88455935.G.A	independent_variable	0,002016005	0,01937927	0,104028945	0,917186799
ADV3	chr5.88458988.C.T	independent_variable	0,002016005	0,01937927	0,104028945	0,917186799
ADV3	chr5.88467651.C.A	independent_variable	0,002016005	0,01937927	0,104028945	0,917186799
ADV3	chr5.88473876.C.T	independent_variable	0,002016005	0,01937927	0,104028945	0,917186799
ADV3	chr5.88477912.G.T	independent_variable	0,002016005	0,01937927	0,104028945	0,917186799
ADV5	chr11.28646124.G.A	independent_variable	-0,000483814	0,004664938	-0,103712845	0,91743654
ADV5	chr11.28646393.C.T	independent_variable	-0,000483814	0,004664938	-0,103712845	0,91743654
ADV5	chr11.28646869.T.C	independent_variable	-0,000483814	0,004664938	-0,103712845	0,91743654
ADV5	chr11.28648417.T.C	independent_variable	-0,000483814	0,004664938	-0,103712845	0,91743654
ADV5	chr11.28649222.A.G	independent_variable	-0,000483814	0,004664938	-0,103712845	0,91743654
ADV5	chr11.28649587.T.C	independent_variable	-0,000483814	0,004664938	-0,103712845	0,91743654
ADV5	chr11.28649653.C.G	independent_variable	-0,000483814	0,004664938	-0,103712845	0,91743654
ADV5	chr11.28650803.C.G	independent_variable	-0,000483814	0,004664938	-0,103712845	0,91743654
ADV5	chr11.28654623.A.G	independent_variable	-0,000483814	0,004664938	-0,103712845	0,91743654
ADV5	chr1.43883733.G.A	independent_variable	0,000496415	0,00479501	0,10352739	0,917586089
ADV4	chr5.104554395.C.T	independent_variable	-5,31098E-05	0,00051697	-0,102732789	0,918214807
ADV4	chr7.114508276.C.A	independent_variable	4,94181E-05	0,000483813	0,102142982	0,91868349
ADV5	chr3.20695381.T.C	independent_variable	-0,000513949	0,005037283	-0,102029022	0,918776695
ADV5	chr1.43510486.G.A	independent_variable	-0,000613314	0,006026798	-0,10176449	0,918988941
ADV5	chr1.43605550.G.A	independent_variable	0,000518005	0,005092706	0,101715081	0,919020941
ADV4	chr7.114451698.G.A	independent_variable	5,23471E-05	0,000515931	0,101461394	0,919221656

ADV5	chr1.43557944.G.A	independent_variable	0,000513811	0,005110039	0,100549424	0,919945745
ADV4	chr7.114442439.T.C	independent_variable	5,25496E-05	0,000522823	0,100511382	0,919976001
ADV5	chr11.28605957.T.C	independent_variable	-0,000456332	0,004575486	-0,09973409	0,920592313
ADV5	chr11.28622330.G.A	independent_variable	-0,000463895	0,004661221	-0,099522276	0,920761376
ADV5	chr11.28622366.T.C	independent_variable	-0,000463895	0,004661221	-0,099522276	0,920761376
ADV5	chr11.28622395.T.C	independent_variable	-0,000463895	0,004661221	-0,099522276	0,920761376
ADV5	chr11.28622424.G.A	independent_variable	-0,000463895	0,004661221	-0,099522276	0,920761376
ADV5	chr11.28622491.T.G	independent_variable	-0,000463895	0,004661221	-0,099522276	0,920761376
ADV5	chr11.28622565.G.T	independent_variable	-0,000463895	0,004661221	-0,099522276	0,920761376
ADV5	chr11.28622745.C.A	independent_variable	-0,000463895	0,004661221	-0,099522276	0,920761376
ADV5	chr11.28623055.A.G	independent_variable	-0,000463895	0,004661221	-0,099522276	0,920761376
ADV5	chr11.28623079.A.G	independent_variable	-0,000463895	0,004661221	-0,099522276	0,920761376
ADV5	chr11.28623338.T.C	independent_variable	-0,000463895	0,004661221	-0,099522276	0,920761376
ADV5	chr11.28624151.G.A	independent_variable	-0,000463895	0,004661221	-0,099522276	0,920761376
ADV5	chr11.28624272.T.G	independent_variable	-0,000463895	0,004661221	-0,099522276	0,920761376
ADV5	chr11.28625064.T.A	independent_variable	-0,000463895	0,004661221	-0,099522276	0,920761376
ADV5	chr11.28625609.A.T	independent_variable	-0,000463895	0,004661221	-0,099522276	0,920761376
ADV5	chr11.28626408.G.T	independent_variable	-0,000463895	0,004661221	-0,099522276	0,920761376
ADV5	chr11.28627124.G.A	independent_variable	-0,000463895	0,004661221	-0,099522276	0,920761376
ADV5	chr11.28628635.A.G	independent_variable	-0,000463895	0,004661221	-0,099522276	0,920761376
ADV5	chr11.28629811.A.C	independent_variable	-0,000463895	0,004661221	-0,099522276	0,920761376
ADV5	chr11.28631449.A.G	independent_variable	-0,000463895	0,004661221	-0,099522276	0,920761376
ADV4	chr7.114407085.A.T	independent_variable	5,1095E-05	0,000514434	0,099322738	0,920920377
ADV2	chr1.43682497.T.C	independent_variable	0,000107307	0,001081806	0,099192734	0,921021987
ADV2	chr1.43686518.G.A	independent_variable	0,000107307	0,001081806	0,099192734	0,921021987
ADV2	chr1.43687044.G.A	independent_variable	0,000107307	0,001081806	0,099192734	0,921021987
ADV2	chr1.43687948.G.C	independent_variable	0,000107307	0,001081806	0,099192734	0,921021987
ADV2	chr1.43688808.G.C	independent_variable	0,000107307	0,001081806	0,099192734	0,921021987
ADV1	chr7.114552490.T.C	independent_variable	0,001702272	0,017362709	0,098041867	0,921937872
ADV5	chr11.28631941.T.G	independent_variable	-0,000454349	0,004645664	-0,097800722	0,922127369
ADV5	chr11.28632083.T.C	independent_variable	-0,000454349	0,004645664	-0,097800722	0,922127369
ADV5	chr11.28632180.T.G	independent_variable	-0,000454349	0,004645664	-0,097800722	0,922127369
ADV5	chr11.28632479.C.T	independent_variable	-0,000454349	0,004645664	-0,097800722	0,922127369
ADV1	chr7.114464334.T.C	independent_variable	0,001627286	0,016697513	0,09745678	0,922399883
ADV4	chr20.21167328.G.C	independent_variable	5,46615E-05	0,000568026	0,096230616	0,923374207
ADV4	chr20.21170255.A.G	independent_variable	5,46615E-05	0,000568026	0,096230616	0,923374207
ADV4	chr20.21170684.G.A	independent_variable	5,46615E-05	0,000568026	0,096230616	0,923374207
ADV4	chr20.21170688.A.G	independent_variable	5,46615E-05	0,000568026	0,096230616	0,923374207
ADV4	chr20.21170708.C.T	independent_variable	5,46615E-05	0,000568026	0,096230616	0,923374207
ADV4	chr20.21171212.C.T	independent_variable	5,46615E-05	0,000568026	0,096230616	0,923374207
ADV4	chr20.21171591.G.A	independent_variable	5,46615E-05	0,000568026	0,096230616	0,923374207
ADV4	chr20.21172102.G.A	independent_variable	5,46615E-05	0,000568026	0,096230616	0,923374207
ADV4	chr20.21172498.G.A	independent_variable	5,46615E-05	0,000568026	0,096230616	0,923374207
ADV4	chr20.21173053.G.A	independent_variable	5,46615E-05	0,000568026	0,096230616	0,923374207
ADV4	chr20.21173632.G.C	independent_variable	5,46615E-05	0,000568026	0,096230616	0,923374207
ADV4	chr20.21173847.G.A	independent_variable	5,46615E-05	0,000568026	0,096230616	0,923374207
ADV2	chr1.43756926.G.A	independent_variable	0,000108071	0,001129803	0,095655124	0,923830707
ADV2	chr1.43674404.A.C	independent_variable	-0,000101353	0,001071409	-0,094598255	0,924669157
ADV2	chr5.88628064.T.G	independent_variable	-0,000103588	0,001119354	-0,092542489	0,926304326
ADV4	chr7.114475213.T.C	independent_variable	4,74862E-05	0,000517379	0,091782329	0,926905021
ADV2	chr5.88630645.G.A	independent_variable	-0,000102614	0,001119342	-0,091673229	0,926994605
ADV2	chr1.43563682.C.G	independent_variable	-8,95689E-05	0,000992428	-0,090252279	0,928121032
ADV5	chr1.43685962.T.G	independent_variable	0,000444201	0,004994072	0,08894569	0,929158049
ADV5	chr7.114522685.G.A	independent_variable	-0,000406874	0,004633011	-0,087820527	0,93005393
ADV5	chr7.114508188.A.G	independent_variable	-0,000402333	0,004634637	-0,086810029	0,930856722
ADV2	chr4.111314599.T.C	independent_variable	0,0001253	0,001443421	0,086807856	0,930857404
ADV3	chr3.20687599.C.T	independent_variable	0,001433456	0,016632284	0,086185146	0,931351008
ADV5	chr3.20703004.G.A	independent_variable	-0,000439165	0,005114414	-0,085868021	0,931608135
ADV4	chr1.43840448.A.G	independent_variable	-4,48814E-05	0,000524671	-0,085541901	0,931863073
ADV4	chr1.43845072.C.T	independent_variable	-4,48814E-05	0,000524671	-0,085541901	0,931863073
ADV4	chr1.43845203.G.A	independent_variable	-4,48814E-05	0,000524671	-0,085541901	0,931863073
ADV4	chr5.104554297.G.C	independent_variable	4,27171E-05	0,000507352	0,084196176	0,932933177
ADV2	chr1.43549154.A.G	independent_variable	-9,69213E-05	0,001152629	-0,084087155	0,933025207
ADV2	chr1.43569422.A.G	independent_variable	8,30263E-05	0,00099105	0,083776072	0,933265752
ADV5	chr20.21161712.T.C	independent_variable	-0,000432301	0,005244879	-0,082423493	0,934341191
ADV5	chr20.21154158.T.C	independent_variable	-0,000402608	0,004961034	-0,081153985	0,935349341
ADV5	chr1.43853701.G.A	independent_variable	-0,000405456	0,005019342	-0,080778714	0,935647646
ADV5	chr11.28622480.G.A	independent_variable	-0,000376445	0,004676322	-0,080500215	0,935870043
ADV2	chr1.43644078.A.G	independent_variable	8,74504E-05	0,001088743	0,08032237	0,936010681
ADV4	chr5.88526854.T.A	independent_variable	4,56485E-05	0,000574933	0,079397964	0,936748837
ADV5	chr1.43576868.C.G	independent_variable	-0,000400456	0,005078678	-0,078850368	0,937181179

ADV2	chr1.43783814.A.G	independent_variable	-8,70353E-05	0,001106231	-0,078677295	0,937319169
ADV1	chr7.114407085.A.T	independent_variable	0,001282291	0,016909813	0,075831153	0,939582652
ADV5	chr4.111311007.T.C	independent_variable	-0,000532638	0,007028374	-0,075783886	0,93961996
ADV1	chr7.114507162.C.A	independent_variable	0,001290969	0,017172841	0,075175012	0,940104771
ADV5	chr11.28655190.A.G	independent_variable	-0,000349899	0,004667529	-0,074964409	0,940271369
ADV5	chr11.28655717.T.C	independent_variable	-0,000349899	0,004667529	-0,074964409	0,940271369
ADV5	chr11.28623283.A.G	independent_variable	-0,00034622	0,004658912	-0,07431349	0,940789089
ADV5	chr11.28623794.G.C	independent_variable	-0,00034622	0,004658912	-0,07431349	0,940789089
ADV5	chr11.28628859.C.T	independent_variable	-0,00034622	0,004658912	-0,07431349	0,940789089
ADV4	chr5.104557847.T.A	independent_variable	-3,83643E-05	0,000516528	-0,074273432	0,94082149
ADV4	chr20.21267478.G.A	independent_variable	3,88579E-05	0,000523849	0,074177728	0,940897826
ADV4	chr1.43610348.G.A	independent_variable	-3,8374E-05	0,000521166	-0,073631049	0,94133103
ADV5	chr20.21158348.G.A	independent_variable	-0,000383582	0,005244887	-0,073134479	0,941726801
ADV5	chr20.21158611.G.A	independent_variable	-0,000383582	0,005244887	-0,073134479	0,941726801
ADV5	chr20.21158741.C.G	independent_variable	-0,000383582	0,005244887	-0,073134479	0,941726801
ADV5	chr20.21159530.G.A	independent_variable	-0,000383582	0,005244887	-0,073134479	0,941726801
ADV5	chr20.21159701.C.T	independent_variable	-0,000383582	0,005244887	-0,073134479	0,941726801
ADV5	chr1.43887565.G.A	independent_variable	0,000339203	0,004754098	0,071349693	0,943147638
ADV5	chr4.111311550.T.C	independent_variable	-0,000499166	0,007021306	-0,071093045	0,943350777
ADV5	chr4.111328737.T.G	independent_variable	-0,000499166	0,007021306	-0,071093045	0,943350777
ADV5	chr1.43558813.A.G	independent_variable	0,00035736	0,005067079	0,070525904	0,943801049
ADV2	chr7.114605694.T.A	independent_variable	6,71294E-05	0,000952407	0,070483944	0,943836493
ADV4	chr7.114482451.C.T	independent_variable	-3,66768E-05	0,000522765	-0,070159282	0,944092905
ADV5	chr1.43692458.C.G	independent_variable	-0,000350501	0,005007659	-0,069992882	0,944225189
ADV5	chr1.43577434.T.C	independent_variable	0,000351691	0,005099168	0,068970249	0,94503912
ADV4	chr7.114499629.G.A	independent_variable	-3,50778E-05	0,000516633	-0,067896876	0,945892858
ADV3	chr7.114435631.A.C	independent_variable	-0,001214598	0,017896133	-0,067869279	0,945915682
ADV3	chr5.88445076.T.C	independent_variable	0,001308252	0,019450524	0,0672605	0,946400426
ADV3	chr5.88468801.G.A	independent_variable	0,001308252	0,019450524	0,0672605	0,946400426
ADV5	chr1.43674404.A.C	independent_variable	-0,000328325	0,004970295	-0,066057343	0,947356711
ADV2	chr1.43584333.G.A	independent_variable	-7,22751E-05	0,001101302	-0,065626961	0,947699293
ADV2	chr1.43584356.A.C	independent_variable	-7,22751E-05	0,001101302	-0,065626961	0,947699293
ADV2	chr1.43586706.T.G	independent_variable	-7,22751E-05	0,001101302	-0,065626961	0,947699293
ADV4	chr7.114434202.C.T	independent_variable	-3,29311E-05	0,000508245	-0,064793794	0,948362988
ADV4	chr5.104568407.A.G	independent_variable	3,29251E-05	0,000513254	0,064149681	0,948875696
ADV5	chr11.28658309.G.A	independent_variable	-0,000298734	0,004674865	-0,063902266	0,949072233
ADV4	chr1.43837594.A.G	independent_variable	3,49241E-05	0,00054731	0,063810379	0,949145595
ADV4	chr7.114483253.C.T	independent_variable	3,28969E-05	0,000518139	0,063490447	0,949399461
ADV4	chr7.114456791.A.T	independent_variable	3,23777E-05	0,000521608	0,062072902	0,95052783
ADV2	chr1.43546066.G.A	independent_variable	-6,92789E-05	0,001136111	-0,060978951	0,951403349
ADV2	chr1.43692458.C.G	independent_variable	6,49993E-05	0,001079706	0,060200924	0,952017841
ADV5	chr1.43682497.T.C	independent_variable	-0,000301672	0,005017436	-0,060124656	0,952078556
ADV5	chr1.43686518.G.A	independent_variable	-0,000301672	0,005017436	-0,060124656	0,952078556
ADV5	chr1.43687044.G.A	independent_variable	-0,000301672	0,005017436	-0,060124656	0,952078556
ADV5	chr1.43687948.G.C	independent_variable	-0,000301672	0,005017436	-0,060124656	0,952078556
ADV5	chr1.43688808.G.C	independent_variable	-0,000301672	0,005017436	-0,060124656	0,952078556
ADV1	chr5.88416702.C.A	independent_variable	0,001076188	0,017931527	0,060016505	0,952165503
ADV1	chr7.114503596.T.A	independent_variable	0,000979827	0,01666719	0,058787749	0,953142915
ADV4	chr5.104559401.T.G	independent_variable	-3,03448E-05	0,000517826	-0,058600345	0,953293078
ADV5	chr1.43563242.G.A	independent_variable	-0,000285502	0,004876663	-0,058544485	0,95333679
ADV2	chr1.44009451.C.T	independent_variable	-7,70426E-05	0,001343258	-0,057355042	0,954286293
ADV1	chr7.114499629.G.A	independent_variable	0,000949049	0,016690971	0,056860046	0,954677714
ADV5	chr1.43547252.C.T	independent_variable	0,000318683	0,005637023	0,056534003	0,954940181
ADV5	chr11.28627190.A.G	independent_variable	-0,000259255	0,004588789	-0,056497402	0,954966502
ADV5	chr3.20700799.C.T	independent_variable	-0,000285838	0,005162273	-0,055370601	0,95586705
ADV2	chr1.43900578.G.A	independent_variable	6,49431E-05	0,001195656	0,054315834	0,956704763
ADV5	chr7.114508276.C.A	independent_variable	-0,000249886	0,004630466	-0,053965588	0,956983797
ADV5	chr1.43721141.A.T	independent_variable	-0,000261396	0,005011393	-0,052160291	0,958420191
ADV1	chr5.88401026.G.A	independent_variable	0,000898777	0,017952726	0,050063557	0,960090964
ADV2	chr4.111310701.T.C	independent_variable	-6,96341E-05	0,001413547	-0,049261921	0,960729372
ADV2	chr11.28554714.G.T	independent_variable	-5,22856E-05	0,001070245	-0,048853847	0,961056434
ADV2	chr5.104578822.T.C	independent_variable	-4,76005E-05	0,000978953	-0,048623921	0,961236885
ADV2	chr7.114589084.G.A	independent_variable	-4,41356E-05	0,000917164	-0,04812183	0,961637414
ADV5	chr1.43552933.G.T	independent_variable	0,000244334	0,005097446	0,047932694	0,961787671
ADV2	chr1.43565986.C.T	independent_variable	4,68964E-05	0,000996204	0,047075123	0,962470921
ADV2	chr7.114627941.T.C	independent_variable	-4,50947E-05	0,000964632	-0,04674813	0,962732786
ADV5	chr4.111333132.A.G	independent_variable	0,000322861	0,007165962	0,045054842	0,964080727
ADV2	chr1.43563291.G.A	independent_variable	4,40175E-05	0,000994917	0,04424236	0,964727694
ADV5	chr4.111309356.T.A	independent_variable	0,000304701	0,006917321	0,044048976	0,964882018
ADV2	chr5.88605345.G.A	independent_variable	-4,65573E-05	0,001122453	-0,041478188	0,966931206
ADV2	chr5.88618013.G.A	independent_variable	-4,65573E-05	0,001122453	-0,041478188	0,966931206

ADV2	chr5.88618446.A.G	independent_variable	-4,65573E-05	0,001122453	-0,041478188	0,966931206
ADV2	chr5.88619663.G.T	independent_variable	-4,65573E-05	0,001122453	-0,041478188	0,966931206
ADV5	chr1.43574039.T.C	independent_variable	0,000211655	0,005186276	0,040810616	0,967462332
ADV4	chr7.114503596.T.A	independent_variable	-2,03764E-05	0,000515899	-0,039496946	0,968508786
ADV4	chr7.114435631.A.C	independent_variable	-1,87165E-05	0,000507538	-0,036877017	0,970597163
ADV2	chr1.43592279.T.C	independent_variable	-4,01788E-05	0,001099183	-0,036553293	0,970854794
ADV5	chr1.43504626.G.A	independent_variable	-0,000214168	0,005952226	-0,03598117	0,971313436
ADV2	chr1.43550138.T.C	independent_variable	-4,14628E-05	0,001171582	-0,035390408	0,97178452
ADV5	chr7.114407085.A.T	independent_variable	0,000173992	0,004943729	0,035194415	0,971938247
ADV4	chr20.21189014.G.A	independent_variable	-1,75068E-05	0,000524737	-0,033362883	0,97339801
ADV4	chr1.43848594.C.T	independent_variable	-1,69593E-05	0,00051852	-0,032707077	0,973920517
ADV3	chr7.114435085.T.C	independent_variable	0,000586713	0,017962776	0,032662716	0,973956065
ADV2	chr11.28511111.T.C	independent_variable	3,41987E-05	0,001075944	0,03178485	0,974657121
ADV4	chr5.88851954.G.A	independent_variable	-1,56778E-05	0,000495822	-0,03161984	0,974787261
ADV4	chr5.88852224.C.T	independent_variable	-1,56778E-05	0,000495822	-0,03161984	0,974787261
ADV4	chr5.88853474.A.G	independent_variable	-1,56778E-05	0,000495822	-0,03161984	0,974787261
ADV4	chr5.88853509.G.A	independent_variable	-1,56778E-05	0,000495822	-0,03161984	0,974787261
ADV4	chr5.88854472.G.A	independent_variable	-1,56778E-05	0,000495822	-0,03161984	0,974787261
ADV4	chr5.88855175.T.A	independent_variable	-1,56778E-05	0,000495822	-0,03161984	0,974787261
ADV4	chr5.88856385.T.C	independent_variable	-1,56778E-05	0,000495822	-0,03161984	0,974787261
ADV4	chr5.88856757.C.A	independent_variable	-1,56778E-05	0,000495822	-0,03161984	0,974787261
ADV4	chr5.88858057.C.T	independent_variable	-1,56778E-05	0,000495822	-0,03161984	0,974787261
ADV5	chr1.43565822.A.G	independent_variable	0,000160627	0,005212695	0,030814659	0,975428906
ADV2	chr5.88624488.C.A	independent_variable	-3,36744E-05	0,001123883	-0,02996256	0,976108868
ADV4	chr1.43612713.T.C	independent_variable	1,4447E-05	0,000522039	0,027674144	0,977932251
ADV4	chr1.43613740.T.C	independent_variable	1,4447E-05	0,000522039	0,027674144	0,977932251
ADV4	chr1.43631859.T.C	independent_variable	1,4447E-05	0,000522039	0,027674144	0,977932251
ADV1	chr7.114435631.A.C	independent_variable	0,000464496	0,016884871	0,027509581	0,978063812
ADV3	chr7.114434202.C.T	independent_variable	-0,000489041	0,017921165	-0,02728848	0,978240074
ADV4	chr1.43840750.C.T	independent_variable	-1,41887E-05	0,000522771	-0,027141309	0,978357301
ADV2	chr1.43579794.A.G	independent_variable	-2,99319E-05	0,001114691	-0,026852211	0,97858766
ADV2	chr1.43634413.G.A	independent_variable	-2,71966E-05	0,001043705	-0,026057755	0,979220863
ADV1	chr7.114442439.T.C	independent_variable	-0,000419789	0,016879212	-0,02487019	0,980167778
ADV5	chr7.114554560.T.G	independent_variable	-0,000115837	0,004682421	-0,024738783	0,980273151
ADV1	chr7.114475213.T.C	independent_variable	0,000405471	0,016715151	0,024257672	0,980656037
ADV4	chr7.114435085.T.C	independent_variable	1,22778E-05	0,000514477	0,02386454	0,980969796
ADV1	chr7.114474994.A.G	independent_variable	-0,000390622	0,016619404	-0,023503983	0,981256896
ADV4	chr20.21141038.A.T	independent_variable	1,23113E-05	0,00055404	0,022220869	0,982280165
ADV1	chr7.114440281.T.G	independent_variable	-0,000336446	0,016859085	-0,01995637	0,984085631
ADV5	chr11.28621752.T.C	independent_variable	-9,21944E-05	0,004830857	-0,019084481	0,984781089
ADV2	chr5.88445076.T.C	independent_variable	1,81202E-05	0,000978146	0,018525011	0,985227199
ADV2	chr5.88468801.G.A	independent_variable	1,81202E-05	0,000978146	0,018525011	0,985227199
ADV1	chr5.88456878.A.G	independent_variable	0,000309143	0,018531956	0,016681619	0,986697034
ADV1	chr5.88461004.C.T	independent_variable	0,000309143	0,018531956	0,016681619	0,986697034
ADV1	chr5.88467699.G.A	independent_variable	0,000309143	0,018531956	0,016681619	0,986697034
ADV1	chr5.88469954.T.C	independent_variable	0,000309143	0,018531956	0,016681619	0,986697034
ADV1	chr5.88475276.A.C	independent_variable	0,000309143	0,018531956	0,016681619	0,986697034
ADV1	chr5.88477838.A.G	independent_variable	0,000309143	0,018531956	0,016681619	0,986697034
ADV2	chr5.88879434.C.G	independent_variable	1,73027E-05	0,001103265	0,015683184	0,987493899
ADV3	chr5.88417014.A.G	independent_variable	0,000289369	0,019067066	0,015176398	0,987897305
ADV2	chr11.28522265.G.A	independent_variable	-1,2444E-05	0,00107094	-0,011619739	0,990733908
ADV1	chr7.114434202.C.T	independent_variable	-0,000156456	0,016908436	-0,009253159	0,992620688
ADV2	chr4.111322536.C.G	independent_variable	-8,76676E-06	0,001422474	-0,006163036	0,995084994
ADV2	chr11.28504006.A.G	independent_variable	-6,36471E-06	0,001075223	-0,005919428	0,995279519
ADV2	chr5.104581838.T.A	independent_variable	4,93407E-06	0,000991003	0,00497887	0,996029305
ADV2	chr5.104582136.T.C	independent_variable	4,93407E-06	0,000991003	0,00497887	0,996029305
ADV2	chr5.88455935.G.A	independent_variable	-4,80337E-06	0,000974673	-0,004928182	0,996069801
ADV2	chr5.88458988.C.T	independent_variable	-4,80337E-06	0,000974673	-0,004928182	0,996069801
ADV2	chr5.88467651.C.A	independent_variable	-4,80337E-06	0,000974673	-0,004928182	0,996069801
ADV2	chr5.88473876.C.T	independent_variable	-4,80337E-06	0,000974673	-0,004928182	0,996069801
ADV2	chr5.88477912.G.T	independent_variable	-4,80337E-06	0,000974673	-0,004928182	0,996069801

Note : ADV1 = European component, ADV2 = American Component, ADV3 = South Asian Component, ADV4 = African Component, ADV5 = East Asian Component.

Supplementary Table 6.

rsid	chrom	start	end	ranking	probability_score	ChIP	Chromatin_accessibility	PWM	Footprint	QTL	PWM_matched	Footprint_matched	IC_max	IC_matched_max
rs2842192	chr1	43504625	43504626	1f	0,22271	FALSE	TRUE	FALSE	FALSE	TRUE	FALSE	FALSE	0	0
rs882464	chr1	43509092	43509093	1f	0,55436	TRUE	TRUE	FALSE	FALSE	TRUE	FALSE	FALSE	0	0
rs2819341	chr1	43510485	43510486	1f	0,55436	TRUE	TRUE	FALSE	FALSE	TRUE	FALSE	FALSE	0	0
rs10789434	chr1	43510949	43510950	1f	0,12417	FALSE	TRUE	TRUE	TRUE	TRUE	FALSE	FALSE	0,35	0
rs2819330	chr1	43537285	43537286	1f	0,55436	TRUE	TRUE	FALSE	FALSE	TRUE	FALSE	FALSE	0	0
rs2782639	chr1	43540667	43540668	1f	0,66703	TRUE	TRUE	FALSE	TRUE	TRUE	FALSE	FALSE	0	0
rs2842189	chr1	43541976	43541977	1b	0,92429	TRUE	TRUE	TRUE	TRUE	TRUE	FALSE	FALSE	0,14	0
rs2782640	chr1	43543361	43543362	1f	0,55436	TRUE	TRUE	FALSE	FALSE	TRUE	FALSE	FALSE	0	0
rs951740	chr1	43546065	43546066	1f	0,70823	TRUE	TRUE	FALSE	TRUE	TRUE	FALSE	TRUE	0	0
rs1889588	chr1	43547251	43547252	1f	0,55324	TRUE	FALSE	FALSE	FALSE	TRUE	FALSE	FALSE	0	0
rs2842188	chr1	43548608	43548609	1f	0,55436	TRUE	TRUE	FALSE	FALSE	TRUE	FALSE	FALSE	0	0
rs2819333	chr1	43548901	43548902	1f	0,55436	TRUE	TRUE	FALSE	FALSE	TRUE	FALSE	FALSE	0	0
rs2819334	chr1	43549063	43549064	1f	0,55436	TRUE	TRUE	FALSE	FALSE	TRUE	FALSE	FALSE	0	0
rs2819335	chr1	43549153	43549154	1f	0,55436	TRUE	TRUE	FALSE	FALSE	TRUE	FALSE	FALSE	0	0
rs2842187	chr1	43549277	43549278	1f	0,55436	TRUE	TRUE	FALSE	FALSE	TRUE	FALSE	FALSE	0	0
rs2819336	chr1	43550137	43550138	1f	0,55436	TRUE	TRUE	FALSE	FALSE	TRUE	FALSE	FALSE	0	0
rs11580258	chr1	43550343	43550344	1f	0,55436	TRUE	TRUE	FALSE	FALSE	TRUE	FALSE	FALSE	0	0
rs2819339	chr1	43552932	43552933	1f	0,55436	TRUE	TRUE	FALSE	FALSE	TRUE	FALSE	FALSE	0	0
rs2842185	chr1	43554059	43554060	1f	0,66703	TRUE	TRUE	FALSE	TRUE	TRUE	FALSE	FALSE	0	0
rs2842183	chr1	43554722	43554723	1f	0,55436	TRUE	TRUE	FALSE	FALSE	TRUE	FALSE	FALSE	0	0
rs10890253	chr1	43555784	43555785	1f	0,55324	TRUE	FALSE	FALSE	FALSE	TRUE	FALSE	FALSE	0	0
rs2842178	chr1	43556355	43556356	1f	0,44208	TRUE	FALSE	TRUE	FALSE	TRUE	FALSE	FALSE	0,7	0
rs2494995	chr1	43556631	43556632	1f	0,55324	TRUE	FALSE	FALSE	FALSE	TRUE	FALSE	FALSE	0	0
rs2842176	chr1	43556862	43556863	1f	0,125	FALSE	TRUE	TRUE	FALSE	TRUE	FALSE	FALSE	1,55	0
rs2842175	chr1	43557943	43557944	1f	0,66703	TRUE	TRUE	FALSE	TRUE	TRUE	FALSE	FALSE	0	0
rs11580074	chr1	43558812	43558813	1b	0,98667	TRUE	TRUE	TRUE	TRUE	TRUE	FALSE	FALSE	1,6	0
rs11210869	chr1	43560368	43560369	1f	0,55436	TRUE	TRUE	FALSE	FALSE	TRUE	FALSE	FALSE	0	0
rs10789435	chr1	43560381	43560382	1b	0,96	TRUE	TRUE	TRUE	TRUE	TRUE	FALSE	FALSE	1,64	0
rs3000761	chr1	43560417	43560418	1b	0,33	TRUE	TRUE	TRUE	TRUE	TRUE	FALSE	FALSE	1,13	0
rs68191270	chr1	43560508	43560509	1b	0,20849	TRUE	TRUE	TRUE	TRUE	TRUE	FALSE	FALSE	0,23	0
rs12076635	chr1	43560984	43560985	1f	0,55436	TRUE	TRUE	FALSE	FALSE	TRUE	FALSE	FALSE	0	0
rs61769611	chr1	43563241	43563242	7	0,51392	FALSE	FALSE	FALSE	FALSE	TRUE	FALSE	FALSE	0	0
rs2842194	chr1	43563290	43563291	7	0,51392	FALSE	FALSE	FALSE	FALSE	TRUE	FALSE	FALSE	0	0
rs11210871	chr1	43563681	43563682	1f	0,55436	TRUE	TRUE	FALSE	FALSE	TRUE	FALSE	FALSE	0	0
rs10890255	chr1	43565821	43565822	1f	0,55436	TRUE	TRUE	FALSE	FALSE	TRUE	FALSE	FALSE	0	0
rs2478978	chr1	43565985	43565986	1d	0,938	TRUE	TRUE	TRUE	FALSE	TRUE	FALSE	FALSE	0,71	0
rs11586016	chr1	43566121	43566122	1f	0,55436	TRUE	TRUE	FALSE	FALSE	TRUE	FALSE	FALSE	0	0
rs12089622	chr1	43568096	43568097	6	0,0475	FALSE	FALSE	TRUE	TRUE	TRUE	FALSE	FALSE	0,47	0
rs2842186	chr1	43569421	43569422	1f	0,55436	TRUE	TRUE	FALSE	FALSE	TRUE	FALSE	FALSE	0	0
rs943513	chr1	43569800	43569801	1a	0,93	TRUE	TRUE	TRUE	TRUE	TRUE	TRUE	TRUE	1,65	0,09
rs1887402	chr1	43570413	43570414	1f	0,08	FALSE	TRUE	TRUE	TRUE	TRUE	FALSE	FALSE	1,5	0
rs3001723	chr1	43572013	43572014	1b	0,77931	TRUE	TRUE	TRUE	TRUE	TRUE	FALSE	FALSE	1,5	0
rs2819340	chr1	43574038	43574039	1f	0,55324	TRUE	FALSE	FALSE	FALSE	TRUE	FALSE	FALSE	0	0
rs2842171	chr1	43574178	43574179	1f	0,25504	FALSE	TRUE	TRUE	FALSE	TRUE	FALSE	FALSE	0,17	0
rs10789436	chr1	43575294	43575295	1f	0,55436	TRUE	TRUE	FALSE	FALSE	TRUE	FALSE	FALSE	0	0
rs650729	chr1	43576867	43576868	1f	0,22271	FALSE	TRUE	FALSE	FALSE	TRUE	FALSE	FALSE	0	0
rs663618	chr1	43577433	43577434	1f	0,55436	TRUE	TRUE	FALSE	FALSE	TRUE	FALSE	FALSE	0	0
rs552638	chr1	43577720	43577721	1f	0,55436	TRUE	TRUE	FALSE	FALSE	TRUE	FALSE	FALSE	0	0

rs2004899	chr1	43579793	43579794	6	0,26331	FALSE	FALSE	TRUE	FALSE	TRUE	FALSE	FALSE	1,81	0
rs1808730	chr1	43579856	43579857	6	0,2927	FALSE	FALSE	TRUE	FALSE	TRUE	FALSE	FALSE	0,78	0
rs3791134	chr1	43583484	43583485	1f	0,55324	TRUE	FALSE	FALSE	FALSE	TRUE	FALSE	FALSE	0	0
rs3791136	chr1	43584275	43584276	1f	0,55436	TRUE	TRUE	FALSE	FALSE	TRUE	FALSE	FALSE	0	0
rs3791137	chr1	43584332	43584333	1f	0,55436	TRUE	TRUE	FALSE	FALSE	TRUE	FALSE	FALSE	0	0
rs3791138	chr1	43584355	43584356	1f	0,55436	TRUE	TRUE	FALSE	FALSE	TRUE	FALSE	FALSE	0	0
rs3828150	chr1	43585184	43585185	1f	0,66703	TRUE	TRUE	FALSE	TRUE	TRUE	FALSE	FALSE	0	0
rs6698389	chr1	43585902	43585903	1b	0,92754	TRUE	TRUE	TRUE	TRUE	TRUE	FALSE	FALSE	0,01	0
rs61769642	chr1	43585958	43585959	1f	0,55436	TRUE	TRUE	FALSE	FALSE	TRUE	FALSE	FALSE	0	0
rs3791139	chr1	43586099	43586100	1b	0,43617	TRUE	TRUE	TRUE	TRUE	TRUE	FALSE	FALSE	0,65	0
rs3791140	chr1	43586106	43586107	1b	0,9225	TRUE	TRUE	TRUE	TRUE	TRUE	FALSE	FALSE	1,06	0
rs501299	chr1	43586162	43586163	1f	0,55324	TRUE	FALSE	FALSE	FALSE	TRUE	FALSE	FALSE	0	0
rs10890261	chr1	43586705	43586706	1f	0,55436	TRUE	TRUE	FALSE	FALSE	TRUE	FALSE	FALSE	0	0
rs573350	chr1	43592278	43592279	1f	0,55436	TRUE	TRUE	FALSE	FALSE	TRUE	FALSE	FALSE	0	0
rs605709	chr1	43592795	43592796	1f	0,55436	TRUE	TRUE	FALSE	FALSE	TRUE	FALSE	FALSE	0	0
rs72673082	chr1	43593570	43593571	1f	0,66703	TRUE	TRUE	FALSE	TRUE	TRUE	FALSE	FALSE	0	0
rs583040	chr1	43594811	43594812	1f	0,55324	TRUE	FALSE	FALSE	FALSE	TRUE	FALSE	FALSE	0	0
rs674725	chr1	43596123	43596124	1f	0,55436	TRUE	TRUE	FALSE	FALSE	TRUE	FALSE	FALSE	0	0
rs673253	chr1	43596482	43596483	1f	0,55324	TRUE	FALSE	FALSE	FALSE	TRUE	FALSE	FALSE	0	0
rs673242	chr1	43596487	43596488	1f	0,55324	TRUE	FALSE	FALSE	FALSE	TRUE	FALSE	FALSE	0	0
rs56175694	chr1	43599476	43599477	7	0,51392	FALSE	FALSE	FALSE	FALSE	TRUE	FALSE	FALSE	0	0
rs596522	chr1	43600752	43600753	1f	0,55324	TRUE	FALSE	FALSE	FALSE	TRUE	FALSE	FALSE	0	0
rs530373	chr1	43604360	43604361	1f	0,55436	TRUE	TRUE	FALSE	FALSE	TRUE	FALSE	FALSE	0	0
rs17371903	chr1	43605019	43605020	1b	0,36991	TRUE	TRUE	TRUE	TRUE	TRUE	FALSE	FALSE	1,85	0
rs631248	chr1	43605549	43605550	1f	0,55324	TRUE	FALSE	FALSE	FALSE	TRUE	FALSE	FALSE	0	0
rs539096	chr1	43606748	43606749	1f	0,55436	TRUE	TRUE	FALSE	FALSE	TRUE	FALSE	FALSE	0	0
rs519669	chr1	43609833	43609834	1f	0,22271	FALSE	TRUE	FALSE	FALSE	TRUE	FALSE	FALSE	0	0
rs11210887	chr1	43610347	43610348	1b	0,04975	TRUE	TRUE	TRUE	TRUE	TRUE	FALSE	FALSE	0,9	0
rs549845	chr1	43610797	43610798	6	0,05	FALSE	FALSE	TRUE	TRUE	TRUE	FALSE	FALSE	1,59	0
rs499257	chr1	43612712	43612713	1f	0,55436	TRUE	TRUE	FALSE	FALSE	TRUE	FALSE	FALSE	0	0
rs603542	chr1	43613739	43613740	1b	0,95092	TRUE	TRUE	TRUE	TRUE	TRUE	FALSE	FALSE	1,63	0
rs588657	chr1	43614740	43614741	1f	0,22271	FALSE	TRUE	FALSE	FALSE	TRUE	FALSE	FALSE	0	0
rs516790	chr1	43616040	43616041	1f	0,55436	TRUE	TRUE	FALSE	FALSE	TRUE	FALSE	FALSE	0	0
rs516921	chr1	43616090	43616091	1f	0,66703	TRUE	TRUE	FALSE	TRUE	TRUE	FALSE	FALSE	0	0
rs517849	chr1	43616201	43616202	1f	0,22271	FALSE	TRUE	FALSE	FALSE	TRUE	FALSE	FALSE	0	0
rs667676	chr1	43616544	43616545	1f	0,13817	FALSE	TRUE	TRUE	TRUE	TRUE	FALSE	FALSE	1,85	0
rs653953	chr1	43617343	43617344	1b	0,35082	TRUE	TRUE	TRUE	TRUE	TRUE	FALSE	FALSE	1,86	0
rs571862	chr1	43617438	43617439	1f	0,55436	TRUE	TRUE	FALSE	FALSE	TRUE	FALSE	FALSE	0	0
rs641365	chr1	43617835	43617836	1f	0,03845	FALSE	TRUE	TRUE	TRUE	TRUE	FALSE	FALSE	0	0
rs641351	chr1	43617847	43617848	1f	0,02937	FALSE	TRUE	TRUE	TRUE	TRUE	FALSE	FALSE	0,01	0
rs639929	chr1	43618202	43618203	1f	0,66703	TRUE	TRUE	FALSE	TRUE	TRUE	FALSE	FALSE	0	0
rs1143701	chr1	43619067	43619068	1f	0,22271	FALSE	TRUE	FALSE	FALSE	TRUE	FALSE	FALSE	0	0
rs568639	chr1	43620408	43620409	1b	0,99173	TRUE	TRUE	TRUE	TRUE	TRUE	FALSE	FALSE	0,51	0
rs1143702	chr1	43621159	43621160	1b	0,53794	TRUE	TRUE	TRUE	TRUE	TRUE	FALSE	FALSE	0,17	0
rs673485	chr1	43621824	43621825	1b	0,46817	TRUE	TRUE	TRUE	TRUE	TRUE	FALSE	FALSE	1,45	0
rs643445	chr1	43623960	43623961	1f	0,55436	TRUE	TRUE	FALSE	FALSE	TRUE	FALSE	FALSE	0	0
rs559575	chr1	43624268	43624269	1f	0,55436	TRUE	TRUE	FALSE	FALSE	TRUE	FALSE	FALSE	0	0
rs531459	chr1	43625003	43625004	1f	0,22271	FALSE	TRUE	FALSE	FALSE	TRUE	FALSE	FALSE	0	0
rs628342	chr1	43625064	43625065	1f	0,22271	FALSE	TRUE	FALSE	FALSE	TRUE	FALSE	FALSE	0	0
rs2916568	chr1	43625602	43625603	1f	0,55436	TRUE	TRUE	FALSE	FALSE	TRUE	FALSE	FALSE	0	0
rs10890268	chr1	43629436	43629437	1f	0,22271	FALSE	TRUE	FALSE	FALSE	TRUE	FALSE	FALSE	0	0

rs12405972	chr1	43631766	43631767	1b	0,98667	TRUE	TRUE	TRUE	TRUE	TRUE	FALSE	FALSE	1,6	0
rs2970609	chr1	43631842	43631843	1d	0,90947	TRUE	TRUE	TRUE	FALSE	TRUE	FALSE	FALSE	0,15	0
rs2970610	chr1	43631858	43631859	1f	0,66703	TRUE	TRUE	FALSE	TRUE	TRUE	FALSE	FALSE	0	0
rs61769649	chr1	43632834	43632835	1f	0,22271	FALSE	TRUE	FALSE	FALSE	TRUE	FALSE	FALSE	0	0
rs585711	chr1	43634191	43634192	1f	0,55436	TRUE	TRUE	FALSE	FALSE	TRUE	FALSE	FALSE	0	0
rs585652	chr1	43634238	43634239	1f	0,55436	TRUE	TRUE	FALSE	FALSE	TRUE	FALSE	FALSE	0	0
rs11210892	chr1	43634412	43634413	1f	0,55436	TRUE	TRUE	FALSE	FALSE	TRUE	FALSE	FALSE	0	0
rs664728	chr1	43636042	43636043	1f	0,55436	TRUE	TRUE	FALSE	FALSE	TRUE	FALSE	FALSE	0	0
rs664336	chr1	43636128	43636129	1f	0,55436	TRUE	TRUE	FALSE	FALSE	TRUE	FALSE	FALSE	0	0
rs530201	chr1	43636585	43636586	1f	0,55436	TRUE	TRUE	FALSE	FALSE	TRUE	FALSE	FALSE	0	0
rs2970606	chr1	43637826	43637827	7	0,51392	FALSE	FALSE	FALSE	FALSE	TRUE	FALSE	FALSE	0	0
rs564318	chr1	43638042	43638043	1f	0,55436	TRUE	TRUE	FALSE	FALSE	TRUE	FALSE	FALSE	0	0
rs486883	chr1	43639332	43639333	1f	0,55436	TRUE	TRUE	FALSE	FALSE	TRUE	FALSE	FALSE	0	0
rs649504	chr1	43640276	43640277	1a	0,66783	TRUE	TRUE	TRUE	TRUE	TRUE	TRUE	TRUE	1,99	0,05
rs574736	chr1	43640594	43640595	1b	0,97052	TRUE	TRUE	TRUE	TRUE	TRUE	FALSE	FALSE	1,89	0
rs636547	chr1	43640844	43640845	1f	0,55436	TRUE	TRUE	FALSE	FALSE	TRUE	FALSE	FALSE	0	0
rs581005	chr1	43641314	43641315	1f	0,22271	FALSE	TRUE	FALSE	FALSE	TRUE	FALSE	FALSE	0	0
rs2367724	chr1	43641756	43641757	1b	0,66917	TRUE	TRUE	TRUE	TRUE	TRUE	FALSE	FALSE	0,96	0
rs694197	chr1	43642284	43642285	1f	0,05	FALSE	TRUE	TRUE	TRUE	TRUE	FALSE	FALSE	0,33	0
rs483679	chr1	43642447	43642448	1f	0,55436	TRUE	TRUE	FALSE	FALSE	TRUE	FALSE	FALSE	0	0
rs594293	chr1	43643527	43643528	6	0,74073	FALSE	FALSE	TRUE	FALSE	TRUE	FALSE	FALSE	2	0
rs607296	chr1	43644077	43644078	1b	0,434	TRUE	TRUE	TRUE	TRUE	TRUE	FALSE	FALSE	1,47	0
rs609171	chr1	43644544	43644545	1b	0,57517	TRUE	TRUE	TRUE	TRUE	TRUE	FALSE	FALSE	1,96	0
rs620851	chr1	43644817	43644818	1f	0,55436	TRUE	TRUE	FALSE	FALSE	TRUE	FALSE	FALSE	0	0
rs623965	chr1	43645515	43645516	1f	0,55436	TRUE	TRUE	FALSE	FALSE	TRUE	FALSE	FALSE	0	0
rs72883034	chr1	43646707	43646708	1f	0,55436	TRUE	TRUE	FALSE	FALSE	TRUE	FALSE	FALSE	0	0
rs617521	chr1	43648527	43648528	1f	0,55436	TRUE	TRUE	FALSE	FALSE	TRUE	FALSE	FALSE	0	0
rs519357	chr1	43649350	43649351	1b	0,98667	TRUE	TRUE	TRUE	TRUE	TRUE	FALSE	FALSE	1,6	0
rs61768371	chr1	43652734	43652735	1f	0,55436	TRUE	TRUE	FALSE	FALSE	TRUE	FALSE	FALSE	0	0
rs2274465	chr1	43655885	43655886	1f	0,55324	TRUE	FALSE	FALSE	FALSE	TRUE	FALSE	FALSE	0	0
rs11589584	chr1	43656801	43656802	1f	0,55436	TRUE	TRUE	FALSE	FALSE	TRUE	FALSE	FALSE	0	0
rs509173	chr1	43657830	43657831	1f	0,11667	FALSE	TRUE	TRUE	TRUE	TRUE	FALSE	FALSE	0,64	0
rs559481	chr1	43658194	43658195	6	0,1625	FALSE	FALSE	TRUE	FALSE	TRUE	FALSE	FALSE	0,58	0
rs61768372	chr1	43658316	43658317	1f	0,22271	FALSE	TRUE	FALSE	FALSE	TRUE	FALSE	FALSE	0	0
rs1973512	chr1	43662375	43662376	1b	0,99542	TRUE	TRUE	TRUE	TRUE	TRUE	FALSE	FALSE	1,59	0
rs656131	chr1	43663427	43663428	7	0,51392	FALSE	FALSE	FALSE	FALSE	TRUE	FALSE	FALSE	0	0
rs607062	chr1	43667344	43667345	6	0,79772	FALSE	FALSE	TRUE	TRUE	TRUE	FALSE	FALSE	2	0
rs586339	chr1	43671585	43671586	1b	0,94984	TRUE	TRUE	TRUE	TRUE	TRUE	FALSE	FALSE	0,06	0
rs10789442	chr1	43674403	43674404	6	0,12848	FALSE	FALSE	TRUE	FALSE	TRUE	FALSE	FALSE	0,11	0
rs4660257	chr1	43682496	43682497	7	0,51392	FALSE	FALSE	FALSE	FALSE	TRUE	FALSE	FALSE	0	0
rs4660740	chr1	43682945	43682946	1f	0,22271	FALSE	TRUE	FALSE	FALSE	TRUE	FALSE	FALSE	0	0
rs6697354	chr1	43685343	43685344	1f	0,12	FALSE	TRUE	TRUE	FALSE	TRUE	FALSE	FALSE	1,59	0
rs61768374	chr1	43685961	43685962	7	0,51392	FALSE	FALSE	FALSE	FALSE	TRUE	FALSE	FALSE	0	0
rs113688544	chr1	43686239	43686240	1f	0,05	FALSE	TRUE	TRUE	TRUE	TRUE	FALSE	FALSE	0,94	0
rs1971342	chr1	43686517	43686518	1f	0,22271	FALSE	TRUE	FALSE	FALSE	TRUE	FALSE	FALSE	0	0
rs11805774	chr1	43687043	43687044	1f	0,03154	FALSE	TRUE	TRUE	TRUE	TRUE	FALSE	FALSE	0,1	0
rs17401357	chr1	43687947	43687948	1f	0,22271	FALSE	TRUE	FALSE	FALSE	TRUE	FALSE	FALSE	0	0
rs3791035	chr1	43688807	43688808	1f	0,55436	TRUE	TRUE	FALSE	FALSE	TRUE	FALSE	FALSE	0	0
rs3791036	chr1	43689711	43689712	1f	0,1	FALSE	TRUE	TRUE	TRUE	TRUE	FALSE	FALSE	0,75	0
rs2270972	chr1	43692457	43692458	1f	0,55436	TRUE	TRUE	FALSE	FALSE	TRUE	FALSE	FALSE	0	0
rs56319043	chr1	43705539	43705540	1b	0,97205	TRUE	TRUE	TRUE	TRUE	TRUE	FALSE	FALSE	0,07	0

rs112984125	chr1	43707751	43707752	1f	0,55436	TRUE	TRUE	FALSE	FALSE	TRUE	FALSE	FALSE	0	0
rs304303	chr1	43712398	43712399	1f	0,41667	TRUE	FALSE	TRUE	TRUE	TRUE	FALSE	FALSE	1,47	0
rs17531412	chr1	43716572	43716573	1f	0,55436	TRUE	TRUE	FALSE	FALSE	TRUE	FALSE	FALSE	0	0
rs6429636	chr1	43717868	43717869	7	0,51392	FALSE	FALSE	FALSE	FALSE	TRUE	FALSE	FALSE	0	0
rs112361411	chr1	43718251	43718252	1f	0,55436	TRUE	TRUE	FALSE	FALSE	TRUE	FALSE	FALSE	0	0
rs10890273	chr1	43720772	43720773	1f	0,22271	FALSE	TRUE	FALSE	FALSE	TRUE	FALSE	FALSE	0	0
rs11810109	chr1	43721140	43721141	1f	0,67917	TRUE	FALSE	TRUE	FALSE	TRUE	FALSE	FALSE	1,9	0
rs12410155	chr1	43722793	43722794	1f	0,55436	TRUE	TRUE	FALSE	FALSE	TRUE	FALSE	FALSE	0	0
rs12410444	chr1	43723047	43723048	1f	0,66703	TRUE	TRUE	FALSE	TRUE	TRUE	FALSE	FALSE	0	0
rs11210907	chr1	43726087	43726088	7	0,51392	FALSE	FALSE	FALSE	FALSE	TRUE	FALSE	FALSE	0	0
rs10890275	chr1	43728544	43728545	7	0,51392	FALSE	FALSE	FALSE	FALSE	TRUE	FALSE	FALSE	0	0
rs4660259	chr1	43728862	43728863	7	0,18412	FALSE	FALSE	FALSE	FALSE	FALSE	FALSE	FALSE	0	0
rs4660260	chr1	43729681	43729682	1f	0,83785	TRUE	FALSE	TRUE	FALSE	TRUE	FALSE	FALSE	1,99	0
rs4660743	chr1	43729732	43729733	1f	0,55324	TRUE	FALSE	FALSE	FALSE	TRUE	FALSE	FALSE	0	0
rs3862228	chr1	43731273	43731274	7	0,51392	FALSE	FALSE	FALSE	FALSE	TRUE	FALSE	FALSE	0	0
rs3791041	chr1	43737319	43737320	1f	0,22271	FALSE	TRUE	FALSE	FALSE	TRUE	FALSE	FALSE	0	0
rs10749850	chr1	43739422	43739423	7	0,51392	FALSE	FALSE	FALSE	FALSE	TRUE	FALSE	FALSE	0	0
rs6686238	chr1	43744581	43744582	1f	0,83785	TRUE	FALSE	TRUE	FALSE	TRUE	FALSE	FALSE	1,99	0
rs6429638	chr1	43745338	43745339	1f	0,55436	TRUE	TRUE	FALSE	FALSE	TRUE	FALSE	FALSE	0	0
rs2158956	chr1	43746025	43746026	6	0,17686	FALSE	FALSE	TRUE	FALSE	TRUE	FALSE	FALSE	0,57	0
rs28833034	chr1	43747270	43747271	1f	0,55324	TRUE	FALSE	FALSE	FALSE	TRUE	FALSE	FALSE	0	0
rs7549094	chr1	43747506	43747507	1f	0,55324	TRUE	FALSE	FALSE	FALSE	TRUE	FALSE	FALSE	0	0
rs6669157	chr1	43751204	43751205	6	0,80877	FALSE	FALSE	TRUE	FALSE	TRUE	FALSE	FALSE	1,99	0
rs3791042	chr1	43753603	43753604	1f	0,55436	TRUE	TRUE	FALSE	FALSE	TRUE	FALSE	FALSE	0	0
rs11210913	chr1	43756925	43756926	7	0,51392	FALSE	FALSE	FALSE	FALSE	TRUE	FALSE	FALSE	0	0
rs4660261	chr1	43763925	43763926	1f	0,55436	TRUE	TRUE	FALSE	FALSE	TRUE	FALSE	FALSE	0	0
rs12119149	chr1	43777611	43777612	1f	0,34417	TRUE	FALSE	TRUE	FALSE	TRUE	FALSE	FALSE	0,22	0
rs7543520	chr1	43778194	43778195	1f	0,55324	TRUE	FALSE	FALSE	FALSE	TRUE	FALSE	FALSE	0	0
rs7546040	chr1	43781561	43781562	1b	0,98042	TRUE	TRUE	TRUE	TRUE	TRUE	FALSE	FALSE	1,55	0
rs917294	chr1	43783813	43783814	1f	0,55436	TRUE	TRUE	FALSE	FALSE	TRUE	FALSE	FALSE	0	0
rs3011216	chr1	43830766	43830767	1f	0,55436	TRUE	TRUE	FALSE	FALSE	TRUE	FALSE	FALSE	0	0
rs37452	chr1	43831900	43831901	1f	0,27	FALSE	TRUE	TRUE	FALSE	TRUE	FALSE	FALSE	1,27	0
rs3011217	chr1	43837593	43837594	1b	0,95833	TRUE	TRUE	TRUE	TRUE	TRUE	FALSE	FALSE	1,97	0
rs3011219	chr1	43840447	43840448	1f	0,22271	FALSE	TRUE	FALSE	FALSE	TRUE	FALSE	FALSE	0	0
rs2906471	chr1	43840749	43840750	7	0,51392	FALSE	FALSE	FALSE	FALSE	TRUE	FALSE	FALSE	0	0
rs3011220	chr1	43843194	43843195	7	0,51392	FALSE	FALSE	FALSE	FALSE	TRUE	FALSE	FALSE	0	0
rs12723279	chr1	43845071	43845072	1f	0,83667	TRUE	FALSE	TRUE	TRUE	TRUE	FALSE	FALSE	1,9	0
rs11210931	chr1	43845202	43845203	1b	0,434	TRUE	TRUE	TRUE	TRUE	TRUE	FALSE	FALSE	1,47	0
rs2367804	chr1	43848593	43848594	1f	0,22271	FALSE	TRUE	FALSE	FALSE	TRUE	FALSE	FALSE	0	0
rs2906468	chr1	43853334	43853335	1f	0,55436	TRUE	TRUE	FALSE	FALSE	TRUE	FALSE	FALSE	0	0
rs3011225	chr1	43853700	43853701	1b	0,8575	TRUE	TRUE	TRUE	TRUE	TRUE	FALSE	FALSE	0,15	0
rs2906457	chr1	43872902	43872903	1f	0,55436	TRUE	TRUE	FALSE	FALSE	TRUE	FALSE	FALSE	0	0
rs2527776	chr1	43879619	43879620	1b	0,50217	TRUE	TRUE	TRUE	TRUE	TRUE	FALSE	FALSE	1,87	0
rs803679	chr1	43883732	43883733	1f	0,55436	TRUE	TRUE	FALSE	FALSE	TRUE	FALSE	FALSE	0	0
rs803678	chr1	43885225	43885226	1f	0,55436	TRUE	TRUE	FALSE	FALSE	TRUE	FALSE	FALSE	0	0
rs803675	chr1	43887564	43887565	1d	0,29222	TRUE	TRUE	TRUE	FALSE	TRUE	FALSE	FALSE	1,72	0
rs803682	chr1	43896950	43896951	7	0,51392	FALSE	FALSE	FALSE	FALSE	TRUE	FALSE	FALSE	0	0
rs3791101	chr1	43900577	43900578	1f	0,22271	FALSE	TRUE	FALSE	FALSE	TRUE	FALSE	FALSE	0	0
rs11210934	chr1	43903762	43903763	1f	0,22271	FALSE	TRUE	FALSE	FALSE	TRUE	FALSE	FALSE	0	0
rs11210935	chr1	43903771	43903772	1f	0,22271	FALSE	TRUE	FALSE	FALSE	TRUE	FALSE	FALSE	0	0
rs113551349	chr1	44009450	44009451	1e	0,82214	TRUE	FALSE	TRUE	TRUE	TRUE	TRUE	FALSE	1,65	0,05

rs2582911	chr11	28504005	28504006	7	0,51392	FALSE	FALSE	FALSE	FALSE	TRUE	FALSE	FALSE	0	0
rs2585807	chr11	28511110	28511111	1f	0,22271	FALSE	TRUE	FALSE	FALSE	TRUE	FALSE	FALSE	0	0
rs4922805	chr11	28521550	28521551	1f	0,16333	FALSE	TRUE	TRUE	TRUE	TRUE	FALSE	FALSE	1,89	0
rs2582915	chr11	28522264	28522265	1f	0,22271	FALSE	TRUE	FALSE	FALSE	TRUE	FALSE	FALSE	0	0
rs7935201	chr11	28526195	28526196	1f	0,55436	TRUE	TRUE	FALSE	FALSE	TRUE	FALSE	FALSE	0	0
rs990845	chr11	28526681	28526682	1b	0,97205	TRUE	TRUE	TRUE	TRUE	TRUE	FALSE	FALSE	0,07	0
rs990846	chr11	28526860	28526861	1f	0,55324	TRUE	FALSE	FALSE	FALSE	TRUE	FALSE	FALSE	0	0
rs2582894	chr11	28554713	28554714	7	0,51392	FALSE	FALSE	FALSE	FALSE	TRUE	FALSE	FALSE	0	0
rs11030380	chr11	28569620	28569621	5	0,13454	FALSE	TRUE	FALSE	FALSE	FALSE	FALSE	FALSE	0	0
rs7127383	chr11	28570039	28570040	7	0,18412	FALSE	FALSE	FALSE	FALSE	FALSE	FALSE	FALSE	0	0
rs11030381	chr11	28578332	28578333	7	0,18412	FALSE	FALSE	FALSE	FALSE	FALSE	FALSE	FALSE	0	0
rs2582895	chr11	28580625	28580626	6	0,36297	FALSE	FALSE	TRUE	FALSE	FALSE	FALSE	FALSE	1,39	0
rs2585817	chr11	28580673	28580674	7	0,18412	FALSE	FALSE	FALSE	FALSE	FALSE	FALSE	FALSE	0	0
rs7128734	chr11	28582121	28582122	1f	0,55436	TRUE	TRUE	FALSE	FALSE	TRUE	FALSE	FALSE	0	0
rs11030382	chr11	28582929	28582930	5	0,58955	TRUE	FALSE	FALSE	FALSE	FALSE	FALSE	FALSE	0	0
rs10835360	chr11	28585151	28585152	5	0	FALSE	TRUE	TRUE	TRUE	FALSE	FALSE	FALSE	0,01	0
rs7952220	chr11	28585697	28585698	5	0,13454	FALSE	TRUE	FALSE	FALSE	FALSE	FALSE	FALSE	0	0
rs4923546	chr11	28586226	28586227	5	0,58955	TRUE	FALSE	FALSE	FALSE	FALSE	FALSE	FALSE	0	0
rs4378371	chr11	28587182	28587183	6	0,39217	FALSE	FALSE	TRUE	FALSE	FALSE	FALSE	FALSE	0,73	0
rs4520584	chr11	28590844	28590845	5	0,13454	FALSE	TRUE	FALSE	FALSE	FALSE	FALSE	FALSE	0	0
rs2585813	chr11	28592942	28592943	5	0,58955	TRUE	FALSE	FALSE	FALSE	FALSE	FALSE	FALSE	0	0
rs2585814	chr11	28593836	28593837	7	0,18412	FALSE	FALSE	FALSE	FALSE	FALSE	FALSE	FALSE	0	0
rs2582896	chr11	28594151	28594152	7	0,18412	FALSE	FALSE	FALSE	FALSE	FALSE	FALSE	FALSE	0	0
rs2582897	chr11	28594152	28594153	7	0,18412	FALSE	FALSE	FALSE	FALSE	FALSE	FALSE	FALSE	0	0
rs10742196	chr11	28594703	28594704	4	0,60906	TRUE	TRUE	FALSE	FALSE	FALSE	FALSE	FALSE	0	0
rs4244537	chr11	28596074	28596075	7	0,18412	FALSE	FALSE	FALSE	FALSE	FALSE	FALSE	FALSE	0	0
rs10835361	chr11	28596495	28596496	7	0,18412	FALSE	FALSE	FALSE	FALSE	FALSE	FALSE	FALSE	0	0
rs2585811	chr11	28596863	28596864	7	0,18412	FALSE	FALSE	FALSE	FALSE	FALSE	FALSE	FALSE	0	0
rs10767727	chr11	28597208	28597209	5	0,13454	FALSE	TRUE	FALSE	FALSE	FALSE	FALSE	FALSE	0	0
rs7931440	chr11	28602325	28602326	7	0,18412	FALSE	FALSE	FALSE	FALSE	FALSE	FALSE	FALSE	0	0
rs11030384	chr11	28603809	28603810	7	0,18412	FALSE	FALSE	FALSE	FALSE	FALSE	FALSE	FALSE	0	0
rs4442537	chr11	28604116	28604117	6	0,07634	FALSE	FALSE	TRUE	TRUE	TRUE	FALSE	FALSE	0	0
rs10835362	chr11	28605238	28605239	6	0,17046	FALSE	FALSE	TRUE	FALSE	FALSE	FALSE	FALSE	1,59	0
rs10835363	chr11	28605956	28605957	7	0,18412	FALSE	FALSE	FALSE	FALSE	FALSE	FALSE	FALSE	0	0
rs10835364	chr11	28606105	28606106	7	0,18412	FALSE	FALSE	FALSE	FALSE	FALSE	FALSE	FALSE	0	0
rs57806515	chr11	28607001	28607002	4	0,60906	TRUE	TRUE	FALSE	FALSE	FALSE	FALSE	FALSE	0	0
rs11030385	chr11	28607567	28607568	5	0,13454	FALSE	TRUE	FALSE	FALSE	FALSE	FALSE	FALSE	0	0
rs10835365	chr11	28608503	28608504	7	0,18412	FALSE	FALSE	FALSE	FALSE	FALSE	FALSE	FALSE	0	0
rs11821132	chr11	28609186	28609187	7	0,18412	FALSE	FALSE	FALSE	FALSE	FALSE	FALSE	FALSE	0	0
rs59617050	chr11	28609198	28609199	6	0,073	FALSE	FALSE	TRUE	FALSE	FALSE	FALSE	FALSE	0,63	0
rs12226610	chr11	28609521	28609522	5	0,58955	TRUE	FALSE	FALSE	FALSE	FALSE	FALSE	FALSE	0	0
rs11030386	chr11	28609700	28609701	5	0,58955	TRUE	FALSE	FALSE	FALSE	FALSE	FALSE	FALSE	0	0
rs11030387	chr11	28609936	28609937	5	0,58955	TRUE	FALSE	FALSE	FALSE	FALSE	FALSE	FALSE	0	0
rs10767729	chr11	28613203	28613204	5	0,13454	FALSE	TRUE	FALSE	FALSE	FALSE	FALSE	FALSE	0	0
rs10767730	chr11	28613314	28613315	7	0,18412	FALSE	FALSE	FALSE	FALSE	FALSE	FALSE	FALSE	0	0
rs10767731	chr11	28613315	28613316	7	0,18412	FALSE	FALSE	FALSE	FALSE	FALSE	FALSE	FALSE	0	0
rs7110556	chr11	28614381	28614382	7	0,18412	FALSE	FALSE	FALSE	FALSE	FALSE	FALSE	FALSE	0	0
rs4923549	chr11	28615788	28615789	4	0,70497	TRUE	TRUE	FALSE	TRUE	FALSE	FALSE	FALSE	0	0
rs4923550	chr11	28615815	28615816	2b	0,55744	TRUE	TRUE	TRUE	TRUE	FALSE	FALSE	FALSE	1,5	0
rs12221661	chr11	28617582	28617583	5	0,13454	FALSE	TRUE	FALSE	FALSE	FALSE	FALSE	FALSE	0	0
rs12226518	chr11	28619514	28619515	7	0,18412	FALSE	FALSE	FALSE	FALSE	FALSE	FALSE	FALSE	0	0

rs12226542	chr11	28619697	28619698	5	0,58955	TRUE	FALSE	FALSE	FALSE	FALSE	FALSE	FALSE	0	0
rs10767732	chr11	28620720	28620721	1f	0,22271	FALSE	TRUE	FALSE	FALSE	TRUE	FALSE	FALSE	0	0
rs10767733	chr11	28620772	28620773	5	0,13454	FALSE	TRUE	FALSE	FALSE	FALSE	FALSE	FALSE	0	0
rs10767734	chr11	28620833	28620834	5	0,13454	FALSE	TRUE	FALSE	FALSE	FALSE	FALSE	FALSE	0	0
rs10767735	chr11	28621013	28621014	5	0,13454	FALSE	TRUE	FALSE	FALSE	FALSE	FALSE	FALSE	0	0
rs4636654	chr11	28621105	28621106	4	0,60906	TRUE	TRUE	FALSE	FALSE	FALSE	FALSE	FALSE	0	0
rs10835368	chr11	28621751	28621752	5	0,07931	FALSE	TRUE	TRUE	TRUE	FALSE	FALSE	FALSE	1,93	0
rs10742197	chr11	28622319	28622320	1f	0,22271	FALSE	TRUE	FALSE	FALSE	TRUE	FALSE	FALSE	0	0
rs10835371	chr11	28622329	28622330	5	0,13454	FALSE	TRUE	FALSE	FALSE	FALSE	FALSE	FALSE	0	0
rs10835372	chr11	28622365	28622366	5	0,13454	FALSE	TRUE	FALSE	FALSE	FALSE	FALSE	FALSE	0	0
rs10835373	chr11	28622394	28622395	5	0,74944	FALSE	TRUE	TRUE	TRUE	FALSE	FALSE	FALSE	2	0
rs10835374	chr11	28622423	28622424	5	0,13454	FALSE	TRUE	FALSE	FALSE	FALSE	FALSE	FALSE	0	0
rs10835375	chr11	28622479	28622480	5	0,13454	FALSE	TRUE	FALSE	FALSE	FALSE	FALSE	FALSE	0	0
rs10767736	chr11	28622490	28622491	5	0,13454	FALSE	TRUE	FALSE	FALSE	FALSE	FALSE	FALSE	0	0
rs11030388	chr11	28622564	28622565	5	0,13454	FALSE	TRUE	FALSE	FALSE	FALSE	FALSE	FALSE	0	0
rs73434729	chr11	28622744	28622745	7	0,18412	FALSE	FALSE	FALSE	FALSE	FALSE	FALSE	FALSE	0	0
rs12576604	chr11	28623054	28623055	5	0,07931	FALSE	TRUE	TRUE	TRUE	FALSE	FALSE	FALSE	1,93	0
rs11030391	chr11	28623078	28623079	6	0,025	FALSE	FALSE	TRUE	TRUE	FALSE	FALSE	FALSE	0,64	0
rs10835381	chr11	28623282	28623283	6	0,36297	FALSE	FALSE	TRUE	FALSE	FALSE	FALSE	FALSE	1,39	0
rs10835382	chr11	28623337	28623338	7	0,18412	FALSE	FALSE	FALSE	FALSE	FALSE	FALSE	FALSE	0	0
rs4268495	chr11	28623793	28623794	7	0,18412	FALSE	FALSE	FALSE	FALSE	FALSE	FALSE	FALSE	0	0
rs4267058	chr11	28624036	28624037	7	0,18412	FALSE	FALSE	FALSE	FALSE	FALSE	FALSE	FALSE	0	0
rs4274191	chr11	28624150	28624151	7	0,18412	FALSE	FALSE	FALSE	FALSE	FALSE	FALSE	FALSE	0	0
rs10767737	chr11	28624271	28624272	7	0,18412	FALSE	FALSE	FALSE	FALSE	FALSE	FALSE	FALSE	0	0
rs4576808	chr11	28624924	28624925	7	0,18412	FALSE	FALSE	FALSE	FALSE	FALSE	FALSE	FALSE	0	0
rs4351802	chr11	28625063	28625064	4	0,60906	TRUE	TRUE	FALSE	FALSE	FALSE	FALSE	FALSE	0	0
rs4434991	chr11	28625608	28625609	4	0,70497	TRUE	TRUE	FALSE	TRUE	FALSE	FALSE	FALSE	0	0
rs4290215	chr11	28626407	28626408	5	0,23589	FALSE	TRUE	FALSE	TRUE	FALSE	FALSE	FALSE	0	0
rs7931459	chr11	28626637	28626638	5	0,13454	FALSE	TRUE	FALSE	FALSE	FALSE	FALSE	FALSE	0	0
rs7931585	chr11	28626782	28626783	7	0,18412	FALSE	FALSE	FALSE	FALSE	FALSE	FALSE	FALSE	0	0
rs7931884	chr11	28626830	28626831	7	0,18412	FALSE	FALSE	FALSE	FALSE	FALSE	FALSE	FALSE	0	0
rs11030392	chr11	28627123	28627124	7	0,18412	FALSE	FALSE	FALSE	FALSE	FALSE	FALSE	FALSE	0	0
rs11030393	chr11	28627189	28627190	7	0,18412	FALSE	FALSE	FALSE	FALSE	FALSE	FALSE	FALSE	0	0
rs7121736	chr11	28628322	28628323	1f	0,55324	TRUE	FALSE	FALSE	FALSE	TRUE	FALSE	FALSE	0	0
rs4471400	chr11	28628548	28628549	4	0,60906	TRUE	TRUE	FALSE	FALSE	FALSE	FALSE	FALSE	0	0
rs4575255	chr11	28628634	28628635	4	0,70497	TRUE	TRUE	FALSE	TRUE	FALSE	FALSE	FALSE	0	0
rs4350350	chr11	28628858	28628859	4	0,60906	TRUE	TRUE	FALSE	FALSE	FALSE	FALSE	FALSE	0	0
rs4568992	chr11	28628988	28628989	5	0,13454	FALSE	TRUE	FALSE	FALSE	FALSE	FALSE	FALSE	0	0
rs6484376	chr11	28629362	28629363	5	0,58955	TRUE	FALSE	FALSE	FALSE	FALSE	FALSE	FALSE	0	0
rs10835383	chr11	28629810	28629811	7	0,18412	FALSE	FALSE	FALSE	FALSE	FALSE	FALSE	FALSE	0	0
rs7951682	chr11	28630346	28630347	7	0,51392	FALSE	FALSE	FALSE	FALSE	TRUE	FALSE	FALSE	0	0
rs4275621	chr11	28631448	28631449	5	0,13454	FALSE	TRUE	FALSE	FALSE	FALSE	FALSE	FALSE	0	0
rs6484377	chr11	28631940	28631941	5	0,13454	FALSE	TRUE	FALSE	FALSE	FALSE	FALSE	FALSE	0	0
rs7124523	chr11	28632082	28632083	4	0,60906	TRUE	TRUE	FALSE	FALSE	FALSE	FALSE	FALSE	0	0
rs7124653	chr11	28632179	28632180	4	0,70497	TRUE	TRUE	FALSE	TRUE	FALSE	FALSE	FALSE	0	0
rs4290216	chr11	28632478	28632479	4	0,70497	TRUE	TRUE	FALSE	TRUE	FALSE	FALSE	FALSE	0	0
rs12282355	chr11	28632739	28632740	3a	0,69529	TRUE	TRUE	TRUE	FALSE	FALSE	FALSE	FALSE	1,54	0
rs10835384	chr11	28632993	28632994	4	0,60906	TRUE	TRUE	FALSE	FALSE	FALSE	FALSE	FALSE	0	0
rs7935241	chr11	28633320	28633321	5	0,15502	FALSE	TRUE	TRUE	TRUE	FALSE	FALSE	FALSE	1,76	0
rs7938026	chr11	28633510	28633511	5	0,23589	FALSE	TRUE	FALSE	TRUE	FALSE	FALSE	FALSE	0	0
rs4244538	chr11	28634124	28634125	4	0,60906	TRUE	TRUE	FALSE	FALSE	FALSE	FALSE	FALSE	0	0

rs6484378	chr11	28634350	28634351	5	0,13454	FALSE	TRUE	FALSE	FALSE	FALSE	FALSE	FALSE	0	0
rs7928893	chr11	28634461	28634462	5	0,32893	FALSE	TRUE	TRUE	TRUE	FALSE	FALSE	FALSE	1,83	0
rs7942078	chr11	28634516	28634517	5	0,13454	FALSE	TRUE	FALSE	FALSE	FALSE	FALSE	FALSE	0	0
rs7942417	chr11	28634686	28634687	4	0,60906	TRUE	TRUE	FALSE	FALSE	FALSE	FALSE	FALSE	0	0
rs4344470	chr11	28635702	28635703	5	0	FALSE	TRUE	TRUE	FALSE	FALSE	FALSE	FALSE	1,01	0
rs12577364	chr11	28636482	28636483	1f	0,55436	TRUE	TRUE	FALSE	FALSE	TRUE	FALSE	FALSE	0	0
rs6484379	chr11	28637409	28637410	7	0,18412	FALSE	FALSE	FALSE	FALSE	FALSE	FALSE	FALSE	0	0
rs3935074	chr11	28638054	28638055	7	0,18412	FALSE	FALSE	FALSE	FALSE	FALSE	FALSE	FALSE	0	0
rs4337023	chr11	28638657	28638658	5	0,13454	FALSE	TRUE	FALSE	FALSE	FALSE	FALSE	FALSE	0	0
rs7937781	chr11	28639270	28639271	5	0,58955	TRUE	FALSE	FALSE	FALSE	FALSE	FALSE	FALSE	0	0
rs7938122	chr11	28639314	28639315	5	0,58955	TRUE	FALSE	FALSE	FALSE	FALSE	FALSE	FALSE	0	0
rs11030397	chr11	28644511	28644512	7	0,18412	FALSE	FALSE	FALSE	FALSE	FALSE	FALSE	FALSE	0	0
rs11030398	chr11	28644516	28644517	6	0,00889	FALSE	FALSE	TRUE	FALSE	FALSE	FALSE	FALSE	0,02	0
rs11030399	chr11	28644792	28644793	7	0,18412	FALSE	FALSE	FALSE	FALSE	FALSE	FALSE	FALSE	0	0
rs7933262	chr11	28644990	28644991	7	0,18412	FALSE	FALSE	FALSE	FALSE	FALSE	FALSE	FALSE	0	0
rs7130649	chr11	28645541	28645542	7	0,18412	FALSE	FALSE	FALSE	FALSE	FALSE	FALSE	FALSE	0	0
rs2883826	chr11	28646123	28646124	7	0,18412	FALSE	FALSE	FALSE	FALSE	FALSE	FALSE	FALSE	0	0
rs4534569	chr11	28646268	28646269	6	0,41186	FALSE	FALSE	TRUE	FALSE	FALSE	FALSE	FALSE	0,31	0
rs4372430	chr11	28646392	28646393	7	0,18412	FALSE	FALSE	FALSE	FALSE	FALSE	FALSE	FALSE	0	0
rs7481939	chr11	28646736	28646737	5	0,58955	TRUE	FALSE	FALSE	FALSE	FALSE	FALSE	FALSE	0	0
rs6484385	chr11	28646868	28646869	5	0,58955	TRUE	FALSE	FALSE	FALSE	FALSE	FALSE	FALSE	0	0
rs7942414	chr11	28648416	28648417	5	0,13454	FALSE	TRUE	FALSE	FALSE	FALSE	FALSE	FALSE	0	0
rs6484386	chr11	28648569	28648570	7	0,18412	FALSE	FALSE	FALSE	FALSE	FALSE	FALSE	FALSE	0	0
rs6484387	chr11	28648574	28648575	7	0,18412	FALSE	FALSE	FALSE	FALSE	FALSE	FALSE	FALSE	0	0
rs7105480	chr11	28648921	28648922	5	0,13454	FALSE	TRUE	FALSE	FALSE	FALSE	FALSE	FALSE	0	0
rs2356016	chr11	28649221	28649222	5	0,13454	FALSE	TRUE	FALSE	FALSE	FALSE	FALSE	FALSE	0	0
rs2356015	chr11	28649586	28649587	5	0,13454	FALSE	TRUE	FALSE	FALSE	FALSE	FALSE	FALSE	0	0
rs2356014	chr11	28649652	28649653	5	0,13454	FALSE	TRUE	FALSE	FALSE	FALSE	FALSE	FALSE	0	0
rs10835387	chr11	28650081	28650082	2b	1	TRUE	TRUE	TRUE	TRUE	FALSE	FALSE	FALSE	1,42	0
rs10835388	chr11	28650802	28650803	7	0,18412	FALSE	FALSE	FALSE	FALSE	FALSE	FALSE	FALSE	0	0
rs11030401	chr11	28654591	28654592	5	0,58955	TRUE	FALSE	FALSE	FALSE	FALSE	FALSE	FALSE	0	0
rs11030402	chr11	28654622	28654623	6	0,00167	FALSE	FALSE	TRUE	FALSE	FALSE	FALSE	FALSE	0,27	0
rs10835389	chr11	28654957	28654958	7	0,18412	FALSE	FALSE	FALSE	FALSE	FALSE	FALSE	FALSE	0	0
rs4923552	chr11	28655189	28655190	6	0,31452	FALSE	FALSE	TRUE	FALSE	FALSE	FALSE	FALSE	1,78	0
rs4923553	chr11	28655214	28655215	7	0,18412	FALSE	FALSE	FALSE	FALSE	FALSE	FALSE	FALSE	0	0
rs4923554	chr11	28655290	28655291	6	0,08912	FALSE	FALSE	TRUE	FALSE	FALSE	FALSE	FALSE	1,58	0
rs4923555	chr11	28655313	28655314	7	0,18412	FALSE	FALSE	FALSE	FALSE	FALSE	FALSE	FALSE	0	0
rs7942482	chr11	28655716	28655717	7	0,18412	FALSE	FALSE	FALSE	FALSE	FALSE	FALSE	FALSE	0	0
rs10767739	chr11	28657322	28657323	4	0,60906	TRUE	TRUE	FALSE	FALSE	FALSE	FALSE	FALSE	0	0
rs11030403	chr11	28658308	28658309	4	0,60906	TRUE	TRUE	FALSE	FALSE	FALSE	FALSE	FALSE	0	0
rs2356013	chr11	28658595	28658596	4	0,60906	TRUE	TRUE	FALSE	FALSE	FALSE	FALSE	FALSE	0	0
rs7947218	chr11	28659175	28659176	5	0,58955	TRUE	FALSE	FALSE	FALSE	FALSE	FALSE	FALSE	0	0
rs7934432	chr11	28662673	28662674	4	0,60906	TRUE	TRUE	FALSE	FALSE	FALSE	FALSE	FALSE	0	0
rs2356009	chr11	28664504	28664505	5	0,58955	TRUE	FALSE	FALSE	FALSE	FALSE	FALSE	FALSE	0	0
rs4923556	chr11	28666218	28666219	5	0,13454	FALSE	TRUE	FALSE	FALSE	FALSE	FALSE	FALSE	0	0
rs7129601	chr11	28666859	28666860	2b	0,739	TRUE	TRUE	TRUE	TRUE	FALSE	FALSE	FALSE	1,57	0
rs4579914	chr11	28668804	28668805	6	0,35072	FALSE	FALSE	TRUE	FALSE	FALSE	FALSE	FALSE	0,06	0
rs10767740	chr11	28669586	28669587	7	0,18412	FALSE	FALSE	FALSE	FALSE	FALSE	FALSE	FALSE	0	0
rs7119404	chr11	28669739	28669740	6	0,14464	FALSE	FALSE	TRUE	FALSE	FALSE	FALSE	FALSE	0,23	0
rs4923557	chr11	28671983	28671984	7	0,18412	FALSE	FALSE	FALSE	FALSE	FALSE	FALSE	FALSE	0	0
rs10835391	chr11	28672892	28672893	7	0,18412	FALSE	FALSE	FALSE	FALSE	FALSE	FALSE	FALSE	0	0

rs10506971	chr12	89364159	89364160	1f	0,55436	TRUE	TRUE	FALSE	FALSE	TRUE	FALSE	FALSE	0	0
rs1427829	chr12	89366966	89366967	1f	0,55436	TRUE	TRUE	FALSE	FALSE	TRUE	FALSE	FALSE	0	0
rs1427828	chr12	89368721	89368722	1f	0,66703	TRUE	TRUE	FALSE	TRUE	TRUE	FALSE	FALSE	0	0
rs12819667	chr12	89369751	89369752	1f	0,55324	TRUE	FALSE	FALSE	FALSE	TRUE	FALSE	FALSE	0	0
rs796374	chr12	89377734	89377735	1f	0,55324	TRUE	FALSE	FALSE	FALSE	TRUE	FALSE	FALSE	0	0
rs704061	chr12	89378125	89378126	1f	0,22271	FALSE	TRUE	FALSE	FALSE	TRUE	FALSE	FALSE	0	0
rs10506965	chr12	89381251	89381252	1f	0,55436	TRUE	TRUE	FALSE	FALSE	TRUE	FALSE	FALSE	0	0
rs770083	chr12	89382506	89382507	1f	0,55436	TRUE	TRUE	FALSE	FALSE	TRUE	FALSE	FALSE	0	0
rs76284431	chr14	98224585	98224586	7	0,18412	FALSE	FALSE	FALSE	FALSE	FALSE	FALSE	FALSE	0	0
rs12589260	chr14	98239062	98239063	7	0,18412	FALSE	FALSE	FALSE	FALSE	FALSE	FALSE	FALSE	0	0
rs78745380	chr14	98244156	98244157	3a	0,9865	TRUE	TRUE	TRUE	FALSE	FALSE	FALSE	FALSE	1,04	0
rs12590023	chr14	98253980	98253981	7	0,18412	FALSE	FALSE	FALSE	FALSE	FALSE	FALSE	FALSE	0	0
rs74469887	chr14	98255845	98255846	5	0	FALSE	TRUE	TRUE	TRUE	FALSE	FALSE	FALSE	1,56	0
rs1954554	chr14	98259645	98259646	7	0,18412	FALSE	FALSE	FALSE	FALSE	FALSE	FALSE	FALSE	0	0
rs4131786	chr16	61746653	61746654	2b	0,62064	TRUE	TRUE	TRUE	TRUE	FALSE	FALSE	FALSE	1,94	0
rs7206338	chr16	61749669	61749670	7	0,18412	FALSE	FALSE	FALSE	FALSE	FALSE	FALSE	FALSE	0	0
rs8061672	chr16	61753222	61753223	6	0,20829	FALSE	FALSE	TRUE	FALSE	FALSE	FALSE	FALSE	0,09	0
rs7187101	chr16	61754061	61754062	7	0,18412	FALSE	FALSE	FALSE	FALSE	FALSE	FALSE	FALSE	0	0
rs7192912	chr16	61754667	61754668	6	0,67385	FALSE	FALSE	TRUE	FALSE	FALSE	FALSE	FALSE	1,98	0
rs9931582	chr16	61755911	61755912	5	0,13454	FALSE	TRUE	FALSE	FALSE	FALSE	FALSE	FALSE	0	0
rs7202312	chr16	61758376	61758377	7	0,18412	FALSE	FALSE	FALSE	FALSE	FALSE	FALSE	FALSE	0	0
rs7196175	chr16	61781741	61781742	7	0,18412	FALSE	FALSE	FALSE	FALSE	FALSE	FALSE	FALSE	0	0
rs1438898	chr2	144956786	144956787	7	0,18412	FALSE	FALSE	FALSE	FALSE	FALSE	FALSE	FALSE	0	0
rs72854462	chr2	144962571	144962572	5	0,58955	TRUE	FALSE	FALSE	FALSE	FALSE	FALSE	FALSE	0	0
rs6082289	chr20	21038663	21038664	6	0,21138	FALSE	FALSE	TRUE	FALSE	TRUE	FALSE	FALSE	0	0
rs6047225	chr20	21073854	21073855	6	0,20689	FALSE	FALSE	TRUE	FALSE	TRUE	FALSE	FALSE	0,07	0
rs6035772	chr20	21074403	21074404	7	0,51392	FALSE	FALSE	FALSE	FALSE	TRUE	FALSE	FALSE	0	0
rs6047268	chr20	21138466	21138467	7	0,51392	FALSE	FALSE	FALSE	FALSE	TRUE	FALSE	FALSE	0	0
rs2328607	chr20	21140566	21140567	1f	0,55324	TRUE	FALSE	FALSE	FALSE	TRUE	FALSE	FALSE	0	0
rs6047269	chr20	21141037	21141038	5	1	TRUE	FALSE	TRUE	FALSE	FALSE	FALSE	FALSE	1,78	0
rs4815022	chr20	21144230	21144231	7	0,51392	FALSE	FALSE	FALSE	FALSE	TRUE	FALSE	FALSE	0	0
rs3762194	chr20	21146081	21146082	1f	0,22271	FALSE	TRUE	FALSE	FALSE	TRUE	FALSE	FALSE	0	0
rs6047273	chr20	21154157	21154158	7	0,51392	FALSE	FALSE	FALSE	FALSE	TRUE	FALSE	FALSE	0	0
rs6035790	chr20	21154955	21154956	1f	0,22271	FALSE	TRUE	FALSE	FALSE	TRUE	FALSE	FALSE	0	0
rs6035791	chr20	21155076	21155077	7	0,51392	FALSE	FALSE	FALSE	FALSE	TRUE	FALSE	FALSE	0	0
rs6047277	chr20	21156916	21156917	1f	0,55436	TRUE	TRUE	FALSE	FALSE	TRUE	FALSE	FALSE	0	0
rs4815024	chr20	21157725	21157726	1f	0,03	FALSE	TRUE	TRUE	TRUE	TRUE	FALSE	FALSE	1,72	0
rs6035792	chr20	21158347	21158348	1f	0,22271	FALSE	TRUE	FALSE	FALSE	TRUE	FALSE	FALSE	0	0
rs6035793	chr20	21158610	21158611	7	0,51392	FALSE	FALSE	FALSE	FALSE	TRUE	FALSE	FALSE	0	0
rs6047278	chr20	21158740	21158741	7	0,51392	FALSE	FALSE	FALSE	FALSE	TRUE	FALSE	FALSE	0	0
rs721785	chr20	21159529	21159530	1f	0,22271	FALSE	TRUE	FALSE	FALSE	TRUE	FALSE	FALSE	0	0
rs2093069	chr20	21159700	21159701	1f	0,55436	TRUE	TRUE	FALSE	FALSE	TRUE	FALSE	FALSE	0	0
rs6047279	chr20	21161711	21161712	1f	0,68	FALSE	TRUE	TRUE	TRUE	TRUE	FALSE	FALSE	1,96	0
rs4815025	chr20	21161881	21161882	1f	0,19549	FALSE	TRUE	FALSE	TRUE	TRUE	FALSE	FALSE	0	0
rs6035795	chr20	21165244	21165245	7	0,51392	FALSE	FALSE	FALSE	FALSE	TRUE	FALSE	FALSE	0	0
rs6047281	chr20	21165646	21165647	1f	0,22271	FALSE	TRUE	FALSE	FALSE	TRUE	FALSE	FALSE	0	0
rs6047282	chr20	21165827	21165828	1f	0,55324	TRUE	FALSE	FALSE	FALSE	TRUE	FALSE	FALSE	0	0
rs2876595	chr20	21166198	21166199	1f	0,55324	TRUE	FALSE	FALSE	FALSE	TRUE	FALSE	FALSE	0	0
rs6047283	chr20	21166368	21166369	7	0,51392	FALSE	FALSE	FALSE	FALSE	TRUE	FALSE	FALSE	0	0
rs6035796	chr20	21166766	21166767	1a	0,99267	TRUE	TRUE	TRUE	TRUE	TRUE	TRUE	TRUE	2	2
rs4625995	chr20	21167327	21167328	1f	0,55436	TRUE	TRUE	FALSE	FALSE	TRUE	FALSE	FALSE	0	0

rs4815026	chr20	21168042	21168043	1f	0,55324	TRUE	FALSE	FALSE	FALSE	TRUE	FALSE	FALSE	0	0
rs6132402	chr20	21169626	21169627	7	0,51392	FALSE	FALSE	FALSE	FALSE	TRUE	FALSE	FALSE	0	0
rs4815028	chr20	21169992	21169993	7	0,51392	FALSE	FALSE	FALSE	FALSE	TRUE	FALSE	FALSE	0	0
rs6047285	chr20	21170254	21170255	7	0,51392	FALSE	FALSE	FALSE	FALSE	TRUE	FALSE	FALSE	0	0
rs6047288	chr20	21170683	21170684	7	0,51392	FALSE	FALSE	FALSE	FALSE	TRUE	FALSE	FALSE	0	0
rs6035799	chr20	21170687	21170688	7	0,51392	FALSE	FALSE	FALSE	FALSE	TRUE	FALSE	FALSE	0	0
rs6035800	chr20	21170707	21170708	7	0,51392	FALSE	FALSE	FALSE	FALSE	TRUE	FALSE	FALSE	0	0
rs1072271	chr20	21170736	21170737	6	0,2561	FALSE	FALSE	TRUE	FALSE	TRUE	FALSE	FALSE	1,8	0
rs2180581	chr20	21171211	21171212	1f	0,55324	TRUE	FALSE	FALSE	FALSE	TRUE	FALSE	FALSE	0	0
rs6082338	chr20	21171590	21171591	1f	0,465	TRUE	FALSE	TRUE	TRUE	TRUE	FALSE	FALSE	1,96	0
rs6082339	chr20	21172101	21172102	6	0,22458	FALSE	FALSE	TRUE	FALSE	TRUE	FALSE	FALSE	0,44	0
rs2328608	chr20	21172497	21172498	6	0,26	FALSE	FALSE	TRUE	FALSE	TRUE	FALSE	FALSE	1,51	0
rs6035802	chr20	21173052	21173053	1f	0,55324	TRUE	FALSE	FALSE	FALSE	TRUE	FALSE	FALSE	0	0
rs6035803	chr20	21173141	21173142	1f	0,55436	TRUE	TRUE	FALSE	FALSE	TRUE	FALSE	FALSE	0	0
rs6035804	chr20	21173631	21173632	1b	0,95	TRUE	TRUE	TRUE	TRUE	TRUE	FALSE	FALSE	1,73	0
rs6047293	chr20	21173846	21173847	1b	0,95	TRUE	TRUE	TRUE	TRUE	TRUE	FALSE	FALSE	1,73	0
rs910975	chr20	21175528	21175529	1f	0,55436	TRUE	TRUE	FALSE	FALSE	TRUE	FALSE	FALSE	0	0
rs2328609	chr20	21175937	21175938	1f	0,55436	TRUE	TRUE	FALSE	FALSE	TRUE	FALSE	FALSE	0	0
rs2103975	chr20	21176290	21176291	7	0,51392	FALSE	FALSE	FALSE	FALSE	TRUE	FALSE	FALSE	0	0
rs2103976	chr20	21176319	21176320	6	0,80877	FALSE	FALSE	TRUE	FALSE	TRUE	FALSE	FALSE	1,99	0
rs2093070	chr20	21176869	21176870	7	0,51392	FALSE	FALSE	FALSE	FALSE	TRUE	FALSE	FALSE	0	0
rs6035808	chr20	21176924	21176925	7	0,51392	FALSE	FALSE	FALSE	FALSE	TRUE	FALSE	FALSE	0	0
rs6047296	chr20	21177598	21177599	1f	0,51221	TRUE	FALSE	TRUE	FALSE	TRUE	FALSE	FALSE	0,94	0
rs2876596	chr20	21177730	21177731	1f	0,55324	TRUE	FALSE	FALSE	FALSE	TRUE	FALSE	FALSE	0	0
rs2145099	chr20	21178511	21178512	7	0,51392	FALSE	FALSE	FALSE	FALSE	TRUE	FALSE	FALSE	0	0
rs6047299	chr20	21179523	21179524	5	0,58955	TRUE	FALSE	FALSE	FALSE	FALSE	FALSE	FALSE	0	0
rs4815030	chr20	21180142	21180143	1f	0,55324	TRUE	FALSE	FALSE	FALSE	TRUE	FALSE	FALSE	0	0
rs2145100	chr20	21183158	21183159	1f	0,55436	TRUE	TRUE	FALSE	FALSE	TRUE	FALSE	FALSE	0	0
rs8120377	chr20	21187720	21187721	1f	0,55324	TRUE	FALSE	FALSE	FALSE	TRUE	FALSE	FALSE	0	0
rs8120293	chr20	21187754	21187755	1f	0,55324	TRUE	FALSE	FALSE	FALSE	TRUE	FALSE	FALSE	0	0
rs57940584	chr20	21189013	21189014	1f	0,55324	TRUE	FALSE	FALSE	FALSE	TRUE	FALSE	FALSE	0	0
rs765585	chr20	21190531	21190532	1f	0,55436	TRUE	TRUE	FALSE	FALSE	TRUE	FALSE	FALSE	0	0
rs879064	chr20	21194909	21194910	7	0,51392	FALSE	FALSE	FALSE	FALSE	TRUE	FALSE	FALSE	0	0
rs2144782	chr20	21195964	21195965	1f	0,55436	TRUE	TRUE	FALSE	FALSE	TRUE	FALSE	FALSE	0	0
rs12625304	chr20	21201748	21201749	7	0,51392	FALSE	FALSE	FALSE	FALSE	TRUE	FALSE	FALSE	0	0
rs6035813	chr20	21227105	21227106	1f	0,55324	TRUE	FALSE	FALSE	FALSE	TRUE	FALSE	FALSE	0	0
rs11697462	chr20	21228387	21228388	1f	0,55436	TRUE	TRUE	FALSE	FALSE	TRUE	FALSE	FALSE	0	0
rs6082358	chr20	21249816	21249817	1f	0,55324	TRUE	FALSE	FALSE	FALSE	TRUE	FALSE	FALSE	0	0
rs1000176	chr20	21252914	21252915	1f	0,05	FALSE	TRUE	TRUE	TRUE	TRUE	FALSE	FALSE	1,63	0
rs6047333	chr20	21260819	21260820	7	0,51392	FALSE	FALSE	FALSE	FALSE	TRUE	FALSE	FALSE	0	0
rs910805	chr20	21267477	21267478	1f	0,66703	TRUE	TRUE	FALSE	TRUE	TRUE	FALSE	FALSE	0	0
rs6047339	chr20	21268899	21268900	1f	0,55324	TRUE	FALSE	FALSE	FALSE	TRUE	FALSE	FALSE	0	0
rs6082363	chr20	21270204	21270205	7	0,51392	FALSE	FALSE	FALSE	FALSE	TRUE	FALSE	FALSE	0	0
rs4813421	chr20	21277414	21277415	7	0,51392	FALSE	FALSE	FALSE	FALSE	TRUE	FALSE	FALSE	0	0
rs6047345	chr20	21277854	21277855	6	0,87945	FALSE	FALSE	TRUE	FALSE	TRUE	FALSE	FALSE	1,95	0
rs6047346	chr20	21277965	21277966	6	0,14536	FALSE	FALSE	TRUE	FALSE	TRUE	FALSE	FALSE	1,6	0
rs6047348	chr20	21281045	21281046	1b	0,98	TRUE	TRUE	TRUE	TRUE	TRUE	FALSE	FALSE	1,61	0
rs6035826	chr20	21283151	21283152	7	0,51392	FALSE	FALSE	FALSE	FALSE	TRUE	FALSE	FALSE	0	0
rs6047353	chr20	21283528	21283529	6	0,74429	FALSE	FALSE	TRUE	FALSE	TRUE	FALSE	FALSE	1,91	0
rs4815036	chr20	21284819	21284820	1f	0,1537	FALSE	TRUE	TRUE	TRUE	TRUE	FALSE	FALSE	0,49	0
rs6035830	chr20	21285089	21285090	1f	0,22271	FALSE	TRUE	FALSE	FALSE	TRUE	FALSE	FALSE	0	0

rs6035831	chr20	21285801	21285802	1d	0,10833	TRUE	TRUE	TRUE	FALSE	TRUE	FALSE	FALSE	1,13	0
rs726024	chr20	21286712	21286713	1f	0,55436	TRUE	TRUE	FALSE	FALSE	TRUE	FALSE	FALSE	0	0
rs6075788	chr20	21287630	21287631	1f	0,22271	FALSE	TRUE	FALSE	FALSE	TRUE	FALSE	FALSE	0	0
rs2328622	chr20	21289657	21289658	7	0,51392	FALSE	FALSE	FALSE	FALSE	TRUE	FALSE	FALSE	0	0
rs4813422	chr20	21290684	21290685	1f	0,55436	TRUE	TRUE	FALSE	FALSE	TRUE	FALSE	FALSE	0	0
rs6082372	chr20	21290914	21290915	1f	0,22271	FALSE	TRUE	FALSE	FALSE	TRUE	FALSE	FALSE	0	0
rs6035834	chr20	21290946	21290947	1f	0,55324	TRUE	FALSE	FALSE	FALSE	TRUE	FALSE	FALSE	0	0
rs6035835	chr20	21291030	21291031	1f	0,55436	TRUE	TRUE	FALSE	FALSE	TRUE	FALSE	FALSE	0	0
rs6047359	chr20	21291135	21291136	1f	0,55436	TRUE	TRUE	FALSE	FALSE	TRUE	FALSE	FALSE	0	0
rs6047360	chr20	21291448	21291449	1b	1	TRUE	TRUE	TRUE	TRUE	TRUE	FALSE	FALSE	0,12	0
rs6047361	chr20	21291892	21291893	1f	0,55436	TRUE	TRUE	FALSE	FALSE	TRUE	FALSE	FALSE	0	0
rs4815038	chr20	21292672	21292673	1b	0,68148	TRUE	TRUE	TRUE	TRUE	TRUE	FALSE	FALSE	1,99	0
rs6113185	chr20	21293228	21293229	1f	0,55324	TRUE	FALSE	FALSE	FALSE	TRUE	FALSE	FALSE	0	0
rs6035837	chr20	21294029	21294030	1b	0,912	TRUE	TRUE	TRUE	TRUE	TRUE	FALSE	FALSE	0,74	0
rs6075789	chr20	21294654	21294655	1f	0,22271	FALSE	TRUE	FALSE	FALSE	TRUE	FALSE	FALSE	0	0
rs4813424	chr20	21299181	21299182	1b	1	TRUE	TRUE	TRUE	TRUE	TRUE	TRUE	FALSE	2	2
rs6082377	chr20	21299700	21299701	7	0,51392	FALSE	FALSE	FALSE	FALSE	TRUE	FALSE	FALSE	0	0
rs6035839	chr20	21308963	21308964	1f	0,55324	TRUE	FALSE	FALSE	FALSE	TRUE	FALSE	FALSE	0	0
rs6035840	chr20	21310892	21310893	1f	0,55436	TRUE	TRUE	FALSE	FALSE	TRUE	FALSE	FALSE	0	0
rs6082384	chr20	21311199	21311200	1f	0,55324	TRUE	FALSE	FALSE	FALSE	TRUE	FALSE	FALSE	0	0
rs6035841	chr20	21311589	21311590	1f	0,14257	FALSE	TRUE	TRUE	TRUE	TRUE	FALSE	FALSE	1,87	0
rs6047370	chr20	21312172	21312173	1f	0,22271	FALSE	TRUE	FALSE	FALSE	TRUE	FALSE	FALSE	0	0
rs6047371	chr20	21312390	21312391	1f	0,22271	FALSE	TRUE	FALSE	FALSE	TRUE	FALSE	FALSE	0	0
rs1359761	chr20	21313245	21313246	1f	0,55436	TRUE	TRUE	FALSE	FALSE	TRUE	FALSE	FALSE	0	0
rs4813425	chr20	21314870	21314871	1f	0,55436	TRUE	TRUE	FALSE	FALSE	TRUE	FALSE	FALSE	0	0
rs6047372	chr20	21315920	21315921	1f	0,26917	TRUE	FALSE	TRUE	FALSE	TRUE	FALSE	FALSE	0,14	0
rs6047374	chr20	21318092	21318093	1f	0,35917	TRUE	FALSE	TRUE	FALSE	TRUE	FALSE	FALSE	1,61	0
rs6047375	chr20	21318093	21318094	1f	0,72917	TRUE	FALSE	TRUE	FALSE	TRUE	FALSE	FALSE	1,78	0
rs1327153	chr20	21319391	21319392	1f	0,34538	TRUE	FALSE	TRUE	FALSE	TRUE	FALSE	FALSE	0,16	0
rs6047377	chr20	21320838	21320839	6	0,85279	FALSE	FALSE	TRUE	FALSE	TRUE	FALSE	FALSE	1,98	0
rs1546997	chr20	21321926	21321927	1f	0,55436	TRUE	TRUE	FALSE	FALSE	TRUE	FALSE	FALSE	0	0
rs1546998	chr20	21321942	21321943	1f	0,55436	TRUE	TRUE	FALSE	FALSE	TRUE	FALSE	FALSE	0	0
rs4813426	chr20	21327057	21327058	1f	0,55436	TRUE	TRUE	FALSE	FALSE	TRUE	FALSE	FALSE	0	0
rs6047380	chr20	21328173	21328174	1f	0,55324	TRUE	FALSE	FALSE	FALSE	TRUE	FALSE	FALSE	0	0
rs4496388	chr20	21329676	21329677	7	0,51392	FALSE	FALSE	FALSE	FALSE	TRUE	FALSE	FALSE	0	0
rs13043724	chr20	21345459	21345460	1f	0,22271	FALSE	TRUE	FALSE	FALSE	TRUE	FALSE	FALSE	0	0
rs6047389	chr20	21346866	21346867	6	0,14536	FALSE	FALSE	TRUE	FALSE	TRUE	FALSE	FALSE	1,6	0
rs1958122	chr20	21349141	21349142	7	0,51392	FALSE	FALSE	FALSE	FALSE	TRUE	FALSE	FALSE	0	0
rs6047395	chr20	21350733	21350734	1f	0,55324	TRUE	FALSE	FALSE	FALSE	TRUE	FALSE	FALSE	0	0
rs6047396	chr20	21350902	21350903	1f	0,55324	TRUE	FALSE	FALSE	FALSE	TRUE	FALSE	FALSE	0	0
rs6035852	chr20	21351810	21351811	1f	0,31067	TRUE	FALSE	TRUE	FALSE	TRUE	FALSE	FALSE	1,53	0
rs6035853	chr20	21352023	21352024	7	0,51392	FALSE	FALSE	FALSE	FALSE	TRUE	FALSE	FALSE	0	0
rs6082393	chr20	21353012	21353013	1b	0,95508	TRUE	TRUE	TRUE	TRUE	TRUE	FALSE	TRUE	1,22	0
rs6075794	chr20	21353029	21353030	1f	0,66703	TRUE	TRUE	FALSE	TRUE	TRUE	FALSE	FALSE	0	0
rs1409816	chr20	21359218	21359219	1f	0,55324	TRUE	FALSE	FALSE	FALSE	TRUE	FALSE	FALSE	0	0
rs4497932	chr20	21361739	21361740	1f	0,55436	TRUE	TRUE	FALSE	FALSE	TRUE	FALSE	FALSE	0	0
rs6047404	chr20	21371597	21371598	1f	0,22271	FALSE	TRUE	FALSE	FALSE	TRUE	FALSE	FALSE	0	0
rs6035860	chr20	21371666	21371667	6	0,56788	FALSE	FALSE	TRUE	TRUE	TRUE	FALSE	FALSE	1,98	0
rs4815041	chr20	21372770	21372771	1f	0,55324	TRUE	FALSE	FALSE	FALSE	TRUE	FALSE	FALSE	0	0
rs4815042	chr20	21372840	21372841	1f	0,55324	TRUE	FALSE	FALSE	FALSE	TRUE	FALSE	FALSE	0	0
rs6047405	chr20	21373276	21373277	1f	0,55436	TRUE	TRUE	FALSE	FALSE	TRUE	FALSE	FALSE	0	0

rs6035862	chr20	21374421	21374422	1f	0,86	TRUE	FALSE	TRUE	TRUE	TRUE	FALSE	FALSE	1,89	0
rs6047407	chr20	21375207	21375208	6	0,01633	FALSE	FALSE	TRUE	TRUE	FALSE	FALSE	FALSE	0,16	0
rs6047414	chr20	21379889	21379890	7	0,51392	FALSE	FALSE	FALSE	FALSE	TRUE	FALSE	FALSE	0	0
rs6035865	chr20	21388293	21388294	1f	0,22271	FALSE	TRUE	FALSE	FALSE	TRUE	FALSE	FALSE	0	0
rs1555946	chr20	21390732	21390733	1f	0,55324	TRUE	FALSE	FALSE	FALSE	TRUE	FALSE	FALSE	0	0
rs720324	chr20	21400133	21400134	1f	0,55436	TRUE	TRUE	FALSE	FALSE	TRUE	FALSE	FALSE	0	0
rs6132424	chr20	21412560	21412561	6	0,14536	FALSE	FALSE	TRUE	FALSE	TRUE	FALSE	FALSE	1,6	0
rs2424362	chr20	21422128	21422129	1f	0,55324	TRUE	FALSE	FALSE	FALSE	TRUE	FALSE	FALSE	0	0
rs804373	chr20	21428074	21428075	1f	0,81932	TRUE	FALSE	TRUE	FALSE	TRUE	FALSE	FALSE	2	0
rs811698	chr20	21436040	21436041	1f	0,55436	TRUE	TRUE	FALSE	FALSE	TRUE	FALSE	FALSE	0	0
rs28650836	chr20	21439853	21439854	1f	0,22271	FALSE	TRUE	FALSE	FALSE	TRUE	FALSE	FALSE	0	0
rs8124380	chr20	21440236	21440237	1f	0,87917	TRUE	FALSE	TRUE	FALSE	TRUE	FALSE	FALSE	1,96	0
rs57897530	chr20	21444511	21444512	6	0,72948	FALSE	FALSE	TRUE	FALSE	FALSE	FALSE	FALSE	0,86	0
rs804381	chr20	21444954	21444955	1f	0,55324	TRUE	FALSE	FALSE	FALSE	TRUE	FALSE	FALSE	0	0
rs804383	chr20	21449931	21449932	7	0,51392	FALSE	FALSE	FALSE	FALSE	TRUE	FALSE	FALSE	0	0
rs804384	chr20	21456153	21456154	1f	0,55324	TRUE	FALSE	FALSE	FALSE	TRUE	FALSE	FALSE	0	0
rs804385	chr20	21456674	21456675	7	0,51392	FALSE	FALSE	FALSE	FALSE	TRUE	FALSE	FALSE	0	0
rs2424370	chr20	21457200	21457201	6	0,12848	FALSE	FALSE	TRUE	FALSE	TRUE	FALSE	FALSE	0,11	0
rs8114493	chr20	21463033	21463034	1f	0,55324	TRUE	FALSE	FALSE	FALSE	TRUE	FALSE	FALSE	0	0
rs722095	chr20	21465659	21465660	7	0,51392	FALSE	FALSE	FALSE	FALSE	TRUE	FALSE	FALSE	0	0
rs4858241	chr3	20627578	20627579	7	0,18412	FALSE	FALSE	FALSE	FALSE	FALSE	FALSE	FALSE	0	0
rs1846723	chr3	20678504	20678505	7	0,51392	FALSE	FALSE	FALSE	FALSE	TRUE	FALSE	FALSE	0	0
rs56285459	chr3	20680325	20680326	7	0,51392	FALSE	FALSE	FALSE	FALSE	TRUE	FALSE	FALSE	0	0
rs55712289	chr3	20680426	20680427	7	0,51392	FALSE	FALSE	FALSE	FALSE	TRUE	FALSE	FALSE	0	0
rs1604133	chr3	20680837	20680838	7	0,51392	FALSE	FALSE	FALSE	FALSE	TRUE	FALSE	FALSE	0	0
rs2886697	chr3	20682711	20682712	1b	0,7915	TRUE	TRUE	TRUE	TRUE	TRUE	FALSE	FALSE	0,98	0
rs55686218	chr3	20683443	20683444	7	0,18412	FALSE	FALSE	FALSE	FALSE	FALSE	FALSE	FALSE	0	0
rs56135409	chr3	20683523	20683524	7	0,51392	FALSE	FALSE	FALSE	FALSE	TRUE	FALSE	FALSE	0	0
rs4858250	chr3	20685149	20685150	7	0,51392	FALSE	FALSE	FALSE	FALSE	TRUE	FALSE	FALSE	0	0
rs4858251	chr3	20685153	20685154	7	0,51392	FALSE	FALSE	FALSE	FALSE	TRUE	FALSE	FALSE	0	0
rs4858253	chr3	20687598	20687599	7	0,51392	FALSE	FALSE	FALSE	FALSE	TRUE	FALSE	FALSE	0	0
rs62234988	chr3	20695380	20695381	1f	0,22271	FALSE	TRUE	FALSE	FALSE	TRUE	FALSE	FALSE	0	0
rs17809565	chr3	20696596	20696597	1f	0,22271	FALSE	TRUE	FALSE	FALSE	TRUE	FALSE	FALSE	0	0
rs2366311	chr3	20698840	20698841	1f	0,55324	TRUE	FALSE	FALSE	FALSE	TRUE	FALSE	FALSE	0	0
rs55964255	chr3	20700798	20700799	1f	0,55436	TRUE	TRUE	FALSE	FALSE	TRUE	FALSE	FALSE	0	0
rs62234991	chr3	20703003	20703004	7	0,51392	FALSE	FALSE	FALSE	FALSE	TRUE	FALSE	FALSE	0	0
rs62234992	chr3	20706827	20706828	7	0,51392	FALSE	FALSE	FALSE	FALSE	TRUE	FALSE	FALSE	0	0
rs17718444	chr3	71450249	71450250	2b	0,8288	TRUE	TRUE	TRUE	TRUE	FALSE	FALSE	FALSE	0,02	0
rs11729045	chr4	111251829	111251830	7	0,18412	FALSE	FALSE	FALSE	FALSE	FALSE	FALSE	FALSE	0	0
rs79861884	chr4	111267774	111267775	5	0,22625	TRUE	FALSE	TRUE	FALSE	FALSE	FALSE	FALSE	1,54	0
rs79573484	chr4	111287591	111287592	5	0,13454	FALSE	TRUE	FALSE	FALSE	FALSE	FALSE	FALSE	0	0
rs56331412	chr4	111295016	111295017	7	0,18412	FALSE	FALSE	FALSE	FALSE	FALSE	FALSE	FALSE	0	0
rs17576773	chr4	111296366	111296367	5	0,73375	TRUE	FALSE	TRUE	TRUE	FALSE	FALSE	FALSE	1,78	0
rs17638593	chr4	111296653	111296654	4	0,60906	TRUE	TRUE	FALSE	FALSE	FALSE	FALSE	FALSE	0	0
rs77216804	chr4	111306829	111306830	4	0,60906	TRUE	TRUE	FALSE	FALSE	FALSE	FALSE	FALSE	0	0
rs12500836	chr4	111309355	111309356	1f	0,19549	FALSE	TRUE	FALSE	TRUE	TRUE	FALSE	FALSE	0	0
rs28391418	chr4	111310700	111310701	7	0,18412	FALSE	FALSE	FALSE	FALSE	FALSE	FALSE	FALSE	0	0
rs76729396	chr4	111311006	111311007	5	0,13454	FALSE	TRUE	FALSE	FALSE	FALSE	FALSE	FALSE	0	0
rs76675076	chr4	111311549	111311550	6	0,24967	FALSE	FALSE	TRUE	FALSE	FALSE	FALSE	FALSE	1,72	0
rs28865977	chr4	111314598	111314599	1f	0,22271	FALSE	TRUE	FALSE	FALSE	TRUE	FALSE	FALSE	0	0
rs12505218	chr4	111322535	111322536	1f	0,55324	TRUE	FALSE	FALSE	FALSE	TRUE	FALSE	FALSE	0	0

rs7665430	chr4	111328736	111328737	7	0,18412	FALSE	FALSE	FALSE	FALSE	FALSE	FALSE	FALSE	0	0
rs12503582	chr4	111333131	111333132	7	0,18412	FALSE	FALSE	FALSE	FALSE	FALSE	FALSE	FALSE	0	0
rs72678859	chr4	111485804	111485805	7	0,18412	FALSE	FALSE	FALSE	FALSE	FALSE	FALSE	FALSE	0	0
rs72678864	chr4	111500988	111500989	1f	0,55436	TRUE	TRUE	FALSE	FALSE	TRUE	FALSE	FALSE	0	0
rs3934797	chr4	111546455	111546456	1f	0,55436	TRUE	TRUE	FALSE	FALSE	TRUE	FALSE	FALSE	0	0
rs11729080	chr4	111582715	111582716	7	0,18412	FALSE	FALSE	FALSE	FALSE	FALSE	FALSE	FALSE	0	0
rs6452784	chr5	88385176	88385177	1f	0,55436	TRUE	TRUE	FALSE	FALSE	TRUE	FALSE	FALSE	0	0
rs6414945	chr5	88386212	88386213	6	0,25649	FALSE	FALSE	TRUE	FALSE	TRUE	FALSE	FALSE	1,76	0
rs6452785	chr5	88389682	88389683	4	0,60906	TRUE	TRUE	FALSE	FALSE	FALSE	FALSE	FALSE	0	0
rs2898558	chr5	88397819	88397820	1f	0,55436	TRUE	TRUE	FALSE	FALSE	TRUE	FALSE	FALSE	0	0
rs4916910	chr5	88401025	88401026	1f	0,22271	FALSE	TRUE	FALSE	FALSE	TRUE	FALSE	FALSE	0	0
rs7708715	chr5	88416701	88416702	1f	0,22271	FALSE	TRUE	FALSE	FALSE	TRUE	FALSE	FALSE	0	0
rs6452787	chr5	88417013	88417014	1f	0,22271	FALSE	TRUE	FALSE	FALSE	TRUE	FALSE	FALSE	0	0
rs6414946	chr5	88433893	88433894	7	0,51392	FALSE	FALSE	FALSE	FALSE	TRUE	FALSE	FALSE	0	0
rs7722095	chr5	88445075	88445076	6	0,81321	FALSE	FALSE	TRUE	TRUE	TRUE	FALSE	FALSE	1,99	0
rs7728883	chr5	88455934	88455935	6	0,77667	FALSE	FALSE	TRUE	TRUE	TRUE	FALSE	FALSE	1,94	0
rs7448716	chr5	88456877	88456878	7	0,51392	FALSE	FALSE	FALSE	FALSE	TRUE	FALSE	FALSE	0	0
rs6866315	chr5	88458987	88458988	7	0,51392	FALSE	FALSE	FALSE	FALSE	TRUE	FALSE	FALSE	0	0
rs4352629	chr5	88461003	88461004	1f	0,55436	TRUE	TRUE	FALSE	FALSE	TRUE	FALSE	FALSE	0	0
rs4571506	chr5	88461100	88461101	1f	0,55436	TRUE	TRUE	FALSE	FALSE	TRUE	FALSE	FALSE	0	0
rs10060720	chr5	88467650	88467651	1f	0,5151	TRUE	FALSE	TRUE	FALSE	TRUE	FALSE	FALSE	1,89	0
rs11952470	chr5	88467698	88467699	1f	0,55324	TRUE	FALSE	FALSE	FALSE	TRUE	FALSE	FALSE	0	0
rs4244212	chr5	88468800	88468801	1f	0,22271	FALSE	TRUE	FALSE	FALSE	TRUE	FALSE	FALSE	0	0
rs9293500	chr5	88469953	88469954	7	0,51392	FALSE	FALSE	FALSE	FALSE	TRUE	FALSE	FALSE	0	0
rs6891239	chr5	88473875	88473876	7	0,51392	FALSE	FALSE	FALSE	FALSE	TRUE	FALSE	FALSE	0	0
rs6452791	chr5	88475275	88475276	1f	0,22271	FALSE	TRUE	FALSE	FALSE	TRUE	FALSE	FALSE	0	0
rs6873449	chr5	88477837	88477838	6	0,27045	FALSE	FALSE	TRUE	FALSE	TRUE	FALSE	FALSE	1,79	0
rs6874021	chr5	88477911	88477912	7	0,51392	FALSE	FALSE	FALSE	FALSE	TRUE	FALSE	FALSE	0	0
rs6452792	chr5	88497026	88497027	7	0,51392	FALSE	FALSE	FALSE	FALSE	TRUE	FALSE	FALSE	0	0
rs2194027	chr5	88526853	88526854	7	0,51392	FALSE	FALSE	FALSE	FALSE	TRUE	FALSE	FALSE	0	0
rs12653396	chr5	88551454	88551455	6	0,17585	FALSE	FALSE	TRUE	FALSE	TRUE	FALSE	FALSE	0,05	0
rs4916723	chr5	88558576	88558577	1f	0,03	FALSE	TRUE	TRUE	TRUE	TRUE	FALSE	FALSE	1,72	0
rs17422060	chr5	88563111	88563112	1f	0,55436	TRUE	TRUE	FALSE	FALSE	TRUE	FALSE	FALSE	0	0
rs1659071	chr5	88567791	88567792	1f	0,22271	FALSE	TRUE	FALSE	FALSE	TRUE	FALSE	FALSE	0	0
rs1644047	chr5	88569969	88569970	7	0,51392	FALSE	FALSE	FALSE	FALSE	TRUE	FALSE	FALSE	0	0
rs10063583	chr5	88574394	88574395	1f	0,22271	FALSE	TRUE	FALSE	FALSE	TRUE	FALSE	FALSE	0	0
rs10071454	chr5	88574429	88574430	1f	0,22271	FALSE	TRUE	FALSE	FALSE	TRUE	FALSE	FALSE	0	0
rs918429	chr5	88577804	88577805	7	0,51392	FALSE	FALSE	FALSE	FALSE	TRUE	FALSE	FALSE	0	0
rs7722396	chr5	88579560	88579561	6	0,27286	FALSE	FALSE	TRUE	FALSE	TRUE	FALSE	FALSE	0,9	0
rs7721028	chr5	88584753	88584754	7	0,51392	FALSE	FALSE	FALSE	FALSE	TRUE	FALSE	FALSE	0	0
rs1080255	chr5	88586360	88586361	1f	0,01	FALSE	TRUE	TRUE	TRUE	TRUE	FALSE	FALSE	0,22	0
rs62369151	chr5	88587684	88587685	1f	0,22271	FALSE	TRUE	FALSE	FALSE	TRUE	FALSE	FALSE	0	0
rs56022653	chr5	88588019	88588020	7	0,51392	FALSE	FALSE	FALSE	FALSE	TRUE	FALSE	FALSE	0	0
rs324886	chr5	88600783	88600784	1f	0,6032	TRUE	FALSE	TRUE	TRUE	TRUE	FALSE	FALSE	0	0
rs324885	chr5	88601452	88601453	1f	0,08	FALSE	TRUE	TRUE	TRUE	TRUE	FALSE	FALSE	1,54	0
rs6859545	chr5	88605344	88605345	1f	0,55436	TRUE	TRUE	FALSE	FALSE	TRUE	FALSE	FALSE	0	0
rs324895	chr5	88618012	88618013	7	0,51392	FALSE	FALSE	FALSE	FALSE	TRUE	FALSE	FALSE	0	0
rs324896	chr5	88618445	88618446	6	0,15544	FALSE	FALSE	TRUE	FALSE	TRUE	FALSE	FALSE	0,14	0
rs10462334	chr5	88619662	88619663	7	0,51392	FALSE	FALSE	FALSE	FALSE	TRUE	FALSE	FALSE	0	0
rs1644042	chr5	88624487	88624488	7	0,51392	FALSE	FALSE	FALSE	FALSE	TRUE	FALSE	FALSE	0	0
rs1644044	chr5	88628063	88628064	1f	0,22271	FALSE	TRUE	FALSE	FALSE	TRUE	FALSE	FALSE	0	0

rs1644045	chr5	88629002	88629003	1f	0,55324	TRUE	FALSE	FALSE	FALSE	TRUE	FALSE	FALSE	0	0
rs11740358	chr5	88630644	88630645	1f	0,55436	TRUE	TRUE	FALSE	FALSE	TRUE	FALSE	FALSE	0	0
rs1628250	chr5	88633147	88633148	1f	0,22271	FALSE	TRUE	FALSE	FALSE	TRUE	FALSE	FALSE	0	0
rs4916661	chr5	88636990	88636991	6	0,74073	FALSE	FALSE	TRUE	FALSE	TRUE	FALSE	FALSE	2	0
rs2362108	chr5	88851953	88851954	7	0,51392	FALSE	FALSE	FALSE	FALSE	TRUE	FALSE	FALSE	0	0
rs2362109	chr5	88852223	88852224	7	0,51392	FALSE	FALSE	FALSE	FALSE	TRUE	FALSE	FALSE	0	0
rs17487515	chr5	88853473	88853474	1f	0,8313	TRUE	FALSE	TRUE	FALSE	TRUE	FALSE	FALSE	1,98	0
rs17559709	chr5	88853508	88853509	6	0,61	FALSE	FALSE	TRUE	FALSE	TRUE	FALSE	FALSE	1,07	0
rs4145738	chr5	88854471	88854472	1b	0,955	TRUE	TRUE	TRUE	TRUE	TRUE	FALSE	FALSE	0,05	0
rs57055575	chr5	88855174	88855175	1b	0,20849	TRUE	TRUE	TRUE	TRUE	TRUE	FALSE	FALSE	0,23	0
rs4373304	chr5	88856384	88856385	1f	0,55324	TRUE	FALSE	FALSE	FALSE	TRUE	FALSE	FALSE	0	0
rs11749060	chr5	88856756	88856757	1f	0,55436	TRUE	TRUE	FALSE	FALSE	TRUE	FALSE	FALSE	0	0
rs55740951	chr5	88858056	88858057	1f	0,55324	TRUE	FALSE	FALSE	FALSE	TRUE	FALSE	FALSE	0	0
rs4518438	chr5	88861734	88861735	7	0,51392	FALSE	FALSE	FALSE	FALSE	TRUE	FALSE	FALSE	0	0
rs7730255	chr5	88862328	88862329	1f	0,55324	TRUE	FALSE	FALSE	FALSE	TRUE	FALSE	FALSE	0	0
rs56947091	chr5	88864536	88864537	5	0,58955	TRUE	FALSE	FALSE	FALSE	FALSE	FALSE	FALSE	0	0
rs12054920	chr5	88865599	88865600	1d	0,31774	TRUE	TRUE	TRUE	FALSE	TRUE	FALSE	FALSE	0,5	0
rs61104616	chr5	88867953	88867954	1d	0,92597	TRUE	TRUE	TRUE	FALSE	TRUE	FALSE	FALSE	0,12	0
rs188582	chr5	88871922	88871923	1f	0,22271	FALSE	TRUE	FALSE	FALSE	TRUE	FALSE	FALSE	0	0
rs1671539	chr5	88872577	88872578	1f	0,55436	TRUE	TRUE	FALSE	FALSE	TRUE	FALSE	FALSE	0	0
rs797419	chr5	88873137	88873138	1b	0,85583	TRUE	TRUE	TRUE	TRUE	TRUE	TRUE	FALSE	1,98	1,9
rs304137	chr5	88873834	88873835	1f	0,55324	TRUE	FALSE	FALSE	FALSE	TRUE	FALSE	FALSE	0	0
rs304136	chr5	88874248	88874249	1f	0,22271	FALSE	TRUE	FALSE	FALSE	TRUE	FALSE	FALSE	0	0
rs216057	chr5	88874513	88874514	1f	0,02937	FALSE	TRUE	TRUE	TRUE	TRUE	FALSE	FALSE	0,03	0
rs188581	chr5	88879433	88879434	1f	0,55324	TRUE	FALSE	FALSE	FALSE	TRUE	FALSE	FALSE	0	0
rs3850651	chr5	88885291	88885292	1f	0,66703	TRUE	TRUE	FALSE	TRUE	TRUE	FALSE	FALSE	0	0
rs4235642	chr5	104482710	104482711	6	0,09219	FALSE	FALSE	TRUE	TRUE	FALSE	FALSE	FALSE	1,86	0
rs6869862	chr5	104550511	104550512	1f	0,22271	FALSE	TRUE	FALSE	FALSE	TRUE	FALSE	FALSE	0	0
rs1592757	chr5	104554296	104554297	6	0,27492	FALSE	FALSE	TRUE	FALSE	TRUE	FALSE	FALSE	0,64	0
rs6867409	chr5	104554394	104554395	7	0,51392	FALSE	FALSE	FALSE	FALSE	TRUE	FALSE	FALSE	0	0
rs2193933	chr5	104556758	104556759	1f	0,55436	TRUE	TRUE	FALSE	FALSE	TRUE	FALSE	FALSE	0	0
rs1592756	chr5	104557286	104557287	1f	0,48567	TRUE	FALSE	TRUE	TRUE	TRUE	FALSE	FALSE	1,7	0
rs1592755	chr5	104557846	104557847	7	0,51392	FALSE	FALSE	FALSE	FALSE	TRUE	FALSE	FALSE	0	0
rs7706353	chr5	104559314	104559315	7	0,51392	FALSE	FALSE	FALSE	FALSE	TRUE	FALSE	FALSE	0	0
rs4438849	chr5	104559400	104559401	7	0,51392	FALSE	FALSE	FALSE	FALSE	TRUE	FALSE	FALSE	0	0
rs4320234	chr5	104559413	104559414	7	0,51392	FALSE	FALSE	FALSE	FALSE	TRUE	FALSE	FALSE	0	0
rs1833514	chr5	104560428	104560429	1f	0,55436	TRUE	TRUE	FALSE	FALSE	TRUE	FALSE	FALSE	0	0
rs4521446	chr5	104562056	104562057	7	0,51392	FALSE	FALSE	FALSE	FALSE	TRUE	FALSE	FALSE	0	0
rs6596578	chr5	104563476	104563477	1f	0,22271	FALSE	TRUE	FALSE	FALSE	TRUE	FALSE	FALSE	0	0
rs6874138	chr5	104563894	104563895	7	0,51392	FALSE	FALSE	FALSE	FALSE	TRUE	FALSE	FALSE	0	0
rs10064425	chr5	104563988	104563989	7	0,51392	FALSE	FALSE	FALSE	FALSE	TRUE	FALSE	FALSE	0	0
rs10059133	chr5	104565722	104565723	6	0,17523	FALSE	FALSE	TRUE	FALSE	TRUE	FALSE	FALSE	0,12	0
rs6865511	chr5	104566478	104566479	1f	0,15917	TRUE	FALSE	TRUE	FALSE	TRUE	FALSE	FALSE	1,11	0
rs1421668	chr5	104567256	104567257	7	0,51392	FALSE	FALSE	FALSE	FALSE	TRUE	FALSE	FALSE	0	0
rs12659431	chr5	104568064	104568065	7	0,51392	FALSE	FALSE	FALSE	FALSE	TRUE	FALSE	FALSE	0	0
rs12657531	chr5	104568135	104568136	7	0,51392	FALSE	FALSE	FALSE	FALSE	TRUE	FALSE	FALSE	0	0
rs12658007	chr5	104568332	104568333	1f	0,55324	TRUE	FALSE	FALSE	FALSE	TRUE	FALSE	FALSE	0	0
rs12658451	chr5	104568335	104568336	1f	0,53917	TRUE	FALSE	TRUE	FALSE	TRUE	FALSE	FALSE	1,34	0
rs12658019	chr5	104568406	104568407	1f	0,55324	TRUE	FALSE	FALSE	FALSE	TRUE	FALSE	FALSE	0	0
rs12658032	chr5	104568524	104568525	1f	0,22271	FALSE	TRUE	FALSE	FALSE	TRUE	FALSE	FALSE	0	0
rs11242522	chr5	104569212	104569213	6	0,17	FALSE	FALSE	TRUE	FALSE	TRUE	FALSE	FALSE	0,54	0

rs11242523	chr5	104569323	104569324	7	0,51392	FALSE	FALSE	FALSE	FALSE	TRUE	FALSE	FALSE	0	0
rs12515429	chr5	104569573	104569574	1f	0,18086	FALSE	TRUE	TRUE	TRUE	TRUE	FALSE	FALSE	1,84	0
rs17156671	chr5	104569708	104569709	1f	0,55436	TRUE	TRUE	FALSE	FALSE	TRUE	FALSE	FALSE	0	0
rs2403284	chr5	104570371	104570372	1f	0,22271	FALSE	TRUE	FALSE	FALSE	TRUE	FALSE	FALSE	0	0
rs7703746	chr5	104571635	104571636	7	0,51392	FALSE	FALSE	FALSE	FALSE	TRUE	FALSE	FALSE	0	0
rs1421667	chr5	104571904	104571905	7	0,51392	FALSE	FALSE	FALSE	FALSE	TRUE	FALSE	FALSE	0	0
rs2032790	chr5	104572544	104572545	6	0,319	FALSE	FALSE	TRUE	FALSE	TRUE	FALSE	FALSE	1,05	0
rs1421666	chr5	104572708	104572709	6	0,27045	FALSE	FALSE	TRUE	FALSE	TRUE	FALSE	FALSE	1,79	0
rs4295362	chr5	104573166	104573167	7	0,51392	FALSE	FALSE	FALSE	FALSE	TRUE	FALSE	FALSE	0	0
rs959857	chr5	104573846	104573847	6	0,15	FALSE	FALSE	TRUE	FALSE	TRUE	FALSE	FALSE	1,54	0
rs62362443	chr5	104576412	104576413	1f	0,55324	TRUE	FALSE	FALSE	FALSE	TRUE	FALSE	FALSE	0	0
rs10071115	chr5	104576506	104576507	1f	0,51511	TRUE	FALSE	TRUE	TRUE	TRUE	FALSE	FALSE	1,98	0
rs11738191	chr5	104577500	104577501	7	0,51392	FALSE	FALSE	FALSE	FALSE	TRUE	FALSE	FALSE	0	0
rs11738197	chr5	104577562	104577563	7	0,51392	FALSE	FALSE	FALSE	FALSE	TRUE	FALSE	FALSE	0	0
rs13172611	chr5	104577566	104577567	7	0,51392	FALSE	FALSE	FALSE	FALSE	TRUE	FALSE	FALSE	0	0
rs13158494	chr5	104577619	104577620	7	0,51392	FALSE	FALSE	FALSE	FALSE	TRUE	FALSE	FALSE	0	0
rs1363106	chr5	104578054	104578055	6	0,20133	FALSE	FALSE	TRUE	FALSE	TRUE	FALSE	FALSE	0,36	0
rs1421665	chr5	104578312	104578313	6	0,27286	FALSE	FALSE	TRUE	FALSE	TRUE	FALSE	FALSE	0,9	0
rs13164188	chr5	104578821	104578822	7	0,51392	FALSE	FALSE	FALSE	FALSE	TRUE	FALSE	FALSE	0	0
rs13177365	chr5	104578841	104578842	7	0,51392	FALSE	FALSE	FALSE	FALSE	TRUE	FALSE	FALSE	0	0
rs10053368	chr5	104579056	104579057	6	0,21138	FALSE	FALSE	TRUE	FALSE	TRUE	FALSE	FALSE	0,01	0
rs10053371	chr5	104579084	104579085	6	0,17686	FALSE	FALSE	TRUE	FALSE	TRUE	FALSE	FALSE	0,57	0
rs10054977	chr5	104579134	104579135	7	0,51392	FALSE	FALSE	FALSE	FALSE	TRUE	FALSE	FALSE	0	0
rs12658276	chr5	104579580	104579581	6	0,12848	FALSE	FALSE	TRUE	FALSE	TRUE	FALSE	FALSE	0,11	0
rs10479296	chr5	104579741	104579742	7	0,51392	FALSE	FALSE	FALSE	FALSE	TRUE	FALSE	FALSE	0	0
rs10479297	chr5	104580026	104580027	1f	0,55436	TRUE	TRUE	FALSE	FALSE	TRUE	FALSE	FALSE	0	0
rs7710489	chr5	104580647	104580648	1f	0,22271	FALSE	TRUE	FALSE	FALSE	TRUE	FALSE	FALSE	0	0
rs7723509	chr5	104580661	104580662	1f	0,22271	FALSE	TRUE	FALSE	FALSE	TRUE	FALSE	FALSE	0	0
rs62362459	chr5	104581129	104581130	1f	0,22271	FALSE	TRUE	FALSE	FALSE	TRUE	FALSE	FALSE	0	0
rs10477834	chr5	104581650	104581651	6	0,13	FALSE	FALSE	TRUE	FALSE	TRUE	FALSE	FALSE	1,53	0
rs10477835	chr5	104581837	104581838	7	0,51392	FALSE	FALSE	FALSE	FALSE	TRUE	FALSE	FALSE	0	0
rs1363103	chr5	104582135	104582136	7	0,51392	FALSE	FALSE	FALSE	FALSE	TRUE	FALSE	FALSE	0	0
rs1363102	chr5	104582165	104582166	7	0,51392	FALSE	FALSE	FALSE	FALSE	TRUE	FALSE	FALSE	0	0
rs10072579	chr5	104582526	104582527	6	0,16	FALSE	FALSE	TRUE	FALSE	TRUE	FALSE	FALSE	1,55	0
rs10072849	chr5	104582920	104582921	6	0,275	FALSE	FALSE	TRUE	FALSE	TRUE	FALSE	FALSE	0,92	0
rs10057459	chr5	104582997	104582998	7	0,51392	FALSE	FALSE	FALSE	FALSE	TRUE	FALSE	FALSE	0	0
rs10057469	chr5	104583047	104583048	7	0,51392	FALSE	FALSE	FALSE	FALSE	TRUE	FALSE	FALSE	0	0
rs10052804	chr5	104584120	104584121	7	0,51392	FALSE	FALSE	FALSE	FALSE	TRUE	FALSE	FALSE	0	0
rs10059643	chr5	104584138	104584139	7	0,51392	FALSE	FALSE	FALSE	FALSE	TRUE	FALSE	FALSE	0	0
rs254011	chr5	104585068	104585069	7	0,51392	FALSE	FALSE	FALSE	FALSE	TRUE	FALSE	FALSE	0	0
rs254013	chr5	104590342	104590343	7	0,51392	FALSE	FALSE	FALSE	FALSE	TRUE	FALSE	FALSE	0	0
rs445578	chr5	104590740	104590741	7	0,51392	FALSE	FALSE	FALSE	FALSE	TRUE	FALSE	FALSE	0	0
rs2447838	chr5	104591877	104591878	7	0,51392	FALSE	FALSE	FALSE	FALSE	TRUE	FALSE	FALSE	0	0
rs2431112	chr5	104596005	104596006	1f	0,5055	TRUE	FALSE	TRUE	FALSE	TRUE	FALSE	FALSE	1,87	0
rs2447832	chr5	104597771	104597772	7	0,51392	FALSE	FALSE	FALSE	FALSE	TRUE	FALSE	FALSE	0	0
rs254035	chr5	104600641	104600642	7	0,51392	FALSE	FALSE	FALSE	FALSE	TRUE	FALSE	FALSE	0	0
rs1592754	chr5	104602413	104602414	6	0,6	FALSE	FALSE	TRUE	FALSE	TRUE	FALSE	FALSE	1,11	0
rs12187898	chr5	104603972	104603973	7	0,51392	FALSE	FALSE	FALSE	FALSE	TRUE	FALSE	FALSE	0	0
rs12187903	chr5	104604068	104604069	6	0,22267	FALSE	FALSE	TRUE	FALSE	TRUE	FALSE	FALSE	0,4	0
rs1363101	chr5	104605368	104605369	1f	0,22271	FALSE	TRUE	FALSE	FALSE	TRUE	FALSE	FALSE	0	0
rs10455065	chr5	104605699	104605700	1f	0,39805	TRUE	FALSE	TRUE	TRUE	TRUE	FALSE	FALSE	0,16	0

rs10455066	chr5	104605713	104605714	1f	0,55324	TRUE	FALSE	FALSE	FALSE	TRUE	FALSE	FALSE	0	0
rs2018142	chr5	104606091	104606092	7	0,51392	FALSE	FALSE	FALSE	FALSE	TRUE	FALSE	FALSE	0	0
rs254025	chr5	104608236	104608237	7	0,51392	FALSE	FALSE	FALSE	FALSE	TRUE	FALSE	FALSE	0	0
rs254024	chr5	104608318	104608319	6	0,23502	FALSE	FALSE	TRUE	FALSE	TRUE	FALSE	FALSE	1,67	0
rs2431109	chr5	104609424	104609425	1f	0,55324	TRUE	FALSE	FALSE	FALSE	TRUE	FALSE	FALSE	0	0
rs2161097	chr5	104609476	104609477	1f	0,55324	TRUE	FALSE	FALSE	FALSE	TRUE	FALSE	FALSE	0	0
rs1363100	chr5	104610504	104610505	7	0,51392	FALSE	FALSE	FALSE	FALSE	TRUE	FALSE	FALSE	0	0
rs2112163	chr5	104610780	104610781	1f	0,14257	FALSE	TRUE	TRUE	TRUE	TRUE	FALSE	FALSE	1,87	0
rs2447828	chr5	104611503	104611504	1f	0,55436	TRUE	TRUE	FALSE	FALSE	TRUE	FALSE	FALSE	0	0
rs2431108	chr5	104612266	104612267	1f	0,55436	TRUE	TRUE	FALSE	FALSE	TRUE	FALSE	FALSE	0	0
rs2447827	chr5	104612343	104612344	1f	0,55436	TRUE	TRUE	FALSE	FALSE	TRUE	FALSE	FALSE	0	0
rs254020	chr5	104614549	104614550	7	0,51392	FALSE	FALSE	FALSE	FALSE	TRUE	FALSE	FALSE	0	0
rs173823	chr5	104619099	104619100	7	0,51392	FALSE	FALSE	FALSE	FALSE	TRUE	FALSE	FALSE	0	0
rs254023	chr5	104619659	104619660	7	0,51392	FALSE	FALSE	FALSE	FALSE	TRUE	FALSE	FALSE	0	0
rs171697	chr5	104620814	104620815	1f	0,55324	TRUE	FALSE	FALSE	FALSE	TRUE	FALSE	FALSE	0	0
rs254045	chr5	104623643	104623644	1f	0,07	FALSE	TRUE	TRUE	TRUE	TRUE	FALSE	FALSE	1,36	0
rs77960	chr5	104628883	104628884	7	0,51392	FALSE	FALSE	FALSE	FALSE	TRUE	FALSE	FALSE	0	0
rs185260	chr5	104629521	104629522	1f	0,55436	TRUE	TRUE	FALSE	FALSE	TRUE	FALSE	FALSE	0	0
rs30266	chr5	104636655	104636656	1f	0,22271	FALSE	TRUE	FALSE	FALSE	TRUE	FALSE	FALSE	0	0
rs33817	chr5	104642624	104642625	1f	0,59058	TRUE	FALSE	TRUE	TRUE	TRUE	FALSE	FALSE	1,94	0
rs40465	chr5	104646024	104646025	1b	0,69	TRUE	TRUE	TRUE	TRUE	TRUE	FALSE	FALSE	1,77	0
rs416223	chr5	104655774	104655775	1f	0,22271	FALSE	TRUE	FALSE	FALSE	TRUE	FALSE	FALSE	0	0
rs325485	chr5	104659666	104659667	1f	0,55436	TRUE	TRUE	FALSE	FALSE	TRUE	FALSE	FALSE	0	0
rs325481	chr5	104665050	104665051	7	0,51392	FALSE	FALSE	FALSE	FALSE	TRUE	FALSE	FALSE	0	0
rs325500	chr5	104670965	104670966	1b	0,43617	TRUE	TRUE	TRUE	TRUE	TRUE	FALSE	FALSE	0,65	0
rs325501	chr5	104671731	104671732	7	0,51392	FALSE	FALSE	FALSE	FALSE	TRUE	FALSE	FALSE	0	0
rs325502	chr5	104672431	104672432	7	0,51392	FALSE	FALSE	FALSE	FALSE	TRUE	FALSE	FALSE	0	0
rs325506	chr5	104676601	104676602	1f	0,22271	FALSE	TRUE	FALSE	FALSE	TRUE	FALSE	FALSE	0	0
rs21126	chr5	104678080	104678081	1f	0,55324	TRUE	FALSE	FALSE	FALSE	TRUE	FALSE	FALSE	0	0
rs396755	chr5	104686537	104686538	7	0,51392	FALSE	FALSE	FALSE	FALSE	TRUE	FALSE	FALSE	0	0
rs410915	chr5	104698438	104698439	1f	0,55436	TRUE	TRUE	FALSE	FALSE	TRUE	FALSE	FALSE	0	0
rs12055234	chr5	104702058	104702059	1f	0,55324	TRUE	FALSE	FALSE	FALSE	TRUE	FALSE	FALSE	0	0
rs325521	chr5	104707454	104707455	1f	0,55324	TRUE	FALSE	FALSE	FALSE	TRUE	FALSE	FALSE	0	0
rs325523	chr5	104709684	104709685	6	0,07348	FALSE	FALSE	TRUE	TRUE	TRUE	FALSE	FALSE	0,02	0
rs325528	chr5	104712888	104712889	1f	0,9	FALSE	TRUE	TRUE	FALSE	TRUE	FALSE	FALSE	0,82	0
rs161645	chr5	104734215	104734216	1f	0,55436	TRUE	TRUE	FALSE	FALSE	TRUE	FALSE	FALSE	0	0
rs6421926	chr5	104739428	104739429	6	0,78688	FALSE	FALSE	TRUE	FALSE	TRUE	FALSE	FALSE	1,94	0
rs60271	chr5	104742531	104742532	7	0,51392	FALSE	FALSE	FALSE	FALSE	TRUE	FALSE	FALSE	0	0
rs323509	chr5	104746477	104746478	6	0,0475	FALSE	FALSE	TRUE	TRUE	TRUE	FALSE	FALSE	0,46	0
rs9342783	chr6	70142600	70142601	1f	0,55324	TRUE	FALSE	FALSE	FALSE	TRUE	FALSE	FALSE	0	0
rs2296013	chr6	70142948	70142949	7	0,51392	FALSE	FALSE	FALSE	FALSE	TRUE	FALSE	FALSE	0	0
rs6935524	chr6	70143875	70143876	1f	0,22271	FALSE	TRUE	FALSE	FALSE	TRUE	FALSE	FALSE	0	0
rs9364074	chr6	70144290	70144291	7	0,51392	FALSE	FALSE	FALSE	FALSE	TRUE	FALSE	FALSE	0	0
rs9454993	chr6	70148036	70148037	1f	0,55436	TRUE	TRUE	FALSE	FALSE	TRUE	FALSE	FALSE	0	0
rs2025286	chr6	70148808	70148809	7	0,51392	FALSE	FALSE	FALSE	FALSE	TRUE	FALSE	FALSE	0	0
rs2025284	chr6	70148890	70148891	7	0,51392	FALSE	FALSE	FALSE	FALSE	TRUE	FALSE	FALSE	0	0
rs2049604	chr7	114350296	114350297	2b	0,62064	TRUE	TRUE	TRUE	TRUE	FALSE	FALSE	FALSE	1,94	0
rs66571810	chr7	114381814	114381815	7	0,51392	FALSE	FALSE	FALSE	FALSE	TRUE	FALSE	FALSE	0	0
rs12537376	chr7	114384997	114384998	6	0,09069	FALSE	FALSE	TRUE	FALSE	FALSE	FALSE	FALSE	0,28	0
rs12536335	chr7	114403103	114403104	1f	0,55436	TRUE	TRUE	FALSE	FALSE	TRUE	FALSE	FALSE	0	0
rs2030915	chr7	114406313	114406314	1f	0,55436	TRUE	TRUE	FALSE	FALSE	TRUE	FALSE	FALSE	0	0

rs7795397	chr7	114407084	114407085	1f	0,41583	TRUE	FALSE	TRUE	FALSE	TRUE	FALSE	FALSE	1,19	0
rs8180817	chr7	114407486	114407487	1f	0,37526	FALSE	TRUE	TRUE	FALSE	TRUE	FALSE	FALSE	1,96	0
rs12533005	chr7	114415999	114416000	1b	0,3284	TRUE	TRUE	TRUE	TRUE	TRUE	FALSE	FALSE	1,81	0
rs2894699	chr7	114419100	114419101	7	0,51392	FALSE	FALSE	FALSE	FALSE	TRUE	FALSE	FALSE	0	0
rs7785701	chr7	114429480	114429481	1f	0,22271	FALSE	TRUE	FALSE	FALSE	TRUE	FALSE	FALSE	0	0
rs1476535	chr7	114430979	114430980	7	0,51392	FALSE	FALSE	FALSE	FALSE	TRUE	FALSE	FALSE	0	0
rs10268637	chr7	114434201	114434202	1b	0,95	TRUE	TRUE	TRUE	TRUE	TRUE	FALSE	FALSE	1,73	0
rs28534444	chr7	114435084	114435085	1f	0,22271	FALSE	TRUE	FALSE	FALSE	TRUE	FALSE	FALSE	0	0
rs10246733	chr7	114435630	114435631	1b	0,49233	TRUE	TRUE	TRUE	TRUE	TRUE	FALSE	FALSE	0,58	0
rs10280045	chr7	114436338	114436339	7	0,51392	FALSE	FALSE	FALSE	FALSE	TRUE	FALSE	FALSE	0	0
rs2014265	chr7	114440280	114440281	6	0,10806	FALSE	FALSE	TRUE	FALSE	TRUE	FALSE	FALSE	0,25	0
rs10261780	chr7	114442438	114442439	1f	0,55436	TRUE	TRUE	FALSE	FALSE	TRUE	FALSE	FALSE	0	0
rs10228494	chr7	114443494	114443495	7	0,51392	FALSE	FALSE	FALSE	FALSE	TRUE	FALSE	FALSE	0	0
rs10262192	chr7	114451697	114451698	7	0,51392	FALSE	FALSE	FALSE	FALSE	TRUE	FALSE	FALSE	0	0
rs10262103	chr7	114451788	114451789	7	0,51392	FALSE	FALSE	FALSE	FALSE	TRUE	FALSE	FALSE	0	0
rs10231382	chr7	114456790	114456791	1f	0,22271	FALSE	TRUE	FALSE	FALSE	TRUE	FALSE	FALSE	0	0
rs10228327	chr7	114464333	114464334	1f	0,29395	FALSE	TRUE	TRUE	TRUE	TRUE	FALSE	FALSE	1,94	0
rs727644	chr7	114469293	114469294	1f	0,22271	FALSE	TRUE	FALSE	FALSE	TRUE	FALSE	FALSE	0	0
rs4727799	chr7	114470512	114470513	7	0,51392	FALSE	FALSE	FALSE	FALSE	TRUE	FALSE	FALSE	0	0
rs2396751	chr7	114474993	114474994	1f	0,55436	TRUE	TRUE	FALSE	FALSE	TRUE	FALSE	FALSE	0	0
rs2189011	chr7	114475212	114475213	6	0,24183	FALSE	FALSE	TRUE	FALSE	TRUE	FALSE	FALSE	1,72	0
rs7783012	chr7	114476825	114476826	7	0,51392	FALSE	FALSE	FALSE	FALSE	TRUE	FALSE	FALSE	0	0
rs2189010	chr7	114479374	114479375	7	0,51392	FALSE	FALSE	FALSE	FALSE	TRUE	FALSE	FALSE	0	0
rs2106900	chr7	114482450	114482451	1f	0,60183	TRUE	FALSE	TRUE	FALSE	TRUE	FALSE	FALSE	1,93	0
rs2189008	chr7	114483252	114483253	1f	0,55324	TRUE	FALSE	FALSE	FALSE	TRUE	FALSE	FALSE	0	0
rs6974757	chr7	114484604	114484605	7	0,51392	FALSE	FALSE	FALSE	FALSE	TRUE	FALSE	FALSE	0	0
rs10249234	chr7	114489081	114489082	1f	0,22271	FALSE	TRUE	FALSE	FALSE	TRUE	FALSE	FALSE	0	0
rs1989903	chr7	114497884	114497885	1f	0,55324	TRUE	FALSE	FALSE	FALSE	TRUE	FALSE	FALSE	0	0
rs2040658	chr7	114498695	114498696	7	0,51392	FALSE	FALSE	FALSE	FALSE	TRUE	FALSE	FALSE	0	0
rs12113612	chr7	114499628	114499629	7	0,51392	FALSE	FALSE	FALSE	FALSE	TRUE	FALSE	FALSE	0	0
rs1015511	chr7	114502194	114502195	1f	0,55436	TRUE	TRUE	FALSE	FALSE	TRUE	FALSE	FALSE	0	0
rs10266297	chr7	114503351	114503352	1f	0,66917	TRUE	FALSE	TRUE	FALSE	TRUE	FALSE	FALSE	1,41	0
rs9649368	chr7	114503595	114503596	5	0,54974	TRUE	FALSE	TRUE	FALSE	FALSE	FALSE	FALSE	0,76	0
rs6466488	chr7	114505469	114505470	7	0,51392	FALSE	FALSE	FALSE	FALSE	TRUE	FALSE	FALSE	0	0
rs10279936	chr7	114507161	114507162	6	0,72212	FALSE	FALSE	TRUE	FALSE	TRUE	FALSE	FALSE	1,92	0
rs2189015	chr7	114508187	114508188	1f	0,55436	TRUE	TRUE	FALSE	FALSE	TRUE	FALSE	FALSE	0	0
rs2396753	chr7	114508275	114508276	1f	0,55436	TRUE	TRUE	FALSE	FALSE	TRUE	FALSE	FALSE	0	0
rs9969232	chr7	114518898	114518899	1f	0,15686	FALSE	TRUE	TRUE	TRUE	TRUE	FALSE	FALSE	0,39	0
rs6969376	chr7	114520815	114520816	7	0,51392	FALSE	FALSE	FALSE	FALSE	TRUE	FALSE	FALSE	0	0
rs6980093	chr7	114522684	114522685	7	0,51392	FALSE	FALSE	FALSE	FALSE	TRUE	FALSE	FALSE	0	0
rs9332390	chr7	114531089	114531090	1f	0,55436	TRUE	TRUE	FALSE	FALSE	TRUE	FALSE	FALSE	0	0
rs10262462	chr7	114540006	114540007	1f	0,51167	TRUE	FALSE	TRUE	TRUE	TRUE	FALSE	FALSE	0,12	0
rs7458242	chr7	114544042	114544043	7	0,51392	FALSE	FALSE	FALSE	FALSE	TRUE	FALSE	FALSE	0	0
rs7794413	chr7	114552489	114552490	1f	0,55324	TRUE	FALSE	FALSE	FALSE	TRUE	FALSE	FALSE	0	0
rs1859100	chr7	114554559	114554560	1f	0,8313	TRUE	FALSE	TRUE	FALSE	TRUE	FALSE	FALSE	1,98	0
rs4355713	chr7	114560478	114560479	1f	0,22271	FALSE	TRUE	FALSE	FALSE	TRUE	FALSE	FALSE	0	0
rs7797335	chr7	114564073	114564074	6	0,04797	FALSE	FALSE	TRUE	FALSE	FALSE	FALSE	FALSE	0,81	0
rs17137124	chr7	114570758	114570759	7	0,51392	FALSE	FALSE	FALSE	FALSE	TRUE	FALSE	FALSE	0	0
rs10269986	chr7	114571391	114571392	7	0,51392	FALSE	FALSE	FALSE	FALSE	TRUE	FALSE	FALSE	0	0
rs2189012	chr7	114571856	114571857	7	0,51392	FALSE	FALSE	FALSE	FALSE	TRUE	FALSE	FALSE	0	0
rs1229762	chr7	114578526	114578527	6	0,84117	FALSE	FALSE	TRUE	FALSE	TRUE	FALSE	FALSE	1,89	0

rs1229761	chr7	114583667	114583668	7	0,51392	FALSE	FALSE	FALSE	FALSE	TRUE	FALSE	FALSE	0	0
rs1229760	chr7	114584107	114584108	1f	0,22271	FALSE	TRUE	FALSE	FALSE	TRUE	FALSE	FALSE	0	0
rs1229758	chr7	114589083	114589084	7	0,51392	FALSE	FALSE	FALSE	FALSE	TRUE	FALSE	FALSE	0	0
rs10230558	chr7	114605693	114605694	7	0,51392	FALSE	FALSE	FALSE	FALSE	TRUE	FALSE	FALSE	0	0
rs12705966	chr7	114608795	114608796	6	0,79772	FALSE	FALSE	TRUE	TRUE	TRUE	FALSE	FALSE	2	0
rs11983431	chr7	114627940	114627941	7	0,51392	FALSE	FALSE	FALSE	FALSE	TRUE	FALSE	FALSE	0	0
rs2045292	chr7	114647060	114647061	7	0,51392	FALSE	FALSE	FALSE	FALSE	TRUE	FALSE	FALSE	0	0

Supplementary Table 7.

CHR	POS	ID	REF	ALT	AF_AFR	AF_AMR	AF_EAS	AF_EUR	AF_SAS	Gpval_AFR	Gpval_AMR	Gpval_EAS	Gpval_EUR	Gpval_SAS
4	31088873	chr4:3108887	A	G	0,388889	0,256135	0,181818	0,152815	0,5	0,51554569	1,2748E-05	0,41559028	0,22360884	0,99896004
4	124474820	chr4:1244748	T	C	0,416667	0,376254	0,166667	0,416471	0,555556	0,73976408	0,03630375	0,98836269	4,054E-05	0,98123006
7	147994354	chr7:1479943	A	G	0,534884	0,463487	0,4	0,314363	0,75	0,14426359	0,22789184	0,98143329	4,1439E-05	0,99140069
8	86116029	chr8:8611602	A	G	0,254237	0,14214	0,1	0,127404	0,111111	0,75364963	0,98422111	0,98886441	3,685E-05	0,98922765
9	3595503	chr9:3595503	A	T	0,219512	0,289941	0,5	0,231806	0,5	0,87678398	7,4301E-06	0,99901945	0,62215141	0,99912663
9	3595674	chr9:3595674	C	T	0,219512	0,289941	0,5	0,231806	0,5	0,87678398	7,4301E-06	0,99901945	0,62215141	0,99912663
9	3601355	chr9:3601355	A	G	0,219512	0,29142	0,5	0,239892	0,5	0,89300361	3,6364E-05	0,99905972	0,2715023	0,99914985
9	3602811	chr9:3602811	C	T	0,219512	0,288462	0,5	0,231806	0,5	0,91310887	1,3899E-05	0,99903541	0,36489888	0,99914433
9	3613712	chr9:3613712	T	G	0,219512	0,294379	0,5	0,231806	0,5	0,90412968	1,6216E-05	0,99904134	0,55539265	0,99914557
9	3615879	chr9:3615879	G	T	0,219512	0,288462	0,5	0,221024	0,5	0,89134336	1,1958E-05	0,99903406	0,61703269	0,99913648
9	3616140	chr9:3616140	G	C	0,219512	0,288462	0,5	0,221024	0,5	0,89134336	1,1958E-05	0,99903406	0,61703269	0,99913648
9	3617602	chr9:3617602	A	G	0,219512	0,292899	0,5	0,231806	0,5	0,9088874	2,4061E-05	0,99904816	0,53651499	0,99914946
9	3618438	chr9:3618438	T	C	0,219512	0,29142	0,5	0,22372	0,5	0,89117728	1,533E-05	0,99903898	0,69121117	0,99913872
9	3620109	chr9:3620109	C	G	0,219512	0,289941	0,5	0,221024	0,5	0,90144177	1,4031E-05	0,99903601	0,51821973	0,99914022
9	3623111	chr9:3623111	A	G	0,219512	0,294379	0,5	0,231806	0,5	0,9096417	2,6018E-05	0,99904892	0,53480057	0,99915082
9	3623117	chr9:3623117	T	TC	0,219512	0,294379	0,5	0,231806	0,5	0,9096417	2,6018E-05	0,99904892	0,53480057	0,99915082
9	3634981	chr9:3634981	C	T	0,219512	0,288462	0,5	0,212938	0,5	0,84292065	1,22E-05	0,99903804	0,63728167	0,99912242
9	101937294	chr9:1019372	C	A	0,509804	0,385993	0,392857	0,472222	0,666667	0,53571559	0,01904458	0,71789173	2,8308E-05	0,98019437
9	101948060	chr9:1019480	G	C	0,490196	0,387622	0,392857	0,472222	0,666667	0,296577	0,01612252	0,74625896	1,9157E-05	0,98023108
9	101950580	chr9:1019505	A	C	0,490196	0,389251	0,392857	0,477273	0,666667	0,26350849	0,01549093	0,78171854	1,735E-05	0,98027916
9	101950772	chr9:1019507	T	A	0,490196	0,389251	0,392857	0,477273	0,666667	0,26350849	0,01549093	0,78171854	1,735E-05	0,98027916
10	112457495	chr10:112457	G	A	0,428571	0,222701	0,333333	0,428161	0,333333	0,11411491	3,731E-05	0,99084996	0,08414616	0,98592017
10	112462144	chr10:112462	G	C	0,333333	0,189655	0,333333	0,405172	0,333333	0,75328858	3,858E-05	0,99057164	0,18968701	0,98570367
10	112463185	chr10:112463	G	A	0,309524	0,189655	0,333333	0,405172	0,333333	0,49442441	3,4952E-05	0,99057222	0,17129297	0,98573169
10	112464304	chr10:112464	A	G	0,333333	0,189655	0,333333	0,405172	0,333333	0,75328858	3,858E-05	0,99057164	0,18968701	0,98570367
10	112464743	chr10:112464	C	G	0,190476	0,173851	0,333333	0,318966	0,333333	0,32110708	4,2994E-05	0,99057516	0,30595144	0,98575833
10	112465451	chr10:112465	T	G	0,333333	0,189655	0,333333	0,405172	0,333333	0,75328858	3,858E-05	0,99057164	0,18968701	0,98570367
10	112466835	chr10:112466	T	TG	0,333333	0,189655	0,333333	0,405172	0,333333	0,75328858	3,858E-05	0,99057164	0,18968701	0,98570367
10	112467502	chr10:112467	G	A	0,333333	0,189655	0,333333	0,405172	0,333333	0,75328858	3,858E-05	0,99057164	0,18968701	0,98570367
10	112467683	chr10:112467	T	A	0,190476	0,173851	0,333333	0,318966	0,333333	0,32110708	4,2994E-05	0,99057516	0,30595144	0,98575833
10	112468685	chr10:112468	T	C	0,190476	0,173851	0,333333	0,321839	0,333333	0,31621204	3,9384E-05	0,9905729	0,2237441	0,98575774
10	112477106	chr10:112477	A	G	0,190476	0,176724	0,333333	0,316092	0,333333	0,31124377	3,7383E-05	0,99056994	0,15565141	0,98575827
10	112478370	chr10:112478	T	C	0,261905	0,182471	0,333333	0,321839	0,333333	0,73458942	3,6834E-05	0,99056932	0,20196765	0,98572116
10	112488148	chr10:112488	A	G	0,190476	0,168103	0,333333	0,310345	0,333333	0,34297088	3,9381E-05	0,99056219	0,34790656	0,985751
13	101776074	chr13:101776	G	A	0,162162	0,298571	0,5	0,174286	0,666667	0,97760433	0,11659702	0,99962956	4,3835E-05	0,98637593
13	101776079	chr13:101776	C	T	0,135135	0,298571	0,5	0,174286	0,666667	0,52239461	0,12144475	0,99962581	4,4327E-05	0,98636151
13	101777315	chr13:101777	G	A	0,162162	0,3	0,5	0,174286	0,666667	0,96643578	0,14598359	0,99964766	4,5825E-05	0,98638404
13	101777494	chr13:101777	A	G	0,135135	0,3	0,5	0,174286	0,666667	0,51954187	0,15214726	0,99964368	4,6413E-05	0,98636854
13	101777913	chr13:101777	G	A	0,135135	0,3	0,5	0,174286	0,666667	0,51954187	0,15214726	0,99964368	4,6413E-05	0,98636854
13	101778686	chr13:101778	G	T	0,135135	0,298571	0,5	0,174286	0,666667	0,52239461	0,12144475	0,99962581	4,4327E-05	0,98636151

13	101778863	chr13:101778 GA	G	0,135135	0,297143	0,5	0,168571	0,666667	0,52465157	0,12720007	0,99963143	2,4967E-05	0,98637534
13	101779001	chr13:101779 G	C	0,135135	0,297143	0,5	0,171429	0,666667	0,51708936	0,18306262	0,9996607	4,4405E-05	0,98637425
16	249981	chr16:249981 T	C	0,020833	0,134801	0,166667	0,065359	0,2	0,99093481	4,2316E-05	0,98498315	0,58528012	0,99212234
16	250178	chr16:250178 A	G	0,208333	0,148556	0,333333	0,078431	0,2	0,14602799	3,2358E-05	0,98191956	0,87531669	0,99215275
16	250642	chr16:250642 G	A	0,333333	0,148556	0,333333	0,081699	0,2	0,23299568	3,1091E-05	0,98193706	0,91281897	0,99216127
16	250697	chr16:250697 T	A	0,333333	0,148556	0,333333	0,081699	0,2	0,23450673	3,1805E-05	0,98194376	0,75575344	0,99214517
16	87957445	chr16:879574 A	C	0,688889	0,388128	0,625	0,34748	0,4	0,27380105	3,3493E-05	0,98312163	0,08618438	0,77665623
16	87962269	chr16:879622 T	C	0,688889	0,388128	0,625	0,342175	0,4	0,26931131	2,4596E-05	0,98309217	0,08498104	0,77785777
16	87969230	chr16:879692 TTTG	T	0,666667	0,392694	0,625	0,339523	0,4	0,08613339	1,3611E-05	0,98257838	0,06143096	0,76236709
16	87971624	chr16:879716 C	G	0,6	0,342466	0,625	0,297082	0,2	0,76329578	4,5279E-05	0,9842865	0,97724008	0,99114189
16	87979423	chr16:879794 C	A	0,688889	0,388128	0,625	0,342175	0,4	0,27116959	2,8041E-05	0,98308988	0,08656614	0,77738988
16	87983645	chr16:879836 C	G	0,688889	0,38965	0,625	0,342175	0,4	0,27821354	3,7352E-05	0,98312206	0,09166349	0,78035101
16	87987204	chr16:879872 A	G	0,688889	0,38965	0,625	0,33687	0,4	0,25532459	2,5591E-05	0,98303334	0,05519636	0,76586742
16	87994371	chr16:879943 A	G	0,6	0,348554	0,625	0,286472	0,2	0,86183343	4,3345E-05	0,98394736	0,81344479	0,99112314
16	87994524	chr16:879945 T	C	0,6	0,348554	0,625	0,286472	0,2	0,86183343	4,3345E-05	0,98394736	0,81344479	0,99112314
16	87995028	chr16:879950 T	C	0,6	0,348554	0,625	0,286472	0,2	0,86183343	4,3345E-05	0,98394736	0,81344479	0,99112314
16	87995037	chr16:879950 TG	T	0,6	0,348554	0,625	0,286472	0,2	0,86183343	4,3345E-05	0,98394736	0,81344479	0,99112314
16	87997882	chr16:879978 T	C	0,577778	0,348554	0,625	0,286472	0,2	0,8438008	4,2553E-05	0,98393459	0,81108219	0,99112297
16	88015046	chr16:880150 C	T	0,088889	0,187215	0,125	0,119363	0,4	0,3287263	2,9926E-05	0,98853439	0,75032868	0,98026535
16	88027383	chr16:880273 G	C	0,066667	0,170472	0,125	0,111406	0,2	0,20600568	1,4035E-05	0,98851869	0,4970957	0,98734119
16	88027970	chr16:880279 C	A	0,066667	0,170472	0,125	0,111406	0,2	0,20600568	1,4035E-05	0,98851869	0,4970957	0,98734119
16	88038673	chr16:880386 A	G	0,533333	0,39726	0,625	0,334218	0,4	0,01975959	1,9977E-05	0,98177287	0,11656006	0,98766687
16	88040274	chr16:880402 A	G	0,688889	0,39726	0,625	0,334218	0,6	0,23658818	3,9553E-05	0,98307495	0,143259	0,84684764
18	43656089	chr18:436560 G	A	0,105263	0,330565	0,25	0,281991	0,333333	0,31436962	1,1087E-05	0,97190431	0,29128591	0,9921072
18	43657313	chr18:436573 C	T	0,105263	0,330565	0,25	0,281991	0,333333	0,31436962	1,1087E-05	0,97190431	0,29128591	0,9921072
18	43657414	chr18:436574 T	G	0,105263	0,330565	0,25	0,281991	0,333333	0,31436962	1,1087E-05	0,97190431	0,29128591	0,9921072
18	43662107	chr18:436621 G	T	0,105263	0,330565	0,25	0,281991	0,333333	0,31436962	1,1087E-05	0,97190431	0,29128591	0,9921072
18	43663830	chr18:436638 A	G	0,140351	0,325581	0,125	0,270142	0,333333	0,13813403	2,3139E-06	0,9898534	0,1485154	0,99486037
18	43664868	chr18:436648 C	T	0,140351	0,325581	0,125	0,270142	0,333333	0,13813403	2,3139E-06	0,9898534	0,1485154	0,99486037
18	43666216	chr18:436662 C	G	0,140351	0,325581	0,125	0,251185	0,333333	0,13645091	2,616E-06	0,98985868	0,18538217	0,99486259
18	43666700	chr18:436667 T	G	0,140351	0,325581	0,125	0,267773	0,333333	0,1388986	2,0006E-06	0,98985087	0,10752577	0,99485244
18	43667287	chr18:436672 T	A	0,140351	0,325581	0,125	0,270142	0,333333	0,13813403	2,3139E-06	0,9898534	0,1485154	0,99486037
20	36956238	chr20:369562 G	A	0,163636	0,377099	0,5	0,165312	0,181818	0,09650552	3,8058E-05	0,9815814	0,83478858	0,94689334
20	36956696	chr20:369566 G	A	0,145455	0,377099	0,5	0,170732	0,181818	0,05586849	3,4321E-05	0,98159247	0,90851493	0,94771291
22	29221249	chr22:292212 GGGA	G	0,043478	0,409763	0,25	0,310249	0,4	0,99255004	2,0666E-05	0,99063685	0,12970052	0,98767822
22	29224243	chr22:292242 C	T	0,021739	0,433432	0,25	0,288089	0,4	0,99476574	3,508E-05	0,99061737	0,09369609	0,98767078

Supplementary Table 8.

Chr	Position	MarkerName	Allele_A	Allele_B	Weight	Zscore	P-value	Direction
12	55347958	chr12:553479	A	G	541	-5,02	5,178E-07	--
7	93366164	chr7:9336616	A	C	513	-5,018	5,224E-07	--
7	93366773	chr7:9336677	A	C	513	-5,018	5,224E-07	--
7	93372196	chr7:9337219	G	T	522	4,971	6,674E-07	++
7	93363677	chr7:9336367	T	C	516	-4,964	6,911E-07	--
7	93381066	chr7:9338106	C	G	528	-4,963	0,000000693	--
7	93384392	chr7:9338439	A	G	525	-4,945	7,609E-07	--
12	55339179	chr12:553391	G	T	525	4,939	7,854E-07	++
12	55344280	chr12:553442	G	C	525	-4,939	7,854E-07	--
12	55343184	chr12:553431	AGTCCTATG	A	525	4,939	7,854E-07	++
12	55337416	chr12:553374	T	A	525	4,939	7,854E-07	++
12	55336639	chr12:553366	T	C	525	-4,939	7,854E-07	--
12	55337706	chr12:553377	A	G	525	-4,939	7,854E-07	--
12	55343619	chr12:553436	A	C	525	-4,939	7,854E-07	--
12	55342455	chr12:553424	TA	T	526	4,935	7,999E-07	++
12	55340727	chr12:553407	G	A	528	4,916	8,854E-07	++
12	55350291	chr12:553502	C	CA	543	-4,911	0,000000906	--
12	55355436	chr12:553554	T	C	543	-4,908	9,207E-07	--
12	55350316	chr12:553503	T	C	543	-4,9	9,602E-07	--
12	55354420	chr12:553544	C	T	543	4,9	9,602E-07	++
12	55350926	chr12:553509	T	G	543	-4,9	9,602E-07	--
12	55353970	chr12:553539	C	T	543	4,9	9,602E-07	++
12	55351641	chr12:553516	T	C	543	-4,9	9,602E-07	--
12	55351935	chr12:553519	A	G	543	-4,9	9,602E-07	--
12	55355347	chr12:553553	T	G	545	-4,888	0,000001021	--
12	55343079	chr12:553430	C	T	527	4,887	0,000001023	++
12	55342875	chr12:553428	T	C	527	-4,887	0,000001023	--
12	55338187	chr12:553381	C	T	527	4,887	0,000001023	++
12	55342454	chr12:553424	G	A	527	4,887	0,000001023	++
12	55342878	chr12:553428	G	A	527	4,887	0,000001023	++
7	93383940	chr7:9338394	T	G	522	-4,887	0,000001023	--
12	55359652	chr12:553596	T	A	544	4,858	0,000001184	++
12	55357517	chr12:553575	T	C	544	-4,858	0,000001184	--
12	55355546	chr12:553555	A	G	544	-4,858	0,000001184	--
12	55364269	chr12:553642	T	C	544	-4,858	0,000001184	--
12	55356183	chr12:553561	T	A	544	4,858	0,000001184	++
12	55361240	chr12:553612	A	C	544	-4,858	0,000001184	--
12	55364234	chr12:553642	C	T	544	4,853	0,000001215	++
12	55361470	chr12:553614	A	G	545	-4,847	0,000001256	--
12	55361722	chr12:553617	C	A	545	4,847	0,000001256	++
12	55363912	chr12:553639	G	C	545	-4,847	0,000001256	--
12	55363596	chr12:553635	C	G	545	-4,847	0,000001256	--
12	55363825	chr12:553638	G	T	545	4,847	0,000001256	++
12	55355063	chr12:553550	A	G	544	-4,844	0,000001271	--
12	55363067	chr12:553630	T	C	546	-4,844	0,000001274	--
12	55350406	chr12:553504	GT	G	541	-4,826	0,000001394	--
7	93375371	chr7:9337537	CTT	C	515	-4,81	0,00000151	--
12	55414965	chr12:554149	C	T	541	4,794	0,000001634	++
12	55344456	chr12:553444	A	G	525	-4,763	0,000001912	--
7	93375756	chr7:9337575	T	A	526	4,721	0,000002345	++
7	93375919	chr7:9337591	T	C	525	-4,692	0,000002703	--
11	10289258	chr11:102892	T	A	525	4,681	0,000002857	++
12	55424287	chr12:554242	C	A	541	4,681	0,000002858	++
12	55420298	chr12:554202	C	G	541	-4,681	0,000002858	--
12	55417881	chr12:554178	A	G	541	-4,681	0,000002858	--
12	55343879	chr12:553438	A	AT	524	-4,678	0,000002903	--

7	93376729	chr7:9337672	G	A	525	4,645	0,0000034	++
7	93376828	chr7:9337682	G	A	527	4,614	0,000003953	++
7	93375231	chr7:9337523	G	A	521	4,601	0,000004199	++
12	55331313	chr12:553313	G	GA	524	-4,596	0,000004305	--
3	107026168	chr3:1070261	C	T	485	4,576	0,000004746	++
12	29576057	chr12:295760	T	C	506	-4,544	0,000005514	--
12	29575762	chr12:295757	G	A	506	4,544	0,000005514	++
12	29575767	chr12:295757	C	A	506	4,544	0,000005514	++
3	107037494	chr3:1070374	T	C	483	-4,527	0,00000599	--
12	55336163	chr12:553361	A	C	526	-4,518	0,000006231	--
12	55328527	chr12:553285	A	G	526	-4,518	0,000006231	--
12	55328952	chr12:553289	T	G	526	-4,518	0,000006231	--
12	55327950	chr12:553279	C	T	526	4,518	0,000006231	++
12	29577648	chr12:295776	A	G	510	-4,511	0,000006452	--
12	29574906	chr12:295749	C	T	510	4,511	0,000006452	++
12	29580222	chr12:295802	G	A	510	4,511	0,000006452	++
12	29575014	chr12:295750	C	T	510	4,511	0,000006452	++
12	29574968	chr12:295749	C	T	510	4,511	0,000006452	++
12	29575184	chr12:295751	T	A	510	4,511	0,000006452	++
12	29574835	chr12:295748	T	A	510	4,511	0,000006452	++
12	29580027	chr12:295800	T	C	510	-4,511	0,000006452	--
12	29578159	chr12:295781	C	A	510	4,511	0,000006452	++
12	29570395	chr12:295703	G	C	510	-4,511	0,000006452	--
12	29579851	chr12:295798	C	G	510	-4,511	0,000006452	--
12	29576837	chr12:295768	T	A	510	4,511	0,000006452	++
12	29576349	chr12:295763	C	T	510	4,511	0,000006452	++
12	29575236	chr12:295752	G	A	510	4,511	0,000006452	++
3	107032082	chr3:1070320	C	CT	484	-4,506	0,000006609	--
12	55327092	chr12:553270	C	G	528	-4,506	0,000006615	--
12	55335138	chr12:553351	A	G	527	-4,505	0,000006635	--
12	29586174	chr12:295861	C	T	509	4,482	0,000007385	++
12	55333173	chr12:553331	A	G	528	-4,481	0,000007439	--
12	55332666	chr12:553326	A	T	528	4,481	0,000007439	++
12	55330296	chr12:553302	A	C	528	-4,481	0,000007439	--
12	55342840	chr12:553428	G	GA	528	-4,481	0,000007439	--
12	55337394	chr12:553373	T	A	528	4,481	0,000007439	++
12	55337407	chr12:553374	G	T	528	4,481	0,000007439	++
12	55342714	chr12:553427	A	C	528	-4,481	0,000007439	--
12	55342766	chr12:553427	T	C	528	-4,481	0,000007439	--
12	55331197	chr12:553311	T	C	528	-4,481	0,000007439	--
12	55335127	chr12:553351	A	T	528	4,481	0,000007439	++
12	55331302	chr12:553313	G	T	528	4,481	0,000007439	++
4	101482523	chr4:1014825	G	T	245	4,475	0,000007624	+?
12	55385775	chr12:553857	A	T	540	4,47	0,000007833	++
12	55327444	chr12:553274	G	C	527	-4,46	0,000008201	--
12	55319822	chr12:553198	A	G	492	-4,46	0,000008208	--
13	37134265	chr13:371342	A	G	478	-4,453	0,000008482	--
12	55387745	chr12:553877	G	T	540	4,445	0,000008769	++
12	55325437	chr12:553254	A	T	526	4,442	0,000008926	++
12	55326158	chr12:553261	ATAT	A	526	4,442	0,000008926	++
12	55326393	chr12:553263	A	G	526	-4,442	0,000008926	--
12	55322659	chr12:553226	C	T	526	4,442	0,000008926	++
12	55322970	chr12:553229	G	T	526	4,442	0,000008926	++
12	55379053	chr12:553790	T	A	544	4,44	0,00000901	++
12	55351856	chr12:553518	A	C	544	-4,429	0,00000947	--
12	55354274	chr12:553542	G	A	544	4,429	0,00000947	++
12	55353066	chr12:553530	G	C	544	-4,429	0,00000947	--
12	55353482	chr12:553534	C	G	544	-4,429	0,00000947	--
12	55351367	chr12:553513	A	T	544	4,429	0,00000947	++

13	36989654	chr13:369896	C	T	459	4,423	0,000009737	++
4	16860541	chr4:1686054	C	T	465	-4,42	0,000009865	--
3	106969683	chr3:1069696	T	C	493	-4,416	0,00001005	--
12	55353781	chr12:553537	T	C	543	-4,416	0,00001007	--
4	101492469	chr4:1014924	C	G	246	-4,411	0,00001028	-?
4	101484796	chr4:1014847	C	T	246	4,411	0,00001028	+?
4	101504437	chr4:1015044	A	G	246	-4,411	0,00001028	-?
4	101488397	chr4:1014883	G	A	246	4,411	0,00001028	+?
4	101504382	chr4:1015043	G	A	246	4,411	0,00001028	+?
4	101503451	chr4:1015034	A	G	246	-4,411	0,00001028	-?
12	29571081	chr12:295710	G	C	510	-4,41	0,00001034	--
12	55325246	chr12:553252	C	T	524	4,406	0,00001051	++
12	55324996	chr12:553249	A	G	524	-4,406	0,00001051	--
12	55323187	chr12:553231	A	C	524	-4,406	0,00001051	--
12	55325408	chr12:553254	A	G	524	-4,406	0,00001051	--
12	55366067	chr12:553660	A	G	546	-4,406	0,00001051	--
12	55323626	chr12:553236	A	G	524	-4,406	0,00001051	--
12	55325306	chr12:553253	T	C	524	-4,406	0,00001051	--
4	101480630	chr4:1014806	T	G	245	-4,405	0,00001059	-?
12	55328251	chr12:553282	G	A	525	4,401	0,00001079	++
12	55364549	chr12:553645	A	G	544	-4,399	0,00001089	--
14	91362191	chr14:913621	A	G	502	-4,393	0,00001118	--
12	55376342	chr12:553763	T	C	544	-4,392	0,00001123	--
12	55466173	chr12:554661	G	T	542	4,391	0,00001126	++
3	106997379	chr3:1069973	G	A	491	4,39	0,00001136	++
12	55335002	chr12:553350	A	AT	527	-4,387	0,0000115	--
12	55400049	chr12:554000	C	G	540	-4,387	0,0000115	--
12	55322785	chr12:553227	T	A	526	4,387	0,00001152	++
12	55403700	chr12:554037	A	C	540	-4,382	0,00001179	--
12	55392639	chr12:553926	G	A	540	4,382	0,00001179	++
12	55409562	chr12:554095	T	C	540	-4,382	0,00001179	--
12	55398617	chr12:553986	T	A	540	4,382	0,00001179	++
12	55398314	chr12:553983	TTTC	T	540	4,382	0,00001179	++
12	55404180	chr12:554041	G	C	540	-4,382	0,00001179	--
12	55408807	chr12:554088	C	T	540	4,382	0,00001179	++
12	55403989	chr12:554039	C	T	540	4,382	0,00001179	++
12	55391807	chr12:553918	T	G	540	-4,382	0,00001179	--
12	55409345	chr12:554093	G	A	540	4,382	0,00001179	++
12	55374239	chr12:553742	C	T	545	4,379	0,0000119	++
12	55363933	chr12:553639	A	G	545	-4,379	0,0000119	--
12	55376017	chr12:553760	C	G	545	-4,379	0,0000119	--
12	55355438	chr12:553554	G	C	545	-4,379	0,0000119	--
12	55373526	chr12:553735	C	T	545	4,379	0,0000119	++
12	55370564	chr12:553705	G	A	545	4,379	0,0000119	++
12	55370625	chr12:553706	A	C	545	-4,379	0,0000119	--
12	55366184	chr12:553661	T	A	545	4,379	0,0000119	++
12	55372732	chr12:553727	A	G	545	-4,379	0,0000119	--
12	55361994	chr12:553619	T	C	545	-4,379	0,0000119	--
12	55373087	chr12:553730	G	A	545	4,379	0,0000119	++
7	126880044	chr7:1268800	G	C	518	-4,374	0,00001222	--
12	55323985	chr12:553239	T	C	526	-4,37	0,00001244	--
12	55356497	chr12:553564	G	A	544	4,366	0,00001263	++
12	55395345	chr12:553953	T	C	541	-4,363	0,00001281	--
12	55402936	chr12:554029	T	C	541	-4,363	0,00001281	--
12	55393072	chr12:553930	A	G	541	-4,363	0,00001281	--
12	55395259	chr12:553952	T	A	541	4,363	0,00001281	++
13	36913994	chr13:369139	G	A	542	-4,36	0,000013	--
12	55436280	chr12:554362	A	G	546	-4,354	0,00001337	--
12	55445847	chr12:554458	G	C	546	-4,354	0,00001337	--

12	55433085	chr12:554330	G	A	546	4,354	0,00001337	++
12	55449144	chr12:554491	A	G	546	-4,354	0,00001337	--
12	55441049	chr12:554410	T	G	546	-4,354	0,00001337	--
12	55443340	chr12:554433	C	A	546	4,354	0,00001337	++
12	55450933	chr12:554509	T	C	546	-4,354	0,00001337	--
12	55433923	chr12:554339	G	A	546	4,354	0,00001337	++
12	55434924	chr12:554349	C	T	546	4,354	0,00001337	++
12	55433951	chr12:554339	G	A	546	4,354	0,00001337	++
12	55447072	chr12:554470	G	A	546	4,354	0,00001337	++
12	55454702	chr12:554547	A	G	546	-4,354	0,00001337	--
12	55442801	chr12:554428	G	A	546	4,354	0,00001337	++
12	55450910	chr12:554509	A	G	546	-4,354	0,00001337	--
12	29585009	chr12:295850	C	G	504	-4,352	0,00001347	--
4	16860132	chr4:1686013	T	C	398	4,341	0,00001415	++
12	55438217	chr12:554382	C	G	544	-4,34	0,00001425	--
14	91362425	chr14:913624	C	T	502	4,34	0,00001428	++
7	153374029	chr7:1533740	T	G	545	-4,337	0,00001442	--
4	101498373	chr4:1014983	A	G	246	-4,333	0,00001468	-?
4	101495282	chr4:1014952	G	A	246	4,333	0,00001468	+?
4	101490990	chr4:1014909	T	C	246	-4,333	0,00001468	-?
4	101482978	chr4:1014829	A	G	245	-4,327	0,00001512	-?
4	101481713	chr4:1014817	G	A	245	4,327	0,00001512	+?
4	101478052	chr4:1014780	G	A	245	4,327	0,00001512	+?
11	10283548	chr11:102835	A	T	533	4,325	0,00001525	++
11	10286089	chr11:102860	T	A	533	4,325	0,00001525	++
12	55418856	chr12:554188	T	G	541	-4,32	0,00001561	--
14	91359251	chr14:913592	G	C	501	-4,319	0,00001568	--
12	55466989	chr12:554669	G	T	542	4,315	0,00001599	++
12	55474132	chr12:554741	G	A	542	4,315	0,00001599	++
12	55473506	chr12:554735	A	T	542	4,315	0,00001599	++
12	55467589	chr12:554675	A	T	542	4,315	0,00001599	++
12	55473756	chr12:554737	G	A	542	4,315	0,00001599	++
12	55469440	chr12:554694	C	G	542	-4,315	0,00001599	--
12	55475152	chr12:554751	G	T	542	4,315	0,00001599	++
12	29589031	chr12:295890	A	G	504	-4,312	0,00001618	--
3	106968030	chr3:1069680	AT	A	493	4,301	0,00001704	++
3	106968774	chr3:1069687	C	T	493	4,301	0,00001704	++
12	55387910	chr12:553879	T	G	536	-4,293	0,0000176	--
12	55474777	chr12:554747	T	C	541	-4,29	0,00001791	--
12	55472735	chr12:554727	T	C	541	-4,29	0,00001791	--
12	55476092	chr12:554760	T	C	541	-4,29	0,00001791	--
4	101511385	chr4:1015113	C	T	234	4,285	0,00001825	+?
12	55313542	chr12:553135	G	A	484	4,278	0,00001886	++
14	91355749	chr14:913557	TTAAG	T	513	4,271	0,00001945	++
12	55458502	chr12:554585	G	T	546	4,264	0,00002011	++
12	55459446	chr12:554594	G	A	546	4,264	0,00002011	++
11	10247828	chr11:102478	A	T	536	4,263	0,00002013	++
4	101463875	chr4:1014638	T	G	244	-4,242	0,00002217	-?
4	101464975	chr4:1014649	C	G	244	-4,242	0,00002217	-?
4	101470298	chr4:1014702	C	G	243	-4,238	0,00002251	-?
12	55383142	chr12:553831	G	A	542	4,237	0,0000227	++
9	32911523	chr9:3291152	T	TA	521	4,225	0,0000239	++
4	101460780	chr4:1014607	T	C	243	-4,225	0,00002391	-?
12	55200675	chr12:552006	A	G	463	-4,225	0,00002395	--
12	55178868	chr12:551788	C	G	461	-4,223	0,00002406	--
4	124459134	chr4:1244591	G	C	533	4,221	0,00002431	++
12	29582837	chr12:295828	T	C	506	-4,217	0,00002476	--
7	51010179	chr7:5101017	C	T	255	-4,215	0,00002498	-?
7	93382694	chr7:9338269	C	T	509	-4,213	0,00002521	--

7	93384812	chr7:9338481	G	A	509	-4,213	0,00002521	--
12	55348257	chr12:553482	G	A	543	4,21	0,00002548	++
12	132493112	chr12:132493	G	A	527	4,21	0,00002555	++
4	101510296	chr4:1015102	T	C	234	-4,207	0,0000259	-?
4	101509548	chr4:1015095	T	C	234	-4,207	0,0000259	-?
12	132589731	chr12:132589	G	A	471	4,201	0,00002655	++
17	9810188	chr17:981018	G	A	542	-4,2	0,0000267	--
12	132483963	chr12:132483	G	A	546	4,19	0,00002786	++
7	93371778	chr7:9337177	G	A	502	-4,19	0,00002788	--
4	162184198	chr4:1621841	T	C	536	-4,188	0,00002819	--
7	93372882	chr7:9337288	G	A	546	-4,185	0,00002853	--
7	126884159	chr7:1268841	C	T	519	4,18	0,00002914	++
12	29576223	chr12:295762	T	C	497	-4,177	0,00002959	--
7	93380576	chr7:9338057	G	A	543	4,176	0,0000297	++
12	132493708	chr12:132493	C	T	526	4,175	0,00002975	++
7	93371232	chr7:9337123	C	T	501	-4,173	0,00003004	--
12	55225164	chr12:552251	A	G	475	-4,169	0,0000306	--
4	124459347	chr4:1244593	G	A	533	-4,168	0,0000308	--
7	93367559	chr7:9336755	G	A	494	-4,165	0,00003117	--
7	93387119	chr7:9338711	G	A	497	-4,164	0,00003134	--
4	101467784	chr4:1014677	C	A	244	4,163	0,00003144	+?
12	55393527	chr12:553935	G	A	538	4,163	0,00003147	++
7	93373204	chr7:9337320	C	T	514	-4,154	0,00003267	--
4	177676467	chr4:1776764	T	G	486	-4,154	0,00003271	--
4	101454897	chr4:1014548	A	G	244	-4,147	0,00003368	-?
4	101454806	chr4:1014548	T	G	244	-4,147	0,00003368	-?
13	36991644	chr13:369916	TG	T	461	4,14	0,00003472	++
13	36988074	chr13:369880	T	C	461	-4,14	0,00003472	--
7	93383571	chr7:9338357	C	A	507	-4,134	0,00003566	--
6	31515681	chr6:3151568	G	A	542	4,119	0,00003801	++
4	149115325	chr4:1491153	A	T	486	-4,119	0,00003808	--
4	101449389	chr4:1014493	T	C	240	-4,118	0,00003825	-?
4	175002121	chr4:1750021	T	G	533	-4,111	0,00003931	--
4	175003582	chr4:1750035	C	T	533	4,111	0,00003931	++
15	61115195	chr15:611151	C	T	545	-4,11	0,00003959	--
5	73151270	chr5:7315127	T	A	502	4,103	0,00004071	++
5	73152191	chr5:7315219	T	A	502	4,103	0,00004071	++
10	128042273	chr10:128042	A	T	436	4,103	0,00004082	++
4	96472443	chr4:9647244	G	GCTGATACTC	532	4,1	0,00004126	++
4	174992647	chr4:1749926	C	T	545	4,092	0,00004271	++
4	124467776	chr4:1244677	G	A	530	-4,073	0,00004636	--
10	110712659	chr10:110712	C	T	537	4,072	0,00004662	++
4	101450675	chr4:1014506	T	C	243	-4,071	0,00004681	-?
9	115077297	chr9:1150772	T	C	526	-4,068	0,00004742	--
7	153368448	chr7:1533684	C	T	522	4,062	0,00004876	++
